# Increasing obsidian diversity during the Chalcolithic Period at Yeghegis-1 Rockshelter (Armenia) reveals shifts in land use and social networks

**DOI:** 10.1038/s41598-024-59661-9

**Published:** 2024-04-25

**Authors:** Ellery Frahm, Mariam Saribekyan, Satenik Mkrtchyan, Laura Furquim, Ara Avagyan, Lilit Sahakyan, Karen Azatyan, Patrick Roberts, Ricardo Fernandes, Levon Yepiskoposyan, Noel Amano, Mariya Antonosyan

**Affiliations:** 1 Council on Archaeological Studies, Department of Anthropology, Yale University, New Haven, USA; 2 Anthropology Division, Peabody Museum of Natural History, Yale University, New Haven, USA; 3 Institute of Archaeology and Ethnography, National Academy of Sciences, Yerevan, Republic of Armenia; 4 Institute of Molecular Biology, National Academy of Sciences, Yerevan, Republic of Armenia; 5 Department of Archaeology, Max Planck Institute of Geoanthropology, Jena, Germany; 6 Institute of Geological Sciences, National Academy of Sciences, Yerevan, Republic of Armenia; 7 Yeghegnadzor Regional Museum, Yeghegnadzor, Republic of Armenia; 8 Arne Faculty of Arts, Masaryk University, Brno, Czech Republic; 9 Department of Bioarchaeology, Faculty of Archaeology, University of Warsaw, Warsaw, Poland

**Keywords:** Behavioural ecology, Environmental social sciences

## Abstract

The newly excavated rockshelter of Yeghegis-1 in Armenia reflects an occupation of five centuries, as attested by radiocarbon dates from ∼ 4100 to 4000 cal BCE in the lowest layer to ∼ 3600–3500 cal BCE at the top. It is a partially collapsed cave in which pastoralists, we hypothesize, wintered with their herds. The stone tool assemblage is predominantly obsidian (92.1%), despite the shelter being > 60 km on foot from the nearest sources. We use obsidian sourcing to investigate two purported trends in the Southern Caucasus during the Chalcolithic Period: (1) occupation of more varied high-altitude environments and (2) more expansive social networks. Our data show both trends were dynamic phenomena. There was a greater balance in use of the nearest pasturelands over time, perhaps linked to risk management and/or resource sustainability. During later occupations, artifacts from distant sources reveal more extensive connections. This increase in connectivity likely played a central role in the shifts in societal complexity that gave rise to widely shared material culture throughout the Armenian Highlands around the start of the Early Bronze Age. In such a model, greater social connectivity becomes a key mechanism for, rather than a product of, the spread of cultural and/or technological innovations.

## Introduction

The Chalcolithic Period of the Southern Caucasus (5000–3500 BCE) corresponds to a phase of increased copper production alongside the continued use of lithic technology, chronologically situated between the agricultural settlements of the Neolithic Period and the far-reaching Kura-Araxes material culture “package” of the Early Bronze Age. Despite the relevance of this time period for understanding the development of social complexity across the Southern Caucasus, relatively little is known about these transformations during the Chalcolithic in this region. Sagona^1^ notes that, in the 20th century, the Chalcolithic “was an elusive period, characterized by fuzzy concepts, multiple traditions, and an array of patchy evidence weakly glued together by a small number of radiocarbon readings” from Soviet-era excavations. Work of that time, as described by Lyonnet^2^, was “extremely abundant and fundamental but often imprecise” and lacked dates, hindering the potential to make chronological correlations among sites. In contrast, the 21st century has seen a shift toward scientific collaboration between national and international research centers (e.g., the collaborative work that we report here). Collaboration at the Chalcolithic site of Areni-1 Cave, for example, has led to detailed chronological^3^, geoarchaeological^4^, botanical^5^, and faunal^6^ results. Recent excavations have yielded a wealth of new data, much of it at odds with the older studies. For decades, Chalcolithic sites found in the Southern Caucasus were assigned to one of two archaeological material cultures—either the Sioni or Chaff-Faced cultural horizons—on the basis of ceramic types^1^. Findings at the archaeological site of Mentesh Tepe, however, indicate that these “cultures” have been erroneously construed: Sioni ceramics seem to have been domestic cooking ware, whereas Chaff-Faced ceramics were common ware, not made or used by a distinct community^7^. Consequently, much about the Chalcolithic Period remains up for debate, while recent studies have sought to address some of the many outstanding questions (e.g., Getahovit-2 Cave^8–11^).

Sagona^1^ synthesizes the findings from Southern Caucasus archaeological studies, focusing on the results of 21st-century excavations (e.g., Aratashen^12^, Godedzor^13^, Mentesh Tepe^14^, Ovçular Tepesi^15,16^). Regarding the Chalcolithic, he proposes that three shifts occurred in land-use practices relative to the preceding Neolithic Period: (1) more diverse strategies of subsistence (i.e., pastoralism, agriculture, hunting) and settlement (i.e., variable occupation, from year-round villages to seasonal camps; open-air and cave sites); (2) occupation of more varied environments, especially those at higher altitudes (i.e., a shift from the alluvial plains to the highlands), and, in turn, access to the corresponding resources, including pastures; and (3) more expansive social networks, as shown by farther resource transports. Sagona^1^ cautions, however, that further research is necessary to substantiate these seeming shifts in land use and to consider the potential interplays among them, such as the role that transhumance played (or did not) in the observed trends.

**Figure 1:**
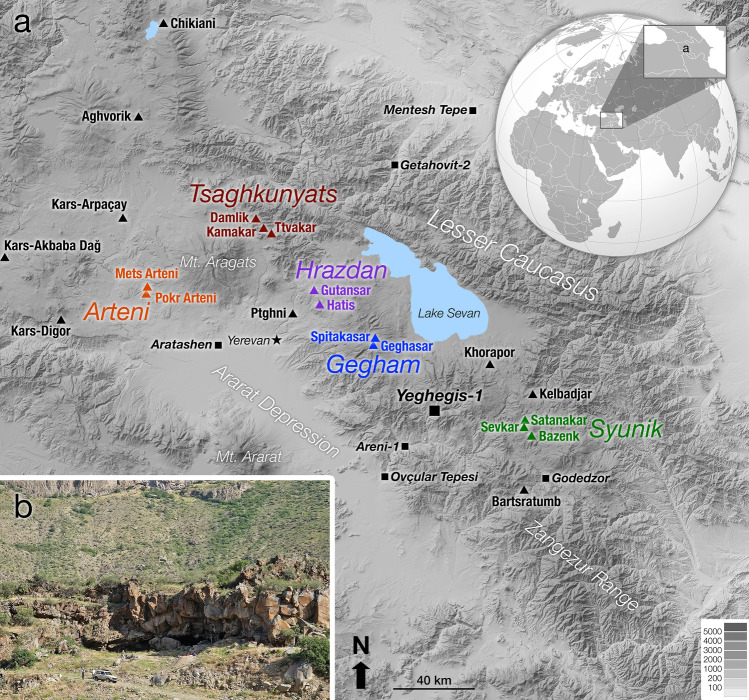
(**a**) Digital elevation model (DEM) with the locations of relevant archaeological sites [square symbols] as well as the individual obsidian sources [triangles] and their combined obsidian source areas [color-coded text in italics]. The background map was generated via SimpleDEMViewerAS v2.5.6 based on open-access Shuttle Radar Topography Mission v3.0 (SRTM3) DEM data. (**b**) Photograph of Yeghegis-1 rockshelter taken from the opposite side of the Yeghegis river valley.

To address these questions our team excavated the Chalcolithic rockshelter site of Yeghegis-1 in the Vayots Dzor Province of southern Armenia (39.8646° N, 45.3449° E, 1500 m asl; Fig. 1)^17^. Recent Neolithic research in Armenia has focused on sedentary agricultural settlements, including Aknashen^18^, Aratashen^12^, and Masis Blur^19^. In contrast, Yeghegis-1 is a former cave, and based on our investigation of the faunal assemblage, which is predominately goat and/or sheep^17^, our working hypothesis is that pastoralists wintered herds inside it during the course of seasonal transhumance. Our investigations suggest that it belongs to an emerging class of low-elevation open-air (e.g., Ovçular Tepesi^15,16^) and cave (e.g., Areni-1^3–6^) sites, both near Yeghegis-1 and farther afield (e.g., Getahovit-2 Cave^8–11^), that appear to correspond to winter camps of pastoralists who spent summers at higher elevations with their flocks. These sites, located along rivers, also show evidence of fishing and hunting (e.g., projectile points, wild animal bones), attesting to a diversity and flexibility of subsistence practices that made full use of local resources. This is not to say, though, that these pastoralists had no connection to agricultural settlements. In neighboring regions such as the Anatolian and Zagros highlands, it has been argued that, although mobile, Chalcolithic pastoralists maintained cultural connections to nearby farming villages^20–22^, and this may also have been true, at least in part, in the Southern Caucasus.

The lithic assemblage of Yeghegis-1 is predominantly made of obsidian (92.1%), similar to other Chalcolithic sites within the region (e.g., 92% obsidian at Ovçular Tepesi^16^, 98% at Godedzor^13^), despite being > 60 km on foot (> 40 km linearly) from the nearest geological sources of obsidian. Here we report new radiocarbon dates showing that the site’s occupation spanned about five centuries, from the end of the Middle Chalcolithic to the end of the Late Chalcolithic, which enables a detailed diachronic perspective. We also report our obsidian sourcing results, based on 2141 artifacts from excavated contexts (excluding surface finds, etc.) analyzed using portable X-ray fluorescence (pXRF) in our field laboratory. Our data support two of Sagona’s^1^ proposed trends—occupation of more varied high-altitude environments and more expansive social networks—and also suggest that these were dynamic phenomena, increasing over time. Transhumance likely played a key role in these changes. Use of the nearest pasturelands became more balanced over time—perhaps a strategy of risk management, subsistence flexibility, and/or resource sustainability—and there were more extensive connections with distant communities during the later occupations. These findings lead to a hypothesis for further work: greater social connectivity across the landscape (i.e., more varied land use and more extensive social networks) is the phenomenon that laid the ground for a widespread shared material culture (i.e., the Kura-Araxes phenomenon) across an expansive area, not vice versa.

### Site excavation results

**Figure 2:**
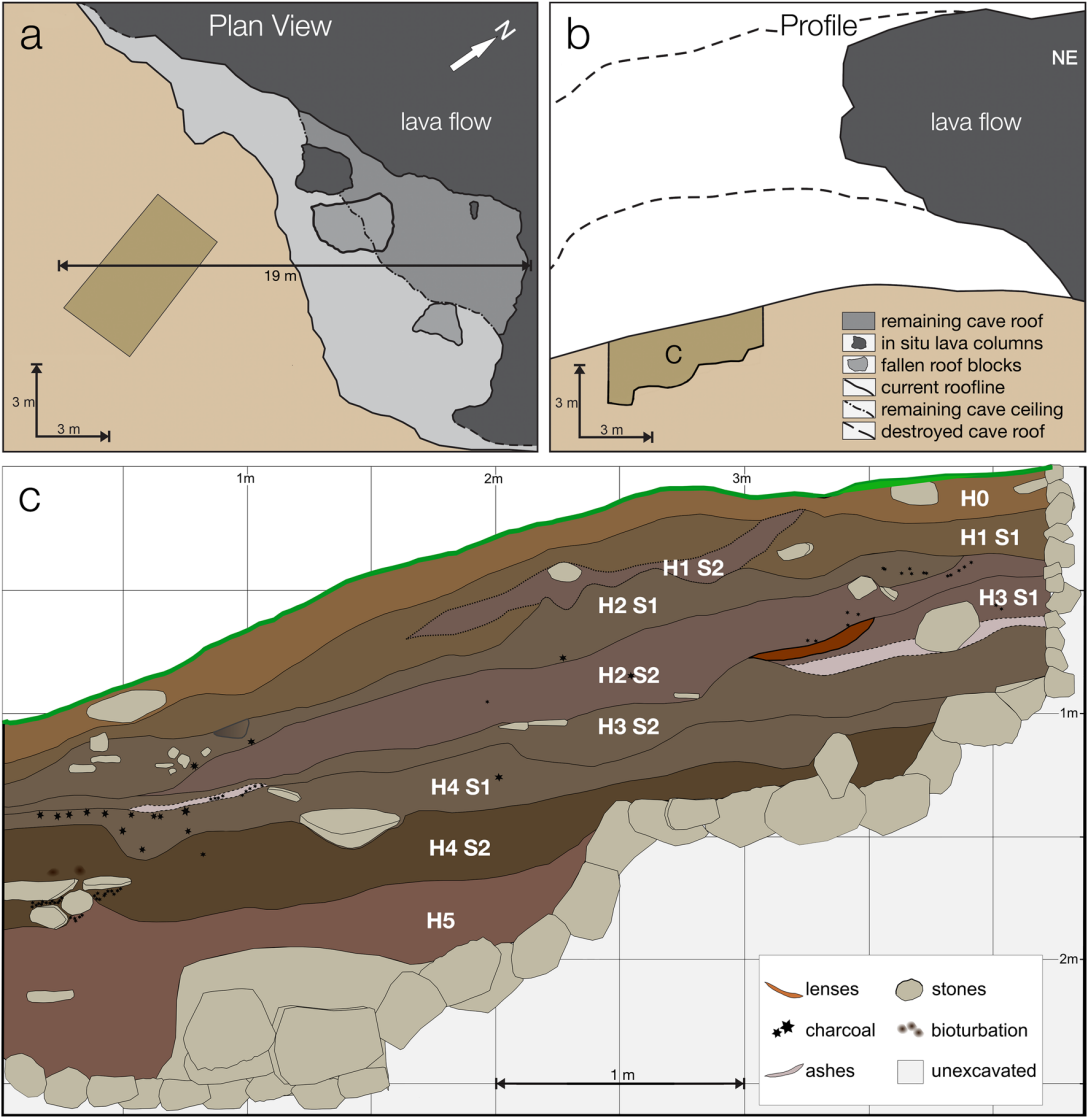
Plan (**a**) and profile (**b**) views of rockshelter and a stratigraphic section (**c**) of the excavations.

Yeghegis-1 lies on the northern (right) bank of the Yeghegis River, a tributary of the Arpa River. It was identified as a surface scatter of lithics, ceramics, and faunal remains during an archaeological survey of the Yeghegis river valley in 2020^17^. Our excavations (Fig. 2) conducted in 2022 and 2023 yielded a stratified sequence with artifact-rich occupation layers (e.g., sherds, lithics, fauna) as well as archaeologically sterile levels. The current rockshelter was initially a larger cave formed by a lava flow from the Smbatassar volcanic vent (~ 12 km north; southern Vardenis mountain range) that covered a Pleistocene river terrace^23^. The lava is a basaltic andesite^24^ and ~ 15 m thick at the rockshelter. The shelter’s entrance has partially collapsed, creating the open area where Trench 2 sits. The collapsed roof spanned an additional ~ 10 m from its current extent and would have covered the location of Trench 2, ~ 19 m from the rear wall of the shelter (Fig. 2). Today the tallest part of the interior reaches ~ 3 m. The archaeological materials lie in colluvial deposits, which cover the clast-rich Pleistocene river terrace that is exposed along the slopes of the river valley.

The site chronology is based on a series of 13 radiocarbon dates (Table 1) performed in the SUERC Radiocarbon Laboratory by accelerator mass spectrometry (AMS)^25^. Measurements were carried out on collagen extracted from medium-sized mammal bones (i.e., one bovid, the other are capra/ovis) recovered from Trench 2. All samples had atomic C/N ratios between 2.9 and 3.6, the preservation range set by DeNiro^26^. Calendar calibration relied on the IntCal20 curve for the northern hemisphere^27^. Bayesian chronological modeling, incorporating an outlier OxCal v4.4 model^28^, was employed to account for any incongruencies in the stratigraphic sequence (see the supplementary materials S1). The resulting dates range from ∼ 4100–4000 cal BCE (Horizon 5, the lowest excavated layer) to ∼ 3600–3500 cal BCE (Horizon 0). Altogether, this sequence of dates indicates an occupation of about five, perhaps six, centuries, from the end of the Middle Chalcolithic (∼ 4100–4000 BCE) through the end of the Late Chalcolithic (∼ 3600–3500 BCE) in this region^1^.

**Table 1:** Radiocarbon dates (all from the 2021–2023 Trench 2 excavations and using collagen extraction).

Layer	Lab #	Reporting #	Species	Bone	d13C	d15N	C/N	Reported age	Calibrated BCE	Prob (%)
H0	GU66344	SUERC-123532	Capra/ovis	Phalanage	− 19.7	2.9	3.4	4813 ± 24 BP	3589–3528	63.3
H1 S1	GU66345	SUERC-123533	Capra/ovis	Radius	− 19.7	3.5	3.2	5229 ± 24 BP	4056–3971	82.9
H1 S2	GU62947	SUERC-108138	Bovidae	Occipital	− 19.5	6.5	3.6	4917 ± 27 BP	3715–3641	82.5
H2 S1	GU66346	SUERC-123534	capra/ovis	Tibia	− 19.5	6.0	3.3	4998 + 24 BP	3806–3704	73.0
H2 S2	GU62948	SUERC-108142	Capra/ovis	Occipital	− 20.8	6.7	3.6	5000 ± 27 BP	3810–3702	67.9
H3 S1	GU62950	SUERC-108144	Capra/ovis	Seasmoid	− 19.3	3.5	3.5	5008 + 27 BP	3814–3704	61.0
H3 S1	GU61230	SUERC-105538	Capra/ovis	Phalanage	− 19.3	3.4	3.3	5008 + 27 BP	3814–3704	61.0
H3 S2	GU62949	SUERC-108143	Capra/ovis	Phalanage	− 20.2	6.0	3.5	5050 + 27 BP	3952–3778	95.4
H4 S1	GU62952	SUERC-108146	Capra/ovis	Occipital	− 19.6	7.9	3.5	5127 + 27 BP	3986–3914	53.6
H4 S2	GU62951	SUERC-108145	Capra/ovis	Occipital	− 20.7	10.9	3.6	5138 + 27 BP	3991–3934	64.0
H5' S1	GU62953	SUERC-108147	Capra/ovis	Occipital	− 19.0	7.6	3.3	5190 + 27 BP	4047–3958	95.4
H5 S1	GU62954	SUERC-108148	Capra/ovis	Occipital	− 19.3	8.7	3.5	5332 + 27 BP	4142–4051	46.5
H5 S1	GU61231	SUERC-105539	Capra/ovis	Phalanage	− 19.3	8.6	3.3	5354 + 27 BP	4136–4054	31.9

Thousands of animal bones (n ≈ 8000 from the 2022 excavations alone) were recovered from the site. About 80% of the faunal assemblage consists of morphologically unidentifiable bone fragments (≤ 7 cm), but the remainder exhibits diagnostic features that permit anatomical and/or taxonomic identifications^17^. The dominance of goat/sheep support use of the cave as a winter camp, but ongoing detailed zooarchaeological (i.e., age of death patterns) and stable isotope analyses will further test this hypothesis. Other ungulates (i.e., cattle, pig/boar, deer) were less abundant. Micromammal and avian bones were numerous, as anticipated in a cave. Carnivores (i.e., bears, wolves) were rare but present. The frequency of burnt/calcined and/or cut-marked bones (> 10%), though, attests that the assemblage is principally anthropogenic in origin, not accumulated by carnivores. Additional zooarchaeological studies, including (1) taxonomic identification via morphology and mass spectrometry (ZooMS) and (2) stable isotopic analyses on dental enamel, are underway and will yield further insights into herd management strategies and seasonal movements.

**Figure 3:**
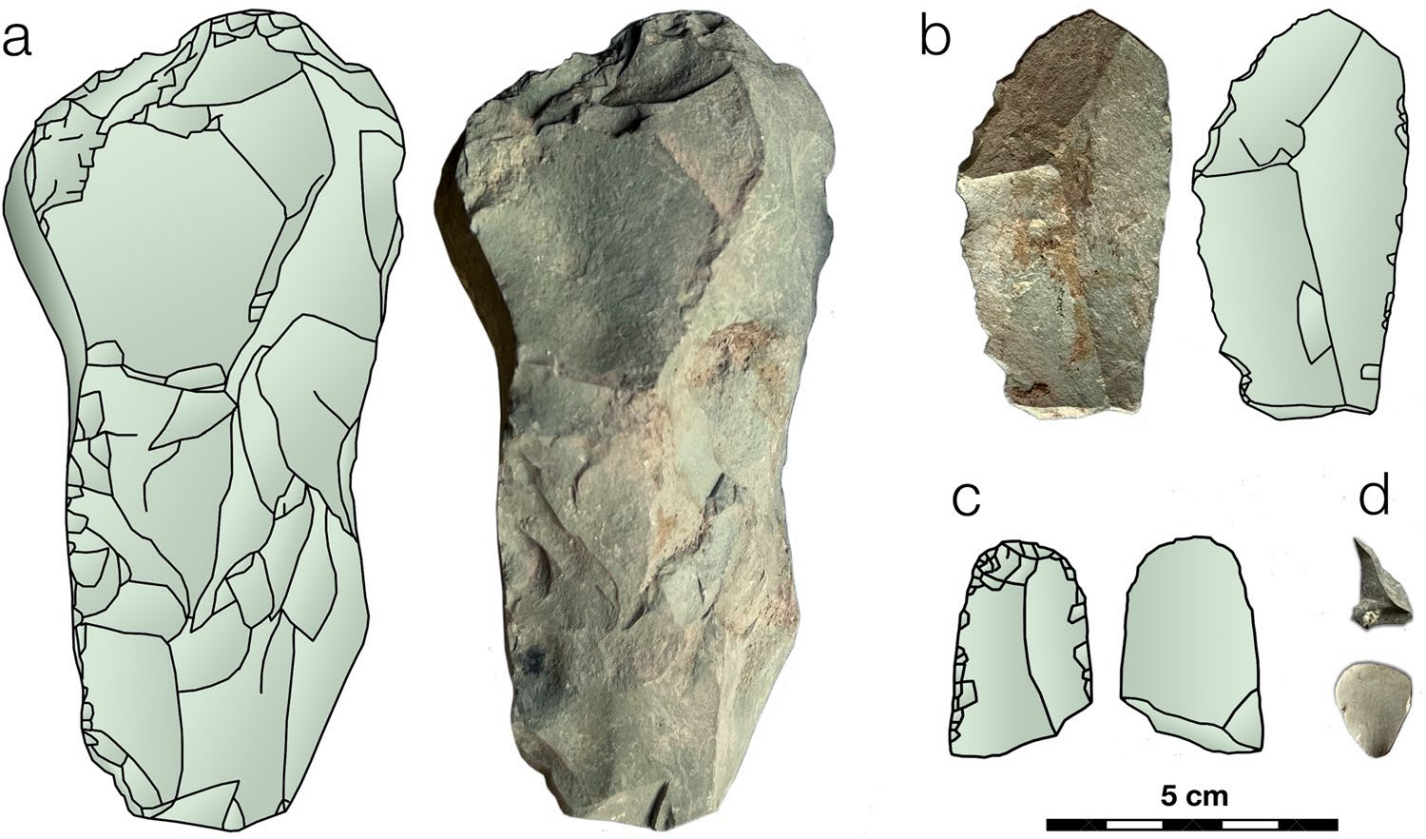
Examples of the green-hued argillites, available in the nearby river terrace, used by the Yeghegis-1 occupants to replenish their lithic stock: (**a**) a large flake core (surface find) and (**b**) a blade or elongated flake, (**c**) a blade segment, and (**d**) small flakes/debitage (all from Trench 2, Horizon 3, Subhorizon 1).

The Yeghegis-1 lithic assemblage (n ≈ 3000 artifacts from the 2022 and 2023 excavations) is, as noted in the Introduction, predominantly obsidian: 92.1% by count (and ∼ 50% by mass due to several large non-obsidian cores). Obsidian is not available in the river valley. Indeed, the site (1500 m asl) is > 60 km on foot (> 40 km linearly) from the nearest obsidian sources, which lie at high elevations (∼ 2900–3300 m asl) in potential summer pastures and are covered by more than a meter of snow during the cold season. Hence, the obsidian supply at Yeghegis-1 could not have been replenished during the winter, and the highly reduced obsidian artifacts in certain periods reflect this. The cave’s occupants appear to have restocked their lithic material supplies principally using green-hued argillites (Fig. 3) collected from the nearby river terrace. The lithic assemblage also includes cherts, which are reportedly available locally from high-quality deposits in the Arpa River basin^3^. Overall, the proportion of obsidian (by count, which minimizes the effect of large cores) remains largely constant through time, varying only between ∼ 85% (Horizon 5) and ∼ 93–95% (multiple layers).

**Figure 4:**
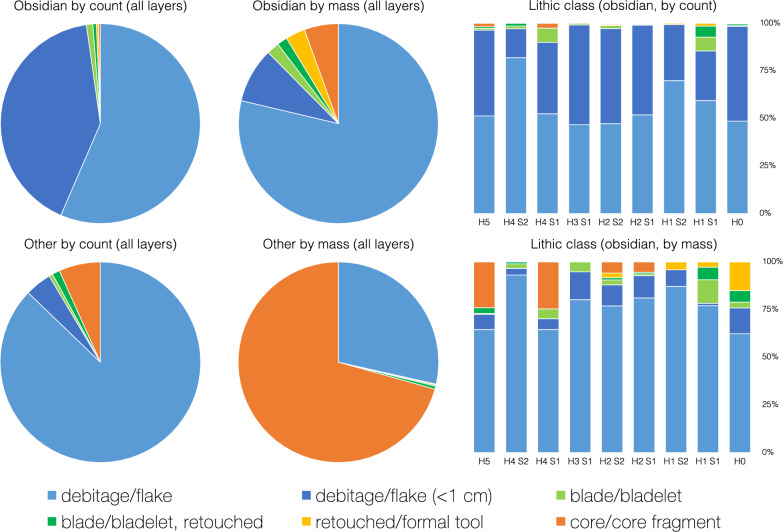
Lithic class for obsidian and other raw materials by count and by mass using our 2022 excavation data. Artifacts made of other raw materials, principally local argillites, are generally larger than those of obsidian (e.g., the obsidian flakes/debitage are much more likely to be smaller than 1 cm). The data for these graphs are included in the supplementary materials S1.

**Figure 5:**
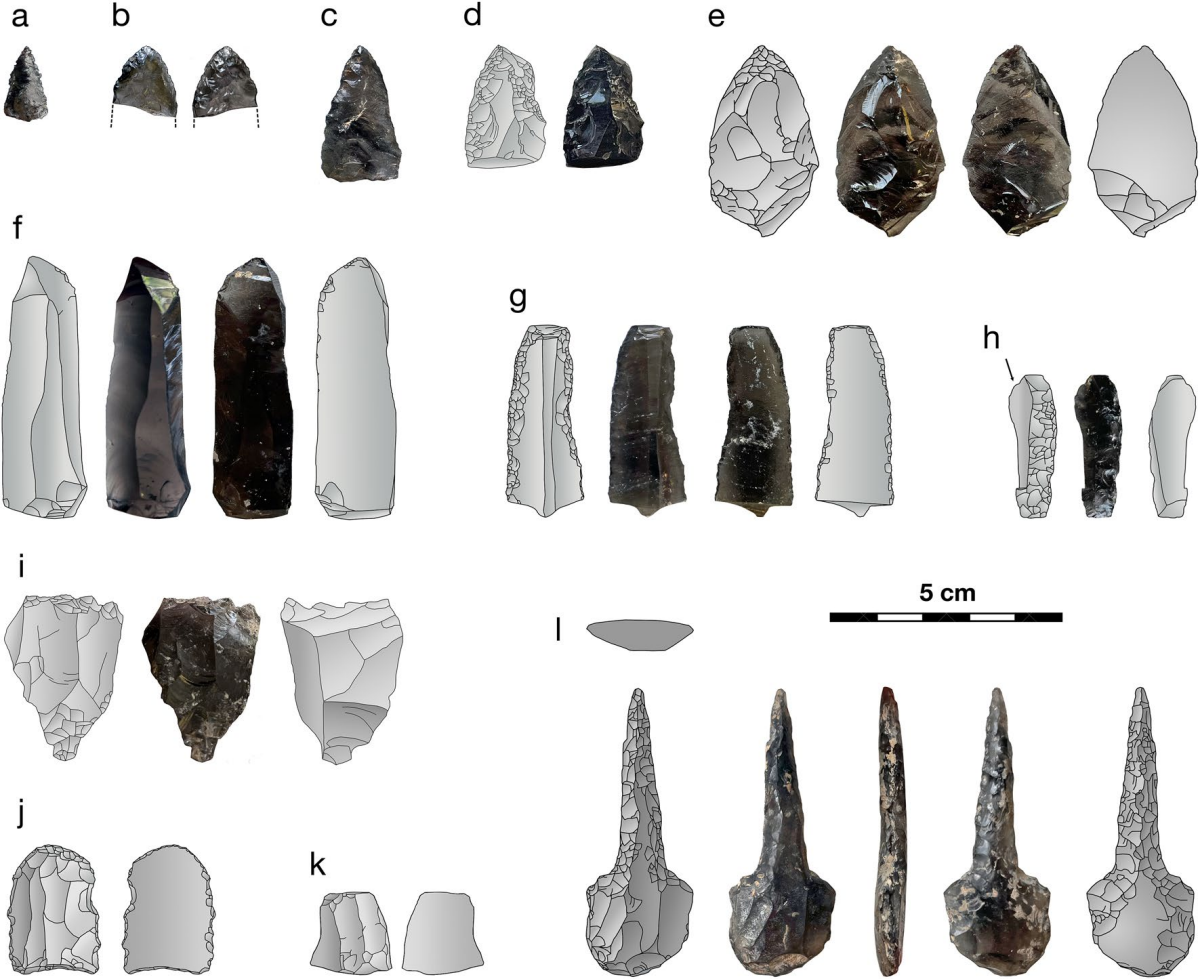
Select obsidian artifacts in the Yeghegis-1 assemblage: (**a**) tiny projectile point (Trench 2, Horizon 2, Subhorizon 1), (**b**) broken projectile point (T2 H5 S1), (**c**) projectile point (T2 H4 S2, ash layer), (**d**) possible point with notch retouch (T2 H1 S1), (**e**) flake retouched to a point (T2 H1 S1), (**f**) unretouched blade (T2 H1 S1), (**g**) retouched blade (T2 H1 S1), (**h**) burin (T2 H2 S2), (**i**) core fragment (T2, collapse), (**j**) blade segment (T2 H2 S1), (**k**) blade segment (T2 H1 S1), and (**l**) blade reshaped into an awl (T2 H2 S2). Note that, as shown in Fig. 4, all of these lithic classes are rarities in the Yeghegis-1 assemblage.

As shown in Fig. 4, based on the 2022 excavation data, the majority of obsidian artifacts (97.8% by count) are unretouched flakes and other debitage, and of those, more (58% by count) are > 1 cm in maximum dimension. Examples of the other artifact types appear in Fig. 5. Blades and bladelets, about one third of which exhibit apparent retouch, constitute 1.6% of the obsidian artifacts. There is no clear impression of highly regularized blade or bladelet production on-site (nor are there enough artifacts to measure reliably and test such a hypothesis). Formal tools are rare: there is, as shown in Fig. 5l, a single obsidian awl, one burin, and three small projectile points (plus two larger flakes shaped into points). Projectile points have frequently been interpreted as hunting tools, awls as piercing tools, and burins as incising tools^29^. Given the presence of projectile points, small flakes might also have been used as hafted microliths for such a purpose. Cores and core fragments reflect 0.3% of the obsidian assemblage. These cores were reduced almost entirely. Most of their remaining fragments are attempts to rejuvenate a core with a new striking platform, sometimes to correct for a step or hinge termination error. Ultimately, one is left with the impression that the cave occupants routinely used their obsidian supplies to the point of near-exhaustion. The cave was clearly not the location of a lithic workshop specialized in, for example, producing standardized blades or their cores for trade purposes. Such a specialized workshop has, though, been reported at Mentesh Tepe^1^, where almost half of the obsidian artifacts relate to blades (e.g., tools, blanks, cores). Instead, the obsidian assemblage at Yeghegis-1 is more similar to that at Ovçular Tepesi^16^, which has been interpreted as a reflection of pastoralists’ seasonal knapping activities. Specifically, the assemblage at Ovçular Tepesi was heterogeneous, lacked standardized blade cores and yet had a few sizable blades, and largely consisted of debitage indicative of ad hoc production^16^. Hence, as shown in Figs. 4 and 5, the Yeghegis-1 assemblage is more consistent with Ovçular Tepesi than Mentesh Tepe.

As the cave’s occupants exhausted their obsidian supply collected from summer pastures, they restocked with other, locally available raw materials. As shown in Fig. 3, the other lithic materials have larger flakes and debitage (< 5% are < 1 cm in maximum dimension, compared to 42% for the obsidian). In addition, there are several large cores among the other materials, one of which was half a kilogram. Together, the cores and larger flakes/debitage (> 1 cm) account for 99.2% of the other lithic material mass.

Figure 4 illustrates, based on the 2022 data, the relative steadiness in obsidian artifacts’ typological classes (by count and by mass) over time. That is, the predominance of unretouched flakes/debitage (of any size) changes little. Other artifact classes (e.g., blades/bladelets, cores) are scattered throughout the layers and exhibit no clear trends. In contrast, Sagona^1^ reports that the obsidian assemblage (∼ 94% of all lithic artifacts) from the archaeological site of Sioni (Georgia) included “some retouched blades” in the earlier phases but was “overwhelmingly flakes” by the end (but he also notes that a quantitative analysis of the assemblage had yet to be carried out). Such an unambiguous trend among the obsidian artifacts is absent at Yeghegis-1—and, reportedly, at other Chalcolithic sites (e.g., Delisi^1^, Ovçular Tepesi^16^). Consequently, as obsidian sourcing data are plotted across time, we have confidence that there are no simple typological trends (e.g., there is no replacement of retouched blades by unretouched flakes—or vice versa—in later phases) that would confound either our results or their interpretations.

### Obsidian sourcing results

Yeghegis-1 is fortuitously located for obsidian artifact sourcing in that the site is surrounded by four highland obsidian source areas, some used as summer pastures today, within ∼ 40–55 km (linearly) in varied directions: (1) the Gegham sources (Geghasar and Spitakasar) to the northwest at ∼ 3000–3300 m asl, (2) Khorapor to the northeast at ∼ 2900 m asl, (3) Kelbadjar to the east at ∼ 3000–3200 m asl, and (4) the Syunik sources (Sevkar and Satanakar) to the southeast at ∼ 2700–3000 m asl (Fig. 1). Each of these four areas corresponds to a potential summer pasture, whereas artifacts from more distant sources are more likely to reflect social contacts (e.g., exchange or, more generally, connectivity).

Our results are conceptualized here in terms of pasturing areas, not specific volcanic outcrops of obsidian. Consequently, we chose to combine the obsidian sources in particular source areas. That is, for example, the Sevkar and Satanakar 1–3 obsidian sources have been merged here into the Syunik source area because these sources are magmatically related, spatially close, and chemically similar. Outcrops of Sevkar and Satanakar 1–3 obsidian occur < 4 km apart, the Satanakar 1–3 obsidian outcrops all lie within ∼ 2 km, and there are small streams and gullies where the obsidian has mixed^30^. Chemically these sources can be reliably differentiated via pXRF of flat surfaces on geological specimens; however, such fine distinctions become less reliable with small, irregular, hydrated artifacts. Similarly, Geghasar and Spitakasar have been combined here into the Gegham source area, and so on (Fig. 1).

**Figure 6:**
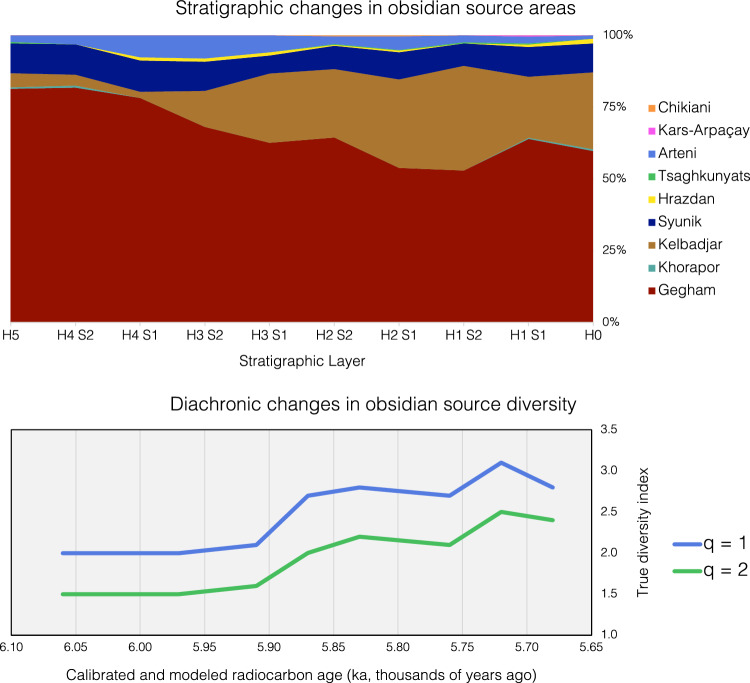
Two methods of plotting diachronic changes in obsidian source areas reflected in the Yeghegis-1 assemblage: a stacked area chart showing the proportions of the different obsidian source areas found in each stratigraphic layer (above) and a line graph of true diversity metrics changing over time (below). With q = 1, each obsidian source (including the rare ones) is equally weighted by its abundance, whereas when q = 2, the most abundant obsidian sources are given additional emphasis.

As shown in Table 2 and Fig. 6, three out of the four nearest obsidian source areas—the Gegham sources (overall: 64.2%), Kelbadjar volcano (21.9%), and the Syunik sources (9.1%)—are predominant. Of these three source areas, the Gegham and Syunik sources are known to have been exploited for their obsidian^30^, but artifacts of Kelbadjar obsidian have rarely been identified, even provisionally^31^. Note that, at the contemporaneous site of Areni-1 Cave, ∼ 20 km to the southwest along the Arpa River, Areshian et al.^3^ proposed that its obsidian artifacts originated from the Gegham and Syunik (Vorotan) sources based on only visual attributes, not chemical analyses. At present, it is unknown if Kelbadjar obsidian artifacts are also present at Areni-1 in a similarly high proportion and have simply not been recognized visually. The other near obsidian source—Khorapor to the northeast—is represented in the Yeghegis-1 assemblage by just four artifacts. It should be noted that Khorapor obsidian is low in both abundance and quality and that no obsidian quarries or workspaces have been reported on its slopes^31^. In fact, to the best of our knowledge^30^, these four are the first knapped obsidian artifacts known to have originated from Khorapor.

**Table 2:** Summarized obsidian source area identifications (with ratios of sourced obsidian artifacts to ceramic sherd total mass) by stratigraphic layer.

Stratigraphy		Nearest source areas				Farther source areas					Totals	Ceramic data	
Horizon	Subhorizon	Gegham	Khorapor	Kelbadjar	Syunik	Hrazdan	Tsaghkunyats	Arteni	Kars-Arpaçay	Chikiani	All sources	Mass (kg)	Ceramics/obs
0	–	107	1	48	18	3		2			*179*	11.9	0.07
1	1	160	1	53	26	2	1	6	1		*250*	5.5	0.02
1	2	135		93	20		1	6			*255*	7.5	0.03
2	1	194		111	34	2	1	17		1	*360*	7.6	0.02
2	2	198		73	25	1	1	8		1	*307*	7.8	0.03
3	1	161		62	16	3		15			*257*	9.7	0.04
3	2	60		11	9	1		7			*88*	3.8	0.04
4	1	72		2	10	1		7			*92*	3.3	0.04
4	2	108	1	5	14			4			*132*	3.6	0.03
5	–	180	1	11	23		1	5			*221*	4.5	0.02
*Totals*		*1375*	*4*	*469*	*195*	*13*	*5*	*77*	*1*	*2*	*2141*	65.0	0.03

Summed values are in italics.

The Arteni source area lies ∼ 145 km linearly (> 200 km on foot), yet it reflects 3.6% of the entire obsidian assemblage. Indeed, it is represented by artifacts in each horizon and subhorizon, varying from 1.1% (Horizon 0) to 8.0% (Horizon 3 Subhorizon 2). Importantly, the Arteni obsidian sources do not lie at high elevations like the Gegham (∼ 3000–3300 m asl) or Syunik (∼ 2700–3000 m asl) sources. Instead, outcrops of Arteni obsidian occur at elevations of ∼ 1400 m asl, lower than that of Yeghegis-1 (1500 m asl). Today, the Arteni sources are surrounded by arid semi-desert steppe and experience hot summers, making the area an unlikely summer pastureland. Less than 20 km to the east, though, the slopes of Mount Aragats (an immense stratovolcano) begin to rise, offering grassland steppe and alpine meadows. Hence, one would anticipate winter encampments in the lowlands near the Arteni sources as well as summer pastures in the nearby highlands of Mount Aragats.

The two Hrazdan obsidian sources—Gutansar and Hatis volcanoes—are ∼ 75 km linearly (> 160 km on foot) from Yeghegis-1. Despite being closer to the site, the Hrazdan sources are represented by 13 artifacts (0.6% overall; six from Gutansar, seven from Hatis) in seven of the ten layers (Table 2). That is, despite sitting somewhat between the Gegham and Arteni sources, the Hrazdan sources are reflected in the assemblage in lower numbers and with less consistency. Notably, like the Arteni sources, the Hrazdan sources occur at relatively low elevations: the lowest Hatis obsidian outcrops sit at ∼ 1600 m asl and the lowest Gutansar ones at ∼ 1400 m asl. It is also notable that all the Hrazdan artifacts are small flakes/debitage ≲1 cm in maximum dimension, rather than curated tools.

Finally, three distant obsidian source areas are represented by eight artifacts. There are five artifacts—all flakes/debitage ≲1–2 cm in diameter—from the Tsaghkunyats sources, which lie ∼ 120 km to the northwest linearly and > 150 km on foot. There is one small (< 1 cm) flake from the Kars-Arpaçay sources (Turkey), which lie ∼ 170 km to the northwest linearly and > 240 km on foot. Two small flakes—one ∼ 2 cm in diameter and the other ≲ 1 cm—derived from Chikiani (Georgia), which lies ∼ 220 km to the northwest linearly and > 340 km on foot.

Given that one of our aims was to consider Sagona’s^1^ idea that Chalcolithic peoples occupied more varied highland environments, which, in turn, opened more pastureland to them, Fig. 6 graphs the diversity of obsidian represented at Yeghegis-1 through time. The results show that there is a 40% (q = 1) to 50% (q = 2) increase from the lowest stratigraphic layer to the highest. Thus, there was an increase in the source diversity of obsidian artifacts through time. This diversity increase is largely due to a drop in the abundance of Gegham obsidian and a corresponding rise in Kelbadjar obsidian. Most of the increase (a 29% rise when q = 1) occurs between Horizon 4 Subhorizon 1 (∼ 5.91 ka) and Horizon 3 Subhorizon 2 (∼ 5.87 ka), and a second increase in the obsidian diversity (a 15% rise when q = 1) is observed between Horizon 2 Subhorizon 2 (∼ 5.76 ka) and Horizon 2 Subhorizon 1 (∼ 5.72 ka). Another trend found in Table 2 is that, with the exception of Horizon 0 (likely due to surface deflation), the number of sourced obsidian artifacts relative to the recovered ceramic sherds (by mass) remains fairly constant, suggesting that our sample adequately reflects the intensity of the site’s occupation over time.

## Discussion

First and most simply, our results highlight the value of considering Chalcolithic phenomena with a greater temporal resolution than Middle versus Late Chalcolithic. Our data derive from ten stratigraphic layers that correspond to about five centuries of occupation, and it is evident that behavioral changes occurred throughout that timeframe. One could suggest that the step in obsidian diversity circa 3900 cal BCE corresponds to the Middle to Late Chalcolithic transition, which Sagona^1^ placed around 4000 BCE in this region. However, it must be stressed that the Early, Middle, and Late Chalcolithic are largely arbitrary 500-year increments (5000–4500, 4500–4000, 4000–3500 BCE) used to subdivide a period with, until recently, few precise radiocarbon dates. Nevertheless, he did propose that the Chalcolithic might be better divided into a later phase and an earlier one, with a boundary between them at ∼ 4000 BCE. This might be no more than coincidence, and ideally data from a longer span of the Chalcolithic Period could be brought to this question. Crucially, our data highlight potential mechanisms for the observed differences in earlier and later phases of the Chalcolithic: shifts in land use and, in turn, social connections across the wider region.

Sagona’s^1^ observation of more diverse subsistence strategies, settlement practices, and social networks during the Chalcolithic, relative to the Late Neolithic, could be seen as flexibility potentially tied to risk mitigation. If, for example, pastoralists’ migration route is impacted by severe weather one year, it would be beneficial to be able to draw upon a social network that extends into an unaffected area. As shown in our data, the obsidian contribution from the Syunik sources (to the southeast) remains fairly constant at ∼ 8–11% (with the exception of 6.2% in H3 S1). Therefore, this can be interpreted as a fairly consistent summer use of pastures near the obsidian sources in the Syunik highlands. The largest trend in our data is the shift from a predominance of Gegham obsidian (∼ 78–82% in the lowest three layers) to more of a balance between Gegham and Kelbadjar obsidian (52.9% and 36.5%, respectively, in H1 S2). We suggest that this shift in obsidian source areas corresponds to a shift in pasture use. That is, our data indicate that the Gegham source area (to the northwest) was a predominant summer pastureland during the site’s earliest occupations; however, there was a later shift toward a greater balance between Gegham and Kelbadjar (to the east) and their corresponding pastures. It remains uncertain, though, if these pastoralists (1) more frequently rotated their herds amongst the different pastures year-to-year or (2) more equally divided their herds into the different pastures each year. Either scenario could be viewed as flexibility to avoid overgrazing in any particular pasture, whereas the second scenario could also be interpreted as risk mitigation—that is, a herd may be impacted by a disaster in one pasture, but others remain safe in pastures that lie in different directions. The aforementioned stable isotope studies of dental enamel should allow further insights into these processes.

Far-traveled obsidian artifacts from the Tsaghkunyats, Kars-Arpaçay, and Chikiani source areas are more common later in the Yeghegis-1 sequence. To be specific, one artifact (from Tsaghkunyats, the closest of these three source areas) is found in Horizon 5, none are found in Horizons 4 and 3, and seven artifacts (from all three) are found in Horizons 2 and 1. Consequently, we would argue that these results indicate more frequent long-distance connections during the cave’s later occupations. We do not suggest that “trade” or “importation” occurred between these regions. Instead, it seems likely that, as proposed by Crawford^32^, the seasonal movements of different pastoral communities created a crisscrossing social network, which could move items long distances. The eight far-traveled artifacts are all small and exhausted in their utility to yield cutting edges, and there is no evidence to suggest that the obsidian from, for example, Chikiani was treated differently than that from closer sources. This stands in stark contrast to the conclusion of Chataigner et al.^11^ at Getahovit-2, where those authors identified two far-traveled obsidian artifacts among the 38 Late Chalcolithic artifacts that they tested in France. Those two artifacts—one from Chikiani and one from the Kars region (possibly even the Kars-Arpaçay sources)—were pressure-flaked blades, and consequently, Chataigner et al.^11^ proposed that such obsidian blades had been “imported” from those distant regions, not made locally.

The somewhat surprising abundance of Arteni obsidian (3.6% overall), despite its distance from Yeghegis-1, has two potential interpretations, given that its obsidian decreases as Gegham obsidian does within the assemblage. First, given that the Arteni and Gegham source areas both lie to the northwest of Yeghegis-1, it might be that, as pastures near the Gegham obsidian sources were used less often and/or less intensively, fewer interactions—direct or indirect—occurred between the pastoralists who wintered at Yeghegis-1 and those who wintered in the lowlands near the Arteni sources. Second, it might be that a given pastoral group did not exclusively winter at Yeghegis-1. Instead, pastoralists who used the summer pastures near the Gegham sources might sometimes have opted to winter near the Arteni sources some years, and consequently, fewer wintered near the Arteni sources when more spent the summer near the Gegham sources. This latter interpretation is more at odds with recent views that Chalcolithic pastoralists were less mobile than widely thought and more “anchored” to nearby farming settlements^20–22^. Consequently, the former explanation—the Arteni obsidian reflects persistent but changing social contacts over a distance—is the most parsimonious one in light of our results and other recent studies involving this topic (and, perhaps, our forthcoming stable isotope research). Under this scenario, the mechanism of the changing social network is changing land use tied to transhumance.

Ultimately our results not only support two of the Chalcolithic trends noted by Sagona^1^—(1) occupation of more varied environments, particularly at higher altitudes, and (2) more expansive social networks across the landscape—but also suggest that these were dynamic phenomena which increased over time. Our work highlights the roles that transhumance likely played in the changes in land use and social networks. A greater balance in the use of the nearest pasturelands (and obsidian source areas) over time can be seen as a strategy related to flexible subsistence, risk management, and/or resource sustainability (i.e., preventing overgrazing), and during the site’s later occupations, there are more artifacts that come from more distant obsidian source areas, revealing more extensive connections.

We suggest, at least initially, that an increase in social connectivity played a key role in the shifts in societal complexity that gave rise to the Kura-Araxes cultural complex, which stretched throughout the Armenian Highlands and into surrounding regions around the start of the Early Bronze Age. Thus, we propose a hypothesis that necessitates additional study: greater connectivity across the landscape (i.e., more varied land use and more extensive networks) is a key phenomenon that laid the ground for a widespread material culture (i.e., Kura-Araxes ceramic types) over an expansive region, not the reverse. In turn, expanded networks would not be a product of a shared (material) culture that enabled greater connectivity, rather shared material culture would be a consequence of greater connectivity through expanded social networks. Such a model, whereby greater social connectivity is a mechanism for, rather than a result of, cultural and/or technological innovations, also holds potential for its application to and testing in a wide variety of contexts around the world, spanning from the Palaeolithic to the modern day.

## Methods

### Excavation methods and stratigraphy

Excavations of Yeghegis-1 started in 2021 and continued in 2022 and 2023. In 2021, two areas of the site were tested: the interior of the rockshelter and a relatively flat area on the slope in front of the current entrance. The interior, designated as Trench 1, was covered by a thick layer of dung (~ 2 m) that was cleared. The dung is evidence that the area has been used to shelter herds until recently. After clearing, the excavation started but was soon terminated due to encountering large (~ 1–3 m) basalt blocks that collapsed from the cave roof. At the same time, another trench (Trench 2, 1.5 × 1.5 m, north–south orientation) was opened to a depth of 1 m outside the remaining rockshelter. In 2022, our excavations continued, expanding Trench 2 by 1 m to the northwest (toward the shelter entrance) to a depth of 1.7 m. In 2023, the excavations in Trench 2 were conducted in six 1 m^2^ units (A0, B0, C0, A1, B1, C1) further to the northwest. A low, stone structure constructed from various rock types (e.g., gabbro, basalt) was unearthed along the length of the excavation area. It consists of three rows of middle-sized stones, approximately 1 m in height. Archaeological finds were discovered below the structure, suggesting the existence of multiple occupations at the site.

Artifacts, faunal, and botanical remains were recovered during the Trench 2 excavations according to stratigraphic divisions by horizon and subhorizon. The stratigraphic sequence of Trench 2 was subdivided into horizons and subhorizons on the basis of visible differences in the sediment (e.g., color, texture, and presence of rocks) and in the abundance of cultural material (e.g., ceramic, lithics, ores, charcoal, and animal bones). Hearths with charcoal and ash were observed in several layers, as shown in Fig. 2c. Five horizons and eight subhorizons were excavated. Horizon 0 represents the topsoil, while Horizon 5 is the last layer excavated, characterized by a low number of artifacts. The intervening horizons (1–4) consist of ashy-clay soil with the presence of charcoals, obsidian, bones, and ceramics, which are evidence of the cultural layers in the site’s stratigraphic sequence.

### Obsidian artifact sourcing

Obsidian artifacts were analyzed by pXRF in our field lab in the Yeghegis valley, permitting us to measure them without damage or exporting them to a distant facility. All artifacts were analyzed with an Evident/Olympus Vanta M-series pXRF instrument with a Rh anode and 4-W X-ray tube. In its two-beam GeoChem measurement mode, the instrument’s settings change to excite heavier and lighter portions of the periodic table: (1) 40 kV tube voltage, ~ 65 µA tube current, 2000-µm-thick Al beam filter, ~ 70,000 counts per second (cps) and (2) 10 kV voltage, ~ 76 µA current, no filter, ~ 70,000 cps. The characteristic X-rays are measured by a large-area Si drift detector (SDD) with high resolution (≲ 140 eV). Instead of employing older empirical methods, the instrument uses the fundamental parameters (FP) approach to data correction, which describes mathematical relationships between the measured X-ray intensities and element concentrations. That is, FP accounts for physical interactions in matter (e.g., X-ray attenuation, fluorescence) and other behavior (e.g., absorption and fluorescent edges, escape peaks). Measurements took 20 s (15 s for heavier elements, immediately followed by 5 s for lighter ones).

Elemental data for obsidian sources of the Southern Caucasus were not merely drawn from the literature, which contains measurements with mixed accuracy. Instead, a geological reference collection, which consists of > 320 mounted obsidian specimens from the Southern Caucasus and surrounding areas, were analyzed with the same instrument. Using the same instrument (i.e., same hardware configuration, same software algorithms) ensures maximum compatibility between the measurements of artifacts and geological specimens. The geological specimens were cut to a target size (~ 10–20 mm diameter, ~ 5–20 mm thick) using a diamond-embedded blade on a rock slab saw and were encapsulated in epoxy resin (EpoxySet, Allied High Tech Products) with two-part mounting cups (25 mm diameter). Hardened epoxy was tested by pXRF, and as expected, the organic compounds contain no elements that would interfere with measurements for obsidian sourcing. The specimens were cut with a diamond-embedded wafering blade on a low-speed precision saw to create puck-shaped mounts with flat, smooth, and fresh surfaces for analysis. That is, the specimens have ideal surfaces for XRF measurements^33–36^, allowing fine elemental distinctions [see 30] among sources that are not always reliable for small and/or irregularly shaped artifacts.

The instrument’s factory-set calibration, largely geared toward applications in economic geology and ore exploration, is based on a set of standard reference materials (SRMs), primarily from the United States’ National Institute of Standards and Technology, the United States Geological Survey (USGS), and similar organizations. This initial GeoChem calibration was fine-tuned for obsidian via linear regression analysis based on a PYRO (Peabody-Yale Reference Obsidians) set, which has 35 obsidian specimens that have been analyzed in a series of different laboratories^37^ and also includes one of the most common SRMs for data assessment: USGS RGM-1 (Glass Mountain, California, US).

Analyzing small and/or irregular artifacts can lead to over- or under-estimates of specimen mass (depending on the correction method used) and, in turn, elemental measurements that are too low or too high, respectively^33,35,36^. Various protocols have been proposed to “correct” skewed measurements from small and/or irregular obsidian specimens^35,38^, but most of these suggestions are impractical for analyzing even small numbers of artifacts. In contrast, Frahm^36^ focused on mathematical methods that could be readily applied to large artifact assemblages, recommending the use of element ratios and linear discriminant analysis (LDA) as a means to minimize the effects of systematic error on the resulting measurements. For our study, we applied both of these mitigations simultaneously. Specifically, as detailed in the supplementary materials S1, we applied LDA to (1) the eight PYRO-calibrated elements (Mn, Fe, Zn, Rb, Sr, Y, Zr, and Nb) and (2) eight mid-Z trace-element ratios (Rb/Zr, Sr/Zr, Y/Zr, Nb/Zr, Sr/Rb, Y/Rb, Zr/Rb, and Nb/Rb). Calibrated analyses of 260 geological reference specimens were used as the training set on which the LDA functions were based. Prior and posterior classification and cross-validation tests revealed no misclassifications of these reference specimens to their known sources. All of our data and statistical outputs—elemental measurements, statistical tests (e.g., correlation and covariance matrices), classification functions, results (e.g., membership probabilities and squared distances), and validations (e.g., confusion matrices for the training and the validation samples)—are included in the supplementary materials S1 so that anyone is able to interrogate and reproduce our results.

### Obsidian sourcing methods in prior studies

Several earlier studies have conducted obsidian artifact sourcing at Chalcolithic sites within the Southern Caucasus, but none of them used pXRF as a means to nondestructively identify obsidian sources layer-by-layer using a large number of artifacts. Almost all of the 20th-century results are unpublished^31^, more than three-quarters of which were tested with neutron activation analysis (NAA)^39^. Other 20th-century researchers instead used wavelength-dispersive XRF (WDXRF), which involved turning artifacts into a fine powder for analysis, or fission-track dating, which involves slicing, grinding, polishing, and chemically etching artifacts for examination using a visible-light microscope. In contrast, 21st-century researchers have, to the best of our knowledge, hitherto only sent artifacts to an analytical lab at the University of Orléans (France) for laser-ablation inductively coupled plasma mass spectrometry (LA-ICP-MS). Chataigner and Gratuze^40^ reported that 21 artifacts from the Late Chalcolithic settlement of Godedzor (southern Armenia) chemically match two Syunik obsidian sources (Sevkar and Satanakar) ~ 10–15 km to the north. Similarly, Chataigner et al.^11^ sourced 61 artifacts from Getahovit-2 Cave (northern Armenia) and separated their results into two phases: Middle Chalcolithic (n = 23) and Late Chalcolithic (n = 38). Gratuze and Rova^41^ tested 50 artifacts from the open-air site of Tsiteli Gorebi 5 (eastern Georgia), which, due to agricultural activities, has almost no in situ archaeological material. Indeed, the authors even report that only one artifact came from a closed Chalcolithic context. Finally, in Orléans, Astruc et al.^42^ used LA-ICP-MS—together with microXRF—to analyze 269 Chalcolithic artifacts from Mentesh Tepe (Azerbaijan).

### Calculating source diversity

Here we use diversity indices that have been primarily developed for ecological research (e.g., a territory in which only two species prevail is less diverse than one in which multiple species have equal abundances) but have also been applied in other fields. The indices can account for both the number of types present in a set (which is known as richness) and their relative abundances (known as evenness). In this scenario, a diverse obsidian assemblage is one in which artifacts come from various sources, each of which is well represented, while an assemblage in which artifacts principally derive from one source would be less diverse. We use two orders of Hill numbers (^q^D) to calculate diversity metrics: one based on the weighted geometric mean (q = 1; each type is equally weighted by abundance) and the other on the weighted arithmetic mean (q = 2; abundant types are given added emphasis)^43^. When q = 1, the resulting index is mathematically related to the Shannon–Wiener and the Simpson diversity indices, which previously have been applied in similar contexts^44,45^. With q = 2, the results emphasize the frequently used sources (which, we propose, mainly reflect summer pastures) over rarer ones (which, we propose, reflect social connections).

### Supplementary Information

Supplementary Information.

## Data Availability

All data are available in tables in either the main text and the supplementary materials S1. OxCal code and statistical outputs are also available in the supplementary materials S1.
