# Author Correction: Increasing obsidian diversity during the Chalcolithic Period at Yeghegis-1 Rockshelter (Armenia) reveals shifts in land use and social networks

**DOI:** 10.1038/s41598-024-63715-3

**Published:** 2024-06-04

**Authors:** Ellery Frahm, Mariam Saribekyan, Satenik Mkrtchyan, Laura Furquim, Ara Avagyan, Lilit Sahakyan, Karen Azatyan, Patrick Roberts, Ricardo Fernandes, Levon Yepiskoposyan, Noel Amano, Mariya Antonosyan

**Affiliations:** 1 Council on Archaeological Studies, Department of Anthropology, Yale University, New Haven, USA; 2 Anthropology Division, Peabody Museum of Natural History, Yale University, New Haven, USA; 3 Institute of Archaeology and Ethnography, National Academy of Sciences, Yerevan, Republic of Armenia; 4 Institute of Molecular Biology, National Academy of Sciences, Yerevan, Republic of Armenia; 5 Department of Archaeology, Max Planck Institute of Geoanthropology, Jena, Germany; 6 Institute of Geological Sciences, National Academy of Sciences, Yerevan, Republic of Armenia; 7 Yeghegnadzor Regional Museum, Yeghegnadzor, Republic of Armenia; 8 Arne Faculty of Arts, Masaryk University, Brno, Czech Republic; 9 Department of Bioarchaeology, Faculty of Archaeology, University of Warsaw, Warsaw, Poland

Correction to: *Scientific Reports* 10.1038/s41598-024-59661-9, published online 25 April 2024

In the original version of this Article, Noel Amano was omitted as a corresponding author. Correspondence and requests for materials should also be addressed to amano@shh.mpg.de.

Furthermore, an affiliation was omitted for Ricardo Fernandes. Their correct affiliations are listed below.

Department of Archaeology, Max Planck Institute of Geoanthropology, Jena, Germany.

Arne Faculty of Arts, Masaryk University, Brno, Czech Republic.

Department of Bioarchaeology, Faculty of Archaeology, University of Warsaw, Warsaw, Poland.

The original Article and accompanying Supplementary Information file have been corrected.

