# Supplementary material for: Increasing obsidian diversity during the Chalcolithic Period at Yeghegis-1 Rockshelter (Armenia) reveals shifts in land use and social networks
Source: Sci Rep. 2024 Apr 25;14:9528. doi: 10.1038/s41598-024-59661-9 (PMC11045823; doi:10.1038/s41598-024-59661-9)
Supplement: Supplementary file 1 — Supplementary Information. [file 41598_2024_59661_MOESM1_ESM.pdf]

Increasing obsidian diversity during the Chalcolithic Period at Yeghegis-1 Rockshelter (Armenia) reveals shifts in land use and social networks

Ellery Frahm[1,2*], Mariam Saribekyan[3], Satenik Mkrtchyan[4,5], Laura Furquim[5], Ara Avagyan[6], Lilit Sahakyan[6], Karen Azatyan[7], Patrick Roberts[5], Ricardo Fernandes[5,8,9], Levon Yepiskoposyan[4], Noel Amano[5*], Mariya Antonosyan[5*]

1. Council on Archaeological Studies, Department of Anthropology, Yale University, United States
2. Anthropology Division, Peabody Museum of Natural History, Yale University, United States
3. Institute of Archaeology and Ethnography, National Academy of Sciences, Republic of Armenia
4. Institute of Molecular Biology, National Academy of Sciences, Republic of Armenia
5. Department of Archaeology, Max Planck Institute of Geoanthropology, Germany
6. Institute of Geological Sciences, National Academy of Sciences, Republic of Armenia
7. Yeghegnadzor Regional Museum, Republic of Armenia
8. Arne Faculty of Arts, Masaryk University, Brno, Czech Republic
9. Department of Bioarchaeology, Faculty of Archaeology, University of Warsaw, Warsaw, Poland.

* Corresponding authors

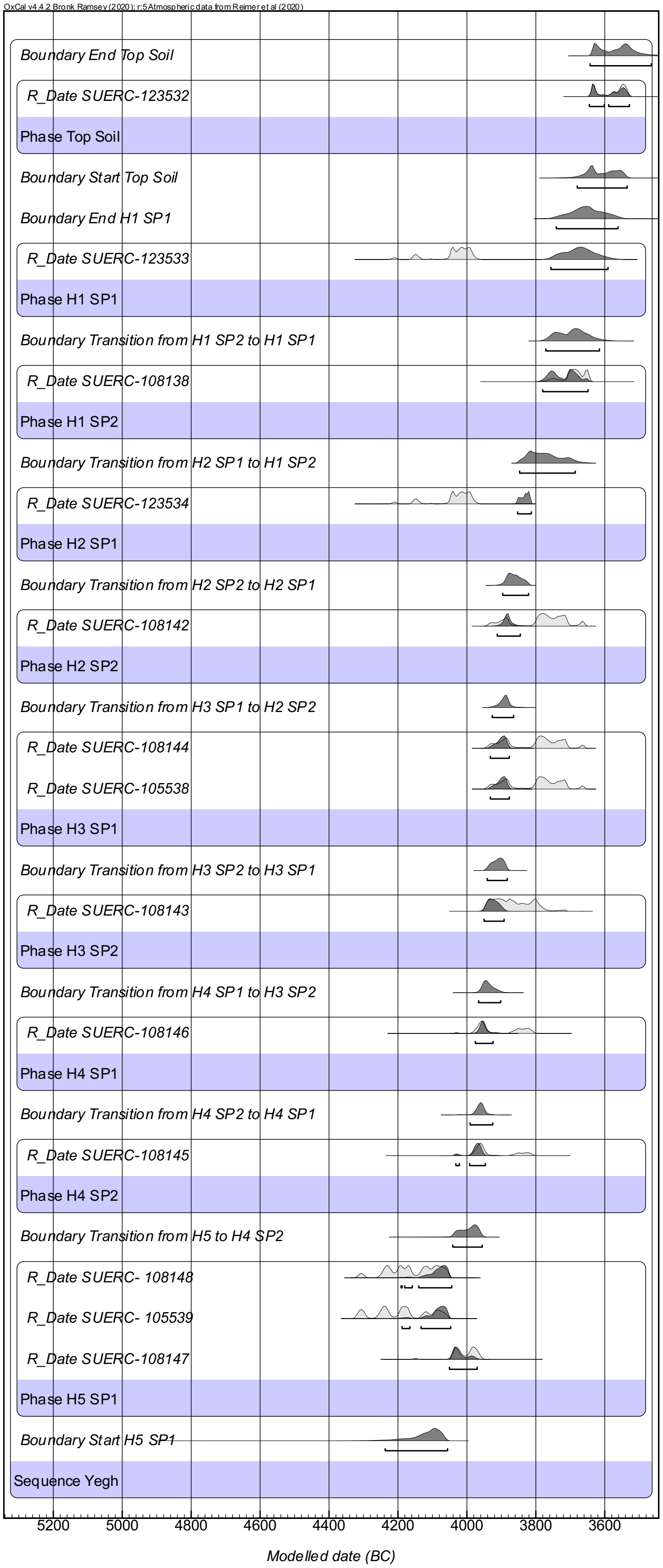
OxCal v4.4.2 Bronk Ramsey (2020); r:5 Atmospheric data from Reimer et al (2020)
Boundary End Top Soil
R_Date SUERC-123532
Phase Top Soil
Boundary Start Top Soil
Boundary End H1 SP1
R_Date SUERC-123533
Phase H1 SP1
Boundary Transition from H1 SP2 to H1 SP1
R_Date SUERC-108138
Phase H1 SP2
Boundary Transition from H2 SP1 to H1 SP2
R_Date SUERC-123534
Phase H2 SP1
Boundary Transition from H2 SP2 to H2 SP1
R_Date SUERC-108142
Phase H2 SP2
Boundary Transition from H3 SP1 to H2 SP2
R_Date SUERC-108144
R_Date SUERC-105538
Phase H3 SP1
Boundary Transition from H3 SP2 to H3 SP1
R_Date SUERC-108143
Phase H3 SP2
Boundary Transition from H4 SP1 to H3 SP2
R_Date SUERC-108146
Phase H4 SP1
Boundary Transition from H4 SP2 to H4 SP1
R_Date SUERC-108145
Phase H4 SP2
Boundary Transition from H5 to H4 SP2
R_Date SUERC- 108148
R_Date SUERC- 105539
R_Date SUERC-108147
Phase H5 SP1
Boundary Start H5 SP1
Sequence Yegh
5200
5000
4800
4600
4400
4200
4000
3800
3600
Modelled date (BC)

OxCal model code:

```
Plot()
 {
  Curve(‘IntCal20’,’IntCal20.14c’);
Outlier_Model(‘General’,T(5),U(0,4),’t’);

  Sequence(‘Yegh’)
  {
   Boundary('Start H5 SP1');
   Phase(‘H5 SP1’)
   {
R_Date('SUERC-108147', 5190, 27);
R_Date('SUERC- 105539', 5354, 27);
R_Date('SUERC- 108148', 5332, 27);
};
   Boundary('Transition from H5 to H4 SP2');

   Phase(‘H4 SP2’)
   {
R_Date('SUERC-108145', 5138, 27);
};
Boundary(‘Transition from H4 SP2 to H4 SP1’);

   Phase(‘H4 SP1’)
   {
R_Date('SUERC-108146', 5127, 27);
};

Boundary(‘Transition from H4 SP1 to H3 SP2’);

   Phase(‘H3 SP2’)
   {
R_Date('SUERC-108143', 5050, 27);
};
Boundary(‘Transition from H3 SP2 to H3 SP1’);

   Phase(‘H3 SP1’)
   {
R_Date('SUERC-105538', 5008, 27);
R_Date('SUERC-108144', 5008, 27);
};
Boundary(‘Transition from H3 SP1 to H2 SP2’);
   Phase(‘H2 SP2’)
```

```
  {
R_Date('SUERC-108142', 5000, 27);
};
Boundary('Transition from H2 SP2 to H2 SP1');
  Phase('H2 SP1')
  {
R_Date(' SUERC-123534', 5229, 24);
};
Boundary('Transition from H2 SP1 to H1 SP2');
  Phase('H1 SP2')
  {
R_Date('SUERC-108138', 4917, 27);
};
Boundary('Transition from H1 SP2 to H1 SP1');
  Phase('H1 SP1')
  {
R_Date('SUERC-123533', 5229, 24) {Outlier('General', 0.05);};

};

Boundary('End H1 SP1');
Boundary('Start Top Soil');

  Phase('Top Soil')
  {
R_Date('SUERC-123532', 4813, 24);
};
Boundary('End Top Soil');



};
};
```

Figure 4 datasets:

Lithic class (obsidian, by count):

Layer	debitage/flake	debitage/flake (<1 cm)	blade/bladelet	blade/bladelet, retouched	retouched/formal tool	core/core fragment
H0	121	124	1	2	1	
H1 S1	41	18	5	4	1	
H1 S2	307	129			3	
H2 S1	195	177	2	1		1
H2 S2	120	127	4	1	1	1
H3 S1	57	64	1			
H4 S1	21	15	3			1
H4 S2	59	11	1	1		
H5	58	51	1	1		2

Lithic class (obsidian, by mass):

Layer	debitage/flake	debitage/flake (<1 cm)	blade/bladelet	blade/bladelet, retouched	retouched/formal tool	core/core fragment
H0	124	27	6	12	30	
H1 S1	55	0.9	8.8	4.7	2	
H1 S2	373	37			18	
H2 S1	326	46.9	3.9	3		22
H2 S2	196	28	6.7	3	6	15
H3 S1	77	14	5			
H4 S1	34	3	2.7			13
H4 S2	80	3	2	0.9		
H5	129	16	0.9	6		48

Obsidian and other toolstone materials by mass and by count:

	Obsidian		Other	
**Class**	**N**	**g**	**N**	**g**
debitage/flake	980	1394	143	484
debitage/flake (<1 cm)	716	157.8	7	1.8
blade/bladelet	18	36	1	4
blade/bladelet, retouched	10	29.6	2	8
retouched/formal tool	6	56		
core/core fragment	5	98	11	1199

Specimen ID	Source Name	Group	Mn	Fe	Zn	Rb	Sr	Y	Zr	Nb	Rb/Zr	Sr/Zr	Y/Zr	Nb/Zr	Sr/Rb	Y/Rb	Zr/Rb	Nb/Rb
Aghvorik AR62.sK1a	Aghvorik	Aghvorik	480	12053	48	100	192	13	240	15	0.42	0.80	0.06	0.06	1.91	0.13	2.39	0.15
Aghvorik AR62.sK1c	Aghvorik	Aghvorik	494	12129	50	103	194	16	245	14	0.42	0.79	0.07	0.06	1.88	0.16	2.37	0.14
Aghvorik AR62.sK1d	Aghvorik	Aghvorik	473	12173	46	101	191	14	240	14	0.42	0.80	0.06	0.06	1.88	0.14	2.37	0.14
Aghvorik AR62.sK1e	Aghvorik	Aghvorik	503	12205	49	99	195	15	240	15	0.41	0.81	0.06	0.06	1.96	0.15	2.41	0.16
Aghvorik AR63.kM1a	Aghvorik	Aghvorik	470	12098	53	99	197	18	243	15	0.41	0.81	0.07	0.06	1.98	0.18	2.44	0.16
Aghvorik AR63.kM1b	Aghvorik	Aghvorik	469	12192	51	101	193	17	246	17	0.41	0.79	0.07	0.07	1.90	0.17	2.42	0.17
Aghvorik AR66.rB1	Aghvorik	Aghvorik	500	12522	48	101	197	17	247	14	0.41	0.80	0.07	0.06	1.94	0.17	2.43	0.14
Aghvorik AR66.rB2	Aghvorik	Aghvorik	508	12360	48	103	196	16	247	13	0.42	0.79	0.07	0.05	1.90	0.16	2.39	0.12
Mets Arteni AR67.rB1	Mets Arteni	Arteni	715	3538	46	149	13	36	70	44	2.14	0.18	0.52	0.64	0.08	0.24	0.47	0.30
Mets Arteni AR67.rB2	Mets Arteni	Arteni	733	3518	46	154	14	38	70	37	2.21	0.19	0.54	0.54	0.09	0.25	0.45	0.24
Mets Arteni AR67.rB3	Mets Arteni	Arteni	733	3531	44	149	14	35	67	46	2.23	0.20	0.52	0.68	0.09	0.23	0.45	0.31
Mets Arteni AR75.rB1a	Mets Arteni	Arteni	756	3578	45	151	13	34	68	43	2.22	0.18	0.50	0.63	0.08	0.23	0.45	0.28
Mets Arteni AR75.rB1b	Mets Arteni	Arteni	779	3574	47	152	13	37	68	42	2.24	0.18	0.54	0.61	0.08	0.24	0.45	0.27
Mets Arteni AR75.rB1c	Mets Arteni	Arteni	768	3577	45	151	13	35	66	42	2.29	0.19	0.53	0.63	0.08	0.23	0.44	0.28
Mets Arteni AR75.rB1d	Mets Arteni	Arteni	749	3555	44	154	14	35	73	43	2.09	0.18	0.48	0.58	0.09	0.23	0.48	0.28
Mets Arteni GPS 61a	Mets Arteni	Arteni	786	3627	45	152	15	32	62	40	2.44	0.23	0.52	0.65	0.10	0.21	0.41	0.26
Mets Arteni GPS 61b	Mets Arteni	Arteni	757	3565	49	149	15	33	68	39	2.20	0.21	0.49	0.57	0.10	0.22	0.46	0.26
Mets Arteni GPS 61c	Mets Arteni	Arteni	710	3758	40	151	21	32	69	39	2.19	0.30	0.47	0.56	0.14	0.21	0.46	0.26
Mets Arteni GPS 61d	Mets Arteni	Arteni	776	3542	50	149	13	32	64	43	2.33	0.20	0.50	0.67	0.08	0.22	0.43	0.29
Pokr Arteni-1 AR05a	Pokr Arteni-1	Arteni	579	4091	40	133	25	28	84	32	1.59	0.29	0.34	0.38	0.18	0.21	0.63	0.24
Pokr Arteni-1 AR05b	Pokr Arteni-1	Arteni	636	3975	38	139	22	27	85	32	1.64	0.25	0.32	0.38	0.15	0.20	0.61	0.23
Pokr Arteni-1 AR41.sK1	Pokr Arteni-1	Arteni	620	3977	39	137	21	27	77	32	1.78	0.27	0.36	0.41	0.15	0.20	0.56	0.23
Pokr Arteni-1 AR41.sK2	Pokr Arteni-1	Arteni	650	3952	40	136	21	28	84	33	1.62	0.24	0.34	0.40	0.15	0.21	0.62	0.24
Pokr Arteni-1 AR42.kM1	Pokr Arteni-1	Arteni	621	4094	39	132	25	26	85	32	1.56	0.29	0.30	0.38	0.19	0.19	0.64	0.24
Pokr Arteni-1 AR42.kM2	Pokr Arteni-1	Arteni	587	4241	43	129	27	25	80	29	1.60	0.33	0.31	0.36	0.21	0.19	0.62	0.23
Pokr Arteni-1 AR68.rB1	Pokr Arteni-1	Arteni	594	4122	40	132	24	27	83	31	1.60	0.28	0.33	0.37	0.18	0.21	0.63	0.23
Pokr Arteni-1 AR68.rB2	Pokr Arteni-1	Arteni	596	4069	37	130	24	27	80	26	1.62	0.29	0.33	0.33	0.18	0.20	0.62	0.20
Pokr Arteni-1 AR68.rB3	Pokr Arteni-1	Arteni	609	3981	38	133	23	29	81	29	1.65	0.28	0.36	0.36	0.17	0.22	0.61	0.22
Pokr Arteni-1 AR68.rB4	Pokr Arteni-1	Arteni	643	4001	40	133	23	27	82	32	1.63	0.27	0.33	0.39	0.17	0.21	0.61	0.24
Pokr Arteni-1 AR68.rB6	Pokr Arteni-1	Arteni	615	4181	40	129	28	24	87	29	1.48	0.32	0.27	0.34	0.21	0.18	0.67	0.23
Pokr Arteni-2 AR18.avH1	Pokr Arteni-2	Arteni	546	4494	36	122	37	25	87	28	1.40	0.42	0.28	0.32	0.30	0.20	0.71	0.23
Pokr Arteni-2 AR19.avH1	Pokr Arteni-2	Arteni	561	4427	35	125	37	25	84	26	1.49	0.44	0.29	0.32	0.29	0.20	0.67	0.21
Pokr Arteni-2 AR20.avH1	Pokr Arteni-2	Arteni	557	4425	36	124	37	23	86	26	1.44	0.43	0.27	0.31	0.30	0.18	0.69	0.21
Pokr Arteni-2 AR68.rB5a	Pokr Arteni-2	Arteni	584	4349	39	123	36	20	84	29	1.46	0.42	0.24	0.35	0.29	0.16	0.68	0.24
Pokr Arteni-2 AR68.rB5b	Pokr Arteni-2	Arteni	579	4419	36	125	35	24	84	29	1.49	0.41	0.28	0.35	0.28	0.19	0.67	0.23
Chikiani-1 GE08.rB2a	Chikiani-1	Chikiani	495	5155	41	129	76	16	84	18	1.53	0.90	0.19	0.22	0.59	0.13	0.65	0.14
Chikiani-1 GE08.rB2b	Chikiani-1	Chikiani	534	5112	44	134	76	16	80	14	1.68	0.94	0.20	0.18	0.56	0.12	0.60	0.10
Chikiani-1 GE12.nS1a	Chikiani-1	Chikiani	503	5004	49	133	75	17	82	17	1.63	0.91	0.21	0.21	0.56	0.13	0.61	0.13
Chikiani-1 GE12.nS1b	Chikiani-1	Chikiani	489	5070	46	132	74	15	81	20	1.63	0.91	0.19	0.24	0.56	0.12	0.61	0.15
Chikiani-1 GE12.nS1c	Chikiani-1	Chikiani	505	5115	45	131	75	14	81	18	1.62	0.92	0.18	0.22	0.57	0.11	0.62	0.14
Chikiani-1 Specimen5	Chikiani-1	Chikiani	486	5172	43	132	75	13	78	18	1.69	0.95	0.17	0.23	0.56	0.10	0.59	0.14
Chikiani-2a GE07.kM1a	Chikiani-2a	Chikiani	465	5553	46	130	86	14	91	14	1.43	0.94	0.16	0.15	0.66	0.11	0.70	0.11
Chikiani-2a GE07.kM1b	Chikiani-2a	Chikiani	515	5674	40	130	87	13	98	18	1.33	0.88	0.14	0.19	0.66	0.10	0.75	0.14
Chikiani-2a GE07.kM1c	Chikiani-2a	Chikiani	464	5508	46	129	83	15	95	18	1.35	0.87	0.16	0.19	0.64	0.12	0.74	0.14
Chikiani-2a Specimen4a	Chikiani-2a	Chikiani	484	5560	44	132	88	15	97	15	1.36	0.90	0.16	0.16	0.66	0.12	0.73	0.12
Chikiani-2a Specimen4b	Chikiani-2a	Chikiani	449	5457	43	130	83	15	92	15	1.40	0.89	0.16	0.17	0.64	0.12	0.71	0.12
Chikiani-2b GE02.iD1a	Chikiani-2b	Chikiani	474	5731	43	129	92	15	101	14	1.27	0.91	0.15	0.14	0.71	0.12	0.78	0.11
Chikiani-2b GE02.iD1b	Chikiani-2b	Chikiani	460	5614	46	128	89	13	101	15	1.26	0.88	0.13	0.15	0.70	0.10	0.79	0.12
Chikiani-2b GE02.iD1c	Chikiani-2b	Chikiani	496	5688	44	129	89	14	100	17	1.29	0.89	0.14	0.17	0.69	0.11	0.78	0.13
Chikiani-2b GE02.iD1d	Chikiani-2b	Chikiani	456	5777	47	125	92	15	108	17	1.15	0.85	0.14	0.16	0.74	0.12	0.87	0.14
Chikiani-2b GE02.iD1e	Chikiani-2b	Chikiani	463	5674	44	130	90	14	97	15	1.34	0.92	0.15	0.16	0.69	0.11	0.74	0.12
Chikiani-2b GE13.nS1	Chikiani-2b	Chikiani	486	5801	44	128	91	14	103	14	1.24	0.88	0.14	0.14	0.71	0.11	0.81	0.11
Chikiani-2b GE13.nS2a	Chikiani-2b	Chikiani	437	5591	45	121	89	15	99	17	1.22	0.90	0.15	0.17	0.73	0.13	0.82	0.14
Chikiani-2b GE13.nS2b	Chikiani-2b	Chikiani	456	5795	42	126	95	16	105	13	1.20	0.90	0.15	0.12	0.75	0.13	0.83	0.10
Chikiani-2b GE13.nS2c	Chikiani-2b	Chikiani	435	5750	42	123	92	15	105	13	1.17	0.88	0.15	0.12	0.75	0.12	0.85	0.10
Chikiani-2b Specimen3a	Chikiani-2b	Chikiani	475	5865	46	125	92	15	107	15	1.17	0.86	0.14	0.15	0.74	0.12	0.85	0.12
Chikiani-2b Specimen3b	Chikiani-2b	Chikiani	463	5758	44	126	93	15	104	15	1.21	0.89	0.15	0.15	0.74	0.12	0.83	0.12
Chikiani-2b Specimen3c	Chikiani-2b	Chikiani	453	5700	45	124	89	14	99	15	1.25	0.90	0.14	0.16	0.72	0.12	0.80	0.12
Chikiani-3 GE09.nS1a	Chikiani-3	Chikiani	459	5900	43	123	93	14	106	14	1.16	0.88	0.14	0.13	0.76	0.12	0.86	0.11
Chikiani-3 GE09.nS1b	Chikiani-3	Chikiani	455	5949	49	123	96	14	103	15	1.19	0.93	0.14	0.15	0.78	0.12	0.84	0.13
Geghasar-1 AR17.010a	Geghasar-1	Gegham 1	707	3580	35	217	9	25	63	55	3.44	0.15	0.39	0.88	0.04	0.11	0.29	0.25
Geghasar-1 AR17.010b	Geghasar-1	Gegham 1	705	3565	35	215	13	27	63	57	3.41	0.20	0.42	0.90	0.06	0.12	0.29	0.26
Geghasar-1 AR17.010c	Geghasar-1	Gegham 1	702	3649	33	222	9	26	64	57	3.46	0.15	0.40	0.88	0.04	0.12	0.29	0.26
Geghasar-1 AR51.sK1	Geghasar-1	Gegham 1	648	3826	34	198	15	24	75	53	2.63	0.19	0.31	0.70	0.07	0.12	0.38	0.26
Geghasar-1 AR52.sK1	Geghasar-1	Gegham 1	671	3684	31	207	11	24	69	53	3.01	0.17	0.34	0.76	0.06	0.11	0.33	0.25

Geghasar-1 AR54.kM1a	Geghasar-1	Gegham 1	701	3716	35	206	13	26	68	51	3.04	0.18	0.38	0.75	0.06	0.12	0.33	0.25
Geghasar-1 AR54.kM1b	Geghasar-1	Gegham 1	685	3784	34	204	11	24	70	54	2.93	0.16	0.34	0.77	0.06	0.12	0.34	0.26
Geghasar-1 AR55.sK1a	Geghasar-1	Gegham 1	658	3783	30	202	13	25	70	53	2.90	0.18	0.35	0.75	0.06	0.12	0.34	0.26
Geghasar-1 AR55.sK1b	Geghasar-1	Gegham 1	672	3716	31	207	10	23	69	54	3.01	0.15	0.33	0.78	0.05	0.11	0.33	0.26
Geghasar-1 AR79.rB2	Geghasar-1	Gegham 1	672	3701	35	205	14	25	72	53	2.87	0.19	0.34	0.73	0.07	0.12	0.35	0.26
Spitakasar-1 2017-1a	Spitakasar-1	Gegham 1	793	3637	35	183	10	22	58	54	3.13	0.18	0.37	0.92	0.06	0.12	0.32	0.29
Spitakasar-1 2017-1b	Spitakasar-1	Gegham 1	814	3647	38	188	11	24	59	51	3.17	0.19	0.40	0.86	0.06	0.13	0.32	0.27
Spitakasar-1 2017-2b	Spitakasar-1	Gegham 1	823	3646	35	186	10	25	57	53	3.24	0.18	0.43	0.91	0.06	0.13	0.31	0.28
Spitakasar-1 2017-3a	Spitakasar-1	Gegham 1	849	3536	36	185	11	23	56	53	3.33	0.21	0.41	0.95	0.06	0.12	0.30	0.28
Spitakasar-1 2017-3b	Spitakasar-1	Gegham 1	840	3696	35	180	11	21	53	54	3.42	0.22	0.40	1.02	0.06	0.12	0.29	0.30
Spitakasar-1 2017-4a	Spitakasar-1	Gegham 1	808	3584	37	186	11	25	55	55	3.41	0.21	0.45	1.01	0.06	0.13	0.29	0.30
Spitakasar-1 2017-4b	Spitakasar-1	Gegham 1	815	3605	37	185	10	22	53	51	3.51	0.20	0.41	0.97	0.06	0.12	0.28	0.28
Spitakasar-1 2017-5a	Spitakasar-1	Gegham 1	806	3609	36	182	10	22	51	51	3.58	0.21	0.43	1.01	0.06	0.12	0.28	0.28
Spitakasar-1 2017-5b	Spitakasar-1	Gegham 1	808	3589	31	183	9	23	55	54	3.35	0.17	0.42	0.99	0.05	0.12	0.30	0.29
Spitakasar-1 2017-6a	Spitakasar-1	Gegham 1	842	3697	36	181	11	24	54	53	3.37	0.21	0.44	0.98	0.06	0.13	0.30	0.29
Spitakasar-1 2017-6b	Spitakasar-1	Gegham 1	859	3602	38	179	11	21	53	54	3.40	0.22	0.40	1.02	0.06	0.12	0.29	0.30
Spitakasar/Geghasar-2 AR53a	Spitakasar/Geghasar-2	Gegham 2	537	6000	44	111	52	26	112	28	0.99	0.46	0.23	0.25	0.46	0.23	1.01	0.25
Spitakasar/Geghasar-2 AR53b	Spitakasar/Geghasar-2	Gegham 2	551	5972	41	115	54	27	117	26	0.98	0.46	0.23	0.23	0.47	0.23	1.02	0.23
Spitakasar/Geghasar-2 AR53c	Spitakasar/Geghasar-2	Gegham 2	571	5904	44	110	53	27	114	29	0.96	0.46	0.23	0.26	0.48	0.24	1.04	0.27
Spitakasar/Geghasar-2 AR53d	Spitakasar/Geghasar-2	Gegham 2	556	6024	46	114	55	26	114	28	1.00	0.48	0.22	0.24	0.48	0.22	1.00	0.24
Spitakasar/Geghasar-2 AR53e	Spitakasar/Geghasar-2	Gegham 2	595	5925	41	111	55	26	113	29	0.98	0.48	0.23	0.26	0.49	0.23	1.02	0.26
Spitakasar/Geghasar-2 AR53f	Spitakasar/Geghasar-2	Gegham 2	611	5912	44	112	53	27	115	29	0.97	0.46	0.23	0.25	0.47	0.24	1.03	0.26
Gutansar AR21.avH1	Gutansar	Gutansar	627	8244	45	142	124	23	173	37	0.82	0.72	0.13	0.22	0.87	0.16	1.22	0.26
Gutansar AR22.avH1	Gutansar	Gutansar	632	8198	46	143	125	23	170	37	0.84	0.73	0.13	0.22	0.87	0.16	1.19	0.26
Gutansar AR44.sK1	Gutansar	Gutansar	616	8296	44	141	128	21	173	37	0.82	0.74	0.12	0.22	0.90	0.15	1.22	0.26
Gutansar AR45.kM1a	Gutansar	Gutansar	619	8064	42	141	124	25	169	36	0.84	0.73	0.15	0.21	0.88	0.17	1.20	0.26
Gutansar AR45.kM1b	Gutansar	Gutansar	652	8220	46	144	125	23	173	36	0.83	0.72	0.13	0.21	0.87	0.16	1.20	0.25
Gutansar AR46.sK1a	Gutansar	Gutansar	678	8260	40	143	132	24	177	37	0.81	0.75	0.13	0.21	0.92	0.17	1.23	0.26
Gutansar AR46.sK1b	Gutansar	Gutansar	642	8405	43	144	131	25	177	36	0.82	0.74	0.14	0.20	0.91	0.17	1.23	0.25
Gutansar AR46.sK1c	Gutansar	Gutansar	645	8431	48	141	130	25	177	36	0.80	0.73	0.14	0.20	0.92	0.17	1.26	0.26
Gutansar AR47.kM3	Gutansar	Gutansar	662	8067	38	141	120	22	166	35	0.85	0.72	0.13	0.21	0.85	0.15	1.18	0.25
Gutansar AR49.sK1	Gutansar	Gutansar	637	7663	40	145	122	22	168	37	0.86	0.72	0.13	0.22	0.84	0.15	1.16	0.26
Gutansar AR50.sK2a	Gutansar	Gutansar	614	8101	48	143	120	24	166	39	0.86	0.72	0.14	0.23	0.84	0.17	1.16	0.27
Gutansar AR50.sK2b	Gutansar	Gutansar	622	8080	40	142	120	24	167	36	0.85	0.72	0.14	0.22	0.84	0.17	1.18	0.25
Gutansar AR72.rB1	Gutansar	Gutansar	662	8243	40	142	124	24	174	35	0.82	0.71	0.14	0.20	0.87	0.17	1.22	0.24
Gutansar AR76.rB1a	Gutansar	Gutansar	631	8135	48	142	126	24	172	40	0.83	0.73	0.14	0.23	0.88	0.17	1.21	0.28
Gutansar AR76.rB1b	Gutansar	Gutansar	646	8626	46	139	123	25	169	37	0.82	0.73	0.15	0.22	0.88	0.18	1.21	0.27
Gutansar AR76.rB3a	Gutansar	Gutansar	645	8213	46	143	127	23	171	40	0.84	0.74	0.13	0.23	0.89	0.16	1.19	0.28
Gutansar AR77.rB2a	Gutansar	Gutansar	643	8509	43	137	121	24	170	36	0.81	0.71	0.14	0.21	0.88	0.17	1.24	0.26
Gutansar AR77.rB2b	Gutansar	Gutansar	612	8146	39	141	124	24	169	36	0.84	0.73	0.14	0.21	0.88	0.17	1.20	0.26
Gutansar AR77.rB3a	Gutansar	Gutansar	642	8157	42	140	124	21	169	36	0.83	0.73	0.12	0.21	0.88	0.15	1.21	0.26
Gutansar AR77.rB3b	Gutansar	Gutansar	637	7802	44	136	121	24	163	36	0.83	0.74	0.15	0.22	0.89	0.17	1.20	0.26
Gutansar AR78.rB1	Gutansar	Gutansar	633	8228	38	143	130	21	172	32	0.83	0.76	0.12	0.19	0.91	0.15	1.20	0.22
Gutansar AR78.rB2	Gutansar	Gutansar	625	8192	42	136	131	23	172	36	0.79	0.76	0.13	0.21	0.96	0.17	1.26	0.26
Gutansar AR78.rB3a	Gutansar	Gutansar	615	8370	44	145	130	23	177	42	0.82	0.73	0.13	0.23	0.89	0.16	1.22	0.29
Gutansar AR78.rB3b	Gutansar	Gutansar	640	8353	45	147	128	23	174	36	0.85	0.74	0.13	0.21	0.87	0.15	1.18	0.25
Hatis-Alpha AR.2016.1a	Hatis-Alpha	Hatis	529	6350	35	111	110	18	92	21	1.20	1.19	0.20	0.23	0.99	0.16	0.83	0.19
Hatis-Alpha AR.2016.1b	Hatis-Alpha	Hatis	514	6409	37	115	112	18	93	22	1.23	1.20	0.19	0.24	0.97	0.16	0.81	0.19
Hatis-Alpha AR.2016.1c	Hatis-Alpha	Hatis	525	6242	35	111	110	15	92	22	1.20	1.19	0.16	0.24	0.99	0.14	0.83	0.20
Hatis-Alpha AR.2016.1d	Hatis-Alpha	Hatis	469	6253	36	112	110	18	95	24	1.18	1.15	0.19	0.25	0.98	0.16	0.85	0.21
Hatis-Alpha AR.2016.1e	Hatis-Alpha	Hatis	496	6293	35	114	110	16	91	18	1.26	1.21	0.18	0.20	0.96	0.14	0.79	0.16
Hatis-Alpha AR.2016.3a	Hatis-Alpha	Hatis	510	6441	37	112	112	17	96	20	1.16	1.16	0.18	0.20	1.00	0.15	0.86	0.17
Hatis-Alpha AR.2016.3b	Hatis-Alpha	Hatis	518	6486	35	112	112	15	97	22	1.15	1.15	0.16	0.23	1.00	0.14	0.87	0.20
Hatis-Alpha AR.2016.3c	Hatis-Alpha	Hatis	501	6499	35	111	113	16	94	22	1.18	1.20	0.17	0.24	1.01	0.15	0.85	0.20
Hatis-Alpha AR.2016.3d	Hatis-Alpha	Hatis	495	6402	35	112	113	17	91	20	1.24	1.25	0.19	0.22	1.01	0.15	0.81	0.17
Hatis-Alpha AR59.kM1a	Hatis-Alpha	Hatis	551	6341	37	116	111	17	91	22	1.28	1.22	0.19	0.25	0.96	0.15	0.78	0.19
Hatis-Alpha AR59.kM1b	Hatis-Alpha	Hatis	495	6227	36	113	111	14	92	21	1.22	1.20	0.15	0.23	0.98	0.13	0.82	0.19
Hatis-Alpha AR72.rB3a	Hatis-Alpha	Hatis	519	6364	38	113	109	15	91	20	1.24	1.19	0.17	0.21	0.96	0.13	0.81	0.17
Hatis-Alpha AR72.rB3b	Hatis-Alpha	Hatis	503	6197	33	114	106	15	91	25	1.26	1.17	0.17	0.28	0.93	0.13	0.79	0.22
Hatis-Alpha AR74.rB1	Hatis-Alpha	Hatis	525	6231	36	114	110	17	93	22	1.22	1.18	0.18	0.24	0.96	0.15	0.82	0.20
Hatis-Alpha AR74.rB2a	Hatis-Alpha	Hatis	515	6392	36	109	112	15	91	20	1.19	1.22	0.17	0.21	1.02	0.14	0.84	0.18
Hatis-Alpha AR74.rB2b	Hatis-Alpha	Hatis	496	6298	36	111	109	15	94	22	1.18	1.15	0.16	0.24	0.98	0.14	0.85	0.20
Hatis-Beta AR.72.rB2b	Hatis-Beta	Hatis	542	7743	36	100	136	16	104	20	0.97	1.31	0.16	0.19	1.35	0.16	1.03	0.20
Hatis-Beta AR48.sK1a	Hatis-Beta	Hatis	496	7606	44	105	134	17	105	18	1.01	1.28	0.16	0.17	1.27	0.16	0.99	0.17
Hatis-Beta AR48.sK1b	Hatis-Beta	Hatis	509	7659	37	108	136	13	102	17	1.06	1.33	0.13	0.17	1.26	0.12	0.94	0.16
Hatis-Beta AR72.rB2a	Hatis-Beta	Hatis	541	7827	41	103	139	14	101	18	1.02	1.38	0.14	0.18	1.34	0.14	0.98	0.18

Hatis-Beta GPS 58.1	Hatis-Beta	Hatis	528	7184	35	108	129	15	103	21	1.05	1.25	0.15	0.20	1.19	0.14	0.95	0.19
Hatis-Beta GPS 58.2	Hatis-Beta	Hatis	483	7181	44	109	125	15	101	22	1.08	1.24	0.15	0.22	1.14	0.14	0.92	0.20
Hatis-Beta GPS 58.3	Hatis-Beta	Hatis	489	7184	35	112	127	15	99	20	1.13	1.28	0.15	0.20	1.13	0.14	0.88	0.17
Hatis-Beta GPS 58.4	Hatis-Beta	Hatis	514	7169	38	110	128	15	98	18	1.12	1.30	0.16	0.19	1.16	0.14	0.89	0.17
Hatis-Beta GPS 58.5	Hatis-Beta	Hatis	531	7220	37	105	126	15	97	21	1.08	1.29	0.16	0.22	1.19	0.14	0.92	0.20
Hatis-Beta GPS 58.6	Hatis-Beta	Hatis	534	7235	37	110	131	16	101	15	1.09	1.30	0.16	0.15	1.19	0.15	0.92	0.14
Hatis-Delta 2011a	Hatis-Delta	Hatis	544	10748	42	94	204	16	135	17	0.69	1.51	0.12	0.12	2.18	0.17	1.44	0.18
Hatis-Delta 2011b	Hatis-Delta	Hatis	533	10773	40	96	207	16	130	17	0.74	1.59	0.12	0.13	2.14	0.17	1.35	0.17
Hatis-Delta 2011c	Hatis-Delta	Hatis	478	10790	40	96	204	15	132	21	0.72	1.54	0.12	0.16	2.13	0.16	1.38	0.22
Hatis-Gamma AR.2016.2a	Hatis-Gamma	Hatis	509	9324	36	100	172	14	116	21	0.87	1.48	0.12	0.18	1.71	0.14	1.16	0.21
Hatis-Gamma AR.2016.2b	Hatis-Gamma	Hatis	498	9242	36	101	168	13	114	18	0.89	1.47	0.12	0.16	1.66	0.13	1.13	0.18
Hatis-Gamma AR.2016.2c	Hatis-Gamma	Hatis	531	9276	40	102	175	13	117	21	0.87	1.49	0.11	0.18	1.71	0.13	1.14	0.20
Hatis-Gamma AR.2016.2d	Hatis-Gamma	Hatis	500	9254	39	102	170	15	114	22	0.90	1.49	0.13	0.20	1.66	0.15	1.12	0.22
Hatis-Gamma GPS 34a	Hatis-Gamma	Hatis	533	8918	40	104	168	14	117	21	0.89	1.43	0.12	0.18	1.61	0.14	1.12	0.20
Hatis-Gamma GPS 34b	Hatis-Gamma	Hatis	463	8973	37	102	164	16	115	18	0.89	1.42	0.14	0.16	1.60	0.16	1.12	0.18
Kars-Arpaçay 1 EA65a	Kars-Arpaçay 1	Kars-Arpaçay	580	6818	63	132	10	46	210	28	0.63	0.05	0.22	0.13	0.08	0.35	1.58	0.21
Kars-Arpaçay 1 EA65b	Kars-Arpaçay 1	Kars-Arpaçay	662	7237	70	142	11	48	219	28	0.65	0.05	0.22	0.13	0.08	0.34	1.54	0.20
Kars-Arpaçay 1 EA65c	Kars-Arpaçay 1	Kars-Arpaçay	682	7281	64	140	11	48	225	26	0.62	0.05	0.21	0.12	0.08	0.34	1.60	0.19
Kars-Arpaçay 1 EA65d	Kars-Arpaçay 1	Kars-Arpaçay	605	6895	62	130	11	43	201	24	0.64	0.06	0.22	0.12	0.09	0.34	1.55	0.18
Kars-Arpaçay-2 EA40a	Kars-Arpaçay 2	Kars-Arpaçay	721	10303	68	131	41	43	215	20	0.61	0.19	0.20	0.09	0.31	0.32	1.64	0.15
Kars-Arpaçay-2 EA40b	Kars-Arpaçay 2	Kars-Arpaçay	721	10141	73	131	41	42	211	18	0.62	0.19	0.20	0.09	0.31	0.32	1.60	0.14
Kars-Arpaçay-2 EA40c	Kars-Arpaçay 2	Kars-Arpaçay	601	9243	57	112	37	39	198	17	0.56	0.18	0.20	0.08	0.33	0.35	1.77	0.15
Kars-Arpaçay-2 EA66a	Kars-Arpaçay 2	Kars-Arpaçay	718	9279	68	130	45	46	267	24	0.48	0.17	0.17	0.09	0.34	0.36	2.06	0.18
Kars-Arpaçay-2 EA66b	Kars-Arpaçay 2	Kars-Arpaçay	652	8766	65	126	54	46	251	21	0.50	0.21	0.18	0.08	0.43	0.37	2.00	0.17
Kelbadjar AZ02	Kelbadjar	Kelbadjar	526	5030	45	153	16	11	110	35	1.38	0.14	0.10	0.31	0.10	0.07	0.72	0.23
Kelbadjar AZ03	Kelbadjar	Kelbadjar	470	5030	39	154	16	11	102	33	1.51	0.15	0.10	0.33	0.10	0.07	0.66	0.22
Kelbadjar AZ04	Kelbadjar	Kelbadjar	450	4848	38	148	16	11	108	32	1.36	0.14	0.10	0.29	0.10	0.07	0.73	0.22
Khorapor AR37.ipS1a	Khorapor	Khorapor	455	3930	27	210	9	11	76	35	2.75	0.12	0.15	0.45	0.05	0.05	0.36	0.16
Khorapor AR37.ipS1b	Khorapor	Khorapor	448	3844	29	207	8	11	77	29	2.68	0.11	0.14	0.38	0.04	0.05	0.37	0.14
Khorapor AR61.sK1a	Khorapor	Khorapor	499	3994	31	221	6	14	79	35	2.79	0.08	0.18	0.44	0.03	0.06	0.36	0.16
Khorapor AR61.sK1b	Khorapor	Khorapor	425	3886	31	215	7	14	80	36	2.68	0.09	0.18	0.45	0.03	0.07	0.37	0.17
Khorapor AR61.sK1c	Khorapor	Khorapor	454	3905	31	221	5	14	78	39	2.82	0.07	0.18	0.50	0.02	0.06	0.35	0.18
Khorapor AR61.sK1d	Khorapor	Khorapor	440	3863	31	213	7	12	80	39	2.66	0.09	0.15	0.48	0.04	0.06	0.38	0.18
Meydan Dag EA09a	Meydan Dağ	Meydan Dağ	569	9672	77	204	21	56	282	29	0.73	0.07	0.20	0.10	0.10	0.27	1.38	0.14
Meydan Dag EA09b	Meydan Dağ	Meydan Dağ	602	9693	79	207	22	57	282	29	0.73	0.08	0.20	0.10	0.10	0.27	1.36	0.14
Meydan Dag EA68-1a	Meydan Dağ	Meydan Dağ	537	9602	78	207	24	52	282	28	0.74	0.08	0.18	0.10	0.11	0.25	1.36	0.13
Meydan Dag EA68-1b	Meydan Dağ	Meydan Dağ	547	9727	75	201	25	54	282	29	0.72	0.09	0.19	0.10	0.12	0.27	1.40	0.14
Meydan Dag EA68-2a	Meydan Dağ	Meydan Dağ	574	9615	74	202	22	53	280	26	0.72	0.08	0.19	0.09	0.11	0.26	1.38	0.13
Meydan Dag EA68-2b	Meydan Dağ	Meydan Dağ	535	9726	82	202	25	56	285	29	0.71	0.09	0.20	0.10	0.12	0.28	1.41	0.14
Meydan Dag EA69-1	Meydan Dağ	Meydan Dağ	582	9707	80	205	22	53	281	29	0.73	0.08	0.19	0.10	0.10	0.26	1.37	0.14
Meydan Dag EA69-2a	Meydan Dağ	Meydan Dağ	542	9690	68	203	25	52	284	29	0.72	0.09	0.18	0.10	0.12	0.26	1.40	0.14
Meydan Dag EA69-2b	Meydan Dağ	Meydan Dağ	530	9654	80	208	26	55	288	28	0.72	0.09	0.19	0.10	0.12	0.26	1.38	0.13
Sarıkamış-1 Ea	Sarıkamış-1	Sarıkamış	337	5774	34	134	26	24	108	4	1.25	0.24	0.22	0.04	0.19	0.18	0.80	0.03
Sarıkamış-1 EA04a	Sarıkamış-1	Sarıkamış	373	5788	33	127	26	23	108	9	1.17	0.24	0.21	0.08	0.20	0.18	0.86	0.07
Sarıkamış-1 Eb	Sarıkamış-1	Sarıkamış	372	5782	37	134	28	24	109	10	1.23	0.25	0.22	0.09	0.20	0.18	0.81	0.07
Sarıkamış-1 Ec	Sarıkamış-1	Sarıkamış	348	5773	34	132	27	23	108	9	1.22	0.24	0.21	0.08	0.20	0.17	0.82	0.06
Sarıkamış-1 Ed	Sarıkamış-1	Sarıkamış	342	5671	32	125	24	24	107	6	1.17	0.22	0.22	0.05	0.19	0.19	0.85	0.05
Sarıkamış-1a EA67a	Sarıkamış-1a	Sarıkamış	237	7690	30	146	26	31	161	11	0.90	0.16	0.19	0.07	0.17	0.21	1.11	0.08
Sarıkamış-1a EA67b	Sarıkamış-1a	Sarıkamış	359	8921	38	142	26	28	166	10	0.86	0.15	0.17	0.06	0.18	0.20	1.17	0.07
Sarıkamış-2 EA63a	Sarıkamış-2	Sarıkamış	349	5597	35	133	26	23	101	11	1.32	0.25	0.23	0.11	0.19	0.17	0.76	0.09
Sarıkamış-2 EA63b	Sarıkamış-2	Sarıkamış	357	5345	35	137	23	25	92	9	1.49	0.24	0.27	0.09	0.16	0.18	0.67	0.06
Sarıkamış-2 EA63c	Sarıkamış-2	Sarıkamış	390	5412	36	136	24	25	92	7	1.48	0.25	0.27	0.08	0.17	0.18	0.68	0.05
Sarıkamış-2 EA63d	Sarıkamış-2	Sarıkamış	395	5327	34	134	25	24	94	11	1.43	0.26	0.25	0.12	0.18	0.18	0.70	0.08
Sarıkamış-2 EA63e	Sarıkamış-2	Sarıkamış	389	5495	34	138	27	23	99	10	1.40	0.27	0.23	0.10	0.19	0.16	0.72	0.07
Sarıkamış-2 EA63f	Sarıkamış-2	Sarıkamış	390	5353	32	135	24	22	96	10	1.41	0.24	0.23	0.10	0.17	0.16	0.71	0.07
Bazenk AR38.ipS1	Bazenk	Syunik	541	4211	38	191	6	7	90	35	2.13	0.07	0.08	0.39	0.03	0.04	0.47	0.18
Bazenk AR58.sK1a	Bazenk	Syunik	523	4236	39	188	6	8	90	40	2.10	0.07	0.09	0.45	0.03	0.04	0.48	0.21
Bazenk AR58.sK1b	Bazenk	Syunik	574	4328	36	201	5	7	90	39	2.25	0.06	0.08	0.43	0.03	0.03	0.44	0.19
Bazenk AR58.sK1c	Bazenk	Syunik	575	4320	37	196	7	8	89	39	2.21	0.08	0.09	0.44	0.04	0.04	0.45	0.20
Bazenk AR58.sK1e	Bazenk	Syunik	589	4297	33	194	6	8	92	39	2.10	0.07	0.08	0.42	0.03	0.04	0.48	0.20
Bazenk AR58.sK1f	Bazenk	Syunik	539	4264	37	194	5	9	93	43	2.07	0.06	0.09	0.46	0.03	0.04	0.48	0.22
Bazenk AR58.sK1g	Bazenk	Syunik	542	4214	38	192	6	8	89	39	2.16	0.07	0.09	0.44	0.03	0.04	0.46	0.20
Bazenk AR58.sK1h	Bazenk	Syunik	531	4240	36	195	5	6	89	37	2.20	0.06	0.07	0.42	0.03	0.03	0.46	0.19
Bazenk AR82.rB1a	Bazenk	Syunik	491	4188	38	190	7	10	98	37	1.93	0.08	0.10	0.38	0.04	0.05	0.52	0.20
Bazenk AR82.rB1b	Bazenk	Syunik	576	4199	41	194	8	6	90	37	2.16	0.09	0.06	0.42	0.04	0.03	0.46	0.19
Bazenk AR82.rB2a	Bazenk	Syunik	547	4249	40	190	6	8	87	37	2.19	0.07	0.09	0.43	0.03	0.04	0.46	0.20

Bazenk AR82.rB2b	Bazenk	Syunik	508	4284	39	193	8	11	91	37	2.13	0.09	0.12	0.41	0.04	0.05	0.47	0.19
Bazenk AR82.rB3a	Bazenk	Syunik	518	4226	36	191	6	8	90	37	2.13	0.07	0.09	0.42	0.03	0.04	0.47	0.20
Bazenk AR82.rB3b	Bazenk	Syunik	541	4233	35	195	7	9	89	36	2.20	0.08	0.10	0.41	0.04	0.04	0.46	0.19
Satanakar-1 AR56.sK1a	Satanakar-1	Syunik	541	4431	38	204	10	11	93	40	2.19	0.11	0.11	0.43	0.05	0.05	0.46	0.20
Satanakar-1 AR56.sK1b	Satanakar-1	Syunik	559	4519	34	202	10	10	94	40	2.15	0.11	0.10	0.43	0.05	0.05	0.47	0.20
Satanakar-1 AR80.rB1	Satanakar-1	Syunik	509	4426	40	193	10	8	90	35	2.15	0.12	0.09	0.39	0.05	0.04	0.46	0.18
Satanakar-1 Cobble1	Satanakar-1	Syunik	541	4566	35	193	10	11	89	37	2.17	0.12	0.12	0.42	0.05	0.05	0.46	0.19
Satanakar-1 Cobble2	Satanakar-1	Syunik	519	4592	42	198	10	11	91	39	2.16	0.11	0.13	0.42	0.05	0.06	0.46	0.20
Satanakar-1 Cobble3	Satanakar-1	Syunik	555	4468	39	199	11	8	90	37	2.23	0.13	0.09	0.42	0.06	0.04	0.45	0.19
Satanakar-1 Cobble4a	Satanakar-1	Syunik	546	4607	35	198	11	10	91	35	2.18	0.13	0.11	0.38	0.06	0.05	0.46	0.18
Satanakar-1 Cobble4b	Satanakar-1	Syunik	503	4556	39	197	10	11	91	36	2.17	0.12	0.12	0.40	0.05	0.05	0.46	0.18
Satanakar-1 Cobble4c	Satanakar-1	Syunik	509	4555	39	199	13	9	91	40	2.18	0.14	0.09	0.44	0.06	0.04	0.46	0.20
Satanakar-1 Cobble6	Satanakar-1	Syunik	531	4574	37	198	11	10	92	36	2.14	0.12	0.10	0.39	0.06	0.05	0.47	0.18
Satanakar-1 Cobble7	Satanakar-1	Syunik	528	4618	37	197	11	11	94	37	2.08	0.12	0.12	0.40	0.06	0.06	0.48	0.19
Satanakar-1 Cobble8	Satanakar-1	Syunik	515	4586	35	198	11	10	89	37	2.23	0.13	0.11	0.42	0.06	0.05	0.45	0.19
Satanakar-2 2017a	Satanakar-2	Syunik	614	4379	37	220	8	11	97	46	2.26	0.09	0.12	0.47	0.04	0.05	0.44	0.21
Satanakar-2 2017b	Satanakar-2	Syunik	620	4377	39	217	8	8	90	43	2.42	0.09	0.09	0.48	0.04	0.04	0.41	0.20
Satanakar-2 2017c	Satanakar-2	Syunik	617	4352	40	220	8	11	91	44	2.43	0.09	0.12	0.49	0.04	0.05	0.41	0.20
Satanakar-2 2017d	Satanakar-2	Syunik	602	4357	38	219	7	8	91	40	2.42	0.08	0.09	0.44	0.03	0.04	0.41	0.18
Satanakar-3 2017a	Satanakar-3	Syunik	594	4411	36	213	8	8	93	43	2.28	0.09	0.08	0.46	0.04	0.04	0.44	0.20
Satanakar-3 2017b	Satanakar-3	Syunik	600	4402	39	212	9	11	92	43	2.30	0.10	0.11	0.46	0.04	0.05	0.44	0.20
Satanakar-3 2017c	Satanakar-3	Syunik	566	4421	35	210	9	7	88	39	2.40	0.11	0.08	0.44	0.05	0.03	0.42	0.18
Satanakar-3 2017d	Satanakar-3	Syunik	576	4421	38	215	9	10	95	39	2.26	0.10	0.10	0.41	0.04	0.04	0.44	0.18
Sevkar AR33.ipS1a	Sevkar	Syunik	450	4807	34	173	18	9	96	36	1.80	0.18	0.09	0.37	0.10	0.05	0.56	0.21
Sevkar AR33.ipS1b	Sevkar	Syunik	481	4813	35	177	20	11	107	33	1.66	0.18	0.10	0.31	0.11	0.06	0.60	0.19
Sevkar AR35.ipS1	Sevkar	Syunik	493	4899	36	179	20	9	100	33	1.79	0.20	0.09	0.33	0.11	0.05	0.56	0.19
Sevkar AR57.sK1a	Sevkar	Syunik	508	4878	35	180	17	9	100	35	1.80	0.17	0.09	0.35	0.09	0.05	0.56	0.19
Sevkar AR57.sK1b	Sevkar	Syunik	469	4847	33	178	20	11	100	33	1.78	0.20	0.11	0.33	0.11	0.06	0.56	0.19
Sevkar AR81.rB1a	Sevkar	Syunik	473	4817	34	179	16	10	96	35	1.86	0.16	0.10	0.36	0.09	0.05	0.54	0.19
Sevkar AR81.rB1b	Sevkar	Syunik	478	4619	35	178	16	10	94	36	1.89	0.16	0.10	0.38	0.09	0.05	0.53	0.20
Sevkar AR81.rB2	Sevkar	Syunik	494	4836	33	186	17	10	97	35	1.91	0.17	0.10	0.36	0.09	0.05	0.52	0.19
Sevkar AR81.rB3	Sevkar	Syunik	450	4779	41	179	19	10	96	31	1.86	0.19	0.10	0.32	0.10	0.05	0.54	0.17
Sevkar GPS 539-1a	Sevkar	Syunik	450	5009	33	169	21	9	103	33	1.65	0.20	0.08	0.32	0.12	0.05	0.61	0.20
Sevkar GPS 539-2a	Sevkar	Syunik	479	4929	38	175	19	10	102	33	1.72	0.18	0.09	0.33	0.11	0.05	0.58	0.19
Sevkar GPS 539-2b	Sevkar	Syunik	465	4902	34	173	21	11	99	33	1.75	0.21	0.11	0.34	0.12	0.06	0.57	0.19
Sevkar GPS 542b	Sevkar	Syunik	494	4966	37	179	20	10	98	31	1.83	0.20	0.10	0.31	0.11	0.05	0.55	0.17
Tsaghk-1 Kamakar AR64.sK1a	Tsaghk-1 Kamakar	Tsaghkunyats	428	7425	35	86	238	10	149	17	0.58	1.60	0.06	0.11	2.77	0.11	1.74	0.20
Tsaghk-1 Kamakar AR64.sK1b	Tsaghk-1 Kamakar	Tsaghkunyats	425	7398	35	90	237	7	149	15	0.60	1.59	0.05	0.10	2.64	0.08	1.66	0.17
Tsaghk-1 Kamakar AR64.sK1c	Tsaghk-1 Kamakar	Tsaghkunyats	439	7526	35	89	239	6	150	18	0.59	1.59	0.04	0.12	2.69	0.07	1.69	0.21
Tsaghk-1 Kamakar AR64.sK1d	Tsaghk-1 Kamakar	Tsaghkunyats	423	7454	36	90	241	7	151	15	0.59	1.60	0.04	0.10	2.69	0.08	1.68	0.17
Tsaghk-1 Kamakar AR64.sK1e	Tsaghk-1 Kamakar	Tsaghkunyats	409	7163	33	86	226	6	141	14	0.61	1.61	0.04	0.10	2.63	0.07	1.64	0.16
Tsaghk-1 Kamakar AR71.rB1a	Tsaghk-1 Kamakar	Tsaghkunyats	432	7531	34	91	241	11	152	17	0.60	1.59	0.07	0.11	2.66	0.12	1.68	0.19
Tsaghk-1 Kamakar AR71.rB1b	Tsaghk-1 Kamakar	Tsaghkunyats	439	7288	37	88	231	9	144	17	0.61	1.60	0.06	0.12	2.63	0.10	1.65	0.19
Tsaghk-2 Ttavakar AR43.kM1a	Tsaghk-2 Ttavakar	Tsaghkunyats	430	6204	31	94	181	10	105	20	0.89	1.73	0.09	0.19	1.93	0.10	1.12	0.21
Tsaghk-2 Ttavakar AR43.kM1b	Tsaghk-2 Ttavakar	Tsaghkunyats	455	6242	32	96	183	8	106	22	0.91	1.73	0.07	0.21	1.90	0.08	1.09	0.23
Tsaghk-2 Ttavakar AR43.kM1c	Tsaghk-2 Ttavakar	Tsaghkunyats	455	6218	34	93	180	10	105	22	0.88	1.72	0.09	0.21	1.94	0.10	1.13	0.24
Tsaghk-2 Ttavakar AR43.kM1d	Tsaghk-2 Ttavakar	Tsaghkunyats	489	6207	27	95	181	8	106	22	0.90	1.71	0.07	0.21	1.91	0.08	1.12	0.24
Tsaghk-2 Ttavakar AR43.kM1e	Tsaghk-2 Ttavakar	Tsaghkunyats	469	6187	34	96	180	10	107	21	0.90	1.69	0.09	0.20	1.88	0.10	1.12	0.22
Tsaghk-2 Ttavakar AR60.sK1a	Tsaghk-2 Ttavakar	Tsaghkunyats	471	6559	27	94	181	11	108	22	0.87	1.68	0.10	0.21	1.93	0.11	1.15	0.24
Tsaghk-2 Ttavakar AR60.sK1b	Tsaghk-2 Ttavakar	Tsaghkunyats	449	6266	30	95	181	7	109	18	0.86	1.65	0.06	0.17	1.91	0.07	1.16	0.19
Tsaghk-2 Ttavakar AR60.sK1c	Tsaghk-2 Ttavakar	Tsaghkunyats	464	6312	30	94	182	9	104	22	0.90	1.75	0.08	0.22	1.94	0.09	1.11	0.24
Tsaghk-2 Ttavakar AR60.sK1d	Tsaghk-2 Ttavakar	Tsaghkunyats	428	6298	31	96	183	11	106	22	0.90	1.73	0.11	0.21	1.91	0.12	1.11	0.23
Tsaghk-2 Ttavakar AR60.sK1e	Tsaghk-2 Ttavakar	Tsaghkunyats	469	6299	38	93	180	9	102	21	0.91	1.77	0.08	0.21	1.94	0.09	1.10	0.23
Tsaghk-2 Ttavakar AR60.sK1f	Tsaghk-2 Ttavakar	Tsaghkunyats	450	6420	28	93	181	9	106	21	0.88	1.71	0.08	0.20	1.95	0.09	1.14	0.23
Tsaghk-2 Ttavakar AR70.rB1a	Tsaghk-2 Ttavakar	Tsaghkunyats	448	6261	34	97	179	7	109	20	0.89	1.63	0.06	0.18	1.84	0.07	1.12	0.20
Tsaghk-2 Ttavakar AR70.rB1b	Tsaghk-2 Ttavakar	Tsaghkunyats	473	6240	34	95	178	10	108	20	0.88	1.65	0.09	0.18	1.88	0.10	1.14	0.21
Tsaghk-2 Ttavakar AR70.rB1c	Tsaghk-2 Ttavakar	Tsaghkunyats	470	6360	32	96	181	11	108	24	0.89	1.68	0.11	0.22	1.89	0.12	1.13	0.25
Tsaghk-2 Ttavakar AR70.rB2a	Tsaghk-2 Ttavakar	Tsaghkunyats	449	6190	30	94	175	10	104	22	0.90	1.69	0.09	0.22	1.87	0.10	1.11	0.24
Tsaghk-2 Ttavakar AR70.rB2b	Tsaghk-2 Ttavakar	Tsaghkunyats	469	6311	31	93	178	10	102	20	0.91	1.75	0.09	0.19	1.92	0.10	1.10	0.21
Tsaghk-3 Damlik AR69.rB1	Tsaghk-3 Damlik	Tsaghkunyats	430	5758	34	112	155	11	95	21	1.18	1.63	0.11	0.22	1.38	0.09	0.85	0.19
Tsaghk-3 Damlik AR69.rB2	Tsaghk-3 Damlik	Tsaghkunyats	419	5765	35	112	157	10	97	18	1.15	1.61	0.10	0.19	1.40	0.09	0.87	0.16
Tsaghk-3 Damlik AR69.rB3a	Tsaghk-3 Damlik	Tsaghkunyats	451	5749	34	114	155	11	98	20	1.16	1.58	0.11	0.20	1.36	0.09	0.86	0.17
Tsaghk-3 Damlik AR69.rB3b	Tsaghk-3 Damlik	Tsaghkunyats	434	5891	39	114	156	11	99	18	1.15	1.57	0.12	0.18	1.37	0.10	0.87	0.16
Tsaghk-3 Damlik AR69.rB3c	Tsaghk-3 Damlik	Tsaghkunyats	433	5697	37	109	152	12	97	17	1.12	1.56	0.13	0.17	1.39	0.11	0.89	0.15

Meas #	Bag information / info field	DA Source ID	Trench	Horizon	Subhoriz/Note	Unit/Note	Dig Date	pXRF #	pXRF Date	Mn	Fe	Zn	Rb	Sr	Y	Zr	Nb	Rb/Zr	Sr/Zr	Y/Zr	Nb/Zr	Sr/Rb	Y/Rb	Zr/Rb	Nb/Rb
1	Point Tr-2 H-2 Sp-2 16-6-22	Gegham 1	2	2	2		16-Jun	80	6/28/23	611	3552	42	198	20	25	73	48	2.72	0.27	0.34	0.67	0.10	0.12	0.37	0.24
3	Pit 2 Hor1 S.1 larger 1 of 2	Gegham 1	2	1	1		?	82	6/28/23	676	4008	35	209	19	25	73	54	2.88	0.26	0.34	0.74	0.09	0.12	0.35	0.26
5	Pit 2 Hor1 S.1 smaller 2 of 2	Kelbadjar	2	1	1		?	84	6/28/23	501	4937	42	157	15	11	106	32	1.48	0.14	0.11	0.30	0.09	0.07	0.67	0.20
8	Tr2 Hor1 Sp1 13-6-22 clear blade 1 of 3	Gegham 1	2	1	1		13-Jun	87	6/28/23	680	4004	34	214	11	26	73	55	2.95	0.16	0.35	0.76	0.05	0.12	0.34	0.26
10	Tr2 Hor1 Sp1 13-6-22 flake frag 2 of 3	Gutansar	2	1	1		13-Jun	89	6/28/23	692	8438	46	146	127	24	176	39	0.83	0.72	0.13	0.22	0.87	0.16	1.20	0.27
12	Tr2 Hor1 Sp1 13-6-22 retouch point tip 3 of 3	Gegham 1	2	1	1		13-Jun	91	6/28/23	741	4064	36	218	15	25	74	54	2.93	0.19	0.33	0.72	0.07	0.11	0.34	0.25
15	Hor1 Tr1 Sp2 13.06.22 larger 1 of 2	Gegham 1	1	1	2		13-Jun	94	6/28/23	703	3773	34	206	13	26	70	55	2.96	0.18	0.37	0.79	0.06	0.12	0.34	0.27
17	Hor1 Tr1 Sp2 13.06.22 smaller 2 of 2	Gegham 1	1	1	2		13-Jun	96	6/28/23	723	3975	34	214	14	25	71	54	3.03	0.19	0.35	0.76	0.06	0.12	0.33	0.25
19	Tr2 Hor5 south part 22-06-22 core? 1 of 2	Arteni	2	5	South		?	98	6/28/23	586	4446	38	131	32	26	84	31	1.57	0.38	0.30	0.36	0.24	0.19	0.64	0.23
21	Tr2 Hor5 south part 22-06-22 broken flake 2 of 2	Gegham 1	2	5	South		?	100	6/28/23	693	3790	31	208	13	25	73	51	2.87	0.17	0.34	0.70	0.06	0.12	0.35	0.25
23	Pit1 TopSoil 10-06-22 retouched blade 1 of 2	Gegham 1	1	TS	-		10-Jun	102	6/28/23	695	3717	34	209	13	26	68	54	3.08	0.18	0.38	0.79	0.06	0.12	0.32	0.26
25	Pit1 TopSoil 10-06-22 unretouched blade 2 of 2	Gegham 1	1	TS	-		10-Jun	104	6/28/23	700	4126	36	218	13	24	73	55	3.00	0.17	0.33	0.76	0.06	0.11	0.33	0.25
27	Trench2 TopSoil 13-Jun-22 broken flake	Kelbadjar	2	TS	-		13-Jun	109	6/28/23	503	5249	40	163	16	10	108	35	1.50	0.14	0.09	0.32	0.10	0.06	0.67	0.21
29	Trench2 Hor4 Spit2 Ash Layer retouched point tip	Gegham 1	2	4	2		?	111	6/28/23	712	4208	33	217	14	26	74	57	2.91	0.18	0.34	0.76	0.06	0.12	0.34	0.26
31	Trench1 Hor1 Sp2 12-Jun-22 retouched tool	Gegham 1	1	1	2		12-Jun	113	6/28/23	715	4004	35	215	13	27	74	58	2.89	0.17	0.36	0.78	0.06	0.12	0.35	0.27
33	Pit1 TopSoil 10-Jun-22 core?	Gegham 1	1	TS	-		10-Jun	115	6/28/23	661	3859	36	209	13	25	72	54	2.92	0.17	0.34	0.75	0.06	0.12	0.34	0.26
35	Pit2 Hor1 S.1 12.06.22 core frag?	Kelbadjar	2	1	1		12-Jun	117	6/28/23	542	5363	42	164	17	11	110	35	1.48	0.15	0.10	0.31	0.10	0.06	0.67	0.21
37	Pit 2 Hor1 S.1 12.06.22 flake retouched?	Arteni	2	1	1		12-Jun	119	6/28/23	658	4097	45	137	20	29	81	33	1.69	0.24	0.36	0.41	0.14	0.21	0.59	0.24
39	Trench1 Hor1 Sp.2 12-Jun-22 broken flake	Syunik	1	1	2		12-Jun	121	6/28/23	474	4828	35	172	21	11	103	33	1.68	0.20	0.11	0.32	0.12	0.07	0.60	0.19
41	Tr-1 Hor-2 Sp 1 core fragment?	Gegham 1	1	2	1		?	123	6/28/23	724	4001	35	210	13	25	73	55	2.86	0.17	0.34	0.75	0.06	0.12	0.35	0.26
43	Tr-2 Hor-4 Sp-1 bladelet	Gegham 1	2	4	1		?	125	6/28/23	799	4286	37	224	16	23	73	54	3.08	0.21	0.31	0.74	0.07	0.10	0.32	0.24
46	smalls Trench2 Hor.1 Sp.1 13-Jun-22 one each	Kelbadjar	2	1	1		13-Jun	1	6/29/23	501	5267	35	159	18	11	108	36	1.48	0.16	0.11	0.34	0.11	0.07	0.68	0.23
47	smalls Trench2 Hor.1 Sp.1 13-Jun-22 one each	Kelbadjar	2	1	1		13-Jun	2	6/29/23	493	5123	35	155	17	11	107	33	1.45	0.15	0.11	0.31	0.11	0.07	0.69	0.22
48	smalls Trench2 Hor.1 Sp.1 13-Jun-22 one each	Kelbadjar	2	1	1		13-Jun	3	6/29/23	515	5473	38	163	16	11	113	35	1.44	0.14	0.09	0.31	0.10	0.06	0.70	0.21
49	smalls Trench2 Hor.1 Sp.1 13-Jun-22 one each	Gegham 1	2	1	1		13-Jun	4	6/29/23	622	3602	27	199	13	25	66	51	3.03	0.19	0.37	0.78	0.06	0.12	0.33	0.26
50	smalls Trench2 Hor.1 Sp.1 13-Jun-22 one each	Gegham 1	2	1	1		13-Jun	5	6/29/23	680	3900	38	209	14	24	70	53	3.00	0.19	0.34	0.75	0.06	0.11	0.33	0.25
51	smalls Trench2 Hor.1 Sp.1 13-Jun-22 one each	Gegham 1	2	1	1		13-Jun	6	6/29/23	736	4412	35	220	15	27	75	55	2.92	0.19	0.35	0.73	0.07	0.12	0.34	0.25
52	smalls Trench2 Hor.1 Sp.1 13-Jun-22 one each	Gegham 1	2	1	1		13-Jun	7	6/29/23	685	4195	36	211	14	25	72	54	2.95	0.19	0.34	0.75	0.06	0.12	0.34	0.26
53	smalls Trench2 Hor.1 Sp.1 13-Jun-22 one each	Gegham 1	2	1	1		13-Jun	8	6/29/23	678	3930	32	212	11	24	76	57	2.78	0.15	0.31	0.74	0.05	0.11	0.36	0.27
54	smalls Trench2 Hor.1 Sp.1 13-Jun-22 one each	Syunik	2	1	1		13-Jun	9	6/29/23	505	5230	38	177	23	11	108	33	1.63	0.21	0.10	0.31	0.13	0.06	0.61	0.19
55	smalls Trench2 Hor.1 Sp.1 13-Jun-22 one each	Kelbadjar	2	1	1		13-Jun	10	6/29/23	562	5473	44	164	16	11	121	39	1.36	0.13	0.09	0.32	0.09	0.07	0.73	0.24
56	smalls Trench2 Hor.1 Sp.1 13-Jun-22 one each	Kelbadjar	2	1	1		13-Jun	11	6/29/23	519	5314	41	168	18	10	106	32	1.59	0.17	0.09	0.30	0.10	0.06	0.63	0.19
57	smalls Trench2 Hor.1 Sp.1 13-Jun-22 one each	Gegham 1	2	1	1		13-Jun	12	6/29/23	675	3960	36	212	15	25	73	51	2.89	0.20	0.34	0.70	0.07	0.12	0.35	0.24
58	smalls Trench2 Hor.1 Sp.1 13-Jun-22 one each	Gegham 1	2	1	1		13-Jun	14	6/29/23	702	3997	33	216	13	25	73	57	2.94	0.17	0.34	0.77	0.06	0.11	0.34	0.26
59	smalls Trench2 Hor.1 Sp.1 13-Jun-22 one each	Kelbadjar	2	1	1		13-Jun	15	6/29/23	542	5417	44	165	16	11	111	33	1.49	0.14	0.09	0.30	0.09	0.06	0.67	0.20
60	smalls Trench2 Hor.1 Sp.1 13-Jun-22 one each	Kelbadjar	2	1	1		13-Jun	16	6/29/23	575	5889	47	168	23	11	116	33	1.45	0.19	0.10	0.29	0.13	0.07	0.69	0.20
61	smalls Trench2 Hor.1 Sp.1 13-Jun-22 one each	Kelbadjar	2	1	1		13-Jun	17	6/29/23	557	5664	44	168	18	12	125	33	1.35	0.14	0.10	0.27	0.10	0.07	0.74	0.20
62	smalls Trench2 Hor.1 Sp.1 13-Jun-22 one each	Gegham 1	2	1	1		13-Jun	18	6/29/23	769	4297	36	222	14	26	73	53	3.02	0.18	0.35	0.71	0.06	0.12	0.33	0.24
63	smalls Trench2 Hor.1 Sp.1 13-Jun-22 one each	Arteni	2	1	1		13-Jun	19	6/29/23	575	5095	42	125	47	24	96	26	1.30	0.48	0.25	0.27	0.37	0.19	0.77	0.21
64	smalls Trench2 Hor.1 Sp.1 13-Jun-22 one each	Kelbadjar	2	1	1		13-Jun	20	6/29/23	575	5919	46	176	16	11	121	40	1.46	0.13	0.09	0.33	0.09	0.07	0.69	0.23
65	smalls Trench2 Hor.1 Sp.1 13-Jun-22 one each	Gegham 1	2	1	1		13-Jun	21	6/29/23	758	4241	37	228	14	26	75	54	3.02	0.18	0.34	0.71	0.06	0.11	0.33	0.24
66	smalls Trench2 Hor.1 Sp.1 13-Jun-22 one each	Gegham 1	2	1	1		13-Jun	22	6/29/23	752	4039	38	223	13	26	73	54	3.07	0.17	0.35	0.74	0.06	0.11	0.33	0.24
67	smalls Trench2 Hor.1 Sp.1 13-Jun-22 one each	Syunik	2	1	1		13-Jun	23	6/29/23	491	5301	35	181	22	11	105	32	1.73	0.21	0.11	0.30	0.12	0.06	0.58	0.18
68	smalls Trench2 Hor.1 Sp.1 13-Jun-22 one each	Kelbadjar	2	1	1		13-Jun	24	6/29/23	570	5701	44	174	17	10	110	36	1.58	0.15	0.09	0.33	0.09	0.06	0.63	0.21
69	smalls Trench2 Hor.1 Sp.1 13-Jun-22 one each	Kelbadjar	2	1	1		13-Jun	25	6/29/23	658	6641	51	187	19	11	123	36	1.52	0.15	0.09	0.29	0.10	0.06	0.66	0.19
70	smalls Trench2 Hor.1 Sp.1 13-Jun-22 one each	Gegham 1	2	1	1		13-Jun	26	6/29/23	681	3878	35	207	14	24	72	51	2.89	0.19	0.33	0.71	0.07	0.11	0.35	0.25
71	smalls Trench2 Hor.1 Sp.1 13-Jun-22 one each	Kelbadjar	2	1	1		13-Jun	27	6/29/23	652	6425	51	184	17	9	110	37	1.67	0.15	0.08	0.34	0.09	0.05	0.60	0.20
72	smalls Trench2 Hor.1 Sp.1 13-Jun-22 one each	Gegham 1	2	1	1		13-Jun	28	6/29/23	791	4487	42	233	14	26	76	54	3.06	0.18	0.34	0.71	0.06	0.11	0.33	0.23
73	smalls Trench2 Hor.1 Sp.1 13-Jun-22 one each	Gegham 1	2	1	1		13-Jun	29	6/29/23	921	5086	46	260	16	25	76	57	3.40	0.20	0.32	0.74	0.06	0.09	0.29	0.22
74	smalls Trench2 Hor.1 Sp.1 13-Jun-22 one each	Kelbadjar	2	1	1		13-Jun	30	6/29/23	600	6144	49	179	19	11	111	39	1.61	0.17	0.10	0.35	0.10	0.06	0.62	0.22

75	smalls Pit2 Hor.1 S.1 12.06.22 one each	Gegham 1	2	1	1	12-Jun	32	6/29/23	715	4039	32	212	13	25	72	58	2.96	0.17	0.34	0.81	0.06	0.12	0.34	0.27
76	smalls Pit2 Hor.1 S.1 12.06.22 one each	Syunik	2	1	1	12-Jun	33	6/29/23	473	5201	35	176	23	10	105	36	1.68	0.22	0.09	0.34	0.13	0.05	0.59	0.20
77	smalls Pit2 Hor.1 S.1 12.06.22 one each	Gegham 1	2	1	1	12-Jun	34	6/29/23	676	3859	33	217	14	26	71	54	3.07	0.19	0.36	0.76	0.06	0.12	0.33	0.25
78	smalls Pit2 Hor.1 S.1 12.06.22 one each	Gegham 1	2	1	1	12-Jun	35	6/29/23	696	3891	30	203	14	24	69	53	2.96	0.20	0.34	0.76	0.07	0.12	0.34	0.26
79	smalls Pit2 Hor.1 S.1 12.06.22 one each	Kelbadjar	2	1	1	12-Jun	36	6/29/23	504	5255	41	161	17	12	108	33	1.48	0.15	0.11	0.31	0.10	0.08	0.68	0.21
80	smalls Pit2 Hor.1 S.1 12.06.22 one each	Syunik	2	1	1	12-Jun	37	6/29/23	495	4972	36	173	21	10	102	36	1.70	0.20	0.09	0.35	0.12	0.06	0.59	0.21
81	smalls Pit2 Hor.1 S.1 12.06.22 one each	Gegham 1	2	1	1	12-Jun	38	6/29/23	616	4132	34	184	18	21	78	43	2.35	0.22	0.27	0.55	0.10	0.11	0.43	0.23
82	smalls Pit2 Hor.1 S.1 12.06.22 one each	Gegham 1	2	1	1	12-Jun	39	6/29/23	804	4215	34	225	14	24	74	57	3.02	0.18	0.32	0.76	0.06	0.11	0.33	0.25
83	smalls Pit2 Hor.1 S.1 12.06.22 one each	Gegham 1	2	1	1	12-Jun	40	6/29/23	681	3784	34	209	13	22	71	54	2.96	0.18	0.31	0.76	0.06	0.10	0.34	0.26
84	smalls Pit2 Hor.1 S.1 12.06.22 one each	Syunik	2	1	1	12-Jun	41	6/29/23	513	4985	35	181	20	10	101	35	1.79	0.19	0.09	0.34	0.11	0.05	0.56	0.19
85	smalls Pit2 Hor.1 S.1 12.06.22 one each	Gegham 1	2	1	1	12-Jun	42	6/29/23	594	3587	34	195	13	23	69	51	2.83	0.18	0.33	0.74	0.06	0.12	0.35	0.26
86	smalls Pit2 Hor.1 S.1 12.06.22 one each	Gegham 1	2	1	1	12-Jun	43	6/29/23	706	4110	33	214	11	25	72	53	2.99	0.16	0.34	0.73	0.05	0.12	0.33	0.25
87	smalls Pit2 Hor.1 S.1 12.06.22 one each	Gegham 1	2	1	1	12-Jun	44	6/29/23	683	4211	33	211	15	24	73	51	2.87	0.20	0.32	0.70	0.07	0.11	0.35	0.24
88	smalls Pit2 Hor.1 S.1 12.06.22 one each	Gegham 1	2	1	1	12-Jun	45	6/29/23	732	4290	36	221	14	25	74	61	2.97	0.18	0.33	0.82	0.06	0.11	0.34	0.28
89	smalls Pit2 Hor.1 S.1 12.06.22 one each	Gegham 1	2	1	1	12-Jun	46	6/29/23	742	4013	35	215	13	25	71	55	3.04	0.18	0.35	0.78	0.06	0.11	0.33	0.26
90	smalls Pit2 Hor.1 S.1 12.06.22 one each	Gegham 1	2	1	1	12-Jun	47	6/29/23	655	3892	33	212	15	26	75	54	2.81	0.19	0.34	0.71	0.07	0.12	0.36	0.25
91	smalls Pit2 Hor.1 S.1 12.06.22 one each	Syunik	2	1	1	12-Jun	48	6/29/23	585	4772	42	226	10	10	94	43	2.39	0.11	0.10	0.45	0.05	0.04	0.42	0.19
92	smalls Pit2 Hor.1 S.1 12.06.22 one each	Gegham 1	2	1	1	12-Jun	49	6/29/23	687	3922	33	210	14	26	71	51	2.97	0.19	0.36	0.72	0.06	0.12	0.34	0.24
93	smalls Pit2 Hor.1 S.1 12.06.22 one each	Gegham 1	2	1	1	12-Jun	50	6/29/23	713	3941	32	213	13	26	73	54	2.90	0.17	0.35	0.73	0.06	0.12	0.34	0.25
94	smalls Pit2 Hor.1 S.1 12.06.22 one each	Gutansar	2	1	1	12-Jun	51	6/29/23	697	8893	44	153	135	23	177	39	0.87	0.76	0.13	0.22	0.88	0.15	1.16	0.25
95	smalls Pit2 Hor.1 S.1 12.06.22 one each	Khorapor	2	1	1	12-Jun	52	6/29/23	752	3922	37	289	11	24	70	53	4.14	0.16	0.34	0.75	0.04	0.08	0.24	0.18
96	smalls Pit2 Hor.1 S.1 12.06.22 one each	Gegham 1	2	1	1	12-Jun	53	6/29/23	777	4432	39	234	14	26	74	57	3.15	0.18	0.34	0.76	0.06	0.11	0.32	0.24
97	smalls Pit2 Hor.1 S.1 12.06.22 one each	Gegham 1	2	1	1	12-Jun	54	6/29/23	723	4401	37	225	17	26	72	54	3.14	0.23	0.36	0.75	0.07	0.11	0.32	0.24
98	smalls Pit2 Hor.1 S.1 12.06.22 one each	Gegham 1	2	1	1	12-Jun	55	6/29/23	728	4195	34	219	14	25	73	54	3.02	0.19	0.34	0.74	0.06	0.11	0.33	0.25
99	smalls Pit2 Hor.1 S.1 12.06.22 one each	Kelbadjar	2	1	1	12-Jun	56	6/29/23	479	5105	40	157	15	10	107	35	1.47	0.14	0.09	0.33	0.09	0.06	0.68	0.22
100	smalls Pit2 Hor.1 S.1 12.06.22 one each	Gegham 1	2	1	1	12-Jun	57	6/29/23	705	3870	33	216	14	27	72	53	3.02	0.19	0.38	0.73	0.06	0.13	0.33	0.24
101	smalls Pit2 Hor.1 S.1 12.06.22 one each	Syunik	2	1	1	12-Jun	58	6/29/23	565	4924	45	203	17	11	96	43	2.11	0.17	0.12	0.45	0.08	0.06	0.47	0.21
102	smalls Pit2 Hor.1 S.1 12.06.22 one each	Syunik	2	1	1	12-Jun	59	6/29/23	446	4962	52	179	26	10	105	36	1.71	0.24	0.09	0.34	0.14	0.05	0.58	0.20
103	smalls Pit2 Hor.1 S.1 12.06.22 one each	Syunik	2	1	1	12-Jun	60	6/29/23	520	5431	39	191	21	10	105	40	1.82	0.20	0.09	0.38	0.11	0.05	0.55	0.21
104	smalls Pit2 Hor.1 S.1 12.06.22 one each	Gegham 1	2	1	1	12-Jun	61	6/29/23	691	3892	35	214	13	26	73	58	2.95	0.17	0.35	0.80	0.06	0.12	0.34	0.27
105	smalls Pit2 Hor.1 S.1 12.06.22 one each	Gegham 1	2	1	1	12-Jun	62	6/29/23	1067	5528	50	263	13	23	73	53	3.62	0.17	0.31	0.72	0.05	0.09	0.28	0.20
106	smalls bag3 Pit2 Hor.1 S.1 12.06.22 one each	Gegham 1	2	1	1	12-Jun	64	6/29/23	802	4356	37	236	13	26	77	58	3.06	0.16	0.33	0.75	0.05	0.11	0.33	0.25
107	smalls bag3 Pit2 Hor.1 S.1 12.06.22 one each	Gegham 1	2	1	1	12-Jun	65	6/29/23	819	4444	39	233	14	27	76	55	3.05	0.18	0.36	0.72	0.06	0.12	0.33	0.24
108	smalls bag3 Pit2 Hor.1 S.1 12.06.22 one each	Gegham 1	2	1	1	12-Jun	66	6/29/23	794	4355	43	234	14	25	75	57	3.11	0.18	0.33	0.75	0.06	0.11	0.32	0.24
109	smalls bag4 Pit2 Hor.1 S.1 12.06.22 one each	Kelbadjar	2	1	1	12-Jun	68	6/29/23	566	5806	43	170	17	10	111	36	1.53	0.15	0.09	0.32	0.10	0.06	0.65	0.21
110	smalls bag4 Pit2 Hor.1 S.1 12.06.22 one each	Kelbadjar	2	1	1	12-Jun	69	6/29/23	479	4999	39	156	16	11	108	35	1.44	0.14	0.10	0.32	0.10	0.07	0.70	0.22
111	smalls bag4 Pit2 Hor.1 S.1 12.06.22 one each	Kelbadjar	2	1	1	12-Jun	70	6/29/23	524	5376	40	165	18	11	110	36	1.50	0.16	0.10	0.33	0.11	0.07	0.67	0.22
112	smalls bag4 Pit2 Hor.1 S.1 12.06.22 one each	Gegham 1	2	1	1	12-Jun	71	6/29/23	695	4033	35	215	13	25	76	55	2.82	0.16	0.32	0.72	0.06	0.11	0.35	0.26
113	smalls bag4 Pit2 Hor.1 S.1 12.06.22 one each	Gegham 1	2	1	1	12-Jun	72	6/29/23	707	3989	35	218	13	26	74	57	2.93	0.17	0.34	0.76	0.06	0.12	0.34	0.26
114	smalls bag4 Pit2 Hor.1 S.1 12.06.22 one each	Gegham 1	2	1	1	12-Jun	73	6/29/23	631	3751	30	197	15	26	73	50	2.67	0.20	0.35	0.68	0.07	0.13	0.37	0.25
115	smalls bag4 Pit2 Hor.1 S.1 12.06.22 one each	Kelbadjar	2	1	1	12-Jun	74	6/29/23	523	5332	40	161	16	11	112	37	1.43	0.14	0.09	0.33	0.10	0.07	0.70	0.23
116	smalls bag4 Pit2 Hor.1 S.1 12.06.22 one each	Gegham 1	2	1	1	12-Jun	75	6/29/23	718	4002	34	214	14	25	73	54	2.95	0.19	0.34	0.74	0.06	0.12	0.34	0.25
117	smalls bag4 Pit2 Hor.1 S.1 12.06.22 one each	Gegham 1	2	1	1	12-Jun	76	6/29/23	687	3779	35	207	13	26	73	55	2.86	0.17	0.35	0.76	0.06	0.12	0.35	0.27
118	smalls bag4 Pit2 Hor.1 S.1 12.06.22 one each	Gegham 1	2	1	1	12-Jun	77	6/29/23	677	3768	35	207	13	24	71	54	2.93	0.18	0.34	0.76	0.06	0.11	0.34	0.26
119	smalls bag4 Pit2 Hor.1 S.1 12.06.22 one each	Kelbadjar	2	1	1	12-Jun	78	6/29/23	514	5431	41	162	18	12	109	36	1.48	0.16	0.11	0.33	0.11	0.08	0.68	0.22
120	smalls bag4 Pit2 Hor.1 S.1 12.06.22 one each	Gegham 1	2	1	1	12-Jun	79	6/29/23	736	4157	35	224	14	27	73	53	3.08	0.19	0.37	0.72	0.06	0.12	0.32	0.23
121	smalls bag4 Pit2 Hor.1 S.1 12.06.22 one each	Kelbadjar	2	1	1	12-Jun	80	6/29/23	528	5662	46	172	18	11	108	35	1.59	0.16	0.10	0.32	0.10	0.06	0.63	0.20
122	smalls bag4 Pit2 Hor.1 S.1 12.06.22 one each	Gegham 1	2	1	1	12-Jun	81	6/29/23	705	4283	40	211	19	26	74	54	2.84	0.25	0.34	0.72	0.09	0.12	0.35	0.26
123	smalls bag4 Pit2 Hor.1 S.1 12.06.22 one each	Gegham 1	2	1	1	12-Jun	82	6/29/23	696	4077	35	216	13	25	73	51	2.98	0.17	0.34	0.70	0.06	0.11	0.34	0.24
124	smalls bag4 Pit2 Hor.1 S.1 12.06.22 one each	Gegham 1	2	1	1	12-Jun	83	6/29/23	688	3952	35	210	15	27	75	54	2.79	0.19	0.36	0.71	0.07	0.13	0.36	0.26
125	smalls bag4 Pit2 Hor.1 S.1 12.06.22 one each	Kelbadjar	2	1	1	12-Jun	84	6/29/23	480	5153	40	163	16	9	109	35	1.49	0.14	0.08	0.32	0.10	0.05	0.67	0.21

126	smalls bag4 Pit2 Hor.1 S.1 12.06.22 one each	Gegham 1	2	1	1	12-Jun	85	6/29/23	748	4115	34	225	14	26	75	54	2.98	0.18	0.34	0.71	0.06	0.11	0.34	0.24
127	smalls bag4 Pit2 Hor.1 S.1 12.06.22 one each	Gegham 1	2	1	1	12-Jun	86	6/29/23	738	4592	36	218	19	26	78	47	2.79	0.24	0.33	0.60	0.08	0.12	0.36	0.22
128	smalls bag4 Pit2 Hor.1 S.1 12.06.22 one each	Gegham 1	2	1	1	12-Jun	87	6/29/23	732	4084	38	221	14	27	77	55	2.86	0.17	0.36	0.71	0.06	0.12	0.35	0.25
129	smalls bag4 Pit2 Hor.1 S.1 12.06.22 one each	Kelbadjar	2	1	1	12-Jun	88	6/29/23	523	5460	41	164	17	11	119	39	1.38	0.14	0.10	0.33	0.10	0.07	0.72	0.24
130	smalls bag4 Pit2 Hor.1 S.1 12.06.22 one each	Gegham 1	2	1	1	12-Jun	89	6/29/23	739	4109	37	221	11	24	75	57	2.93	0.15	0.31	0.75	0.05	0.11	0.34	0.26
131	smalls bag4 Pit2 Hor.1 S.1 12.06.22 one each	Gegham 1	2	1	1	12-Jun	90	6/29/23	683	3809	33	211	13	24	77	55	2.73	0.16	0.31	0.71	0.06	0.11	0.37	0.26
132	smalls bag4 Pit2 Hor.1 S.1 12.06.22 one each	Kelbadjar	2	1	1	12-Jun	91	6/29/23	529	5484	43	169	16	11	112	33	1.51	0.14	0.09	0.30	0.09	0.06	0.66	0.20
133	smalls bag4 Pit2 Hor.1 S.1 12.06.22 one each	Gegham 1	2	1	1	12-Jun	92	6/29/23	751	4194	38	229	14	27	73	53	3.15	0.19	0.38	0.72	0.06	0.12	0.32	0.23
134	smalls bag4 Pit2 Hor.1 S.1 12.06.22 one each	Gegham 1	2	1	1	12-Jun	93	6/29/23	763	4084	40	226	16	25	74	54	3.03	0.21	0.33	0.72	0.07	0.11	0.33	0.24
135	smalls bag4 Pit2 Hor.1 S.1 12.06.22 one each	Gegham 1	2	1	1	12-Jun	94	6/29/23	672	3790	33	206	14	23	71	54	2.92	0.19	0.32	0.76	0.07	0.11	0.34	0.26
136	smalls bag4 Pit2 Hor.1 S.1 12.06.22 one each	Gegham 1	2	1	1	12-Jun	95	6/29/23	742	4311	35	226	11	25	75	55	2.99	0.15	0.33	0.73	0.05	0.11	0.33	0.24
137	smalls bag4 Pit2 Hor.1 S.1 12.06.22 one each	Gegham 1	2	1	1	12-Jun	96	6/29/23	818	4581	38	241	14	26	73	57	3.28	0.18	0.35	0.77	0.06	0.11	0.30	0.23
138	smalls bag4 Pit2 Hor.1 S.1 12.06.22 one each	Gegham 1	2	1	1	12-Jun	97	6/29/23	676	3917	33	215	13	26	75	55	2.85	0.17	0.34	0.73	0.06	0.12	0.35	0.26
139	smalls bag4 Pit2 Hor.1 S.1 12.06.22 one each	Gegham 1	2	1	1	12-Jun	98	6/29/23	797	4415	37	231	15	26	73	59	3.14	0.20	0.35	0.81	0.06	0.11	0.32	0.26
140	smalls bag4 Pit2 Hor.1 S.1 12.06.22 one each	Gegham 1	2	1	1	12-Jun	99	6/29/23	807	4597	39	233	15	24	73	54	3.18	0.20	0.32	0.73	0.06	0.10	0.31	0.23
141	smalls bag4 Pit2 Hor.1 S.1 12.06.22 one each	Gegham 1	2	1	1	12-Jun	100	6/29/23	794	4415	42	233	15	27	76	54	3.05	0.19	0.35	0.71	0.06	0.11	0.33	0.23
142	smalls bag4 Pit2 Hor.1 S.1 12.06.22 one each	Gegham 1	2	1	1	12-Jun	101	6/29/23	854	4738	43	243	15	24	77	55	3.15	0.19	0.31	0.71	0.06	0.10	0.32	0.23
143	smalls bag4 Pit2 Hor.1 S.1 12.06.22 one each	Gegham 1	2	1	1	12-Jun	102	6/29/23	878	4798	40	244	13	25	73	58	3.32	0.17	0.34	0.79	0.05	0.10	0.30	0.24
144	smalls bag4 Pit2 Hor.1 S.1 12.06.22 one each	Gegham 1	2	1	1	12-Jun	103	6/29/23	797	4288	40	231	14	27	76	58	3.02	0.18	0.35	0.76	0.06	0.12	0.33	0.25
145	smalls bag4 Pit2 Hor.1 S.1 12.06.22 one each	Kelbadjar	2	1	1	12-Jun	104	6/29/23	576	5941	47	177	20	10	118	37	1.50	0.17	0.08	0.32	0.11	0.05	0.67	0.21
146	smalls bag4 Pit2 Hor.1 S.1 12.06.22 one each	Gegham 1	2	1	1	12-Jun	105	6/29/23	852	4711	37	237	16	25	74	57	3.19	0.21	0.33	0.76	0.07	0.10	0.31	0.24
147	smalls bag4 Pit2 Hor.1 S.1 12.06.22 one each	Gegham 1	2	1	1	12-Jun	106	6/29/23	764	4368	44	237	14	27	78	59	3.03	0.17	0.35	0.76	0.06	0.12	0.33	0.25
148	smalls bag4 Pit2 Hor.1 S.1 12.06.22 one each	Gegham 1	2	1	1	12-Jun	107	6/29/23	757	4276	38	226	15	27	78	55	2.89	0.19	0.34	0.71	0.06	0.12	0.35	0.24
149	smalls bag5 Pit2 Hor.1 S.1 12.06.22 one each	Gegham 1	2	1	1	12-Jun	108	6/29/23	853	4715	39	242	16	27	77	57	3.13	0.20	0.34	0.73	0.06	0.11	0.32	0.23
150	smalls bag5 Pit2 Hor.1 S.1 12.06.22 one each	Gegham 1	2	1	1	12-Jun	109	6/29/23	773	4389	36	232	15	27	76	58	3.03	0.19	0.35	0.76	0.06	0.11	0.33	0.25
151	smalls bag5 Pit2 Hor.1 S.1 12.06.22 one each	Gegham 1	2	1	1	12-Jun	110	6/29/23	727	4143	36	220	13	27	76	57	2.88	0.16	0.36	0.74	0.06	0.12	0.35	0.26
152	smalls bag5 Pit2 Hor.1 S.1 12.06.22 one each	Gegham 1	2	1	1	12-Jun	111	6/29/23	680	3908	33	210	14	27	73	55	2.86	0.18	0.37	0.75	0.06	0.13	0.35	0.26
153	smalls bag5 Pit2 Hor.1 S.1 12.06.22 one each	Gegham 1	2	1	1	12-Jun	112	6/29/23	739	4212	36	218	14	27	73	57	2.97	0.18	0.36	0.77	0.06	0.12	0.34	0.26
154	smalls bag5 Pit2 Hor.1 S.1 12.06.22 one each	Arteni	2	1	1	12-Jun	113	6/29/23	647	5023	49	144	34	27	92	31	1.56	0.36	0.30	0.33	0.23	0.19	0.64	0.21
155	smalls bag5 Pit2 Hor.1 S.1 12.06.22 one each	Syunik	2	1	1	12-Jun	114	6/29/23	494	5238	44	192	25	11	104	36	1.85	0.24	0.11	0.35	0.13	0.06	0.54	0.19
156	smalls bag5 Pit2 Hor.1 S.1 12.06.22 one each	Kelbadjar	2	1	1	12-Jun	115	6/29/23	577	5868	48	178	21	10	118	33	1.51	0.17	0.08	0.28	0.12	0.05	0.66	0.19
157	smalls bag5 Pit2 Hor.1 S.1 12.06.22 one each	Kelbadjar	2	1	1	12-Jun	116	6/29/23	609	6049	45	176	17	10	111	35	1.58	0.15	0.09	0.31	0.09	0.05	0.63	0.20
158	smalls bag5 Pit2 Hor.1 S.1 12.06.22 one each	Gegham 1	2	1	1	12-Jun	117	6/29/23	754	4113	35	219	14	26	73	54	2.98	0.18	0.35	0.73	0.06	0.12	0.34	0.25
159	smalls bag5 Pit2 Hor.1 S.1 12.06.22 one each	Gegham 1	2	1	1	12-Jun	118	6/29/23	720	3983	36	219	15	25	74	58	2.94	0.19	0.33	0.78	0.07	0.11	0.34	0.26
160	smalls bag5 Pit2 Hor.1 S.1 12.06.22 one each	Syunik	2	1	1	12-Jun	119	6/29/23	690	5322	48	244	7	7	98	46	2.49	0.08	0.07	0.47	0.03	0.03	0.40	0.19
161	smalls bag5 Pit2 Hor.1 S.1 12.06.22 one each	Gegham 1	2	1	1	12-Jun	120	6/29/23	691	4019	33	219	14	26	73	58	2.98	0.18	0.35	0.79	0.06	0.12	0.34	0.26
162	smalls bag5 Pit2 Hor.1 S.1 12.06.22 one each	Gegham 1	2	1	1	12-Jun	121	6/29/23	754	4290	36	226	13	28	74	61	3.03	0.17	0.38	0.82	0.06	0.13	0.33	0.27
163	smalls bag5 Pit2 Hor.1 S.1 12.06.22 one each	Gegham 1	2	1	1	12-Jun	122	6/29/23	961	5204	49	253	15	26	75	57	3.35	0.19	0.34	0.75	0.06	0.10	0.30	0.22
164	smalls bag5 Pit2 Hor.1 S.1 12.06.22 one each	Gegham 1	2	1	1	12-Jun	123	6/29/23	803	4525	39	236	14	26	75	57	3.14	0.18	0.34	0.75	0.06	0.11	0.32	0.24
165	smalls bag5 Pit2 Hor.1 S.1 12.06.22 one each	Gegham 1	2	1	1	12-Jun	124	6/29/23	742	3986	38	217	13	24	74	57	2.91	0.17	0.32	0.76	0.06	0.11	0.34	0.26
166	smalls bag5 Pit2 Hor.1 S.1 12.06.22 one each	Gegham 1	2	1	1	12-Jun	125	6/29/23	746	4180	34	221	15	25	73	54	3.04	0.20	0.34	0.74	0.07	0.11	0.33	0.24
167	smalls bag5 Pit2 Hor.1 S.1 12.06.22 one each	Gegham 1	2	1	1	12-Jun	126	6/29/23	807	4527	36	234	14	25	75	57	3.11	0.18	0.33	0.75	0.06	0.11	0.32	0.24
168	smalls bag5 Pit2 Hor.1 S.1 12.06.22 one each	Kelbadjar	2	1	1	12-Jun	127	6/29/23	549	5577	45	170	18	11	114	37	1.49	0.15	0.10	0.33	0.10	0.07	0.67	0.22
169	smalls bag5 Pit2 Hor.1 S.1 12.06.22 one each	Kelbadjar	2	1	1	12-Jun	128	6/29/23	575	5992	46	178	17	11	109	32	1.63	0.15	0.10	0.29	0.09	0.06	0.61	0.18
170	smalls bag5 Pit2 Hor.1 S.1 12.06.22 one each	Gegham 1	2	1	1	12-Jun	129	6/29/23	722	3991	37	216	13	25	74	57	2.90	0.17	0.33	0.76	0.06	0.11	0.34	0.26
171	smalls bag5 Pit2 Hor.1 S.1 12.06.22 one each	Gegham 1	2	1	1	12-Jun	130	6/29/23	955	5263	49	254	14	26	78	54	3.25	0.17	0.33	0.69	0.05	0.10	0.31	0.21
172	smalls bag5 Pit2 Hor.1 S.1 12.06.22 one each	Gegham 1	2	1	1	12-Jun	131	6/29/23	920	5163	46	242	15	27	74	57	3.25	0.19	0.36	0.76	0.06	0.11	0.31	0.23
173	smalls bag5 Pit2 Hor.1 S.1 12.06.22 one each	Gegham 1	2	1	1	12-Jun	132	6/29/23	792	4387	38	234	14	27	74	54	3.15	0.18	0.36	0.72	0.06	0.11	0.32	0.23
174	smalls bag5 Pit2 Hor.1 S.1 12.06.22 one each	Gegham 1	2	1	1	12-Jun	133	6/29/23	792	4555	39	240	14	24	73	54	3.31	0.19	0.33	0.74	0.06	0.10	0.30	0.22
175	smalls bag5 Pit2 Hor.1 S.1 12.06.22 one each	Gegham 1	2	1	1	12-Jun	134	6/29/23	763	4238	39	223	14	25	74	55	2.99	0.18	0.33	0.74	0.06	0.11	0.33	0.25
176	smalls bag5 Pit2 Hor.1 S.1 12.06.22 one each	Gegham 1	2	1	1	12-Jun	135	6/29/23	860	4527	37	236	13	27	75	55	3.14	0.17	0.35	0.73	0.05	0.11	0.32	0.23

177	smalls bag5 Pit2 Hor.1 S.1 12.06.22 one each	Gegham 1	2	1	1	12-Jun	136	6/29/23	756	4181	40	233	14	25	74	55	3.12	0.18	0.33	0.74	0.06	0.11	0.32	0.24
178	smalls bag5 Pit2 Hor.1 S.1 12.06.22 one each	Gegham 1	2	1	1	12-Jun	137	6/29/23	731	4220	40	220	14	25	75	57	2.92	0.18	0.33	0.75	0.06	0.11	0.34	0.26
179	smalls bag5 Pit2 Hor.1 S.1 12.06.22 one each	Syunik	2	1	1	12-Jun	138	6/29/23	661	6566	57	188	19	9	116	36	1.62	0.16	0.07	0.31	0.10	0.05	0.62	0.19
180	smalls bag5 Pit2 Hor.1 S.1 12.06.22 one each	Gegham 1	2	1	1	12-Jun	139	6/29/23	776	4168	35	219	14	26	73	57	3.02	0.19	0.35	0.78	0.06	0.12	0.33	0.26
181	smalls bag5 Pit2 Hor.1 S.1 12.06.22 one each	Syunik	2	1	1	12-Jun	140	6/29/23	787	7835	63	208	21	11	119	37	1.75	0.17	0.10	0.31	0.10	0.06	0.57	0.18
182	smalls bag5 Pit2 Hor.1 S.1 12.06.22 one each	Kelbadjar	2	1	1	12-Jun	141	6/29/23	523	5432	54	169	20	11	113	36	1.50	0.17	0.09	0.32	0.12	0.06	0.67	0.21
183	smalls bag5 Pit2 Hor.1 S.1 12.06.22 one each	Gegham 1	2	1	1	12-Jun	142	6/29/23	769	4270	38	225	14	23	72	58	3.14	0.19	0.32	0.81	0.06	0.10	0.32	0.26
184	smalls bag5 Pit2 Hor.1 S.1 12.06.22 one each	Syunik	2	1	1	12-Jun	143	6/29/23	486	5493	40	191	23	10	106	35	1.81	0.21	0.09	0.33	0.12	0.05	0.55	0.18
185	smalls bag5 Pit2 Hor.1 S.1 12.06.22 one each	Gegham 1	2	1	1	12-Jun	145	6/29/23	799	4120	36	226	15	27	75	55	2.99	0.19	0.36	0.73	0.06	0.12	0.33	0.24
186	smalls bag5 Pit2 Hor.1 S.1 12.06.22 one each	Kelbadjar	2	1	1	12-Jun	146	6/29/23	701	6648	59	191	19	11	114	37	1.67	0.16	0.09	0.33	0.10	0.06	0.60	0.20
187	smalls bag5 Pit2 Hor.1 S.1 12.06.22 one each	Gegham 1	2	1	1	12-Jun	147	6/29/23	934	5067	45	254	15	24	79	55	3.21	0.18	0.30	0.70	0.06	0.09	0.31	0.22
188	smalls bag5 Pit2 Hor.1 S.1 12.06.22 one each	Kelbadjar	2	1	1	12-Jun	148	6/29/23	678	6671	55	189	18	11	121	37	1.56	0.15	0.09	0.31	0.09	0.06	0.64	0.20
189	smalls bag5 Pit2 Hor.1 S.1 12.06.22 one each	Gegham 2	2	1	1	12-Jun	149	6/29/23	756	5694	53	143	45	26	91	29	1.56	0.49	0.28	0.32	0.31	0.18	0.64	0.20
190	smalls bag5 Pit2 Hor.1 S.1 12.06.22 one each	Gegham 1	2	1	1	12-Jun	150	6/29/23	844	4754	94	209	16	27	77	58	2.71	0.20	0.36	0.75	0.07	0.13	0.37	0.28
191	smalls bag5 Pit2 Hor.1 S.1 12.06.22 one each	Gegham 1	2	1	1	12-Jun	151	6/29/23	1064	5414	48	268	15	26	82	57	3.27	0.18	0.31	0.69	0.05	0.10	0.31	0.21
192	smalls bag5 Pit2 Hor.1 S.1 12.06.22 one each	Kelbadjar	2	1	1	12-Jun	152	6/29/23	596	5846	52	181	18	11	119	39	1.52	0.15	0.10	0.33	0.10	0.06	0.66	0.21
193	smalls bag5 Pit2 Hor.1 S.1 12.06.22 one each	Gegham 1	2	1	1	12-Jun	153	6/29/23	776	4408	46	227	14	26	73	55	3.08	0.18	0.35	0.75	0.06	0.11	0.32	0.24
194	smalls bag5 Pit2 Hor.1 S.1 12.06.22 one each	Gegham 1	2	1	1	12-Jun	154	6/29/23	844	4579	41	242	16	25	77	58	3.13	0.20	0.32	0.75	0.06	0.10	0.32	0.24
195	Trench1 Hor5 Sp1 23-Jan-22 one each	Gegham 1	1	5	1	23-Jun	163	6/29/23	671	3720	43	205	14	25	70	51	2.95	0.19	0.35	0.73	0.07	0.12	0.34	0.25
196	Trench1 Hor5 Sp1 23-Jan-22 one each	Gegham 1	1	5	1	23-Jun	164	6/29/23	710	4103	37	213	14	26	73	58	2.94	0.19	0.35	0.80	0.06	0.12	0.34	0.27
197	Trench1 Hor5 Sp1 23-Jan-22 one each	Gegham 1	1	5	1	23-Jun	165	6/29/23	710	3861	32	205	10	26	73	55	2.79	0.14	0.35	0.75	0.05	0.12	0.36	0.27
198	Trench1 Hor5 Sp1 23-Jan-22 one each	Gegham 1	1	5	1	23-Jun	166	6/29/23	657	3751	36	205	14	27	70	50	2.95	0.19	0.39	0.71	0.07	0.13	0.34	0.24
199	Trench1 Hor5 Sp1 23-Jan-22 one each	Gegham 1	1	5	1	23-Jun	167	6/29/23	620	3684	32	203	13	24	69	51	2.96	0.18	0.34	0.74	0.06	0.12	0.34	0.25
200	Trench1 Hor5 Sp1 23-Jan-22 one each	Gegham 1	1	5	1	23-Jun	168	6/29/23	746	4056	33	219	13	24	72	54	3.06	0.17	0.33	0.75	0.06	0.11	0.33	0.25
201	Trench1 Hor5 Sp1 23-Jan-22 one each	Gegham 1	1	5	1	23-Jun	169	6/29/23	680	3967	31	207	14	26	71	54	2.93	0.19	0.36	0.76	0.07	0.12	0.34	0.26
202	Trench1 Hor5 Sp1 23-Jan-22 one each	Kelbadjar	1	5	1	23-Jun	170	6/29/23	523	5549	43	165	18	10	108	37	1.54	0.16	0.09	0.35	0.11	0.06	0.65	0.23
203	Trench1 Hor5 Sp1 23-Jan-22 one each	Gegham 1	1	5	1	23-Jun	171	6/29/23	700	3989	37	210	14	24	71	55	2.97	0.19	0.34	0.78	0.06	0.11	0.34	0.26
204	Trench1 Hor5 Sp1 23-Jan-22 one each	Gegham 1	1	5	1	23-Jun	172	6/29/23	711	4022	36	213	15	26	74	57	2.86	0.19	0.34	0.76	0.07	0.12	0.35	0.27
205	Trench1 Hor5 Sp1 23-Jan-22 one each	Gegham 1	1	5	1	23-Jun	173	6/29/23	681	4007	33	209	14	24	70	55	3.00	0.19	0.34	0.79	0.06	0.11	0.33	0.26
206	Trench1 Hor5 Sp1 23-Jan-22 one each	Gegham 1	1	5	1	23-Jun	174	6/29/23	671	4249	35	209	18	24	72	54	2.92	0.24	0.33	0.75	0.08	0.11	0.34	0.26
207	Trench1 Hor5 Sp1 23-Jan-22 one each	Gegham 1	1	5	1	23-Jun	175	6/29/23	646	4047	33	212	15	23	71	55	3.00	0.21	0.32	0.78	0.07	0.11	0.33	0.26
208	Trench1 Hor5 Sp1 23-Jan-22 one each	Gegham 1	1	5	1	23-Jun	176	6/29/23	685	3891	33	206	13	25	73	54	2.81	0.17	0.34	0.73	0.06	0.12	0.36	0.26
209	Trench1 Hor5 Sp1 23-Jan-22 one each	Gegham 1	1	5	1	23-Jun	177	6/29/23	691	4089	36	215	13	26	72	51	3.00	0.17	0.36	0.71	0.06	0.12	0.33	0.24
210	Trench1 Hor5 Sp1 23-Jan-22 one each	Gegham 1	1	5	1	23-Jun	178	6/29/23	648	4289	35	209	19	24	72	54	2.92	0.26	0.33	0.75	0.09	0.11	0.34	0.26
211	Trench1 Hor5 Sp1 23-Jan-22 one each	Gegham 1	1	5	1	23-Jun	179	6/29/23	658	3894	63	214	14	25	70	53	3.07	0.19	0.35	0.75	0.06	0.12	0.33	0.25
212	Trench1 Hor5 Sp1 23-Jan-22 one each	Gegham 1	1	5	1	23-Jun	180	6/29/23	700	3923	35	209	14	26	73	58	2.85	0.18	0.35	0.79	0.06	0.12	0.35	0.28
213	Trench1 Hor5 Sp1 23-Jan-22 one each	Gegham 1	1	5	1	23-Jun	181	6/29/23	710	3940	33	215	14	25	71	55	3.04	0.19	0.35	0.78	0.06	0.11	0.33	0.26
214	Trench1 Hor5 Sp1 23-Jan-22 one each	Gegham 1	1	5	1	23-Jun	182	6/29/23	737	4272	34	224	14	26	75	58	2.97	0.18	0.34	0.77	0.06	0.11	0.34	0.26
215	Trench1 Hor5 Sp1 23-Jan-22 one each	Gegham 1	1	5	1	23-Jun	183	6/29/23	627	3821	40	203	20	24	71	54	2.88	0.28	0.34	0.76	0.10	0.12	0.35	0.26
216	Trench1 Hor5 Sp1 23-Jan-22 one each	Gegham 1	1	5	1	23-Jun	184	6/29/23	666	3904	33	214	14	25	72	54	2.99	0.19	0.34	0.75	0.06	0.12	0.33	0.25
217	Trench1 Hor5 Sp1 23-Jan-22 one each	Gegham 1	1	5	1	23-Jun	185	6/29/23	621	3530	34	197	15	27	70	53	2.82	0.21	0.38	0.75	0.07	0.13	0.35	0.27
218	Trench1 Hor5 Sp1 23-Jan-22 one each	Gegham 1	1	5	1	23-Jun	186	6/29/23	711	4023	34	219	13	25	69	53	3.18	0.18	0.36	0.76	0.06	0.11	0.31	0.24
219	Trench1 Hor5 Sp1 23-Jan-22 one each	Gegham 1	1	5	1	23-Jun	187	6/29/23	645	3808	34	209	14	27	74	57	2.81	0.18	0.36	0.76	0.06	0.13	0.36	0.27
220	Trench1 Hor5 Sp1 23-Jan-22 one each	Gegham 1	1	5	1	23-Jun	188	6/29/23	631	3711	38	205	14	25	74	55	2.76	0.18	0.33	0.74	0.07	0.12	0.36	0.27
221	Trench1 Hor5 Sp1 23-Jan-22 one each	Gegham 1	1	5	1	23-Jun	189	6/29/23	757	4277	38	225	14	25	74	55	3.02	0.18	0.33	0.74	0.06	0.11	0.33	0.25
222	Trench1 Hor5 Sp1 23-Jan-22 one each	Gegham 1	1	5	1	23-Jun	190	6/29/23	763	3972	35	221	13	27	75	54	2.93	0.17	0.35	0.71	0.06	0.12	0.34	0.24
223	Trench1 Hor5 Sp1 23-Jan-22 one each	Gegham 1	1	5	1	23-Jun	191	6/29/23	746	4149	38	216	14	26	70	53	3.10	0.19	0.37	0.75	0.06	0.12	0.32	0.24
224	Trench1 Hor5 Sp1 23-Jan-22 one each	Gegham 1	1	5	1	23-Jun	192	6/29/23	671	3882	35	212	11	27	73	57	2.89	0.16	0.36	0.77	0.05	0.13	0.35	0.27
225	Trench1 Hor5 Sp1 23-Jan-22 one each	Gegham 1	1	5	1	23-Jun	193	6/29/23	703	4073	34	219	13	27	75	57	2.90	0.17	0.35	0.75	0.06	0.12	0.34	0.26
226	Trench1 Hor5 Sp1 23-Jan-22 one each	Gegham 1	1	5	1	23-Jun	194	6/29/23	776	4112	37	224	15	25	75	57	2.97	0.19	0.33	0.75	0.06	0.11	0.34	0.25
227	Trench1 Hor5 Sp1 23-Jan-22 one each	Gegham 1	1	5	1	23-Jun	195	6/29/23	703	3933	32	214	15	27	73	53	2.91	0.20	0.36	0.71	0.07	0.12	0.34	0.25

228	Trench1 Hor5 Sp1 23-Jan-22 one each	Kelbadjar	1	5	1	23-Jun	196	6/29/23	476	5191	36	173	24	12	103	33	1.69	0.23	0.12	0.32	0.14	0.07	0.59	0.19
229	Trench1 Hor5 Sp1 23-Jan-22 one each	Syunik	1	5	1	23-Jun	197	6/29/23	530	5525	36	191	21	12	103	36	1.86	0.20	0.12	0.35	0.11	0.07	0.54	0.19
230	Trench1 Hor5 Sp1 23-Jan-22 one each	Gegham 1	1	5	1	23-Jun	198	6/29/23	681	3863	34	210	14	25	75	57	2.79	0.18	0.33	0.75	0.06	0.12	0.36	0.27
231	Trench1 Hor5 Sp1 23-Jan-22 one each	Gegham 1	1	5	1	23-Jun	199	6/29/23	600	3751	35	205	16	25	73	53	2.83	0.21	0.34	0.72	0.08	0.12	0.35	0.26
232	Trench1 Hor5 Sp1 23-Jan-22 one each	Gegham 1	1	5	1	23-Jun	200	6/29/23	772	4252	35	223	14	23	73	57	3.07	0.19	0.31	0.78	0.06	0.10	0.33	0.25
233	Trench1 Hor5 Sp1 23-Jan-22 one each	Gegham 1	1	5	1	23-Jun	201	6/29/23	789	4551	42	240	17	25	74	54	3.23	0.22	0.33	0.72	0.07	0.10	0.31	0.22
234	Trench1 Hor5 Sp1 23-Jan-22 one each	Syunik	1	5	1	23-Jun	202	6/29/23	666	6546	48	187	19	11	113	36	1.65	0.16	0.09	0.32	0.10	0.06	0.61	0.19
235	Trench1 Hor5 Sp1 23-Jan-22 one each	Syunik	1	5	1	23-Jun	203	6/29/23	536	5622	41	192	24	9	106	36	1.81	0.22	0.08	0.34	0.12	0.05	0.55	0.19
236	Trench1 Hor5 Sp1 23-Jan-22 one each	Gegham 1	1	5	1	23-Jun	204	6/29/23	718	4467	38	217	15	26	74	55	2.91	0.19	0.34	0.74	0.07	0.12	0.34	0.25
237	Trench1 Hor5 Sp1 23-Jan-22 one each	Gegham 1	1	5	1	23-Jun	205	6/29/23	757	4249	37	225	15	27	74	54	3.02	0.19	0.36	0.72	0.06	0.12	0.33	0.24
238	obs obtool frag ? surface finds Jun 2022	Gegham 1	surface	surface	-	?	217	6/29/23	681	3874	36	202	14	27	73	54	2.75	0.18	0.36	0.73	0.07	0.13	0.36	0.27
240	Tr-1 Hor2 larger 1 of 2 brkn tool?	Gegham 1	1	2	?	?	47	6/30/23	711	4309	39	215	14	26	73	53	2.96	0.19	0.35	0.72	0.06	0.12	0.34	0.24
242	Tr-1 Hor2 smaller 2 of 2 debitage	Gegham 1	1	2	?	?	49	6/30/23	720	4156	34	214	13	25	72	55	2.99	0.17	0.34	0.77	0.06	0.12	0.33	0.26
244	Trench-2 Horizon-4 Spit-2 Ash Layer 22Jan22 1 of 7 rtch flake	Gegham 1	2	4	2	22-Jun	55	6/30/23	678	3890	34	204	13	23	71	54	2.89	0.18	0.32	0.76	0.06	0.11	0.35	0.26
247	Trench-2 Horizon-4 Spit-2 Ash Layer 22Jan22 2 of 7 bkn flake	Syunik	2	4	2	22-Jun	58	6/30/23	463	5390	35	176	23	9	104	33	1.70	0.22	0.08	0.32	0.13	0.05	0.59	0.19
248	Trench-2 Horizon-4 Spit-2 Ash Layer 22Jan22 3 of 7	Syunik	2	4	2	22-Jun	59	6/30/23	496	5425	39	179	24	10	108	35	1.65	0.22	0.09	0.32	0.13	0.05	0.61	0.19
250	Trench-2 Horizon-4 Spit-2 Ash Layer 22Jan22 4 of 7	Gegham 1	2	4	2	22-Jun	61	6/30/23	680	4197	36	219	14	25	73	54	2.98	0.18	0.34	0.73	0.06	0.11	0.34	0.25
252	Trench-2 Horizon-4 Spit-2 Ash Layer 22Jan22 5 of 7	Gegham 1	2	4	2	22-Jun	63	6/30/23	710	4104	35	214	15	27	76	55	2.80	0.19	0.36	0.72	0.07	0.13	0.36	0.26
254	Trench-2 Horizon-4 Spit-2 Ash Layer 22Jan22 6 of 7	Gegham 1	2	4	2	22-Jun	65	6/30/23	690	4068	38	219	15	24	73	57	2.98	0.20	0.32	0.77	0.07	0.11	0.34	0.26
256	Trench-2 Horizon-4 Spit-2 Ash Layer 22Jan22 7 of 7	Gegham 1	2	4	2	22-Jun	67	6/30/23	746	4037	35	220	14	27	74	61	2.95	0.18	0.36	0.82	0.06	0.12	0.34	0.28
257	PIT1. HOR1. SPIT1. 10.06.22 1 of 10 core frag?	Gegham 1	1	1	1	10-Jun	68	6/30/23	720	4113	37	217	13	26	72	59	3.03	0.17	0.36	0.83	0.06	0.12	0.33	0.27
259	PIT1. HOR1. SPIT1. 10.06.22 2 of 10 rtch flake	Gegham 1	1	1	1	10-Jun	70	6/30/23	655	3986	34	208	14	23	74	51	2.80	0.18	0.31	0.69	0.06	0.11	0.36	0.25
261	PIT1. HOR1. SPIT1. 10.06.22 3 of 10	Gegham 1	1	1	1	10-Jun	72	6/30/23	661	3941	35	209	13	26	72	53	2.92	0.17	0.36	0.73	0.06	0.12	0.34	0.25
263	PIT1. HOR1. SPIT1. 10.06.22 4 of 10	Gegham 1	1	1	1	10-Jun	74	6/30/23	705	3930	34	212	14	25	76	54	2.78	0.18	0.32	0.71	0.06	0.12	0.36	0.25
265	PIT1. HOR1. SPIT1. 10.06.22 5 of 10	Arteni	1	1	1	10-Jun	76	6/30/23	727	3915	48	148	21	35	76	40	1.94	0.27	0.46	0.53	0.14	0.24	0.52	0.27
267	PIT1. HOR1. SPIT1. 10.06.22 6 of 10	Syunik	1	1	1	10-Jun	78	6/30/23	496	5335	35	182	21	11	106	37	1.72	0.19	0.11	0.35	0.11	0.06	0.58	0.21
269	PIT1. HOR1. SPIT1. 10.06.22 7 of 10	Syunik	1	1	1	10-Jun	80	6/30/23	491	4912	42	189	23	12	105	33	1.80	0.22	0.12	0.32	0.12	0.07	0.55	0.18
271	PIT1. HOR1. SPIT1. 10.06.22 8 of 10	Kars-Arpaçay	1	1	1	10-Jun	82	6/30/23	774	8567	81	167	6	55	226	28	0.74	0.03	0.24	0.12	0.04	0.33	1.35	0.17
273	PIT1. HOR1. SPIT1. 10.06.22 9 of 10	Gegham 1	1	1	1	10-Jun	84	6/30/23	815	4430	39	238	15	25	75	55	3.16	0.19	0.33	0.73	0.06	0.10	0.32	0.23
275	PIT1. HOR1. SPIT1. 10.06.22 10 of 10	Gegham 1	1	1	1	10-Jun	86	6/30/23	786	4355	41	232	14	25	75	55	3.07	0.18	0.33	0.73	0.06	0.11	0.33	0.24
277	Tr-2 H-3 Sp-1 17Jun22 one each	Kelbadjar	2	3	1	17-Jun	94	6/30/23	504	5015	34	156	17	9	105	35	1.49	0.16	0.08	0.33	0.11	0.06	0.67	0.22
278	Tr-2 H-3 Sp-1 17Jun22 one each	Gegham 1	2	3	1	17-Jun	96	6/30/23	721	4065	37	216	13	27	75	55	2.87	0.17	0.35	0.73	0.06	0.12	0.35	0.26
279	Tr-2 H-3 Sp-1 17Jun22 one each	Gegham 1	2	3	1	17-Jun	97	6/30/23	706	4134	35	206	15	25	74	51	2.77	0.19	0.33	0.69	0.07	0.12	0.36	0.25
280	Tr-2 H-3 Sp-1 17Jun22 one each	Gegham 1	2	3	1	17-Jun	98	6/30/23	718	4191	34	215	15	27	73	57	2.93	0.20	0.36	0.77	0.07	0.12	0.34	0.26
281	Tr-2 H-3 Sp-1 17Jun22 one each	Syunik	2	3	1	17-Jun	99	6/30/23	468	5155	31	173	25	10	102	32	1.70	0.24	0.09	0.31	0.14	0.06	0.59	0.18
282	Tr-2 H-3 Sp-1 17Jun22 one each	Syunik	2	3	1	17-Jun	100	6/30/23	491	5571	37	181	26	12	109	35	1.65	0.23	0.11	0.32	0.14	0.07	0.60	0.19
283	Tr-2 H-3 Sp-1 17Jun22 one each	Gegham 1	2	3	1	17-Jun	101	6/30/23	747	4110	38	227	17	24	73	50	3.08	0.22	0.32	0.68	0.07	0.10	0.32	0.22
284	Tr-2 H-3 Sp-1 17Jun22 one each	Gegham 1	2	3	1	17-Jun	102	6/30/23	657	4169	33	208	14	25	74	54	2.80	0.18	0.33	0.72	0.06	0.12	0.36	0.26
285	Tr-2 H-3 Sp-1 17Jun22 one each	Gegham 1	2	3	1	17-Jun	103	6/30/23	717	4077	34	213	14	26	74	55	2.86	0.18	0.34	0.74	0.06	0.12	0.35	0.26
286	Tr-2 H-3 Sp-1 17Jun22 one each	Arteni	2	3	1	17-Jun	104	6/30/23	627	4711	39	130	38	27	89	26	1.47	0.42	0.30	0.30	0.29	0.20	0.68	0.20
287	Tr-2 H-3 Sp-1 17Jun22 one each	Kelbadjar	2	3	1	17-Jun	105	6/30/23	576	5675	41	169	18	11	114	36	1.48	0.15	0.10	0.32	0.10	0.07	0.67	0.21
288	Tr-2 H-3 Sp-1 17Jun22 one each	Gegham 1	2	3	1	17-Jun	106	6/30/23	758	4339	38	216	17	24	73	51	2.94	0.22	0.32	0.70	0.08	0.11	0.34	0.24
289	Tr-2 H-3 Sp-1 17Jun22 one each	Gegham 1	2	3	1	17-Jun	107	6/30/23	722	4196	35	219	14	26	77	58	2.83	0.17	0.33	0.75	0.06	0.12	0.35	0.26
290	Tr-2 H-3 Sp-1 17Jun22 one each	Gegham 1	2	3	1	17-Jun	108	6/30/23	677	4094	35	216	13	25	75	55	2.87	0.17	0.33	0.73	0.06	0.11	0.35	0.26
291	Tr-2 H-3 Sp-1 17Jun22 one each	Arteni	2	3	1	17-Jun	109	6/30/23	635	5149	46	137	39	26	92	28	1.49	0.42	0.28	0.30	0.28	0.19	0.67	0.20
292	Tr-2 H-3 Sp-1 17Jun22 own bag core frag??	Gegham 1	2	3	1	17-Jun	110	6/30/23	680	3729	34	201	11	25	70	51	2.89	0.16	0.35	0.73	0.06	0.12	0.35	0.25
294	Trench-2 Hor-4 Spit-2 22Jun22 one each	Kelbadjar	2	4	2	22-Jun	112	6/30/23	521	5309	40	161	19	10	107	36	1.51	0.17	0.09	0.34	0.12	0.06	0.66	0.22
295	Trench-2 Hor-4 Spit-2 22Jun22 one each	Syunik	2	4	2	22-Jun	113	6/30/23	505	5356	35	175	25	11	105	35	1.67	0.23	0.11	0.33	0.14	0.07	0.60	0.20
296	Trench-2 Hor-4 Spit-2 22Jun22 one each	Gegham 1	2	4	2	22-Jun	114	6/30/23	655	3995	35	198	18	22	73	47	2.74	0.24	0.30	0.65	0.09	0.11	0.37	0.24
297	Trench-2 Hor-4 Spit-2 22Jun22 one each	Gegham 1	2	4	2	22-Jun	115	6/30/23	784	4398	37	222	15	25	78	57	2.84	0.19	0.32	0.72	0.07	0.11	0.35	0.26
298	Trench-2 Hor-4 Spit-2 22Jun22 one each	Syunik	2	4	2	22-Jun	116	6/30/23	602	4724	37	224	8	11	95	43	2.35	0.09	0.12	0.45	0.04	0.05	0.43	0.19

299	Trench-2 Hor-4 Spit-2 22Jun22 one each	Gegham 1	2	4	2	22-Jun	117	6/30/23	757	4312	36	227	14	27	75	51	3.01	0.18	0.35	0.68	0.06	0.12	0.33	0.23
300	Trench-2 Hor-4 Spit-2 22Jun22 one each	Gegham 1	2	4	2	22-Jun	118	6/30/23	784	4386	35	226	14	25	77	62	2.92	0.17	0.32	0.80	0.06	0.11	0.34	0.28
301	Trench-2 Hor-4 Spit-2 22Jun22 one each	Gegham 1	2	4	2	22-Jun	119	6/30/23	675	4075	35	216	13	23	73	58	2.94	0.17	0.31	0.79	0.06	0.11	0.34	0.27
302	Trench-2 Hor-4 Spit-2 22Jun22 one each	Gegham 1	2	4	2	22-Jun	120	6/30/23	772	4558	42	230	16	26	73	51	3.12	0.21	0.35	0.70	0.07	0.11	0.32	0.22
303	Trench-2 Hor-4 Spit-2 22Jun22 one each	Gegham 1	2	4	2	22-Jun	121	6/30/23	700	4060	30	213	13	26	74	57	2.86	0.17	0.34	0.76	0.06	0.12	0.35	0.27
304	Trench-2 Hor-4 Spit-2 22Jun22 one each	Syunik	2	4	2	22-Jun	122	6/30/23	560	5815	40	203	22	10	107	35	1.91	0.20	0.09	0.33	0.11	0.05	0.52	0.17
305	Trench-2 Hor-4 Spit-2 22Jun22 one each	Kelbadjar	2	4	2	22-Jun	123	6/30/23	501	5331	42	161	17	12	113	36	1.42	0.15	0.11	0.32	0.10	0.08	0.70	0.22
306	Trench-2 Hor-4 Spit-2 22Jun22 one each	Gegham 1	2	4	2	22-Jun	124	6/30/23	726	4288	38	219	15	26	77	57	2.83	0.19	0.33	0.73	0.07	0.12	0.35	0.26
307	Trench-2 Hor-4 Spit-2 22Jun22 one each	Gegham 1	2	4	2	22-Jun	125	6/30/23	829	4443	40	233	14	28	75	54	3.10	0.18	0.38	0.71	0.06	0.12	0.32	0.23
308	Trench-2 Hor-4 Spit-2 22Jun22 one each	Gegham 1	2	4	2	22-Jun	126	6/30/23	769	4386	36	214	14	24	73	58	2.91	0.18	0.32	0.79	0.06	0.11	0.34	0.27
309	Trench-2 Hor-4 Spit-2 22Jun22 one each	Syunik	2	4	2	22-Jun	127	6/30/23	444	5040	34	175	20	11	99	35	1.77	0.20	0.12	0.35	0.11	0.07	0.57	0.20
310	Trench-2 Hor-4 Spit-2 22Jun22 one each	Gegham 1	2	4	2	22-Jun	128	6/30/23	736	4006	33	216	14	27	74	58	2.90	0.18	0.36	0.78	0.06	0.12	0.34	0.27
311	Trench-2 Hor-4 Spit-2 22Jun22 one each	Gegham 1	2	4	2	22-Jun	129	6/30/23	637	3742	32	206	11	25	71	55	2.92	0.16	0.35	0.78	0.06	0.12	0.34	0.27
312	Trench-2 Hor-4 Spit-2 22Jun22 one each	Gegham 1	2	4	2	22-Jun	130	6/30/23	716	4131	35	216	14	25	73	57	2.98	0.19	0.34	0.78	0.06	0.11	0.34	0.26
313	Trench-2 Hor-4 Spit-2 22Jun22 one each	Arteni	2	4	2	22-Jun	131	6/30/23	768	4817	47	161	24	31	83	33	1.94	0.28	0.38	0.40	0.15	0.19	0.52	0.21
314	Trench-2 Hor-4 Spit-2 22Jun22 one each	Gegham 1	2	4	2	22-Jun	132	6/30/23	734	4108	30	218	14	25	73	59	2.97	0.18	0.34	0.81	0.06	0.11	0.34	0.27
315	Trench-2 Hor-4 Spit-2 22Jun22 one each	Gegham 1	2	4	2	22-Jun	133	6/30/23	720	4135	33	220	15	25	76	55	2.88	0.19	0.32	0.72	0.07	0.11	0.35	0.25
316	Trench-2 Hor-4 Spit-2 22Jun22 one each	Gegham 1	2	4	2	22-Jun	134	6/30/23	731	5788	51	232	27	25	76	57	3.03	0.35	0.32	0.74	0.11	0.11	0.33	0.24
317	Trench-2 Hor-4 Spit-2 22Jun22 one each	Gegham 1	2	4	2	22-Jun	135	6/30/23	840	4660	40	233	15	26	73	55	3.16	0.20	0.35	0.75	0.06	0.11	0.32	0.24
318	Trench-2 Hor-4 Spit-2 22Jun22 one each	Gegham 1	2	4	2	22-Jun	136	6/30/23	711	3961	37	220	17	27	76	57	2.88	0.22	0.35	0.74	0.08	0.12	0.35	0.26
319	Trench-2 Hor-4 Spit-2 22Jun22 one each	Gegham 1	2	4	2	22-Jun	137	6/30/23	758	4082	37	224	13	26	74	55	3.01	0.17	0.34	0.74	0.06	0.11	0.33	0.25
320	Trench-2 Hor-4 Spit-2 22Jun22 one each	Syunik	2	4	2	22-Jun	138	6/30/23	516	5326	37	192	22	11	108	35	1.78	0.20	0.11	0.32	0.11	0.06	0.56	0.18
321	Trench-2 Hor-4 Spit-2 22Jun22 one each	Gegham 1	2	4	2	22-Jun	139	6/30/23	918	4873	44	245	15	25	72	55	3.42	0.20	0.33	0.77	0.06	0.10	0.29	0.23
322	Trench-2 Hor-4 Spit-2 22Jun22 one each	Gegham 1	2	4	2	22-Jun	140	6/30/23	779	4395	46	234	16	24	72	55	3.27	0.22	0.33	0.77	0.07	0.10	0.31	0.24
323	Trench-2 Hor-4 Spit-2 22Jun22 one each	Gegham 1	2	4	2	22-Jun	141	6/30/23	849	4626	43	237	14	24	74	54	3.19	0.18	0.32	0.72	0.06	0.10	0.31	0.23
324	Trench-2 Hor-4 Spit-2 22Jun22 one each	Gegham 1	2	4	2	22-Jun	142	6/30/23	850	4736	42	237	15	25	72	55	3.32	0.20	0.34	0.77	0.06	0.10	0.30	0.23
325	Trench-2 Hor-4 Spit-2 22Jun22 one each	Gegham 1	2	4	2	22-Jun	143	6/30/23	808	4501	36	224	15	23	75	55	2.97	0.19	0.30	0.73	0.06	0.10	0.34	0.25
326	Trench-2 Hor-4 Spit-2 22Jun22 one each	Gegham 1	2	4	2	22-Jun	144	6/30/23	687	4218	40	217	14	26	73	53	2.95	0.18	0.35	0.71	0.06	0.12	0.34	0.24
327	Trench-2 Hor-4 Spit-2 22Jun22 one each	Gegham 1	2	4	2	22-Jun	145	6/30/23	855	4754	38	243	15	25	73	51	3.31	0.20	0.34	0.70	0.06	0.10	0.30	0.21
328	Trench-2 Hor-4 Spit-2 22Jun22 one each	Arteni	2	4	2	22-Jun	146	6/30/23	620	4824	42	130	40	27	91	28	1.44	0.44	0.30	0.31	0.30	0.21	0.69	0.21
329	Tr-2 Hor 5 Southern Part 22Jun22 one each	Gegham 1	2	5	South	22-Jun	147	6/30/23	697	3915	34	209	13	27	72	54	2.92	0.17	0.37	0.75	0.06	0.13	0.34	0.26
330	Tr-2 Hor 5 Southern Part 22Jun22 one each	Syunik	2	5	South	22-Jun	148	6/30/23	506	4831	34	180	19	10	101	35	1.78	0.18	0.09	0.34	0.10	0.05	0.56	0.19
331	Tr-2 Hor 5 Southern Part 22Jun22 one each	Gegham 1	2	5	South	22-Jun	149	6/30/23	650	3647	29	198	13	24	70	54	2.83	0.18	0.34	0.77	0.06	0.12	0.35	0.27
332	Tr-2 Hor 5 Southern Part 22Jun22 one each	Gegham 1	2	5	South	22-Jun	150	6/30/23	698	4184	35	217	13	27	73	54	2.95	0.17	0.36	0.73	0.06	0.12	0.34	0.25
333	Tr-2 Hor 5 Southern Part 22Jun22 one each	Gegham 1	2	5	South	22-Jun	151	6/30/23	708	4104	35	211	16	27	73	55	2.91	0.21	0.38	0.76	0.07	0.13	0.34	0.26
334	Tr-2 Hor 5 Southern Part 22Jun22 one each	Gegham 1	2	5	South	22-Jun	152	6/30/23	743	4251	36	222	14	25	75	57	2.94	0.18	0.33	0.75	0.06	0.11	0.34	0.26
335	Tr-2 Hor 5 Southern Part 22Jun22 one each	Syunik	2	5	South	22-Jun	153	6/30/23	528	5520	40	192	21	14	106	37	1.81	0.19	0.14	0.35	0.11	0.07	0.55	0.20
336	Tr-2 Hor 5 Southern Part 22Jun22 one each	Gegham 1	2	5	South	22-Jun	154	6/30/23	681	3793	34	201	13	27	73	53	2.78	0.17	0.37	0.72	0.06	0.13	0.36	0.26
337	Tr-2 Hor 5 Southern Part 22Jun22 one each	Gegham 1	2	5	South	22-Jun	155	6/30/23	756	4267	35	226	15	25	72	55	3.15	0.20	0.34	0.77	0.06	0.11	0.32	0.24
338	Tr-2 Hor 5 Southern Part 22Jun22 one each	Gegham 1	2	5	South	22-Jun	156	6/30/23	666	3849	33	208	14	26	72	54	2.91	0.19	0.36	0.75	0.06	0.12	0.34	0.26
339	Tr-2 Hor 5 Southern Part 22Jun22 one each	Gegham 1	2	5	South	22-Jun	157	6/30/23	728	4143	33	216	13	25	73	57	2.94	0.17	0.34	0.77	0.06	0.11	0.34	0.26
340	Tr-2 Hor 5 Southern Part 22Jun22 one each	Gegham 1	2	5	South	22-Jun	158	6/30/23	673	4082	36	214	15	25	74	57	2.88	0.19	0.33	0.76	0.07	0.12	0.35	0.26
341	Tr-2 Hor 5 Southern Part 22Jun22 one each	Gegham 1	2	5	South	22-Jun	159	6/30/23	650	3919	32	210	15	26	73	54	2.86	0.20	0.35	0.73	0.07	0.12	0.35	0.26
342	Tr-2 Hor 5 Southern Part 22Jun22 one each	Gegham 1	2	5	South	22-Jun	160	6/30/23	723	3997	38	216	14	23	73	55	2.98	0.19	0.31	0.76	0.06	0.11	0.34	0.26
343	Tr-2 Hor 5 Southern Part 22Jun22 one each	Gegham 1	2	5	South	22-Jun	161	6/30/23	702	4058	34	215	11	25	74	54	2.89	0.15	0.33	0.72	0.05	0.11	0.35	0.25
344	Tr-2 Hor 5 Southern Part 22Jun22 one each	Gegham 1	2	5	South	22-Jun	162	6/30/23	777	4188	35	233	15	26	76	57	3.05	0.19	0.34	0.74	0.06	0.11	0.33	0.24
345	Tr-2 Hor 5 Southern Part 22Jun22 one each	Gegham 1	2	5	South	22-Jun	163	6/30/23	682	3957	36	226	14	27	75	57	2.99	0.18	0.35	0.75	0.06	0.12	0.33	0.25
346	Tr-2 Hor 5 Southern Part 22Jun22 one each	Gegham 1	2	5	South	22-Jun	164	6/30/23	638	4077	36	204	17	24	77	53	2.64	0.21	0.31	0.68	0.08	0.12	0.38	0.26
347	Tr-2 Hor 5 Southern Part 22Jun22 one each	Gegham 1	2	5	South	22-Jun	165	6/30/23	657	3873	36	217	18	25	70	55	3.11	0.25	0.35	0.79	0.08	0.11	0.32	0.25
348	Tr-2 Hor-4 Sp-1 20Jun22 one each	Gegham 1	2	4	1	20-Jun	166	6/30/23	748	4224	37	221	14	25	73	57	3.00	0.18	0.34	0.77	0.06	0.11	0.33	0.26
349	Tr-2 Hor-4 Sp-1 20Jun22 one each	Gegham 1	2	4	1	20-Jun	167	6/30/23	852	4894	40	242	16	23	76	59	3.17	0.20	0.30	0.78	0.06	0.09	0.32	0.25

350	Tr-2 Hor-4 Sp-1 20Jun22 one each	Arteni	2	4	1	20-Jun	168	6/30/23	726	4891	48	151	26	29	90	32	1.68	0.29	0.33	0.36	0.17	0.19	0.59	0.21
351	Tr-2 Hor-4 Sp-1 20Jun22 one each	Gegham 1	2	4	1	20-Jun	169	6/30/23	951	5003	44	246	16	26	76	54	3.22	0.20	0.34	0.71	0.06	0.10	0.31	0.22
352	Tr-2 Hor-4 Sp-1 20Jun22 one each	Gegham 1	2	4	1	20-Jun	185	6/30/23	662	4181	38	206	16	27	72	55	2.88	0.22	0.37	0.77	0.08	0.13	0.35	0.27
353	Tr-2 Hor-4 Sp-1 20Jun22 one each	Syunik	2	4	1	20-Jun	186	6/30/23	473	5301	36	175	24	9	103	31	1.70	0.23	0.08	0.30	0.13	0.05	0.59	0.17
354	Tr-2 Hor-4 Sp-1 20Jun22 one each	Gegham 1	2	4	1	20-Jun	187	6/30/23	705	4470	33	208	17	28	72	57	2.91	0.23	0.40	0.79	0.08	0.14	0.34	0.27
355	Tr-2 Hor-4 Sp-1 20Jun22 one each	Gegham 1	2	4	1	20-Jun	188	6/30/23	662	4116	36	209	16	26	74	54	2.81	0.21	0.34	0.72	0.07	0.12	0.36	0.26
356	Tr-2 Hor-4 Sp-1 20Jun22 one each	Syunik	2	4	1	20-Jun	189	6/30/23	638	5197	41	221	11	11	95	44	2.32	0.12	0.11	0.46	0.05	0.05	0.43	0.20
357	Tr-2 Hor-4 Sp-1 20Jun22 one each	Kelbadjar	2	4	1	20-Jun	190	6/30/23	499	5160	40	151	19	12	106	35	1.43	0.18	0.12	0.33	0.12	0.08	0.70	0.23
358	Tr-2 Hor-4 Sp-1 20Jun22 one each	Arteni	2	4	1	20-Jun	191	6/30/23	531	5062	41	117	45	24	89	28	1.32	0.50	0.27	0.31	0.38	0.20	0.76	0.24
359	Tr-2 Hor-4 Sp-1 20Jun22 one each	Gegham 1	2	4	1	20-Jun	192	6/30/23	734	4598	39	220	16	27	78	58	2.81	0.20	0.34	0.74	0.07	0.12	0.36	0.26
360	Tr-2 Hor-4 Sp-1 20Jun22 one each	Syunik	2	4	1	20-Jun	193	6/30/23	666	4823	37	226	9	10	96	43	2.35	0.10	0.10	0.45	0.04	0.04	0.43	0.19
361	Tr-2 Hor-4 Sp-1 20Jun22 one each	Gegham 1	2	4	1	20-Jun	194	6/30/23	647	4087	35	209	14	27	74	53	2.81	0.18	0.36	0.71	0.06	0.13	0.36	0.25
362	Tr-2 Hor-4 Sp-1 20Jun22 one each	Kelbadjar	2	4	1	20-Jun	195	6/30/23	513	5626	42	170	19	11	108	35	1.58	0.17	0.11	0.32	0.11	0.07	0.63	0.20
363	Tr-2 Hor-4 Sp-1 20Jun22 one each	Gegham 1	2	4	1	20-Jun	196	6/30/23	722	4589	35	220	17	26	77	57	2.85	0.21	0.33	0.73	0.08	0.12	0.35	0.26
364	Tr-2 Hor-4 Sp-1 20Jun22 one each	Gegham 1	2	4	1	20-Jun	197	6/30/23	754	4919	41	222	26	25	71	54	3.14	0.36	0.35	0.76	0.12	0.11	0.32	0.24
365	Tr-2 Hor-4 Sp-1 20Jun22 one each	Arteni	2	4	1	20-Jun	198	6/30/23	726	4881	43	146	25	31	90	35	1.63	0.27	0.35	0.39	0.17	0.21	0.61	0.24
366	Tr-2 Hor-4 Sp-1 20Jun22 one each	Syunik	2	4	1	20-Jun	199	6/30/23	518	5235	37	187	19	11	105	39	1.78	0.18	0.10	0.37	0.10	0.06	0.56	0.21
367	Tr-2 Hor-4 Sp-1 20Jun22 one each	Gegham 1	2	4	1	20-Jun	200	6/30/23	696	4151	33	210	14	26	78	54	2.69	0.17	0.33	0.69	0.06	0.12	0.37	0.26
368	Tr-2 Hor-4 Sp-1 20Jun22 one each	Gegham 1	2	4	1	20-Jun	201	6/30/23	809	4916	39	239	15	26	76	53	3.14	0.19	0.34	0.69	0.06	0.11	0.32	0.22
369	Tr-2 Hor-4 Sp-1 20Jun22 one each	Arteni	2	4	1	20-Jun	202	6/30/23	663	4201	41	141	22	30	89	35	1.59	0.24	0.34	0.39	0.15	0.21	0.63	0.25
370	Tr-2 Hor-4 Sp-1 20Jun22 one each	Gegham 1	2	4	1	20-Jun	203	6/30/23	811	4469	46	234	16	27	72	54	3.27	0.22	0.37	0.75	0.07	0.11	0.31	0.23
371	Tr-2 Hor-4 Sp-1 20Jun22 one each	Gegham 1	2	4	1	20-Jun	204	6/30/23	652	3900	44	215	24	25	73	54	2.96	0.32	0.34	0.74	0.11	0.11	0.34	0.25
372	Tr-2 Hor-4 Sp-1 20Jun22 one each	Gegham 1	2	4	1	20-Jun	205	6/30/23	697	4031	37	222	15	25	73	54	3.06	0.20	0.34	0.74	0.07	0.11	0.33	0.24
373	Tr-2 Hor-4 Sp-1 20Jun22 one each	Gegham 1	2	4	1	20-Jun	206	6/30/23	783	4532	39	227	14	25	73	55	3.12	0.19	0.34	0.76	0.06	0.11	0.32	0.24
374	Tr-2 Hor-2 Sp-2 floatation one each	Kelbadjar	2	2	2	?	213	6/30/23	510	5518	39	167	18	10	111	36	1.50	0.16	0.09	0.32	0.10	0.06	0.66	0.22
375	Tr-2 Hor-2 Sp-2 floatation one each	Gegham 1	2	2	2	?	214	6/30/23	673	3808	28	206	11	25	70	55	2.96	0.16	0.35	0.79	0.06	0.12	0.34	0.27
376	Tr-2 Hor-2 Sp-2 floatation one each	Gegham 1	2	2	2	?	215	6/30/23	768	4444	40	223	16	27	76	57	2.92	0.20	0.35	0.74	0.07	0.12	0.34	0.25
377	Tr-2 Hor-2 Sp-2 floatation one each	Gegham 1	2	2	2	?	216	6/30/23	705	4320	35	211	15	27	73	54	2.91	0.20	0.37	0.74	0.07	0.13	0.34	0.26
378	Tr-2 Hor-2 Sp-2 floatation one each	Kelbadjar	2	2	2	?	217	6/30/23	539	5537	40	164	17	9	114	33	1.44	0.14	0.08	0.29	0.10	0.05	0.69	0.20
379	Tr-2 Hor-2 Sp-2 floatation one each	Gegham 1	2	2	2	?	218	6/30/23	711	4402	39	216	17	22	72	54	3.02	0.23	0.30	0.75	0.08	0.10	0.33	0.25
380	Tr-2 Hor-2 Sp-2 floatation one each	Gegham 1	2	2	2	?	219	6/30/23	665	3662	33	204	13	25	70	53	2.93	0.18	0.35	0.75	0.06	0.12	0.34	0.26
381	Tr-2 Hor-2 Sp-2 floatation one each	Kelbadjar	2	2	2	?	220	6/30/23	541	5372	40	166	18	11	115	36	1.45	0.15	0.10	0.31	0.11	0.07	0.69	0.22
382	Tr-2 Hor-2 Sp-2 floatation one each	Gegham 1	2	2	2	?	221	6/30/23	662	3864	33	208	19	24	73	54	2.87	0.26	0.33	0.74	0.09	0.11	0.35	0.26
383	Tr-2 Hor-2 Sp-2 floatation one each	Gegham 1	2	2	2	?	222	6/30/23	774	4635	40	233	14	25	73	55	3.16	0.18	0.34	0.75	0.06	0.11	0.32	0.24
384	Tr-2 Hor-2 Sp-2 floatation one each	Gegham 1	2	2	2	?	223	6/30/23	781	4644	38	239	17	26	75	54	3.17	0.22	0.34	0.71	0.07	0.11	0.32	0.23
385	Tr-2 Hor-2 Sp-2 floatation one each	Gegham 1	2	2	2	?	224	6/30/23	752	4128	35	220	14	24	73	54	2.99	0.18	0.32	0.73	0.06	0.11	0.33	0.25
386	Tr-2 Hor-2 Sp-2 floatation one each	Gegham 1	2	2	2	?	225	6/30/23	711	4174	35	222	13	25	73	57	3.02	0.17	0.34	0.77	0.06	0.11	0.33	0.26
387	Tr-2 Hor-2 Sp-2 floatation one each	Gegham 1	2	2	2	?	226	6/30/23	752	4496	39	222	17	25	72	57	3.10	0.23	0.34	0.79	0.07	0.11	0.32	0.26
388	Tr-2 Hor-2 Sp-2 floatation one each	Gegham 1	2	2	2	?	228	6/30/23	751	4176	35	220	11	27	79	57	2.78	0.15	0.35	0.72	0.05	0.12	0.36	0.26
389	Trench-2 Hor-4 Spit-2 21Jun22 one each	Arteni	2	4	2	21-Jun	229	6/30/23	812	4028	46	156	15	36	66	43	2.36	0.22	0.55	0.65	0.09	0.23	0.42	0.28
390	Trench-2 Hor-4 Spit-2 21Jun22 one each	Kelbadjar	2	4	2	21-Jun	230	6/30/23	432	5009	38	168	24	11	100	35	1.68	0.24	0.11	0.35	0.14	0.07	0.59	0.21
391	Trench-2 Hor-4 Spit-2 21Jun22 one each	Gegham 1	2	4	2	21-Jun	231	6/30/23	690	4062	35	207	16	26	72	51	2.89	0.22	0.36	0.71	0.07	0.12	0.35	0.25
392	Trench-2 Hor-4 Spit-2 21Jun22 one each	Gegham 1	2	4	2	21-Jun	232	6/30/23	662	3973	34	211	13	27	74	54	2.84	0.17	0.36	0.72	0.06	0.13	0.35	0.26
393	Trench-2 Hor-4 Spit-2 21Jun22 one each	Gegham 1	2	4	2	21-Jun	233	6/30/23	665	3998	33	207	14	24	72	55	2.89	0.19	0.33	0.77	0.07	0.11	0.35	0.27
394	Trench-2 Hor-4 Spit-2 21Jun22 one each	Gegham 1	2	4	2	21-Jun	234	6/30/23	686	4542	36	215	17	26	73	54	2.93	0.22	0.35	0.73	0.08	0.12	0.34	0.25
395	Trench-2 Hor-4 Spit-2 21Jun22 one each	Gegham 1	2	4	2	21-Jun	235	6/30/23	705	3977	37	209	14	25	71	54	2.96	0.19	0.35	0.76	0.06	0.12	0.34	0.26
396	Trench-2 Hor-4 Spit-2 21Jun22 one each	Syunik	2	4	2	21-Jun	236	6/30/23	529	5668	40	191	21	9	102	36	1.87	0.20	0.08	0.35	0.11	0.05	0.53	0.19
397	Trench-2 Hor-4 Spit-2 21Jun22 one each	Gegham 1	2	4	2	21-Jun	237	6/30/23	758	4149	32	214	16	26	74	55	2.88	0.21	0.34	0.74	0.07	0.12	0.35	0.26
398	Trench-2 Hor-4 Spit-2 21Jun22 one each	Gegham 1	2	4	2	21-Jun	238	6/30/23	724	4100	34	222	13	26	72	54	3.10	0.17	0.36	0.75	0.06	0.12	0.32	0.24
399	Trench-2 Hor-4 Spit-2 21Jun22 one each	Gegham 1	2	4	2	21-Jun	239	6/30/23	717	3920	48	219	21	26	74	53	2.94	0.28	0.34	0.71	0.09	0.12	0.34	0.24
400	Trench-2 Hor-4 Spit-2 21Jun22 one each	Gegham 1	2	4	2	21-Jun	240	6/30/23	668	3643	35	207	16	25	72	54	2.89	0.22	0.34	0.75	0.07	0.12	0.35	0.26

401	Trench-2 Hor-4 Spit-2 21Jun22 one each	Gegham 1	2	4	2	21-Jun	241	6/30/23	668	4147	38	210	18	24	73	57	2.86	0.24	0.32	0.77	0.08	0.11	0.35	0.27
402	Trench-2 Hor-4 Spit-2 21Jun22 one each	Gegham 1	2	4	2	21-Jun	242	6/30/23	733	4312	35	226	15	27	74	57	3.03	0.19	0.36	0.76	0.06	0.12	0.33	0.25
403	Trench-2 Hor-4 Spit-2 21Jun22 one each	Gegham 1	2	4	2	21-Jun	243	6/30/23	685	4106	39	215	15	26	74	54	2.89	0.19	0.34	0.72	0.07	0.12	0.35	0.25
404	Trench-2 Hor-4 Spit-2 21Jun22 one each	Gegham 1	2	4	2	21-Jun	244	6/30/23	706	3985	32	212	14	26	73	54	2.92	0.19	0.35	0.74	0.06	0.12	0.34	0.25
405	Trench-2 Hor-4 Spit-2 21Jun22 one each	Gegham 1	2	4	2	21-Jun	245	6/30/23	697	4847	42	217	19	22	80	51	2.71	0.23	0.27	0.64	0.09	0.10	0.37	0.24
406	Trench-2 Hor-4 Spit-2 21Jun22 one each	Gegham 1	2	4	2	21-Jun	246	6/30/23	732	4743	41	225	18	23	73	53	3.10	0.24	0.31	0.72	0.08	0.10	0.32	0.23
407	Trench-2 Hor-4 Spit-2 21Jun22 one each	Gegham 1	2	4	2	21-Jun	247	6/30/23	840	4744	35	233	17	24	75	57	3.10	0.22	0.31	0.75	0.07	0.10	0.32	0.24
408	Trench-2 Hor-4 Spit-2 21Jun22 one each	Gegham 2?	2	4	2	21-Jun	248	6/30/23	837	7243	61	159	49	23	87	29	1.83	0.56	0.26	0.34	0.31	0.14	0.55	0.18
409	Trench-2 Hor-4 Spit-2 21Jun22 one each	Gegham 1	2	4	2	21-Jun	249	6/30/23	688	4327	35	216	15	26	74	53	2.90	0.19	0.34	0.71	0.07	0.12	0.34	0.24
410	Trench-2 Hor-4 Spit-2 21Jun22 one each	Gegham 1	2	4	2	21-Jun	250	6/30/23	882	5539	50	253	20	26	77	59	3.27	0.25	0.33	0.77	0.08	0.10	0.31	0.23
411	Trench-2 Hor-4 Spit-2 21Jun22 one each	Gegham 1	2	4	2	21-Jun	251	6/30/23	773	4365	38	226	16	27	76	57	2.96	0.20	0.35	0.74	0.07	0.12	0.34	0.25
412	Trench-2 Hor-4 Spit-2 21Jun22 one each	Gegham 1	2	4	2	21-Jun	252	6/30/23	661	4252	42	232	18	25	73	53	3.15	0.24	0.34	0.71	0.08	0.11	0.32	0.23
413	Trench-2 Hor-4 Spit-2 21Jun22 one each	Gegham 1	2	4	2	21-Jun	253	6/30/23	764	4374	37	230	14	25	77	58	2.97	0.17	0.32	0.75	0.06	0.11	0.34	0.25
414	Trench-2 Hor-4 Spit-2 21Jun22 one each	Gegham 1	2	4	2	21-Jun	254	6/30/23	726	4506	42	220	16	26	74	51	2.95	0.21	0.34	0.69	0.07	0.12	0.34	0.23
415	Trench-2 Hor-4 Spit-2 21Jun22 one each	Gegham 1	2	4	2	21-Jun	255	6/30/23	777	4305	35	222	14	26	73	54	3.06	0.19	0.35	0.74	0.06	0.12	0.33	0.24
416	Trench-2 Hor-4 Spit-2 21Jun22 one each	Gegham 1	2	4	2	21-Jun	256	6/30/23	779	4409	39	231	15	24	75	55	3.06	0.19	0.31	0.73	0.06	0.10	0.33	0.24
417	Trench-2 Hor-4 Spit-2 21Jun22 one each	Gegham 1	2	4	2	21-Jun	257	6/30/23	718	4626	38	207	19	24	82	50	2.53	0.23	0.29	0.61	0.09	0.11	0.40	0.24
418	Trench-2 Hor-4 Spit-2 21Jun22 one each	Gegham 1	2	4	2	21-Jun	258	6/30/23	792	4669	40	233	16	25	74	55	3.14	0.21	0.33	0.74	0.07	0.11	0.32	0.24
419	Trench-2 Hor-4 Spit-2 21Jun22 one each	Gegham 1	2	4	2	21-Jun	259	6/30/23	843	4730	41	233	14	27	75	57	3.08	0.18	0.35	0.75	0.06	0.11	0.32	0.24
420	Trench-2 Hor-4 Spit-2 21Jun22 one each	Gegham 1	2	4	2	21-Jun	260	6/30/23	806	4495	42	231	15	26	75	57	3.06	0.19	0.34	0.75	0.06	0.11	0.33	0.25
421	Trench-2 Hor-4 Spit-2 21Jun22 one each	Gegham 1	2	4	2	21-Jun	261	6/30/23	716	3996	35	217	14	24	73	57	2.99	0.19	0.33	0.78	0.06	0.11	0.33	0.26
422	Trench-2 Hor-4 Spit-2 21Jun22 one each	Gegham 1	2	4	2	21-Jun	262	6/30/23	839	4700	41	241	16	26	76	57	3.16	0.20	0.34	0.74	0.06	0.11	0.32	0.23
423	Trench-2 Hor-4 Spit-2 21Jun22 one each	Gegham 1	2	4	2	21-Jun	263	6/30/23	827	4778	44	234	17	27	74	58	3.15	0.22	0.36	0.78	0.07	0.11	0.32	0.25
424	Trench-2 Hor-4 Spit-2 21Jun22 one each	Gegham 1	2	4	2	21-Jun	264	6/30/23	768	4548	37	233	15	27	78	54	2.97	0.19	0.34	0.69	0.06	0.11	0.34	0.23
425	Trench-2 Hor-4 Spit-2 21Jun22 blade in own bag	Gegham 1	2	4	2	21-Jun	265	6/30/23	680	4557	38	215	15	22	71	51	3.04	0.21	0.31	0.72	0.07	0.10	0.33	0.24
427	smaller bag 12-JUN-22 Trench 1 Horizon 1 Spit.2 one each	Gegham 1	1	1	2	12-Jun	267	6/30/23	640	4224	36	211	18	25	73	53	2.87	0.24	0.34	0.71	0.08	0.12	0.35	0.25
428	smaller bag 12-JUN-22 Trench 1 Horizon 1 Spit.2 one each	Gegham 1	1	1	2	12-Jun	268	6/30/23	705	4280	38	213	15	26	72	53	2.98	0.20	0.36	0.73	0.07	0.12	0.34	0.25
429	smaller bag 12-JUN-22 Trench 1 Horizon 1 Spit.2 one each	Syunik	1	1	2	12-Jun	269	6/30/23	505	5342	36	180	21	11	108	36	1.66	0.19	0.10	0.33	0.11	0.06	0.60	0.20
430	smaller bag 12-JUN-22 Trench 1 Horizon 1 Spit.2 one each	Gegham 1	1	1	2	12-Jun	270	6/30/23	722	5322	37	212	14	26	72	53	2.96	0.19	0.36	0.73	0.06	0.12	0.34	0.25
431	smaller bag 12-JUN-22 Trench 1 Horizon 1 Spit.2 one each	Gegham 1	1	1	2	12-Jun	271	6/30/23	732	4155	35	215	14	26	72	55	3.00	0.19	0.36	0.77	0.06	0.12	0.33	0.26
432	smaller bag 12-JUN-22 Trench 1 Horizon 1 Spit.2 one each	Gegham 1	1	1	2	12-Jun	272	6/30/23	718	4232	38	218	13	26	73	57	3.00	0.17	0.35	0.78	0.06	0.12	0.33	0.26
433	smaller bag 12-JUN-22 Trench 1 Horizon 1 Spit.2 one each	Gegham 1	1	1	2	12-Jun	273	6/30/23	662	4216	38	212	18	24	73	54	2.89	0.24	0.32	0.73	0.08	0.11	0.35	0.25
434	smaller bag 12-JUN-22 Trench 1 Horizon 1 Spit.2 one each	Tsaghkunyats	1	1	2	12-Jun	274	6/30/23	503	7046	33	100	188	9	111	24	0.90	1.69	0.08	0.21	1.87	0.09	1.11	0.24
435	smaller bag 12-JUN-22 Trench 1 Horizon 1 Spit.2 one each	Gegham 1	1	1	2	12-Jun	275	6/30/23	683	3971	38	211	14	25	72	53	2.95	0.19	0.34	0.73	0.06	0.12	0.34	0.25
436	Tr-1 H-1 Sp-2 14-Jun-22 bag one each	Gegham 1	1	1	2	14-Jun	276	6/30/23	726	4094	34	218	13	27	77	55	2.82	0.16	0.34	0.71	0.06	0.12	0.35	0.25
437	Tr-1 H-1 Sp-2 14-Jun-22 bag one each	Arteni	1	1	2	14-Jun	277	6/30/23	549	4554	40	123	37	22	88	28	1.40	0.42	0.25	0.32	0.30	0.18	0.71	0.23
438	Tr-1 H-1 Sp-2 14-Jun-22 bag one each	Kelbadjar	1	1	2	14-Jun	279	6/30/23	510	4924	35	155	15	11	105	36	1.48	0.14	0.10	0.34	0.09	0.07	0.68	0.23
439	Tr-1 H-1 Sp-2 14-Jun-22 bag one each	Gegham 1	1	1	2	14-Jun	280	6/30/23	772	4199	35	220	14	24	73	57	2.99	0.18	0.32	0.77	0.06	0.11	0.33	0.26
440	Tr-1 H-1 Sp-2 14-Jun-22 bag one each	Gegham 1	1	1	2	14-Jun	281	6/30/23	722	3940	34	213	14	27	73	57	2.90	0.18	0.36	0.77	0.06	0.12	0.34	0.27
441	Tr-1 H-1 Sp-2 14-Jun-22 bag one each	Arteni	1	1	2	14-Jun	282	6/30/23	614	4137	41	142	24	29	85	33	1.68	0.28	0.35	0.39	0.17	0.21	0.60	0.23
442	Tr-1 H-1 Sp-2 14-Jun-22 bag one each	Gegham 1	1	1	2	14-Jun	283	6/30/23	710	4087	34	218	14	26	74	54	2.93	0.18	0.34	0.72	0.06	0.12	0.34	0.25
443	Tr-1 H-1 Sp-2 14-Jun-22 bag one each	Gegham 1	1	1	2	14-Jun	284	6/30/23	710	3952	33	214	14	25	73	51	2.91	0.18	0.34	0.70	0.06	0.12	0.34	0.24
444	Tr-1 H-1 Sp-2 14-Jun-22 bag one each	Arteni	1	1	2	14-Jun	285	6/30/23	635	4438	41	140	26	27	83	31	1.69	0.31	0.33	0.37	0.18	0.20	0.59	0.22
445	Tr-1 H-1 Sp-2 14-Jun-22 bag one each	Gegham 1	1	1	2	14-Jun	286	6/30/23	702	4059	37	219	13	24	73	55	3.02	0.17	0.33	0.76	0.06	0.11	0.33	0.25
446	Tr-1 H-1 Sp-2 14-Jun-22 bag one each	Gegham 1	1	1	2	14-Jun	287	6/30/23	670	3802	33	210	11	24	71	55	2.97	0.16	0.34	0.78	0.05	0.11	0.34	0.26
447	Tr-1 H-1 Sp-2 14-Jun-22 bag one each	Gegham 1	1	1	2	14-Jun	288	6/30/23	827	4828	45	239	16	23	77	53	3.10	0.20	0.29	0.68	0.06	0.10	0.32	0.22
448	Tr-1 H-1 Sp-2 14-Jun-22 bag one each	Gegham 1	1	1	2	14-Jun	289	6/30/23	706	4129	35	219	13	26	76	55	2.87	0.16	0.34	0.72	0.06	0.12	0.35	0.25
449	Tr-1 H-1 Sp-2 14-Jun-22 bag one each	Kelbadjar	1	1	2	14-Jun	290	6/30/23	540	5582	44	167	16	10	114	37	1.47	0.14	0.08	0.33	0.09	0.06	0.68	0.22
450	Tr-1 H-1 Sp-2 14-Jun-22 bag one each	Gegham 1	1	1	2	14-Jun	291	6/30/23	686	3785	32	211	11	27	73	57	2.87	0.16	0.36	0.77	0.05	0.13	0.35	0.27
451	Tr-1 H-1 Sp-2 14-Jun-22 bag one each	Gegham 1	1	1	2	14-Jun	292	6/30/23	814	4530	40	233	16	27	73	54	3.20	0.21	0.37	0.74	0.07	0.11	0.31	0.23
452	Tr-1 H-1 Sp-2 14-Jun-22 bag one each	Kelbadjar	1	1	2	14-Jun	293	6/30/23	567	5555	39	169	18	10	118	36	1.44	0.15	0.08	0.31	0.10	0.06	0.70	0.21

453	Tr-1 H-1 Sp-2 14-Jun-22 bag one each	Gegham 1	1	1	2	14-Jun	294	6/30/23	879	4701	43	248	16	26	73	57	3.38	0.21	0.35	0.77	0.06	0.10	0.30	0.23
454	Tr-1 H-1 Sp-2 14-Jun-22 bag one each	Gegham 1	1	1	2	14-Jun	295	6/30/23	698	4078	35	220	15	25	77	62	2.85	0.19	0.32	0.80	0.07	0.11	0.35	0.28
455	Tr-1 H-1 Sp-2 14-Jun-22 bag one each	Gegham 1	1	1	2	14-Jun	296	6/30/23	713	4492	38	220	18	24	74	53	2.95	0.24	0.32	0.71	0.08	0.11	0.34	0.24
456	Tr-1 H-1 Sp-2 14-Jun-22 bag one each	Kelbadjar	1	1	2	14-Jun	297	6/30/23	561	5514	42	170	19	11	108	33	1.57	0.17	0.11	0.31	0.11	0.07	0.64	0.20
457	Pit 1 10-JUN-22 TOP SOIL one each	Gegham 1	1	TS	-	10-Jun	298	6/30/23	661	3712	35	204	13	26	68	53	3.01	0.18	0.38	0.77	0.06	0.13	0.33	0.26
458	Pit 1 10-JUN-22 TOP SOIL one each	Syunik	1	TS	-	10-Jun	299	6/30/23	510	5322	36	177	24	12	108	33	1.65	0.22	0.12	0.31	0.13	0.07	0.61	0.19
459	Pit 1 10-JUN-22 TOP SOIL one each	Gegham 1	1	TS	-	10-Jun	300	6/30/23	728	4065	34	217	11	26	73	57	2.99	0.16	0.35	0.78	0.05	0.12	0.33	0.26
460	Pit 1 10-JUN-22 TOP SOIL one each	Gegham 1	1	TS	-	10-Jun	301	6/30/23	667	3780	36	205	13	26	72	54	2.87	0.17	0.36	0.75	0.06	0.12	0.35	0.26
461	Pit 1 10-JUN-22 TOP SOIL one each	Gegham 1	1	TS	-	10-Jun	302	6/30/23	627	3780	37	200	17	24	72	47	2.80	0.23	0.33	0.66	0.08	0.12	0.36	0.23
462	Pit 1 10-JUN-22 TOP SOIL one each	Gegham 1	1	TS	-	10-Jun	303	6/30/23	738	5282	46	211	24	24	72	57	2.95	0.33	0.33	0.79	0.11	0.11	0.34	0.27
463	Pit 1 10-JUN-22 TOP SOIL one each	Syunik	1	TS	-	10-Jun	304	6/30/23	570	5097	51	219	17	11	95	42	2.30	0.17	0.11	0.44	0.08	0.05	0.44	0.19
464	Pit 1 10-JUN-22 TOP SOIL one each	Gegham 1	1	TS	-	10-Jun	305	6/30/23	652	3705	33	202	11	24	73	55	2.79	0.16	0.33	0.76	0.06	0.12	0.36	0.27
465	Pit 1 10-JUN-22 TOP SOIL one each	Gegham 1	1	TS	-	10-Jun	306	6/30/23	697	3921	37	215	13	26	73	53	2.93	0.17	0.35	0.71	0.06	0.12	0.34	0.24
466	Trench 2 17-JUN-22 Horizon 2 Spit 2 one each	Gegham 1	2	2	2	17-Jun	307	6/30/23	647	3704	33	203	14	25	69	55	2.96	0.20	0.36	0.80	0.07	0.12	0.34	0.27
467	Trench 2 17-JUN-22 Horizon 2 Spit 2 one each	Gegham 1	2	2	2	17-Jun	308	6/30/23	651	3684	32	202	13	26	73	55	2.79	0.17	0.35	0.76	0.06	0.13	0.36	0.27
468	Trench 2 17-JUN-22 Horizon 2 Spit 2 one each	Gegham 1	2	2	2	17-Jun	309	6/30/23	707	3971	35	213	14	26	74	55	2.86	0.18	0.34	0.74	0.06	0.12	0.35	0.26
469	Trench 2 17-JUN-22 Horizon 2 Spit 2 one each	Gegham 1	2	2	2	17-Jun	310	6/30/23	698	3853	33	211	13	22	70	51	3.03	0.18	0.31	0.73	0.06	0.10	0.33	0.24
470	Trench 2 17-JUN-22 Horizon 2 Spit 2 one each	Syunik	2	2	2	17-Jun	311	6/30/23	503	5328	35	184	21	11	105	35	1.76	0.20	0.11	0.33	0.11	0.06	0.57	0.19
471	Trench 2 17-JUN-22 Horizon 2 Spit 2 one each	Gegham 1	2	2	2	17-Jun	312	6/30/23	672	3907	31	209	14	26	71	51	2.96	0.19	0.36	0.72	0.06	0.12	0.34	0.24
472	Trench 2 17-JUN-22 Horizon 2 Spit 2 one each	Gegham 1	2	2	2	17-Jun	313	6/30/23	734	4002	35	212	14	25	73	57	2.89	0.18	0.34	0.77	0.06	0.12	0.35	0.27
473	Trench 2 17-JUN-22 Horizon 2 Spit 2 one each	Gegham 1	2	2	2	17-Jun	314	6/30/23	715	4114	36	214	14	24	71	57	3.03	0.19	0.34	0.80	0.06	0.11	0.33	0.26
474	Trench 2 17-JUN-22 Horizon 2 Spit 2 one each	Gegham 1	2	2	2	17-Jun	315	6/30/23	680	4220	35	207	15	26	75	54	2.75	0.19	0.34	0.71	0.07	0.12	0.36	0.26
475	Trench 2 17-JUN-22 Horizon 2 Spit 2 one each	Gegham 1	2	2	2	17-Jun	316	6/30/23	706	3976	33	216	13	24	71	57	3.06	0.18	0.34	0.80	0.06	0.11	0.33	0.26
476	Trench 2 17-JUN-22 Horizon 2 Spit 2 one each	Gegham 1	2	2	2	17-Jun	317	6/30/23	676	3670	33	204	14	27	71	54	2.89	0.19	0.38	0.76	0.07	0.13	0.35	0.26
477	Trench 2 17-JUN-22 Horizon 2 Spit 2 one each	Gegham 1	2	2	2	17-Jun	318	6/30/23	733	4247	35	226	15	25	73	58	3.07	0.20	0.34	0.79	0.06	0.11	0.33	0.26
478	Trench 2 17-JUN-22 Horizon 2 Spit 2 one each	Gegham 1	2	2	2	17-Jun	319	6/30/23	702	4097	31	213	15	26	73	54	2.90	0.20	0.35	0.73	0.07	0.12	0.34	0.25
479	Trench 2 17-JUN-22 Horizon 2 Spit 2 one each	Syunik	2	2	2	17-Jun	320	6/30/23	499	5611	40	188	24	10	106	35	1.78	0.22	0.09	0.33	0.13	0.05	0.56	0.18
480	Trench 2 17-JUN-22 Horizon 2 Spit 2 one each	Gegham 1	2	2	2	17-Jun	321	6/30/23	676	3894	27	214	11	25	73	57	2.91	0.16	0.34	0.77	0.05	0.12	0.34	0.26
481	Trench 2 17-JUN-22 Horizon 2 Spit 2 one each	Syunik	2	2	2	17-Jun	322	6/30/23	465	5331	38	190	21	13	103	35	1.85	0.20	0.13	0.34	0.11	0.07	0.54	0.18
482	Trench 2 17-JUN-22 Horizon 2 Spit 2 one each	Gegham 1	2	2	2	17-Jun	323	6/30/23	702	4124	34	223	15	26	76	55	2.92	0.19	0.34	0.72	0.07	0.11	0.34	0.25
483	Trench 2 17-JUN-22 Horizon 2 Spit 2 one each	Arteni	2	2	2	17-Jun	324	6/30/23	589	5012	43	134	38	25	90	29	1.50	0.42	0.28	0.33	0.28	0.18	0.67	0.22
484	Trench 2 17-JUN-22 Horizon 2 Spit 2 one each	Gegham 1	2	2	2	17-Jun	325	6/30/23	762	4438	37	230	15	25	73	57	3.12	0.20	0.34	0.77	0.06	0.11	0.32	0.25
485	Trench 2 17-JUN-22 Horizon 2 Spit 2 one each	Gegham 1	2	2	2	17-Jun	326	6/30/23	798	4492	38	235	15	26	74	55	3.16	0.19	0.34	0.74	0.06	0.11	0.32	0.23
486	Trench 2 17-JUN-22 Horizon 2 Spit 2 one each	Gegham 1	2	2	2	17-Jun	327	6/30/23	695	3954	33	209	14	26	74	53	2.81	0.18	0.34	0.71	0.06	0.12	0.36	0.25
487	Trench 2 17-JUN-22 Horizon 2 Spit 2 one each	Syunik	2	2	2	17-Jun	328	6/30/23	479	5017	35	180	20	11	106	36	1.70	0.18	0.11	0.34	0.11	0.06	0.59	0.20
488	Trench 2 17-JUN-22 Horizon 2 Spit 2 one each	Gegham 1	2	2	2	17-Jun	329	6/30/23	713	4068	32	217	14	24	75	55	2.88	0.18	0.31	0.73	0.06	0.11	0.35	0.25
489	Trench 2 17-JUN-22 Horizon 2 Spit 2 one each	Arteni	2	2	2	17-Jun	330	6/30/23	617	5184	45	130	43	27	94	28	1.37	0.45	0.28	0.29	0.33	0.20	0.73	0.21
490	Trench 2 17-JUN-22 Horizon 2 Spit 2 one each	Arteni	2	2	2	17-Jun	331	6/30/23	596	4442	35	129	32	25	81	29	1.59	0.39	0.30	0.36	0.25	0.19	0.63	0.23
491	Trench 2 17-JUN-22 Horizon 2 Spit 2 one each	Gegham 1	2	2	2	17-Jun	332	6/30/23	744	4352	39	220	14	24	77	57	2.85	0.17	0.31	0.73	0.06	0.11	0.35	0.26
492	Trench 2 17-JUN-22 Horizon 2 Spit 2 one each	Gegham 1	2	2	2	17-Jun	333	6/30/23	723	3889	36	220	15	25	71	54	3.11	0.21	0.35	0.76	0.07	0.11	0.32	0.25
493	Trench 2 17-JUN-22 Horizon 2 Spit 2 one each	Gegham 1	2	2	2	17-Jun	334	6/30/23	724	4108	33	218	13	25	76	57	2.86	0.16	0.32	0.74	0.06	0.11	0.35	0.26
494	Trench 2 17-JUN-22 Horizon 2 Spit 2 one each	Gegham 1	2	2	2	17-Jun	335	6/30/23	751	4339	61	224	14	26	81	59	2.76	0.17	0.32	0.73	0.06	0.11	0.36	0.27
495	Trench 2 17-JUN-22 Horizon 2 Spit 2 one each	Syunik	2	2	2	17-Jun	336	6/30/23	454	5263	35	178	24	11	106	36	1.69	0.22	0.10	0.34	0.13	0.06	0.59	0.20
496	Trench 2 17-JUN-22 Horizon 2 Spit 2 one each	Gegham 1	2	2	2	17-Jun	337	6/30/23	663	4138	53	200	17	27	73	58	2.76	0.23	0.37	0.80	0.08	0.13	0.36	0.29
497	Trench 2 17-JUN-22 Horizon 2 Spit 2 one each	Gegham 1	2	2	2	17-Jun	338	6/30/23	653	3953	34	213	15	26	73	57	2.94	0.20	0.35	0.78	0.07	0.12	0.34	0.27
498	Trench 2 17-JUN-22 Horizon 2 Spit 2 one each	Syunik	2	2	2	17-Jun	339	6/30/23	523	5348	41	192	23	11	104	37	1.85	0.22	0.10	0.36	0.12	0.05	0.54	0.20
499	Trench 2 17-JUN-22 Horizon 2 Spit 2 one each	Gegham 1	2	2	2	17-Jun	340	6/30/23	726	4412	36	228	14	27	76	57	2.98	0.18	0.35	0.74	0.06	0.12	0.34	0.25
500	Trench 2 17-JUN-22 Horizon 2 Spit 2 one each	Kelbadjar	2	2	2	17-Jun	341	6/30/23	586	5770	45	174	19	11	125	36	1.40	0.15	0.08	0.29	0.11	0.06	0.71	0.21
501	Trench 2 17-JUN-22 Horizon 2 Spit 2 one each	Gegham 1	2	2	2	17-Jun	342	6/30/23	762	4404	35	234	14	24	74	57	3.15	0.18	0.32	0.76	0.06	0.10	0.32	0.24
502	Trench 2 17-JUN-22 Horizon 2 Spit 2 one each	Gegham 1	2	2	2	17-Jun	343	6/30/23	753	4303	35	226	14	25	73	53	3.07	0.18	0.34	0.71	0.06	0.11	0.33	0.23
503	Trench 2 17-JUN-22 Horizon 2 Spit 2 one each	Gegham 1	2	2	2	17-Jun	344	6/30/23	779	4241	35	229	14	24	73	55	3.11	0.18	0.32	0.75	0.06	0.10	0.32	0.24

504	Trench 2 17-JUN-22 Horizon 2 Spit 2 one each	Gegham 1	2	2	2	17-Jun	345	6/30/23	757	4411	38	224	14	27	73	55	3.04	0.18	0.36	0.75	0.06	0.12	0.33	0.25
505	Trench 2 17-JUN-22 Horizon 2 Spit 2 one each	Gegham 1	2	2	2	17-Jun	346	6/30/23	884	5064	46	252	15	24	76	57	3.30	0.19	0.31	0.74	0.06	0.09	0.30	0.22
506	Trench 2 17-JUN-22 Horizon 2 Spit 2 one each	Syunik	2	2	2	17-Jun	347	6/30/23	667	6785	49	192	19	11	120	33	1.60	0.15	0.10	0.28	0.10	0.06	0.63	0.17
507	Trench 2 17-JUN-22 Horizon 2 Spit 2 one each	Gegham 1	2	2	2	17-Jun	349	6/30/23	758	4196	38	227	14	27	77	58	2.93	0.17	0.34	0.75	0.06	0.12	0.34	0.26
508	Trench 2 17-JUN-22 Horizon 2 Spit 2 one each	Gegham 1	2	2	2	17-Jun	350	6/30/23	751	4124	35	219	14	27	75	57	2.90	0.18	0.36	0.75	0.06	0.13	0.34	0.26
509	Trench 2 17-JUN-22 Horizon 2 Spit 2 one each	Gegham 1	2	2	2	17-Jun	351	6/30/23	820	5210	44	249	22	25	76	61	3.26	0.28	0.32	0.80	0.09	0.10	0.31	0.24
510	Tr-2 Hor-3 Sp-1 floatation one each	Gegham 1	2	3	1	?	352	6/30/23	671	4149	36	205	16	22	73	50	2.79	0.21	0.30	0.68	0.08	0.11	0.36	0.24
511	Tr-2 Hor-3 Sp-1 floatation one each	Kelbadjar	2	3	1	?	353	6/30/23	484	5012	63	164	17	11	111	33	1.47	0.15	0.10	0.30	0.10	0.07	0.68	0.20
512	Tr-2 Hor-3 Sp-1 floatation one each	Gegham 1	2	3	1	?	354	6/30/23	717	3908	33	210	15	26	71	58	2.97	0.21	0.36	0.82	0.07	0.12	0.34	0.28
513	Tr-2 Hor-3 Sp-1 floatation one each	Kelbadjar	2	3	1	?	355	6/30/23	545	5695	41	171	17	12	118	37	1.45	0.14	0.11	0.32	0.10	0.07	0.69	0.22
514	Tr-2 Hor-3 Sp-1 floatation one each	Gegham 1	2	3	1	?	356	6/30/23	849	4691	41	249	16	25	74	58	3.35	0.21	0.33	0.78	0.06	0.10	0.30	0.23
515	Tr-2 Hor-3 Sp-1 floatation one each	Gegham 1	2	3	1	?	357	6/30/23	855	4979	45	236	15	29	82	55	2.88	0.18	0.36	0.67	0.06	0.12	0.35	0.23
516	Tr-2 Hor-3 Sp-1 floatation one each	Gegham 1	2	3	1	?	358	6/30/23	914	4863	43	250	16	24	72	53	3.49	0.22	0.33	0.73	0.06	0.09	0.29	0.21
517	Trench-2 Horizon 3 Spit 1 18Jun22 one each	Kelbadjar	2	3	1	18-Jun	371	6/30/23	469	7220	78	176	25	11	104	31	1.70	0.24	0.11	0.29	0.14	0.07	0.59	0.17
518	Trench-2 Horizon 3 Spit 1 18Jun22 one each	Gegham 1	2	3	1	18-Jun	372	6/30/23	701	4048	33	211	13	27	72	55	2.95	0.17	0.37	0.77	0.06	0.13	0.34	0.26
519	Trench-2 Horizon 3 Spit 1 18Jun22 one each	Kelbadjar	2	3	1	18-Jun	373	6/30/23	505	5453	43	164	17	11	108	33	1.51	0.15	0.11	0.31	0.10	0.07	0.66	0.20
520	Trench-2 Horizon 3 Spit 1 18Jun22 one each	Gegham 1	2	3	1	18-Jun	374	6/30/23	741	4116	42	222	14	26	73	57	3.02	0.18	0.35	0.77	0.06	0.12	0.33	0.26
521	Trench-2 Horizon 3 Spit 1 18Jun22 one each	Gegham 1	2	3	1	18-Jun	375	6/30/23	670	3944	34	204	13	25	73	57	2.82	0.17	0.34	0.78	0.06	0.12	0.36	0.28
522	Trench-2 Horizon 3 Spit 1 18Jun22 one each	Gegham 1	2	3	1	18-Jun	376	6/30/23	783	4479	37	225	15	25	73	57	3.06	0.20	0.34	0.77	0.06	0.11	0.33	0.25
523	Trench-2 Horizon 3 Spit 1 18Jun22 one each	Gegham 1	2	3	1	18-Jun	377	6/30/23	773	4311	38	219	15	23	73	53	3.02	0.20	0.31	0.72	0.07	0.10	0.33	0.24
524	Trench-2 Horizon 3 Spit 1 18Jun22 one each	Gegham 1	2	3	1	18-Jun	378	6/30/23	743	4077	35	218	14	23	72	55	3.04	0.19	0.32	0.77	0.06	0.10	0.33	0.25
525	Trench-2 Horizon 3 Spit 1 18Jun22 one each	Gegham 1	2	3	1	18-Jun	379	6/30/23	680	3796	34	207	13	27	73	54	2.86	0.17	0.37	0.74	0.06	0.13	0.35	0.26
526	Trench-2 Horizon 3 Spit 1 18Jun22 one each	Gegham 1	2	3	1	18-Jun	380	6/30/23	656	4140	33	202	17	26	77	48	2.62	0.21	0.33	0.63	0.08	0.13	0.38	0.24
527	Trench-2 Horizon 3 Spit 1 18Jun22 one each	Gegham 1	2	3	1	18-Jun	381	6/30/23	681	3988	32	210	15	27	71	53	2.97	0.21	0.38	0.74	0.07	0.13	0.34	0.25
528	Trench-2 Horizon 3 Spit 1 18Jun22 one each	Gegham 1	2	3	1	18-Jun	382	6/30/23	617	3797	41	204	20	25	75	55	2.71	0.26	0.33	0.73	0.10	0.12	0.37	0.27
529	Trench-2 Horizon 3 Spit 1 18Jun22 one each	Gegham 1	2	3	1	18-Jun	383	6/30/23	700	3919	32	210	13	27	75	55	2.79	0.17	0.35	0.73	0.06	0.13	0.36	0.26
530	Trench-2 Horizon 3 Spit 1 18Jun22 one each	Gegham 2	2	3	1	18-Jun	384	6/30/23	661	5391	44	127	47	27	97	29	1.30	0.48	0.27	0.30	0.37	0.21	0.77	0.23
531	Trench-2 Horizon 3 Spit 1 18Jun22 one each	Gegham 1	2	3	1	18-Jun	385	6/30/23	756	4400	35	223	15	26	74	54	2.99	0.19	0.34	0.72	0.07	0.11	0.33	0.24
532	Trench-2 Horizon 3 Spit 1 18Jun22 one each	Gegham 1	2	3	1	18-Jun	386	6/30/23	697	4034	35	214	14	25	73	58	2.91	0.18	0.34	0.79	0.06	0.12	0.34	0.27
533	Trench-2 Horizon 3 Spit 1 18Jun22 one each	Gegham 1	2	3	1	18-Jun	387	6/30/23	749	4309	36	230	14	25	76	54	3.01	0.18	0.32	0.71	0.06	0.11	0.33	0.23
534	Trench-2 Horizon 3 Spit 1 18Jun22 one each	Arteni	2	3	1	18-Jun	388	6/30/23	661	4861	44	134	38	25	89	25	1.52	0.42	0.28	0.28	0.28	0.18	0.66	0.19
535	Trench-2 Horizon 3 Spit 1 18Jun22 one each	Gegham 1	2	3	1	18-Jun	389	6/30/23	650	4106	33	204	17	22	73	54	2.82	0.23	0.30	0.74	0.08	0.11	0.36	0.26
536	Trench-2 Horizon 3 Spit 1 18Jun22 one each	Gegham 1	2	3	1	18-Jun	391	6/30/23	702	4065	35	218	13	27	73	54	2.97	0.17	0.36	0.73	0.06	0.12	0.34	0.25
537	Trench-2 Horizon 3 Spit 1 18Jun22 one each	Gegham 1	2	3	1	18-Jun	392	6/30/23	672	3817	33	214	18	26	74	57	2.88	0.24	0.34	0.76	0.08	0.12	0.35	0.26
538	Trench-2 Horizon 3 Spit 1 18Jun22 one each	Gegham 1	2	3	1	18-Jun	393	6/30/23	727	4467	41	216	19	26	75	57	2.87	0.25	0.34	0.75	0.09	0.12	0.35	0.26
539	Trench-2 Horizon 3 Spit 1 18Jun22 one each	Gegham 1	2	3	1	18-Jun	394	6/30/23	698	3730	35	214	13	26	73	54	2.95	0.17	0.35	0.74	0.06	0.12	0.34	0.25
540	Trench-2 Horizon 3 Spit 1 18Jun22 one each	Gegham 1	2	3	1	18-Jun	395	6/30/23	808	4523	40	234	14	25	75	57	3.11	0.18	0.33	0.75	0.06	0.11	0.32	0.24
541	Trench-2 Horizon 3 Spit 1 18Jun22 one each	Gegham 1	2	3	1	18-Jun	396	6/30/23	702	3969	37	218	13	27	73	55	2.97	0.17	0.36	0.75	0.06	0.12	0.34	0.25
542	Trench-2 Horizon 3 Spit 1 18Jun22 one each	Arteni	2	3	1	18-Jun	397	6/30/23	691	4301	41	143	20	30	81	35	1.77	0.24	0.37	0.43	0.14	0.21	0.57	0.24
543	Trench-2 Horizon 3 Spit 1 18Jun22 one each	Gegham 1	2	3	1	18-Jun	398	6/30/23	711	4013	36	223	14	26	75	54	2.96	0.18	0.34	0.71	0.06	0.11	0.34	0.24
544	Trench-2 Horizon 3 Spit 1 18Jun22 one each	Arteni	2	3	1	18-Jun	399	6/30/23	724	4884	45	147	29	27	85	31	1.73	0.34	0.31	0.36	0.19	0.18	0.58	0.21
545	Trench-2 Horizon 3 Spit 1 18Jun22 one each	Gegham 1	2	3	1	18-Jun	400	6/30/23	809	4709	42	241	15	25	77	59	3.12	0.19	0.32	0.77	0.06	0.10	0.32	0.25
546	Trench-2 Horizon 3 Spit 1 18Jun22 one each	Arteni	2	3	1	18-Jun	401	6/30/23	643	4654	40	141	33	27	85	29	1.66	0.38	0.32	0.34	0.23	0.19	0.60	0.21
547	Trench-2 Horizon 3 Spit 1 18Jun22 one each	Gegham 1	2	3	1	18-Jun	402	6/30/23	1110	6138	58	272	16	26	73	54	3.71	0.21	0.35	0.73	0.06	0.09	0.27	0.20
548	Trench-2 Horizon 3 Spit 1 18Jun22 one each	Gegham 1	2	3	1	18-Jun	403	6/30/23	761	4340	43	230	14	24	73	55	3.12	0.18	0.32	0.75	0.06	0.10	0.32	0.24
549	Trench-2 Horizon 3 Spit 1 18Jun22 one each	Gegham 1	2	3	1	18-Jun	404	6/30/23	712	3863	35	215	14	27	73	55	2.93	0.18	0.36	0.75	0.06	0.12	0.34	0.26
550	Trench 2 Top Soil 13-JUN-22 sample of 60 one each	Gegham 1	2	TS	-	13-Jun	483	6/30/23	697	4084	38	217	14	25	72	55	3.03	0.19	0.34	0.77	0.06	0.11	0.33	0.25
551	Trench 2 Top Soil 13-JUN-22 sample of 60 one each	Gegham 1	2	TS	-	13-Jun	484	6/30/23	643	3923	30	207	13	23	69	54	3.01	0.18	0.33	0.78	0.06	0.11	0.33	0.26
552	Trench 2 Top Soil 13-JUN-22 sample of 60 one each	Gegham 1	2	TS	-	13-Jun	485	6/30/23	741	4023	33	208	11	27	72	55	2.91	0.16	0.37	0.77	0.06	0.13	0.34	0.27
553	Trench 2 Top Soil 13-JUN-22 sample of 60 one each	Gegham 1	2	TS	-	13-Jun	486	6/30/23	693	3922	35	215	14	27	73	55	2.93	0.18	0.36	0.75	0.06	0.12	0.34	0.26
554	Trench 2 Top Soil 13-JUN-22 sample of 60 one each	Gegham 1	2	TS	-	13-Jun	487	6/30/23	633	3919	37	208	14	23	72	55	2.91	0.19	0.32	0.77	0.06	0.11	0.34	0.27

555	Trench 2 Top Soil 13-JUN-22 sample of 60 one each	Gegham 1	2	TS	-	13-Jun	488	6/30/23	686	3905	35	212	13	27	72	51	2.96	0.17	0.38	0.71	0.06	0.13	0.34	0.24
556	Trench 2 Top Soil 13-JUN-22 sample of 60 one each	Gegham 1	2	TS	-	13-Jun	489	6/30/23	723	4292	35	222	14	25	74	55	2.98	0.18	0.33	0.74	0.06	0.11	0.34	0.25
557	Trench 2 Top Soil 13-JUN-22 sample of 60 one each	Gegham 1	2	TS	-	13-Jun	490	6/30/23	672	3927	36	217	11	25	73	53	2.99	0.16	0.34	0.72	0.05	0.11	0.33	0.24
558	Trench 2 Top Soil 13-JUN-22 sample of 60 one each	Gegham 1	2	TS	-	13-Jun	491	6/30/23	715	4169	36	215	15	25	74	53	2.89	0.19	0.33	0.71	0.07	0.11	0.35	0.24
559	Trench 2 Top Soil 13-JUN-22 sample of 60 one each	Gegham 1	2	TS	-	13-Jun	492	6/30/23	716	4060	35	214	13	25	77	59	2.77	0.16	0.32	0.77	0.06	0.12	0.36	0.28
560	Trench 2 Top Soil 13-JUN-22 sample of 60 one each	Gegham 1	2	TS	-	13-Jun	493	6/30/23	734	4488	39	217	18	23	79	51	2.74	0.22	0.29	0.65	0.08	0.10	0.36	0.24
561	Trench 2 Top Soil 13-JUN-22 sample of 60 one each	Kelbadjar	2	TS	-	13-Jun	494	6/30/23	524	5309	43	160	17	11	107	32	1.50	0.15	0.11	0.30	0.10	0.07	0.67	0.20
562	Trench 2 Top Soil 13-JUN-22 sample of 60 one each	Gegham 1	2	TS	-	13-Jun	495	6/30/23	722	4050	31	217	13	27	74	55	2.91	0.17	0.36	0.74	0.06	0.12	0.34	0.25
563	Trench 2 Top Soil 13-JUN-22 sample of 60 one each	Kelbadjar	2	TS	-	13-Jun	496	6/30/23	485	5147	40	153	19	12	108	36	1.42	0.17	0.12	0.34	0.12	0.08	0.70	0.24
564	Trench 2 Top Soil 13-JUN-22 sample of 60 one each	Gegham 1	2	TS	-	13-Jun	497	6/30/23	698	3996	34	215	13	27	74	54	2.89	0.17	0.37	0.72	0.06	0.13	0.35	0.25
565	Trench 2 Top Soil 13-JUN-22 sample of 60 one each	Syunik	2	TS	-	13-Jun	498	6/30/23	501	5146	37	181	22	11	108	37	1.68	0.20	0.11	0.35	0.12	0.06	0.59	0.21
566	Trench 2 Top Soil 13-JUN-22 sample of 60 one each	Kelbadjar	2	TS	-	13-Jun	499	6/30/23	526	5294	38	160	16	11	112	37	1.42	0.14	0.10	0.33	0.10	0.07	0.70	0.23
567	Trench 2 Top Soil 13-JUN-22 sample of 60 one each	Gegham 1	2	TS	-	13-Jun	500	6/30/23	667	3894	32	206	13	23	72	54	2.88	0.17	0.32	0.75	0.06	0.11	0.35	0.26
568	Trench 2 Top Soil 13-JUN-22 sample of 60 one each	Gegham 1	2	TS	-	13-Jun	501	6/30/23	706	3839	32	206	11	22	70	54	2.96	0.16	0.31	0.77	0.06	0.11	0.34	0.26
569	Trench 2 Top Soil 13-JUN-22 sample of 60 one each	Kelbadjar	2	TS	-	13-Jun	502	6/30/23	524	5320	40	159	17	10	109	37	1.45	0.15	0.09	0.34	0.10	0.06	0.69	0.24
570	Trench 2 Top Soil 13-JUN-22 sample of 60 one each	Gegham 1	2	TS	-	13-Jun	503	6/30/23	734	4236	33	221	14	25	77	57	2.86	0.17	0.32	0.73	0.06	0.11	0.35	0.26
571	Trench 2 Top Soil 13-JUN-22 sample of 60 one each	Gegham 1	2	TS	-	13-Jun	504	6/30/23	686	3851	35	216	13	25	77	54	2.80	0.16	0.32	0.70	0.06	0.11	0.36	0.25
572	Trench 2 Top Soil 13-JUN-22 sample of 60 one each	Kelbadjar	2	TS	-	13-Jun	505	6/30/23	544	5606	45	173	18	11	116	37	1.49	0.15	0.10	0.32	0.10	0.07	0.67	0.22
573	Trench 2 Top Soil 13-JUN-22 sample of 60 one each	Kelbadjar	2	TS	-	13-Jun	506	6/30/23	523	5246	39	162	16	11	120	36	1.35	0.13	0.09	0.30	0.10	0.07	0.74	0.22
574	Trench 2 Top Soil 13-JUN-22 sample of 60 one each	Gegham 1	2	TS	-	13-Jun	507	6/30/23	576	3778	44	200	18	24	73	51	2.73	0.24	0.32	0.70	0.09	0.12	0.37	0.26
575	Trench 2 Top Soil 13-JUN-22 sample of 60 one each	Gegham 1	2	TS	-	13-Jun	508	6/30/23	636	3788	35	196	15	24	73	51	2.66	0.20	0.32	0.70	0.07	0.12	0.38	0.26
576	Trench 2 Top Soil 13-JUN-22 sample of 60 one each	Gegham 1	2	TS	-	13-Jun	509	6/30/23	717	4188	34	222	14	25	73	53	3.06	0.19	0.34	0.72	0.06	0.11	0.33	0.24
577	Trench 2 Top Soil 13-JUN-22 sample of 60 one each	Gegham 1	2	TS	-	13-Jun	510	6/30/23	782	4271	35	227	14	25	75	55	3.01	0.18	0.33	0.73	0.06	0.11	0.33	0.24
578	Trench 2 Top Soil 13-JUN-22 sample of 60 one each	Gegham 1	2	TS	-	13-Jun	511	6/30/23	727	4100	39	216	16	27	74	57	2.90	0.21	0.36	0.76	0.07	0.12	0.34	0.26
579	Trench 2 Top Soil 13-JUN-22 sample of 60 one each	Gegham 1	2	TS	-	13-Jun	512	6/30/23	640	4277	37	208	23	26	73	51	2.87	0.31	0.35	0.70	0.11	0.12	0.35	0.25
580	Trench 2 Top Soil 13-JUN-22 sample of 60 one each	Gegham 1	2	TS	-	13-Jun	513	6/30/23	757	4101	33	221	13	27	81	54	2.72	0.15	0.34	0.66	0.06	0.12	0.37	0.24
581	Trench 2 Top Soil 13-JUN-22 sample of 60 one each	Gegham 1	2	TS	-	13-Jun	514	6/30/23	715	4062	35	216	14	29	78	58	2.76	0.17	0.38	0.74	0.06	0.14	0.36	0.27
582	Trench 2 Top Soil 13-JUN-22 sample of 60 one each	Gegham 1	2	TS	-	13-Jun	515	6/30/23	788	4324	36	224	14	27	77	59	2.90	0.17	0.34	0.77	0.06	0.12	0.35	0.27
583	Trench 2 Top Soil 13-JUN-22 sample of 60 one each	Kelbadjar	2	TS	-	13-Jun	516	6/30/23	471	5048	40	159	17	11	117	36	1.36	0.14	0.09	0.31	0.10	0.07	0.74	0.23
584	Trench 2 Top Soil 13-JUN-22 sample of 60 one each	Kelbadjar	2	TS	-	13-Jun	517	6/30/23	537	5401	41	165	18	11	110	36	1.50	0.16	0.10	0.33	0.11	0.07	0.67	0.22
585	Trench 2 Top Soil 13-JUN-22 sample of 60 one each	Gegham 1	2	TS	-	13-Jun	518	6/30/23	702	3835	33	211	13	27	73	53	2.87	0.17	0.36	0.71	0.06	0.13	0.35	0.25
586	Trench 2 Top Soil 13-JUN-22 sample of 60 one each	Gegham 1	2	TS	-	13-Jun	519	6/30/23	721	4388	45	209	16	27	73	55	2.85	0.21	0.36	0.75	0.07	0.13	0.35	0.26
587	Trench 2 Top Soil 13-JUN-22 sample of 60 one each	Gegham 1	2	TS	-	13-Jun	520	6/30/23	711	4079	35	221	14	27	73	55	3.04	0.19	0.37	0.76	0.06	0.12	0.33	0.25
588	Trench 2 Top Soil 13-JUN-22 sample of 60 one each	Gegham 1	2	TS	-	13-Jun	521	6/30/23	673	3844	34	210	14	25	72	55	2.94	0.19	0.34	0.77	0.06	0.12	0.34	0.26
589	Trench 2 Top Soil 13-JUN-22 sample of 60 one each	Kelbadjar	2	TS	-	13-Jun	522	6/30/23	503	5062	40	161	16	13	118	35	1.36	0.13	0.11	0.29	0.10	0.08	0.73	0.22
590	Trench 2 Top Soil 13-JUN-22 sample of 60 one each	Kelbadjar	2	TS	-	13-Jun	523	6/30/23	610	6135	49	183	17	11	114	35	1.60	0.14	0.10	0.30	0.09	0.06	0.62	0.19
591	Trench 2 Top Soil 13-JUN-22 sample of 60 one each	Gegham 1	2	TS	-	13-Jun	524	6/30/23	681	3865	32	215	13	26	73	57	2.96	0.17	0.35	0.78	0.06	0.12	0.34	0.26
592	Trench 2 Top Soil 13-JUN-22 sample of 60 one each	Gegham 1	2	TS	-	13-Jun	525	6/30/23	748	4200	38	226	13	25	73	57	3.07	0.17	0.34	0.77	0.06	0.11	0.33	0.25
593	Trench 2 Top Soil 13-JUN-22 sample of 60 one each	Gegham 1	2	TS	-	13-Jun	526	6/30/23	771	4228	40	228	14	26	74	55	3.06	0.18	0.34	0.74	0.06	0.11	0.33	0.24
594	Trench 2 Top Soil 13-JUN-22 sample of 60 one each	Kelbadjar	2	TS	-	13-Jun	527	6/30/23	499	4991	39	163	18	11	125	33	1.31	0.14	0.09	0.27	0.11	0.07	0.77	0.20
595	Trench 2 Top Soil 13-JUN-22 sample of 60 one each	Kelbadjar	2	TS	-	13-Jun	528	6/30/23	596	5963	48	166	22	11	113	36	1.47	0.19	0.09	0.32	0.13	0.06	0.68	0.22
596	Trench 2 Top Soil 13-JUN-22 sample of 60 one each	Gegham 1	2	TS	-	13-Jun	529	6/30/23	713	4134	35	227	13	26	73	55	3.08	0.17	0.35	0.75	0.06	0.11	0.32	0.24
597	Trench 2 Top Soil 13-JUN-22 sample of 60 one each	Kelbadjar	2	TS	-	13-Jun	530	6/30/23	511	5379	40	166	16	11	112	35	1.48	0.14	0.09	0.31	0.09	0.06	0.67	0.21
598	Trench 2 Top Soil 13-JUN-22 sample of 60 one each	Gegham 1	2	TS	-	13-Jun	531	6/30/23	715	4146	36	219	14	26	73	57	2.98	0.18	0.35	0.77	0.06	0.12	0.34	0.26
599	Trench 2 Top Soil 13-JUN-22 sample of 60 one each	Gegham 1	2	TS	-	13-Jun	532	6/30/23	825	4577	41	240	15	25	75	54	3.19	0.19	0.33	0.71	0.06	0.10	0.31	0.22
600	Trench 2 Top Soil 13-JUN-22 sample of 60 one each	Gegham 1	2	TS	-	13-Jun	533	6/30/23	752	4259	36	227	13	25	73	54	3.12	0.17	0.34	0.74	0.06	0.11	0.32	0.24
601	Trench 2 Top Soil 13-JUN-22 sample of 60 one each	Gegham 1	2	TS	-	13-Jun	534	6/30/23	701	4093	35	212	14	26	70	54	3.04	0.19	0.37	0.77	0.06	0.12	0.33	0.25
602	Trench 2 Top Soil 13-JUN-22 sample of 60 one each	Gegham 1	2	TS	-	13-Jun	535	6/30/23	676	3730	32	208	11	27	72	55	2.91	0.16	0.37	0.77	0.06	0.13	0.34	0.27
603	Trench 2 Top Soil 13-JUN-22 sample of 60 one each	Kelbadjar	2	TS	-	13-Jun	536	6/30/23	519	5311	38	164	17	8	117	36	1.40	0.14	0.07	0.31	0.10	0.05	0.72	0.22
604	Trench 2 Top Soil 13-JUN-22 sample of 60 one each	Gegham 1	2	TS	-	13-Jun	537	6/30/23	650	3922	35	219	15	27	74	55	2.94	0.19	0.36	0.74	0.07	0.12	0.34	0.25
605	Trench 2 Top Soil 13-JUN-22 sample of 60 one each	Gegham 1	2	TS	-	13-Jun	538	6/30/23	727	4002	31	216	13	24	72	57	3.02	0.17	0.33	0.79	0.06	0.11	0.33	0.26

606	Trench 2 Top Soil 13-JUN-22 sample of 60 one each	Gegham 1	2	TS	-	13-Jun	539	6/30/23	705	3930	34	214	14	26	73	54	2.95	0.19	0.35	0.74	0.06	0.12	0.34	0.25
607	Trench 2 Top Soil 13-JUN-22 sample of 60 one each	Kelbadjar	2	TS	-	13-Jun	540	6/30/23	597	6180	52	178	18	10	111	37	1.60	0.16	0.09	0.34	0.10	0.05	0.63	0.21
608	Trench 2 Top Soil 13-JUN-22 sample of 60 one each	Syunik	2	TS	-	13-Jun	541	6/30/23	520	5284	39	190	22	10	105	36	1.81	0.21	0.09	0.34	0.11	0.05	0.55	0.19
609	Trench 2 Top Soil 13-JUN-22 sample of 60 one each	Gegham 1	2	TS	-	13-Jun	542	6/30/23	676	3783	33	206	14	26	73	54	2.81	0.18	0.35	0.73	0.07	0.12	0.36	0.26
610	Tr-2 H-2 Sp-2 16 Jun 22 one each	Gegham 1	2	2	2	16-Jun	1	7/1/23	687	3983	34	215	13	26	72	54	3.00	0.17	0.36	0.75	0.06	0.12	0.33	0.25
611	Tr-2 H-2 Sp-2 16 Jun 22 one each	Gegham 1	2	2	2	16-Jun	2	7/1/23	673	3828	33	206	13	27	74	58	2.77	0.17	0.36	0.78	0.06	0.13	0.36	0.28
612	Tr-2 H-2 Sp-2 16 Jun 22 one each	Kelbadjar	2	2	2	16-Jun	3	7/1/23	524	5630	46	165	19	11	113	37	1.46	0.16	0.10	0.33	0.11	0.07	0.68	0.23
613	Tr-2 H-2 Sp-2 16 Jun 22 one each	Kelbadjar	2	2	2	16-Jun	4	7/1/23	489	5076	41	155	17	9	108	33	1.43	0.15	0.08	0.31	0.11	0.06	0.70	0.22
614	Tr-2 H-2 Sp-2 16 Jun 22 one each	Syunik	2	2	2	16-Jun	5	7/1/23	508	4878	34	181	18	11	102	37	1.78	0.17	0.11	0.37	0.10	0.06	0.56	0.21
615	Tr-2 H-2 Sp-2 16 Jun 22 one each	Kelbadjar	2	2	2	16-Jun	6	7/1/23	523	5259	43	159	16	9	110	39	1.44	0.14	0.08	0.35	0.10	0.05	0.70	0.24
616	Tr-2 H-2 Sp-2 16 Jun 22 one each	Kelbadjar	2	2	2	16-Jun	7	7/1/23	511	5846	42	163	25	11	120	36	1.36	0.20	0.10	0.30	0.15	0.07	0.74	0.22
617	Tr-2 H-2 Sp-2 16 Jun 22 one each	Gegham 1	2	2	2	16-Jun	8	7/1/23	733	3994	34	215	13	25	74	54	2.89	0.17	0.33	0.72	0.06	0.11	0.35	0.25
618	Tr-2 H-2 Sp-2 16 Jun 22 one each	Kelbadjar	2	2	2	16-Jun	9	7/1/23	523	5095	39	160	16	9	103	33	1.55	0.15	0.08	0.32	0.10	0.05	0.64	0.21
619	Tr-2 H-2 Sp-2 16 Jun 22 one each	Gegham 1	2	2	2	16-Jun	10	7/1/23	652	3834	31	201	15	24	69	51	2.93	0.21	0.34	0.74	0.07	0.12	0.34	0.25
620	Tr-2 H-2 Sp-2 16 Jun 22 one each	Kelbadjar	2	2	2	16-Jun	12	7/1/23	508	5544	39	164	17	11	111	33	1.48	0.15	0.10	0.30	0.10	0.07	0.68	0.20
621	Tr-2 H-2 Sp-2 16 Jun 22 one each	Kelbadjar	2	2	2	16-Jun	13	7/1/23	565	5720	43	169	18	11	113	36	1.50	0.15	0.09	0.32	0.10	0.06	0.67	0.21
622	Tr-2 H-2 Sp-2 16 Jun 22 one each	Kelbadjar	2	2	2	16-Jun	14	7/1/23	519	5587	39	168	17	11	116	33	1.45	0.14	0.10	0.29	0.10	0.07	0.69	0.20
623	Tr-2 H-2 Sp-2 16 Jun 22 one each	Kelbadjar	2	2	2	16-Jun	15	7/1/23	514	5201	42	162	16	12	106	35	1.53	0.15	0.12	0.33	0.10	0.08	0.65	0.21
624	Tr-2 H-2 Sp-2 16 Jun 22 one each	Gegham 1	2	2	2	16-Jun	16	7/1/23	746	4135	35	221	13	27	73	57	3.00	0.17	0.36	0.77	0.06	0.12	0.33	0.26
625	Tr-2 H-2 Sp-2 16 Jun 22 one each	Gegham 1	2	2	2	16-Jun	17	7/1/23	632	3807	32	205	16	28	75	54	2.72	0.21	0.38	0.71	0.08	0.14	0.37	0.26
626	Tr-2 H-2 Sp-2 16 Jun 22 one each	Kelbadjar	2	2	2	16-Jun	18	7/1/23	476	5196	43	163	16	11	116	39	1.40	0.13	0.09	0.33	0.10	0.06	0.71	0.24
627	Tr-2 H-2 Sp-2 16 Jun 22 one each	Gegham 1	2	2	2	16-Jun	19	7/1/23	690	4197	36	213	14	26	76	55	2.79	0.18	0.34	0.72	0.06	0.12	0.36	0.26
628	Tr-2 H-2 Sp-2 16 Jun 22 one each	Gegham 1	2	2	2	16-Jun	20	7/1/23	693	4003	36	216	15	25	74	58	2.90	0.19	0.33	0.78	0.07	0.11	0.34	0.27
629	Tr-2 H-2 Sp-2 16 Jun 22 one each	Gegham 1	2	2	2	16-Jun	21	7/1/23	661	3891	33	206	14	24	70	51	2.96	0.19	0.34	0.73	0.07	0.11	0.34	0.25
630	Tr-2 H-2 Sp-2 16 Jun 22 one each	Gegham 1	2	2	2	16-Jun	22	7/1/23	697	4156	39	221	14	25	77	54	2.86	0.17	0.32	0.70	0.06	0.11	0.35	0.24
631	Tr-2 H-2 Sp-2 16 Jun 22 one each	Kelbadjar	2	2	2	16-Jun	23	7/1/23	474	5024	38	157	15	11	109	32	1.43	0.13	0.10	0.29	0.09	0.07	0.70	0.20
632	Tr-2 H-2 Sp-2 16 Jun 22 one each	Kelbadjar	2	2	2	16-Jun	24	7/1/23	531	5570	42	167	18	11	113	37	1.48	0.15	0.10	0.33	0.10	0.07	0.68	0.22
633	Tr-2 H-2 Sp-2 16 Jun 22 one each	Gegham 1	2	2	2	16-Jun	25	7/1/23	622	3804	32	205	14	26	74	53	2.76	0.18	0.34	0.71	0.07	0.12	0.36	0.26
634	Tr-2 H-2 Sp-2 16 Jun 22 one each	Gegham 1	2	2	2	16-Jun	26	7/1/23	672	3946	35	215	15	25	72	54	3.00	0.20	0.34	0.75	0.07	0.11	0.33	0.25
635	Tr-2 H-2 Sp-2 16 Jun 22 one each	Kelbadjar	2	2	2	16-Jun	27	7/1/23	500	5450	38	164	18	11	107	33	1.54	0.16	0.10	0.31	0.11	0.06	0.65	0.20
636	Tr-2 H-2 Sp-2 16 Jun 22 one each	Gegham 1	2	2	2	16-Jun	28	7/1/23	757	4149	36	215	11	25	70	51	3.08	0.16	0.35	0.73	0.05	0.11	0.32	0.24
637	Tr-2 H-2 Sp-2 16 Jun 22 one each	Gegham 1	2	2	2	16-Jun	29	7/1/23	792	4516	38	231	16	27	77	58	2.98	0.20	0.36	0.75	0.07	0.12	0.34	0.25
638	Tr-2 H-2 Sp-2 16 Jun 22 one each	Gegham 1	2	2	2	16-Jun	30	7/1/23	681	4160	37	219	16	27	74	55	2.94	0.21	0.36	0.74	0.07	0.12	0.34	0.25
639	Tr-2 H-2 Sp-2 16 Jun 22 one each	Gegham 1	2	2	2	16-Jun	31	7/1/23	833	5265	47	231	28	27	76	55	3.02	0.36	0.36	0.72	0.12	0.12	0.33	0.24
640	Tr-2 H-2 Sp-2 16 Jun 22 one each	Gegham 1	2	2	2	16-Jun	32	7/1/23	705	4156	38	224	15	25	75	55	2.97	0.19	0.33	0.73	0.06	0.11	0.34	0.25
641	Tr-2 H-2 Sp-2 16 Jun 22 one each	Gegham 1	2	2	2	16-Jun	33	7/1/23	741	4073	36	217	13	26	84	54	2.59	0.15	0.30	0.64	0.06	0.12	0.39	0.25
642	Tr-2 H-2 Sp-2 16 Jun 22 one each	Kelbadjar	2	2	2	16-Jun	34	7/1/23	546	5506	42	170	18	11	111	35	1.53	0.16	0.10	0.31	0.10	0.07	0.65	0.20
643	Tr-2 H-2 Sp-2 16 Jun 22 one each	Gegham 1	2	2	2	16-Jun	35	7/1/23	693	4127	35	222	17	25	76	57	2.91	0.22	0.32	0.74	0.07	0.11	0.34	0.26
644	Tr-2 H-2 Sp-2 16 Jun 22 one each	Gegham 1	2	2	2	16-Jun	36	7/1/23	657	3916	32	206	15	27	73	55	2.84	0.20	0.38	0.76	0.07	0.13	0.35	0.27
645	Tr-2 H-2 Sp-2 16 Jun 22 one each	Kelbadjar	2	2	2	16-Jun	37	7/1/23	620	6319	46	184	18	11	112	35	1.64	0.16	0.10	0.31	0.10	0.06	0.61	0.19
646	Tr-2 H-2 Sp-2 16 Jun 22 one each	Gegham 1	2	2	2	16-Jun	38	7/1/23	727	4165	36	220	13	24	73	57	2.99	0.17	0.32	0.77	0.06	0.11	0.33	0.26
647	Tr-2 H-2 Sp-2 16 Jun 22 one each	Kelbadjar	2	2	2	16-Jun	39	7/1/23	566	5570	43	167	19	11	119	35	1.41	0.16	0.09	0.29	0.11	0.06	0.71	0.21
648	Tr-2 H-2 Sp-2 16 Jun 22 one each	Gegham 1	2	2	2	16-Jun	40	7/1/23	718	4062	37	220	14	24	74	55	2.95	0.18	0.32	0.74	0.06	0.11	0.34	0.25
649	Tr-2 H-2 Sp-2 16 Jun 22 one each	Kelbadjar	2	2	2	16-Jun	41	7/1/23	574	5793	46	170	20	11	115	39	1.48	0.17	0.10	0.34	0.11	0.07	0.68	0.23
650	Tr-2 H-2 Sp-2 16 Jun 22 one each	Gegham 1	2	2	2	16-Jun	42	7/1/23	711	4236	38	220	15	24	75	54	2.92	0.19	0.31	0.71	0.07	0.11	0.34	0.25
651	Tr-2 H-2 Sp-2 16 Jun 22 one each	Kelbadjar	2	2	2	16-Jun	43	7/1/23	523	5183	39	164	15	13	122	36	1.35	0.12	0.11	0.30	0.09	0.08	0.74	0.22
652	Tr-2 H-2 Sp-2 16 Jun 22 one each	Gegham 1	2	2	2	16-Jun	45	7/1/23	666	3854	35	210	13	28	74	54	2.82	0.17	0.38	0.72	0.06	0.14	0.35	0.26
653	Tr-2 H-2 Sp-2 16 Jun 22 one each	Gegham 1	2	2	2	16-Jun	46	7/1/23	774	4545	35	229	15	25	76	54	3.00	0.19	0.32	0.71	0.06	0.11	0.33	0.24
654	Tr-2 H-2 Sp-2 16 Jun 22 one each	Kelbadjar	2	2	2	16-Jun	47	7/1/23	621	5924	44	169	19	13	110	35	1.53	0.17	0.12	0.31	0.11	0.08	0.65	0.20
655	Tr-2 H-2 Sp-2 16 Jun 22 one each	Gegham 1	2	2	2	16-Jun	48	7/1/23	771	4355	38	227	14	25	74	55	3.05	0.18	0.33	0.74	0.06	0.11	0.33	0.24
656	Tr-2 H-2 Sp-2 16 Jun 22 one each	Gegham 1	2	2	2	16-Jun	49	7/1/23	646	3805	33	207	14	24	71	55	2.93	0.19	0.34	0.78	0.07	0.11	0.34	0.27

657	Tr-2 H-2 Sp-2 16 Jun 22 one each	Gegham 1	2	2	2	16-Jun	50	7/1/23	746	4151	38	219	13	24	73	54	3.02	0.17	0.33	0.74	0.06	0.11	0.33	0.25
658	Tr-2 H-2 Sp-2 16 Jun 22 one each	Gegham 1	2	2	2	16-Jun	51	7/1/23	700	4149	33	218	14	25	72	55	3.04	0.19	0.34	0.77	0.06	0.11	0.33	0.25
659	Tr-2 H-2 Sp-2 16 Jun 22 one each	Kelbadjar	2	2	2	16-Jun	52	7/1/23	605	6023	46	180	17	11	114	36	1.58	0.14	0.09	0.32	0.09	0.06	0.63	0.20
660	Tr-2 H-2 Sp-2 16 Jun 22 one each	Gegham 1	2	2	2	16-Jun	53	7/1/23	728	4160	35	219	13	27	73	57	2.98	0.17	0.37	0.77	0.06	0.13	0.34	0.26
661	Tr-2 H-2 Sp-2 16 Jun 22 one each	Gegham 1	2	2	2	16-Jun	54	7/1/23	687	3992	33	212	13	26	71	57	3.00	0.18	0.36	0.80	0.06	0.12	0.33	0.27
662	Tr-2 H-2 Sp-2 16 Jun 22 one each	Kelbadjar	2	2	2	16-Jun	55	7/1/23	506	4998	40	159	16	10	107	33	1.49	0.15	0.09	0.31	0.10	0.06	0.67	0.21
663	Tr-2 H-2 Sp-2 16 Jun 22 one each	Gegham 1	2	2	2	16-Jun	56	7/1/23	712	4022	35	215	11	24	73	55	2.96	0.16	0.33	0.76	0.05	0.11	0.34	0.26
664	Tr-2 H-2 Sp-2 16 Jun 22 one each	Kelbadjar	2	2	2	16-Jun	58	7/1/23	495	5260	39	160	17	12	111	36	1.43	0.15	0.11	0.32	0.10	0.08	0.70	0.23
665	Tr-2 H-2 Sp-2 16 Jun 22 one each	Syunik	2	2	2	16-Jun	59	7/1/23	488	5376	36	182	22	11	106	37	1.72	0.20	0.11	0.35	0.12	0.06	0.58	0.21
666	Tr-2 H-2 Sp-2 16 Jun 22 one each	Gegham 1	2	2	2	16-Jun	60	7/1/23	716	4187	35	223	14	27	73	55	3.07	0.19	0.37	0.76	0.06	0.12	0.33	0.25
667	Tr-2 H-2 Sp-2 16 Jun 22 one each	Gegham 1	2	2	2	16-Jun	61	7/1/23	787	4382	38	231	13	27	75	57	3.06	0.17	0.36	0.75	0.05	0.12	0.33	0.25
668	Tr-2 H-2 Sp-2 16 Jun 22 one each	Gegham 1	2	2	2	16-Jun	62	7/1/23	729	4070	36	219	11	27	78	59	2.80	0.15	0.35	0.76	0.05	0.13	0.36	0.27
669	Tr-2 H-2 Sp-2 16 Jun 22 one each	Gegham 1	2	2	2	16-Jun	63	7/1/23	701	3892	38	208	13	27	74	57	2.80	0.17	0.36	0.76	0.06	0.13	0.36	0.27
670	Tr-2 H-2 Sp-2 16 Jun 22 one each	Kelbadjar	2	2	2	16-Jun	64	7/1/23	549	5768	46	166	19	12	117	35	1.42	0.16	0.11	0.30	0.11	0.07	0.70	0.21
671	Tr-2 H-2 Sp-2 16 Jun 22 one each	Gegham 1	2	2	2	16-Jun	65	7/1/23	766	4152	36	223	14	26	74	55	2.99	0.18	0.34	0.74	0.06	0.11	0.33	0.25
672	Tr-2 H-2 Sp-2 16 Jun 22 one each	Syunik	2	2	2	16-Jun	66	7/1/23	488	5398	39	182	25	11	108	32	1.68	0.23	0.11	0.29	0.13	0.06	0.60	0.18
673	Tr-2 H-2 Sp-2 16 Jun 22 one each	Kelbadjar	2	2	2	16-Jun	67	7/1/23	547	5615	46	166	18	11	108	36	1.55	0.16	0.10	0.34	0.11	0.06	0.65	0.22
674	Tr-2 H-2 Sp-2 16 Jun 22 one each	Gegham 1	2	2	2	16-Jun	68	7/1/23	762	4318	37	226	14	26	76	55	2.96	0.18	0.34	0.72	0.06	0.11	0.34	0.24
675	Tr-2 H-2 Sp-1 16Jun22 smalls sample of 25 one each	Gegham 1	2	2	1	16-Jun	69	7/1/23	763	4297	37	227	13	27	76	55	2.97	0.16	0.35	0.72	0.06	0.12	0.34	0.24
676	Tr-2 H-2 Sp-1 16Jun22 smalls sample of 25 one each	Kelbadjar	2	2	1	16-Jun	70	7/1/23	511	5115	38	161	16	9	108	36	1.49	0.14	0.08	0.34	0.10	0.05	0.67	0.22
677	Tr-2 H-2 Sp-1 16Jun22 smalls sample of 25 one each	Kelbadjar	2	2	1	16-Jun	71	7/1/23	536	5689	45	176	18	11	112	36	1.57	0.16	0.10	0.32	0.10	0.07	0.64	0.20
678	Tr-2 H-2 Sp-1 16Jun22 smalls sample of 25 one each	Gegham 1	2	2	1	16-Jun	72	7/1/23	676	3994	35	214	16	27	78	58	2.74	0.20	0.34	0.74	0.07	0.12	0.37	0.27
679	Tr-2 H-2 Sp-1 16Jun22 smalls sample of 25 one each	Gegham 1	2	2	1	16-Jun	73	7/1/23	658	3835	34	211	14	26	71	55	2.99	0.19	0.36	0.78	0.06	0.12	0.33	0.26
680	Tr-2 H-2 Sp-1 16Jun22 smalls sample of 25 one each	Gegham 1	2	2	1	16-Jun	74	7/1/23	833	4861	43	245	16	26	74	53	3.29	0.21	0.34	0.71	0.06	0.10	0.30	0.21
681	Tr-2 H-2 Sp-1 16Jun22 smalls sample of 25 one each	Kelbadjar	2	2	1	16-Jun	75	7/1/23	612	5688	46	168	19	9	114	36	1.48	0.16	0.08	0.32	0.11	0.05	0.68	0.21
682	Tr-2 H-2 Sp-1 16Jun22 smalls sample of 25 one each	Kelbadjar	2	2	1	16-Jun	76	7/1/23	506	5085	40	161	17	12	114	37	1.41	0.14	0.11	0.33	0.10	0.08	0.71	0.23
683	Tr-2 H-2 Sp-1 16Jun22 smalls sample of 25 one each	Gegham 1	2	2	1	16-Jun	77	7/1/23	787	4307	36	230	13	27	75	58	3.05	0.17	0.35	0.77	0.05	0.12	0.33	0.25
684	Tr-2 H-2 Sp-1 16Jun22 smalls sample of 25 one each	Gegham 1	2	2	1	16-Jun	78	7/1/23	762	4231	35	223	13	27	76	57	2.92	0.16	0.35	0.74	0.06	0.12	0.34	0.25
685	Tr-2 H-2 Sp-1 16Jun22 smalls sample of 25 one each	Kelbadjar	2	2	1	16-Jun	79	7/1/23	510	5321	40	164	16	11	112	36	1.47	0.14	0.09	0.32	0.09	0.06	0.68	0.22
686	Tr-2 H-2 Sp-1 16Jun22 smalls sample of 25 one each	Kelbadjar	2	2	1	16-Jun	80	7/1/23	562	6088	44	178	18	10	110	36	1.61	0.16	0.09	0.33	0.10	0.05	0.62	0.20
687	Tr-2 H-2 Sp-1 16Jun22 smalls sample of 25 one each	Gegham 1	2	2	1	16-Jun	81	7/1/23	690	4012	38	217	15	26	73	55	2.95	0.20	0.35	0.75	0.07	0.12	0.34	0.25
688	Tr-2 H-2 Sp-1 16Jun22 smalls sample of 25 one each	Syunik	2	2	1	16-Jun	82	7/1/23	521	5948	40	185	30	11	107	36	1.73	0.28	0.10	0.34	0.16	0.06	0.58	0.19
689	Tr-2 H-2 Sp-1 16Jun22 smalls sample of 25 one each	Gegham 1	2	2	1	16-Jun	83	7/1/23	759	4228	36	225	11	26	73	53	3.10	0.16	0.35	0.72	0.05	0.11	0.32	0.23
690	Tr-2 H-2 Sp-1 16Jun22 smalls sample of 25 one each	Gegham 1	2	2	1	16-Jun	84	7/1/23	729	4079	35	220	14	25	72	55	3.07	0.19	0.34	0.77	0.06	0.11	0.33	0.25
691	Tr-2 H-2 Sp-1 16Jun22 smalls sample of 25 one each	Gegham 1	2	2	1	16-Jun	85	7/1/23	739	4267	35	224	14	27	74	57	3.01	0.18	0.36	0.76	0.06	0.12	0.33	0.25
692	Tr-2 H-2 Sp-1 16Jun22 smalls sample of 25 one each	Gegham 1	2	2	1	16-Jun	86	7/1/23	713	4048	31	220	15	26	77	58	2.85	0.19	0.33	0.75	0.07	0.12	0.35	0.26
693	Tr-2 H-2 Sp-1 16Jun22 smalls sample of 25 one each	Kelbadjar	2	2	1	16-Jun	87	7/1/23	505	5443	46	170	17	10	113	35	1.50	0.15	0.08	0.31	0.10	0.06	0.66	0.20
694	Tr-2 H-2 Sp-1 16Jun22 smalls sample of 25 one each	Kelbadjar	2	2	1	16-Jun	88	7/1/23	519	5379	42	166	17	12	117	36	1.42	0.14	0.11	0.31	0.10	0.07	0.70	0.22
695	Tr-2 H-2 Sp-1 16Jun22 smalls sample of 25 one each	Kelbadjar	2	2	1	16-Jun	89	7/1/23	556	5557	44	168	18	11	109	36	1.54	0.16	0.10	0.33	0.10	0.07	0.65	0.21
696	Tr-2 H-2 Sp-1 16Jun22 smalls sample of 25 one each	Syunik	2	2	1	16-Jun	90	7/1/23	495	5135	35	180	24	12	110	37	1.63	0.21	0.11	0.34	0.13	0.07	0.61	0.21
697	Tr-2 H-2 Sp-1 16Jun22 smalls sample of 25 one each	Gegham 1	2	2	1	16-Jun	91	7/1/23	672	4241	34	206	17	22	77	51	2.67	0.21	0.28	0.66	0.08	0.11	0.37	0.25
698	Tr-2 H-2 Sp-1 16Jun22 smalls sample of 25 one each	Gegham 1	2	2	1	16-Jun	92	7/1/23	859	4599	41	243	16	25	73	54	3.31	0.21	0.34	0.73	0.06	0.10	0.30	0.22
699	Tr-2 H-2 Sp-1 16Jun22 smalls sample of 25 one each	Gegham 1	2	2	1	16-Jun	93	7/1/23	733	4232	36	218	14	27	76	54	2.86	0.18	0.36	0.71	0.06	0.13	0.35	0.25
700	Trench-2 Hor-2 Sp-1 15Jun22 one each	Kelbadjar	2	2	1	15-Jun	94	7/1/23	484	4934	43	156	19	11	106	35	1.47	0.18	0.11	0.33	0.12	0.07	0.68	0.22
701	Trench-2 Hor-2 Sp-1 15Jun22 one each	Kelbadjar	2	2	1	15-Jun	95	7/1/23	478	5253	41	164	16	11	121	36	1.35	0.13	0.09	0.30	0.09	0.07	0.74	0.22
702	Trench-2 Hor-2 Sp-1 15Jun22 one each	Gegham 1	2	2	1	15-Jun	96	7/1/23	736	4051	38	214	13	25	73	55	2.95	0.17	0.34	0.76	0.06	0.12	0.34	0.26
703	Trench-2 Hor-2 Sp-1 15Jun22 one each	Kelbadjar	2	2	1	15-Jun	97	7/1/23	515	5347	43	164	16	10	106	35	1.55	0.15	0.09	0.33	0.09	0.06	0.65	0.21
704	Trench-2 Hor-2 Sp-1 15Jun22 one each	Kelbadjar	2	2	1	15-Jun	98	7/1/23	481	4749	36	154	16	11	110	33	1.39	0.14	0.10	0.30	0.10	0.07	0.72	0.22
705	Trench-2 Hor-2 Sp-1 15Jun22 one each	Gegham 1	2	2	1	15-Jun	99	7/1/23	711	4124	32	212	15	26	72	58	2.96	0.20	0.36	0.81	0.07	0.12	0.34	0.27
706	Trench-2 Hor-2 Sp-1 15Jun22 one each	Syunik	2	2	1	15-Jun	100	7/1/23	464	4767	35	176	24	11	101	35	1.75	0.23	0.11	0.34	0.13	0.07	0.57	0.20
707	Trench-2 Hor-2 Sp-1 15Jun22 one each	Syunik	2	2	1	15-Jun	101	7/1/23	633	4521	40	226	8	7	91	39	2.49	0.09	0.07	0.43	0.04	0.03	0.40	0.17

708	Trench-2 Hor-2 Sp-1 15Jun22 one each	Kelbadjar	2	2	1	15-Jun	102	7/1/23	466	5155	41	160	23	11	134	32	1.19	0.17	0.09	0.24	0.14	0.07	0.84	0.20
709	Trench-2 Hor-2 Sp-1 15Jun22 one each	Gegham 1	2	2	1	15-Jun	103	7/1/23	682	3876	35	215	13	26	74	57	2.89	0.17	0.34	0.76	0.06	0.12	0.35	0.26
710	Trench-2 Hor-2 Sp-1 15Jun22 one each	Kelbadjar	2	2	1	15-Jun	104	7/1/23	451	4722	39	151	20	11	108	36	1.40	0.18	0.11	0.34	0.13	0.08	0.71	0.24
711	Trench-2 Hor-2 Sp-1 15Jun22 one each	Gegham 1	2	2	1	15-Jun	105	7/1/23	655	3653	32	207	11	26	73	57	2.82	0.16	0.35	0.77	0.06	0.12	0.35	0.27
712	Trench-2 Hor-2 Sp-1 15Jun22 one each	Kelbadjar	2	2	1	15-Jun	106	7/1/23	524	5651	43	162	19	12	114	36	1.42	0.16	0.11	0.32	0.11	0.08	0.71	0.22
713	Trench-2 Hor-2 Sp-1 15Jun22 one each	Gegham 1	2	2	1	15-Jun	107	7/1/23	772	4213	34	220	14	26	75	55	2.92	0.18	0.34	0.73	0.06	0.12	0.34	0.25
714	Trench-2 Hor-2 Sp-1 15Jun22 one each	Gegham 1	2	2	1	15-Jun	108	7/1/23	727	4301	35	225	14	26	78	57	2.87	0.17	0.33	0.72	0.06	0.11	0.35	0.25
715	Trench-2 Hor-2 Sp-1 15Jun22 one each	Gegham 1	2	2	1	15-Jun	110	7/1/23	670	3813	31	204	13	27	73	55	2.82	0.17	0.37	0.76	0.06	0.13	0.36	0.27
716	Trench-2 Hor-2 Sp-1 15Jun22 one each	Syunik	2	2	1	15-Jun	111	7/1/23	508	5365	32	175	25	10	104	35	1.69	0.24	0.09	0.33	0.14	0.05	0.59	0.20
717	Trench-2 Hor-2 Sp-1 15Jun22 one each	Kelbadjar	2	2	1	15-Jun	112	7/1/23	521	5071	43	161	15	11	111	35	1.44	0.13	0.09	0.31	0.09	0.07	0.69	0.22
718	Trench-2 Hor-2 Sp-1 15Jun22 one each	Gegham 1	2	2	1	15-Jun	113	7/1/23	599	3629	35	200	16	24	73	54	2.76	0.21	0.33	0.74	0.08	0.12	0.36	0.27
719	Trench-2 Hor-2 Sp-1 15Jun22 one each	Kelbadjar	2	2	1	15-Jun	114	7/1/23	508	5259	39	159	16	10	108	36	1.48	0.14	0.09	0.34	0.10	0.06	0.68	0.23
720	Trench-2 Hor-2 Sp-1 15Jun22 one each	Gegham 1	2	2	1	15-Jun	115	7/1/23	675	3847	32	214	13	23	74	54	2.88	0.17	0.31	0.72	0.06	0.11	0.35	0.25
721	Trench-2 Hor-2 Sp-1 15Jun22 one each	Kelbadjar	2	2	1	15-Jun	116	7/1/23	470	5024	39	156	15	10	108	32	1.45	0.13	0.09	0.30	0.09	0.06	0.69	0.20
722	Trench-2 Hor-2 Sp-1 15Jun22 one each	Gegham 1	2	2	1	15-Jun	117	7/1/23	682	3869	34	212	14	24	73	55	2.89	0.18	0.32	0.75	0.06	0.11	0.35	0.26
723	Trench-2 Hor-2 Sp-1 15Jun22 one each	Gegham 1	2	2	1	15-Jun	118	7/1/23	751	4380	37	231	14	25	73	54	3.14	0.18	0.34	0.73	0.06	0.11	0.32	0.23
724	Trench-2 Hor-2 Sp-1 15Jun22 one each	Kelbadjar	2	2	1	15-Jun	119	7/1/23	505	4916	38	159	17	11	104	35	1.53	0.16	0.10	0.33	0.10	0.07	0.65	0.22
725	Trench-2 Hor-2 Sp-1 15Jun22 one each	Kelbadjar	2	2	1	15-Jun	121	7/1/23	520	5091	36	160	17	11	109	36	1.46	0.15	0.10	0.33	0.10	0.07	0.69	0.23
726	Trench-2 Hor-2 Sp-1 15Jun22 one each	Gegham 1	2	2	1	15-Jun	122	7/1/23	870	4698	43	244	14	21	71	54	3.46	0.19	0.30	0.76	0.06	0.09	0.29	0.22
727	Trench-2 Hor-2 Sp-1 15Jun22 one each	Kelbadjar	2	2	1	15-Jun	123	7/1/23	490	4973	39	164	19	11	113	37	1.45	0.16	0.10	0.33	0.11	0.07	0.69	0.23
728	Trench-2 Hor-2 Sp-1 15Jun22 one each	Kelbadjar	2	2	1	15-Jun	124	7/1/23	476	5070	36	166	16	11	115	36	1.45	0.13	0.10	0.31	0.09	0.07	0.69	0.22
729	Trench-2 Hor-2 Sp-1 15Jun22 one each	Kelbadjar	2	2	1	15-Jun	125	7/1/23	494	5246	41	163	17	11	116	36	1.40	0.14	0.09	0.31	0.10	0.06	0.71	0.22
730	Trench-2 Hor-2 Sp-1 15Jun22 one each	Gegham 1	2	2	1	15-Jun	126	7/1/23	693	3951	37	218	13	27	73	55	2.97	0.17	0.36	0.75	0.06	0.12	0.34	0.25
731	Trench-2 Hor-2 Sp-1 15Jun22 one each	Gegham 1	2	2	1	15-Jun	127	7/1/23	688	3926	32	215	13	24	73	55	2.93	0.17	0.32	0.75	0.06	0.11	0.34	0.26
732	Trench-2 Hor-2 Sp-1 15Jun22 one each	Gutansar	2	2	1	15-Jun	128	7/1/23	614	8501	53	143	135	25	183	35	0.78	0.74	0.13	0.19	0.94	0.17	1.28	0.24
733	Trench-2 Hor-2 Sp-1 15Jun22 one each	Kelbadjar	2	2	1	15-Jun	129	7/1/23	624	6024	46	178	19	10	121	37	1.47	0.15	0.08	0.31	0.10	0.05	0.68	0.21
734	Trench-2 Hor-2 Sp-1 15Jun22 one each	Gegham 1	2	2	1	15-Jun	130	7/1/23	806	4387	37	232	11	26	77	57	3.00	0.15	0.33	0.73	0.05	0.11	0.33	0.24
735	Trench-2 Hor-2 Sp-1 15Jun22 one each	Kelbadjar	2	2	1	15-Jun	131	7/1/23	579	5839	44	174	16	11	112	36	1.55	0.14	0.09	0.32	0.09	0.06	0.64	0.21
736	Trench-2 Hor-2 Sp-1 15Jun22 one each	Gegham 1	2	2	1	15-Jun	132	7/1/23	713	4037	31	218	14	25	73	58	2.97	0.18	0.34	0.79	0.06	0.11	0.34	0.27
737	Trench-2 Hor-2 Sp-1 15Jun22 one each	Kelbadjar	2	2	1	15-Jun	133	7/1/23	555	5547	41	164	18	11	111	33	1.48	0.16	0.09	0.30	0.11	0.06	0.68	0.20
738	Trench-2 Hor-2 Sp-1 15Jun22 one each	Kelbadjar	2	2	1	15-Jun	134	7/1/23	547	5814	43	173	18	12	112	36	1.54	0.16	0.11	0.32	0.10	0.07	0.65	0.21
739	Trench-2 Hor-2 Sp-1 15Jun22 one each	Gegham 1	2	2	1	15-Jun	135	7/1/23	696	3844	35	212	13	26	73	55	2.89	0.17	0.35	0.75	0.06	0.12	0.35	0.26
740	Trench-2 Hor-2 Sp-1 15Jun22 one each	Kelbadjar	2	2	1	15-Jun	136	7/1/23	485	5163	35	164	16	11	117	39	1.41	0.13	0.09	0.33	0.09	0.06	0.71	0.24
741	Trench-2 Hor-2 Sp-1 15Jun22 one each	Gegham 1	2	2	1	15-Jun	137	7/1/23	717	3961	35	219	13	25	76	55	2.87	0.16	0.32	0.72	0.06	0.11	0.35	0.25
742	Trench-2 Hor-2 Sp-1 15Jun22 one each	Gegham 1	2	2	1	15-Jun	138	7/1/23	814	4499	40	233	15	27	75	55	3.08	0.19	0.35	0.73	0.06	0.11	0.32	0.24
743	Trench-2 Hor-2 Sp-1 15Jun22 one each	Syunik	2	2	1	15-Jun	139	7/1/23	535	5700	42	193	23	10	108	33	1.79	0.21	0.09	0.31	0.12	0.05	0.56	0.17
744	Trench-2 Hor-2 Sp-1 15Jun22 one each	Kelbadjar	2	2	1	15-Jun	140	7/1/23	521	5417	40	163	18	11	132	37	1.23	0.13	0.09	0.28	0.11	0.07	0.81	0.23
745	Trench-2 Hor-2 Sp-1 15Jun22 one each	Gegham 1	2	2	1	15-Jun	141	7/1/23	791	4339	37	229	14	25	75	57	3.03	0.18	0.33	0.75	0.06	0.11	0.33	0.25
746	Trench-2 Hor-2 Sp-1 15Jun22 one each	Gegham 1	2	2	1	15-Jun	142	7/1/23	690	3873	35	213	11	27	75	57	2.83	0.15	0.35	0.75	0.05	0.12	0.35	0.27
747	Trench-2 Hor-2 Sp-1 15Jun22 one each	Gegham 1	2	2	1	15-Jun	143	7/1/23	783	4315	36	234	15	25	74	55	3.15	0.19	0.33	0.74	0.06	0.11	0.32	0.24
748	Trench-2 Hor-2 Sp-1 15Jun22 one each	Kelbadjar	2	2	1	15-Jun	144	7/1/23	544	5348	44	161	17	10	111	35	1.44	0.15	0.09	0.31	0.10	0.06	0.69	0.22
749	Trench-2 Hor-2 Sp-1 15Jun22 one each	Kelbadjar	2	2	1	15-Jun	145	7/1/23	515	5341	40	168	17	10	118	36	1.43	0.14	0.08	0.31	0.10	0.06	0.70	0.21
750	Trench-2 Hor-2 Sp-1 15Jun22 one each	Syunik	2	2	1	15-Jun	146	7/1/23	653	6466	49	187	19	10	116	36	1.61	0.16	0.08	0.31	0.10	0.05	0.62	0.19
751	Trench-2 Hor-2 Sp-1 15Jun22 one each	Gegham 1	2	2	1	15-Jun	147	7/1/23	742	4118	35	218	15	27	74	55	2.93	0.19	0.36	0.74	0.07	0.12	0.34	0.25
752	Trench-2 Hor-2 Sp-1 15Jun22 one each	Gegham 1	2	2	1	15-Jun	148	7/1/23	723	4070	37	219	13	25	75	55	2.90	0.17	0.33	0.73	0.06	0.11	0.34	0.25
753	Trench-2 Hor-2 Sp-1 15Jun22 one each	Gegham 1	2	2	1	15-Jun	149	7/1/23	692	4058	32	214	14	26	73	54	2.95	0.19	0.35	0.74	0.06	0.12	0.34	0.25
754	Trench-2 Hor-2 Sp-1 15Jun22 one each	Arteni	2	2	1	15-Jun	150	7/1/23	576	4625	39	128	35	25	85	29	1.50	0.41	0.29	0.34	0.27	0.19	0.66	0.23
755	Trench 2 Horizon 1 Spit 2 15-JUN-22 sample of 80 one each	Gegham 1	2	1	2	15-Jun	151	7/1/23	636	3574	30	198	11	26	73	54	2.74	0.16	0.35	0.74	0.06	0.13	0.37	0.27
756	Trench 2 Horizon 1 Spit 2 15-JUN-22 sample of 80 one each	Kelbadjar	2	1	2	15-Jun	152	7/1/23	530	5464	42	164	17	11	115	37	1.42	0.14	0.09	0.33	0.10	0.06	0.70	0.23
757	Trench 2 Horizon 1 Spit 2 15-JUN-22 sample of 80 one each	Kelbadjar	2	1	2	15-Jun	153	7/1/23	508	5382	41	162	17	12	122	35	1.33	0.14	0.10	0.28	0.10	0.08	0.75	0.21
758	Trench 2 Horizon 1 Spit 2 15-JUN-22 sample of 80 one each	Gegham 1	2	1	2	15-Jun	154	7/1/23	646	3846	36	207	14	24	71	53	2.93	0.19	0.34	0.74	0.07	0.11	0.34	0.25

759	Trench 2 Horizon 1 Spit 2 15-JUN-22 sample of 80 one each	Syunik	2	1	2	15-Jun	155	7/1/23	440	4628	41	174	23	10	95	32	1.83	0.24	0.10	0.34	0.13	0.06	0.55	0.18
760	Trench 2 Horizon 1 Spit 2 15-JUN-22 sample of 80 one each	Kelbadjar	2	1	2	15-Jun	156	7/1/23	458	4973	39	156	16	11	111	36	1.40	0.14	0.09	0.32	0.10	0.07	0.71	0.23
761	Trench 2 Horizon 1 Spit 2 15-JUN-22 sample of 80 one each	Gegham 1	2	1	2	15-Jun	157	7/1/23	711	3845	33	207	13	23	72	54	2.89	0.17	0.32	0.75	0.06	0.11	0.35	0.26
762	Trench 2 Horizon 1 Spit 2 15-JUN-22 sample of 80 one each	Kelbadjar	2	1	2	15-Jun	158	7/1/23	490	5110	36	157	18	10	108	33	1.44	0.16	0.09	0.31	0.11	0.06	0.69	0.21
763	Trench 2 Horizon 1 Spit 2 15-JUN-22 sample of 80 one each	Kelbadjar	2	1	2	15-Jun	159	7/1/23	508	4817	39	159	17	11	108	35	1.46	0.15	0.11	0.32	0.10	0.07	0.68	0.22
764	Trench 2 Horizon 1 Spit 2 15-JUN-22 sample of 80 one each	Kelbadjar	2	1	2	15-Jun	160	7/1/23	521	5141	39	161	16	10	108	32	1.49	0.14	0.09	0.30	0.10	0.06	0.67	0.20
765	Trench 2 Horizon 1 Spit 2 15-JUN-22 sample of 80 one each	Gegham 1	2	1	2	15-Jun	161	7/1/23	636	3674	35	203	15	27	70	53	2.92	0.21	0.38	0.75	0.07	0.13	0.34	0.26
766	Trench 2 Horizon 1 Spit 2 15-JUN-22 sample of 80 one each	Kelbadjar	2	1	2	15-Jun	162	7/1/23	451	4623	37	151	16	11	105	32	1.44	0.15	0.10	0.30	0.10	0.07	0.69	0.21
767	Trench 2 Horizon 1 Spit 2 15-JUN-22 sample of 80 one each	Kelbadjar	2	1	2	15-Jun	163	7/1/23	551	5568	47	166	15	11	111	33	1.50	0.13	0.10	0.30	0.09	0.07	0.67	0.20
768	Trench 2 Horizon 1 Spit 2 15-JUN-22 sample of 80 one each	Kelbadjar	2	1	2	15-Jun	164	7/1/23	506	5557	45	168	18	12	110	36	1.53	0.16	0.11	0.33	0.10	0.07	0.66	0.21
769	Trench 2 Horizon 1 Spit 2 15-JUN-22 sample of 80 one each	Kelbadjar	2	1	2	15-Jun	165	7/1/23	520	5171	40	158	17	11	115	33	1.37	0.14	0.10	0.29	0.10	0.07	0.73	0.21
770	Trench 2 Horizon 1 Spit 2 15-JUN-22 sample of 80 one each	Kelbadjar	2	1	2	15-Jun	167	7/1/23	515	5311	39	162	15	11	110	37	1.46	0.13	0.10	0.34	0.09	0.07	0.68	0.23
771	Trench 2 Horizon 1 Spit 2 15-JUN-22 sample of 80 one each	Gegham 1	2	1	2	15-Jun	168	7/1/23	741	4085	35	215	15	27	75	57	2.85	0.19	0.36	0.75	0.07	0.13	0.35	0.26
772	Trench 2 Horizon 1 Spit 2 15-JUN-22 sample of 80 one each	Kelbadjar	2	1	2	15-Jun	169	7/1/23	500	5235	37	162	16	11	113	36	1.43	0.14	0.09	0.32	0.10	0.07	0.70	0.22
773	Trench 2 Horizon 1 Spit 2 15-JUN-22 sample of 80 one each	Gegham 1	2	1	2	15-Jun	170	7/1/23	632	3839	33	203	18	27	73	54	2.80	0.24	0.37	0.74	0.09	0.13	0.36	0.26
774	Trench 2 Horizon 1 Spit 2 15-JUN-22 sample of 80 one each	Gegham 1	2	1	2	15-Jun	171	7/1/23	690	3926	34	209	14	27	73	55	2.85	0.18	0.37	0.75	0.06	0.13	0.35	0.26
775	Trench 2 Horizon 1 Spit 2 15-JUN-22 sample of 80 one each	Gegham 1	2	1	2	15-Jun	172	7/1/23	734	4057	39	221	14	26	74	55	2.97	0.18	0.34	0.74	0.06	0.12	0.34	0.25
776	Trench 2 Horizon 1 Spit 2 15-JUN-22 sample of 80 one each	Kelbadjar	2	1	2	15-Jun	173	7/1/23	526	5596	46	165	20	11	118	35	1.40	0.17	0.09	0.29	0.12	0.06	0.71	0.21
777	Trench 2 Horizon 1 Spit 2 15-JUN-22 sample of 80 one each	Arteni	2	1	2	15-Jun	174	7/1/23	668	4286	43	148	23	28	80	33	1.85	0.28	0.35	0.42	0.15	0.19	0.54	0.23
778	Trench 2 Horizon 1 Spit 2 15-JUN-22 sample of 80 one each	Gegham 1	2	1	2	15-Jun	176	7/1/23	670	3855	33	209	14	24	70	57	3.00	0.19	0.34	0.81	0.06	0.11	0.33	0.27
779	Trench 2 Horizon 1 Spit 2 15-JUN-22 sample of 80 one each	Gegham 1	2	1	2	15-Jun	177	7/1/23	672	3911	33	208	15	26	73	55	2.87	0.20	0.35	0.76	0.07	0.12	0.35	0.27
780	Trench 2 Horizon 1 Spit 2 15-JUN-22 sample of 80 one each	Kelbadjar	2	1	2	15-Jun	178	7/1/23	550	5354	40	164	18	10	109	33	1.50	0.16	0.09	0.30	0.11	0.06	0.67	0.20
781	Trench 2 Horizon 1 Spit 2 15-JUN-22 sample of 80 one each	Gegham 1	2	1	2	15-Jun	179	7/1/23	705	3861	34	204	14	27	74	54	2.74	0.18	0.36	0.72	0.07	0.13	0.36	0.26
782	Trench 2 Horizon 1 Spit 2 15-JUN-22 sample of 80 one each	Kelbadjar	2	1	2	15-Jun	180	7/1/23	528	5456	42	164	16	11	116	36	1.42	0.13	0.10	0.31	0.09	0.07	0.71	0.22
783	Trench 2 Horizon 1 Spit 2 15-JUN-22 sample of 80 one each	Kelbadjar	2	1	2	15-Jun	181	7/1/23	520	5371	47	168	19	11	105	33	1.61	0.18	0.10	0.32	0.11	0.06	0.62	0.20
784	Trench 2 Horizon 1 Spit 2 15-JUN-22 sample of 80 one each	Kelbadjar	2	1	2	15-Jun	182	7/1/23	566	5460	46	164	18	12	111	35	1.47	0.16	0.11	0.31	0.11	0.08	0.68	0.21
785	Trench 2 Horizon 1 Spit 2 15-JUN-22 sample of 80 one each	Gegham 1	2	1	2	15-Jun	183	7/1/23	676	3679	35	203	11	21	73	51	2.80	0.16	0.29	0.70	0.06	0.10	0.36	0.25
786	Trench 2 Horizon 1 Spit 2 15-JUN-22 sample of 80 one each	Gegham 1	2	1	2	15-Jun	184	7/1/23	604	3797	44	205	33	25	71	53	2.91	0.46	0.35	0.74	0.16	0.12	0.34	0.26
787	Trench 2 Horizon 1 Spit 2 15-JUN-22 sample of 80 one each	Syunik	2	1	2	15-Jun	185	7/1/23	455	4753	44	175	21	11	101	35	1.74	0.20	0.10	0.34	0.12	0.06	0.58	0.20
788	Trench 2 Horizon 1 Spit 2 15-JUN-22 sample of 80 one each	Gegham 1	2	1	2	15-Jun	186	7/1/23	708	4016	37	216	13	25	74	57	2.90	0.17	0.33	0.76	0.06	0.11	0.34	0.26
789	Trench 2 Horizon 1 Spit 2 15-JUN-22 sample of 80 one each	Kelbadjar	2	1	2	15-Jun	187	7/1/23	496	5040	38	158	16	11	112	35	1.40	0.14	0.10	0.31	0.10	0.07	0.71	0.22
790	Trench 2 Horizon 1 Spit 2 15-JUN-22 sample of 80 one each	Kelbadjar	2	1	2	15-Jun	189	7/1/23	580	5595	38	171	17	9	112	35	1.53	0.15	0.08	0.31	0.10	0.05	0.66	0.20
791	Trench 2 Horizon 1 Spit 2 15-JUN-22 sample of 80 one each	Gegham 1	2	1	2	15-Jun	190	7/1/23	768	4396	40	229	15	27	74	58	3.07	0.19	0.36	0.78	0.06	0.12	0.33	0.25
792	Trench 2 Horizon 1 Spit 2 15-JUN-22 sample of 80 one each	Gegham 1	2	1	2	15-Jun	191	7/1/23	575	3604	35	206	14	25	73	54	2.81	0.18	0.34	0.73	0.07	0.12	0.36	0.26
793	Trench 2 Horizon 1 Spit 2 15-JUN-22 sample of 80 one each	Kelbadjar	2	1	2	15-Jun	192	7/1/23	531	5504	41	166	17	11	113	36	1.47	0.15	0.10	0.32	0.10	0.07	0.68	0.22
794	Trench 2 Horizon 1 Spit 2 15-JUN-22 sample of 80 one each	Kelbadjar	2	1	2	15-Jun	193	7/1/23	508	5244	41	164	18	9	112	37	1.46	0.16	0.08	0.33	0.11	0.05	0.69	0.23
795	Trench 2 Horizon 1 Spit 2 15-JUN-22 sample of 80 one each	Kelbadjar	2	1	2	15-Jun	194	7/1/23	489	5052	36	155	16	11	115	33	1.34	0.13	0.09	0.29	0.10	0.07	0.74	0.22
796	Trench 2 Horizon 1 Spit 2 15-JUN-22 sample of 80 one each	Gegham 1	2	1	2	15-Jun	195	7/1/23	683	4048	36	214	15	27	75	59	2.84	0.19	0.35	0.79	0.07	0.12	0.35	0.28
797	Trench 2 Horizon 1 Spit 2 15-JUN-22 sample of 80 one each	Gegham 1	2	1	2	15-Jun	196	7/1/23	711	4044	35	212	14	25	77	54	2.74	0.17	0.32	0.70	0.06	0.12	0.36	0.25
798	Trench 2 Horizon 1 Spit 2 15-JUN-22 sample of 80 one each	Kelbadjar	2	1	2	15-Jun	197	7/1/23	494	5148	35	158	16	11	108	31	1.47	0.14	0.10	0.28	0.10	0.07	0.68	0.19
799	Trench 2 Horizon 1 Spit 2 15-JUN-22 sample of 80 one each	Gegham 1	2	1	2	15-Jun	198	7/1/23	731	4038	32	216	15	24	76	58	2.83	0.19	0.31	0.76	0.07	0.11	0.35	0.27
800	Trench 2 Horizon 1 Spit 2 15-JUN-22 sample of 80 one each	Gegham 1	2	1	2	15-Jun	199	7/1/23	739	4083	37	217	14	24	73	55	2.95	0.18	0.32	0.75	0.06	0.11	0.34	0.25
801	Trench 2 Horizon 1 Spit 2 15-JUN-22 sample of 80 one each	Gegham 1	2	1	2	15-Jun	200	7/1/23	753	4350	36	225	11	27	73	55	3.06	0.16	0.36	0.75	0.05	0.12	0.33	0.25
802	Trench 2 Horizon 1 Spit 2 15-JUN-22 sample of 80 one each	Kelbadjar	2	1	2	15-Jun	201	7/1/23	544	5089	40	159	17	11	107	36	1.49	0.15	0.11	0.34	0.10	0.07	0.67	0.23
803	Trench 2 Horizon 1 Spit 2 15-JUN-22 sample of 80 one each	Gegham 1	2	1	2	15-Jun	202	7/1/23	722	4118	36	210	15	25	74	51	2.82	0.19	0.33	0.69	0.07	0.12	0.35	0.24
804	Trench 2 Horizon 1 Spit 2 15-JUN-22 sample of 80 one each	Kelbadjar	2	1	2	15-Jun	203	7/1/23	539	5518	43	168	17	10	113	35	1.49	0.15	0.08	0.31	0.10	0.06	0.67	0.21
805	Trench 2 Horizon 1 Spit 2 15-JUN-22 sample of 80 one each	Gegham 1	2	1	2	15-Jun	204	7/1/23	759	4069	37	220	11	23	73	55	3.03	0.16	0.31	0.76	0.05	0.10	0.33	0.25
806	Trench 2 Horizon 1 Spit 2 15-JUN-22 sample of 80 one each	Kelbadjar	2	1	2	15-Jun	205	7/1/23	520	5437	42	164	16	12	107	35	1.54	0.15	0.12	0.33	0.09	0.08	0.65	0.21
807	Trench 2 Horizon 1 Spit 2 15-JUN-22 sample of 80 one each	Syunik	2	1	2	15-Jun	206	7/1/23	499	5192	35	182	21	11	104	37	1.75	0.20	0.10	0.36	0.11	0.06	0.57	0.21
808	Trench 2 Horizon 1 Spit 2 15-JUN-22 sample of 80 one each	Syunik	2	1	2	15-Jun	207	7/1/23	509	5153	37	179	20	12	100	37	1.79	0.20	0.12	0.37	0.11	0.07	0.56	0.21
809	Trench 2 Horizon 1 Spit 2 15-JUN-22 sample of 80 one each	Arteni	2	1	2	15-Jun	208	7/1/23	678	4586	42	141	26	27	86	31	1.65	0.30	0.32	0.36	0.18	0.19	0.61	0.22

810	Trench 2 Horizon 1 Spit 2 15-JUN-22 sample of 80 one each	Kelbadjar	2	1	2	15-Jun	209	7/1/23	496	4961	43	158	16	11	106	32	1.49	0.15	0.10	0.30	0.10	0.07	0.67	0.20
811	Trench 2 Horizon 1 Spit 2 15-JUN-22 sample of 80 one each	Gegham 1	2	1	2	15-Jun	210	7/1/23	631	3922	39	212	24	26	72	58	2.96	0.33	0.36	0.81	0.11	0.12	0.34	0.27
812	Trench 2 Horizon 1 Spit 2 15-JUN-22 sample of 80 one each	Gegham 1	2	1	2	15-Jun	211	7/1/23	670	3907	35	210	13	27	73	55	2.90	0.17	0.37	0.76	0.06	0.13	0.35	0.26
813	Trench 2 Horizon 1 Spit 2 15-JUN-22 sample of 80 one each	Kelbadjar	2	1	2	15-Jun	212	7/1/23	529	5403	39	162	17	11	123	35	1.32	0.13	0.09	0.28	0.10	0.07	0.76	0.21
814	Trench 2 Horizon 1 Spit 2 15-JUN-22 sample of 80 one each	Gegham 1	2	1	2	15-Jun	213	7/1/23	728	3969	37	216	14	25	74	53	2.90	0.18	0.33	0.71	0.06	0.11	0.34	0.24
815	Trench 2 Horizon 1 Spit 2 15-JUN-22 sample of 80 one each	Syunik	2	1	2	15-Jun	214	7/1/23	495	5081	35	183	21	11	104	36	1.76	0.20	0.10	0.35	0.11	0.06	0.57	0.20
816	Trench 2 Horizon 1 Spit 2 15-JUN-22 sample of 80 one each	Gegham 1	2	1	2	15-Jun	215	7/1/23	738	4021	38	218	13	25	73	57	2.97	0.17	0.34	0.77	0.06	0.11	0.34	0.26
817	Trench 2 Horizon 1 Spit 2 15-JUN-22 sample of 80 one each	Kelbadjar	2	1	2	15-Jun	216	7/1/23	662	6378	47	181	17	9	120	36	1.51	0.14	0.07	0.30	0.09	0.05	0.66	0.20
818	Trench 2 Horizon 1 Spit 2 15-JUN-22 sample of 80 one each	Gegham 1	2	1	2	15-Jun	217	7/1/23	663	3768	35	204	13	26	76	53	2.68	0.16	0.34	0.69	0.06	0.13	0.37	0.26
819	Trench 2 Horizon 1 Spit 2 15-JUN-22 sample of 80 one each	Gegham 1	2	1	2	15-Jun	218	7/1/23	739	4227	34	222	13	25	73	57	3.06	0.17	0.34	0.78	0.06	0.11	0.33	0.26
820	Trench 2 Horizon 1 Spit 2 15-JUN-22 sample of 80 one each	Kelbadjar	2	1	2	15-Jun	219	7/1/23	525	5506	45	165	18	12	112	36	1.47	0.16	0.11	0.32	0.11	0.07	0.68	0.22
821	Trench 2 Horizon 1 Spit 2 15-JUN-22 sample of 80 one each	Gegham 1	2	1	2	15-Jun	220	7/1/23	736	4236	36	223	15	27	75	57	2.96	0.19	0.35	0.75	0.07	0.12	0.34	0.25
822	Trench 2 Horizon 1 Spit 2 15-JUN-22 sample of 80 one each	Kelbadjar	2	1	2	15-Jun	221	7/1/23	572	5401	39	165	17	12	113	35	1.46	0.15	0.11	0.31	0.10	0.07	0.68	0.21
823	Trench 2 Horizon 1 Spit 2 15-JUN-22 sample of 80 one each	Kelbadjar	2	1	2	15-Jun	222	7/1/23	569	5365	41	164	18	11	113	36	1.45	0.15	0.09	0.32	0.11	0.06	0.69	0.22
824	Trench 2 Horizon 1 Spit 2 15-JUN-22 sample of 80 one each	Kelbadjar	2	1	2	15-Jun	223	7/1/23	524	5235	40	165	18	12	112	36	1.47	0.16	0.11	0.32	0.11	0.07	0.68	0.22
825	Trench 2 Horizon 1 Spit 2 15-JUN-22 sample of 80 one each	Gegham 1	2	1	2	15-Jun	224	7/1/23	673	3966	33	210	15	24	75	55	2.79	0.19	0.31	0.73	0.07	0.11	0.36	0.26
826	Trench 2 Horizon 1 Spit 2 15-JUN-22 sample of 80 one each	Kelbadjar	2	1	2	15-Jun	225	7/1/23	529	5551	40	166	18	11	112	36	1.48	0.16	0.10	0.32	0.11	0.07	0.67	0.22
827	Trench 2 Horizon 1 Spit 2 15-JUN-22 sample of 80 one each	Kelbadjar	2	1	2	15-Jun	226	7/1/23	551	5490	43	167	18	12	110	39	1.52	0.16	0.11	0.35	0.10	0.07	0.66	0.23
828	Trench 2 Horizon 1 Spit 2 15-JUN-22 sample of 80 one each	Gegham 1	2	1	2	15-Jun	227	7/1/23	717	3913	35	213	14	27	77	57	2.76	0.17	0.34	0.73	0.06	0.12	0.36	0.27
829	Trench 2 Horizon 1 Spit 2 15-JUN-22 sample of 80 one each	Kelbadjar	2	1	2	15-Jun	228	7/1/23	518	5286	40	162	18	11	112	35	1.44	0.16	0.10	0.31	0.11	0.07	0.69	0.21
830	Trench 2 Horizon 1 Spit 2 15-JUN-22 sample of 80 one each	Syunik	2	1	2	15-Jun	229	7/1/23	520	5447	39	190	21	11	108	39	1.76	0.19	0.11	0.36	0.11	0.06	0.57	0.20
831	Hor-2 Tr-2 Sp-1 16.06.22 small bag 1 of 3	Gegham 1	2	2	1	16-Jun	230	7/1/23	672	3944	34	209	13	22	69	53	3.04	0.18	0.32	0.76	0.06	0.10	0.33	0.25
833	Hor-2 Tr-2 Sp-1 16.06.22 small bag 2 of 3	Gegham 1	2	2	1	16-Jun	232	7/1/23	728	3860	35	210	14	24	70	55	3.01	0.19	0.34	0.79	0.06	0.11	0.33	0.26
835	Hor-2 Tr-2 Sp-1 16.06.22 small bag 3 of 3	Gegham 1	2	2	1	16-Jun	234	7/1/23	688	3849	37	213	14	27	73	54	2.94	0.19	0.37	0.74	0.06	0.12	0.34	0.25
837	Hor-2 Tr-2 Sp-1 16.06.22 big bag one each	Kelbadjar	2	2	1	16-Jun	236	7/1/23	567	5560	41	167	17	10	120	35	1.40	0.14	0.08	0.29	0.10	0.06	0.72	0.21
838	Hor-2 Tr-2 Sp-1 16.06.22 big bag one each	Syunik	2	2	1	16-Jun	237	7/1/23	480	5129	33	179	21	10	100	36	1.79	0.21	0.10	0.36	0.11	0.05	0.56	0.20
839	Hor-2 Tr-2 Sp-1 16.06.22 big bag one each	Kelbadjar	2	2	1	16-Jun	238	7/1/23	494	5132	43	156	17	10	108	32	1.44	0.15	0.09	0.29	0.11	0.06	0.70	0.20
840	Hor-2 Tr-2 Sp-1 16.06.22 big bag one each	Kelbadjar	2	2	1	16-Jun	239	7/1/23	547	5508	46	165	16	11	115	36	1.44	0.13	0.09	0.31	0.09	0.06	0.70	0.22
841	Hor-2 Tr-2 Sp-1 16.06.22 big bag one each	Gegham 1	2	2	1	16-Jun	240	7/1/23	739	4143	36	219	13	27	74	55	2.94	0.17	0.36	0.74	0.06	0.12	0.34	0.25
842	Hor-2 Tr-2 Sp-1 16.06.22 big bag one each	Syunik	2	2	1	16-Jun	241	7/1/23	506	5164	37	180	19	10	107	37	1.69	0.17	0.09	0.35	0.10	0.05	0.59	0.21
843	Hor-2 Tr-2 Sp-1 16.06.22 big bag one each	Kelbadjar	2	2	1	16-Jun	243	7/1/23	511	5273	39	162	20	13	114	36	1.42	0.17	0.12	0.32	0.12	0.08	0.71	0.22
844	Hor-2 Tr-2 Sp-1 16.06.22 big bag one each	Kelbadjar	2	2	1	16-Jun	244	7/1/23	534	5348	41	161	16	10	112	36	1.43	0.14	0.09	0.32	0.10	0.06	0.70	0.22
845	Hor-2 Tr-2 Sp-1 16.06.22 big bag one each	Kelbadjar	2	2	1	16-Jun	245	7/1/23	506	5183	44	160	17	11	110	33	1.45	0.15	0.10	0.30	0.10	0.07	0.69	0.21
846	Hor-2 Tr-2 Sp-1 16.06.22 big bag one each	Gegham 1	2	2	1	16-Jun	246	7/1/23	706	3858	35	213	11	26	73	55	2.90	0.16	0.35	0.75	0.05	0.12	0.34	0.26
847	Hor-2 Tr-2 Sp-1 16.06.22 big bag one each	Gegham 1	2	2	1	16-Jun	247	7/1/23	717	3820	33	207	11	25	72	55	2.89	0.16	0.34	0.77	0.06	0.12	0.35	0.27
848	Hor-2 Tr-2 Sp-1 16.06.22 big bag one each	Kelbadjar	2	2	1	16-Jun	249	7/1/23	557	5546	43	166	19	11	120	36	1.39	0.15	0.09	0.30	0.11	0.06	0.72	0.22
849	Hor-2 Tr-2 Sp-1 16.06.22 big bag one each	Kelbadjar	2	2	1	16-Jun	250	7/1/23	498	5123	40	162	18	11	108	35	1.50	0.16	0.10	0.32	0.11	0.07	0.67	0.21
850	Hor-2 Tr-2 Sp-1 16.06.22 big bag one each	Arteni	2	2	1	16-Jun	251	7/1/23	621	4267	38	130	30	26	85	29	1.53	0.35	0.30	0.34	0.23	0.20	0.66	0.23
851	Hor-2 Tr-2 Sp-1 16.06.22 big bag one each	Gegham 1	2	2	1	16-Jun	252	7/1/23	751	4088	35	218	13	25	71	57	3.08	0.18	0.35	0.80	0.06	0.11	0.32	0.26
852	Hor-2 Tr-2 Sp-1 16.06.22 big bag one each	Syunik	2	2	1	16-Jun	253	7/1/23	537	4548	40	197	11	8	91	39	2.15	0.13	0.08	0.42	0.06	0.04	0.47	0.20
853	Hor-2 Tr-2 Sp-1 16.06.22 big bag one each	Gegham 1	2	2	1	16-Jun	254	7/1/23	782	4258	35	226	14	27	73	54	3.11	0.19	0.38	0.74	0.06	0.12	0.32	0.24
854	Hor-2 Tr-2 Sp-1 16.06.22 big bag one each	Kelbadjar	2	2	1	16-Jun	255	7/1/23	539	5575	40	168	18	11	116	35	1.45	0.15	0.10	0.30	0.10	0.07	0.69	0.21
855	Hor-2 Tr-2 Sp-1 16.06.22 big bag one each	Arteni	2	2	1	16-Jun	256	7/1/23	604	4091	39	136	24	27	79	31	1.72	0.30	0.35	0.39	0.17	0.20	0.58	0.22
856	Hor-2 Tr-2 Sp-1 16.06.22 big bag one each	Gegham 1	2	2	1	16-Jun	257	7/1/23	705	3973	35	216	14	24	73	58	2.94	0.18	0.32	0.79	0.06	0.11	0.34	0.27
857	Hor-2 Tr-2 Sp-1 16.06.22 big bag one each	Kelbadjar	2	2	1	16-Jun	258	7/1/23	478	5301	42	156	17	11	108	35	1.44	0.15	0.10	0.32	0.11	0.07	0.70	0.22
858	Hor-2 Tr-2 Sp-1 16.06.22 big bag one each	Gegham 1	2	2	1	16-Jun	259	7/1/23	693	3722	30	208	13	26	69	55	3.03	0.18	0.37	0.80	0.06	0.12	0.33	0.27
859	Hor-2 Tr-2 Sp-1 16.06.22 big bag one each	Syunik	2	2	1	16-Jun	260	7/1/23	488	5127	36	183	20	11	106	35	1.73	0.18	0.11	0.33	0.11	0.06	0.58	0.19
860	Hor-2 Tr-2 Sp-1 16.06.22 big bag one each	Kelbadjar	2	2	1	16-Jun	261	7/1/23	503	5313	39	161	16	11	110	35	1.46	0.14	0.10	0.31	0.10	0.07	0.69	0.22
861	Hor-2 Tr-2 Sp-1 16.06.22 big bag one each	Syunik	2	2	1	16-Jun	263	7/1/23	488	5283	36	183	19	12	105	36	1.75	0.18	0.12	0.34	0.10	0.07	0.57	0.20
862	Hor-2 Tr-2 Sp-1 16.06.22 big bag one each	Gegham 1	2	2	1	16-Jun	264	7/1/23	686	3803	35	208	14	26	71	53	2.95	0.19	0.36	0.74	0.06	0.12	0.34	0.25
863	Hor-2 Tr-2 Sp-1 16.06.22 big bag one each	Kelbadjar	2	2	1	16-Jun	265	7/1/23	496	5012	35	154	16	11	105	35	1.47	0.15	0.10	0.33	0.10	0.07	0.68	0.23

864	Hor-2 Tr-2 Sp-1 16.06.22 big bag one each	Syunik	2	2	1	16-Jun	266	7/1/23	520	5535	38	188	23	11	108	37	1.75	0.21	0.10	0.35	0.12	0.06	0.57	0.20
865	Hor-2 Tr-2 Sp-1 16.06.22 big bag one each	Kelbadjar	2	2	1	16-Jun	268	7/1/23	511	5508	43	165	18	11	114	35	1.45	0.15	0.09	0.30	0.11	0.06	0.69	0.21
866	Hor-2 Tr-2 Sp-1 16.06.22 big bag one each	Syunik	2	2	1	16-Jun	269	7/1/23	520	5389	35	185	19	12	107	39	1.73	0.17	0.12	0.36	0.10	0.07	0.58	0.21
867	Hor-2 Tr-2 Sp-1 16.06.22 big bag one each	Kelbadjar	2	2	1	16-Jun	270	7/1/23	537	5193	40	162	17	12	108	32	1.50	0.15	0.12	0.30	0.10	0.08	0.67	0.20
868	Hor-2 Tr-2 Sp-1 16.06.22 big bag one each	Gegham 1	2	2	1	16-Jun	271	7/1/23	692	3829	35	206	14	26	72	54	2.88	0.19	0.36	0.75	0.07	0.12	0.35	0.26
869	Hor-2 Tr-2 Sp-1 16.06.22 big bag one each	Gegham 1	2	2	1	16-Jun	272	7/1/23	751	4158	37	222	14	25	73	57	3.06	0.19	0.34	0.78	0.06	0.11	0.33	0.26
870	Hor-2 Tr-2 Sp-1 16.06.22 big bag one each	Gegham 1	2	2	1	16-Jun	273	7/1/23	729	4395	42	218	17	25	73	57	3.00	0.23	0.34	0.78	0.08	0.11	0.33	0.26
871	Hor-2 Tr-2 Sp-1 16.06.22 big bag one each	Arteni	2	2	1	16-Jun	274	7/1/23	549	4794	40	123	44	23	91	26	1.36	0.48	0.25	0.29	0.36	0.19	0.74	0.22
872	Hor-2 Tr-2 Sp-1 16.06.22 big bag one each	Gegham 1	2	2	1	16-Jun	275	7/1/23	665	3907	34	209	16	24	70	51	3.00	0.22	0.34	0.73	0.07	0.11	0.33	0.24
873	Hor-2 Tr-2 Sp-1 16.06.22 big bag one each	Kelbadjar	2	2	1	16-Jun	276	7/1/23	523	5484	40	164	16	11	115	37	1.42	0.13	0.10	0.33	0.09	0.07	0.70	0.23
874	Hor-2 Tr-2 Sp-1 16.06.22 big bag one each	Gegham 1	2	2	1	16-Jun	277	7/1/23	637	3929	32	209	16	27	73	57	2.88	0.21	0.37	0.78	0.07	0.13	0.35	0.27
875	Hor-2 Tr-2 Sp-1 16.06.22 big bag one each	Syunik	2	2	1	16-Jun	278	7/1/23	509	5273	38	184	20	9	106	35	1.74	0.18	0.08	0.33	0.11	0.05	0.57	0.19
876	Hor-2 Tr-2 Sp-1 16.06.22 big bag one each	Arteni	2	2	1	16-Jun	279	7/1/23	652	4032	46	138	20	27	80	32	1.73	0.24	0.34	0.40	0.14	0.20	0.58	0.23
877	Hor-2 Tr-2 Sp-1 16.06.22 big bag one each	Gegham 1	2	2	1	16-Jun	280	7/1/23	661	3844	31	206	15	26	73	55	2.81	0.20	0.35	0.75	0.07	0.12	0.36	0.27
878	Hor-2 Tr-2 Sp-1 16.06.22 big bag one each	Syunik	2	2	1	16-Jun	281	7/1/23	516	5213	36	186	19	12	104	36	1.79	0.18	0.12	0.35	0.10	0.07	0.56	0.19
879	Hor-2 Tr-2 Sp-1 16.06.22 big bag one each	Kelbadjar	2	2	1	16-Jun	282	7/1/23	514	5259	40	161	17	10	108	36	1.49	0.15	0.09	0.34	0.10	0.06	0.67	0.22
880	Hor-2 Tr-2 Sp-1 16.06.22 big bag one each	Kelbadjar	2	2	1	16-Jun	283	7/1/23	551	5699	45	169	20	11	116	36	1.46	0.17	0.09	0.31	0.12	0.06	0.69	0.21
881	Hor-2 Tr-2 Sp-1 16.06.22 big bag one each	Gegham 1	2	2	1	16-Jun	284	7/1/23	715	4101	33	217	13	25	73	54	2.99	0.17	0.34	0.74	0.06	0.11	0.33	0.25
882	Hor-2 Tr-2 Sp-1 16.06.22 big bag one each	Kelbadjar	2	2	1	16-Jun	285	7/1/23	557	5267	46	164	17	11	121	33	1.35	0.14	0.09	0.28	0.10	0.06	0.74	0.20
883	Hor-2 Tr-2 Sp-1 16.06.22 big bag one each	Gegham 1	2	2	1	16-Jun	286	7/1/23	609	3480	28	195	14	25	71	54	2.75	0.19	0.35	0.76	0.07	0.13	0.36	0.28
884	Hor-2 Tr-2 Sp-1 16.06.22 big bag one each	Kelbadjar	2	2	1	16-Jun	287	7/1/23	530	5819	45	169	19	11	119	36	1.42	0.16	0.09	0.30	0.11	0.06	0.70	0.21
885	Hor-2 Tr-2 Sp-1 16.06.22 big bag one each	Kelbadjar	2	2	1	16-Jun	288	7/1/23	521	5452	45	164	18	11	111	35	1.48	0.16	0.10	0.31	0.11	0.07	0.68	0.21
886	Hor-2 Tr-2 Sp-1 16.06.22 big bag one each	Gegham 1	2	2	1	16-Jun	289	7/1/23	712	4162	35	216	14	26	74	55	2.90	0.18	0.34	0.74	0.06	0.12	0.34	0.26
887	Hor-2 Tr-2 Sp-1 16.06.22 big bag one each	Gegham 1	2	2	1	16-Jun	290	7/1/23	743	4139	32	219	16	24	73	54	3.02	0.21	0.33	0.74	0.07	0.11	0.33	0.25
888	Hor-2 Tr-2 Sp-1 16.06.22 big bag one each	Syunik	2	2	1	16-Jun	291	7/1/23	524	5371	39	186	25	11	108	35	1.73	0.23	0.11	0.32	0.13	0.06	0.58	0.19
889	Hor-2 Tr-2 Sp-1 16.06.22 big bag one each	Kelbadjar	2	2	1	16-Jun	292	7/1/23	499	5214	40	162	19	10	108	35	1.50	0.17	0.09	0.32	0.11	0.06	0.67	0.21
890	Hor-2 Tr-2 Sp-1 16.06.22 big bag one each	Gegham 1	2	2	1	16-Jun	293	7/1/23	685	3921	32	213	13	24	69	54	3.10	0.18	0.34	0.78	0.06	0.11	0.32	0.25
891	Hor-2 Tr-2 Sp-1 16.06.22 big bag one each	Arteni	2	2	1	16-Jun	294	7/1/23	564	4185	37	127	28	26	80	28	1.58	0.34	0.32	0.35	0.22	0.20	0.63	0.22
892	Hor-2 Tr-2 Sp-1 16.06.22 big bag one each	Gegham 1	2	2	1	16-Jun	295	7/1/23	705	3920	34	212	13	24	73	55	2.89	0.17	0.32	0.75	0.06	0.11	0.35	0.26
893	Hor-2 Tr-2 Sp-1 16.06.22 big bag one each	Kelbadjar	2	2	1	16-Jun	296	7/1/23	530	5220	36	164	18	12	117	36	1.40	0.15	0.11	0.31	0.11	0.08	0.72	0.22
894	Hor-2 Tr-2 Sp-1 16.06.22 big bag one each	Arteni	2	2	1	16-Jun	297	7/1/23	685	4343	40	144	20	31	85	35	1.70	0.23	0.37	0.41	0.14	0.22	0.59	0.24
895	Hor-2 Tr-2 Sp-1 16.06.22 big bag one each	Gegham 1	2	2	1	16-Jun	298	7/1/23	640	4003	34	205	19	24	70	53	2.95	0.27	0.34	0.75	0.09	0.12	0.34	0.26
896	Hor-2 Tr-2 Sp-1 16.06.22 big bag one each	Gegham 1	2	2	1	16-Jun	299	7/1/23	727	4246	37	213	15	25	74	57	2.86	0.19	0.33	0.76	0.07	0.12	0.35	0.27
897	Hor-2 Tr-2 Sp-1 16.06.22 big bag one each	Kelbadjar	2	2	1	16-Jun	300	7/1/23	515	5006	36	157	16	10	105	37	1.50	0.15	0.09	0.36	0.10	0.06	0.67	0.24
898	Hor-2 Tr-2 Sp-1 16.06.22 big bag one each	Gegham 1	2	2	1	16-Jun	301	7/1/23	753	4023	37	217	11	23	73	54	2.95	0.16	0.31	0.73	0.05	0.10	0.34	0.25
899	Hor-2 Tr-2 Sp-1 16.06.22 big bag one each	Gegham 1	2	2	1	16-Jun	303	7/1/23	645	3904	31	205	14	26	72	54	2.87	0.19	0.36	0.75	0.07	0.12	0.35	0.26
900	Hor-2 Tr-2 Sp-1 16.06.22 big bag one each	Gegham 1	2	2	1	16-Jun	304	7/1/23	729	4143	37	215	14	24	72	54	3.00	0.19	0.33	0.75	0.06	0.11	0.33	0.25
901	Hor-2 Tr-2 Sp-1 16.06.22 big bag one each	Kelbadjar	2	2	1	16-Jun	305	7/1/23	575	5724	43	169	19	10	114	36	1.48	0.16	0.08	0.32	0.11	0.06	0.67	0.21
902	Hor-2 Tr-2 Sp-1 16.06.22 big bag one each	Kelbadjar	2	2	1	16-Jun	306	7/1/23	524	5674	44	168	19	11	114	37	1.48	0.16	0.10	0.33	0.11	0.07	0.68	0.22
903	Hor-2 Tr-2 Sp-1 16.06.22 big bag one each	Kelbadjar	2	2	1	16-Jun	307	7/1/23	478	5054	37	157	17	11	114	36	1.37	0.14	0.10	0.32	0.11	0.07	0.73	0.23
904	Hor-2 Tr-2 Sp-1 16.06.22 big bag one each	Hatis	2	2	1	16-Jun	308	7/1/23	531	6571	35	116	114	20	99	21	1.17	1.15	0.20	0.21	0.98	0.17	0.85	0.18
905	Hor-2 Tr-2 Sp-1 16.06.22 big bag one each	Gegham 1	2	2	1	16-Jun	309	7/1/23	671	4263	34	210	16	25	78	54	2.69	0.20	0.32	0.69	0.07	0.12	0.37	0.26
906	Hor-2 Tr-2 Sp-1 16.06.22 big bag one each	Gegham 1	2	2	1	16-Jun	310	7/1/23	739	4744	37	225	20	25	72	55	3.14	0.27	0.34	0.77	0.09	0.11	0.32	0.25
907	Hor-2 Tr-2 Sp-1 16.06.22 big bag one each	Gegham 1	2	2	1	16-Jun	311	7/1/23	690	4029	35	208	16	27	75	55	2.76	0.21	0.35	0.73	0.07	0.13	0.36	0.27
908	Hor-2 Tr-2 Sp-1 16.06.22 big bag one each	Gegham 1	2	2	1	16-Jun	312	7/1/23	724	4106	34	217	14	27	73	57	2.95	0.18	0.36	0.77	0.06	0.12	0.34	0.26
909	Hor-2 Tr-2 Sp-1 16.06.22 big bag one each	Syunik	2	2	1	16-Jun	313	7/1/23	585	6117	42	199	21	11	108	37	1.84	0.19	0.11	0.34	0.10	0.06	0.54	0.19
910	Hor-2 Tr-2 Sp-1 16.06.22 big bag one each	Kelbadjar	2	2	1	16-Jun	314	7/1/23	551	5615	43	167	18	11	115	35	1.45	0.15	0.09	0.30	0.10	0.06	0.69	0.21
911	Hor-2 Tr-2 Sp-1 16.06.22 big bag one each	Kelbadjar	2	2	1	16-Jun	315	7/1/23	514	5509	41	164	16	13	117	35	1.41	0.13	0.11	0.30	0.09	0.08	0.71	0.21
912	Hor-2 Tr-2 Sp-1 16.06.22 big bag one each	Arteni	2	2	1	16-Jun	316	7/1/23	565	4500	37	123	37	27	90	29	1.37	0.41	0.30	0.33	0.30	0.22	0.73	0.24
913	Hor-2 Tr-2 Sp-1 16.06.22 big bag one each	Gegham 1	2	2	1	16-Jun	317	7/1/23	751	4095	35	217	14	24	72	54	3.03	0.19	0.33	0.75	0.06	0.11	0.33	0.25
914	Hor-2 Tr-2 Sp-1 16.06.22 big bag one each	Kelbadjar	2	2	1	16-Jun	318	7/1/23	503	4971	38	163	16	11	115	37	1.41	0.13	0.10	0.33	0.10	0.07	0.71	0.23

915	Hor-2 Tr-2 Sp-1 16.06.22 big bag one each	Syunik	2	2	1		16-Jun	319	7/1/23	544	5420	39	186	22	11	104	37	1.79	0.21	0.11	0.36	0.12	0.06	0.56	0.20
916	Hor-2 Tr-2 Sp-1 16.06.22 big bag one each	Gegham 1	2	2	1		16-Jun	320	7/1/23	748	4153	31	218	15	26	73	57	3.00	0.20	0.35	0.78	0.07	0.12	0.33	0.26
917	Hor-2 Tr-2 Sp-1 16.06.22 big bag one each	Gegham 1	2	2	1		16-Jun	321	7/1/23	729	4058	37	216	14	25	71	53	3.06	0.19	0.35	0.74	0.06	0.11	0.33	0.24
918	Hor-2 Tr-2 Sp-1 16.06.22 big bag one each	Syunik	2	2	1		16-Jun	322	7/1/23	488	5416	39	180	28	10	100	36	1.80	0.28	0.10	0.36	0.15	0.05	0.56	0.20
919	Hor-2 Tr-2 Sp-1 16.06.22 big bag one each	Gegham 1	2	2	1		16-Jun	323	7/1/23	620	3870	36	205	14	27	73	53	2.79	0.18	0.36	0.71	0.07	0.13	0.36	0.26
920	Hor-2 Tr-2 Sp-1 16.06.22 big bag one each	Kelbadjar	2	2	1		16-Jun	324	7/1/23	535	5472	42	169	17	12	112	35	1.51	0.15	0.11	0.31	0.10	0.07	0.66	0.20
921	Hor-2 Tr-2 Sp-1 16.06.22 big bag one each	Gegham 1	2	2	1		16-Jun	325	7/1/23	751	4361	35	225	15	25	76	55	2.94	0.19	0.32	0.72	0.06	0.11	0.34	0.25
922	Hor-2 Tr-2 Sp-1 16.06.22 big bag one each	Gegham 1	2	2	1		16-Jun	326	7/1/23	697	4188	36	221	14	23	75	55	2.93	0.18	0.30	0.73	0.06	0.10	0.34	0.25
923	Hor-2 Tr-2 Sp-1 16.06.22 big bag one each	Gegham 1	2	2	1		16-Jun	327	7/1/23	729	4267	42	220	14	26	76	55	2.88	0.18	0.34	0.72	0.06	0.12	0.35	0.25
924	Hor-2 Tr-2 Sp-1 16.06.22 big bag one each	Gegham 1	2	2	1		16-Jun	328	7/1/23	691	3807	37	206	13	25	73	54	2.81	0.17	0.34	0.73	0.06	0.12	0.36	0.26
925	Hor-2 Tr-2 Sp-1 16.06.22 big bag one each	Gegham 1	2	2	1		16-Jun	329	7/1/23	700	4072	35	223	14	26	73	57	3.07	0.19	0.35	0.78	0.06	0.11	0.33	0.25
926	Hor-2 Tr-2 Sp-1 16.06.22 big bag one each	Gegham 1	2	2	1		16-Jun	330	7/1/23	676	3703	32	207	13	26	73	54	2.86	0.17	0.35	0.74	0.06	0.12	0.35	0.26
927	Hor-2 Tr-2 Sp-1 16.06.22 big bag one each	Gegham 1	2	2	1		16-Jun	331	7/1/23	717	3946	36	219	15	27	75	54	2.90	0.19	0.35	0.71	0.07	0.12	0.34	0.25
928	Hor-2 Tr-2 Sp-1 16.06.22 big bag one each	Gegham 1	2	2	1		16-Jun	332	7/1/23	721	4768	47	219	22	25	72	57	3.06	0.30	0.34	0.79	0.10	0.11	0.33	0.26
929	Hor-2 Tr-2 Sp-1 16.06.22 big bag one each	Kelbadjar	2	2	1		16-Jun	333	7/1/23	521	5247	40	162	17	10	108	39	1.49	0.15	0.09	0.36	0.10	0.06	0.67	0.24
930	Hor-2 Tr-2 Sp-1 16.06.22 big bag one each	Syunik	2	2	1		16-Jun	334	7/1/23	526	5298	36	186	20	11	101	36	1.84	0.19	0.10	0.36	0.11	0.06	0.54	0.19
931	Hor-2 Tr-2 Sp-1 16.06.22 big bag one each	Kelbadjar	2	2	1		16-Jun	335	7/1/23	533	5453	43	169	19	11	111	35	1.52	0.17	0.10	0.31	0.11	0.07	0.66	0.20
932	Hor-2 Tr-2 Sp-1 16.06.22 big bag one each	Kelbadjar	2	2	1		16-Jun	336	7/1/23	536	5385	42	165	17	11	131	35	1.26	0.13	0.08	0.26	0.10	0.06	0.79	0.21
933	Hor-2 Tr-2 Sp-1 16.06.22 big bag one each	Arteni	2	2	1		16-Jun	337	7/1/23	734	4781	47	149	24	28	90	33	1.66	0.26	0.32	0.37	0.16	0.19	0.60	0.22
934	Hor-2 Tr-2 Sp-1 16.06.22 big bag one each	Gegham 1	2	2	1		16-Jun	338	7/1/23	753	4324	34	231	15	24	74	54	3.10	0.19	0.32	0.72	0.06	0.10	0.32	0.23
935	Hor-2 Tr-2 Sp-1 16.06.22 big bag one each	Gegham 1	2	2	1		16-Jun	339	7/1/23	720	4259	39	231	14	24	71	54	3.26	0.19	0.34	0.76	0.06	0.10	0.31	0.23
936	Hor-2 Tr-2 Sp-1 16.06.22 big bag one each	Gegham 1	2	2	1		16-Jun	340	7/1/23	708	3871	33	213	13	27	74	59	2.86	0.17	0.36	0.80	0.06	0.12	0.35	0.28
937	Hor-2 Tr-2 Sp-1 16.06.22 big bag one each	Gegham 1	2	2	1		16-Jun	341	7/1/23	747	4325	35	221	17	23	75	50	2.93	0.22	0.30	0.66	0.07	0.10	0.34	0.23
938	Hor-2 Tr-2 Sp-1 16.06.22 big bag one each	Gegham 1	2	2	1		16-Jun	342	7/1/23	726	3965	32	212	13	25	73	53	2.92	0.17	0.34	0.72	0.06	0.12	0.34	0.25
939	Hor-2 Tr-2 Sp-1 16.06.22 big bag one each	Gegham 1	2	2	1		16-Jun	343	7/1/23	702	3898	34	215	14	25	75	55	2.85	0.18	0.33	0.73	0.06	0.11	0.35	0.26
940	Hor-2 Tr-2 Sp-1 16.06.22 big bag one each	Gegham 1	2	2	1		16-Jun	344	7/1/23	670	3890	35	209	13	25	74	57	2.81	0.17	0.33	0.76	0.06	0.12	0.36	0.27
941	Hor-2 Tr-2 Sp-1 16.06.22 big bag one each	Gegham 1	2	2	1		16-Jun	345	7/1/23	736	4350	35	225	15	26	74	55	3.02	0.19	0.34	0.74	0.06	0.11	0.33	0.25
942	Hor-2 Tr-2 Sp-1 16.06.22 big bag one each	Gegham 1	2	2	1		16-Jun	346	7/1/23	772	4202	38	228	13	25	73	55	3.14	0.17	0.34	0.76	0.05	0.11	0.32	0.24
943	Hor-2 Tr-2 Sp-1 16.06.22 big bag one each	Gegham 1	2	2	1		16-Jun	347	7/1/23	668	3836	33	210	14	26	74	55	2.82	0.18	0.34	0.74	0.06	0.12	0.35	0.26
944	Hor-2 Tr-2 Sp-1 16.06.22 big bag one each	Gegham 1	2	2	1		16-Jun	348	7/1/23	686	3902	35	213	13	27	74	55	2.86	0.17	0.36	0.74	0.06	0.12	0.35	0.26
945	Hor-2 Tr-2 Sp-1 16.06.22 big bag one each	Gegham 1	2	2	1		16-Jun	349	7/1/23	691	3976	35	209	15	26	72	54	2.92	0.20	0.36	0.75	0.07	0.12	0.34	0.26
946	Hor-2 Tr-2 Sp-1 16.06.22 big bag one each	Gegham 1	2	2	1		16-Jun	350	7/1/23	830	4593	38	231	15	27	78	58	2.95	0.19	0.35	0.74	0.06	0.12	0.34	0.25
947	Hor-2 Tr-2 Sp-1 16.06.22 big bag one each	Gegham 1	2	2	1		16-Jun	351	7/1/23	784	4301	35	226	14	26	79	58	2.85	0.17	0.32	0.73	0.06	0.11	0.35	0.26
948	Hor-2 Tr-2 Sp-1 16.06.22 big bag one each	Gegham 1	2	2	1		16-Jun	352	7/1/23	753	4469	39	215	20	27	73	54	2.96	0.27	0.38	0.74	0.09	0.13	0.34	0.25
949	Hor-2 Tr-2 Sp-1 16.06.22 big bag one each	Gegham 2	2	2	1		16-Jun	353	7/1/23	840	6400	55	155	43	28	94	29	1.64	0.45	0.30	0.31	0.28	0.18	0.61	0.19
950	Hor-2 Tr-2 Sp-1 16.06.22 big bag one each	Kelbadjar	2	2	1		16-Jun	354	7/1/23	585	6105	44	178	18	12	119	39	1.50	0.15	0.10	0.33	0.10	0.07	0.67	0.22
951	Hor-2 Tr-2 Sp-1 16.06.22 big bag one each	Kelbadjar	2	2	1		16-Jun	355	7/1/23	518	5448	40	168	18	11	119	35	1.42	0.15	0.09	0.29	0.10	0.06	0.71	0.21
952	Hor-2 Tr-2 Sp-1 16.06.22 big bag one each	Gegham 1	2	2	1		16-Jun	356	7/1/23	797	4591	39	242	15	24	74	57	3.25	0.19	0.32	0.76	0.06	0.10	0.31	0.23
953	Hor-2 Tr-2 Sp-1 16.06.22 big bag one each	Kelbadjar	2	2	1		16-Jun	357	7/1/23	524	5346	42	168	20	13	109	35	1.54	0.18	0.12	0.32	0.12	0.08	0.65	0.21
954	Hor-2 Tr-2 Sp-1 16.06.22 big bag one each	Gegham 1	2	2	1		16-Jun	358	7/1/23	682	4017	35	216	14	24	78	58	2.76	0.17	0.30	0.74	0.06	0.11	0.36	0.27
955	Tr-2 Hor-0 Un-A5 1 of 8	Kelbadjar	2	0	-	A5	-	1	7/2/23	491	4844	37	158	17	11	106	35	1.49	0.16	0.11	0.33	0.10	0.07	0.67	0.22
956	Tr-2 Hor-0 Un-A5 2 of 8	Gegham 1	2	0	-	A5	-	3	7/2/23	685	3780	35	214	14	25	74	57	2.88	0.18	0.33	0.76	0.06	0.12	0.35	0.26
957	Tr-2 Hor-0 Un-A5 3 of 8	Gegham 1	2	0	-	A5	-	5	7/2/23	724	4057	35	223	14	26	77	58	2.88	0.17	0.33	0.75	0.06	0.11	0.35	0.26
958	Tr-2 Hor-0 Un-A5 4 of 8	Gegham 1	2	0	-	A5	-	7	7/2/23	675	3845	36	225	13	25	73	55	3.06	0.17	0.34	0.75	0.06	0.11	0.33	0.25
959	Tr-2 Hor-0 Un-A5 5 of 8	Gegham 1	2	0	-	A5	-	9	7/2/23	747	4059	34	220	15	27	75	55	2.92	0.19	0.36	0.73	0.07	0.12	0.34	0.25
960	Tr-2 Hor-0 Un-A5 6 of 8	Gegham 1	2	0	-	A5	-	11	7/2/23	910	4916	47	250	15	26	77	58	3.24	0.19	0.33	0.75	0.06	0.10	0.31	0.23
961	Tr-2 Hor-0 Un-A5 7 of 8	Kelbadjar	2	0	-	A5	-	13	7/2/23	520	5202	39	161	18	11	110	39	1.46	0.16	0.10	0.35	0.11	0.07	0.69	0.24
962	Tr-2 Hor-0 Un-A5 8 of 8	Gegham 1	2	0	-	A5	-	15	7/2/23	712	3845	38	214	15	27	79	57	2.70	0.18	0.34	0.72	0.07	0.12	0.37	0.26
963	Tr-2 Hor-0 Un-A1 30.06.23 1 of 8	Gegham 1	2	0	-	A1	6/30/23	18	7/2/23	662	3907	32	210	14	25	70	54	3.01	0.19	0.35	0.77	0.06	0.12	0.33	0.26
964	Tr-2 Hor-0 Un-A1 30.06.23 2 of 8	Kelbadjar	2	0	-	A1	6/30/23	19	7/2/23	435	4929	36	164	27	11	109	33	1.49	0.24	0.10	0.30	0.16	0.07	0.67	0.20
965	Tr-2 Hor-0 Un-A1 30.06.23 3 of 8	Syunik	2	0	-	A1	6/30/23	21	7/2/23	491	4998	42	179	23	10	100	35	1.79	0.23	0.10	0.35	0.13	0.05	0.56	0.19

966	Tr-2 Hor-0 Un-A1 30.06.23 4 of 8	Kelbadjar	2	0	-	A1	6/30/23	23	7/2/23	503	4989	40	161	20	12	110	36	1.46	0.18	0.11	0.33	0.12	0.08	0.69	0.22
967	Tr-2 Hor-0 Un-A1 30.06.23 5 of 8	Gegham 1	2	0	-	A1	6/30/23	25	7/2/23	824	4390	33	226	13	27	76	57	2.96	0.16	0.36	0.74	0.06	0.12	0.34	0.25
968	Tr-2 Hor-0 Un-A1 30.06.23 6 of 8	Kelbadjar	2	0	-	A1	6/30/23	27	7/2/23	499	5043	40	159	16	11	108	36	1.48	0.14	0.11	0.34	0.10	0.07	0.68	0.23
969	Tr-2 Hor-0 Un-A1 30.06.23 7 of 8	Kelbadjar	2	0	-	A1	6/30/23	29	7/2/23	565	5473	40	164	16	11	108	35	1.52	0.14	0.11	0.32	0.09	0.07	0.66	0.21
970	Tr-2 Hor-0 Un-A1 30.06.23 8 of 8	Kelbadjar	2	0	-	A1	6/30/23	31	7/2/23	591	5768	46	172	16	10	110	39	1.56	0.14	0.09	0.35	0.09	0.06	0.64	0.23
971	Tr.2 UN-A2-3 H2 S2 30.06.23 one each	Gegham 1	2	2	2	A2-A3	6/30/23	33	7/2/23	766	4171	34	218	14	27	73	53	2.97	0.18	0.36	0.71	0.06	0.12	0.34	0.24
972	Tr.2 UN-A2-3 H2 S2 30.06.23 one each	Kelbadjar	2	2	2	A2-A3	6/30/23	34	7/2/23	528	5608	41	166	18	11	112	40	1.48	0.16	0.10	0.36	0.11	0.07	0.67	0.24
973	Tr.2 UN-A2-3 H2 S2 30.06.23 one each	Gegham 1	2	2	2	A2-A3	6/30/23	35	7/2/23	767	4155	35	224	14	26	72	54	3.13	0.19	0.36	0.75	0.06	0.11	0.32	0.24
974	Tr.2 UN-A2-3 H2 S2 30.06.23 one each	Gegham 1	2	2	2	A2-A3	6/30/23	36	7/2/23	818	4406	36	238	13	26	71	55	3.37	0.18	0.36	0.78	0.05	0.11	0.30	0.23
975	Tr.2 UN-A2-3 H2 S2 30.06.23 one each	Kelbadjar	2	2	2	A2-A3	6/30/23	37	7/2/23	493	5404	39	162	18	11	110	35	1.46	0.16	0.10	0.31	0.11	0.07	0.68	0.21
976	Tr.2 UN-A2-3 H2 S2 30.06.23 one each	Gegham 1	2	2	2	A2-A3	6/30/23	38	7/2/23	701	4127	37	216	15	27	75	54	2.87	0.19	0.35	0.71	0.07	0.12	0.35	0.25
977	Tr.2 UN-A2-3 H2 S2 30.06.23 one each	Kelbadjar	2	2	2	A2-A3	6/30/23	39	7/2/23	560	5924	43	175	17	11	113	36	1.55	0.15	0.09	0.32	0.09	0.06	0.65	0.21
978	Tr.2 UN-A2-3 H2 S2 30.06.23 one each	Syunik	2	2	2	A2-A3	6/30/23	40	7/2/23	496	5227	40	189	19	12	108	36	1.76	0.17	0.12	0.34	0.10	0.07	0.57	0.19
979	Tr.2 UN-A2-3 H2 S2 30.06.23 one each	Kelbadjar	2	2	2	A2-A3	6/30/23	41	7/2/23	555	5707	42	170	17	11	113	36	1.50	0.15	0.10	0.32	0.10	0.07	0.66	0.21
980	Tr.2 UN-A2-3 H2 S2 30.06.23 one each	Gegham 1	2	2	2	A2-A3	6/30/23	42	7/2/23	717	3896	32	214	13	27	75	57	2.84	0.17	0.36	0.75	0.06	0.13	0.35	0.26
981	Tr.2 UN-A2-3 H2 S2 30.06.23 one each	Kelbadjar	2	2	2	A2-A3	6/30/23	43	7/2/23	479	5238	38	161	17	11	115	36	1.40	0.14	0.09	0.31	0.10	0.07	0.72	0.22
982	Tr.2 UN-A2-3 H2 S2 30.06.23 one each	Arteni	2	2	2	A2-A3	6/30/23	44	7/2/23	793	5858	53	153	41	27	91	29	1.69	0.45	0.29	0.32	0.27	0.17	0.59	0.19
983	Tr.2 UN-A2-3 H2 S2 30.06.23 one each	Kelbadjar	2	2	2	A2-A3	6/30/23	45	7/2/23	570	5718	46	169	17	11	121	35	1.40	0.14	0.09	0.29	0.10	0.06	0.71	0.20
984	Tr.2 UN-A2-3 H2 S2 30.06.23 one each	Kelbadjar	2	2	2	A2-A3	6/30/23	46	7/2/23	559	5477	41	167	18	11	119	39	1.41	0.15	0.10	0.33	0.10	0.07	0.71	0.23
985	Tr.2 UN-A2-3 H2 S2 30.06.23 one each	Kelbadjar	2	2	2	A2-A3	6/30/23	47	7/2/23	549	5570	43	172	20	10	117	37	1.47	0.17	0.08	0.32	0.11	0.06	0.68	0.22
986	Tr.2 UN-A2-3 H2 S2 30.06.23 one each	Kelbadjar	2	2	2	A2-A3	6/30/23	48	7/2/23	580	5460	43	165	16	10	109	36	1.51	0.14	0.09	0.33	0.09	0.06	0.66	0.22
987	Tr.2 UN-A2-3 H2 S2 30.06.23 one each	Gegham 1	2	2	2	A2-A3	6/30/23	49	7/2/23	705	4008	33	218	13	23	73	55	2.97	0.17	0.31	0.75	0.06	0.10	0.34	0.25
988	Tr.2 UN-A2-3 H2 S2 30.06.23 one each	Gegham 1	2	2	2	A2-A3	6/30/23	50	7/2/23	908	4915	41	243	14	24	76	57	3.19	0.18	0.31	0.74	0.06	0.10	0.31	0.23
989	Tr.2 UN-A2-3 H2 S2 30.06.23 one each	Gegham 1	2	2	2	A2-A3	6/30/23	51	7/2/23	786	4237	33	229	15	24	72	54	3.19	0.20	0.33	0.75	0.06	0.10	0.31	0.24
990	Tr.2 UN-A2-3 H2 S2 30.06.23 one each	Gegham 1	2	2	2	A2-A3	6/30/23	52	7/2/23	757	4121	36	218	14	26	73	55	2.97	0.18	0.35	0.75	0.06	0.12	0.34	0.25
991	Tr.2 UN-A2-3 H2 S2 30.06.23 one each	Kelbadjar	2	2	2	A2-A3	6/30/23	53	7/2/23	549	5600	41	168	17	12	113	37	1.49	0.15	0.11	0.33	0.10	0.07	0.67	0.22
992	Tr.2 UN-A2-3 H2 S2 30.06.23 one each	Kelbadjar	2	2	2	A2-A3	6/30/23	54	7/2/23	498	5382	38	165	17	11	112	36	1.47	0.15	0.10	0.32	0.10	0.07	0.68	0.22
993	Tr.2 UN-A2-3 H2 S2 30.06.23 one each	Syunik	2	2	2	A2-A3	6/30/23	55	7/2/23	612	6340	45	207	22	9	106	33	1.96	0.20	0.08	0.32	0.10	0.04	0.51	0.16
994	Tr.2 UN-A2-3 H2 S2 30.06.23 one each	Gegham 1	2	2	2	A2-A3	6/30/23	56	7/2/23	808	4508	40	244	13	24	76	57	3.20	0.16	0.31	0.74	0.05	0.10	0.31	0.23
995	Tr.2 UN-A2-3 H2 S2 30.06.23 one each	Gegham 1	2	2	2	A2-A3	6/30/23	57	7/2/23	733	4021	35	220	14	25	73	54	2.99	0.18	0.34	0.73	0.06	0.11	0.33	0.25
996	Tr.2 UN-A2-3 H2 S2 30.06.23 one each	Gegham 1	2	2	2	A2-A3	6/30/23	58	7/2/23	741	4051	35	223	13	24	70	54	3.20	0.18	0.34	0.77	0.06	0.11	0.31	0.24
997	Tr.2 UN-A2-3 H2 S2 30.06.23 one each	Kelbadjar	2	2	2	A2-A3	6/30/23	59	7/2/23	511	5781	45	172	18	11	124	36	1.39	0.14	0.09	0.29	0.10	0.07	0.72	0.21
998	Tr.2 UN-A2-3 H2 S2 30.06.23 one each	Gegham 1	2	2	2	A2-A3	6/30/23	60	7/2/23	778	4325	40	233	14	25	77	55	3.01	0.17	0.32	0.71	0.06	0.11	0.33	0.24
999	Tr.2 UN-A2-3 H2 S2 30.06.23 one each	Kelbadjar	2	2	2	A2-A3	6/30/23	61	7/2/23	581	5933	46	175	19	11	115	37	1.52	0.16	0.09	0.33	0.11	0.06	0.66	0.21
1000	Tr.2 UN-A2-3 H2 S2 30.06.23 one each	Kelbadjar	2	2	2	A2-A3	6/30/23	62	7/2/23	607	6050	47	180	17	9	116	33	1.55	0.14	0.07	0.29	0.09	0.05	0.64	0.18
1001	Tr.2 UN-A2-3 H2 S2 30.06.23 one each	Gegham 1	2	2	2	A2-A3	6/30/23	63	7/2/23	889	4619	43	240	13	27	79	55	3.04	0.16	0.34	0.70	0.05	0.11	0.33	0.23
1002	Tr.2 UN-A2-3 H2 S2 30.06.23 one each	Kelbadjar	2	2	2	A2-A3	6/30/23	64	7/2/23	529	5109	45	165	15	13	111	37	1.49	0.13	0.12	0.34	0.09	0.08	0.67	0.23
1003	Tr.2 UN-A2-3 H2 S2 30.06.23 one each	Kelbadjar	2	2	2	A2-A3	6/30/23	65	7/2/23	545	5669	46	171	17	10	116	39	1.48	0.14	0.08	0.33	0.10	0.06	0.68	0.23
1004	Tr.2 UN-A2-3 H2 S2 30.06.23 one each	Gegham 1	2	2	2	A2-A3	6/30/23	66	7/2/23	726	4085	34	228	14	27	73	57	3.14	0.19	0.37	0.78	0.06	0.12	0.32	0.25
1005	Tr.2 UN-A2-3 H2 S2 30.06.23 one each	Kelbadjar	2	2	2	A2-A3	6/30/23	67	7/2/23	561	5651	43	171	18	12	115	39	1.49	0.15	0.11	0.34	0.10	0.07	0.67	0.23
1006	Tr.2 UN-A2-3 H2 S2 30.06.23 one each	Kelbadjar	2	2	2	A2-A3	6/30/23	68	7/2/23	586	6020	49	179	18	10	121	37	1.48	0.15	0.08	0.31	0.10	0.05	0.67	0.21
1007	Tr.2 UN-A2-3 H2 S2 30.06.23 one each	Kelbadjar	2	2	2	A2-A3	6/30/23	69	7/2/23	601	6244	49	182	17	11	117	39	1.56	0.14	0.09	0.33	0.09	0.06	0.64	0.21
1008	Tr.2 UN-A2-3 H2 S2 30.06.23 one each	Gegham 1	2	2	2	A2-A3	6/30/23	70	7/2/23	818	4526	38	232	15	27	74	54	3.11	0.19	0.36	0.72	0.06	0.11	0.32	0.23
1009	Tr.2 UN-A2-3 H2 S2 30.06.23 one each	Gegham 1	2	2	2	A2-A3	6/30/23	71	7/2/23	839	4786	44	250	13	25	76	53	3.28	0.16	0.32	0.69	0.05	0.10	0.31	0.21
1010	Tr.2 UN.C1 H1.S1 30.06.23 1 of 6	Kelbadjar	2	1	1	C1	6/30/23	73	7/2/23	510	5601	38	163	16	11	113	36	1.44	0.14	0.10	0.32	0.10	0.07	0.70	0.22
1011	Tr.2 UN.C1 H1.S1 30.06.23 3 of 6	Gegham 1	2	1	1	C1	6/30/23	77	7/2/23	784	4303	36	227	16	25	74	58	3.05	0.21	0.33	0.78	0.07	0.11	0.33	0.26
1012	Tr.2 UN.C1 H1.S1 30.06.23 4 of 6	Gegham 1	2	1	1	C1	6/30/23	79	7/2/23	729	4013	36	221	14	25	73	57	3.00	0.18	0.34	0.77	0.06	0.11	0.33	0.26
1013	Tr.2 UN.C1 H1.S1 30.06.23 5 of 6	Gegham 1	2	1	1	C1	6/30/23	81	7/2/23	683	3916	35	206	14	23	74	51	2.77	0.18	0.31	0.69	0.07	0.11	0.36	0.25
1014	Tr.2 UN.C1 H1.S1 30.06.23 6 of 6	Gegham 1	2	1	1	C1	6/30/23	83	7/2/23	692	4118	40	224	23	26	75	59	2.97	0.30	0.34	0.79	0.10	0.11	0.34	0.27
1015	Trench-2 H3 Sp2 A2-A3 1 of 9	Gegham 1	2	3	2	A2-A3	-	85	7/2/23	702	3824	34	208	11	25	73	55	2.83	0.16	0.34	0.75	0.06	0.12	0.35	0.27
1016	Trench-2 H3 Sp2 A2-A3 2 of 9	Gegham 1	2	3	2	A2-A3	-	87	7/2/23	712	3848	33	209	11	26	73	53	2.88	0.16	0.35	0.72	0.05	0.12	0.35	0.25

1017	Trench-2 H3 Sp2 A2-A3 3 of 9	Gegham 1	2	3	2	A2-A3	-	89	7/2/23	783	4202	38	224	14	25	72	54	3.13	0.19	0.34	0.75	0.06	0.11	0.32	0.24
1018	Trench-2 H3 Sp2 A2-A3 4 of 9	Gegham 1	2	3	2	A2-A3	-	91	7/2/23	787	4493	36	234	15	23	77	58	3.03	0.19	0.29	0.75	0.06	0.10	0.33	0.25
1019	Trench-2 H3 Sp2 A2-A3 5 of 9	Gegham 1	2	3	2	A2-A3	-	93	7/2/23	718	4125	35	220	15	25	73	54	2.99	0.20	0.34	0.73	0.07	0.11	0.33	0.25
1020	Trench-2 H3 Sp2 A2-A3 6 of 9	Kelbadjar	2	3	2	A2-A3	-	95	7/2/23	518	5389	41	165	17	13	109	36	1.51	0.15	0.12	0.33	0.10	0.08	0.66	0.22
1021	Trench-2 H3 Sp2 A2-A3 7 of 9	Gegham 1	2	3	2	A2-A3	-	97	7/2/23	731	4233	40	230	16	26	78	58	2.94	0.20	0.33	0.74	0.07	0.11	0.34	0.25
1022	Trench-2 H3 Sp2 A2-A3 9 of 9	Syunik	2	3	2	A2-A3	-	102	7/2/23	729	7280	57	195	18	10	120	35	1.62	0.15	0.08	0.29	0.09	0.05	0.62	0.18
1023	Tr.2 UN A2-A4 H.0 ??.06.23 2 of 3	Kelbadjar	2	0	-	A2-A4	-	105	7/2/23	565	5737	43	175	16	11	115	35	1.52	0.13	0.09	0.30	0.09	0.06	0.66	0.20
1024	Tr.2 UN A2-A4 H.0 ??.06.23 3 of 3	Gegham 1	2	0	-	A2-A4	-	107	7/2/23	733	4157	33	220	16	24	73	58	2.99	0.21	0.32	0.79	0.07	0.11	0.33	0.26
1025	Tr-2 Hor-0 Unit-A0 1 of 10	Syunik	2	0	-	A0	-	111	7/2/23	437	4947	36	173	23	11	103	36	1.69	0.22	0.11	0.35	0.13	0.07	0.59	0.21
1026	Tr-2 Hor-0 Unit-A0 2 of 10	Gegham 1	2	0	-	A0	-	113	7/2/23	682	3783	35	212	13	22	73	57	2.92	0.17	0.30	0.78	0.06	0.10	0.34	0.27
1027	Tr-2 Hor-0 Unit-A0 3 of 10	Kelbadjar	2	0	-	A0	-	115	7/2/23	486	5153	40	163	19	10	112	35	1.45	0.16	0.09	0.31	0.11	0.06	0.69	0.21
1028	Tr-2 Hor-0 Unit-A0 4 of 10	Arteni	2	0	-	A0	-	117	7/2/23	614	4668	39	133	32	27	90	31	1.49	0.35	0.30	0.34	0.24	0.20	0.67	0.23
1029	Tr-2 Hor-0 Unit-A0 5 of 10	Gegham 1	2	0	-	A0	-	119	7/2/23	793	4384	37	229	16	25	71	55	3.24	0.22	0.35	0.78	0.07	0.11	0.31	0.24
1030	Tr-2 Hor-0 Unit-A0 7 of 10	Gegham 1	2	0	-	A0	-	122	7/2/23	650	3928	35	211	22	26	77	57	2.73	0.28	0.33	0.73	0.10	0.12	0.37	0.27
1031	Tr-2 Hor-0 Unit-A0 8 of 10	Gegham 1	2	0	-	A0	-	124	7/2/23	792	4305	38	231	15	26	76	55	3.02	0.19	0.34	0.72	0.06	0.11	0.33	0.24
1032	Tr-2 Hor-0 Unit-A0 10 of 10	Gegham 1	2	0	-	A0	-	127	7/2/23	833	4631	40	247	15	27	82	57	3.01	0.18	0.33	0.69	0.06	0.11	0.33	0.23
1033	Tr.2 UN-A4 H2.S1 29.06.23 1 of 3	Gegham 1	2	2	1	A4	6/29/23	130	7/2/23	626	3745	31	205	11	25	73	55	2.83	0.16	0.34	0.76	0.06	0.12	0.35	0.27
1034	Tr.2 UN-A4 H2.S1 29.06.23 2 of 3	Gegham 1	2	2	1	A4	6/29/23	132	7/2/23	646	3908	40	207	17	24	78	54	2.65	0.21	0.30	0.69	0.08	0.11	0.38	0.26
1035	Tr.2 UN-A4 H2.S1 29.06.23 3 of 3	Gegham 1	2	2	1	A4	6/29/23	133	7/2/23	681	3869	35	223	14	24	75	58	2.96	0.18	0.31	0.77	0.06	0.11	0.34	0.26
1036	Tr.2 UN-A4 H2.S2 29.06.23 1 of 9	Kelbadjar	2	2	1	A4	6/29/23	135	7/2/23	576	5388	41	164	18	9	108	36	1.51	0.16	0.08	0.33	0.11	0.05	0.66	0.22
1037	Tr.2 UN-A4 H2.S2 29.06.23 2 of 9	Gegham 1	2	2	1	A4	6/29/23	137	7/2/23	648	3755	35	213	14	26	73	55	2.94	0.19	0.35	0.76	0.06	0.12	0.34	0.26
1038	Tr.2 UN-A4 H2.S2 29.06.23 3 of 9	Gegham 1	2	2	1	A4	6/29/23	139	7/2/23	769	4241	35	223	13	26	75	57	2.96	0.17	0.34	0.75	0.06	0.11	0.34	0.25
1039	Tr.2 UN-A4 H2.S2 29.06.23 4 of 9	Kelbadjar	2	2	1	A4	6/29/23	141	7/2/23	545	5717	46	172	17	11	110	37	1.56	0.15	0.10	0.34	0.10	0.06	0.64	0.22
1040	Tr.2 UN-A4 H2.S2 29.06.23 5 of 9	Kelbadjar	2	2	1	A4	6/29/23	2	7/3/23	508	5396	44	165	19	12	113	42	1.46	0.16	0.11	0.37	0.11	0.07	0.68	0.25
1041	Tr.2 UN-A4 H2.S2 29.06.23 6 of 9	Gegham 1	2	2	1	A4	6/29/23	5	7/3/23	849	4567	40	238	13	24	75	54	3.16	0.17	0.31	0.71	0.05	0.10	0.32	0.23
1042	Tr.2 UN-A4 H2.S2 29.06.23 7 of 9	Gegham 1	2	2	1	A4	6/29/23	7	7/3/23	819	4556	42	233	15	25	77	58	3.02	0.19	0.32	0.75	0.06	0.11	0.33	0.25
1043	Tr.2 UN-A4 H2.S2 29.06.23 8 of 9	Kelbadjar	2	2	1	A4	6/29/23	8	7/3/23	542	5285	39	164	18	12	112	40	1.46	0.16	0.11	0.36	0.11	0.08	0.69	0.25
1044	Tr.2 UN-A4 H2.S2 29.06.23 9 of 9	Kelbadjar	2	2	1	A4	6/29/23	10	7/3/23	585	5869	47	174	19	10	120	37	1.45	0.15	0.08	0.31	0.11	0.06	0.69	0.21
1045	TR.2 UN.C1 H.0 29.06.23 1 of 9	Gegham 1	2	0	-	C1	6/29/23	12	7/3/23	688	3824	34	214	16	26	70	55	3.07	0.22	0.37	0.79	0.07	0.12	0.33	0.26
1046	TR.2 UN.C1 H.0 29.06.23 2 of 9	Gegham 1	2	0	-	C1	6/29/23	14	7/3/23	710	4021	34	219	14	27	73	54	2.98	0.18	0.36	0.73	0.06	0.12	0.34	0.25
1047	TR.2 UN.C1 H.0 29.06.23 3 of 9	Gutansar	2	0	-	C1	6/29/23	16	7/3/23	653	8776	44	153	132	26	184	40	0.83	0.72	0.14	0.22	0.86	0.17	1.20	0.26
1048	TR.2 UN.C1 H.0 29.06.23 4 of 9	Syunik	2	0	-	C1	6/29/23	19	7/3/23	511	5229	37	185	22	12	105	37	1.77	0.21	0.12	0.36	0.12	0.07	0.57	0.20
1049	TR.2 UN.C1 H.0 29.06.23 5 of 9	Hatis	2	0	-	C1	6/29/23	20	7/3/23	537	6325	34	116	113	17	99	24	1.17	1.14	0.17	0.24	0.97	0.15	0.85	0.20
1050	TR.2 UN.C1 H.0 29.06.23 6 of 9	Gegham 1	2	0	-	C1	6/29/23	22	7/3/23	761	4147	35	229	14	26	76	57	3.00	0.18	0.34	0.74	0.06	0.11	0.33	0.25
1051	TR.2 UN.C1 H.0 29.06.23 7 of 9	Syunik	2	0	-	C1	6/29/23	24	7/3/23	501	5633	38	180	24	11	107	33	1.69	0.22	0.11	0.31	0.13	0.06	0.59	0.18
1052	TR.2 UN.C1 H.0 29.06.23 8 of 9	Gutansar	2	0	-	C1	6/29/23	26	7/3/23	680	8774	42	152	133	25	180	37	0.84	0.74	0.14	0.21	0.87	0.16	1.19	0.25
1053	TR.2 UN.C1 H.0 29.06.23 9 of 9	Kelbadjar	2	0	-	C1	6/29/23	28	7/3/23	616	6101	48	184	18	11	115	39	1.60	0.15	0.10	0.34	0.10	0.06	0.63	0.21
1054	TR.2 UN-21 TS 29.06.23 1 of 5	Gegham 1	2	0	-	?	6/29/23	30	7/3/23	680	3779	33	209	13	25	72	55	2.92	0.17	0.34	0.77	0.06	0.12	0.34	0.26
1055	TR.2 UN-21 TS 29.06.23 2 of 5	Syunik	2	0	-	?	6/29/23	32	7/3/23	511	5073	34	179	18	11	101	39	1.77	0.17	0.11	0.38	0.10	0.06	0.56	0.22
1056	TR.2 UN-21 TS 29.06.23 3 of 5	Gegham 1	2	0	-	?	6/29/23	34	7/3/23	776	4398	38	231	13	25	73	58	3.14	0.17	0.34	0.79	0.05	0.11	0.32	0.25
1057	TR.2 UN-21 TS 29.06.23 4 of 5	Kelbadjar	2	0	-	?	6/29/23	36	7/3/23	503	5104	38	161	16	10	106	33	1.52	0.15	0.09	0.32	0.10	0.06	0.66	0.21
1058	TR.2 UN-21 TS 29.06.23 5 of 5	Gegham 1	2	0	-	?	6/29/23	38	7/3/23	889	4772	42	246	15	27	79	54	3.11	0.18	0.34	0.68	0.06	0.11	0.32	0.22
1059	Tr-2 Hor4 Sp2 U-A5 1.7.23 :) 1 of 15	Syunik	2	4	2	A5	7/1/23	41	7/3/23	498	5062	35	188	16	11	99	36	1.90	0.16	0.12	0.36	0.08	0.06	0.53	0.19
1060	Tr-2 Hor4 Sp2 U-A5 1.7.23 :) 2 of 15	Gegham 1	2	4	2	A5	7/1/23	43	7/3/23	710	3932	35	213	13	27	74	57	2.86	0.17	0.36	0.76	0.06	0.12	0.35	0.27
1061	Tr-2 Hor4 Sp2 U-A5 1.7.23 :) 3 of 15	Gegham 1	2	4	2	A5	7/1/23	45	7/3/23	656	3748	34	205	11	26	73	57	2.83	0.16	0.35	0.78	0.06	0.12	0.35	0.28
1062	Tr-2 Hor4 Sp2 U-A5 1.7.23 :) 4 of 15	Gegham 1	2	4	2	A5	7/1/23	48	7/3/23	658	3543	34	205	13	25	70	53	2.95	0.18	0.35	0.75	0.06	0.12	0.34	0.26
1063	Tr-2 Hor4 Sp2 U-A5 1.7.23 :) 5 of 15	Kelbadjar	2	4	2	A5	7/1/23	49	7/3/23	461	4941	35	168	22	12	103	32	1.64	0.21	0.12	0.31	0.13	0.07	0.61	0.19
1064	Tr-2 Hor4 Sp2 U-A5 1.7.23 :) 6 of 15	Kelbadjar	2	4	2	A5	7/1/23	51	7/3/23	535	5596	44	166	17	10	111	35	1.50	0.15	0.09	0.31	0.10	0.06	0.67	0.21
1065	Tr-2 Hor4 Sp2 U-A5 1.7.23 :) 7 of 15	Gegham 1	2	4	2	A5	7/1/23	53	7/3/23	788	4438	37	230	15	28	78	57	2.94	0.19	0.36	0.72	0.06	0.12	0.34	0.25
1066	Tr-2 Hor4 Sp2 U-A5 1.7.23 :) 8 of 15	Gegham 1	2	4	2	A5	7/1/23	55	7/3/23	712	4064	32	219	13	26	73	55	3.02	0.17	0.35	0.76	0.06	0.12	0.33	0.25
1067	Tr-2 Hor4 Sp2 U-A5 1.7.23 :) 9 of 15	Gegham 1	2	4	2	A5	7/1/23	57	7/3/23	985	5305	49	258	14	25	77	54	3.34	0.17	0.32	0.70	0.05	0.10	0.30	0.21

1068	Tr-2 Hor4 Sp2 U-A5 1.7.23 :) 10 of 15	Gegham 1	2	4	2	A5	7/1/23	59	7/3/23	786	4450	37	229	14	26	78	58	2.92	0.17	0.33	0.74	0.06	0.11	0.34	0.25
1069	Tr-2 Hor4 Sp2 U-A5 1.7.23 :) 11 of 15	Gegham 1	2	4	2	A5	7/1/23	61	7/3/23	746	4077	36	224	13	26	73	58	3.08	0.17	0.35	0.80	0.06	0.11	0.32	0.26
1070	Tr-2 Hor4 Sp2 U-A5 1.7.23 :) 12 of 15	Gegham 1	2	4	2	A5	7/1/23	63	7/3/23	736	4251	37	228	13	25	74	57	3.06	0.17	0.33	0.76	0.05	0.11	0.33	0.25
1071	Tr-2 Hor4 Sp2 U-A5 1.7.23 :) 13 of 15	Syunik	2	4	2	A5	7/1/23	65	7/3/23	658	6832	47	211	28	10	113	37	1.87	0.24	0.08	0.33	0.13	0.05	0.54	0.18
1072	Tr-2 Hor4 Sp2 U-A5 1.7.23 :) 14 of 15	Syunik	2	4	2	A5	7/1/23	67	7/3/23	586	5992	39	199	27	11	110	35	1.81	0.24	0.10	0.31	0.13	0.05	0.55	0.17
1073	Tr-2 Hor4 Sp2 U-A5 1.7.23 :) 15 of 15	Syunik	2	4	2	A5	7/1/23	70	7/3/23	574	5662	40	195	21	10	103	36	1.89	0.20	0.09	0.35	0.11	0.05	0.53	0.19
1074	TR.2 UN-A1 H1.S1 29.06.23 1 of 5	Kelbadjar	2	1	1	A1	6/29/23	71	7/3/23	557	5987	42	170	22	8	112	37	1.52	0.19	0.07	0.33	0.13	0.05	0.66	0.22
1075	TR.2 UN-A1 H1.S1 29.06.23 2 of 5	Kelbadjar	2	1	1	A1	6/29/23	73	7/3/23	594	5724	45	176	18	11	111	36	1.58	0.16	0.09	0.32	0.10	0.06	0.63	0.20
1076	TR.2 UN-A1 H1.S1 29.06.23 3 of 5	Kelbadjar	2	1	1	A1	6/29/23	75	7/3/23	556	5768	40	168	16	11	111	35	1.51	0.14	0.09	0.31	0.09	0.06	0.66	0.21
1077	TR.2 UN-A1 H1.S1 29.06.23 4 of 5	Gegham 1	2	1	1	A1	6/29/23	77	7/3/23	813	4438	35	232	14	27	75	55	3.07	0.18	0.35	0.73	0.06	0.11	0.33	0.24
1078	TR.2 UN-A1 H1.S1 29.06.23 5 of 5	Gegham 1	2	1	1	A1	6/29/23	79	7/3/23	838	4960	38	233	16	27	75	59	3.10	0.21	0.35	0.79	0.07	0.11	0.32	0.25
1079	TR.2 UN-C! TS H.0 29.06.23 1 of 1	Syunik	2	0	-	C1	6/29/23	81	7/3/23	589	6035	45	200	21	11	110	39	1.82	0.19	0.10	0.35	0.10	0.05	0.55	0.19
1080	TR.2 UN-A5 TS 29-06-23 1 of 1	Kelbadjar	2	0	-	A5	6/29/23	83	7/3/23	519	5388	42	162	19	13	110	39	1.46	0.17	0.12	0.35	0.11	0.08	0.68	0.24
1081	TR.2 UN.A2-A4 H0 collapse 1 of 2	Gegham 1	2	0	collapse	A2-A4	-	85	7/3/23	678	3615	34	197	11	26	71	53	2.78	0.16	0.36	0.74	0.06	0.13	0.36	0.27
1082	TR.2 UN.A2-A4 H0 collapse 2 of 2	Kelbadjar	2	0	collapse	A2-A4	-	87	7/3/23	483	5112	43	161	17	11	106	33	1.52	0.16	0.10	0.32	0.10	0.07	0.66	0.21
1083	TR2 UN.A4 H1 S2 29.06.23 2 of 5	Kelbadjar	2	1	2	A4	6/29/23	91	7/3/23	494	5077	37	159	16	11	109	33	1.45	0.14	0.10	0.30	0.10	0.07	0.69	0.21
1084	TR2 UN.A4 H1 S2 29.06.23 4 of 5	Gegham 1	2	1	2	A4	6/29/23	95	7/3/23	796	4185	38	229	14	27	73	54	3.11	0.18	0.36	0.73	0.06	0.12	0.32	0.24
1085	TR2 UN.A4 H1 S2 29.06.23 5 of 5	Gegham 1	2	1	2	A4	6/29/23	97	7/3/23	774	4228	46	226	15	26	73	54	3.11	0.20	0.35	0.74	0.06	0.11	0.32	0.24
1086	TR.2 UN-A2-A3 H.0 S1 29.06.23 2 of 7	Kelbadjar	2	0	1	A2-A3	6/29/23	101	7/3/23	652	6588	50	188	18	10	125	37	1.50	0.14	0.08	0.30	0.09	0.05	0.67	0.20
1087	TR.2 UN-A2-A3 H.0 S1 29.06.23 3 of 7	Gegham 1	2	0	1	A2-A3	6/29/23	103	7/3/23	744	4247	36	222	15	28	76	57	2.91	0.19	0.37	0.74	0.07	0.13	0.34	0.26
1088	TR.2 UN-A2-A3 H.0 S1 29.06.23 4 of 7	Kelbadjar	2	0	1	A2-A3	6/29/23	105	7/3/23	496	5060	45	159	20	10	114	35	1.39	0.17	0.08	0.30	0.12	0.06	0.72	0.22
1089	TR.2 UN-A2-A3 H.0 S1 29.06.23 5 of 7	Gegham 1	2	0	1	A2-A3	6/29/23	107	7/3/23	802	4106	35	226	13	26	74	54	3.03	0.17	0.34	0.72	0.06	0.11	0.33	0.24
1090	TR.2 UN-A1-A3 H.0 S1 29.06.23 6 of 7	Gegham 1	2	0	1	A2-A3	6/29/23	109	7/3/23	804	4429	39	233	14	23	77	57	3.01	0.17	0.29	0.73	0.06	0.10	0.33	0.24
1091	TR.2 UN-A1-A3 H.0 S1 29.06.23 7 of 7	Gegham 1	2	0	1	A2-A3	6/29/23	111	7/3/23	712	3956	33	221	14	28	74	57	2.97	0.18	0.38	0.76	0.06	0.13	0.34	0.26
1092	Tr-2 Hor4 Sp2 A2-A3 1 of 5	Gegham 1	2	4	2	A2-A3	-	113	7/3/23	631	3696	31	210	11	25	74	58	2.82	0.15	0.33	0.78	0.05	0.12	0.35	0.28
1093	Tr-2 Hor4 Sp2 A2-A3 2 of 5	Gegham 1	2	4	2	A2-A3	-	115	7/3/23	658	3814	32	207	14	27	73	53	2.82	0.18	0.37	0.71	0.07	0.13	0.35	0.25
1094	Tr-2 Hor4 Sp2 A2-A3 3 of 5	Gegham 1	2	4	2	A2-A3	-	117	7/3/23	835	4612	42	237	17	26	75	58	3.15	0.22	0.34	0.77	0.07	0.11	0.32	0.24
1095	Tr-2 Hor4 Sp2 A2-A3 4 of 5	Gegham 1	2	4	2	A2-A3	-	119	7/3/23	842	4712	41	241	16	26	77	54	3.12	0.20	0.33	0.70	0.06	0.11	0.32	0.22
1096	Tr-2 Hor4 Sp2 A2-A3 5 of 5	Gegham 1	2	4	2	A2-A3	-	121	7/3/23	893	4902	46	249	14	26	79	58	3.15	0.17	0.32	0.73	0.05	0.10	0.32	0.23
1097	Tr-2 Hor3 Sp1 A4 one each	Gegham 1	2	3	1	A4	-	123	7/3/23	653	3676	29	205	13	23	69	53	2.99	0.18	0.33	0.76	0.06	0.11	0.33	0.26
1098	Tr-2 Hor3 Sp1 A4 one each	Gegham 1	2	3	1	A4	-	124	7/3/23	673	3841	34	209	13	26	71	53	2.96	0.18	0.36	0.74	0.06	0.12	0.34	0.25
1099	Tr-2 Hor3 Sp1 A4 one each	Gegham 1	2	3	1	A4	-	125	7/3/23	673	3785	31	204	10	24	71	54	2.89	0.15	0.34	0.76	0.05	0.12	0.35	0.26
1100	Tr-2 Hor3 Sp1 A4 one each	Kelbadjar	2	3	1	A4	-	126	7/3/23	478	4887	38	154	15	13	125	37	1.23	0.12	0.11	0.30	0.09	0.09	0.82	0.24
1101	Tr-2 Hor3 Sp1 A4 one each	Gegham 1	2	3	1	A4	-	127	7/3/23	660	3747	29	205	14	26	71	53	2.91	0.19	0.36	0.74	0.07	0.12	0.34	0.26
1102	Tr-2 Hor3 Sp1 A4 one each	Gegham 1	2	3	1	A4	-	128	7/3/23	731	4100	35	218	14	27	74	54	2.93	0.18	0.36	0.72	0.06	0.12	0.34	0.25
1103	Tr-2 Hor3 Sp1 A4 one each	Kelbadjar	2	3	1	A4	-	129	7/3/23	510	5219	43	166	16	10	114	39	1.46	0.14	0.08	0.34	0.09	0.06	0.69	0.23
1104	Tr-2 Hor3 Sp1 A4 one each	Kelbadjar	2	3	1	A4	-	130	7/3/23	564	5942	41	177	18	9	112	36	1.58	0.16	0.08	0.32	0.10	0.05	0.63	0.20
1105	Tr-2 Hor3 Sp1 A4 one each	Gegham 1	2	3	1	A4	-	131	7/3/23	741	4208	36	232	16	26	73	53	3.19	0.21	0.35	0.72	0.07	0.11	0.31	0.23
1106	Tr-2 Hor3 Sp1 A4 one each	Gegham 1	2	3	1	A4	-	132	7/3/23	772	4202	36	222	11	25	74	55	2.98	0.15	0.33	0.74	0.05	0.11	0.34	0.25
1107	Tr-2 Hor3 Sp1 A4 one each	Gegham 1	2	3	1	A4	-	133	7/3/23	736	4258	38	224	14	26	75	57	2.97	0.18	0.34	0.75	0.06	0.11	0.34	0.25
1108	Tr-2 Hor3 Sp1 A4 one each	Syunik	2	3	1	A4	-	134	7/3/23	455	5213	36	181	22	10	105	39	1.73	0.21	0.09	0.37	0.12	0.05	0.58	0.21
1109	Tr-2 Hor3 Sp1 A4 one each	Gegham 1	2	3	1	A4	-	135	7/3/23	724	4093	34	222	14	25	73	58	3.06	0.19	0.34	0.80	0.06	0.11	0.33	0.26
1110	Tr-2 Hor3 Sp1 A4 one each	Kelbadjar	2	3	1	A4	-	136	7/3/23	560	5477	46	170	22	10	118	35	1.44	0.18	0.08	0.29	0.13	0.06	0.69	0.20
1111	Tr-2 Hor3 Sp1 A4 one each	Gegham 1	2	3	1	A4	-	137	7/3/23	715	4353	38	219	14	27	74	55	2.94	0.18	0.36	0.74	0.06	0.12	0.34	0.25
1112	Tr-2 Hor3 Sp1 A4 one each	Gegham 1	2	3	1	A4	-	138	7/3/23	668	3966	34	214	14	25	75	55	2.84	0.18	0.33	0.73	0.06	0.12	0.35	0.26
1113	Tr-2 Hor3 Sp1 A4 one each	Syunik	2	3	1	A4	-	139	7/3/23	504	5177	38	181	23	11	105	36	1.73	0.22	0.10	0.34	0.12	0.06	0.58	0.20
1114	Tr-2 Hor3 Sp1 A4 one each	Gegham 1	2	3	1	A4	-	140	7/3/23	706	4160	37	222	14	26	72	54	3.10	0.19	0.36	0.75	0.06	0.12	0.32	0.24
1115	Tr-2 Hor3 Sp1 A4 one each	Gegham 1	2	3	1	A4	-	141	7/3/23	726	4073	35	224	11	25	73	55	3.04	0.16	0.34	0.75	0.05	0.11	0.33	0.25
1116	Tr-2 Hor3 Sp1 A4 one each	Gegham 1	2	3	1	A4	-	142	7/3/23	726	4106	35	222	14	27	74	55	2.98	0.18	0.36	0.74	0.06	0.12	0.34	0.25
1117	Tr-2 Hor3 Sp1 A4 one each	Gegham 1	2	3	1	A4	-	143	7/3/23	688	3947	33	217	13	26	73	57	2.99	0.17	0.35	0.78	0.06	0.12	0.33	0.26
1118	Tr-2 Hor3 Sp1 A4 one each	Kelbadjar	2	3	1	A4	-	144	7/3/23	524	5385	41	165	21	13	118	36	1.40	0.17	0.11	0.31	0.12	0.08	0.71	0.22

1119	Tr-2 Hor3 Sp1 A4 one each	Gegham 1	2	3	1	A4	-	145	7/3/23	697	4033	35	217	15	25	75	54	2.88	0.19	0.33	0.71	0.07	0.11	0.35	0.25
1120	Tr-2 Hor3 Sp1 A4 one each	Arteni	2	3	1	A4	-	146	7/3/23	628	4113	41	141	22	29	86	35	1.65	0.25	0.34	0.40	0.15	0.21	0.61	0.25
1121	Tr-2 Hor3 Sp1 A4 one each	Gegham 1	2	3	1	A4	-	147	7/3/23	648	3665	34	209	15	25	69	53	3.04	0.21	0.36	0.76	0.07	0.12	0.33	0.25
1122	Tr-2 Hor3 Sp1 A4 one each	Gegham 1	2	3	1	A4	-	148	7/3/23	743	4091	35	220	14	25	76	55	2.88	0.18	0.32	0.72	0.06	0.11	0.35	0.25
1123	Tr-2 Hor3 Sp1 A4 one each	Gegham 1	2	3	1	A4	-	149	7/3/23	692	4085	40	222	21	25	76	58	2.91	0.27	0.32	0.76	0.09	0.11	0.34	0.26
1124	Tr-2 Hor3 Sp1 A4 one each	Gegham 1	2	3	1	A4	-	150	7/3/23	716	4116	35	209	15	26	75	55	2.77	0.19	0.34	0.73	0.07	0.12	0.36	0.26
1125	Tr-2 Hor3 Sp1 A4 one each	Kelbadjar	2	3	1	A4	-	151	7/3/23	531	5276	43	170	18	11	111	35	1.53	0.16	0.10	0.31	0.10	0.07	0.65	0.20
1126	Tr-2 Hor3 Sp1 A4 one each	Gegham 1	2	3	1	A4	-	152	7/3/23	751	4251	39	232	15	27	79	57	2.92	0.18	0.34	0.72	0.06	0.11	0.34	0.24
1127	Tr-2 Hor3 Sp1 A4 one each	Gutansar	2	3	1	A4	-	153	7/3/23	673	8387	43	146	127	25	177	39	0.82	0.71	0.14	0.22	0.87	0.17	1.22	0.27
1128	Tr-2 Hor3 Sp1 A4 one each	Gegham 1	2	3	1	A4	-	154	7/3/23	702	4006	36	215	14	27	75	57	2.85	0.18	0.35	0.75	0.06	0.12	0.35	0.26
1129	Tr-2 Hor3 Sp1 A4 one each	Gegham 1	2	3	1	A4	-	155	7/3/23	747	4087	39	223	13	25	72	55	3.11	0.17	0.34	0.77	0.06	0.11	0.32	0.25
1130	Tr-2 Hor3 Sp1 A4 one each	Gegham 1	2	3	1	A4	-	156	7/3/23	767	4348	36	229	15	27	75	58	3.03	0.19	0.35	0.77	0.06	0.12	0.33	0.25
1131	Tr-2 Hor3 Sp1 A4 one each	Gegham 1	2	3	1	A4	-	157	7/3/23	809	4541	40	237	13	27	79	62	3.00	0.16	0.35	0.78	0.05	0.12	0.33	0.26
1132	Tr-2 Hor3 Sp1 A4 one each	Kelbadjar	2	3	1	A4	-	158	7/3/23	561	5937	49	176	17	11	116	36	1.52	0.14	0.09	0.31	0.09	0.06	0.66	0.20
1133	Tr-2 Hor3 Sp1 A4 one each	Gegham 1	2	3	1	A4	-	159	7/3/23	698	4015	35	214	13	27	74	54	2.88	0.17	0.36	0.72	0.06	0.12	0.35	0.25
1134	Tr-2 Hor3 Sp1 A4 one each	Gegham 1	2	3	1	A4	-	160	7/3/23	840	4898	46	249	15	25	73	53	3.39	0.20	0.34	0.71	0.06	0.10	0.30	0.21
1135	Tr-2 Hor3 Sp1 A4 one each	Kelbadjar	2	3	1	A4	-	161	7/3/23	557	6070	47	177	21	11	115	35	1.54	0.18	0.09	0.30	0.12	0.06	0.65	0.20
1136	Tr-2 Hor3 Sp1 A4 one each	Gegham 1	2	3	1	A4	-	162	7/3/23	867	4544	42	250	15	24	77	58	3.24	0.19	0.31	0.75	0.06	0.09	0.31	0.23
1137	Tr-2 Hor3 Sp1 A4 one each	Gegham 1	2	3	1	A4	-	163	7/3/23	777	4296	38	230	15	27	74	57	3.08	0.19	0.36	0.76	0.06	0.12	0.32	0.25
1138	Tr-2 Hor3 Sp1 A4 one each	Syunik	2	3	1	A4	-	164	7/3/23	647	6549	49	188	18	11	116	36	1.62	0.15	0.09	0.31	0.09	0.06	0.62	0.19
1139	Tr-2 Hor3 Sp1 A4 one each	Syunik	2	3	1	A4	-	165	7/3/23	731	7681	58	234	23	9	108	36	2.18	0.21	0.08	0.34	0.10	0.04	0.46	0.15
1140	Tr-2 Hor3 Sp1 A4 one each	Gegham 1	2	3	1	A4	-	166	7/3/23	862	4610	45	238	14	25	74	55	3.20	0.18	0.33	0.74	0.06	0.10	0.31	0.23
1141	Tr-2 Hor3 Sp1 A4 one each	Gegham 1	2	3	1	A4	-	167	7/3/23	818	4512	39	239	15	27	79	58	3.02	0.18	0.34	0.73	0.06	0.11	0.33	0.24
1142	Tr-2 Hor3 Sp1 A4 one each	Gegham 1	2	3	1	A4	-	168	7/3/23	845	4691	41	242	17	27	78	57	3.10	0.21	0.35	0.72	0.07	0.11	0.32	0.23
1143	Tr-2 Hor3 Sp1 A4 one each	Gegham 1	2	3	1	A4	-	169	7/3/23	637	3844	35	210	13	26	73	55	2.90	0.17	0.35	0.76	0.06	0.12	0.35	0.26
1144	Tr-2 Hor3 Sp1 A4 one each	Gegham 1	2	3	1	A4	-	170	7/3/23	777	4099	38	226	14	24	75	59	2.99	0.18	0.31	0.79	0.06	0.11	0.33	0.26
1145	Tr-2 Hor3 Sp1 A4 one each	Gegham 1	2	3	1	A4	-	171	7/3/23	742	4071	34	225	15	27	77	57	2.91	0.19	0.34	0.73	0.06	0.12	0.34	0.25
1146	Tr2 A4 H2 S2 30.06.23 one each	Gegham 1	2	1	2	A2-A3	-	173	7/3/23	671	3846	34	207	14	24	72	57	2.89	0.19	0.33	0.79	0.07	0.11	0.35	0.27
1147	TR2 U A2-A3 H1 S2 ??.06.23 one each	Kelbadjar	2	1	2	A2-A3	-	174	7/3/23	508	5466	45	163	17	11	115	37	1.41	0.14	0.10	0.33	0.10	0.07	0.71	0.23
1148	TR2 U A2-A3 H1 S2 ??.06.23 one each	Gegham 1	2	1	2	A2-A3	-	175	7/3/23	697	4057	34	218	13	26	77	55	2.82	0.16	0.33	0.71	0.06	0.12	0.35	0.25
1149	TR2 U A2-A3 H1 S2 ??.06.23 one each	Gegham 1	2	1	2	A2-A3	-	176	7/3/23	737	4052	34	220	14	25	71	55	3.11	0.19	0.35	0.78	0.06	0.11	0.32	0.25
1150	TR2 U A2-A3 H1 S2 ??.06.23 one each	Gegham 1	2	1	2	A2-A3	-	177	7/3/23	723	3986	36	213	14	27	73	55	2.90	0.18	0.36	0.75	0.06	0.12	0.34	0.26
1151	TR2 U A2-A3 H1 S2 ??.06.23 one each	Gegham 1	2	1	2	A2-A3	-	178	7/3/23	767	4272	36	226	14	26	75	59	2.99	0.18	0.34	0.79	0.06	0.11	0.33	0.26
1152	TR2 U A2-A3 H1 S2 ??.06.23 one each	Gegham 1	2	1	2	A2-A3	-	179	7/3/23	687	4389	46	224	28	27	73	58	3.08	0.38	0.38	0.80	0.12	0.12	0.32	0.26
1153	TR2 U A2-A3 H1 S2 ??.06.23 one each	Gegham 1	2	1	2	A2-A3	-	180	7/3/23	763	4184	36	221	14	26	75	55	2.93	0.18	0.34	0.73	0.06	0.12	0.34	0.25
1154	TR2 U A2-A3 H1 S2 ??.06.23 one each	Gegham 1	2	1	2	A2-A3	-	181	7/3/23	652	3659	35	205	13	26	72	54	2.87	0.17	0.36	0.75	0.06	0.12	0.35	0.26
1155	TR2 U A2-A3 H1 S2 ??.06.23 one each	Gegham 1	2	1	2	A2-A3	-	182	7/3/23	696	3894	35	213	15	24	71	54	3.02	0.21	0.34	0.76	0.07	0.11	0.33	0.25
1156	TR2 U A2-A3 H1 S2 ??.06.23 one each	Kelbadjar	2	1	2	A2-A3	-	183	7/3/23	520	5227	43	165	17	11	110	32	1.50	0.15	0.10	0.29	0.10	0.06	0.67	0.19
1157	TR2 U A2-A3 H1 S2 ??.06.23 one each	Gegham 1	2	1	2	A2-A3	-	184	7/3/23	648	3714	31	201	14	26	73	54	2.78	0.19	0.35	0.74	0.07	0.13	0.36	0.27
1158	TR2 U A2-A3 H1 S2 ??.06.23 one each	Kelbadjar	2	1	2	A2-A3	-	186	7/3/23	566	5776	48	179	19	11	135	36	1.33	0.14	0.08	0.27	0.10	0.06	0.75	0.20
1159	TR2 U A2-A3 H1 S2 ??.06.23 one each	Kelbadjar	2	1	2	A2-A3	-	187	7/3/23	545	5524	46	167	23	11	110	39	1.52	0.20	0.10	0.35	0.13	0.06	0.66	0.23
1160	TR2 U A2-A3 H1 S2 ??.06.23 one each	Gegham 1	2	1	2	A2-A3	-	188	7/3/23	794	4518	42	233	16	27	77	57	3.01	0.20	0.36	0.73	0.07	0.12	0.33	0.24
1161	TR2 U A2-A3 H1 S2 ??.06.23 one each	Gegham 1	2	1	2	A2-A3	-	189	7/3/23	983	5317	51	262	16	25	76	58	3.43	0.20	0.32	0.76	0.06	0.09	0.29	0.22
1162	TR2 U A2-A3 H1 S2 ??.06.23 one each	Gegham 1	2	1	2	A2-A3	-	190	7/3/23	808	4508	41	237	15	27	81	57	2.93	0.18	0.33	0.70	0.06	0.11	0.34	0.24
1163	TR2 U A2-A3 H1 S2 ??.06.23 one each	Gegham 1	2	1	2	A2-A3	-	191	7/3/23	747	4120	34	222	15	27	71	53	3.14	0.21	0.38	0.74	0.07	0.12	0.32	0.24
1164	TR2 U A2-A3 H1 S2 ??.06.23 one each	Syunik	2	1	2	A2-A3	-	192	7/3/23	641	6143	46	204	22	11	107	40	1.92	0.20	0.11	0.38	0.11	0.06	0.52	0.20
1165	TR2 U A2-A3 H1 S2 ??.06.23 one each	Gegham 1	2	1	2	A2-A3	-	193	7/3/23	779	4069	38	226	15	29	74	55	3.03	0.19	0.39	0.74	0.06	0.13	0.33	0.24
1166	TR2 U A2-A3 H1 S2 ??.06.23 one each	Gegham 1	2	1	2	A2-A3	-	194	7/3/23	811	4314	46	232	16	27	78	58	2.96	0.20	0.34	0.74	0.07	0.11	0.34	0.25
1167	TR2 U A2-A3 H1 S2 ??.06.23 one each	Gegham 1	2	1	2	A2-A3	-	195	7/3/23	746	4177	40	227	14	24	74	55	3.05	0.18	0.32	0.74	0.06	0.10	0.33	0.24
1168	TR.2 UN B1 H0 29.06.23 one each	Kelbadjar	2	0	-	B1	6/29/23	196	7/3/23	581	6076	46	180	18	11	119	37	1.51	0.15	0.09	0.31	0.10	0.06	0.66	0.21
1169	TR.2 UN B1 H0 29.06.23 one each	Gegham 1	2	0	-	B1	6/29/23	197	7/3/23	706	3954	31	212	15	25	73	51	2.92	0.20	0.34	0.70	0.07	0.12	0.34	0.24

1170	TR.2 UN B1 H0 29.06.23 one each	Kelbadjar	2	0	-	B1	6/29/23	198	7/3/23	443	4902	40	158	22	12	114	37	1.38	0.19	0.11	0.33	0.14	0.08	0.72	0.24
1171	TR.2 UN B1 H0 29.06.23 one each	Kelbadjar	2	0	-	B1	6/29/23	199	7/3/23	495	5111	35	164	17	11	109	36	1.49	0.15	0.10	0.33	0.10	0.07	0.67	0.22
1172	TR.2 UN B1 H0 29.06.23 one each	Gegham 1	2	0	-	B1	6/29/23	201	7/3/23	752	4294	35	222	13	25	75	53	2.94	0.17	0.33	0.70	0.06	0.11	0.34	0.24
1173	TR.2 UN B1 H0 29.06.23 one each	Arteni	2	0	-	B1	6/29/23	203	7/3/23	655	5241	47	142	53	26	98	26	1.45	0.54	0.26	0.27	0.37	0.18	0.69	0.19
1174	TR.2 UN B1 H0 29.06.23 one each	Kelbadjar	2	0	-	B1	6/29/23	204	7/3/23	534	5593	46	169	19	11	111	36	1.52	0.17	0.10	0.32	0.11	0.07	0.66	0.21
1175	TR.2 UN B1 H0 29.06.23 one each	Gegham 1	2	0	-	B1	6/29/23	205	7/3/23	711	3967	33	210	11	27	73	57	2.86	0.16	0.36	0.77	0.05	0.13	0.35	0.27
1176	TR.2 UN B1 H0 29.06.23 one each	Kelbadjar	2	0	-	B1	6/29/23	206	7/3/23	536	5257	43	164	18	11	112	37	1.46	0.16	0.10	0.33	0.11	0.07	0.69	0.23
1177	TR.2 UN B1 H0 29.06.23 one each	Gegham 1	2	0	-	B1	6/29/23	208	7/3/23	791	4642	40	241	15	25	73	55	3.28	0.20	0.34	0.75	0.06	0.10	0.30	0.23
1178	TR.2 UN B1 H0 29.06.23 one each	Gegham 1	2	0	-	B1	6/29/23	209	7/3/23	862	4337	38	224	14	28	72	53	3.13	0.19	0.40	0.73	0.06	0.13	0.32	0.23
1179	TR.2 UN B1 H0 29.06.23 one each	Gegham 1	2	0	-	B1	6/29/23	210	7/3/23	757	4181	36	227	15	26	73	57	3.08	0.20	0.35	0.77	0.06	0.11	0.32	0.25
1180	TR.2 UN B1 H0 29.06.23 one each	Gegham 1	2	0	-	B1	6/29/23	211	7/3/23	762	4314	36	227	14	27	77	55	2.93	0.17	0.36	0.71	0.06	0.12	0.34	0.24
1181	TR.2 UN B1 H0 29.06.23 one each	Gegham 1	2	0	-	B1	6/29/23	212	7/3/23	979	5395	50	257	17	26	75	54	3.41	0.22	0.34	0.71	0.06	0.10	0.29	0.21
1182	TR.2 UN B1 H0 29.06.23 one each	Gegham 1	2	0	-	B1	6/29/23	213	7/3/23	925	4905	40	251	15	26	76	58	3.29	0.19	0.34	0.76	0.06	0.10	0.30	0.23
1183	TR.2 UN B1 H0 29.06.23 one each	Gegham 1	2	0	-	B1	6/29/23	214	7/3/23	798	4514	39	241	17	27	79	57	3.05	0.21	0.34	0.72	0.07	0.11	0.33	0.23
1184	TR.2 UN B1 H0 29.06.23 one each	Gegham 1	2	0	-	B1	6/29/23	215	7/3/23	771	4438	34	233	16	25	73	55	3.18	0.21	0.34	0.75	0.07	0.11	0.31	0.24
1185	TR.2 UN B1 H0 29.06.23 one each	Gegham 1	2	0	-	B1	6/29/23	217	7/3/23	844	4288	40	233	19	26	116	57	2.01	0.16	0.22	0.49	0.08	0.11	0.50	0.24
1186	TR.2 UN B1 H0 29.06.23 one each	Syunik	2	0	-	B1	6/29/23	218	7/3/23	627	6470	52	210	29	10	115	35	1.83	0.25	0.08	0.30	0.14	0.05	0.55	0.16
1187	TR2 UN-A1 H0 29.06.23 one each	Gegham 1	2	0	-	A1	6/29/23	219	7/3/23	757	4087	35	220	13	24	72	54	3.07	0.17	0.33	0.75	0.06	0.11	0.33	0.25
1188	TR2 UN-A1 H0 29.06.23 one each	Syunik	2	0	-	A1	6/29/23	220	7/3/23	430	4868	37	172	23	11	103	33	1.68	0.22	0.10	0.32	0.13	0.06	0.60	0.19
1189	TR2 UN-A1 H0 29.06.23 one each	Kelbadjar	2	0	-	A1	6/29/23	221	7/3/23	475	4905	41	158	18	11	113	33	1.39	0.15	0.09	0.29	0.11	0.07	0.72	0.21
1190	TR2 UN-A1 H0 29.06.23 one each	Gegham 1	2	0	-	A1	6/29/23	222	7/3/23	715	4095	34	219	16	24	73	55	2.98	0.21	0.32	0.75	0.07	0.11	0.34	0.25
1191	TR2 UN-A1 H0 29.06.23 one each	Syunik	2	0	-	A1	6/29/23	223	7/3/23	494	5417	39	189	22	11	105	35	1.80	0.21	0.10	0.33	0.11	0.06	0.55	0.18
1192	TR2 UN-A1 H0 29.06.23 one each	Gegham 1	2	0	-	A1	6/29/23	224	7/3/23	678	3897	32	213	13	26	74	58	2.86	0.17	0.34	0.78	0.06	0.12	0.35	0.27
1193	TR2 UN-A1 H0 29.06.23 one each	Kelbadjar	2	0	-	A1	6/29/23	225	7/3/23	525	5241	39	162	18	13	111	35	1.45	0.16	0.12	0.31	0.11	0.08	0.69	0.21
1194	TR2 UN-A1 H0 29.06.23 one each	Gegham 1	2	0	-	A1	6/29/23	226	7/3/23	695	3902	31	212	13	26	75	53	2.81	0.17	0.34	0.70	0.06	0.12	0.36	0.25
1195	TR2 UN-A1 H0 29.06.23 one each	Kelbadjar	2	0	-	A1	6/29/23	227	7/3/23	537	5662	44	172	18	11	115	39	1.50	0.15	0.10	0.34	0.10	0.07	0.67	0.23
1196	TR2 UN-A1 H0 29.06.23 one each	Syunik	2	0	-	A1	6/29/23	228	7/3/23	498	5297	40	182	25	11	106	37	1.72	0.23	0.10	0.35	0.13	0.06	0.58	0.21
1197	TR2 UN-A1 H0 29.06.23 one each	Syunik	2	0	-	A1	6/29/23	229	7/3/23	711	6957	54	196	19	9	123	36	1.59	0.15	0.07	0.29	0.09	0.04	0.63	0.18
1198	TR2 UN-A1 H0 29.06.23 one each	Kelbadjar	2	0	-	A1	6/29/23	230	7/3/23	541	5605	46	170	21	11	113	37	1.50	0.18	0.09	0.33	0.12	0.06	0.66	0.22
1199	TR2 UN-A1 H0 29.06.23 one each	Gegham 1	2	0	-	A1	6/29/23	231	7/3/23	715	4267	35	214	15	24	77	51	2.77	0.19	0.31	0.66	0.07	0.11	0.36	0.24
1200	TR2 UN-A1 H0 29.06.23 one each	Gegham 1	2	0	-	A1	6/29/23	232	7/3/23	746	4426	39	229	17	27	73	53	3.15	0.23	0.37	0.72	0.07	0.12	0.32	0.23
1201	TR2 UN-A1 H0 29.06.23 one each	Gegham 1	2	0	-	A1	6/29/23	233	7/3/23	668	4066	37	218	15	25	72	54	3.04	0.20	0.34	0.75	0.07	0.11	0.33	0.25
1202	TR2 UN-A1 H0 29.06.23 one each	Kelbadjar	2	0	-	A1	6/29/23	234	7/3/23	565	5829	46	175	18	11	109	37	1.60	0.16	0.10	0.34	0.10	0.06	0.62	0.21
1203	TR2 UN-A1 H0 29.06.23 one each	Kelbadjar	2	0	-	A1	6/29/23	235	7/3/23	529	5597	46	168	20	13	116	36	1.45	0.17	0.12	0.31	0.12	0.08	0.69	0.21
1204	TR2 UN-A1 H0 29.06.23 one each	Syunik	2	0	-	A1	6/29/23	236	7/3/23	552	5832	36	197	23	11	112	37	1.75	0.20	0.09	0.33	0.11	0.05	0.57	0.19
1205	TR2 UN-A1 H0 29.06.23 one each	Gegham 1	2	0	-	A1	6/29/23	237	7/3/23	799	4483	39	235	13	25	74	58	3.16	0.17	0.33	0.78	0.05	0.10	0.32	0.25
1206	TR2 UN-A1 H0 29.06.23 one each	Gegham 1	2	0	-	A1	6/29/23	238	7/3/23	744	4166	35	226	15	27	77	58	2.92	0.19	0.36	0.75	0.06	0.12	0.34	0.26
1207	TR2 UN-A1 H0 29.06.23 one each	Kelbadjar	2	0	-	A1	6/29/23	239	7/3/23	577	6106	46	177	23	10	129	36	1.37	0.17	0.07	0.28	0.13	0.05	0.73	0.20
1208	TR2 UN-A1 H0 29.06.23 one each	Gegham 1	2	0	-	A1	6/29/23	240	7/3/23	754	4286	35	233	16	25	74	54	3.12	0.21	0.33	0.72	0.07	0.11	0.32	0.23
1209	TR2 UN-A1 H0 29.06.23 one each	Gegham 1	2	0	-	A1	6/29/23	241	7/3/23	903	4641	41	238	15	27	75	53	3.16	0.19	0.35	0.70	0.06	0.11	0.32	0.22
1210	TR2 UN-A1 H0 29.06.23 one each	Kelbadjar	2	0	-	A1	6/29/23	242	7/3/23	516	5315	43	166	19	13	111	36	1.50	0.17	0.12	0.32	0.11	0.08	0.67	0.22
1211	TR2 UN-A1 H0 29.06.23 one each	Kelbadjar	2	0	-	A1	6/29/23	243	7/3/23	585	6019	48	179	19	11	113	37	1.58	0.16	0.10	0.33	0.10	0.06	0.63	0.21
1212	TR2 UN-A1 H0 29.06.23 one each	Gegham 1	2	0	-	A1	6/29/23	246	7/3/23	819	4493	42	249	18	29	82	58	3.04	0.21	0.36	0.71	0.07	0.12	0.33	0.23
1213	TR2 UN-A1 H0 29.06.23 one each	Kelbadjar	2	0	-	A1	6/29/23	248	7/3/23	552	5633	48	178	18	12	142	36	1.26	0.12	0.09	0.25	0.10	0.07	0.79	0.20
1214	Tr-2 H1S2 - H5 collapse one each	Kelbadjar	2	1 Sp2-H5	collapse	-	-	249	7/3/23	474	5080	53	164	25	9	113	36	1.44	0.22	0.08	0.32	0.15	0.05	0.69	0.22
1215	Tr-2 H1S2 - H5 collapse one each	Gegham 1	2	1 Sp2-H5	collapse	-	-	250	7/3/23	772	4307	35	227	14	25	73	57	3.08	0.18	0.34	0.77	0.06	0.11	0.32	0.25
1216	Tr-2 H1S2 - H5 collapse one each	Kelbadjar	2	1 Sp2-H5	collapse	-	-	251	7/3/23	503	5339	39	178	24	14	110	36	1.61	0.21	0.13	0.33	0.13	0.08	0.62	0.20
1217	Tr-2 H1S2 - H5 collapse one each	Gegham 1	2	1 Sp2-H5	collapse	-	-	252	7/3/23	748	4348	35	214	16	27	76	57	2.80	0.20	0.35	0.74	0.07	0.12	0.36	0.26
1218	Tr-2 H1S2 - H5 collapse one each	Kelbadjar	2	1 Sp2-H5	collapse	-	-	253	7/3/23	555	5774	40	172	18	11	113	37	1.52	0.15	0.10	0.33	0.10	0.07	0.66	0.22
1219	Tr-2 H1S2 - H5 collapse one each	Gegham 1	2	1 Sp2-H5	collapse	-	-	254	7/3/23	769	4183	33	223	15	27	77	55	2.88	0.19	0.36	0.71	0.07	0.12	0.35	0.25
1220	Tr-2 H1S2 - H5 collapse one each	Gegham 1	2	1 Sp2-H5	collapse	-	-	256	7/3/23	718	4008	38	217	11	25	73	54	2.95	0.16	0.34	0.73	0.05	0.11	0.34	0.25

1221	Tr-2 H1S2 - H5 collapse one each	Gutansar	2	1 Sp2-H5	collapse	-	-	257	7/3/23	701	9217	46	158	141	24	187	40	0.84	0.75	0.13	0.21	0.89	0.15	1.19	0.25
1222	Tr-2 H1S2 - H5 collapse one each	Kelbadjar	2	1 Sp2-H5	collapse	-	-	259	7/3/23	513	5478	44	167	16	12	113	39	1.48	0.14	0.11	0.34	0.09	0.07	0.68	0.23
1223	Tr-2 H1S2 - H5 collapse one each	Kelbadjar	2	1 Sp2-H5	collapse	-	-	260	7/3/23	506	5459	39	164	18	11	112	37	1.46	0.16	0.10	0.33	0.11	0.07	0.69	0.23
1224	Tr-2 H1S2 - H5 collapse one each	Kelbadjar	2	1 Sp2-H5	collapse	-	-	261	7/3/23	545	5973	46	174	25	12	114	40	1.53	0.21	0.11	0.35	0.14	0.07	0.66	0.23
1225	Tr-2 H1S2 - H5 collapse one each	Gegham 1	2	1 Sp2-H5	collapse	-	-	262	7/3/23	784	4214	38	232	14	26	74	58	3.11	0.18	0.34	0.78	0.06	0.11	0.32	0.25
1226	Tr-2 H1S2 - H5 collapse one each	Gegham 1	2	1 Sp2-H5	collapse	-	-	263	7/3/23	695	3807	35	208	14	27	74	58	2.80	0.18	0.36	0.78	0.06	0.13	0.36	0.28
1227	Tr-2 H1S2 - H5 collapse one each	Syunik	2	1 Sp2-H5	collapse	-	-	264	7/3/23	596	4525	40	223	10	9	94	43	2.36	0.11	0.09	0.45	0.05	0.04	0.42	0.19
1228	Tr-2 H1S2 - H5 collapse one each	Kelbadjar	2	1 Sp2-H5	collapse	-	-	265	7/3/23	530	5454	40	168	18	12	120	36	1.41	0.15	0.10	0.30	0.10	0.07	0.71	0.21
1229	Tr-2 H1S2 - H5 collapse one each	Kelbadjar	2	1 Sp2-H5	collapse	-	-	266	7/3/23	546	5593	46	167	17	12	117	33	1.43	0.14	0.11	0.28	0.10	0.07	0.70	0.20
1230	Tr-2 H1S2 - H5 collapse one each	Gegham 1	2	1 Sp2-H5	collapse	-	-	267	7/3/23	880	4712	46	247	14	27	77	55	3.20	0.17	0.36	0.71	0.05	0.11	0.31	0.22
1231	Tr-2 H1S2 - H5 collapse one each	Kelbadjar	2	1 Sp2-H5	collapse	-	-	268	7/3/23	540	5627	43	170	17	10	115	35	1.48	0.14	0.08	0.30	0.10	0.06	0.68	0.20
1232	Tr-2 H1S2 - H5 collapse one each	Gegham 1	2	1 Sp2-H5	collapse	-	-	269	7/3/23	872	4782	45	247	15	25	73	59	3.36	0.20	0.34	0.81	0.06	0.10	0.30	0.24
1233	Tr-2 H1S2 - H5 collapse one each	Syunik	2	1 Sp2-H5	collapse	-	-	270	7/3/23	519	5624	40	192	23	10	108	39	1.78	0.21	0.09	0.36	0.12	0.05	0.56	0.20
1234	Tr-2 H1S2 - H5 collapse one each	Gegham 1	2	1 Sp2-H5	collapse	-	-	271	7/3/23	814	4493	46	241	15	26	79	61	3.05	0.18	0.32	0.77	0.06	0.11	0.33	0.25
1235	Tr-2 H1S2 - H5 collapse one each	Arteni	2	1 Sp2-H5	collapse	-	-	272	7/3/23	574	4429	39	123	39	26	87	29	1.41	0.44	0.29	0.34	0.31	0.21	0.71	0.24
1236	Tr-2 H1S2 - H5 collapse one each	Syunik	2	1 Sp2-H5	collapse	-	-	273	7/3/23	561	6117	44	198	27	11	111	35	1.78	0.24	0.09	0.31	0.13	0.05	0.56	0.17
1237	Tr-2 H1S2 - H5 collapse one each	Kelbadjar	2	1 Sp2-H5	collapse	-	-	274	7/3/23	635	6460	50	181	18	9	116	36	1.56	0.15	0.07	0.31	0.10	0.05	0.64	0.20
1238	Tr-2 H1S2 - H5 collapse one each	Arteni	2	1 Sp2-H5	collapse	-	-	275	7/3/23	1005	5918	66	177	23	32	83	32	2.14	0.27	0.39	0.38	0.13	0.18	0.47	0.18
1239	Tr-2 H1S2 - H5 collapse one each	Syunik	2	1 Sp2-H5	collapse	-	-	276	7/3/23	753	7551	52	222	28	9	111	36	1.99	0.25	0.08	0.32	0.12	0.04	0.50	0.16
1240	Tr-2 Hor 2 Sp 1 U-A5 one each	Kelbadjar	2	2	1	A5	-	279	7/3/23	460	4966	38	154	17	11	107	35	1.44	0.15	0.10	0.33	0.11	0.07	0.69	0.23
1241	Tr-2 Hor 2 Sp 1 U-A5 one each	Arteni	2	2	1	A5	-	280	7/3/23	642	4535	41	140	26	28	87	33	1.62	0.29	0.33	0.38	0.18	0.20	0.62	0.24
1242	Tr-2 Hor 2 Sp 1 U-A5 one each	Gegham 1	2	2	1	A5	-	281	7/3/23	739	4079	34	216	13	27	77	57	2.80	0.16	0.34	0.73	0.06	0.12	0.36	0.26
1243	Tr-2 Hor 2 Sp 1 U-A5 one each	Gegham 1	2	2	1	A5	-	282	7/3/23	741	4164	35	224	13	27	78	57	2.86	0.16	0.35	0.72	0.06	0.12	0.35	0.25
1244	Tr-2 Hor 2 Sp 1 U-A5 one each	Gegham 1	2	2	1	A5	-	283	7/3/23	657	3804	41	211	16	26	73	54	2.91	0.21	0.35	0.74	0.07	0.12	0.34	0.26
1245	Tr-2 Hor 2 Sp 1 U-A5 one each	Gegham 1	2	2	1	A5	-	284	7/3/23	801	4373	38	226	15	27	78	58	2.89	0.19	0.34	0.74	0.06	0.12	0.35	0.26
1246	Tr-2 Hor 2 Sp 1 U-A5 one each	Kelbadjar	2	2	1	A5	-	285	7/3/23	542	5513	42	172	16	9	108	35	1.60	0.14	0.08	0.32	0.09	0.05	0.62	0.20
1247	Tr-2 Hor 2 Sp 1 U-A5 one each	Kelbadjar	2	2	1	A5	-	286	7/3/23	590	5874	44	176	18	13	133	36	1.32	0.13	0.10	0.27	0.10	0.08	0.76	0.20
1248	Tr-2 Hor 2 Sp 1 U-A5 one each	Gegham 1	2	2	1	A5	-	287	7/3/23	756	4128	35	221	11	23	73	51	3.00	0.16	0.31	0.70	0.05	0.10	0.33	0.23
1249	Tr-2 Hor 2 Sp 1 U-A5 one each	Gegham 1	2	2	1	A5	-	288	7/3/23	722	4109	35	219	13	27	74	55	2.94	0.17	0.36	0.74	0.06	0.12	0.34	0.25
1250	Tr-2 Hor 2 Sp 1 U-A5 one each	Gegham 1	2	2	1	A5	-	289	7/3/23	706	4110	35	218	14	26	72	55	3.04	0.19	0.36	0.77	0.06	0.12	0.33	0.25
1251	Tr-2 Hor 2 Sp 1 U-A5 one each	Gegham 1	2	2	1	A5	-	290	7/3/23	753	4246	35	227	15	26	75	55	3.01	0.19	0.34	0.73	0.06	0.11	0.33	0.24
1252	Tr-2 Hor 2 Sp 1 U-A5 one each	Gegham 1	2	2	1	A5	-	291	7/3/23	894	4965	41	255	15	27	80	57	3.18	0.18	0.33	0.71	0.06	0.10	0.31	0.22
1253	Tr-2 Hor 2 Sp 1 U-A5 one each	Kelbadjar	2	2	1	A5	-	293	7/3/23	633	6369	50	174	17	12	120	39	1.45	0.14	0.10	0.32	0.09	0.07	0.69	0.22
1254	Tr-2 Hor 2 Sp 1 U-A5 one each	Gegham 1	2	2	1	A5	-	294	7/3/23	746	4070	35	220	15	27	73	54	2.99	0.20	0.36	0.73	0.07	0.12	0.33	0.25
1255	Tr-2 Hor 2 Sp 1 U-A5 one each	Gegham 1	2	2	1	A5	-	295	7/3/23	974	5115	46	258	16	27	76	57	3.38	0.20	0.35	0.74	0.06	0.10	0.30	0.22
1256	Tr-2 Hor 2 Sp 1 U-A5 one each	Gegham 1	2	2	1	A5	-	296	7/3/23	783	4228	40	227	13	25	73	55	3.08	0.17	0.34	0.75	0.06	0.11	0.32	0.24
1257	Tr-2 Hor 2 Sp 1 U-A5 one each	Gegham 1	2	2	1	A5	-	297	7/3/23	864	4745	40	249	17	26	73	54	3.39	0.22	0.35	0.73	0.07	0.10	0.30	0.22
1258	Tr-2 Hor 2 Sp 1 U-A5 one each	Gegham 1	2	2	1	A5	-	298	7/3/23	781	4440	40	236	15	26	75	55	3.14	0.19	0.34	0.73	0.06	0.11	0.32	0.23
1259	Tr-2 Hor 2 Sp 1 U-A5 one each	Gegham 1	2	2	1	A5	-	299	7/3/23	894	5176	46	259	15	25	75	59	3.43	0.19	0.33	0.79	0.06	0.10	0.29	0.23
1260	Tr-2 Hor 2 Sp 1 U-A5 one each	Kelbadjar	2	2	1	A5	-	300	7/3/23	616	6300	50	181	17	10	115	37	1.57	0.14	0.08	0.33	0.09	0.05	0.64	0.21
1261	Tr-2 Hor 2 Sp 1 U-A5 one each	Gegham 1	2	2	1	A5	-	301	7/3/23	920	5269	49	265	15	24	79	57	3.34	0.18	0.30	0.72	0.05	0.09	0.30	0.21
1262	Tr-2 Hor 2 Sp 1 U-A5 one each	Kelbadjar	2	2	1	A5	-	302	7/3/23	587	5744	48	177	17	11	117	35	1.51	0.14	0.09	0.30	0.09	0.06	0.66	0.20
1263	Tr-2 Hor 2 Sp 1 U-A5 one each	Syunik	2	2	1	A5	-	303	7/3/23	791	6199	59	276	9	6	120	47	2.31	0.08	0.05	0.39	0.03	0.02	0.43	0.17
1264	Tr-2 Hor 2 Sp 1 U-A5 one each	Gegham 1	2	2	1	A5	-	304	7/3/23	743	4174	36	219	15	26	73	55	3.02	0.20	0.35	0.76	0.07	0.12	0.33	0.25
1265	Tr-2 Hor 2 Sp 1 U-A5 one each	Kelbadjar	2	2	1	A5	-	305	7/3/23	580	5782	48	173	17	11	112	39	1.54	0.15	0.09	0.35	0.10	0.06	0.65	0.22
1266	Tr-2 Hor 2 Sp 1 U-A5 one each	Gegham 1	2	2	1	A5	-	306	7/3/23	784	4150	36	221	15	26	77	57	2.86	0.19	0.33	0.73	0.07	0.12	0.35	0.26
1267	Tr-2 Hor 2 Sp 1 U-A5 one each	Gegham 1	2	2	1	A5	-	307	7/3/23	819	4405	43	245	15	27	81	59	3.02	0.18	0.33	0.73	0.06	0.11	0.33	0.24
1268	TR.2 A1 H1S1 [S4] 03.07 1 of 1	Arteni	2	1	1	A1	7/3/23	1	7/4/23	561	3942	35	124	26	23	81	25	1.53	0.31	0.28	0.31	0.21	0.18	0.66	0.20
1269	Tr2 A4 H3 S2 30.06.23 one each	Gegham 1	2	3	2	A4	6/30/23	3	7/4/23	690	4002	31	212	14	26	73	53	2.92	0.19	0.35	0.72	0.06	0.12	0.34	0.25
1270	Tr2 A4 H3 S2 30.06.23 one each	Gegham 1	2	3	2	A4	6/30/23	4	7/4/23	676	4037	37	216	18	25	75	53	2.87	0.23	0.33	0.70	0.08	0.11	0.35	0.24
1271	Tr2 A4 H3 S2 30.06.23 one each	Gegham 1	2	3	2	A4	6/30/23	5	7/4/23	742	4175	32	218	13	25	73	55	2.97	0.17	0.34	0.75	0.06	0.11	0.34	0.25

1272	Tr2 A4 H3 S2 30.06.23 one each	Gegham 1	2	3	2	A4	6/30/23	6	7/4/23	688	3776	28	208	13	26	75	54	2.76	0.17	0.34	0.71	0.06	0.12	0.36	0.26
1273	Tr2 A4 H3 S2 30.06.23 one each	Kelbadjar	2	3	2	A4	6/30/23	7	7/4/23	501	5417	41	166	17	10	118	33	1.41	0.14	0.08	0.28	0.10	0.06	0.71	0.20
1274	Tr2 A4 H3 S2 30.06.23 one each	Gegham 1	2	3	2	A4	6/30/23	8	7/4/23	744	4144	35	222	11	26	77	55	2.87	0.15	0.33	0.71	0.05	0.12	0.35	0.25
1275	Tr2 A4 H3 S2 30.06.23 one each	Gegham 1	2	3	2	A4	6/30/23	9	7/4/23	706	4156	34	223	16	27	73	55	3.03	0.21	0.36	0.75	0.07	0.12	0.33	0.25
1276	Tr2 A4 H3 S2 30.06.23 one each	Syunik	2	3	2	A4	6/30/23	10	7/4/23	476	5152	37	180	19	11	106	36	1.70	0.18	0.11	0.34	0.10	0.06	0.59	0.20
1277	Tr2 A4 H3 S2 30.06.23 one each	Gegham 1	2	3	2	A4	6/30/23	11	7/4/23	711	4053	35	214	13	26	74	54	2.88	0.17	0.34	0.72	0.06	0.12	0.35	0.25
1278	Tr2 A4 H3 S2 30.06.23 one each	Gegham 1	2	3	2	A4	6/30/23	12	7/4/23	645	3734	34	206	14	27	75	55	2.74	0.18	0.35	0.73	0.07	0.13	0.37	0.27
1279	Tr2 A4 H3 S2 30.06.23 one each	Kelbadjar	2	3	2	A4	6/30/23	13	7/4/23	547	5775	47	173	17	10	108	33	1.60	0.15	0.09	0.31	0.10	0.06	0.63	0.19
1280	Tr2 A4 H3 S2 30.06.23 one each	Gegham 1	2	3	2	A4	6/30/23	14	7/4/23	696	3861	36	222	13	27	73	58	3.02	0.17	0.37	0.79	0.06	0.12	0.33	0.26
1281	Tr2 A4 H3 S2 30.06.23 one each	Gegham 1	2	3	2	A4	6/30/23	15	7/4/23	697	3929	33	210	15	26	77	55	2.72	0.19	0.33	0.71	0.07	0.12	0.37	0.26
1282	Tr2 A4 H3 S2 30.06.23 one each	Kelbadjar	2	3	2	A4	6/30/23	16	7/4/23	489	5261	40	164	17	11	111	39	1.48	0.15	0.10	0.35	0.10	0.07	0.68	0.24
1283	Tr2 A4 H3 S2 30.06.23 one each	Gegham 1	2	3	2	A4	6/30/23	17	7/4/23	909	4753	39	244	15	26	77	62	3.16	0.19	0.33	0.80	0.06	0.10	0.32	0.25
1284	TR.2 collapse A2-A3 01.07.23 1 of 4	Kelbadjar	2	-	collapse	A2-A3	7/1/23	18	7/4/23	444	4452	33	145	15	12	108	33	1.35	0.13	0.12	0.31	0.10	0.09	0.74	0.23
1285	TR.2 collapse A2-A3 01.07.23 1 of 4	Kelbadjar	2	-	collapse	A2-A3	7/1/23	20	7/4/23	484	4805	35	154	16	12	108	36	1.42	0.14	0.11	0.33	0.10	0.08	0.71	0.23
1286	TR.2 collapse A2-A3 01.07.23 2 of 4	Kelbadjar	2	-	collapse	A2-A3	7/1/23	21	7/4/23	463	4909	33	167	25	13	103	33	1.63	0.24	0.13	0.32	0.15	0.08	0.61	0.20
1287	TR.2 collapse A2-A3 01.07.23 3 of 4	Arteni	2	-	collapse	A2-A3	7/1/23	23	7/4/23	599	4475	36	137	29	27	82	31	1.67	0.35	0.32	0.37	0.21	0.19	0.60	0.22
1288	TR.2 collapse A2-A3 01.07.23 4 of 4	Gegham 1	2	-	collapse	A2-A3	7/1/23	25	7/4/23	757	4199	38	223	14	23	74	55	2.99	0.18	0.31	0.74	0.06	0.10	0.33	0.25
1289	TR.2 A2-A3 H5 S1 01.07.23 one each	Gegham 1	2	5	1	A2-A3	7/1/23	27	7/4/23	657	3735	32	207	13	27	71	53	2.93	0.18	0.38	0.74	0.06	0.13	0.34	0.25
1290	TR.2 A2-A3 H5 S1 01.07.23 one each	Gegham 1	2	5	1	A2-A3	7/1/23	28	7/4/23	660	3854	33	209	11	27	71	54	2.96	0.16	0.38	0.76	0.05	0.13	0.34	0.26
1291	TR.2 A2-A3 H5 S1 01.07.23 one each	Syunik	2	5	1	A2-A3	7/1/23	29	7/4/23	483	5003	38	178	22	9	96	31	1.85	0.22	0.09	0.32	0.12	0.05	0.54	0.17
1292	TR.2 A2-A3 H5 S1 01.07.23 one each	Gegham 1	2	5	1	A2-A3	7/1/23	30	7/4/23	722	4063	34	217	14	25	72	55	3.03	0.19	0.34	0.77	0.06	0.11	0.33	0.25
1293	TR.2 A2-A3 H5 S1 01.07.23 one each	Gegham 1	2	5	1	A2-A3	7/1/23	31	7/4/23	783	4300	38	231	14	27	74	58	3.10	0.18	0.37	0.78	0.06	0.12	0.32	0.25
1294	TR.2 A2-A3 H5 S1 01.07.23 one each	Gegham 1	2	5	1	A2-A3	7/1/23	32	7/4/23	667	3681	31	206	11	27	76	55	2.70	0.15	0.36	0.72	0.06	0.13	0.37	0.27
1295	TR.2 A2-A3 H5 S1 01.07.23 one each	Gegham 1	2	5	1	A2-A3	7/1/23	33	7/4/23	822	4552	38	242	15	27	78	55	3.10	0.19	0.35	0.71	0.06	0.11	0.32	0.23
1296	TR.2 A2-A3 H5 S1 01.07.23 one each	Gegham 1	2	5	1	A2-A3	7/1/23	34	7/4/23	753	4243	39	222	14	27	78	61	2.84	0.17	0.35	0.78	0.06	0.12	0.35	0.27
1297	TR.2 A2-A3 H5 S1 01.07.23 one each	Gegham 1	2	5	1	A2-A3	7/1/23	35	7/4/23	763	4261	35	225	14	27	74	58	3.02	0.18	0.36	0.78	0.06	0.12	0.33	0.26
1298	TR.2 A2-A3 H5 S1 01.07.23 one each	Gegham 1	2	5	1	A2-A3	7/1/23	36	7/4/23	792	4359	37	230	13	25	73	58	3.12	0.17	0.34	0.79	0.05	0.11	0.32	0.25
1299	TR.2 A2-A3 H5 S1 01.07.23 one each	Gegham 1	2	5	1	A2-A3	7/1/23	37	7/4/23	819	4535	40	238	15	27	80	58	2.98	0.18	0.33	0.72	0.06	0.11	0.34	0.24
1300	TR.2 A2-A3 H5 S1 01.07.23 one each	Gegham 1	2	5	1	A2-A3	7/1/23	39	7/4/23	802	4300	44	228	15	25	74	55	3.06	0.19	0.33	0.74	0.06	0.11	0.33	0.24
1301	TR.2 A2-A3 H5 S1 01.07.23 one each	Gegham 1	2	5	1	A2-A3	7/1/23	40	7/4/23	637	3465	35	206	13	27	73	55	2.81	0.17	0.36	0.75	0.06	0.13	0.36	0.27
1302	Tr2 A2-A3 H5 Sp2 01.07.23 one each	Gegham 1	2	5	1	A2-A3	7/1/23	47	7/4/23	774	4191	28	219	15	27	73	54	3.02	0.20	0.37	0.74	0.07	0.12	0.33	0.25
1303	Tr2 A2-A3 H5 Sp2 01.07.23 one each	Gegham 1	2	5	1	A2-A3	7/1/23	49	7/4/23	799	4470	39	229	16	25	74	55	3.07	0.21	0.33	0.74	0.07	0.11	0.33	0.24
1304	Tr2 A2-A3 H5 Sp2 01.07.23 one each	Gegham 1	2	5	1	A2-A3	7/1/23	50	7/4/23	867	4586	43	243	16	27	78	58	3.11	0.20	0.34	0.74	0.06	0.11	0.32	0.24
1305	Tr2 A2-A3 H5 Sp2 01.07.23 one each	Gegham 1	2	5	1	A2-A3	7/1/23	51	7/4/23	929	5183	55	256	15	26	76	57	3.35	0.19	0.34	0.74	0.06	0.10	0.30	0.22
1306	Tr2 A2-A3 H5 Sp2 01.07.23 one each	Gegham 1	2	5	1	A2-A3	7/1/23	52	7/4/23	885	4707	43	245	15	27	74	58	3.29	0.19	0.36	0.78	0.06	0.11	0.30	0.24
1307	Tr2 A2-A3 H5 Sp2 01.07.23 one each	Kelbadjar	2	5	1	A2-A3	7/1/23	53	7/4/23	465	4849	42	161	18	11	124	36	1.30	0.14	0.09	0.29	0.11	0.07	0.77	0.22
1308	TR.2 A4 H5 Sp.1 1.07.23 one each	Gegham 1	2	5	1	A4	7/1/23	54	7/4/23	799	4600	39	235	15	26	78	55	3.01	0.19	0.33	0.71	0.06	0.11	0.33	0.23
1309	TR.2 A4 H5 Sp.1 1.07.23 one each	Syunik	2	5	1	A4	7/1/23	55	7/4/23	617	6456	41	207	24	8	108	36	1.91	0.22	0.07	0.33	0.11	0.04	0.52	0.17
1310	TR.2 A4 H5 Sp.1 1.07.23 one each	Gegham 1	2	5	1	A4	7/1/23	56	7/4/23	804	4365	36	233	14	25	71	54	3.30	0.19	0.35	0.76	0.06	0.11	0.30	0.23
1311	TR.2 A4 H5 Sp.1 1.07.23 one each	Gegham 1	2	5	1	A4	7/1/23	57	7/4/23	701	4135	36	221	16	26	76	54	2.89	0.20	0.34	0.71	0.07	0.12	0.35	0.24
1312	TR.2 A4 H5 Sp.1 1.07.23 one each	Gegham 1	2	5	1	A4	7/1/23	58	7/4/23	906	4927	41	243	15	28	76	58	3.19	0.19	0.37	0.76	0.06	0.12	0.31	0.24
1313	TR.2 A4 H5 Sp.1 1.07.23 one each	Syunik	2	5	1	A4	7/1/23	59	7/4/23	706	7874	57	226	27	13	114	33	1.98	0.23	0.12	0.29	0.12	0.06	0.51	0.15
1314	TR.2 A4 H5 Sp.1 1.07.23 one each	Syunik	2	5	1	A4	7/1/23	60	7/4/23	672	7165	53	228	23	10	102	36	2.23	0.22	0.09	0.35	0.10	0.04	0.45	0.16
1315	Tr2 Unit A4 H4 Sp2 01.07.23 one each	Syunik	2	4	2	A4	7/1/23	63	7/4/23	560	5759	36	187	25	10	106	35	1.77	0.23	0.09	0.33	0.13	0.05	0.57	0.19
1316	Tr2 Unit A4 H4 Sp2 01.07.23 one each	Gegham 1	2	4	2	A4	7/1/23	64	7/4/23	728	4081	37	221	15	26	76	57	2.89	0.19	0.34	0.74	0.07	0.12	0.35	0.26
1317	Tr2 Unit A4 H4 Sp2 01.07.23 one each	Gegham 1	2	4	2	A4	7/1/23	65	7/4/23	825	4581	40	234	17	24	73	54	3.19	0.22	0.32	0.73	0.07	0.10	0.31	0.23
1318	Tr2 Unit A4 H4 Sp2 01.07.23 one each	Gegham 1	2	4	2	A4	7/1/23	66	7/4/23	832	4507	39	233	16	26	73	55	3.20	0.21	0.35	0.76	0.07	0.11	0.31	0.24
1319	Tr2 Unit A4 H4 Sp2 01.07.23 one each	Arteni	2	4	2	A4	7/1/23	67	7/4/23	616	4915	42	132	39	27	86	29	1.54	0.45	0.31	0.34	0.29	0.20	0.65	0.22
1320	Tr2 Unit A4 H4 Sp2 01.07.23 one each	Gegham 1	2	4	2	A4	7/1/23	68	7/4/23	840	4855	46	240	15	27	77	54	3.11	0.19	0.36	0.70	0.06	0.11	0.32	0.22
1321	Tr.2 Unit B1 H1 Sp1 one each	Gegham 1	2	1	1	B1	-	69	7/4/23	663	3716	33	211	14	24	72	53	2.95	0.19	0.33	0.73	0.06	0.11	0.34	0.25
1322	Tr.2 Unit B1 H1 Sp1 one each	Kelbadjar	2	1	1	B1	-	70	7/4/23	498	5074	36	161	17	11	116	33	1.38	0.14	0.10	0.29	0.10	0.07	0.72	0.21

1323	Tr.2 Unit B1 H1 Sp1 one each	Gegham 1	2	1	1	B1	-	71	7/4/23	772	4199	35	225	14	27	73	57	3.06	0.18	0.36	0.77	0.06	0.12	0.33	0.25
1324	Tr.2 Unit B1 H1 Sp1 one each	Gegham 1	2	1	1	B1	-	72	7/4/23	733	3842	35	227	9	27	63	57	3.59	0.15	0.44	0.90	0.04	0.12	0.28	0.25
1325	Tr.2 Unit B1 H1 Sp1 one each	Kelbadjar	2	1	1	B1	-	73	7/4/23	451	4784	40	154	18	11	108	33	1.43	0.16	0.11	0.31	0.11	0.07	0.70	0.22
1326	Tr.2 Unit B1 H1 Sp1 one each	Kelbadjar	2	1	1	B1	-	74	7/4/23	526	5353	41	163	16	10	108	35	1.50	0.14	0.09	0.32	0.10	0.06	0.67	0.21
1327	Tr.2 Unit B1 H1 Sp1 one each	Kelbadjar	2	1	1	B1	-	75	7/4/23	488	4922	40	160	19	10	105	35	1.52	0.18	0.09	0.33	0.12	0.06	0.66	0.22
1328	Tr.2 Unit B1 H1 Sp1 one each	Kelbadjar	2	1	1	B1	-	76	7/4/23	499	5403	47	164	26	12	112	39	1.47	0.23	0.11	0.35	0.16	0.08	0.68	0.24
1329	Tr.2 Unit B1 H1 Sp1 one each	Kelbadjar	2	1	1	B1	-	77	7/4/23	559	5733	44	169	17	10	112	36	1.51	0.15	0.09	0.32	0.10	0.06	0.66	0.21
1330	Tr.2 Unit B1 H1 Sp1 one each	Gegham 1	2	1	1	B1	-	79	7/4/23	757	4233	36	226	15	27	74	57	3.03	0.19	0.36	0.76	0.06	0.12	0.33	0.25
1331	Tr.2 Unit B1 H1 Sp1 one each	Gegham 1	2	1	1	B1	-	80	7/4/23	835	4952	46	256	14	22	76	55	3.35	0.18	0.29	0.72	0.05	0.09	0.30	0.22
1332	Tr.2 Unit B1 H1 Sp1 one each	Gegham 1	2	1	1	B1	-	81	7/4/23	692	4006	34	219	14	27	76	57	2.87	0.18	0.36	0.74	0.06	0.13	0.35	0.26
1333	Tr.2 Unit B1 H1 Sp1 one each	Gegham 1	2	1	1	B1	-	82	7/4/23	712	3996	35	217	13	23	73	58	2.99	0.17	0.31	0.80	0.06	0.10	0.33	0.27
1334	Tr.2 Unit B1 H1 Sp1 one each	Gegham 1	2	1	1	B1	-	83	7/4/23	736	3944	33	217	13	27	74	54	2.91	0.17	0.37	0.72	0.06	0.13	0.34	0.25
1335	Tr.2 Unit B1 H1 Sp1 one each	Syunik	2	1	1	B1	-	84	7/4/23	602	6262	46	184	18	9	114	37	1.61	0.15	0.08	0.33	0.10	0.05	0.62	0.20
1336	Tr.2 Unit B1 H1 Sp1 one each	Gegham 1	2	1	1	B1	-	85	7/4/23	783	4586	36	232	17	23	79	55	2.92	0.21	0.29	0.70	0.07	0.10	0.34	0.24
1337	Tr.2 Unit B1 H1 Sp1 one each	Gegham 1	2	1	1	B1	-	86	7/4/23	727	4038	33	215	14	27	73	55	2.93	0.18	0.36	0.75	0.06	0.12	0.34	0.26
1338	Tr.2 Unit B1 H1 Sp1 one each	Gegham 1	2	1	1	B1	-	87	7/4/23	858	4661	43	233	15	27	75	53	3.08	0.19	0.35	0.70	0.06	0.11	0.32	0.23
1339	Tr.2 Unit B1 H1 Sp1 one each	Kelbadjar	2	1	1	B1	-	88	7/4/23	539	5624	44	168	17	11	108	37	1.55	0.15	0.10	0.34	0.10	0.06	0.64	0.22
1340	Tr.2 Unit B1 H1 Sp1 one each	Gegham 1	2	1	1	B1	-	89	7/4/23	818	4407	38	234	15	27	76	57	3.07	0.19	0.35	0.74	0.06	0.11	0.33	0.24
1341	Tr.2 Unit B1 H1 Sp1 one each	Gegham 1	2	1	1	B1	-	90	7/4/23	904	4879	43	249	14	24	74	55	3.35	0.18	0.32	0.74	0.05	0.10	0.30	0.22
1342	Tr.2 Unit B1 H1 Sp1 one each	Gegham 1	2	1	1	B1	-	91	7/4/23	741	4033	36	219	14	25	79	57	2.77	0.17	0.31	0.72	0.06	0.11	0.36	0.26
1343	Tr.2 Unit B1 H1 Sp1 one each	Gegham 1	2	1	1	B1	-	92	7/4/23	847	4751	41	245	14	26	75	57	3.25	0.18	0.34	0.75	0.06	0.10	0.31	0.23
1344	Tr.2 Unit B1 H1 Sp1 one each	Gegham 1	2	1	1	B1	-	93	7/4/23	839	4537	44	236	14	26	74	58	3.18	0.18	0.34	0.78	0.06	0.11	0.31	0.25
1345	Tr.2 Unit B1 H1 Sp1 one each	Gegham 1	2	1	1	B1	-	94	7/4/23	738	4144	34	219	14	27	75	57	2.90	0.18	0.36	0.75	0.06	0.13	0.34	0.26
1346	Tr.2 Unit B1 H1 Sp1 one each	Gegham 1	2	1	1	B1	-	95	7/4/23	802	4507	40	235	14	26	74	57	3.16	0.18	0.34	0.76	0.06	0.11	0.32	0.24
1347	Tr.2 Unit B1 H1 Sp1 one each	Gegham 1	2	1	1	B1	-	96	7/4/23	738	4241	39	227	14	27	73	55	3.08	0.18	0.36	0.75	0.06	0.12	0.32	0.24
1348	Tr.2 Unit B1 H1 Sp1 one each	Kelbadjar	2	1	1	B1	-	97	7/4/23	566	5831	42	172	17	11	118	37	1.46	0.14	0.09	0.32	0.10	0.06	0.68	0.22
1349	Tr.2 Unit B1 H1 Sp1 one each	Syunik	2	1	1	B1	-	98	7/4/23	525	5420	37	196	22	11	106	35	1.85	0.20	0.10	0.33	0.11	0.05	0.54	0.18
1350	Tr.2 Unit B1 H1 Sp1 one each	Gegham 1	2	1	1	B1	-	99	7/4/23	777	4176	35	226	14	24	72	53	3.15	0.19	0.33	0.73	0.06	0.11	0.32	0.23
1351	Tr.2 Unit B1 H1 Sp1 one each	Gegham 1	2	1	1	B1	-	100	7/4/23	911	5062	46	257	14	27	79	57	3.24	0.17	0.34	0.72	0.05	0.10	0.31	0.22
1352	Tr.2 Unit B1 H1 Sp1 one each	Gegham 1	2	1	1	B1	-	101	7/4/23	807	4577	38	238	18	24	75	53	3.16	0.23	0.31	0.70	0.07	0.10	0.32	0.22
1353	Tr.2 Unit B1 H1 Sp1 one each	Gegham 1	2	1	1	B1	-	102	7/4/23	793	4419	41	224	13	26	73	57	3.04	0.17	0.35	0.77	0.06	0.11	0.33	0.25
1354	Tr.2 Unit B1 H1 Sp1 one each	Gegham 1	2	1	1	B1	-	103	7/4/23	928	5127	46	254	16	27	76	59	3.33	0.20	0.35	0.78	0.06	0.10	0.30	0.23
1355	Tr.2 Unit B1 H1 Sp1 one each	Gegham 1	2	1	1	B1	-	104	7/4/23	705	4275	39	221	15	24	73	55	3.00	0.20	0.32	0.75	0.07	0.11	0.33	0.25
1356	Tr.2 Unit B1 H1 Sp1 one each	Gegham 1	2	1	1	B1	-	105	7/4/23	869	4735	42	241	15	27	78	58	3.08	0.19	0.34	0.74	0.06	0.11	0.32	0.24
1357	Tr.2 Unit B1 H1 Sp1 one each	Gegham 1	2	1	1	B1	-	106	7/4/23	858	4891	44	242	16	26	76	55	3.17	0.20	0.34	0.72	0.06	0.11	0.32	0.23
1358	Tr.2 Unit B1 H1 Sp1 one each	Gegham 1	2	1	1	B1	-	107	7/4/23	815	4569	43	237	15	26	79	55	3.00	0.18	0.32	0.70	0.06	0.11	0.33	0.23
1359	Tr.2 Unit B1 H1 Sp1 one each	Gegham 1	2	1	1	B1	-	108	7/4/23	828	4670	41	244	18	27	77	54	3.16	0.23	0.36	0.70	0.07	0.11	0.32	0.22
1360	Tr.2 Unit B1 H1 Sp1 one each	Gegham 1	2	1	1	B1	-	111	7/4/23	824	4617	42	247	14	26	73	54	3.36	0.18	0.35	0.73	0.05	0.10	0.30	0.22
1361	Tr2 A5 H5 S2 01.07.22 one each	Syunik	2	5	2	A5	7/1/22	112	7/4/23	498	5394	35	184	25	10	108	33	1.71	0.23	0.09	0.31	0.13	0.05	0.58	0.18
1362	Tr2 A5 H5 S2 01.07.22 one each	Arteni	2	5	2	A5	7/1/22	113	7/4/23	627	4612	37	135	27	26	83	32	1.63	0.32	0.31	0.38	0.20	0.19	0.61	0.24
1363	Tr2 A5 H5 S2 01.07.22 one each	Gegham 1	2	5	2	A5	7/1/22	114	7/4/23	641	3702	35	208	18	27	76	54	2.73	0.23	0.35	0.71	0.08	0.13	0.37	0.26
1364	Tr2 A5 H5 S2 01.07.22 one each	Syunik	2	5	2	A5	7/1/22	115	7/4/23	520	5460	38	192	23	12	107	35	1.80	0.21	0.12	0.33	0.12	0.06	0.56	0.18
1365	Tr2 A5 H5 S2 01.07.22 one each	Gegham 1	2	5	2	A5	7/1/22	116	7/4/23	671	3854	34	203	15	25	76	57	2.66	0.19	0.32	0.74	0.07	0.12	0.38	0.28
1366	Tr2 A5 H5 S2 01.07.22 one each	Syunik	2	5	2	A5	7/1/22	117	7/4/23	485	5266	37	181	25	12	109	36	1.65	0.22	0.11	0.33	0.14	0.07	0.60	0.20
1367	Tr2 A5 H5 S2 01.07.22 one each	Syunik	2	5	2	A5	7/1/22	118	7/4/23	519	5943	43	188	28	13	111	32	1.69	0.25	0.12	0.29	0.15	0.07	0.59	0.17
1368	Tr2 A5 H5 S2 01.07.22 one each	Kelbadjar	2	5	2	A5	7/1/22	119	7/4/23	531	5605	41	170	18	10	114	36	1.49	0.15	0.08	0.32	0.10	0.06	0.67	0.21
1369	Tr2 A5 H5 S2 01.07.22 one each	Syunik	2	5	2	A5	7/1/22	120	7/4/23	665	7107	53	211	29	7	106	31	2.00	0.27	0.06	0.29	0.14	0.03	0.50	0.14
1370	Tr2 A5 H2 S2 30.06.23 one each	Gegham 1	2	5	2	A5	7/1/22	122	7/4/23	697	3970	32	213	15	25	76	55	2.79	0.19	0.32	0.72	0.07	0.12	0.36	0.26
1371	Tr2 A5 H2 S2 30.06.23 one each	Gegham 1	2	5	2	A5	7/1/22	123	7/4/23	848	4606	42	235	15	26	78	61	3.01	0.19	0.33	0.78	0.06	0.11	0.33	0.26
1372	Tr2 A5 H2 S2 30.06.23 one each	Gegham 1	2	5	2	A5	7/1/22	124	7/4/23	803	4678	39	238	15	24	74	54	3.20	0.19	0.32	0.72	0.06	0.10	0.31	0.23
1373	Tr2 A5 H2 S2 30.06.23 one each	Arteni	2	5	2	A5	7/1/22	125	7/4/23	803	4700	45	157	22	29	89	35	1.77	0.24	0.33	0.39	0.14	0.19	0.57	0.22

1374	Tr2 A5 H2 S2 30.06.23 one each	Gegham 1	2	5	2	A5	7/1/22	126	7/4/23	854	4551	36	241	14	24	74	57	3.24	0.18	0.32	0.76	0.06	0.10	0.31	0.23
1375	Tr2 A5 H2 S2 30.06.23 one each	Gegham 1	2	5	2	A5	7/1/22	127	7/4/23	744	4155	35	223	15	27	73	57	3.03	0.20	0.37	0.77	0.07	0.12	0.33	0.25
1376	Tr2 A5 H2 S2 30.06.23 one each	Gegham 1	2	5	2	A5	7/1/22	128	7/4/23	869	4785	42	241	15	27	76	55	3.16	0.19	0.35	0.72	0.06	0.11	0.32	0.23
1377	Tr2 A5 H2 S2 30.06.23 one each	Kelbadjar	2	5	2	A5	7/1/22	129	7/4/23	587	5970	51	183	18	11	121	36	1.51	0.15	0.09	0.30	0.10	0.06	0.66	0.20
1378	TR.2 Unit-B0 TS (H0) 01.07.23 one each	Gegham 1	2	0	-	B0	7/1/23	130	7/4/23	690	3942	35	217	18	27	76	58	2.84	0.23	0.36	0.76	0.08	0.13	0.35	0.27
1379	TR.2 Unit-B0 TS (H0) 01.07.23 one each	Gegham 1	2	0	-	B0	7/1/23	131	7/4/23	661	3785	32	202	14	25	72	53	2.83	0.19	0.34	0.73	0.07	0.12	0.35	0.26
1380	TR.2 Unit-B0 TS (H0) 01.07.23 one each	Syunik	2	0	-	B0	7/1/23	132	7/4/23	481	5265	36	182	24	11	107	35	1.71	0.22	0.10	0.33	0.13	0.06	0.59	0.19
1381	TR.2 Unit-B0 TS (H0) 01.07.23 one each	Gegham 1	2	0	-	B0	7/1/23	133	7/4/23	656	3729	32	206	15	25	73	57	2.81	0.20	0.34	0.77	0.07	0.12	0.36	0.27
1382	TR.2 Unit-B0 TS (H0) 01.07.23 one each	Gegham 1	2	0	-	B0	7/1/23	135	7/4/23	724	3870	36	220	15	28	73	55	2.99	0.20	0.39	0.75	0.07	0.13	0.33	0.25
1383	TR.2 Unit-B0 TS (H0) 01.07.23 one each	Kelbadjar	2	0	-	B0	7/1/23	136	7/4/23	529	5417	43	164	16	11	113	36	1.45	0.14	0.10	0.32	0.09	0.07	0.69	0.22
1384	TR.2 Unit-B0 TS (H0) 01.07.23 one each	Khorapor	2	0	-	B0	7/1/23	1	7/5/23	541	4575	38	242	7	19	89	37	2.73	0.08	0.21	0.42	0.03	0.08	0.37	0.15
1385	TR.2 Unit-B0 TS (H0) 01.07.23 one each	Gegham 1	2	0	-	B0	7/1/23	2	7/5/23	875	4672	46	245	15	26	77	61	3.17	0.19	0.33	0.79	0.06	0.10	0.32	0.25
1386	Tr2 A5 H5 S1 29.06.23 one each	Syunik	2	5	1	A5	6/29/23	5	7/5/23	523	5699	38	182	26	11	116	37	1.57	0.22	0.10	0.32	0.14	0.06	0.64	0.21
1387	Tr2 A5 H5 S1 29.06.23 one each	Gegham 1	2	5	1	A5	6/29/23	6	7/5/23	722	4029	37	211	15	26	73	53	2.87	0.20	0.35	0.71	0.07	0.12	0.35	0.25
1388	Tr2 A5 H5 S1 29.06.23 one each	Gegham 1	2	5	1	A5	6/29/23	7	7/5/23	833	4454	38	233	14	24	73	54	3.22	0.19	0.33	0.74	0.06	0.10	0.31	0.23
1389	Tr2 A5 H5 S1 29.06.23 one each	Gegham 1	2	5	1	A5	6/29/23	8	7/5/23	738	4157	37	218	14	24	73	57	2.97	0.18	0.32	0.77	0.06	0.11	0.34	0.26
1390	Tr2 A5 H5 S1 29.06.23 one each	Kelbadjar	2	5	1	A5	6/29/23	9	7/5/23	458	4964	35	168	22	11	103	33	1.64	0.21	0.11	0.32	0.13	0.07	0.61	0.20
1391	Tr2 A5 H5 S1 29.06.23 one each	Syunik	2	5	1	A5	6/29/23	10	7/5/23	534	5291	44	190	20	11	102	32	1.86	0.19	0.11	0.31	0.10	0.06	0.54	0.17
1392	Tr2 A5 H5 S1 29.06.23 one each	Kelbadjar	2	5	1	A5	6/29/23	11	7/5/23	446	5077	40	173	25	13	107	32	1.63	0.23	0.13	0.30	0.14	0.08	0.62	0.18
1393	Tr2 A5 H5 S1 29.06.23 one each	Gegham 1	2	5	1	A5	6/29/23	12	7/5/23	713	3878	34	209	13	29	74	58	2.81	0.17	0.39	0.78	0.06	0.14	0.36	0.28
1394	Tr2 A5 H5 S1 29.06.23 one each	Gegham 1	2	5	1	A5	6/29/23	13	7/5/23	766	4269	36	222	15	23	74	51	2.98	0.19	0.31	0.69	0.07	0.10	0.34	0.23
1395	Tr2 A5 H5 S1 29.06.23 one each	Tsaghkunyats	2	5	1	A5	6/29/23	14	7/5/23	427	7961	38	96	254	10	156	17	0.61	1.63	0.06	0.11	2.66	0.10	1.63	0.18
1396	Tr2 A5 H5 S1 29.06.23 one each	Gegham 1	2	5	1	A5	6/29/23	16	7/5/23	938	4867	44	246	15	27	73	55	3.35	0.20	0.36	0.75	0.06	0.11	0.30	0.22
1397	Tr2 A5 H5 S1 29.06.23 one each	Gegham 1	2	5	1	A5	6/29/23	17	7/5/23	792	4495	41	233	15	25	79	55	2.95	0.18	0.31	0.70	0.06	0.11	0.34	0.24
1398	Tr2 A5 H5 S1 29.06.23 one each	Gegham 1	2	5	1	A5	6/29/23	18	7/5/23	946	5216	46	260	14	25	79	58	3.28	0.17	0.31	0.73	0.05	0.09	0.30	0.22
1399	Tr2 A5 H5 S1 29.06.23 one each	Syunik	2	5	1	A5	6/29/23	19	7/5/23	590	6234	44	195	26	11	108	35	1.81	0.24	0.11	0.32	0.13	0.06	0.55	0.18
1400	TR.2 A4 H5 S2 02.07.23 one each	Gegham 1	2	5	2	A4	7/2/23	20	7/5/23	700	3870	35	212	14	24	70	57	3.04	0.19	0.34	0.81	0.06	0.11	0.33	0.27
1401	TR.2 A4 H5 S2 02.07.23 one each	Gegham 1	2	5	2	A4	7/2/23	21	7/5/23	715	4035	35	217	13	24	74	57	2.91	0.17	0.32	0.76	0.06	0.11	0.34	0.26
1402	TR.2 A4 H5 S2 02.07.23 one each	Gegham 1	2	5	2	A4	7/2/23	22	7/5/23	691	3823	33	212	13	27	77	55	2.74	0.16	0.36	0.71	0.06	0.13	0.36	0.26
1403	TR.2 A4 H5 S2 02.07.23 one each	Syunik	2	5	2	A4	7/2/23	23	7/5/23	483	5086	35	183	19	12	102	33	1.80	0.18	0.12	0.33	0.10	0.07	0.56	0.18
1404	TR.2 A4 H5 S2 02.07.23 one each	Gegham 1	2	5	2	A4	7/2/23	24	7/5/23	786	4224	36	220	15	26	73	54	3.03	0.20	0.35	0.74	0.07	0.12	0.33	0.25
1405	TR.2 A4 H5 S2 02.07.23 one each	Gegham 1	2	5	2	A4	7/2/23	25	7/5/23	717	4140	38	218	14	26	73	54	2.97	0.18	0.35	0.73	0.06	0.12	0.34	0.25
1406	TR.2 A4 H5 S2 02.07.23 one each	Arteni	2	5	2	A4	7/2/23	26	7/5/23	681	4307	41	144	21	28	81	35	1.78	0.25	0.35	0.43	0.14	0.20	0.56	0.24
1407	TR.2 A4 H5 S2 02.07.23 one each	Gegham 1	2	5	2	A4	7/2/23	27	7/5/23	703	4022	34	216	13	27	74	55	2.90	0.17	0.36	0.74	0.06	0.12	0.34	0.26
1408	TR.2 A4 H5 S2 02.07.23 one each	Gegham 1	2	5	2	A4	7/2/23	28	7/5/23	782	4476	35	232	14	26	76	55	3.03	0.18	0.34	0.72	0.06	0.11	0.33	0.24
1409	TR.2 A4 H5 S2 02.07.23 one each	Gegham 1	2	5	2	A4	7/2/23	29	7/5/23	691	3903	34	209	13	25	73	55	2.85	0.17	0.34	0.75	0.06	0.12	0.35	0.26
1410	TR.2 A4 H5 S2 02.07.23 one each	Gegham 1	2	5	2	A4	7/2/23	30	7/5/23	736	4221	36	222	14	27	77	57	2.87	0.17	0.34	0.73	0.06	0.12	0.35	0.26
1411	TR.2 A4 H5 S2 02.07.23 one each	Kelbadjar	2	5	2	A4	7/2/23	31	7/5/23	524	5525	43	162	22	11	114	39	1.42	0.19	0.10	0.34	0.13	0.07	0.71	0.24
1412	TR.2 A4 H5 S2 02.07.23 one each	Gegham 1	2	5	2	A4	7/2/23	32	7/5/23	688	3963	33	218	14	25	76	58	2.86	0.18	0.32	0.76	0.06	0.11	0.35	0.27
1413	TR.2 A4 H5 S2 02.07.23 one each	Gegham 1	2	5	2	A4	7/2/23	33	7/5/23	670	3820	32	213	11	26	76	57	2.79	0.15	0.34	0.74	0.05	0.12	0.36	0.27
1414	TR.2 A4 H5 S2 02.07.23 one each	Gegham 1	2	5	2	A4	7/2/23	34	7/5/23	776	4389	37	238	15	25	73	54	3.24	0.20	0.34	0.73	0.06	0.10	0.31	0.23
1415	TR.2 A4 H5 S2 02.07.23 one each	Gegham 1	2	5	2	A4	7/2/23	35	7/5/23	741	4093	35	215	13	25	73	55	2.96	0.17	0.34	0.76	0.06	0.11	0.34	0.26
1416	TR.2 A4 H5 S2 02.07.23 one each	Gegham 1	2	5	2	A4	7/2/23	36	7/5/23	839	4726	42	246	13	24	79	58	3.11	0.16	0.30	0.73	0.05	0.10	0.32	0.24
1417	TR.2 A4 H5 S2 02.07.23 one each	Syunik	2	5	2	A4	7/2/23	37	7/5/23	621	4784	40	231	8	11	96	47	2.40	0.09	0.11	0.49	0.04	0.05	0.42	0.20
1418	TR.2 A4 H5 S2 02.07.23 one each	Gegham 1	2	5	2	A4	7/2/23	38	7/5/23	721	3947	34	217	14	26	73	57	2.95	0.18	0.35	0.77	0.06	0.12	0.34	0.26
1419	TR.2 A4 H5 S2 02.07.23 one each	Gegham 1	2	5	2	A4	7/2/23	39	7/5/23	701	3986	34	220	10	26	74	55	2.95	0.14	0.34	0.74	0.05	0.12	0.34	0.25
1420	TR.2 A4 H5 S2 02.07.23 one each	Gegham 1	2	5	2	A4	7/2/23	40	7/5/23	766	4169	40	223	13	26	74	55	2.99	0.17	0.34	0.74	0.06	0.11	0.33	0.25
1421	TR.2 A4 H5 S2 02.07.23 one each	Gegham 1	2	5	2	A4	7/2/23	41	7/5/23	711	4054	35	220	14	27	74	58	2.95	0.18	0.36	0.78	0.06	0.12	0.34	0.26
1422	TR.2 A4 H5 S2 02.07.23 one each	Gegham 1	2	5	2	A4	7/2/23	42	7/5/23	771	4076	35	227	16	27	74	57	3.05	0.21	0.36	0.76	0.07	0.12	0.33	0.25
1423	TR.2 A4 H5 S2 02.07.23 one each	Kelbadjar	2	5	2	A4	7/2/23	43	7/5/23	530	5618	46	168	18	12	114	37	1.48	0.15	0.11	0.33	0.10	0.07	0.68	0.22
1424	TR.2 A4 H5 S2 02.07.23 one each	Gegham 1	2	5	2	A4	7/2/23	44	7/5/23	753	4108	38	225	13	28	79	57	2.84	0.16	0.36	0.72	0.06	0.13	0.35	0.25

1425	TR.2 A4 H5 S2 02.07.23 one each	Gegham 1	2	5	2	A4	7/2/23	45	7/5/23	951	5242	45	257	16	26	74	54	3.45	0.21	0.34	0.72	0.06	0.10	0.29	0.21
1426	TR.2 A4 H5 S2 02.07.23 one each	Arteni	2	5	2	A4	7/2/23	46	7/5/23	642	4243	39	142	26	28	86	32	1.66	0.30	0.33	0.37	0.18	0.20	0.60	0.22
1427	TR.2 A4 H5 S2 02.07.23 one each	Kelbadjar	2	5	2	A4	7/2/23	47	7/5/23	586	5942	45	175	18	11	120	35	1.46	0.15	0.10	0.29	0.10	0.07	0.68	0.20
1428	TR.2 A4 H5 S2 02.07.23 one each	Gegham 1	2	5	2	A4	7/2/23	48	7/5/23	830	4505	42	239	15	25	77	61	3.10	0.19	0.32	0.79	0.06	0.10	0.32	0.25
1429	Tr2 CO TS 01.07.23 one each	Gegham 1	2	0	-	C0	7/1/23	49	7/5/23	676	3723	36	210	16	26	72	54	2.94	0.22	0.36	0.75	0.07	0.12	0.34	0.26
1430	Tr2 CO TS 01.07.23 one each	Kelbadjar	2	0	-	C0	7/1/23	50	7/5/23	638	6586	57	181	22	10	113	36	1.60	0.19	0.08	0.32	0.12	0.05	0.63	0.20
1431	TR.2 B1 H1 S2 03.07.23 one each	Gegham 1	2	1	2	B1	7/3/23	51	7/5/23	724	3861	39	212	13	26	72	54	2.96	0.17	0.36	0.75	0.06	0.12	0.34	0.25
1432	TR.2 B1 H1 S2 03.07.23 one each	Kelbadjar	2	1	2	B1	7/3/23	52	7/5/23	519	5497	40	161	22	9	108	33	1.48	0.20	0.08	0.31	0.13	0.05	0.68	0.21
1433	TR.2 B1 H1 S2 03.07.23 one each	Kelbadjar	2	1	2	B1	7/3/23	53	7/5/23	534	5284	41	161	15	11	112	35	1.43	0.13	0.10	0.31	0.09	0.07	0.70	0.22
1434	TR.2 B1 H1 S2 03.07.23 one each	Gegham 1	2	1	2	B1	7/3/23	55	7/5/23	814	4382	38	233	14	24	75	58	3.10	0.18	0.31	0.77	0.06	0.10	0.32	0.25
1435	TR.2 B1 H1 S2 03.07.23 one each	Kelbadjar	2	1	2	B1	7/3/23	56	7/5/23	519	5340	45	164	16	11	109	36	1.49	0.14	0.10	0.33	0.09	0.06	0.67	0.22
1436	TR.2 B1 H1 S2 03.07.23 one each	Kelbadjar	2	1	2	B1	7/3/23	57	7/5/23	508	5096	38	162	17	13	109	33	1.48	0.15	0.12	0.30	0.10	0.08	0.68	0.21
1437	TR.2 B1 H1 S2 03.07.23 one each	Kelbadjar	2	1	2	B1	7/3/23	58	7/5/23	557	5345	42	162	18	10	119	35	1.36	0.15	0.08	0.29	0.11	0.06	0.74	0.21
1438	TR.2 B1 H1 S2 03.07.23 one each	Gegham 1	2	1	2	B1	7/3/23	60	7/5/23	683	3889	35	219	14	25	74	58	2.94	0.18	0.33	0.78	0.06	0.11	0.34	0.26
1439	TR.2 B1 H1 S2 03.07.23 one each	Kelbadjar	2	1	2	B1	7/3/23	61	7/5/23	508	5052	38	154	16	11	110	35	1.39	0.14	0.10	0.31	0.10	0.07	0.72	0.23
1440	TR.2 B1 H1 S2 03.07.23 one each	Gegham 1	2	1	2	B1	7/3/23	62	7/5/23	882	4731	43	239	18	27	73	57	3.26	0.24	0.36	0.77	0.07	0.11	0.31	0.24
1441	TR.2 B1 H1 S2 03.07.23 one each	Gegham 1	2	1	2	B1	7/3/23	63	7/5/23	734	4384	37	222	13	24	74	55	2.98	0.17	0.32	0.74	0.06	0.11	0.34	0.25
1442	TR.2 B1 H1 S2 03.07.23 one each	Kelbadjar	2	1	2	B1	7/3/23	64	7/5/23	626	5844	45	176	18	9	113	35	1.56	0.15	0.08	0.31	0.10	0.05	0.64	0.20
1443	TR.2 B1 H1 S2 03.07.23 one each	Syunik	2	1	2	B1	7/3/23	65	7/5/23	488	4883	35	177	19	12	103	32	1.72	0.18	0.12	0.31	0.10	0.07	0.58	0.18
1444	TR.2 B1 H1 S2 03.07.23 one each	Gegham 1	2	1	2	B1	7/3/23	66	7/5/23	753	4241	38	231	14	25	76	58	3.02	0.18	0.32	0.76	0.06	0.11	0.33	0.25
1445	TR.2 B1 H1 S2 03.07.23 one each	Kelbadjar	2	1	2	B1	7/3/23	67	7/5/23	569	5793	49	170	18	12	109	39	1.56	0.16	0.11	0.35	0.10	0.07	0.64	0.23
1446	TR.2 B1 H1 S2 03.07.23 one each	Kelbadjar	2	1	2	B1	7/3/23	68	7/5/23	560	5459	45	167	17	12	120	35	1.40	0.14	0.10	0.29	0.10	0.07	0.72	0.21
1447	TR.2 B1 H1 S2 03.07.23 one each	Gegham 1	2	1	2	B1	7/3/23	70	7/5/23	863	4720	44	246	16	24	77	57	3.18	0.20	0.31	0.73	0.06	0.10	0.31	0.23
1448	TR.2 B1 H1 S2 03.07.23 one each	Arteni	2	1	2	B1	7/3/23	71	7/5/23	656	5777	47	145	46	26	97	29	1.49	0.47	0.26	0.30	0.31	0.18	0.67	0.20
1449	TR.2 B1 H1 S2 03.07.23 one each	Gegham 1	2	1	2	B1	7/3/23	72	7/5/23	933	5208	44	258	15	27	77	58	3.34	0.19	0.34	0.75	0.06	0.10	0.30	0.22
1450	TR.2 B1 H1 S2 03.07.23 one each	Gegham 1	2	1	2	B1	7/3/23	73	7/5/23	1183	6294	53	280	16	25	80	61	3.50	0.19	0.31	0.76	0.06	0.09	0.29	0.22
1451	Tr2 A4 H5 S2 01.07.23 one each	Gegham 1	2	5	2	A4	7/1/23	74	7/5/23	701	3984	36	212	11	26	73	57	2.89	0.16	0.35	0.77	0.05	0.12	0.35	0.27
1452	Tr2 A4 H5 S2 01.07.23 one each	Gegham 1	2	5	2	A4	7/1/23	75	7/5/23	703	3976	33	215	14	27	73	58	2.93	0.18	0.36	0.79	0.06	0.12	0.34	0.27
1453	Tr2 A4 H5 S2 01.07.23 one each	Gegham 1	2	5	2	A4	7/1/23	76	7/5/23	715	4159	37	227	13	26	76	53	2.97	0.16	0.34	0.69	0.06	0.11	0.34	0.23
1454	Tr2 A4 H5 S2 01.07.23 one each	Gegham 1	2	5	2	A4	7/1/23	77	7/5/23	732	4108	39	215	15	27	72	54	3.00	0.20	0.37	0.75	0.07	0.12	0.33	0.25
1455	Tr2 A4 H5 S2 01.07.23 one each	Gegham 1	2	5	2	A4	7/1/23	78	7/5/23	678	4057	35	213	14	23	71	55	3.02	0.19	0.32	0.78	0.06	0.11	0.33	0.26
1456	Tr2 A4 H5 S2 01.07.23 one each	Khorapor	2	5	2	A4	7/1/23	79	7/5/23	470	4107	33	226	11	15	82	36	2.75	0.14	0.19	0.44	0.05	0.07	0.36	0.16
1457	Tr2 A4 H5 S2 01.07.23 one each	Gegham 1	2	5	2	A4	7/1/23	80	7/5/23	708	3876	33	210	14	24	69	50	3.06	0.20	0.34	0.72	0.06	0.11	0.33	0.24
1458	Tr2 A4 H5 S2 01.07.23 one each	Gegham 1	2	5	2	A4	7/1/23	81	7/5/23	687	3990	36	213	15	24	71	54	3.02	0.21	0.34	0.76	0.07	0.11	0.33	0.25
1459	Tr2 A4 H5 S2 01.07.23 one each	Gegham 1	2	5	2	A4	7/1/23	82	7/5/23	749	3933	36	224	16	26	79	58	2.83	0.20	0.32	0.73	0.07	0.11	0.35	0.26
1460	Tr2 HOR.3 SP.1 A5 one each	Kelbadjar	2	3	1	A5	-	83	7/5/23	491	5204	35	158	15	11	106	35	1.49	0.14	0.11	0.33	0.09	0.07	0.67	0.22
1461	Tr2 HOR.3 SP.1 A5 one each	Gegham 1	2	3	1	A5	-	84	7/5/23	667	3715	33	210	13	26	71	55	2.97	0.18	0.36	0.78	0.06	0.12	0.34	0.26
1462	Tr2 HOR.3 SP.1 A5 one each	Kelbadjar	2	3	1	A5	-	85	7/5/23	555	5596	44	165	17	11	123	35	1.35	0.13	0.09	0.28	0.10	0.07	0.74	0.21
1463	Tr2 HOR.3 SP.1 A5 one each	Kelbadjar	2	3	1	A5	-	86	7/5/23	480	4916	39	157	17	11	112	35	1.40	0.15	0.09	0.31	0.11	0.07	0.72	0.22
1464	Tr2 HOR.3 SP.1 A5 one each	Gegham 1	2	3	1	A5	-	87	7/5/23	677	4724	39	211	16	27	76	57	2.77	0.20	0.35	0.74	0.07	0.13	0.36	0.27
1465	Tr2 HOR.3 SP.1 A5 one each	Gegham 1	2	3	1	A5	-	88	7/5/23	746	4071	36	222	13	27	76	54	2.91	0.16	0.36	0.71	0.06	0.12	0.34	0.24
1466	Tr2 HOR.3 SP.1 A5 one each	Gegham 1	2	3	1	A5	-	89	7/5/23	671	3928	33	216	14	25	74	58	2.90	0.18	0.33	0.78	0.06	0.11	0.34	0.27
1467	Tr2 HOR.3 SP.1 A5 one each	Gegham 1	2	3	1	A5	-	90	7/5/23	744	4207	35	227	16	26	73	54	3.12	0.21	0.35	0.74	0.07	0.11	0.32	0.24
1468	Tr2 HOR.3 SP.1 A5 one each	Gegham 1	2	3	1	A5	-	91	7/5/23	737	4131	35	221	14	24	75	54	2.93	0.18	0.31	0.71	0.06	0.11	0.34	0.24
1469	Tr2 HOR.3 SP.1 A5 one each	Syunik	2	3	1	A5	-	92	7/5/23	484	5464	38	185	25	12	113	36	1.63	0.22	0.11	0.32	0.13	0.07	0.61	0.19
1470	Tr2 HOR.3 SP.1 A5 one each	Kelbadjar	2	3	1	A5	-	93	7/5/23	597	5907	46	174	17	11	115	37	1.51	0.14	0.10	0.33	0.09	0.07	0.66	0.21
1471	Tr2 HOR.3 SP.1 A5 one each	Syunik	2	3	1	A5	-	94	7/5/23	569	5763	39	197	21	10	109	39	1.80	0.19	0.09	0.35	0.10	0.05	0.56	0.20
1472	Tr2 HOR.3 SP.1 A5 one each	Gegham 1	2	3	1	A5	-	95	7/5/23	812	4568	46	239	15	27	73	57	3.26	0.20	0.36	0.77	0.06	0.11	0.31	0.24
1473	Tr2 A4 H3 S2 30.06.23 one each	Kelbadjar	2	3	2	A4	6/30/23	96	7/5/23	565	5447	43	167	17	11	109	35	1.53	0.15	0.10	0.32	0.10	0.06	0.65	0.21
1474	Tr2 A4 H3 S2 30.06.23 one each	Kelbadjar	2	3	2	A4	6/30/23	97	7/5/23	550	5669	41	165	17	11	116	39	1.43	0.14	0.10	0.33	0.10	0.07	0.70	0.23
1475	Tr2 A4 H3 S2 30.06.23 one each	Gegham 1	2	3	2	A4	6/30/23	98	7/5/23	683	4228	33	213	16	26	75	57	2.83	0.21	0.34	0.75	0.07	0.12	0.35	0.27

1476	Tr2 A4 H3 S2 30.06.23 one each	Syunik	2	3	2	A4	6/30/23	99	7/5/23	463	5022	34	174	25	9	98	33	1.78	0.25	0.09	0.34	0.14	0.05	0.56	0.19
1477	Tr2 A4 H3 S2 30.06.23 one each	Kelbadjar	2	3	2	A4	6/30/23	100	7/5/23	597	5966	46	172	16	10	120	35	1.44	0.13	0.08	0.29	0.09	0.06	0.70	0.20
1478	Tr2 A4 H3 S2 30.06.23 one each	Kelbadjar	2	3	2	A4	6/30/23	101	7/5/23	516	5407	40	164	17	10	108	39	1.51	0.15	0.09	0.36	0.10	0.06	0.66	0.24
1479	Tr2 A4 H3 S2 30.06.23 one each	Gegham 1	2	3	2	A4	6/30/23	102	7/5/23	746	4288	35	226	15	26	78	57	2.89	0.19	0.33	0.72	0.06	0.11	0.35	0.25
1480	Tr2 A4 H3 S2 30.06.23 one each	Arteni	2	3	2	A4	6/30/23	103	7/5/23	697	4944	46	147	32	28	93	33	1.57	0.34	0.30	0.36	0.21	0.19	0.63	0.23
1481	Tr2 A4 H3 S2 30.06.23 one each	Arteni	2	3	2	A4	6/30/23	104	7/5/23	656	4449	45	140	27	28	82	33	1.71	0.32	0.35	0.41	0.19	0.20	0.58	0.24
1482	Tr2 A4 H3 S2 30.06.23 one each	Gegham 1	2	3	2	A4	6/30/23	105	7/5/23	757	4256	40	222	15	27	73	58	3.02	0.20	0.37	0.79	0.07	0.12	0.33	0.26
1483	Tr2 A4 H3 S2 30.06.23 one each	Gegham 1	2	3	2	A4	6/30/23	106	7/5/23	842	4645	42	233	13	26	77	57	3.01	0.16	0.33	0.73	0.05	0.11	0.33	0.24
1484	Tr2 A4 H3 S2 30.06.23 one each	Syunik	2	3	2	A4	6/30/23	107	7/5/23	720	7463	59	231	26	6	108	36	2.13	0.24	0.05	0.33	0.11	0.03	0.47	0.16
1485	Tr2 A4 H3 S2 30.06.23 one each	Gegham 1	2	3	2	A4	6/30/23	108	7/5/23	971	5253	49	265	14	26	77	54	3.42	0.17	0.33	0.70	0.05	0.10	0.29	0.20
1486	Tr2 A4 H3 S2 30.06.23 one each	Kelbadjar	2	3	2	A4	6/30/23	109	7/5/23	506	5223	42	164	18	10	111	39	1.48	0.16	0.09	0.35	0.11	0.06	0.68	0.24
1487	Tr2 A4 H3 S2 30.06.23 one each	Gegham 1	2	3	2	A4	6/30/23	111	7/5/23	784	4404	35	230	14	24	75	55	3.05	0.18	0.31	0.73	0.06	0.10	0.33	0.24
1488	Tr2 A4 H2 S2 30.06.23 one each	Gegham 1	2	2	2	A4	6/30/23	112	7/5/23	686	3925	32	208	13	27	72	55	2.91	0.17	0.38	0.77	0.06	0.13	0.34	0.27
1489	Tr2 A4 H2 S2 30.06.23 one each	Gegham 1	2	2	2	A4	6/30/23	113	7/5/23	739	4104	42	227	20	25	75	55	3.01	0.26	0.33	0.73	0.09	0.11	0.33	0.24
1490	Tr2 A4 H2 S2 30.06.23 one each	Gegham 1	2	2	2	A4	6/30/23	114	7/5/23	839	4639	44	244	16	22	73	54	3.37	0.21	0.30	0.74	0.06	0.09	0.30	0.22
1491	Tr2 A4 H2 S2 30.06.23 one each	Chikiani?	2	2	2	A4	6/30/23	115	7/5/23	736	4213	135	186	16	27	73	55	2.53	0.21	0.36	0.75	0.08	0.14	0.40	0.30
1492	Tr2 A4 H2 S2 30.06.23 one each	Gegham 1	2	2	2	A4	6/30/23	116	7/5/23	686	4313	36	218	15	25	74	57	2.93	0.19	0.33	0.76	0.07	0.11	0.34	0.26
1493	Tr2 A4 H2 S2 30.06.23 one each	Gegham 1	2	2	2	A4	6/30/23	117	7/5/23	636	3597	35	203	18	27	73	55	2.80	0.24	0.38	0.76	0.09	0.14	0.36	0.27
1494	Tr2 A4 H2 S2 30.06.23 one each	Gegham 1	2	2	2	A4	6/30/23	118	7/5/23	802	4487	38	235	16	27	79	58	2.97	0.20	0.35	0.73	0.07	0.12	0.34	0.25
1495	Tr2 A4 H2 S2 30.06.23 one each	Kelbadjar	2	2	2	A4	6/30/23	119	7/5/23	529	5395	40	166	17	11	111	36	1.50	0.15	0.10	0.32	0.10	0.07	0.67	0.22
1496	Tr2 A4 H2 S2 30.06.23 one each	Gegham 1	2	2	2	A4	6/30/23	120	7/5/23	747	4219	36	225	14	25	76	58	2.94	0.18	0.32	0.76	0.06	0.11	0.34	0.26
1497	Tr2 A4 H2 S2 30.06.23 one each	Gegham 1	2	2	2	A4	6/30/23	121	7/5/23	824	4501	40	235	14	29	77	58	3.05	0.17	0.38	0.75	0.06	0.12	0.33	0.25
1498	Tr2 A4 H2 S2 30.06.23 one each	Kelbadjar	2	2	2	A4	6/30/23	122	7/5/23	564	5739	42	171	19	14	113	37	1.51	0.16	0.13	0.33	0.11	0.08	0.66	0.22
1499	Tr2 A4 H2 S2 30.06.23 one each	Gegham 1	2	2	2	A4	6/30/23	123	7/5/23	743	4114	35	223	13	26	76	57	2.92	0.16	0.34	0.74	0.06	0.11	0.34	0.25
1500	Tr2 A4 H2 S2 30.06.23 one each	Gegham 1	2	2	2	A4	6/30/23	124	7/5/23	799	4228	38	230	14	25	78	55	2.94	0.17	0.32	0.71	0.06	0.11	0.34	0.24
1501	Tr2 A4 H2 S2 30.06.23 one each	Gegham 1	2	2	2	A4	6/30/23	125	7/5/23	774	4163	35	224	14	27	78	59	2.86	0.17	0.35	0.76	0.06	0.12	0.35	0.27
1502	Tr2 A4 H2 S2 30.06.23 one each	Gegham 1	2	2	2	A4	6/30/23	126	7/5/23	723	3634	35	231	15	27	78	58	2.95	0.19	0.34	0.74	0.06	0.12	0.34	0.25
1503	Tr2 A4 H2 S2 30.06.23 one each	Gegham 1	2	2	2	A4	6/30/23	127	7/5/23	777	4637	41	230	15	26	73	57	3.12	0.20	0.35	0.77	0.06	0.11	0.32	0.25
1504	Tr2 A4 H2 S2 30.06.23 one each	Gegham 1	2	2	2	A4	6/30/23	128	7/5/23	823	4131	42	237	15	27	74	58	3.19	0.19	0.36	0.78	0.06	0.11	0.31	0.24
1505	Tr2 A4 H2 S2 30.06.23 one each	Gegham 1	2	2	2	A4	6/30/23	130	7/5/23	814	4464	39	233	15	27	77	55	3.01	0.19	0.36	0.71	0.06	0.12	0.33	0.24
1506	Tr2 A4 H2 S2 30.06.23 one each	Gegham 1	2	2	2	A4	6/30/23	131	7/5/23	870	4715	39	246	15	25	80	62	3.07	0.18	0.31	0.78	0.06	0.10	0.33	0.25
1507	Tr2 A4 H2 S2 30.06.23 one each	Gegham 1	2	2	2	A4	6/30/23	132	7/5/23	888	4859	46	251	14	28	78	55	3.21	0.17	0.36	0.71	0.05	0.11	0.31	0.22
1508	Tr2 A4 H2 S2 30.06.23 one each	Gegham 1	2	2	2	A4	6/30/23	133	7/5/23	774	4233	38	220	16	24	75	54	2.92	0.21	0.31	0.71	0.07	0.11	0.34	0.25
1509	Tr2 A4 H2 S2 30.06.23 one each	Gegham 1	2	2	2	A4	6/30/23	134	7/5/23	737	4212	40	229	15	27	79	57	2.89	0.18	0.34	0.72	0.06	0.12	0.35	0.25
1510	TR.2 Un.5 H1 S2 collapse 30.06.23 one each	Gegham 1	2	1	2	collapse	6/30/23	136	7/5/23	695	3865	34	209	13	23	73	55	2.88	0.17	0.31	0.76	0.06	0.11	0.35	0.26
1511	TR.2 Un.5 H1 S2 collapse 30.06.23 one each	Syunik	2	1	2	collapse	6/30/23	137	7/5/23	525	5605	44	183	25	11	106	37	1.73	0.23	0.10	0.35	0.13	0.06	0.58	0.20
1512	TR.2 Un.5 H1 S2 collapse 30.06.23 one each	Gegham 1	2	1	2	collapse	6/30/23	138	7/5/23	652	4058	33	199	16	24	73	51	2.71	0.21	0.32	0.70	0.08	0.12	0.37	0.26
1513	TR.2 Un.5 H1 S2 collapse 30.06.23 one each	Gegham 1	2	1	2	collapse	6/30/23	139	7/5/23	605	3645	39	205	15	24	70	53	2.95	0.21	0.34	0.75	0.07	0.12	0.34	0.26
1514	TR.2 Un.5 H1 S2 collapse 30.06.23 one each	Syunik	2	1	2	collapse	6/30/23	140	7/5/23	483	4964	35	173	25	11	103	32	1.69	0.24	0.10	0.31	0.14	0.06	0.59	0.18
1515	TR.2 Un.5 H1 S2 collapse 30.06.23 one each	Syunik	2	1	2	collapse	6/30/23	141	7/5/23	459	5185	37	175	23	11	104	35	1.69	0.22	0.10	0.33	0.13	0.06	0.59	0.20
1516	TR.2 Un.5 H1 S2 collapse 30.06.23 one each	Kelbadjar	2	1	2	collapse	6/30/23	142	7/5/23	491	5275	36	163	18	11	111	37	1.46	0.16	0.09	0.34	0.11	0.06	0.68	0.23
1517	TR.2 Un.5 H1 S2 collapse 30.06.23 one each	Kelbadjar	2	1	2	collapse	6/30/23	143	7/5/23	475	5127	38	157	16	10	116	33	1.35	0.13	0.08	0.29	0.10	0.06	0.74	0.21
1518	TR.2 Un.5 H1 S2 collapse 30.06.23 one each	Kelbadjar	2	1	2	collapse	6/30/23	144	7/5/23	505	5153	39	159	17	12	108	36	1.46	0.15	0.11	0.33	0.10	0.08	0.68	0.23
1519	TR.2 Un.5 H1 S2 collapse 30.06.23 one each	Gegham 1	2	1	2	collapse	6/30/23	145	7/5/23	695	3728	31	205	13	25	71	55	2.91	0.18	0.35	0.78	0.06	0.12	0.34	0.27
1520	TR.2 Un.5 H1 S2 collapse 30.06.23 one each	Gegham 1	2	1	2	collapse	6/30/23	146	7/5/23	686	3900	36	212	13	26	76	54	2.78	0.16	0.34	0.71	0.06	0.12	0.36	0.25
1521	TR.2 Un.5 H1 S2 collapse 30.06.23 one each	Kelbadjar	2	1	2	collapse	6/30/23	147	7/5/23	473	5017	41	161	17	11	108	35	1.49	0.15	0.10	0.32	0.10	0.07	0.67	0.22
1522	TR.2 Un.5 H1 S2 collapse 30.06.23 one each	Kelbadjar	2	1	2	collapse	6/30/23	148	7/5/23	596	5891	44	171	18	12	114	40	1.50	0.15	0.11	0.35	0.10	0.07	0.67	0.23
1523	TR.2 Un.5 H1 S2 collapse 30.06.23 one each	Gegham 1	2	1	2	collapse	6/30/23	149	7/5/23	773	4222	35	224	13	26	77	59	2.90	0.16	0.33	0.77	0.06	0.11	0.35	0.27
1524	TR.2 Un.5 H1 S2 collapse 30.06.23 one each	Gegham 1	2	1	2	collapse	6/30/23	150	7/5/23	687	3964	33	213	13	25	74	53	2.86	0.17	0.33	0.71	0.06	0.12	0.35	0.25
1525	TR.2 Un.5 H1 S2 collapse 30.06.23 one each	Gegham 1	2	1	2	collapse	6/30/23	151	7/5/23	643	3952	35	213	13	22	74	54	2.86	0.17	0.29	0.72	0.06	0.10	0.35	0.25
1526	TR.2 Un.5 H1 S2 collapse 30.06.23 one each	Kelbadjar	2	1	2	collapse	6/30/23	152	7/5/23	516	5521	40	163	17	10	109	39	1.49	0.15	0.09	0.35	0.10	0.06	0.67	0.24

1527	TR.2 Un.5 H1 S2 collapse 30.06.23 one each	Gegham 1	2	1	2	collapse	6/30/23	153	7/5/23	751	4124	35	216	13	27	74	55	2.90	0.17	0.36	0.74	0.06	0.12	0.34	0.26
1528	TR.2 Un.5 H1 S2 collapse 30.06.23 one each	Kelbadjar	2	1	2	collapse	6/30/23	154	7/5/23	552	5669	43	171	18	11	118	37	1.45	0.15	0.10	0.32	0.10	0.07	0.69	0.22
1529	TR.2 Un.5 H1 S2 collapse 30.06.23 one each	Gegham 1	2	1	2	collapse	6/30/23	155	7/5/23	806	4400	37	227	15	27	75	54	3.01	0.19	0.35	0.71	0.06	0.12	0.33	0.24
1530	TR.2 Un.5 H1 S2 collapse 30.06.23 one each	Gutansar	2	1	2	collapse	6/30/23	156	7/5/23	638	8396	43	146	128	25	175	37	0.84	0.73	0.14	0.21	0.87	0.17	1.20	0.26
1531	TR.2 Un.5 H1 S2 collapse 30.06.23 one each	Kelbadjar	2	1	2	collapse	6/30/23	157	7/5/23	533	5456	40	164	16	11	108	35	1.51	0.14	0.11	0.32	0.09	0.07	0.66	0.21
1532	TR.2 Un.5 H1 S2 collapse 30.06.23 one each	Kelbadjar	2	1	2	collapse	6/30/23	158	7/5/23	504	5390	35	174	25	12	108	35	1.61	0.23	0.11	0.32	0.14	0.07	0.62	0.20
1533	TR.2 Un.5 H1 S2 collapse 30.06.23 one each	Gegham 1	2	1	2	collapse	6/30/23	159	7/5/23	638	3722	29	206	13	25	70	53	2.96	0.18	0.35	0.75	0.06	0.12	0.34	0.25
1534	TR.2 Un.5 H1 S2 collapse 30.06.23 one each	Arteni	2	1	2	collapse	6/30/23	160	7/5/23	560	4847	41	119	44	25	93	25	1.27	0.47	0.26	0.27	0.37	0.21	0.79	0.21
1535	TR.2 Un.5 H1 S2 collapse 30.06.23 one each	Gegham 1	2	1	2	collapse	6/30/23	161	7/5/23	685	3933	36	214	13	26	75	55	2.84	0.17	0.34	0.73	0.06	0.12	0.35	0.26
1536	TR.2 Un.5 H1 S2 collapse 30.06.23 one each	Arteni	2	1	2	collapse	6/30/23	162	7/5/23	606	4219	34	129	25	27	79	32	1.62	0.31	0.35	0.40	0.19	0.21	0.62	0.25
1537	TR.2 Un.5 H1 S2 collapse 30.06.23 one each	Gegham 1	2	1	2	collapse	6/30/23	163	7/5/23	723	4146	35	217	15	24	73	55	2.99	0.20	0.33	0.76	0.07	0.11	0.33	0.25
1538	TR.2 Un.5 H1 S2 collapse 30.06.23 one each	Syunik	2	1	2	collapse	6/30/23	164	7/5/23	427	4735	35	176	20	11	98	36	1.80	0.20	0.11	0.37	0.11	0.06	0.56	0.20
1539	TR.2 Un.5 H1 S2 collapse 30.06.23 one each	Gegham 1	2	1	2	collapse	6/30/23	165	7/5/23	670	3926	32	211	11	27	77	54	2.73	0.15	0.34	0.70	0.05	0.13	0.37	0.26
1540	TR.2 Un.5 H1 S2 collapse 30.06.23 one each	Kelbadjar	2	1	2	collapse	6/30/23	166	7/5/23	485	5153	41	156	19	11	111	35	1.40	0.17	0.09	0.31	0.12	0.07	0.71	0.22
1541	TR.2 Un.5 H1 S2 collapse 30.06.23 one each	Gegham 1	2	1	2	collapse	6/30/23	167	7/5/23	753	4268	37	229	15	27	72	53	3.19	0.20	0.37	0.73	0.06	0.12	0.31	0.23
1542	TR.2 Un.5 H1 S2 collapse 30.06.23 one each	Gegham 1	2	1	2	collapse	6/30/23	168	7/5/23	718	3884	33	213	14	26	74	54	2.86	0.18	0.34	0.72	0.06	0.12	0.35	0.25
1543	TR.2 Un.5 H1 S2 collapse 30.06.23 one each	Gegham 1	2	1	2	collapse	6/30/23	169	7/5/23	822	4418	40	233	14	25	73	55	3.20	0.19	0.34	0.76	0.06	0.11	0.31	0.24
1544	TR.2 Un.5 H1 S2 collapse 30.06.23 one each	Gegham 1	2	1	2	collapse	6/30/23	170	7/5/23	670	3883	32	212	13	24	73	54	2.89	0.17	0.32	0.73	0.06	0.11	0.35	0.25
1545	TR.2 Un.5 H1 S2 collapse 30.06.23 one each	Kelbadjar	2	1	2	collapse	6/30/23	171	7/5/23	540	5451	40	164	17	11	129	39	1.27	0.13	0.08	0.30	0.10	0.06	0.79	0.24
1546	TR.2 Un.5 H1 S2 collapse 30.06.23 one each	Kelbadjar	2	1	2	collapse	6/30/23	172	7/5/23	475	4898	35	153	17	12	110	35	1.38	0.15	0.11	0.31	0.11	0.08	0.72	0.23
1547	TR.2 Un.5 H1 S2 collapse 30.06.23 one each	Kelbadjar	2	1	2	collapse	6/30/23	173	7/5/23	496	4866	37	152	17	11	105	35	1.45	0.16	0.10	0.33	0.11	0.07	0.69	0.23
1548	TR.2 Un.5 H1 S2 collapse 30.06.23 one each	Kelbadjar	2	1	2	collapse	6/30/23	174	7/5/23	471	5102	41	157	19	10	107	33	1.47	0.17	0.09	0.31	0.12	0.06	0.68	0.21
1549	TR.2 Un.5 H1 S2 collapse 30.06.23 one each	Gegham 1	2	1	2	collapse	6/30/23	175	7/5/23	763	4326	35	222	14	25	78	57	2.84	0.17	0.32	0.72	0.06	0.11	0.35	0.26
1550	TR.2 Un.5 H1 S2 collapse 30.06.23 one each	Kelbadjar	2	1	2	collapse	6/30/23	176	7/5/23	504	5375	43	164	18	11	112	35	1.46	0.16	0.09	0.31	0.11	0.06	0.69	0.21
1551	TR.2 Un.5 H1 S2 collapse 30.06.23 one each	Kelbadjar	2	1	2	collapse	6/30/23	177	7/5/23	584	5656	45	178	20	10	115	33	1.55	0.17	0.08	0.29	0.11	0.05	0.65	0.19
1552	TR.2 Un.5 H1 S2 collapse 30.06.23 one each	Gegham 1	2	1	2	collapse	6/30/23	178	7/5/23	717	4234	35	220	13	25	75	53	2.92	0.17	0.33	0.70	0.06	0.11	0.34	0.24
1553	TR.2 Un.5 H1 S2 collapse 30.06.23 one each	Gegham 1	2	1	2	collapse	6/30/23	179	7/5/23	638	3620	32	198	13	25	72	50	2.77	0.17	0.34	0.69	0.06	0.12	0.36	0.25
1554	TR.2 Un.5 H1 S2 collapse 30.06.23 one each	Gegham 1	2	1	2	collapse	6/30/23	180	7/5/23	711	3933	35	216	14	26	74	54	2.90	0.18	0.34	0.72	0.06	0.12	0.34	0.25
1555	TR.2 Un.5 H1 S2 collapse 30.06.23 one each	Kelbadjar	2	1	2	collapse	6/30/23	181	7/5/23	503	4823	40	154	15	10	104	32	1.48	0.14	0.09	0.31	0.09	0.06	0.67	0.21
1556	TR.2 Un.5 H1 S2 collapse 30.06.23 one each	Kelbadjar	2	1	2	collapse	6/30/23	182	7/5/23	500	5345	39	159	18	10	109	33	1.45	0.16	0.09	0.30	0.11	0.06	0.69	0.21
1557	TR.2 Un.5 H1 S2 collapse 30.06.23 one each	Gegham 1	2	1	2	collapse	6/30/23	183	7/5/23	693	3925	32	218	14	24	71	51	3.08	0.19	0.34	0.72	0.06	0.11	0.32	0.23
1558	TR.2 Un.5 H1 S2 collapse 30.06.23 one each	Kelbadjar	2	1	2	collapse	6/30/23	184	7/5/23	555	5689	49	173	22	12	112	36	1.54	0.19	0.11	0.32	0.12	0.07	0.65	0.21
1559	TR.2 Un.5 H1 S2 collapse 30.06.23 one each	Gegham 1	2	1	2	collapse	6/30/23	185	7/5/23	720	4017	37	225	13	27	74	55	3.02	0.17	0.37	0.74	0.06	0.12	0.33	0.25
1560	TR.2 Un.5 H1 S2 collapse 30.06.23 one each	Kelbadjar	2	1	2	collapse	6/30/23	186	7/5/23	556	5563	45	165	22	11	108	35	1.53	0.20	0.11	0.32	0.13	0.07	0.66	0.21
1561	TR.2 Un.5 H1 S2 collapse 30.06.23 one each	Gegham 1	2	1	2	collapse	6/30/23	187	7/5/23	758	4407	38	228	21	27	77	63	2.95	0.27	0.34	0.82	0.09	0.12	0.34	0.28
1562	TR.2 Un.5 H1 S2 collapse 30.06.23 one each	Kelbadjar	2	1	2	collapse	6/30/23	188	7/5/23	560	5736	42	171	17	11	112	35	1.53	0.15	0.09	0.31	0.10	0.06	0.66	0.20
1563	TR.2 Un.5 H1 S2 collapse 30.06.23 one each	Gegham 1	2	1	2	collapse	6/30/23	189	7/5/23	685	4234	35	214	15	27	76	57	2.80	0.19	0.36	0.74	0.07	0.13	0.36	0.26
1564	TR.2 Un.5 H1 S2 collapse 30.06.23 one each	Gegham 1	2	1	2	collapse	6/30/23	190	7/5/23	880	4754	45	245	15	29	76	54	3.21	0.19	0.38	0.71	0.06	0.12	0.31	0.22
1565	TR.2 Un.5 H1 S2 collapse 30.06.23 one each	Gegham 1	2	1	2	collapse	6/30/23	191	7/5/23	732	3755	33	206	13	25	71	57	2.92	0.18	0.35	0.80	0.06	0.12	0.34	0.27
1566	TR.2 Un.5 H1 S2 collapse 30.06.23 one each	Gegham 1	2	1	2	collapse	6/30/23	192	7/5/23	791	4209	37	229	13	28	78	59	2.92	0.16	0.36	0.76	0.05	0.12	0.34	0.26
1567	TR.2 Un.5 H1 S2 collapse 30.06.23 one each	Kelbadjar	2	1	2	collapse	6/30/23	193	7/5/23	533	5460	39	169	18	11	114	35	1.48	0.15	0.10	0.30	0.10	0.07	0.67	0.20
1568	TR.2 Un.5 H1 S2 collapse 30.06.23 one each	Gegham 1	2	1	2	collapse	6/30/23	194	7/5/23	840	4654	39	234	14	24	73	54	3.19	0.18	0.32	0.73	0.06	0.10	0.31	0.23
1569	TR.2 Un.5 H1 S2 collapse 30.06.23 one each	Gegham 1	2	1	2	collapse	6/30/23	195	7/5/23	746	4411	37	231	17	28	74	54	3.10	0.22	0.38	0.72	0.07	0.12	0.32	0.23
1570	TR.2 Un.5 H1 S2 collapse 30.06.23 one each	Gegham 1	2	1	2	collapse	6/30/23	196	7/5/23	726	4188	39	221	16	28	73	53	3.00	0.21	0.39	0.71	0.07	0.13	0.33	0.24
1571	TR.2 Un.5 H1 S2 collapse 30.06.23 one each	Gegham 1	2	1	2	collapse	6/30/23	197	7/5/23	670	3927	33	209	14	23	74	53	2.81	0.18	0.31	0.71	0.06	0.11	0.36	0.25
1572	TR.2 Un.5 H1 S2 collapse 30.06.23 one each	Kelbadjar	2	1	2	collapse	6/30/23	200	7/5/23	556	5557	41	169	17	11	120	35	1.41	0.14	0.10	0.29	0.10	0.07	0.71	0.20
1573	TR.2 Un.5 H1 S2 collapse 30.06.23 one each	Gegham 1	2	1	2	collapse	6/30/23	201	7/5/23	720	4333	40	229	19	25	73	55	3.15	0.26	0.34	0.76	0.08	0.11	0.32	0.24
1574	TR.2 Un.5 H1 S2 collapse 30.06.23 one each	Kelbadjar	2	1	2	collapse	6/30/23	202	7/5/23	530	5510	44	167	21	13	118	39	1.42	0.17	0.11	0.33	0.12	0.08	0.70	0.23
1575	TR.2 Un.5 H1 S2 collapse 30.06.23 one each	Gegham 1	2	1	2	collapse	6/30/23	203	7/5/23	763	4277	38	224	13	25	74	55	3.01	0.17	0.33	0.74	0.06	0.11	0.33	0.25
1576	TR.2 Un.5 H1 S2 collapse 30.06.23 one each	Gegham 1	2	1	2	collapse	6/30/23	204	7/5/23	716	4016	37	233	17	27	75	55	3.08	0.22	0.35	0.73	0.07	0.11	0.32	0.24
1577	TR.2 Un.5 H1 S2 collapse 30.06.23 one each	Kelbadjar	2	1	2	collapse	6/30/23	205	7/5/23	601	6280	49	180	18	11	116	37	1.55	0.15	0.09	0.32	0.10	0.06	0.64	0.21

1578	TR.2 Un.5 H1 S2 collapse 30.06.23 one each	Gegham 1	2	1	2	collapse	6/30/23	206	7/5/23	868	4947	47	245	16	25	75	55	3.25	0.21	0.33	0.73	0.06	0.10	0.31	0.23
1579	TR2 UN A1-A4 H0-H1Sp1 collapse one each	Kelbadjar	2	0 - H1Sp1	collapse	A1-A4	-	207	7/5/23	501	5331	41	164	16	9	108	36	1.51	0.14	0.08	0.33	0.09	0.05	0.66	0.22
1580	TR2 UN A1-A4 H0-H1Sp1 collapse one each	Kelbadjar	2	0 - H1Sp1	collapse	A1-A4	-	208	7/5/23	529	5240	39	163	17	11	110	36	1.47	0.15	0.10	0.33	0.10	0.06	0.68	0.22
1581	TR2 UN A1-A4 H0-H1Sp1 collapse one each	Kelbadjar	2	0 - H1Sp1	collapse	A1-A4	-	209	7/5/23	483	4880	40	156	18	11	102	33	1.53	0.17	0.10	0.33	0.11	0.07	0.65	0.21
1582	TR2 UN A1-A4 H0-H1Sp1 collapse one each	Gegham 1	2	0 - H1Sp1	collapse	A1-A4	-	210	7/5/23	697	3935	35	213	13	26	73	53	2.94	0.17	0.35	0.72	0.06	0.12	0.34	0.25
1583	TR2 UN A1-A4 H0-H1Sp1 collapse one each	Kelbadjar	2	0 - H1Sp1	collapse	A1-A4	-	211	7/5/23	499	5091	39	158	17	11	109	35	1.44	0.15	0.10	0.32	0.10	0.07	0.69	0.22
1584	TR2 UN A1-A4 H0-H1Sp1 collapse one each	Gegham 1	2	0 - H1Sp1	collapse	A1-A4	-	212	7/5/23	693	3804	31	209	13	24	73	54	2.88	0.17	0.33	0.74	0.06	0.11	0.35	0.26
1585	TR2 UN A1-A4 H0-H1Sp1 collapse one each	Gegham 1	2	0 - H1Sp1	collapse	A1-A4	-	213	7/5/23	710	4021	33	216	13	26	73	54	2.98	0.17	0.35	0.74	0.06	0.12	0.34	0.25
1586	TR2 UN A1-A4 H0-H1Sp1 collapse one each	Gegham 1	2	0 - H1Sp1	collapse	A1-A4	-	214	7/5/23	720	4021	35	219	13	26	70	53	3.14	0.18	0.37	0.75	0.06	0.12	0.32	0.24
1587	TR2 UN A1-A4 H0-H1Sp1 collapse one each	Kelbadjar	2	0 - H1Sp1	collapse	A1-A4	-	215	7/5/23	594	6006	46	178	18	12	116	39	1.53	0.15	0.11	0.33	0.10	0.07	0.65	0.22
1588	TR2 UN A1-A4 H0-H1Sp1 collapse one each	Gegham 1	2	0 - H1Sp1	collapse	A1-A4	-	216	7/5/23	748	4482	36	217	16	24	79	57	2.74	0.20	0.30	0.72	0.07	0.11	0.36	0.26
1589	TR2 UN A1-A4 H0-H1Sp1 collapse one each	Kelbadjar	2	0 - H1Sp1	collapse	A1-A4	-	217	7/5/23	521	5500	42	166	18	8	111	36	1.50	0.16	0.07	0.32	0.11	0.05	0.67	0.22
1590	TR2 UN A1-A4 H0-H1Sp1 collapse one each	Syunik	2	0 - H1Sp1	collapse	A1-A4	-	218	7/5/23	493	5437	39	183	25	11	109	36	1.67	0.22	0.10	0.33	0.13	0.06	0.60	0.20
1591	TR2 UN A1-A4 H0-H1Sp1 collapse one each	Kelbadjar	2	0 - H1Sp1	collapse	A1-A4	-	219	7/5/23	518	5538	43	166	17	11	110	35	1.51	0.15	0.10	0.31	0.10	0.06	0.66	0.21
1592	TR2 UN A1-A4 H0-H1Sp1 collapse one each	Gegham 1	2	0 - H1Sp1	collapse	A1-A4	-	220	7/5/23	685	3851	33	212	14	27	72	57	2.96	0.19	0.37	0.79	0.06	0.13	0.34	0.27
1593	TR2 UN A1-A4 H0-H1Sp1 collapse one each	Syunik	2	0 - H1Sp1	collapse	A1-A4	-	221	7/5/23	508	5384	39	190	20	11	107	36	1.78	0.18	0.11	0.34	0.10	0.06	0.56	0.19
1594	TR2 UN A1-A4 H0-H1Sp1 collapse one each	Gegham 1	2	0 - H1Sp1	collapse	A1-A4	-	222	7/5/23	738	4134	36	221	14	26	76	53	2.89	0.18	0.34	0.69	0.06	0.12	0.35	0.24
1595	TR2 UN A1-A4 H0-H1Sp1 collapse one each	Arteni	2	0 - H1Sp1	collapse	A1-A4	-	223	7/5/23	542	4236	32	119	38	25	87	26	1.37	0.43	0.28	0.30	0.32	0.21	0.73	0.22
1596	TR2 UN A1-A4 H0-H1Sp1 collapse one each	Gegham 1	2	0 - H1Sp1	collapse	A1-A4	-	224	7/5/23	657	3633	34	201	11	24	71	51	2.85	0.16	0.34	0.72	0.06	0.12	0.35	0.25
1597	TR2 UN A1-A4 H0-H1Sp1 collapse one each	Gegham 1	2	0 - H1Sp1	collapse	A1-A4	-	225	7/5/23	784	4327	35	221	13	27	73	53	3.00	0.17	0.37	0.71	0.06	0.12	0.33	0.24
1598	TR2 UN A1-A4 H0-H1Sp1 collapse one each	Kelbadjar	2	0 - H1Sp1	collapse	A1-A4	-	226	7/5/23	514	5111	41	158	17	11	104	35	1.52	0.16	0.10	0.33	0.10	0.07	0.66	0.22
1599	TR2 UN A1-A4 H0-H1Sp1 collapse one each	Kelbadjar	2	0 - H1Sp1	collapse	A1-A4	-	227	7/5/23	516	5126	38	159	16	11	106	36	1.50	0.15	0.10	0.34	0.10	0.07	0.67	0.23
1600	TR2 UN A1-A4 H0-H1Sp1 collapse one each	Gegham 1	2	0 - H1Sp1	collapse	A1-A4	-	228	7/5/23	707	4063	32	214	14	27	74	54	2.88	0.18	0.37	0.72	0.06	0.13	0.35	0.25
1601	TR2 UN A1-A4 H0-H1Sp1 collapse one each	Arteni	2	0 - H1Sp1	collapse	A1-A4	-	229	7/5/23	637	4197	42	143	21	27	82	35	1.75	0.25	0.33	0.42	0.14	0.19	0.57	0.24
1602	TR2 UN A1-A4 H0-H1Sp1 collapse one each	Gegham 1	2	0 - H1Sp1	collapse	A1-A4	-	230	7/5/23	748	4632	39	232	17	27	76	55	3.03	0.22	0.35	0.72	0.07	0.11	0.33	0.24
1603	TR2 UN A1-A4 H0-H1Sp1 collapse one each	Gegham 1	2	0 - H1Sp1	collapse	A1-A4	-	231	7/5/23	716	4076	34	213	13	24	70	54	3.06	0.18	0.34	0.77	0.06	0.11	0.33	0.25
1604	TR2 UN A1-A4 H0-H1Sp1 collapse one each	Kelbadjar	2	0 - H1Sp1	collapse	A1-A4	-	232	7/5/23	518	5240	42	167	18	11	108	33	1.56	0.16	0.11	0.31	0.10	0.07	0.64	0.20
1605	TR2 UN A1-A4 H0-H1Sp1 collapse one each	Gegham 1	2	0 - H1Sp1	collapse	A1-A4	-	233	7/5/23	738	4137	34	222	13	27	75	54	2.94	0.17	0.35	0.71	0.06	0.12	0.34	0.24
1606	TR2 UN A1-A4 H0-H1Sp1 collapse one each	Arteni	2	0 - H1Sp1	collapse	A1-A4	-	234	7/5/23	633	3944	40	133	20	30	82	39	1.63	0.24	0.37	0.47	0.15	0.23	0.61	0.29
1607	TR2 UN A1-A4 H0-H1Sp1 collapse one each	Kelbadjar	2	0 - H1Sp1	collapse	A1-A4	-	235	7/5/23	510	5397	40	164	17	10	111	35	1.48	0.15	0.09	0.31	0.10	0.06	0.68	0.21
1608	TR2 UN A1-A4 H0-H1Sp1 collapse one each	Gegham 1	2	0 - H1Sp1	collapse	A1-A4	-	236	7/5/23	768	4401	39	228	15	27	77	59	2.95	0.19	0.34	0.77	0.06	0.12	0.34	0.26
1609	TR2 UN A1-A4 H0-H1Sp1 collapse one each	Gegham 1	2	0 - H1Sp1	collapse	A1-A4	-	237	7/5/23	824	4564	39	235	15	24	72	53	3.29	0.20	0.33	0.73	0.06	0.10	0.30	0.22
1610	TR2 UN A1-A4 H0-H1Sp1 collapse one each	Kelbadjar	2	0 - H1Sp1	collapse	A1-A4	-	238	7/5/23	579	5904	46	173	16	11	110	37	1.57	0.14	0.10	0.34	0.09	0.07	0.64	0.22
1611	TR2 UN A1-A4 H0-H1Sp1 collapse one each	Gegham 1	2	0 - H1Sp1	collapse	A1-A4	-	239	7/5/23	783	4487	40	233	15	24	73	55	3.18	0.20	0.32	0.75	0.06	0.10	0.31	0.24
1612	TR2 UN A1-A4 H0-H1Sp1 collapse one each	Gegham 1	2	0 - H1Sp1	collapse	A1-A4	-	240	7/5/23	726	4190	37	218	18	25	74	58	2.93	0.24	0.33	0.78	0.08	0.11	0.34	0.27
1613	TR2 UN A1-A4 H0-H1Sp1 collapse one each	Gegham 1	2	0 - H1Sp1	collapse	A1-A4	-	241	7/5/23	751	4238	35	228	14	27	73	58	3.10	0.18	0.37	0.79	0.06	0.12	0.32	0.25
1614	TR2 UN A1-A4 H0-H1Sp1 collapse one each	Kelbadjar	2	0 - H1Sp1	collapse	A1-A4	-	242	7/5/23	510	5319	41	165	18	11	118	37	1.40	0.15	0.10	0.32	0.11	0.07	0.71	0.23
1615	TR2 UN A1-A4 H0-H1Sp1 collapse one each	Gegham 1	2	0 - H1Sp1	collapse	A1-A4	-	243	7/5/23	702	3914	31	209	11	27	75	57	2.77	0.15	0.35	0.75	0.05	0.13	0.36	0.27
1616	TR2 UN A1-A4 H0-H1Sp1 collapse one each	Gegham 1	2	0 - H1Sp1	collapse	A1-A4	-	244	7/5/23	782	4088	35	217	14	25	73	55	2.95	0.18	0.34	0.75	0.06	0.11	0.34	0.25
1617	TR2 UN A1-A4 H0-H1Sp1 collapse one each	Gegham 1	2	0 - H1Sp1	collapse	A1-A4	-	245	7/5/23	759	4369	34	227	14	25	73	57	3.08	0.18	0.34	0.77	0.06	0.11	0.32	0.25
1618	TR2 UN A1-A4 H0-H1Sp1 collapse one each	Gegham 1	2	0 - H1Sp1	collapse	A1-A4	-	246	7/5/23	778	4339	40	232	13	25	77	53	3.00	0.16	0.32	0.68	0.05	0.11	0.33	0.23
1619	TR2 UN A1-A4 H0-H1Sp1 collapse one each	Kelbadjar	2	0 - H1Sp1	collapse	A1-A4	-	247	7/5/23	513	5376	38	164	18	11	109	39	1.50	0.16	0.10	0.35	0.11	0.06	0.67	0.24
1620	TR2 UN A1-A4 H0-H1Sp1 collapse one each	Gegham 1	2	0 - H1Sp1	collapse	A1-A4	-	248	7/5/23	741	4141	35	218	14	25	73	57	3.00	0.19	0.34	0.78	0.06	0.11	0.33	0.26
1621	TR2 UN A1-A4 H0-H1Sp1 collapse one each	Gegham 1	2	0 - H1Sp1	collapse	A1-A4	-	250	7/5/23	784	4357	43	231	14	26	76	55	3.02	0.18	0.34	0.72	0.06	0.11	0.33	0.24
1622	TR2 UN A1-A4 H0-H1Sp1 collapse one each	Gegham 1	2	0 - H1Sp1	collapse	A1-A4	-	251	7/5/23	828	4527	41	231	15	27	80	59	2.88	0.18	0.34	0.74	0.06	0.12	0.35	0.26
1623	TR2 UN A1-A4 H0-H1Sp1 collapse one each	Arteni	2	0 - H1Sp1	collapse	A1-A4	-	252	7/5/23	537	4604	45	122	48	26	91	29	1.35	0.53	0.28	0.32	0.39	0.21	0.74	0.24
1624	TR2 UN A1-A4 H0-H1Sp1 collapse one each	Gegham 1	2	0 - H1Sp1	collapse	A1-A4	-	253	7/5/23	701	3956	35	213	14	25	75	54	2.83	0.18	0.33	0.71	0.06	0.12	0.35	0.25
1625	TR2 UN A1-A4 H0-H1Sp1 collapse one each	Gegham 1	2	0 - H1Sp1	collapse	A1-A4	-	254	7/5/23	773	4232	36	225	13	27	74	58	3.02	0.17	0.36	0.78	0.06	0.12	0.33	0.26
1626	TR2 UN A1-A4 H0-H1Sp1 collapse one each	Gegham 1	2	0 - H1Sp1	collapse	A1-A4	-	255	7/5/23	766	4216	35	223	15	27	75	58	2.96	0.19	0.36	0.77	0.07	0.12	0.34	0.26
1627	TR2 UN A1-A4 H0-H1Sp1 collapse one each	Kelbadjar	2	0 - H1Sp1	collapse	A1-A4	-	256	7/5/23	520	5214	42	159	18	11	111	35	1.43	0.16	0.09	0.31	0.11	0.07	0.70	0.22
1628	TR2 UN A1-A4 H0-H1Sp1 collapse one each	Gegham 1	2	0 - H1Sp1	collapse	A1-A4	-	257	7/5/23	729	4016	34	220	14	24	71	51	3.11	0.19	0.34	0.72	0.06	0.11	0.32	0.23

1629	TR2 UN A1-A4 H0-H1Sp1 collapse one each	Gegham 1	2	0 - H1Sp1	collapse	A1-A4	-	259	7/5/23	691	3947	35	211	14	26	75	54	2.80	0.18	0.34	0.71	0.06	0.12	0.36	0.26
1630	TR2 UN A1-A4 H0-H1Sp1 collapse one each	Gegham 1	2	0 - H1Sp1	collapse	A1-A4	-	260	7/5/23	660	3950	35	216	14	28	75	57	2.87	0.18	0.38	0.75	0.06	0.13	0.35	0.26
1631	TR2 UN A1-A4 H0-H1Sp1 collapse one each	Gegham 1	2	0 - H1Sp1	collapse	A1-A4	-	261	7/5/23	729	4081	37	225	14	26	76	61	2.94	0.18	0.34	0.80	0.06	0.11	0.34	0.27
1632	TR2 UN A1-A4 H0-H1Sp1 collapse one each	Gegham 1	2	0 - H1Sp1	collapse	A1-A4	-	262	7/5/23	666	3724	35	213	15	27	73	55	2.94	0.20	0.38	0.76	0.07	0.13	0.34	0.26
1633	TR2 UN A1-A4 H0-H1Sp1 collapse one each	Syunik	2	0 - H1Sp1	collapse	A1-A4	-	263	7/5/23	536	5587	39	195	21	10	111	35	1.75	0.18	0.09	0.31	0.11	0.05	0.57	0.18
1634	TR2 UN A1-A4 H0-H1Sp1 collapse one each	Gegham 1	2	0 - H1Sp1	collapse	A1-A4	-	264	7/5/23	656	3693	29	202	13	27	73	54	2.75	0.17	0.36	0.73	0.06	0.13	0.36	0.27
1635	TR2 UN A1-A4 H0-H1Sp1 collapse one each	Kelbadjar	2	0 - H1Sp1	collapse	A1-A4	-	266	7/5/23	566	5668	43	173	16	11	110	35	1.57	0.14	0.10	0.31	0.09	0.06	0.64	0.20
1636	TR2 UN A1-A4 H0-H1Sp1 collapse one each	Gegham 1	2	0 - H1Sp1	collapse	A1-A4	-	267	7/5/23	792	4717	42	237	15	27	83	57	2.86	0.17	0.33	0.68	0.06	0.12	0.35	0.24
1637	TR2 UN A1-A4 H0-H1Sp1 collapse one each	Gegham 1	2	0 - H1Sp1	collapse	A1-A4	-	268	7/5/23	808	4423	42	234	13	25	76	53	3.07	0.16	0.32	0.69	0.05	0.11	0.33	0.22
1638	TR2 UN A1-A4 H0-H1Sp1 collapse one each	Arteni	2	0 - H1Sp1	collapse	A1-A4	-	269	7/5/23	748	5346	49	149	36	27	96	32	1.55	0.37	0.29	0.33	0.24	0.18	0.65	0.21
1639	TR2 UN A1-A4 H0-H1Sp1 collapse one each	Kelbadjar	2	0 - H1Sp1	collapse	A1-A4	-	270	7/5/23	546	5509	41	169	17	11	119	36	1.42	0.14	0.10	0.30	0.10	0.07	0.70	0.21
1640	TR2 UN A1-A4 H0-H1Sp1 collapse one each	Gegham 1	2	0 - H1Sp1	collapse	A1-A4	-	271	7/5/23	792	4672	45	242	19	25	72	58	3.38	0.26	0.34	0.81	0.08	0.10	0.30	0.24
1641	TR2 UN A1-A4 H0-H1Sp1 collapse one each	Gegham 1	2	0 - H1Sp1	collapse	A1-A4	-	272	7/5/23	782	4375	39	228	16	27	78	57	2.91	0.20	0.34	0.72	0.07	0.12	0.34	0.25
1642	TR2 UN A1-A4 H0-H1Sp1 collapse one each	Kelbadjar	2	0 - H1Sp1	collapse	A1-A4	-	273	7/5/23	642	6596	51	185	19	12	121	40	1.53	0.15	0.10	0.33	0.10	0.07	0.65	0.22
1643	TR2 UN A1-A4 H0-H1Sp1 collapse one each	Gegham 1	2	0 - H1Sp1	collapse	A1-A4	-	274	7/5/23	779	4249	35	229	15	29	79	57	2.89	0.18	0.37	0.72	0.06	0.13	0.35	0.25
1644	TR2 UN A1-A4 H0-H1Sp1 collapse one each	Gegham 1	2	0 - H1Sp1	collapse	A1-A4	-	275	7/5/23	779	4252	39	230	13	26	76	55	3.01	0.16	0.34	0.72	0.05	0.11	0.33	0.24
1645	TR2 UN A1-A4 H0-H1Sp1 collapse one each	Kelbadjar	2	0 - H1Sp1	collapse	A1-A4	-	276	7/5/23	586	6047	51	180	18	11	112	36	1.60	0.16	0.09	0.32	0.10	0.06	0.62	0.20
1646	TR2 UN A1-A4 H0-H1Sp1 collapse one each	Kelbadjar	2	0 - H1Sp1	collapse	A1-A4	-	277	7/5/23	556	5975	49	180	18	10	113	36	1.59	0.15	0.08	0.32	0.10	0.05	0.63	0.20
1647	TR2 UN A1-A4 H0-H1Sp1 collapse one each	Kelbadjar	2	0 - H1Sp1	collapse	A1-A4	-	278	7/5/23	552	5624	44	169	17	12	108	36	1.56	0.15	0.11	0.33	0.10	0.07	0.64	0.21
1648	TR2 UN A1-A4 H0-H1Sp1 collapse one each	Gegham 1	2	0 - H1Sp1	collapse	A1-A4	-	279	7/5/23	869	4893	40	248	14	27	78	58	3.17	0.17	0.34	0.74	0.05	0.11	0.32	0.23
1649	TR2 UN A1-A4 H0-H1Sp1 collapse one each	Kelbadjar	2	0 - H1Sp1	collapse	A1-A4	-	280	7/5/23	606	6167	48	183	17	11	111	36	1.64	0.15	0.09	0.32	0.09	0.06	0.61	0.20
1650	TR2 UN A1-A4 H0-H1Sp1 collapse one each	Gegham 1	2	0 - H1Sp1	collapse	A1-A4	-	281	7/5/23	782	4315	37	231	14	27	80	58	2.88	0.17	0.33	0.72	0.06	0.12	0.35	0.25
1651	TR2 UN A1-A4 H0-H1Sp1 collapse one each	Gegham 1	2	0 - H1Sp1	collapse	A1-A4	-	282	7/5/23	915	4890	48	243	15	26	73	53	3.31	0.20	0.35	0.71	0.06	0.11	0.30	0.22
1652	TR2 UN A1-A4 H0-H1Sp1 collapse one each	Gegham 1	2	0 - H1Sp1	collapse	A1-A4	-	283	7/5/23	737	4281	38	230	13	23	78	57	2.94	0.16	0.29	0.72	0.05	0.10	0.34	0.25
1653	TR2 UN A1-A4 H0-H1Sp1 collapse one each	Gegham 1	2	0 - H1Sp1	collapse	A1-A4	-	284	7/5/23	682	3834	40	218	18	27	74	57	2.93	0.24	0.37	0.76	0.08	0.13	0.34	0.26
1654	TR2 UN A1-A4 H0-H1Sp1 collapse one each	Gegham 1	2	0 - H1Sp1	collapse	A1-A4	-	285	7/5/23	726	3984	35	216	13	24	75	54	2.87	0.17	0.31	0.71	0.06	0.11	0.35	0.25
1655	TR2 UN A1-A4 H0-H1Sp1 collapse one each	Kelbadjar	2	0 - H1Sp1	collapse	A1-A4	-	286	7/5/23	585	6023	46	176	16	11	115	35	1.53	0.13	0.10	0.30	0.09	0.07	0.65	0.20
1656	TR2 UN A1-A4 H0-H1Sp1 collapse one each	Gegham 1	2	0 - H1Sp1	collapse	A1-A4	-	287	7/5/23	921	4986	42	252	15	25	79	61	3.18	0.18	0.31	0.77	0.06	0.10	0.31	0.24
1657	TR.2 A5 H5 S2 02.07.23 one each	Gegham 1	2	5	2	A5	7/2/23	312	7/5/23	678	4058	35	210	13	24	72	58	2.94	0.17	0.33	0.81	0.06	0.11	0.34	0.28
1658	TR.2 A5 H5 S2 02.07.23 one each	Gegham 1	2	5	2	A5	7/2/23	313	7/5/23	729	4315	36	211	13	23	72	55	2.95	0.17	0.32	0.77	0.06	0.11	0.34	0.26
1659	TR.2 A5 H5 S2 02.07.23 one each	Gegham 1	2	5	2	A5	7/2/23	314	7/5/23	602	3995	36	198	13	26	70	50	2.85	0.18	0.37	0.71	0.06	0.13	0.35	0.25
1660	TR.2 A5 H5 S2 02.07.23 one each	Gegham 1	2	5	2	A5	7/2/23	315	7/5/23	663	4315	36	212	15	26	74	55	2.85	0.19	0.34	0.74	0.07	0.12	0.35	0.26
1661	TR.2 A5 H5 S2 02.07.23 one each	Gegham 1	2	5	2	A5	7/2/23	316	7/5/23	655	3649	31	200	13	22	72	50	2.80	0.17	0.30	0.69	0.06	0.11	0.36	0.25
1662	TR.2 A5 H5 S2 02.07.23 one each	Gegham 1	2	5	2	A5	7/2/23	317	7/5/23	687	3908	34	208	11	25	73	55	2.83	0.16	0.34	0.75	0.06	0.12	0.35	0.27
1663	TR.2 A5 H5 S2 02.07.23 one each	Gegham 1	2	5	2	A5	7/2/23	318	7/5/23	707	3861	35	208	13	26	71	54	2.95	0.18	0.36	0.76	0.06	0.12	0.34	0.26
1664	TR.2 A5 H5 S2 02.07.23 one each	Gegham 1	2	5	2	A5	7/2/23	319	7/5/23	626	3724	36	208	13	26	73	54	2.87	0.17	0.35	0.74	0.06	0.12	0.35	0.26
1665	TR.2 A5 H5 S2 02.07.23 one each	Gegham 1	2	5	2	A5	7/2/23	320	7/5/23	671	3883	35	204	13	26	72	55	2.85	0.17	0.36	0.77	0.06	0.13	0.35	0.27
1666	TR.2 A5 H5 S2 02.07.23 one each	Syunik	2	5	2	A5	7/2/23	321	7/5/23	540	5774	37	188	23	11	104	33	1.81	0.22	0.10	0.32	0.12	0.06	0.55	0.18
1667	TR.2 A5 H5 S2 02.07.23 one each	Gegham 1	2	5	2	A5	7/2/23	322	7/5/23	632	3711	35	208	22	26	73	54	2.87	0.30	0.35	0.74	0.10	0.12	0.35	0.26
1668	TR.2 A5 H5 S2 02.07.23 one each	Syunik	2	5	2	A5	7/2/23	323	7/5/23	614	5096	33	213	13	9	92	39	2.31	0.14	0.09	0.42	0.06	0.04	0.43	0.18
1669	TR.2 A5 H5 S2 02.07.23 one each	Gegham 1	2	5	2	A5	7/2/23	324	7/5/23	718	4349	37	218	15	26	77	57	2.82	0.19	0.33	0.73	0.07	0.12	0.35	0.26
1670	TR.2 A5 H5 S2 02.07.23 one each	Gegham 1	2	5	2	A5	7/2/23	325	7/5/23	741	4240	36	223	15	25	73	57	3.03	0.20	0.34	0.77	0.07	0.11	0.33	0.25
1671	TR.2 A5 H5 S2 02.07.23 one each	Gegham 1	2	5	2	A5	7/2/23	326	7/5/23	732	4038	35	220	14	27	74	57	2.95	0.18	0.37	0.76	0.06	0.12	0.34	0.26
1672	TR.2 A5 H5 S2 02.07.23 one each	Gegham 1	2	5	2	A5	7/2/23	327	7/5/23	652	3684	33	206	11	26	72	55	2.88	0.16	0.36	0.77	0.06	0.12	0.35	0.27
1673	TR.2 A5 H5 S2 02.07.23 one each	Gegham 1	2	5	2	A5	7/2/23	328	7/5/23	667	4058	40	211	18	27	72	54	2.95	0.24	0.38	0.75	0.08	0.13	0.34	0.26
1674	TR.2 A5 H5 S2 02.07.23 one each	Gegham 1	2	5	2	A5	7/2/23	329	7/5/23	663	3838	33	213	13	25	71	54	3.02	0.18	0.35	0.76	0.06	0.12	0.33	0.25
1675	TR.2 A5 H5 S2 02.07.23 one each	Gegham 1	2	5	2	A5	7/2/23	330	7/5/23	736	4208	37	221	13	24	73	55	3.00	0.17	0.32	0.75	0.06	0.11	0.33	0.25
1676	TR.2 A5 H5 S2 02.07.23 one each	Gegham 1	2	5	2	A5	7/2/23	331	7/5/23	773	4300	36	223	14	27	73	58	3.03	0.18	0.36	0.79	0.06	0.12	0.33	0.26
1677	TR.2 A5 H5 S2 02.07.23 one each	Gegham 1	2	5	2	A5	7/2/23	332	7/5/23	643	3706	34	203	21	25	69	54	2.96	0.30	0.36	0.78	0.10	0.12	0.34	0.26
1678	TR.2 A5 H5 S2 02.07.23 one each	Gegham 1	2	5	2	A5	7/2/23	333	7/5/23	738	4081	42	219	14	26	75	57	2.90	0.18	0.34	0.75	0.06	0.12	0.34	0.26
1679	TR.2 A5 H5 S2 02.07.23 one each	Gegham 1	2	5	2	A5	7/2/23	334	7/5/23	746	4106	35	219	14	27	74	58	2.94	0.18	0.36	0.78	0.06	0.12	0.34	0.26

1680	TR.2 A5 H5 S2 02.07.23 one each	Gegham 1	2	5	2	A5	7/2/23	335	7/5/23	813	4496	43	235	14	25	75	55	3.12	0.18	0.33	0.73	0.06	0.10	0.32	0.23
1681	TR.2 A5 H5 S2 02.07.23 one each	Gegham 1	2	5	2	A5	7/2/23	336	7/5/23	796	4339	40	234	15	27	80	58	2.93	0.18	0.34	0.72	0.06	0.12	0.34	0.25
1682	TR.2 A5 H5 S2 02.07.23 one each	Syunik	2	5	2	A5	7/2/23	337	7/5/23	581	4973	41	206	13	11	92	39	2.23	0.14	0.12	0.42	0.06	0.06	0.45	0.19
1683	TR.2 A5 H5 S2 02.07.23 one each	Syunik	2	5	2	A5	7/2/23	338	7/5/23	681	5211	49	237	8	10	99	42	2.40	0.09	0.10	0.42	0.04	0.04	0.42	0.17
1684	TR.2 A5 H5 S2 02.07.23 one each	Gegham 1	2	5	2	A5	7/2/23	339	7/5/23	741	4786	40	223	18	27	75	57	2.96	0.23	0.35	0.75	0.08	0.12	0.34	0.25
1685	TR.2 A5 H5 S2 02.07.23 one each	Kelbadjar	2	5	2	A5	7/2/23	340	7/5/23	619	5924	47	178	18	12	119	39	1.50	0.15	0.10	0.33	0.10	0.07	0.67	0.22
1686	TR.2 A5 H5 S2 02.07.23 one each	Gegham 1	2	5	2	A5	7/2/23	341	7/5/23	923	4871	43	249	15	26	80	59	3.11	0.18	0.32	0.74	0.06	0.10	0.32	0.24
1687	TR.2 Unit A0 H1 S1 02.07.23 one each	Syunik	2	1	1	A0	7/2/23	343	7/5/23	475	4989	32	177	21	12	108	35	1.65	0.19	0.12	0.32	0.12	0.07	0.61	0.20
1688	TR.2 Unit A0 H1 S1 02.07.23 one each	Arteni	2	1	1	A0	7/2/23	344	7/5/23	557	4467	39	122	38	27	88	25	1.39	0.43	0.31	0.29	0.31	0.23	0.72	0.21
1689	TR.2 Unit A0 H1 S1 02.07.23 one each	Syunik	2	1	1	A0	7/2/23	345	7/5/23	508	5466	34	185	22	11	110	36	1.68	0.20	0.10	0.33	0.12	0.06	0.60	0.19
1690	TR.2 Unit A0 H1 S1 02.07.23 one each	Kelbadjar	2	1	1	A0	7/2/23	346	7/5/23	525	5437	44	164	17	11	112	33	1.46	0.15	0.10	0.30	0.10	0.07	0.69	0.20
1691	TR.2 Unit A0 H1 S1 02.07.23 one each	Gegham 1	2	1	1	A0	7/2/23	347	7/5/23	683	4029	35	219	13	27	74	55	2.94	0.17	0.36	0.74	0.06	0.12	0.34	0.25
1692	TR.2 Unit A0 H1 S1 02.07.23 one each	Gegham 1	2	1	1	A0	7/2/23	348	7/5/23	727	4227	35	226	16	26	74	55	3.03	0.21	0.34	0.74	0.07	0.11	0.33	0.24
1693	TR.2 Unit A0 H1 S1 02.07.23 one each	Syunik	2	1	1	A0	7/2/23	349	7/5/23	551	5995	45	208	21	8	106	40	1.97	0.19	0.07	0.38	0.10	0.04	0.51	0.19
1694	TR.2 Unit A0 H1 S1 02.07.23 one each	Gegham 1	2	1	1	A0	7/2/23	350	7/5/23	690	3847	35	216	14	25	72	54	3.02	0.19	0.34	0.75	0.06	0.11	0.33	0.25
1695	TR.2 Unit A0 H1 S1 02.07.23 one each	Gegham 1	2	1	1	A0	7/2/23	351	7/5/23	683	4093	38	222	15	26	75	58	2.94	0.19	0.34	0.77	0.07	0.12	0.34	0.26
1696	TR.2 Unit A0 H1 S1 02.07.23 one each	Kelbadjar	2	1	1	A0	7/2/23	352	7/5/23	560	5652	40	173	17	12	121	37	1.43	0.14	0.10	0.31	0.10	0.07	0.70	0.22
1697	TR.2 Unit A0 H1 S1 02.07.23 one each	Gegham 1	2	1	1	A0	7/2/23	353	7/5/23	729	4096	35	225	13	27	74	58	3.02	0.17	0.36	0.78	0.06	0.12	0.33	0.26
1698	TR.2 Unit A0 H1 S1 02.07.23 one each	Gegham 1	2	1	1	A0	7/2/23	354	7/5/23	784	4413	40	233	14	27	78	55	2.98	0.17	0.35	0.71	0.06	0.12	0.34	0.24
1699	TR.2 Unit A0 H1 S1 02.07.23 one each	Syunik	2	1	1	A0	7/2/23	355	7/5/23	528	6296	42	187	26	13	110	35	1.69	0.23	0.12	0.31	0.14	0.07	0.59	0.19
1700	Tr-2 B0 H1 S2 03.07.23 one each	Gegham 1	2	1	2	B0	7/3/23	356	7/5/23	681	4009	32	213	11	24	73	54	2.94	0.16	0.33	0.74	0.05	0.11	0.34	0.25
1701	Tr-2 B0 H1 S2 03.07.23 one each	Kelbadjar	2	1	2	B0	7/3/23	357	7/5/23	500	5433	40	162	17	10	114	35	1.42	0.14	0.08	0.30	0.10	0.06	0.71	0.21
1702	Tr-2 B0 H1 S2 03.07.23 one each	Gegham 1	2	1	2	B0	7/3/23	358	7/5/23	645	3772	33	211	18	27	73	55	2.87	0.24	0.36	0.75	0.08	0.13	0.35	0.26
1703	Tr-2 B0 H1 S2 03.07.23 one each	Gegham 1	2	1	2	B0	7/3/23	359	7/5/23	688	4133	36	219	21	24	72	55	3.06	0.29	0.33	0.77	0.09	0.11	0.33	0.25
1704	Tr-2 B0 H1 S2 03.07.23 one each	Kelbadjar	2	1	2	B0	7/3/23	360	7/5/23	535	5723	45	167	18	10	115	35	1.45	0.15	0.08	0.30	0.10	0.06	0.69	0.21
1705	Tr-2 B0 H1 S2 03.07.23 one each	Syunik	2	1	2	B0	7/3/23	361	7/5/23	534	5575	37	196	20	10	106	36	1.85	0.18	0.09	0.34	0.10	0.05	0.54	0.18
1706	Tr-2 B0 H1 S2 03.07.23 one each	Gegham 1	2	1	2	B0	7/3/23	362	7/5/23	663	3979	35	216	14	26	73	57	2.94	0.18	0.35	0.77	0.06	0.12	0.34	0.26
1707	Tr-2 B0 H1 S2 03.07.23 one each	Gegham 1	2	1	2	B0	7/3/23	363	7/5/23	880	4869	40	245	14	27	78	58	3.13	0.17	0.35	0.74	0.06	0.11	0.32	0.24
1708	Tr-2 B0 H1 S2 03.07.23 one each	Gegham 1	2	1	2	B0	7/3/23	364	7/5/23	729	4158	35	222	14	26	76	57	2.91	0.18	0.34	0.74	0.06	0.12	0.34	0.26
1709	Tr-2 B0 H1 S2 03.07.23 one each	Kelbadjar	2	1	2	B0	7/3/23	365	7/5/23	580	5770	46	176	18	13	166	39	1.06	0.11	0.08	0.23	0.10	0.08	0.94	0.22
1710	Tr-2 B0 H1 S2 03.07.23 one each	Gegham 1	2	1	2	B0	7/3/23	366	7/5/23	681	3973	34	217	14	27	73	53	2.95	0.18	0.36	0.71	0.06	0.12	0.34	0.24
1711	Tr-2 B0 H1 S2 03.07.23 one each	Kelbadjar	2	1	2	B0	7/3/23	367	7/5/23	580	5905	45	180	19	11	109	40	1.65	0.17	0.10	0.37	0.10	0.06	0.61	0.22
1712	Tr-2 B0 H1 S2 03.07.23 one each	Syunik	2	1	2	B0	7/3/23	368	7/5/23	504	5171	43	181	30	11	108	33	1.67	0.27	0.11	0.31	0.16	0.06	0.60	0.18
1713	Tr-2 B0 H1 S2 03.07.23 one each	Gegham 1	2	1	2	B0	7/3/23	370	7/5/23	741	4157	35	233	14	28	77	58	3.01	0.17	0.37	0.75	0.06	0.12	0.33	0.25
1714	Tr-2 B0 H1 S2 03.07.23 one each	Gegham 1	2	1	2	B0	7/3/23	371	7/5/23	1000	5269	47	256	15	27	79	59	3.23	0.18	0.34	0.75	0.06	0.10	0.31	0.23
1715	Tr-2 B0 H1 S2 03.07.23 one each	Gegham 1	2	1	2	B0	7/3/23	372	7/5/23	888	4738	43	245	16	27	75	54	3.25	0.21	0.35	0.71	0.06	0.11	0.31	0.22
1716	Tr-2 B0 H1 S2 03.07.23 one each	Gegham 1	2	1	2	B0	7/3/23	373	7/5/23	802	4270	40	235	14	27	76	55	3.08	0.18	0.35	0.72	0.06	0.11	0.32	0.23
1717	TR.2 C0 H1 S2 03.07.23 one each	Gegham 1	2	1	2	C0	7/3/23	374	7/5/23	787	4394	40	231	15	24	74	57	3.10	0.19	0.32	0.76	0.06	0.10	0.32	0.25
1718	TR.2 C0 H1 S2 03.07.23 one each	Gegham 1	2	1	2	C0	7/3/23	375	7/5/23	731	4216	33	222	13	24	73	54	3.02	0.17	0.32	0.73	0.06	0.11	0.33	0.24
1719	TR.2 C0 H1 S2 03.07.23 one each	Syunik	2	1	2	C0	7/3/23	376	7/5/23	506	5420	37	181	21	11	106	37	1.71	0.19	0.11	0.35	0.11	0.06	0.58	0.21
1720	TR.2 C0 H1 S2 03.07.23 one each	Syunik	2	1	2	C0	7/3/23	377	7/5/23	670	6787	50	190	23	9	124	35	1.54	0.18	0.07	0.28	0.12	0.05	0.65	0.18
1721	TR.2 C0 H1 S2 03.07.23 one each	Syunik	2	1	2	C0	7/3/23	378	7/5/23	511	5426	37	187	25	11	110	36	1.69	0.22	0.10	0.33	0.13	0.06	0.59	0.19
1722	TR.2 C1 H1 S2 03.07.23 one each	Gegham 1	2	1	2	C0	7/3/23	379	7/5/23	677	3796	30	210	11	27	73	55	2.90	0.16	0.37	0.76	0.05	0.13	0.35	0.26
1723	TR.2 C1 H1 S2 03.07.23 one each	Gegham 1	2	1	2	C0	7/3/23	380	7/5/23	711	3951	34	214	13	23	69	55	3.11	0.18	0.33	0.80	0.06	0.11	0.32	0.26
1724	TR.2 C1 H1 S2 03.07.23 one each	Gegham 1	2	1	2	C0	7/3/23	381	7/5/23	768	4282	35	225	13	27	80	55	2.81	0.16	0.33	0.69	0.06	0.12	0.36	0.25
1725	TR.2 C1 H1 S2 03.07.23 one each	Syunik	2	1	2	C0	7/3/23	382	7/5/23	541	5787	40	191	24	10	108	36	1.76	0.22	0.09	0.33	0.12	0.05	0.57	0.19
1726	TR.2 C1 H1 S2 03.07.23 one each	Gegham 1	2	1	2	C0	7/3/23	383	7/5/23	743	4356	45	211	13	26	74	57	2.84	0.17	0.34	0.76	0.06	0.12	0.35	0.27
1727	TR.2 C1 H1 S2 03.07.23 one each	Kelbadjar	2	1	2	C0	7/3/23	384	7/5/23	584	5848	47	176	18	12	113	37	1.56	0.15	0.11	0.33	0.10	0.07	0.64	0.21
1728	TR2 Unit-C0 H1 S1 02.07.23 one each	Syunik	2	1	1	C0	7/2/23	385	7/5/23	515	5378	34	178	26	11	106	35	1.69	0.24	0.11	0.33	0.14	0.06	0.59	0.19
1729	TR2 Unit-C0 H1 S1 02.07.23 one each	Tsaghkunyats	2	1	1	C0	7/2/23	386	7/5/23	486	6874	31	100	193	9	112	22	0.89	1.72	0.08	0.20	1.92	0.09	1.12	0.22
1730	TR2 Unit-C0 H1 S1 02.07.23 one each	Gegham 1	2	1	1	C0	7/2/23	387	7/5/23	786	4412	40	228	13	26	73	57	3.10	0.17	0.35	0.77	0.05	0.11	0.32	0.25

1731	TR2 Unit-C0 H1 S1 02.07.23 one each	Gegham 1	2	1	1	C0	7/2/23	388	7/5/23	743	4095	40	218	15	27	74	54	2.93	0.19	0.36	0.72	0.07	0.12	0.34	0.25
1732	TR2 Unit-C0 H1 S1 02.07.23 one each	Gegham 1	2	1	1	C0	7/2/23	390	7/5/23	811	4518	35	235	14	26	74	53	3.16	0.18	0.34	0.71	0.06	0.11	0.32	0.22
1733	TR2 Unit-C0 H1 S1 02.07.23 one each	Syunik	2	1	1	C0	7/2/23	391	7/5/23	468	5331	38	177	24	11	108	33	1.63	0.22	0.11	0.31	0.13	0.06	0.61	0.19
1734	TR2 Unit-C0 H1 S1 02.07.23 one each	Gegham 1	2	1	1	C0	7/2/23	392	7/5/23	712	4050	35	223	13	26	75	55	2.96	0.17	0.34	0.73	0.06	0.11	0.34	0.25
1735	TR2 Unit-C0 H1 S1 02.07.23 one each	Gegham 1	2	1	1	C0	7/2/23	393	7/5/23	656	3786	36	206	16	27	75	58	2.74	0.21	0.35	0.77	0.08	0.13	0.37	0.28
1736	TR2 Unit-C0 H1 S1 02.07.23 one each	Gegham 1	2	1	1	C0	7/2/23	394	7/5/23	955	5149	48	257	16	25	74	59	3.45	0.21	0.33	0.80	0.06	0.10	0.29	0.23
1737	TR2 Unit-C0 H1 S1 02.07.23 one each	Kelbadjar	2	1	1	C0	7/2/23	395	7/5/23	601	5762	46	175	21	10	122	33	1.44	0.17	0.08	0.27	0.12	0.05	0.69	0.19
1738	TR2 Unit-C0 H1 S1 02.07.23 one each	Syunik	2	1	1	C0	7/2/23	397	7/5/23	518	5633	42	196	24	10	110	36	1.77	0.21	0.09	0.33	0.12	0.05	0.56	0.18
1739	TR2 Unit-C0 H1 S1 02.07.23 one each	Gegham 1	2	1	1	C0	7/2/23	398	7/5/23	879	4548	43	241	14	22	73	54	3.33	0.19	0.30	0.74	0.06	0.09	0.30	0.22
1740	TR.2 A1 H2 S1 28.06.23 one each	Kelbadjar	2	2	1	A1	6/28/23	399	7/5/23	476	5120	39	160	19	11	111	36	1.43	0.17	0.10	0.32	0.12	0.07	0.70	0.23
1741	TR.2 A1 H2 S1 28.06.23 one each	Gegham 1	2	2	1	A1	6/28/23	400	7/5/23	705	4071	34	219	13	25	76	58	2.87	0.16	0.32	0.76	0.06	0.11	0.35	0.26
1742	TR.2 A1 H2 S1 28.06.23 one each	Kelbadjar	2	2	1	A1	6/28/23	401	7/5/23	518	5402	41	166	18	11	108	36	1.53	0.16	0.11	0.33	0.11	0.07	0.65	0.22
1743	TR.2 A1 H2 S1 28.06.23 one each	Kelbadjar	2	2	1	A1	6/28/23	402	7/5/23	510	5191	37	163	18	11	111	35	1.46	0.16	0.10	0.31	0.11	0.07	0.68	0.21
1744	TR.2 A1 H2 S1 28.06.23 one each	Kelbadjar	2	2	1	A1	6/28/23	403	7/5/23	523	5258	37	164	17	11	112	36	1.47	0.15	0.09	0.32	0.10	0.06	0.68	0.22
1745	TR.2 A1 H2 S1 28.06.23 one each	Kelbadjar	2	2	1	A1	6/28/23	404	7/5/23	530	5275	40	164	17	11	109	36	1.49	0.15	0.10	0.33	0.10	0.06	0.67	0.22
1746	TR.2 A1 H2 S1 28.06.23 one each	Arteni	2	2	1	A1	6/28/23	405	7/5/23	716	5381	45	143	41	26	92	25	1.55	0.44	0.28	0.27	0.28	0.18	0.65	0.18
1747	TR.2 A1 H2 S1 28.06.23 one each	Kelbadjar	2	2	1	A1	6/28/23	406	7/5/23	587	5665	46	170	17	10	109	35	1.56	0.15	0.09	0.32	0.10	0.06	0.64	0.20
1748	TR.2 A1 H2 S1 28.06.23 one each	Kelbadjar	2	2	1	A1	6/28/23	407	7/5/23	501	5271	38	163	17	12	111	37	1.46	0.15	0.11	0.34	0.10	0.08	0.68	0.23
1749	TR.2 A1 H2 S1 28.06.23 one each	Arteni	2	2	1	A1	6/28/23	408	7/5/23	675	4376	46	147	23	29	83	35	1.77	0.27	0.35	0.42	0.15	0.20	0.56	0.24
1750	TR.2 A1 H2 S1 28.06.23 one each	Kelbadjar	2	2	1	A1	6/28/23	409	7/5/23	597	6161	48	177	19	10	117	36	1.51	0.16	0.08	0.31	0.10	0.05	0.66	0.20
1751	TR.2 A1 H2 S1 28.06.23 one each	Kelbadjar	2	2	1	A1	6/28/23	410	7/5/23	498	5525	40	160	18	11	125	36	1.28	0.14	0.08	0.29	0.11	0.07	0.78	0.23
1752	TR.2 A1 H2 S1 28.06.23 one each	Gegham 1	2	2	1	A1	6/28/23	411	7/5/23	683	3684	32	211	11	25	73	57	2.91	0.16	0.34	0.78	0.05	0.12	0.34	0.27
1753	TR.2 A1 H2 S1 28.06.23 one each	Kelbadjar	2	2	1	A1	6/28/23	412	7/5/23	520	5249	43	162	18	11	112	35	1.44	0.16	0.10	0.31	0.11	0.07	0.69	0.21
1754	TR.2 A1 H2 S1 28.06.23 one each	Arteni	2	2	1	A1	6/28/23	413	7/5/23	614	4804	37	128	38	26	91	28	1.41	0.41	0.28	0.31	0.29	0.20	0.71	0.22
1755	TR.2 A1 H2 S1 28.06.23 one each	Kelbadjar	2	2	1	A1	6/28/23	414	7/5/23	513	5316	42	164	16	11	110	37	1.49	0.14	0.10	0.34	0.09	0.06	0.67	0.23
1756	TR.2 A1 H2 S1 28.06.23 one each	Syunik	2	2	1	A1	6/28/23	415	7/5/23	723	7046	57	198	20	10	119	35	1.66	0.16	0.08	0.29	0.10	0.05	0.60	0.18
1757	TR.2 A1 H2 S1 28.06.23 one each	Arteni	2	2	1	A1	6/28/23	416	7/5/23	614	4812	45	133	40	27	91	26	1.47	0.44	0.29	0.29	0.30	0.20	0.68	0.20
1758	TR.2 A1 H2 S1 28.06.23 one each	Kelbadjar	2	2	1	A1	6/28/23	417	7/5/23	552	5724	47	175	19	11	108	36	1.61	0.17	0.10	0.33	0.11	0.06	0.62	0.21
1759	TR.2 A1 H2 S1 28.06.23 one each	Syunik	2	2	1	A1	6/28/23	418	7/5/23	663	6478	54	188	17	10	117	36	1.61	0.14	0.08	0.31	0.09	0.05	0.62	0.19
1760	TR.2 A1 H2 S1 28.06.23 one each	Kelbadjar	2	2	1	A1	6/28/23	419	7/5/23	596	5955	46	175	17	12	115	33	1.52	0.14	0.11	0.29	0.09	0.07	0.66	0.19
1761	TR.2 A1 H2 S1 28.06.23 one each	Tsaghkunyats	2	2	1	A1	6/28/23	420	7/5/23	609	5582	64	137	202	10	108	29	1.28	1.88	0.09	0.27	1.47	0.07	0.78	0.21
1762	Tr2 A2-A3 H2 S1 20.06 one each	Gegham 1	2	2	1	A2-A3	6/30/23?	1	7/6/23	640	3860	38	206	19	24	70	57	2.96	0.27	0.34	0.81	0.09	0.11	0.34	0.27
1763	Tr2 A2-A3 H2 S1 20.06 one each	Syunik	2	2	1	A2-A3	6/30/23?	2	7/6/23	485	5289	36	176	25	11	104	35	1.70	0.24	0.11	0.33	0.14	0.07	0.59	0.20
1764	Tr2 A2-A3 H2 S1 20.06 one each	Gegham 1	2	2	1	A2-A3	6/30/23?	3	7/6/23	712	3890	30	218	14	26	73	58	3.00	0.19	0.35	0.80	0.06	0.12	0.33	0.27
1765	Tr2 A2-A3 H2 S1 20.06 one each	Gegham 1	2	2	1	A2-A3	6/30/23?	4	7/6/23	707	4008	33	214	14	27	73	54	2.95	0.19	0.38	0.74	0.06	0.13	0.34	0.25
1766	Tr2 A2-A3 H2 S1 20.06 one each	Gegham 1	2	2	1	A2-A3	6/30/23?	5	7/6/23	779	4405	41	226	14	27	73	54	3.11	0.19	0.37	0.74	0.06	0.12	0.32	0.24
1767	Tr2 A2-A3 H2 S1 20.06 one each	Gegham 1	2	2	1	A2-A3	6/30/23?	6	7/6/23	751	4143	35	211	14	27	73	58	2.87	0.18	0.36	0.79	0.06	0.13	0.35	0.27
1768	Tr2 A2-A3 H2 S1 20.06 one each	Gegham 1	2	2	1	A2-A3	6/30/23?	7	7/6/23	622	3754	33	205	17	27	73	54	2.83	0.23	0.37	0.74	0.08	0.13	0.35	0.26
1769	Tr2 A2-A3 H2 S1 20.06 one each	Kelbadjar	2	2	1	A2-A3	6/30/23?	8	7/6/23	496	4946	38	156	18	11	107	36	1.46	0.16	0.11	0.34	0.11	0.07	0.68	0.23
1770	Tr2 A2-A3 H2 S1 20.06 one each	Gegham 1	2	2	1	A2-A3	6/30/23?	9	7/6/23	645	3858	37	209	18	25	73	51	2.85	0.24	0.34	0.70	0.08	0.12	0.35	0.24
1771	Tr2 A2-A3 H2 S1 20.06 one each	Gegham 1	2	2	1	A2-A3	6/30/23?	10	7/6/23	620	3560	34	201	14	27	71	50	2.85	0.19	0.38	0.70	0.07	0.13	0.35	0.25
1772	Tr2 A2-A3 H2 S1 20.06 one each	Gegham 1	2	2	1	A2-A3	6/30/23?	11	7/6/23	722	4064	31	213	15	26	74	53	2.86	0.19	0.34	0.71	0.07	0.12	0.35	0.25
1773	Tr2 A2-A3 H2 S1 20.06 one each	Kelbadjar	2	2	1	A2-A3	6/30/23?	12	7/6/23	530	5322	39	163	16	12	117	39	1.39	0.13	0.11	0.33	0.10	0.08	0.72	0.24
1774	Tr2 A2-A3 H2 S1 20.06 one each	Gegham 1	2	2	1	A2-A3	6/30/23?	13	7/6/23	692	3813	34	209	14	25	73	53	2.85	0.18	0.34	0.71	0.06	0.12	0.35	0.25
1775	Tr2 A2-A3 H2 S1 20.06 one each	Gegham 1	2	2	1	A2-A3	6/30/23?	14	7/6/23	707	4243	32	224	14	25	76	55	2.93	0.18	0.32	0.72	0.06	0.11	0.34	0.25
1776	Tr2 A2-A3 H2 S1 20.06 one each	Kelbadjar	2	2	1	A2-A3	6/30/23?	15	7/6/23	584	5952	47	178	18	10	114	40	1.56	0.15	0.08	0.35	0.10	0.05	0.64	0.23
1777	Tr2 A2-A3 H2 S1 20.06 one each	Gegham 1	2	2	1	A2-A3	6/30/23?	16	7/6/23	784	4327	35	225	14	26	73	55	3.06	0.18	0.35	0.75	0.06	0.11	0.33	0.25
1778	Tr2 A2-A3 H2 S1 20.06 one each	Gegham 1	2	2	1	A2-A3	6/30/23?	17	7/6/23	793	4436	37	233	13	25	75	55	3.10	0.17	0.33	0.73	0.05	0.11	0.32	0.24
1779	Tr2 A2-A3 H2 S1 20.06 one each	Gegham 1	2	2	1	A2-A3	6/30/23?	18	7/6/23	759	4294	34	233	15	25	74	55	3.12	0.19	0.33	0.74	0.06	0.11	0.32	0.24
1780	Tr2 A2-A3 H2 S1 20.06 one each	Kelbadjar	2	2	1	A2-A3	6/30/23?	19	7/6/23	546	5570	44	174	18	8	108	36	1.61	0.16	0.07	0.33	0.10	0.04	0.62	0.21
1781	Tr2 A2-A3 H2 S1 20.06 one each	Syunik	2	2	1	A2-A3	6/30/23?	20	7/6/23	471	4940	33	174	20	12	102	36	1.71	0.19	0.12	0.35	0.11	0.07	0.58	0.21

1782	Tr2 A2-A3 H2 S1 20.06 one each	Chikiani	2	2	1	A2-A3	6/30/23?	21	7/6/23	344	4205	42	138	97	12	96	29	1.44	1.01	0.13	0.30	0.70	0.09	0.70	0.21
1783	Tr2 A2-A3 H2 S1 20.06 one each	Gegham 1	2	2	1	A2-A3	6/30/23?	22	7/6/23	761	3954	35	216	15	26	75	54	2.87	0.19	0.34	0.71	0.07	0.12	0.35	0.25
1784	Tr2 A2-A3 H2 S1 20.06 one each	Kelbadjar	2	2	1	A2-A3	6/30/23?	23	7/6/23	575	5519	45	171	18	11	115	39	1.49	0.15	0.09	0.34	0.10	0.06	0.67	0.23
1785	Tr2 A2-A3 H2 S1 20.06 one each	Gegham 1	2	2	1	A2-A3	6/30/23?	24	7/6/23	768	4552	39	233	14	27	78	58	2.97	0.17	0.34	0.74	0.06	0.11	0.34	0.25
1786	Tr2 A2-A3 H2 S1 20.06 one each	Syunik	2	2	1	A2-A3	6/30/23?	25	7/6/23	587	6089	46	202	26	11	108	39	1.88	0.24	0.10	0.36	0.13	0.05	0.53	0.19
1787	TR.2 B0 H2 S2 02.07.23 one each	Gegham 1	2	2	2	B0	7/2/23	26	7/6/23	660	3805	33	212	11	26	73	55	2.89	0.16	0.35	0.75	0.05	0.12	0.35	0.26
1788	TR.2 B0 H2 S2 02.07.23 one each	Gegham 1	2	2	2	B0	7/2/23	27	7/6/23	708	3951	37	215	16	25	72	57	3.00	0.22	0.34	0.79	0.07	0.11	0.33	0.26
1789	TR.2 B0 H2 S2 02.07.23 one each	Syunik	2	2	2	B0	7/2/23	28	7/6/23	493	5280	36	185	20	11	104	37	1.78	0.19	0.11	0.36	0.11	0.06	0.56	0.20
1790	TR.2 B0 H2 S2 02.07.23 one each	Kelbadjar	2	2	2	B0	7/2/23	29	7/6/23	481	4680	46	152	16	14	109	35	1.39	0.14	0.13	0.32	0.10	0.09	0.72	0.23
1791	TR.2 B0 H2 S2 02.07.23 one each	Gegham 1	2	2	2	B0	7/2/23	30	7/6/23	668	3733	32	205	11	24	72	53	2.87	0.16	0.33	0.73	0.06	0.12	0.35	0.26
1792	TR.2 B0 H2 S2 02.07.23 one each	Gegham 1	2	2	2	B0	7/2/23	31	7/6/23	640	3704	33	205	14	23	71	53	2.91	0.19	0.32	0.74	0.07	0.11	0.34	0.26
1793	TR.2 B0 H2 S2 02.07.23 one each	Gegham 1	2	2	2	B0	7/2/23	32	7/6/23	643	3547	33	208	13	27	75	54	2.76	0.17	0.36	0.71	0.06	0.13	0.36	0.26
1794	TR.2 B0 H2 S2 02.07.23 one each	Kelbadjar	2	2	2	B0	7/2/23	33	7/6/23	607	6428	48	183	19	11	113	36	1.62	0.16	0.09	0.32	0.10	0.06	0.62	0.20
1795	TR.2 B0 H2 S2 02.07.23 one each	Gegham 1	2	2	2	B0	7/2/23	34	7/6/23	675	3692	34	207	14	25	69	54	3.01	0.20	0.36	0.78	0.07	0.12	0.33	0.26
1796	TR.2 B0 H2 S2 02.07.23 one each	Syunik	2	2	2	B0	7/2/23	35	7/6/23	458	4931	40	184	22	11	110	35	1.67	0.20	0.10	0.31	0.12	0.06	0.60	0.19
1797	TR.2 B0 H2 S2 02.07.23 one each	Kelbadjar	2	2	2	B0	7/2/23	36	7/6/23	528	5258	42	164	17	12	121	39	1.36	0.14	0.10	0.32	0.10	0.08	0.73	0.24
1798	TR.2 B0 H2 S2 02.07.23 one each	Gegham 1	2	2	2	B0	7/2/23	37	7/6/23	688	3849	35	208	14	25	74	55	2.80	0.18	0.33	0.74	0.06	0.12	0.36	0.27
1799	TR.2 B0 H2 S2 02.07.23 one each	Gegham 1	2	2	2	B0	7/2/23	38	7/6/23	660	3772	34	214	13	26	73	55	2.95	0.17	0.35	0.76	0.06	0.12	0.34	0.26
1800	TR.2 B0 H2 S2 02.07.23 one each	Gegham 1	2	2	2	B0	7/2/23	39	7/6/23	715	3873	32	216	18	25	72	53	3.02	0.24	0.34	0.73	0.08	0.11	0.33	0.24
1801	TR.2 B0 H2 S2 02.07.23 one each	Kelbadjar	2	2	2	B0	7/2/23	40	7/6/23	509	5321	39	165	16	10	106	33	1.57	0.15	0.09	0.32	0.09	0.06	0.64	0.20
1802	TR.2 B0 H2 S2 02.07.23 one each	Syunik	2	2	2	B0	7/2/23	41	7/6/23	464	4993	39	187	24	11	108	37	1.74	0.22	0.11	0.35	0.13	0.06	0.58	0.20
1803	TR.2 B0 H2 S2 02.07.23 one each	Kelbadjar	2	2	2	B0	7/2/23	42	7/6/23	552	5863	44	173	20	12	121	39	1.43	0.16	0.10	0.32	0.11	0.07	0.70	0.22
1804	TR.2 B0 H2 S2 02.07.23 one each	Gegham 1	2	2	2	B0	7/2/23	43	7/6/23	721	3947	32	220	11	25	73	55	3.03	0.16	0.34	0.76	0.05	0.11	0.33	0.25
1805	TR.2 B0 H2 S2 02.07.23 one each	Gegham 1	2	2	2	B0	7/2/23	44	7/6/23	747	4138	32	223	13	27	75	58	2.96	0.17	0.35	0.77	0.06	0.12	0.34	0.26
1806	TR.2 B0 H2 S2 02.07.23 one each	Gegham 1	2	2	2	B0	7/2/23	45	7/6/23	696	3839	35	210	14	26	75	54	2.79	0.18	0.34	0.71	0.06	0.12	0.36	0.26
1807	TR.2 B0 H2 S2 02.07.23 one each	Gegham 1	2	2	2	B0	7/2/23	46	7/6/23	753	4268	38	233	16	25	74	54	3.14	0.21	0.33	0.72	0.07	0.11	0.32	0.23
1808	TR.2 B0 H2 S2 02.07.23 one each	Syunik	2	2	2	B0	7/2/23	47	7/6/23	666	6919	51	196	20	10	134	36	1.46	0.15	0.07	0.27	0.10	0.05	0.69	0.18
1809	TR.2 B0 H2 S2 02.07.23 one each	Gegham 1	2	2	2	B0	7/2/23	48	7/6/23	698	4028	34	222	14	26	75	54	2.94	0.18	0.34	0.71	0.06	0.12	0.34	0.24
1810	TR.2 B0 H2 S2 02.07.23 one each	Arteni	2	2	2	B0	7/2/23	49	7/6/23	601	5251	45	131	44	25	93	26	1.41	0.47	0.26	0.28	0.33	0.19	0.71	0.20
1811	TR.2 B0 H2 S2 02.07.23 one each	Gegham 1	2	2	2	B0	7/2/23	50	7/6/23	819	4517	42	235	15	26	77	57	3.05	0.19	0.33	0.73	0.06	0.11	0.33	0.24
1812	TR.2 B0 H2 S2 02.07.23 one each	Syunik	2	2	2	B0	7/2/23	51	7/6/23	564	4620	41	198	11	11	91	40	2.17	0.13	0.13	0.44	0.06	0.06	0.46	0.20
1813	TR.2 B0 H2 S2 02.07.23 one each	Gegham 1	2	2	2	B0	7/2/23	52	7/6/23	756	4022	35	226	13	27	73	57	3.07	0.17	0.36	0.77	0.06	0.12	0.33	0.25
1814	TR.2 B0 H2 S2 02.07.23 one each	Gegham 1	2	2	2	B0	7/2/23	53	7/6/23	1093	5819	51	271	15	27	78	59	3.47	0.19	0.34	0.76	0.05	0.10	0.29	0.22
1815	TR.2 B0 H2 S2 02.07.23 one each	Gegham 1	2	2	2	B0	7/2/23	54	7/6/23	792	4187	42	226	15	27	75	57	2.99	0.19	0.36	0.75	0.06	0.12	0.33	0.25
1816	TR.2 B0 H2 S2 02.07.23 one each	Kelbadjar	2	2	2	B0	7/2/23	55	7/6/23	523	5329	45	166	20	10	107	33	1.56	0.18	0.09	0.31	0.12	0.06	0.64	0.20
1817	T2 B1 Hor.3 Sp.2 one each	Gegham 1	2	3	2	B1	-	59	7/6/23	671	3723	35	203	11	25	73	51	2.77	0.16	0.34	0.70	0.06	0.12	0.36	0.25
1818	T2 B1 Hor.3 Sp.2 one each	Gegham 1	2	3	2	B1	-	60	7/6/23	712	3971	35	213	14	27	71	55	3.02	0.19	0.38	0.78	0.06	0.12	0.33	0.26
1819	T2 B1 Hor.3 Sp.2 one each	Syunik	2	3	2	B1	-	61	7/6/23	545	5420	40	190	22	11	101	37	1.88	0.21	0.11	0.37	0.11	0.06	0.53	0.20
1820	T2 B1 Hor.3 Sp.2 one each	Gegham 1	2	3	2	B1	-	62	7/6/23	678	4189	38	209	15	24	69	53	3.04	0.21	0.34	0.76	0.07	0.11	0.33	0.25
1821	T2 B1 Hor.3 Sp.2 one each	Syunik	2	3	2	B1	-	63	7/6/23	520	5696	39	185	24	12	112	35	1.65	0.21	0.11	0.31	0.13	0.07	0.61	0.19
1822	T2 B1 Hor.3 Sp.2 one each	Gegham 1	2	3	2	B1	-	64	7/6/23	708	4009	34	220	14	27	73	55	2.99	0.18	0.37	0.75	0.06	0.12	0.33	0.25
1823	T2 B1 Hor.3 Sp.2 one each	Syunik	2	3	2	B1	-	65	7/6/23	556	5989	46	190	26	10	107	35	1.78	0.24	0.09	0.33	0.13	0.05	0.56	0.18
1824	T2 B1 Hor.3 Sp.2 one each	Arteni	2	3	2	B1	-	66	7/6/23	758	4745	47	154	23	31	85	40	1.81	0.27	0.37	0.47	0.15	0.20	0.55	0.26
1825	T2 B1 Hor.3 Sp.2 one each	Gegham 1	2	3	2	B1	-	67	7/6/23	869	5071	43	252	17	27	79	58	3.18	0.21	0.34	0.73	0.07	0.11	0.31	0.23
1826	T2 B1 Hor.3 Sp.2 one each	Kelbadjar	2	3	2	B1	-	68	7/6/23	584	5944	43	175	18	12	117	35	1.50	0.15	0.11	0.30	0.10	0.07	0.67	0.20
1827	T.2 C1 Hor.3 Sp.1 one each	Gegham 1	2	3	1	C1	-	69	7/6/23	599	3684	35	204	13	25	72	53	2.85	0.17	0.34	0.73	0.06	0.12	0.35	0.26
1828	T.2 C1 Hor.3 Sp.1 one each	Syunik	2	3	1	C1	-	70	7/6/23	596	5857	46	190	24	12	108	37	1.75	0.22	0.11	0.34	0.12	0.07	0.57	0.20
1829	T.2 B0 Hor.3 Sp.2 one each	Gegham 1	2	3	2	B0	-	71	7/6/23	707	4096	37	219	15	25	75	59	2.90	0.19	0.33	0.79	0.07	0.11	0.34	0.27
1830	T.2 B0 Hor.3 Sp.2 one each	Gegham 1	2	3	2	B0	-	72	7/6/23	711	4050	35	218	16	26	73	57	2.97	0.21	0.35	0.77	0.07	0.12	0.34	0.26
1831	T.2 B0 Hor.3 Sp.2 one each	Gegham 1	2	3	2	B0	-	73	7/6/23	764	4362	40	232	13	22	74	57	3.11	0.17	0.29	0.76	0.05	0.09	0.32	0.24
1832	T.2 B0 Hor.3 Sp.2 one each	Gegham 1	2	3	2	B0	-	74	7/6/23	713	3900	36	214	14	27	76	55	2.80	0.18	0.35	0.72	0.06	0.12	0.36	0.26

1833	Tr-2 Unit-A0 H3 Sp1 one each	Gegham 1	2	3	1	A0	-	75	7/6/23	676	3736	32	203	13	24	71	54	2.88	0.18	0.34	0.76	0.06	0.12	0.35	0.26
1834	Tr-2 Unit-A0 H3 Sp1 one each	Gegham 1	2	3	1	A0	-	76	7/6/23	731	4178	35	221	14	26	72	57	3.08	0.19	0.36	0.79	0.06	0.12	0.32	0.26
1835	Tr-2 Unit-A0 H3 Sp1 one each	Hatis	2	3	1	A0	-	77	7/6/23	529	7420	35	114	130	17	102	22	1.12	1.27	0.17	0.22	1.14	0.15	0.89	0.20
1836	Tr-2 Unit-A0 H3 Sp1 one each	Gegham 1	2	3	1	A0	-	78	7/6/23	660	3757	33	214	15	23	71	54	3.03	0.21	0.32	0.76	0.07	0.11	0.33	0.25
1837	Tr-2 Unit-A0 H3 Sp1 one each	Gegham 1	2	3	1	A0	-	79	7/6/23	751	4159	39	228	13	25	76	57	2.98	0.16	0.32	0.74	0.05	0.11	0.34	0.25
1838	Tr-2 Unit-A0 H3 Sp1 one each	Kelbadjar	2	3	1	A0	-	80	7/6/23	641	6508	52	182	18	11	126	36	1.44	0.14	0.08	0.29	0.10	0.06	0.69	0.20
1839	Tr-2 Unit-A0 H3 Sp1 one each	Kelbadjar	2	3	1	A0	-	81	7/6/23	564	5638	44	173	18	11	113	35	1.53	0.15	0.10	0.31	0.10	0.07	0.65	0.20
1840	Tr-2 Unit-A0 H3 Sp1 one each	Gegham 1	2	3	1	A0	-	82	7/6/23	597	3665	44	202	23	24	72	54	2.83	0.31	0.33	0.75	0.11	0.12	0.35	0.27
1841	Tr-2 Unit-A0 H3 Sp1 one each	Gegham 1	2	3	1	A0	-	83	7/6/23	1009	5600	46	254	15	25	78	54	3.25	0.19	0.32	0.69	0.06	0.10	0.31	0.21
1842	Tr-2 Unit-A0 H3 Sp1 one each	Gegham 1	2	3	1	A0	-	84	7/6/23	1016	5333	51	264	15	25	76	54	3.45	0.19	0.32	0.71	0.06	0.09	0.29	0.20
1843	T2 B0 Bor.3 Sp.1 one each	Gegham 1	2	3	1	B0	-	85	7/6/23	670	3839	33	205	14	26	73	58	2.83	0.19	0.35	0.80	0.07	0.12	0.35	0.28
1844	T2 B0 Bor.3 Sp.1 one each	Kelbadjar	2	3	1	B0	-	86	7/6/23	526	5410	38	162	17	11	113	36	1.43	0.15	0.10	0.32	0.10	0.07	0.70	0.22
1845	T2 B0 Bor.3 Sp.1 one each	Gegham 1	2	3	1	B0	-	87	7/6/23	728	4056	35	222	14	28	75	55	2.94	0.18	0.38	0.73	0.06	0.13	0.34	0.25
1846	T2 B0 Bor.3 Sp.1 one each	Gegham 1	2	3	1	B0	-	88	7/6/23	898	5059	45	252	16	27	74	57	3.38	0.21	0.36	0.76	0.06	0.11	0.30	0.22
1847	T2 B0 Hor 2 Sp.2 one each	Gegham 1	2	2	2	B0	-	89	7/6/23	726	4089	32	217	13	25	72	54	3.03	0.17	0.34	0.75	0.06	0.11	0.33	0.25
1848	T2 B0 Hor 2 Sp.2 one each	Gegham 1	2	2	2	B0	-	90	7/6/23	685	3782	36	211	15	27	71	58	2.99	0.21	0.38	0.82	0.07	0.13	0.33	0.27
1849	T2 B0 Hor 2 Sp.2 one each	Kelbadjar	2	2	2	B0	-	91	7/6/23	602	6035	48	179	17	11	118	37	1.52	0.14	0.09	0.32	0.09	0.06	0.66	0.21
1850	T2 B0 Hor 2 Sp.2 one each	Kelbadjar	2	2	2	B0	-	92	7/6/23	651	6442	55	182	19	11	127	35	1.43	0.15	0.09	0.27	0.10	0.06	0.70	0.19
1851	T2 C0 Hor 3 Sp.1 one each	Kelbadjar	2	3	1	C0	-	93	7/6/23	514	5538	45	171	18	11	111	36	1.54	0.16	0.10	0.32	0.10	0.07	0.65	0.21
1852	T2 C0 Hor 3 Sp.1 one each	Gegham 1	2	3	1	C0	-	94	7/6/23	663	3618	34	205	14	25	73	54	2.83	0.19	0.34	0.74	0.07	0.12	0.35	0.26
1853	T2 C0 Hor 3 Sp.1 one each	Syunik	2	3	1	C0	-	95	7/6/23	602	6684	45	203	27	10	111	35	1.83	0.24	0.09	0.31	0.13	0.05	0.55	0.17
1854	T2 C0 Hor.2 Sp.2 one each	Kelbadjar	2	2	2	C0	-	96	7/6/23	509	5133	39	160	18	10	114	33	1.40	0.15	0.08	0.29	0.11	0.06	0.72	0.21
1855	T2 C0 Hor.2 Sp.2 one each	Gegham 1	2	2	2	C0	-	97	7/6/23	733	4040	38	218	11	26	74	58	2.93	0.15	0.34	0.78	0.05	0.12	0.34	0.27
1856	T2 C0 Hor.2 Sp.2 one each	Gegham 1	2	2	2	C0	-	98	7/6/23	762	4368	39	232	16	27	73	57	3.19	0.21	0.37	0.78	0.07	0.11	0.31	0.24
1857	Tr-2 Unit-A0 H3 Sp2 05.07 one each	Gegham 1	2	3	2	A0	7/5/23	99	7/6/23	676	3916	32	216	14	27	69	54	3.14	0.20	0.39	0.78	0.06	0.12	0.32	0.25
1858	Tr-2 Unit-A0 H3 Sp2 05.07 one each	Gegham 1	2	3	2	A0	7/5/23	100	7/6/23	722	3928	35	215	20	28	73	55	2.96	0.27	0.39	0.76	0.09	0.13	0.34	0.26
1859	Tr-2 Unit-A1 H3 S2 one each	Arteni	2	3	2	A1	-	101	7/6/23	696	4320	44	141	22	31	83	35	1.70	0.26	0.38	0.42	0.15	0.22	0.59	0.25
1860	Tr-2 Unit-A1 H3 S2 one each	Syunik	2	3	2	A1	-	102	7/6/23	562	5748	39	203	18	11	104	36	1.96	0.17	0.10	0.35	0.09	0.05	0.51	0.18
1861	Tr-2 Unit-A1 H3 S2 one each	Gegham 1	2	3	2	A1	-	103	7/6/23	688	3947	32	213	13	25	72	55	2.98	0.17	0.34	0.77	0.06	0.12	0.34	0.26
1862	Tr-2 Unit-A1 H3 S2 one each	Gegham 1	2	3	2	A1	-	104	7/6/23	738	4184	35	217	13	25	76	55	2.84	0.16	0.32	0.72	0.06	0.11	0.35	0.25
1863	Tr-2 Unit-A1 H3 S2 one each	Gegham 1	2	3	2	A1	-	105	7/6/23	675	3863	36	213	14	25	71	55	3.02	0.19	0.35	0.78	0.06	0.12	0.33	0.26
1864	Tr-2 Unit-A1 H3 S2 one each	Gegham 1	2	3	2	A1	-	106	7/6/23	682	3991	34	205	15	26	73	53	2.79	0.20	0.35	0.71	0.07	0.12	0.36	0.26
1865	Tr-2 Unit-A1 H3 S2 one each	Gegham 1	2	3	2	A1	-	107	7/6/23	824	4288	46	240	18	28	73	57	3.27	0.24	0.39	0.77	0.07	0.12	0.31	0.24
1866	Tr-2 Unit-A1 H3 S2 one each	Arteni	2	3	2	A1	-	108	7/6/23	693	4405	43	150	21	28	83	33	1.81	0.25	0.34	0.40	0.14	0.19	0.55	0.22
1867	Tr-2 Unit-A1 H3 S2 one each	Gegham 1	2	3	2	A1	-	109	7/6/23	818	4689	42	237	15	26	71	54	3.36	0.21	0.36	0.76	0.06	0.11	0.30	0.23
1868	Tr-2 Unit-C1 H3 Sp2 05.07 one each	Gegham 1	2	3	2	C1	7/5/23	110	7/6/23	687	3958	35	212	11	25	73	54	2.92	0.16	0.34	0.74	0.05	0.12	0.34	0.25
1869	Tr-2 Unit-C1 H3 Sp2 05.07 one each	Kelbadjar	2	3	2	C1	7/5/23	112	7/6/23	533	5572	43	165	17	10	113	35	1.46	0.15	0.08	0.31	0.10	0.06	0.68	0.21
1870	Tr-2 Unit-C1 H3 Sp2 05.07 one each	Gegham 1	2	3	2	C1	7/5/23	113	7/6/23	685	3574	33	210	13	26	72	57	2.94	0.17	0.36	0.79	0.06	0.12	0.34	0.27
1871	Tr-2 Unit-C1 H3 Sp2 05.07 one each	Gegham 1	2	3	2	C1	7/5/23	114	7/6/23	739	3914	34	212	14	27	70	54	3.04	0.19	0.38	0.77	0.06	0.13	0.33	0.25
1872	Tr-2 Unit-C1 H3 Sp2 05.07 one each	Hatis	2	3	2	C1	7/5/23	115	7/6/23	506	6695	38	120	120	18	100	22	1.20	1.20	0.18	0.22	1.00	0.15	0.83	0.19
1873	Tr-2 Unit-C1 H3 Sp2 05.07 one each	Gegham 1	2	3	2	C1	7/5/23	116	7/6/23	686	3986	34	216	13	23	74	54	2.90	0.17	0.31	0.72	0.06	0.11	0.34	0.25
1874	Tr-2 Unit-C1 H3 Sp2 05.07 one each	Gegham 1	2	3	2	C1	7/5/23	117	7/6/23	729	4103	37	214	15	27	73	55	2.91	0.20	0.36	0.75	0.07	0.12	0.34	0.26
1875	Tr-2 Unit-C1 H3 Sp2 05.07 one each	Gegham 1	2	3	2	C1	7/5/23	118	7/6/23	769	4393	35	218	14	27	73	53	2.97	0.18	0.36	0.71	0.06	0.12	0.34	0.24
1876	Tr-2 Unit-C1 H3 Sp2 05.07 one each	Syunik	2	3	2	C1	7/5/23	119	7/6/23	614	4486	39	221	9	11	93	47	2.37	0.10	0.11	0.50	0.04	0.05	0.42	0.21
1877	Tr-2 Unit-C1 H3 Sp2 05.07 one each	Gegham 1	2	3	2	C1	7/5/23	120	7/6/23	617	3901	32	166	15	25	68	48	2.45	0.21	0.36	0.71	0.09	0.15	0.41	0.29
1878	Tr-2 Unit-C1 H3 Sp2 05.07 one each	Gegham 1	2	3	2	C1	7/5/23	121	7/6/23	767	4045	35	219	14	23	69	53	3.18	0.20	0.33	0.76	0.06	0.10	0.31	0.24
1879	Tr-2 Unit-C1 H3 Sp2 05.07 one each	Gegham 1	2	3	2	C1	7/5/23	122	7/6/23	817	4479	41	237	14	26	75	58	3.15	0.18	0.34	0.77	0.06	0.11	0.32	0.24
1880	Tr-2 Unit-C1 H3 Sp2 05.07 one each	Gegham 1	2	3	2	C1	7/5/23	123	7/6/23	739	4226	39	226	15	25	75	55	2.99	0.19	0.33	0.73	0.06	0.11	0.33	0.24
1881	Tr-2 Unit-C1 H3 Sp2 05.07 one each	Gegham 1	2	3	2	C1	7/5/23	124	7/6/23	701	4103	33	221	13	27	74	55	2.97	0.17	0.37	0.74	0.06	0.12	0.34	0.25
1882	Tr-2 Unit-C1 H3 Sp2 05.07 one each	Gegham 1	2	3	2	C1	7/5/23	125	7/6/23	754	4836	37	225	19	28	76	58	2.94	0.24	0.37	0.76	0.08	0.13	0.34	0.26
1883	Tr-2 Unit-C1 H3 Sp2 05.07 one each	Gegham 1	2	3	2	C1	7/5/23	126	7/6/23	729	4150	36	217	13	26	72	54	3.03	0.17	0.36	0.75	0.06	0.12	0.33	0.25

1884	Tr-2 Unit-C1 H3 Sp2 05.07 one each	Gegham 1	2	3	2	C1	7/5/23	127	7/6/23	791	4119	39	229	15	26	71	57	3.24	0.21	0.36	0.80	0.06	0.11	0.31	0.25
1885	Tr-2 Unit-C1 H3 Sp2 05.07 one each	Gegham 1	2	3	2	C1	7/5/23	129	7/6/23	820	4231	38	232	14	23	76	54	3.03	0.18	0.30	0.71	0.06	0.10	0.33	0.23
1886	Tr-2 Unit-C1 H3 Sp2 05.07 one each	Gegham 1	2	3	2	C1	7/5/23	130	7/6/23	814	4425	46	233	15	27	76	57	3.05	0.19	0.35	0.74	0.06	0.11	0.33	0.24
1887	Tr-2 Unit-C1 H3 Sp2 05.07 one each	Gegham 1	2	3	2	C1	7/5/23	131	7/6/23	777	4402	41	230	14	27	73	55	3.12	0.18	0.36	0.75	0.06	0.12	0.32	0.24
1888	Tr-2 Unit-C1 H3 Sp2 05.07 one each	Arteni	2	3	2	C1	7/5/23	132	7/6/23	756	5868	51	150	41	27	94	31	1.59	0.43	0.28	0.32	0.27	0.18	0.63	0.20
1889	Tr-2 Unit-A1 H3 Sp1 one each	Gegham 1	2	3	1	A1	-	133	7/6/23	637	3654	29	200	13	26	70	50	2.88	0.18	0.37	0.71	0.06	0.13	0.35	0.25
1890	Tr-2 Unit-A1 H3 Sp1 one each	Gegham 1	2	3	1	A1	-	135	7/6/23	692	4129	35	223	14	25	73	55	3.03	0.18	0.34	0.75	0.06	0.11	0.33	0.25
1891	Tr-2 Unit-A1 H3 Sp1 one each	Gegham 1	2	3	1	A1	-	136	7/6/23	616	4027	32	199	17	25	70	55	2.86	0.24	0.35	0.79	0.08	0.12	0.35	0.28
1892	Tr-2 Unit-A1 H3 Sp1 one each	Arteni	2	3	1	A1	-	137	7/6/23	651	5378	46	140	43	27	91	28	1.53	0.47	0.29	0.30	0.30	0.19	0.65	0.20
1893	Tr-2 Unit-A1 H3 Sp1 one each	Gegham 1	2	3	1	A1	-	138	7/6/23	752	3994	34	223	14	26	73	54	3.03	0.18	0.35	0.73	0.06	0.11	0.33	0.24
1894	Tr-2 Unit-A1 H3 Sp1 one each	Gegham 1	2	3	1	A1	-	139	7/6/23	817	4469	42	244	15	23	74	55	3.28	0.19	0.31	0.74	0.06	0.09	0.30	0.23
1895	Tr-2 Unit-A1 H3 Sp1 one each	Arteni	2	3	1	A1	-	140	7/6/23	601	5234	37	126	50	27	91	31	1.39	0.55	0.30	0.34	0.39	0.22	0.72	0.24
1896	Tr-2 Unit-A1 H3 Sp1 one each	Gegham 1	2	3	1	A1	-	141	7/6/23	706	4062	42	208	14	24	75	54	2.76	0.18	0.31	0.71	0.06	0.11	0.36	0.26
1897	Tr-2 Unit-A1 H3 Sp1 one each	Gegham 1	2	3	1	A1	-	142	7/6/23	782	4390	36	233	14	25	73	57	3.20	0.19	0.34	0.78	0.06	0.11	0.31	0.24
1898	Tr-2 Unit-A1 H3 Sp1 one each	Gegham 1	2	3	1	A1	-	143	7/6/23	681	3894	35	212	14	25	71	53	3.00	0.19	0.35	0.74	0.06	0.12	0.33	0.25
1899	Tr-2 Unit-A1 H3 Sp1 one each	Arteni	2	3	1	A1	-	144	7/6/23	833	4912	48	163	23	32	85	36	1.92	0.27	0.38	0.42	0.14	0.20	0.52	0.22
1900	Tr-2 Unit-A1 H3 Sp1 one each	Arteni	2	3	1	A1	-	145	7/6/23	690	5115	43	140	40	26	87	26	1.62	0.46	0.29	0.30	0.28	0.18	0.62	0.19
1901	Tr-2 Unit-A1 H3 Sp1 one each	Gegham 1	2	3	1	A1	-	146	7/6/23	1024	5499	46	266	16	26	73	55	3.66	0.21	0.35	0.76	0.06	0.10	0.27	0.21
1902	Tr-2 Unit-A1 H3 Sp1 one each	Kelbadjar	2	3	1	A1	-	147	7/6/23	586	5633	47	166	17	12	111	36	1.50	0.15	0.11	0.32	0.10	0.07	0.67	0.22
1903	TR2 B1 Hor.3 Sp.1 one each	Arteni	2	3	1	B1	-	149	7/6/23	590	4756	43	130	38	25	89	31	1.47	0.42	0.28	0.34	0.29	0.19	0.68	0.23
1904	TR2 B1 Hor.3 Sp.1 one each	Gegham 1	2	3	1	B1	-	150	7/6/23	707	3752	34	213	14	23	71	53	3.02	0.19	0.32	0.74	0.06	0.11	0.33	0.25
1905	TR2 B1 Hor.3 Sp.1 one each	Gegham 1	2	3	1	B1	-	151	7/6/23	716	3953	31	206	14	24	70	53	2.96	0.19	0.34	0.75	0.07	0.11	0.34	0.25
1906	TR2 B1 Hor.3 Sp.1 one each	Gegham 1	2	3	1	B1	-	152	7/6/23	746	4075	35	221	13	25	73	58	3.00	0.17	0.34	0.79	0.06	0.11	0.33	0.26
1907	TR2 B1 Hor.3 Sp.1 one each	Gegham 1	2	3	1	B1	-	153	7/6/23	647	3864	35	202	14	25	72	51	2.83	0.19	0.34	0.71	0.07	0.12	0.35	0.25
1908	TR2 B1 Hor.3 Sp.1 one each	Gegham 1	2	3	1	B1	-	154	7/6/23	723	3953	35	216	13	25	72	54	3.02	0.17	0.34	0.75	0.06	0.11	0.33	0.25
1909	TR2 B1 Hor.3 Sp.1 one each	Arteni	2	3	1	B1	-	155	7/6/23	576	4314	41	133	30	27	85	29	1.57	0.35	0.31	0.34	0.22	0.20	0.64	0.22
1910	TR2 B1 Hor.3 Sp.1 one each	Gegham 1	2	3	1	B1	-	156	7/6/23	695	4097	34	207	14	23	75	53	2.75	0.18	0.30	0.70	0.07	0.11	0.36	0.25
1911	TR2 B1 Hor.3 Sp.1 one each	Gegham 1	2	3	1	B1	-	157	7/6/23	656	3587	36	204	10	24	71	55	2.89	0.15	0.34	0.78	0.05	0.12	0.35	0.27
1912	TR2 B1 Hor.3 Sp.1 one each	Gegham 1	2	3	1	B1	-	158	7/6/23	713	4114	36	225	13	26	74	55	3.02	0.17	0.34	0.74	0.06	0.11	0.33	0.25
1913	TR2 B1 Hor.3 Sp.1 one each	Gegham 1	2	3	1	B1	-	159	7/6/23	820	4564	38	244	15	27	77	55	3.16	0.19	0.34	0.71	0.06	0.11	0.32	0.23
1914	TR2 B1 Hor.3 Sp.1 one each	Gegham 1	2	3	1	B1	-	160	7/6/23	827	4455	41	241	14	24	72	58	3.37	0.19	0.33	0.81	0.06	0.10	0.30	0.24
1915	TR2 B1 Hor.3 Sp.1 one each	Gegham 1	2	3	1	B1	-	161	7/6/23	691	3883	36	214	13	25	73	54	2.95	0.17	0.34	0.74	0.06	0.12	0.34	0.25
1916	TR2 B1 Hor.3 Sp.1 one each	Gegham 1	2	3	1	B1	-	162	7/6/23	829	4734	45	238	17	27	74	55	3.20	0.22	0.37	0.74	0.07	0.12	0.31	0.23
1917	TR.2 A0 H2 S2 03.07.23 one each	Gegham 1	2	2	2	A0	7/3/23	163	7/6/23	721	4094	35	220	13	26	75	58	2.92	0.17	0.34	0.77	0.06	0.12	0.34	0.26
1918	TR.2 A0 H2 S2 03.07.23 one each	Gegham 1	2	2	2	A0	7/3/23	164	7/6/23	652	3876	33	208	18	26	73	57	2.87	0.24	0.35	0.78	0.08	0.12	0.35	0.27
1919	TR.2 A0 H2 S2 03.07.23 one each	Syunik	2	2	2	A0	7/3/23	165	7/6/23	464	5390	37	184	22	11	103	33	1.79	0.21	0.11	0.32	0.12	0.06	0.56	0.18
1920	TR.2 A0 H2 S2 03.07.23 one each	Gegham 1	2	2	2	A0	7/3/23	166	7/6/23	636	3648	34	205	24	26	72	51	2.87	0.33	0.36	0.71	0.11	0.12	0.35	0.25
1921	TR.2 A0 H2 S2 03.07.23 one each	Tsaghkunyats	2	2	2	A0	7/3/23	168	7/6/23	535	6882	33	105	193	9	111	24	0.95	1.73	0.08	0.21	1.83	0.08	1.06	0.23
1922	TR.2 A0 H2 S2 03.07.23 one each	Gegham 1	2	2	2	A0	7/3/23	169	7/6/23	688	3817	34	210	13	25	73	53	2.90	0.17	0.34	0.72	0.06	0.12	0.35	0.25
1923	TR.2 A0 H2 S2 03.07.23 one each	Gegham 1	2	2	2	A0	7/3/23	170	7/6/23	686	3860	29	210	13	27	74	57	2.82	0.17	0.36	0.76	0.06	0.13	0.35	0.27
1924	TR.2 A0 H2 S2 03.07.23 one each	Gegham 1	2	2	2	A0	7/3/23	171	7/6/23	638	4226	37	209	21	23	73	50	2.88	0.28	0.31	0.69	0.10	0.11	0.35	0.24
1925	TR.2 A0 H2 S2 03.07.23 one each	Syunik	2	2	2	A0	7/3/23	172	7/6/23	506	5457	35	180	24	11	108	35	1.67	0.22	0.11	0.32	0.13	0.06	0.60	0.19
1926	TR.2 A0 H2 S2 03.07.23 one each	Gegham 1	2	2	2	A0	7/3/23	173	7/6/23	698	3988	42	225	24	25	73	55	3.10	0.32	0.34	0.76	0.10	0.11	0.32	0.25
1927	TR.2 A0 H2 S2 03.07.23 one each	Kelbadjar	2	2	2	A0	7/3/23	174	7/6/23	572	5466	40	164	18	11	112	36	1.47	0.16	0.10	0.32	0.11	0.07	0.68	0.22
1928	TR.2 A0 H2 S2 03.07.23 one each	Kelbadjar	2	2	2	A0	7/3/23	175	7/6/23	607	5889	44	181	19	10	124	35	1.46	0.15	0.08	0.28	0.10	0.05	0.68	0.19
1929	TR.2 A0 H2 S2 03.07.23 one each	Kelbadjar	2	2	2	A0	7/3/23	176	7/6/23	554	4784	37	180	15	16	99	43	1.82	0.15	0.16	0.43	0.08	0.09	0.55	0.24
1930	TR.2 A0 H2 S2 03.07.23 one each	Kelbadjar	2	2	2	A0	7/3/23	177	7/6/23	513	5344	41	167	16	11	115	37	1.45	0.13	0.09	0.33	0.09	0.06	0.69	0.22
1931	TR.2 A0 H2 S2 03.07.23 one each	Arteni	2	2	2	A0	7/3/23	178	7/6/23	544	4532	38	128	41	27	88	26	1.46	0.46	0.30	0.30	0.32	0.21	0.69	0.21
1932	TR.2 A0 H2 S2 03.07.23 one each	Gegham 1	2	2	2	A0	7/3/23	179	7/6/23	818	4414	37	228	14	27	76	59	2.98	0.18	0.36	0.78	0.06	0.12	0.34	0.26
1933	TR.2 A0 H2 S2 03.07.23 one each	Kelbadjar	2	2	2	A0	7/3/23	180	7/6/23	614	6151	52	180	19	8	111	36	1.62	0.17	0.07	0.32	0.10	0.04	0.62	0.20
1934	TR.2 A0 H2 S2 03.07.23 one each	Gegham 1	2	2	2	A0	7/3/23	181	7/6/23	864	4519	43	235	15	25	74	54	3.16	0.19	0.33	0.72	0.06	0.10	0.32	0.23

1935	TR.2 A0 H2 S2 03.07.23 one each	Gegham 1	2	2	2	A0	7/3/23	182	7/6/23	1117	5919	55	272	18	25	76	59	3.57	0.23	0.32	0.78	0.06	0.09	0.28	0.22
1936	TR.2 A0 H2 S2 03.07.23 one each	Syunik	2	2	2	A0	7/3/23	183	7/6/23	496	5255	38	178	26	12	106	33	1.69	0.24	0.12	0.32	0.14	0.07	0.59	0.19
1937	TR.2 A0 H2 S2 03.07.23 one each	Gegham 1	2	2	2	A0	7/3/23	184	7/6/23	859	4778	40	240	14	24	82	58	2.93	0.16	0.29	0.71	0.06	0.10	0.34	0.24
1938	TR.2 A0 H2 S2 03.07.23 one each	Gegham 1	2	2	2	A0	7/3/23	185	7/6/23	727	4052	43	221	19	27	74	57	2.97	0.25	0.36	0.76	0.08	0.12	0.34	0.26
1939	TR.2 A1 H1 S2 03.07.23 one each	Kelbadjar	2	1	2	A1	7/3/23	186	7/6/23	514	5587	42	161	23	8	108	37	1.48	0.21	0.07	0.34	0.14	0.05	0.68	0.23
1940	TR.2 A1 H1 S2 03.07.23 one each	Gegham 1	2	1	2	A1	7/3/23	187	7/6/23	757	4174	33	226	13	26	75	58	2.99	0.17	0.34	0.77	0.06	0.11	0.33	0.26
1941	TR.2 A1 H1 S2 03.07.23 one each	Gegham 1	2	1	2	A1	7/3/23	188	7/6/23	736	4077	35	217	11	24	72	54	3.03	0.16	0.33	0.75	0.05	0.11	0.33	0.25
1942	TR.2 A1 H1 S2 03.07.23 one each	Kelbadjar	2	1	2	A1	7/3/23	189	7/6/23	536	5452	42	164	16	10	113	35	1.45	0.14	0.08	0.31	0.09	0.06	0.69	0.21
1943	TR.2 A1 H1 S2 03.07.23 one each	Kelbadjar	2	1	2	A1	7/3/23	190	7/6/23	519	5255	40	161	18	11	109	35	1.47	0.16	0.10	0.32	0.11	0.07	0.68	0.22
1944	TR.2 A1 H1 S2 03.07.23 one each	Kelbadjar	2	1	2	A1	7/3/23	191	7/6/23	537	5565	46	169	19	9	117	35	1.45	0.16	0.07	0.30	0.11	0.05	0.69	0.20
1945	TR.2 A1 H1 S2 03.07.23 one each	Gegham 1	2	1	2	A1	7/3/23	192	7/6/23	665	4094	38	217	14	24	70	53	3.11	0.19	0.34	0.75	0.06	0.11	0.32	0.24
1946	TR.2 A1 H1 S2 03.07.23 one each	Kelbadjar	2	1	2	A1	7/3/23	193	7/6/23	555	5441	41	165	17	11	113	36	1.46	0.15	0.10	0.32	0.10	0.07	0.68	0.22
1947	TR.2 A1 H1 S2 03.07.23 one each	Kelbadjar	2	1	2	A1	7/3/23	194	7/6/23	520	5332	44	162	17	13	119	33	1.36	0.14	0.11	0.28	0.10	0.08	0.74	0.21
1948	TR.2 A1 H1 S2 03.07.23 one each	Gegham 1	2	1	2	A1	7/3/23	195	7/6/23	767	4265	36	221	16	27	73	54	3.04	0.21	0.37	0.74	0.07	0.12	0.33	0.24
1949	TR.2 A1 H1 S2 03.07.23 one each	Gegham 1	2	1	2	A1	7/3/23	196	7/6/23	794	4275	39	229	14	26	78	55	2.92	0.17	0.33	0.71	0.06	0.11	0.34	0.24
1950	TR.2 A1 H1 S2 03.07.23 one each	Gegham 1	2	1	2	A1	7/3/23	197	7/6/23	729	4063	35	217	13	26	73	55	2.95	0.17	0.35	0.75	0.06	0.12	0.34	0.25
1951	TR.2 A1 H1 S2 03.07.23 one each	Gegham 1	2	1	2	A1	7/3/23	198	7/6/23	675	4063	34	214	16	27	73	58	2.91	0.21	0.36	0.79	0.07	0.12	0.34	0.27
1952	TR.2 A1 H1 S2 03.07.23 one each	Kelbadjar	2	1	2	A1	7/3/23	199	7/6/23	446	4597	41	147	16	11	103	33	1.43	0.15	0.11	0.32	0.11	0.08	0.70	0.23
1953	TR.2 A1 H1 S2 03.07.23 one each	Gegham 1	2	1	2	A1	7/3/23	200	7/6/23	645	3610	29	202	11	26	73	51	2.79	0.16	0.35	0.70	0.06	0.13	0.36	0.25
1954	TR.2 A1 H1 S2 03.07.23 one each	Kelbadjar	2	1	2	A1	7/3/23	201	7/6/23	519	5340	46	164	17	10	108	36	1.52	0.15	0.09	0.34	0.10	0.06	0.66	0.22
1955	TR.2 A1 H1 S2 03.07.23 one each	Gegham 1	2	1	2	A1	7/3/23	202	7/6/23	829	4567	41	236	13	24	73	57	3.22	0.17	0.32	0.77	0.05	0.10	0.31	0.24
1956	TR.2 A1 H1 S2 03.07.23 one each	Kelbadjar	2	1	2	A1	7/3/23	203	7/6/23	542	5708	47	171	17	11	110	39	1.55	0.15	0.10	0.35	0.10	0.07	0.64	0.23
1957	TR.2 A1 H1 S2 03.07.23 one each	Kelbadjar	2	1	2	A1	7/3/23	204	7/6/23	486	4981	36	156	17	11	110	35	1.41	0.15	0.10	0.31	0.11	0.07	0.71	0.22
1958	TR.2 A1 H1 S2 03.07.23 one each	Gegham 1	2	1	2	A1	7/3/23	205	7/6/23	754	4243	39	226	13	23	71	54	3.19	0.18	0.32	0.76	0.06	0.10	0.31	0.24
1959	TR.2 A1 H1 S2 03.07.23 one each	Gegham 1	2	1	2	A1	7/3/23	206	7/6/23	908	5002	42	247	15	25	73	54	3.41	0.20	0.34	0.74	0.06	0.10	0.29	0.22
1960	TR.2 A1 H1 S2 03.07.23 one each	Gegham 1	2	1	2	A1	7/3/23	207	7/6/23	814	4508	44	236	14	25	76	55	3.10	0.18	0.32	0.72	0.06	0.10	0.32	0.23
1961	TR.2 A1 H1 S2 03.07.23 one each	Gegham 1	2	1	2	A1	7/3/23	208	7/6/23	721	4113	37	227	14	25	73	57	3.08	0.18	0.34	0.77	0.06	0.11	0.32	0.25
1962	TR.2 A1 H1 S2 03.07.23 one each	Kelbadjar	2	1	2	A1	7/3/23	209	7/6/23	539	5538	46	168	17	11	113	37	1.49	0.15	0.10	0.33	0.10	0.07	0.67	0.22
1963	TR.2 A1 H1 S2 03.07.23 one each	Kelbadjar	2	1	2	A1	7/3/23	210	7/6/23	549	5618	39	169	18	11	108	33	1.56	0.16	0.11	0.31	0.10	0.07	0.64	0.20
1964	TR.2 A1 H1 S2 03.07.23 one each	Kelbadjar	2	1	2	A1	7/3/23	211	7/6/23	604	6325	48	179	17	12	119	39	1.51	0.14	0.10	0.33	0.09	0.07	0.66	0.22
1965	TR.2 A1 H1 S2 03.07.23 one each	Gegham 1	2	1	2	A1	7/3/23	212	7/6/23	734	4180	35	218	15	27	73	55	2.97	0.20	0.36	0.75	0.07	0.12	0.34	0.25
1966	TR.2 A1 H1 S2 03.07.23 one each	Syunik	2	1	2	A1	7/3/23	213	7/6/23	478	5338	41	184	23	12	101	33	1.82	0.22	0.12	0.33	0.12	0.07	0.55	0.18
1967	TR.2 A1 H1 S2 03.07.23 one each	Gegham 1	2	1	2	A1	7/3/23	214	7/6/23	801	4183	35	219	13	27	75	55	2.90	0.17	0.35	0.73	0.06	0.12	0.34	0.25
1968	TR.2 A1 H1 S2 03.07.23 one each	Gegham 1	2	1	2	A1	7/3/23	215	7/6/23	749	4233	36	224	14	26	72	54	3.13	0.19	0.36	0.75	0.06	0.11	0.32	0.24
1969	TR.2 A1 H1 S2 03.07.23 one each	Gegham 1	2	1	2	A1	7/3/23	216	7/6/23	731	4010	35	222	14	27	75	58	2.94	0.18	0.35	0.77	0.06	0.12	0.34	0.26
1970	TR.2 A1 H1 S2 03.07.23 one each	Gegham 1	2	1	2	A1	7/3/23	217	7/6/23	703	4175	33	221	13	27	73	57	3.04	0.17	0.37	0.78	0.06	0.12	0.33	0.26
1971	TR.2 A1 H1 S2 03.07.23 one each	Gegham 1	2	1	2	A1	7/3/23	219	7/6/23	787	4398	40	241	15	27	76	58	3.16	0.19	0.35	0.76	0.06	0.11	0.32	0.24
1972	TR.2 A1 H1 S2 03.07.23 one each	Kelbadjar	2	1	2	A1	7/3/23	220	7/6/23	638	5931	51	175	19	10	108	37	1.61	0.17	0.09	0.34	0.11	0.05	0.62	0.21
1973	TR.2 A1 H1 S2 03.07.23 one each	Kelbadjar	2	1	2	A1	7/3/23	221	7/6/23	595	5932	48	176	17	11	116	39	1.52	0.14	0.09	0.33	0.09	0.06	0.66	0.22
1974	TR.2 A1 H1 S2 03.07.23 one each	Kelbadjar	2	1	2	A1	7/3/23	222	7/6/23	574	5670	46	170	21	11	109	33	1.56	0.19	0.10	0.30	0.12	0.06	0.64	0.20
1975	TR.2 A1 H1 S2 03.07.23 one each	Kelbadjar	2	1	2	A1	7/3/23	223	7/6/23	620	6148	51	184	18	10	116	39	1.59	0.15	0.08	0.33	0.10	0.05	0.63	0.21
1976	TR.2 A1 H1 S2 03.07.23 one each	Kelbadjar	2	1	2	A1	7/3/23	224	7/6/23	651	6159	52	186	21	11	116	43	1.60	0.18	0.09	0.37	0.11	0.06	0.62	0.23
1977	TR.2 A1 H1 S2 03.07.23 one each	Gegham 1	2	1	2	A1	7/3/23	225	7/6/23	894	4992	41	247	14	24	74	57	3.32	0.18	0.32	0.76	0.05	0.10	0.30	0.23
1978	TR.2 A1 H1 S2 03.07.23 one each	Gegham 1	2	1	2	A1	7/3/23	226	7/6/23	843	4399	35	232	13	25	73	54	3.15	0.17	0.34	0.73	0.05	0.11	0.32	0.23
1979	TR.2 A1 H1 S2 03.07.23 one each	Kelbadjar	2	1	2	A1	7/3/23	227	7/6/23	570	5478	46	174	18	11	109	37	1.59	0.16	0.10	0.34	0.10	0.07	0.63	0.21
1980	TR.2 A1 H1 S2 03.07.23 one each	Kelbadjar	2	1	2	A1	7/3/23	228	7/6/23	581	5926	45	178	18	11	112	36	1.59	0.16	0.10	0.32	0.10	0.06	0.63	0.20
1981	TR.2 A1 H1 S2 03.07.23 one each	Gegham 1	2	1	2	A1	7/3/23	229	7/6/23	771	4201	36	229	14	25	77	59	2.96	0.17	0.32	0.77	0.06	0.11	0.34	0.26
1982	TR.2 A1 H1 S2 03.07.23 one each	Kelbadjar	2	1	2	A1	7/3/23	230	7/6/23	541	5764	44	170	20	11	113	37	1.50	0.17	0.10	0.33	0.11	0.07	0.66	0.22
1983	TR.2 A1 H1 S2 03.07.23 one each	Kelbadjar	2	1	2	A1	7/3/23	231	7/6/23	626	5696	44	176	17	11	122	39	1.45	0.14	0.09	0.32	0.09	0.06	0.69	0.22
1984	TR.2 A1 H1 S2 03.07.23 one each	Gegham 1	2	1	2	A1	7/3/23	232	7/6/23	920	4794	42	245	15	25	73	54	3.34	0.20	0.34	0.73	0.06	0.10	0.30	0.22
1985	TR.2 A1 H1 S2 03.07.23 one each	Kelbadjar	2	1	2	A1	7/3/23	233	7/6/23	567	5728	46	171	18	11	107	39	1.61	0.16	0.11	0.36	0.10	0.07	0.62	0.23

1986	TR.2 A1 H1 S2 03.07.23 one each	Kelbadjar	2	1	2	A1	7/3/23	234	7/6/23	575	5529	46	172	17	11	115	36	1.50	0.14	0.09	0.31	0.10	0.06	0.67	0.21
1987	TR.2 A1 H1 S2 03.07.23 one each	Kelbadjar	2	1	2	A1	7/3/23	236	7/6/23	566	5742	42	169	17	12	112	36	1.51	0.15	0.11	0.32	0.10	0.07	0.66	0.21
1988	TR.2 A1 H1 S2 03.07.23 one each	Gegham 1	2	1	2	A1	7/3/23	237	7/6/23	791	4166	37	222	15	25	72	53	3.10	0.20	0.34	0.73	0.07	0.11	0.32	0.24
1989	TR.2 A1 H1 S2 03.07.23 one each	Kelbadjar	2	1	2	A1	7/3/23	238	7/6/23	615	6483	51	181	17	10	114	36	1.59	0.14	0.08	0.32	0.09	0.05	0.63	0.20
1990	TR.2 A1 H1 S2 03.07.23 one each	Gegham 1	2	1	2	A1	7/3/23	239	7/6/23	813	4543	43	230	16	25	74	55	3.08	0.21	0.33	0.74	0.07	0.11	0.32	0.24
1991	TR.2 A1 H1 S2 03.07.23 one each	Kelbadjar	2	1	2	A1	7/3/23	240	7/6/23	609	6069	49	178	18	13	114	36	1.56	0.15	0.12	0.32	0.10	0.07	0.64	0.20
1992	TR.2 A1 H1 S2 03.07.23 one each	Gegham 1	2	1	2	A1	7/3/23	242	7/6/23	812	4228	43	235	15	26	75	55	3.12	0.19	0.34	0.73	0.06	0.11	0.32	0.23
1993	TR.2 A1 H1 S2 03.07.23 one each	Kelbadjar	2	1	2	A1	7/3/23	244	7/6/23	546	5584	43	165	18	12	108	39	1.53	0.16	0.11	0.36	0.11	0.07	0.66	0.23
1994	TR.2 A1 H1 S2 03.07.23 one each	Syunik	2	1	2	A1	7/3/23	245	7/6/23	597	5906	45	198	24	9	103	36	1.93	0.23	0.08	0.35	0.12	0.04	0.52	0.18
1995	TR.2 A1 H1 S2 03.07.23 one each	Kelbadjar	2	1	2	A1	7/3/23	246	7/6/23	581	5914	50	177	17	11	117	36	1.51	0.14	0.10	0.31	0.09	0.06	0.66	0.20
1996	TR.2 A1 H1 S2 03.07.23 one each	Syunik	2	1	2	A1	7/3/23	247	7/6/23	655	6700	51	190	18	11	120	37	1.58	0.15	0.09	0.31	0.09	0.06	0.63	0.20
1997	TR.2 A1 H1 S2 03.07.23 one each	Syunik	2	1	2	A1	7/3/23	248	7/6/23	602	6016	43	181	19	10	110	33	1.64	0.17	0.09	0.30	0.10	0.05	0.61	0.18
1998	TR.2 A1 H1 S2 03.07.23 one each	Kelbadjar	2	1	2	A1	7/3/23	249	7/6/23	555	5775	47	167	17	11	114	39	1.47	0.14	0.09	0.34	0.10	0.06	0.68	0.23
1999	TR.2 A1 H1 S2 03.07.23 one each	Kelbadjar	2	1	2	A1	7/3/23	250	7/6/23	530	5341	46	166	21	14	115	39	1.45	0.18	0.12	0.34	0.12	0.09	0.69	0.23
2000	Tr2 A2-A3 HOR.3 Sp.1 30.06.23 bag1 1 of 5	Gegham 1	2	3	1	A2-A3	6/30/23	251	7/6/23	697	3714	34	207	13	27	73	53	2.82	0.17	0.36	0.71	0.06	0.13	0.35	0.25
2001	Tr2 A2-A3 HOR.3 Sp.1 30.06.23 bag1 2 of 5	Kelbadjar	2	3	1	A2-A3	6/30/23	252	7/6/23	504	4965	41	159	16	11	107	35	1.49	0.15	0.10	0.33	0.10	0.07	0.67	0.22
2002	Tr2 A2-A3 HOR.3 Sp.1 30.06.23 bag1 3 of 5	Gegham 1	2	3	1	A2-A3	6/30/23	253	7/6/23	879	4786	42	240	16	25	77	58	3.11	0.20	0.32	0.75	0.06	0.10	0.32	0.24
2003	Tr2 A2-A3 HOR.3 Sp.1 30.06.23 bag1 4 of 5	Kelbadjar	2	3	1	A2-A3	6/30/23	254	7/6/23	557	5448	39	164	16	10	114	36	1.44	0.14	0.08	0.32	0.09	0.06	0.69	0.22
2004	Tr2 A2-A3 HOR.3 Sp.1 30.06.23 bag1 5 of 5	Kelbadjar	2	3	1	A2-A3	6/30/23	255	7/6/23	565	5659	48	170	18	10	108	35	1.57	0.16	0.09	0.32	0.10	0.06	0.64	0.20
2005	Tr2 A2-A3 HOR.3 Sp.1 30.06.23 bag2 large frac one each	Kelbadjar	2	3	1	A2-A3	6/30/23	256	7/6/23	490	4950	39	155	16	11	110	33	1.40	0.14	0.10	0.30	0.10	0.07	0.71	0.22
2006	Tr2 A2-A3 HOR.3 Sp.1 30.06.23 bag2 large frac one each	Gegham 1	2	3	1	A2-A3	6/30/23	257	7/6/23	693	3992	33	212	13	25	75	54	2.81	0.17	0.33	0.71	0.06	0.12	0.36	0.25
2007	Tr2 A2-A3 HOR.3 Sp.1 30.06.23 bag2 large frac one each	Kelbadjar	2	3	1	A2-A3	6/30/23	258	7/6/23	524	5367	39	166	16	11	111	36	1.50	0.14	0.10	0.32	0.09	0.07	0.67	0.22
2008	Tr2 A2-A3 HOR.3 Sp.1 30.06.23 bag2 large frac one each	Kelbadjar	2	3	1	A2-A3	6/30/23	259	7/6/23	572	5303	39	164	17	11	113	35	1.45	0.15	0.09	0.31	0.10	0.06	0.69	0.21
2009	Tr2 A2-A3 HOR.3 Sp.1 30.06.23 bag2 large frac one each	Kelbadjar	2	3	1	A2-A3	6/30/23	260	7/6/23	460	5142	38	157	17	10	106	35	1.48	0.16	0.09	0.33	0.11	0.06	0.67	0.22
2010	Tr2 A2-A3 HOR.3 Sp.1 30.06.23 bag2 large frac one each	Gegham 1	2	3	1	A2-A3	6/30/23	261	7/6/23	701	3716	31	207	14	23	68	54	3.06	0.20	0.34	0.79	0.07	0.11	0.33	0.26
2011	Tr2 A2-A3 HOR.3 Sp.1 30.06.23 bag2 large frac one each	Kelbadjar	2	3	1	A2-A3	6/30/23	262	7/6/23	508	5336	40	162	17	10	107	36	1.52	0.15	0.09	0.34	0.10	0.06	0.66	0.22
2012	Tr2 A2-A3 HOR.3 Sp.1 30.06.23 bag2 large frac one each	Gegham 1	2	3	1	A2-A3	6/30/23	263	7/6/23	739	4240	37	226	11	25	73	58	3.11	0.16	0.34	0.80	0.05	0.11	0.32	0.26
2013	Tr2 A2-A3 HOR.3 Sp.1 30.06.23 bag2 large frac one each	Kelbadjar	2	3	1	A2-A3	6/30/23	264	7/6/23	494	4991	43	159	22	10	108	36	1.48	0.20	0.09	0.34	0.14	0.06	0.68	0.23
2014	Tr2 A2-A3 HOR.3 Sp.1 30.06.23 bag2 large frac one each	Arteni	2	3	1	A2-A3	6/30/23	265	7/6/23	713	4317	42	145	22	28	83	36	1.75	0.26	0.34	0.43	0.15	0.20	0.57	0.25
2015	Tr2 A2-A3 HOR.3 Sp.1 30.06.23 bag2 large frac one each	Kelbadjar	2	3	1	A2-A3	6/30/23	266	7/6/23	486	5109	40	160	17	11	111	37	1.43	0.15	0.10	0.34	0.10	0.07	0.70	0.23
2016	Tr2 A2-A3 HOR.3 Sp.1 30.06.23 bag2 large frac one each	Gegham 1	2	3	1	A2-A3	6/30/23	267	7/6/23	753	4082	36	216	15	26	73	54	2.98	0.20	0.35	0.74	0.07	0.12	0.34	0.25
2017	Tr2 A2-A3 HOR.3 Sp.1 30.06.23 bag2 large frac one each	Kelbadjar	2	3	1	A2-A3	6/30/23	268	7/6/23	481	5023	37	158	17	9	107	32	1.48	0.15	0.08	0.30	0.10	0.05	0.68	0.20
2018	Tr2 A2-A3 HOR.3 Sp.1 30.06.23 bag2 large frac one each	Gegham 1	2	3	1	A2-A3	6/30/23	269	7/6/23	777	4450	39	232	15	23	76	57	3.03	0.19	0.30	0.74	0.06	0.10	0.33	0.24
2019	Tr2 A2-A3 HOR.3 Sp.1 30.06.23 bag2 large frac one each	Arteni	2	3	1	A2-A3	6/30/23	270	7/6/23	574	4514	40	126	39	26	88	28	1.43	0.44	0.29	0.32	0.31	0.20	0.70	0.22
2020	Tr2 A2-A3 HOR.3 Sp.1 30.06.23 bag2 large frac one each	Gegham 1	2	3	1	A2-A3	6/30/23	271	7/6/23	715	3866	35	209	16	27	75	59	2.77	0.21	0.35	0.79	0.07	0.13	0.36	0.28
2021	Tr2 A2-A3 HOR.3 Sp.1 30.06.23 bag2 large frac one each	Gegham 1	2	3	1	A2-A3	6/30/23	272	7/6/23	746	4104	35	211	13	27	69	53	3.07	0.18	0.39	0.76	0.06	0.13	0.33	0.25
2022	Tr2 A2-A3 HOR.3 Sp.1 30.06.23 bag2 large frac one each	Gegham 1	2	3	1	A2-A3	6/30/23	273	7/6/23	707	3849	33	214	13	25	72	55	2.99	0.17	0.34	0.77	0.06	0.12	0.33	0.26
2023	Tr2 A2-A3 HOR.3 Sp.1 30.06.23 bag2 large frac one each	Gegham 1	2	3	1	A2-A3	6/30/23	274	7/6/23	676	3654	34	203	14	26	70	54	2.92	0.19	0.37	0.77	0.07	0.13	0.34	0.26
2024	Tr2 A2-A3 HOR.3 Sp.1 30.06.23 bag2 large frac one each	Kelbadjar	2	3	1	A2-A3	6/30/23	276	7/6/23	520	5327	35	166	17	11	112	37	1.48	0.15	0.10	0.33	0.10	0.07	0.67	0.22
2025	Tr2 A2-A3 HOR.3 Sp.1 30.06.23 bag2 large frac one each	Syunik	2	3	1	A2-A3	6/30/23	277	7/6/23	571	5787	42	198	21	11	108	35	1.85	0.19	0.11	0.32	0.10	0.06	0.54	0.17
2026	Tr2 A2-A3 HOR.3 Sp.1 30.06.23 bag2 large frac one each	Gegham 1	2	3	1	A2-A3	6/30/23	278	7/6/23	726	4023	35	219	15	25	72	54	3.06	0.20	0.34	0.75	0.07	0.11	0.33	0.25
2027	Tr2 A2-A3 HOR.3 Sp.1 30.06.23 bag2 large frac one each	Kelbadjar	2	3	1	A2-A3	6/30/23	279	7/6/23	523	5553	41	166	17	11	113	39	1.47	0.15	0.09	0.34	0.10	0.06	0.68	0.23
2028	Tr2 A2-A3 HOR.3 Sp.1 30.06.23 bag2 large frac one each	Kelbadjar	2	3	1	A2-A3	6/30/23	281	7/6/23	540	5756	46	171	19	11	112	36	1.53	0.16	0.09	0.32	0.11	0.06	0.66	0.21
2029	Tr2 A2-A3 HOR.3 Sp.1 30.06.23 bag2 large frac one each	Kelbadjar	2	3	1	A2-A3	6/30/23	282	7/6/23	519	5085	41	160	16	11	111	33	1.43	0.14	0.10	0.30	0.10	0.07	0.70	0.21
2030	Tr2 A2-A3 HOR.3 Sp.1 30.06.23 bag2 large frac one each	Kelbadjar	2	3	1	A2-A3	6/30/23	283	7/6/23	567	5740	44	176	17	9	110	35	1.60	0.15	0.08	0.31	0.09	0.05	0.63	0.20
2031	Tr2 A2-A3 HOR.3 Sp.1 30.06.23 bag2 large frac one each	Kelbadjar	2	3	1	A2-A3	6/30/23	285	7/6/23	632	6122	46	180	17	11	117	39	1.54	0.14	0.09	0.33	0.09	0.06	0.65	0.22
2032	Tr2 A2-A3 HOR.3 Sp.1 30.06.23 bag2 large frac one each	Gegham 1	2	3	1	A2-A3	6/30/23	287	7/6/23	710	4078	35	211	15	26	73	58	2.87	0.20	0.35	0.79	0.07	0.12	0.35	0.27
2033	Tr2 A2-A3 HOR.3 Sp.1 30.06.23 bag2 large frac one each	Gegham 1	2	3	1	A2-A3	6/30/23	288	7/6/23	742	4220	35	226	13	24	73	55	3.07	0.17	0.32	0.75	0.06	0.11	0.33	0.24
2034	Tr2 A2-A3 HOR.3 Sp.1 30.06.23 bag2 large frac one each	Syunik	2	3	1	A2-A3	6/30/23	289	7/6/23	561	5558	38	188	21	11	108	37	1.73	0.19	0.10	0.34	0.11	0.06	0.58	0.20
2035	Tr2 A2-A3 HOR.3 Sp.1 30.06.23 bag2 large frac one each	Hatis	2	3	1	A2-A3	6/30/23	290	7/6/23	562	7432	41	124	118	16	98	20	1.26	1.20	0.16	0.20	0.95	0.13	0.79	0.16
2036	Tr2 A2-A3 Hor3 Sp1 30.06 bag3 smalls sample50 one each	Gegham 1	2	3	1	A2-A3	6/30/23	293	7/6/23	706	4031	35	218	15	24	73	55	3.00	0.20	0.33	0.76	0.07	0.11	0.33	0.25

2037	Tr2 A2-A3 Hor3 Sp1 30.06 bag3 smalls sample50 one each	Kelbadjar	2	3	1	A2-A3	6/30/23	294	7/6/23	546	5298	42	164	18	12	108	35	1.52	0.16	0.11	0.32	0.11	0.08	0.66	0.21
2038	Tr2 A2-A3 Hor3 Sp1 30.06 bag3 smalls sample50 one each	Kelbadjar	2	3	1	A2-A3	6/30/23	295	7/6/23	524	5310	43	160	16	11	112	37	1.42	0.14	0.10	0.33	0.10	0.07	0.70	0.23
2039	Tr2 A2-A3 Hor3 Sp1 30.06 bag3 smalls sample50 one each	Kelbadjar	2	3	1	A2-A3	6/30/23	296	7/6/23	526	5378	41	163	16	10	111	37	1.46	0.14	0.09	0.34	0.10	0.06	0.68	0.23
2040	Tr2 A2-A3 Hor3 Sp1 30.06 bag3 smalls sample50 one each	Kelbadjar	2	3	1	A2-A3	6/30/23	297	7/6/23	607	6092	47	176	18	11	112	35	1.57	0.16	0.09	0.31	0.10	0.06	0.64	0.20
2041	Tr2 A2-A3 Hor3 Sp1 30.06 bag3 smalls sample50 one each	Kelbadjar	2	3	1	A2-A3	6/30/23	298	7/6/23	529	5207	40	160	17	11	110	35	1.45	0.15	0.10	0.31	0.10	0.07	0.69	0.22
2042	Tr2 A2-A3 Hor3 Sp1 30.06 bag3 smalls sample50 one each	Gegham 1	2	3	1	A2-A3	6/30/23	299	7/6/23	671	3959	34	213	14	27	73	53	2.94	0.19	0.37	0.72	0.06	0.12	0.34	0.25
2043	Tr2 A2-A3 Hor3 Sp1 30.06 bag3 smalls sample50 one each	Kelbadjar	2	3	1	A2-A3	6/30/23	300	7/6/23	546	5489	41	165	18	11	114	37	1.45	0.15	0.09	0.33	0.11	0.06	0.69	0.23
2044	Tr2 A2-A3 Hor3 Sp1 30.06 bag3 smalls sample50 one each	Gegham 1	2	3	1	A2-A3	6/30/23	301	7/6/23	903	5022	44	251	15	26	77	54	3.25	0.19	0.33	0.70	0.06	0.10	0.31	0.21
2045	Tr2 A2-A3 Hor3 Sp1 30.06 bag3 smalls sample50 one each	Kelbadjar	2	3	1	A2-A3	6/30/23	302	7/6/23	614	6191	46	180	18	11	122	35	1.48	0.14	0.09	0.28	0.10	0.06	0.68	0.19
2046	Tr2 A2-A3 Hor3 Sp1 30.06 bag3 smalls sample50 one each	Gegham 1	2	3	1	A2-A3	6/30/23	303	7/6/23	712	4240	35	220	17	24	74	53	2.95	0.22	0.32	0.71	0.08	0.11	0.34	0.24
2047	Tr2 A2-A3 Hor3 Sp1 30.06 bag3 smalls sample50 one each	Kelbadjar	2	3	1	A2-A3	6/30/23	304	7/6/23	561	5864	44	171	17	11	114	39	1.50	0.14	0.09	0.34	0.10	0.06	0.67	0.23
2048	Tr2 A2-A3 Hor3 Sp1 30.06 bag3 smalls sample50 one each	Kelbadjar	2	3	1	A2-A3	6/30/23	305	7/6/23	589	5974	46	179	17	11	113	35	1.58	0.15	0.09	0.31	0.09	0.06	0.63	0.19
2049	Tr2 A2-A3 Hor3 Sp1 30.06 bag3 smalls sample50 one each	Gegham 1	2	3	1	A2-A3	6/30/23	306	7/6/23	726	4069	36	223	14	25	76	55	2.92	0.18	0.32	0.72	0.06	0.11	0.34	0.25
2050	Tr2 A2-A3 Hor3 Sp1 30.06 bag3 smalls sample50 one each	Kelbadjar	2	3	1	A2-A3	6/30/23	307	7/6/23	607	5831	46	174	17	11	110	33	1.58	0.15	0.10	0.30	0.09	0.07	0.63	0.19
2051	Tr2 A2-A3 Hor3 Sp1 30.06 bag3 smalls sample50 one each	Kelbadjar	2	3	1	A2-A3	6/30/23	308	7/6/23	547	5748	40	171	17	11	108	36	1.58	0.15	0.10	0.33	0.10	0.06	0.63	0.21
2052	Tr2 A2-A3 Hor3 Sp1 30.06 bag3 smalls sample50 one each	Kelbadjar	2	3	1	A2-A3	6/30/23	309	7/6/23	496	5239	42	163	18	12	114	33	1.42	0.15	0.11	0.29	0.11	0.08	0.70	0.20
2053	Tr2 A2-A3 Hor3 Sp1 30.06 bag3 smalls sample50 one each	Kelbadjar	2	3	1	A2-A3	6/30/23	310	7/6/23	539	5697	44	170	17	11	122	37	1.40	0.14	0.09	0.31	0.10	0.06	0.71	0.22
2054	Tr2 A2-A3 Hor3 Sp1 30.06 bag3 smalls sample50 one each	Gegham 1	2	3	1	A2-A3	6/30/23	311	7/6/23	726	4034	35	223	14	27	73	54	3.03	0.18	0.36	0.73	0.06	0.12	0.33	0.24
2055	Tr2 A2-A3 Hor3 Sp1 30.06 bag3 smalls sample50 one each	Gegham 1	2	3	1	A2-A3	6/30/23	312	7/6/23	687	3803	33	211	13	26	72	54	2.95	0.17	0.36	0.75	0.06	0.12	0.34	0.26
2056	Tr2 A2-A3 Hor3 Sp1 30.06 bag3 smalls sample50 one each	Gegham 1	2	3	1	A2-A3	6/30/23	313	7/6/23	777	4220	41	226	16	26	74	57	3.03	0.21	0.34	0.76	0.07	0.11	0.33	0.25
2057	Tr2 A2-A3 Hor3 Sp1 30.06 bag3 smalls sample50 one each	Kelbadjar	2	3	1	A2-A3	6/30/23	314	7/6/23	515	5298	41	163	19	10	112	35	1.45	0.16	0.09	0.31	0.11	0.06	0.69	0.21
2058	Tr2 A2-A3 Hor3 Sp1 30.06 bag3 smalls sample50 one each	Kelbadjar	2	3	1	A2-A3	6/30/23	315	7/6/23	515	5382	41	168	19	11	112	33	1.50	0.16	0.10	0.30	0.11	0.07	0.67	0.20
2059	Tr2 A2-A3 Hor3 Sp1 30.06 bag3 smalls sample50 one each	Kelbadjar	2	3	1	A2-A3	6/30/23	316	7/6/23	566	5818	45	175	18	10	114	35	1.53	0.15	0.08	0.30	0.10	0.05	0.65	0.20
2060	Tr2 A2-A3 Hor3 Sp1 30.06 bag3 smalls sample50 one each	Syunik	2	3	1	A2-A3	6/30/23	317	7/6/23	519	5507	36	183	25	11	109	35	1.67	0.22	0.10	0.32	0.13	0.06	0.60	0.19
2061	Tr2 A2-A3 Hor3 Sp1 30.06 bag3 smalls sample50 one each	Gegham 1	2	3	1	A2-A3	6/30/23	318	7/6/23	792	4321	35	231	14	25	73	54	3.18	0.19	0.34	0.74	0.06	0.11	0.31	0.23
2062	Tr2 A2-A3 Hor3 Sp1 30.06 bag3 smalls sample50 one each	Syunik	2	3	1	A2-A3	6/30/23	319	7/6/23	547	5709	43	191	25	13	108	36	1.77	0.23	0.12	0.34	0.13	0.07	0.56	0.19
2063	Tr2 A2-A3 Hor3 Sp1 30.06 bag3 smalls sample50 one each	Gegham 1	2	3	1	A2-A3	6/30/23	320	7/6/23	804	4350	37	228	14	27	73	55	3.14	0.19	0.38	0.76	0.06	0.12	0.32	0.24
2064	Tr2 A2-A3 Hor3 Sp1 30.06 bag3 smalls sample50 one each	Gegham 1	2	3	1	A2-A3	6/30/23	321	7/6/23	727	4175	39	225	15	26	76	57	2.94	0.19	0.34	0.74	0.06	0.11	0.34	0.25
2065	Tr2 A2-A3 Hor3 Sp1 30.06 bag3 smalls sample50 one each	Gegham 1	2	3	1	A2-A3	6/30/23	322	7/6/23	761	4482	37	235	14	23	73	57	3.25	0.19	0.31	0.78	0.06	0.10	0.31	0.24
2066	Tr2 A2-A3 Hor3 Sp1 30.06 bag3 smalls sample50 one each	Gegham 1	2	3	1	A2-A3	6/30/23	323	7/6/23	822	4518	38	239	15	26	76	54	3.14	0.19	0.34	0.71	0.06	0.11	0.32	0.23
2067	Tr2 A2-A3 Hor3 Sp1 30.06 bag3 smalls sample50 one each	Gegham 1	2	3	1	A2-A3	6/30/23	324	7/6/23	716	4001	35	216	13	27	73	53	2.94	0.17	0.36	0.71	0.06	0.12	0.34	0.24
2068	Tr2 A2-A3 Hor3 Sp1 30.06 bag3 smalls sample50 one each	Kelbadjar	2	3	1	A2-A3	6/30/23	325	7/6/23	520	5196	45	164	17	11	113	36	1.44	0.15	0.09	0.32	0.10	0.06	0.69	0.22
2069	Tr2 A2-A3 Hor3 Sp1 30.06 bag3 smalls sample50 one each	Gegham 1	2	3	1	A2-A3	6/30/23	326	7/6/23	788	4146	38	222	13	25	74	53	2.98	0.17	0.33	0.71	0.06	0.11	0.34	0.24
2070	Tr2 A2-A3 Hor3 Sp1 30.06 bag3 smalls sample50 one each	Gegham 1	2	3	1	A2-A3	6/30/23	327	7/6/23	749	4384	35	230	15	27	75	55	3.05	0.19	0.36	0.73	0.06	0.12	0.33	0.24
2071	Tr2 A2-A3 Hor3 Sp1 30.06 bag3 smalls sample50 one each	Gegham 1	2	3	1	A2-A3	6/30/23	328	7/6/23	691	3989	37	216	15	26	72	54	3.02	0.20	0.36	0.75	0.07	0.12	0.33	0.25
2072	Tr2 A2-A3 Hor3 Sp1 30.06 bag3 smalls sample50 one each	Kelbadjar	2	3	1	A2-A3	6/30/23	329	7/6/23	565	5606	43	163	18	13	120	37	1.36	0.15	0.11	0.31	0.11	0.08	0.74	0.23
2073	Tr2 A2-A3 Hor3 Sp1 30.06 bag3 smalls sample50 one each	Gegham 1	2	3	1	A2-A3	6/30/23	330	7/6/23	811	4460	42	234	16	22	73	55	3.23	0.21	0.30	0.76	0.07	0.09	0.31	0.24
2074	Tr2 A2-A3 Hor3 Sp1 30.06 bag3 smalls sample50 one each	Gegham 1	2	3	1	A2-A3	6/30/23	331	7/6/23	779	4212	40	232	16	27	74	54	3.11	0.21	0.37	0.72	0.07	0.12	0.32	0.23
2075	Tr2 A2-A3 Hor3 Sp1 30.06 bag3 smalls sample50 one each	Syunik	2	3	1	A2-A3	6/30/23	332	7/6/23	665	6491	48	211	25	11	108	36	1.96	0.23	0.11	0.34	0.12	0.05	0.51	0.17
2076	Tr2 A2-A3 Hor3 Sp1 30.06 bag3 smalls sample50 one each	Kelbadjar	2	3	1	A2-A3	6/30/23	333	7/6/23	624	5948	45	175	19	11	109	36	1.60	0.17	0.10	0.33	0.11	0.06	0.62	0.21
2077	Tr2 A2-A3 Hor3 Sp1 30.06 bag3 smalls sample50 one each	Gegham 1	2	3	1	A2-A3	6/30/23	334	7/6/23	718	3990	36	220	13	24	71	55	3.11	0.18	0.34	0.78	0.06	0.11	0.32	0.25
2078	Tr2 A2-A3 Hor3 Sp1 30.06 bag3 smalls sample50 one each	Gegham 1	2	3	1	A2-A3	6/30/23	335	7/6/23	860	4800	47	245	18	25	75	55	3.25	0.23	0.33	0.73	0.07	0.10	0.31	0.23
2079	Tr2 A2-A3 Hor3 Sp1 30.06 bag3 smalls sample50 one each	Gegham 1	2	3	1	A2-A3	6/30/23	336	7/6/23	737	4151	35	224	13	26	73	53	3.08	0.17	0.35	0.72	0.06	0.11	0.32	0.23
2080	Tr2 A2-A3 Hor3 Sp1 30.06 bag3 smalls sample50 one each	Gegham 1	2	3	1	A2-A3	6/30/23	337	7/6/23	868	4629	40	239	15	27	76	61	3.14	0.19	0.35	0.80	0.06	0.11	0.32	0.25
2081	Tr2 A2-A3 Hor3 Sp1 30.06 bag3 smalls sample50 one each	Kelbadjar	2	3	1	A2-A3	6/30/23	338	7/6/23	513	5494	41	164	17	12	108	33	1.52	0.15	0.11	0.31	0.10	0.08	0.66	0.20
2082	Tr2 A2-A3 Hor3 Sp1 30.06 bag3 smalls sample50 one each	Gegham 1	2	3	1	A2-A3	6/30/23	339	7/6/23	1115	5926	48	281	16	24	76	59	3.68	0.20	0.31	0.78	0.06	0.08	0.27	0.21
2083	Tr2 A2-A3 Hor3 Sp1 30.06 bag3 smalls sample50 one each	Kelbadjar	2	3	1	A2-A3	6/30/23	340	7/6/23	565	5651	47	174	19	14	112	37	1.55	0.16	0.13	0.33	0.11	0.08	0.64	0.21
2084	Tr2 A2-A3 Hor3 Sp1 30.06 bag3 smalls sample50 one each	Gegham 1	2	3	1	A2-A3	6/30/23	341	7/6/23	902	4953	40	256	15	24	74	53	3.44	0.19	0.32	0.71	0.06	0.09	0.29	0.21
2085	TR.2 UN.B2 H2 Sp2 one each	Gegham 1	2	2	2	B2	-	343	7/6/23	636	3680	33	201	14	26	75	54	2.67	0.18	0.34	0.71	0.07	0.13	0.37	0.27
2086	TR.2 UN.B2 H2 Sp2 one each	Gegham 1	2	2	2	B2	-	344	7/6/23	705	3936	35	214	11	27	74	54	2.88	0.15	0.37	0.72	0.05	0.13	0.35	0.25
2087	TR.2 UN.B2 H2 Sp2 one each	Gegham 1	2	2	2	B2	-	345	7/6/23	673	3796	32	207	15	24	73	57	2.82	0.20	0.32	0.77	0.07	0.11	0.35	0.27

2088	TR.2 UN.B2 H2 Sp2 one each	Gegham 1	2	2	2	B2	-	346	7/6/23	783	4367	38	232	13	24	74	55	3.11	0.17	0.32	0.74	0.05	0.10	0.32	0.24
2089	TR.2 UN.B2 H2 Sp2 one each	Gegham 2?	2	2	2	B2	-	347	7/6/23	587	4955	64	135	46	25	93	25	1.45	0.49	0.26	0.27	0.34	0.18	0.69	0.19
2090	TR.2 UN.B2 H2 Sp2 one each	Gegham 1	2	2	2	B2	-	348	7/6/23	782	4251	37	225	14	26	74	53	3.02	0.18	0.34	0.71	0.06	0.11	0.33	0.23
2091	TR.2 UN.B2 H2 Sp2 one each	Gegham 1	2	2	2	B2	-	349	7/6/23	737	4181	41	218	14	25	73	55	2.97	0.18	0.34	0.75	0.06	0.11	0.34	0.25
2092	TR.2 UN.B2 H2 Sp2 one each	Gegham 1	2	2	2	B2	-	350	7/6/23	1002	5126	53	256	16	26	76	57	3.35	0.20	0.34	0.74	0.06	0.10	0.30	0.22
2093	TR.2 UN.B2 H2 Sp2 one each	Gegham 1	2	2	2	B2	-	351	7/6/23	820	4357	44	235	15	26	77	55	3.05	0.19	0.33	0.71	0.06	0.11	0.33	0.23
2094	TR.2 UN.B2 H2 Sp2 one each	Kelbadjar	2	2	2	B2	-	352	7/6/23	587	5879	46	175	18	11	109	39	1.60	0.16	0.10	0.35	0.10	0.06	0.62	0.22
2095	TR.2 UN-C0 H2 S1 04.07.23 1 of 2	Gegham 1	2	2	1	C0	7/4/23	353	7/6/23	695	3915	32	218	14	26	72	55	3.04	0.19	0.36	0.77	0.06	0.12	0.33	0.25
2096	TR.2 UN-C0 H2 S1 04.07.23 2 of 2	Gegham 1	2	2	1	C0	7/4/23	354	7/6/23	884	4471	39	239	15	27	77	57	3.10	0.19	0.36	0.73	0.06	0.11	0.32	0.24
2097	TR.2 UN-C0 H2 S1 04.07.23 2 of 2	Gegham 1	2	2	1	C0	7/4/23	355	7/6/23	731	3951	41	229	14	26	75	53	3.03	0.18	0.34	0.70	0.06	0.11	0.33	0.23
2098	TR.2 UN B1 H4 Sp1 PtS8 1 of 2	Gegham 1	2	4	1	B1	-	356	7/6/23	655	3805	36	210	13	26	72	55	2.94	0.17	0.36	0.77	0.06	0.12	0.34	0.26
2099	TR.2 UN B1 H4 Sp1 PtS8 2 of 2	Gegham 1	2	4	1	B1	-	357	7/6/23	661	3706	35	205	11	24	72	54	2.87	0.16	0.33	0.75	0.06	0.12	0.35	0.26
2100	TR.2 H2.S2 UN.A1 04.07.23 1 of 1	Gegham 2	2	2	2	A1	7/4/23	358	7/6/23	556	5888	44	113	53	26	113	26	1.00	0.46	0.23	0.23	0.47	0.23	1.00	0.23
2101	TR.2 UN-C2 H2 Sp1 04.07.23 one each	Gegham 1	2	2	1	C2	7/4/23	359	7/6/23	682	3790	34	208	13	24	73	55	2.83	0.17	0.32	0.75	0.06	0.11	0.35	0.27
2102	TR.2 UN-C2 H2 Sp1 04.07.23 one each	Gegham 1	2	2	1	C2	7/4/23	360	7/6/23	700	4068	33	215	14	25	73	51	2.93	0.18	0.34	0.70	0.06	0.11	0.34	0.24
2103	TR.2 UN-C2 H2 Sp1 04.07.23 one each	Arteni	2	2	1	C2	7/4/23	361	7/6/23	845	4624	51	160	23	35	78	47	2.04	0.29	0.45	0.60	0.14	0.22	0.49	0.29
2104	TR.2 UN-C2 H2 Sp1 04.07.23 one each	Gegham 1	2	2	1	C2	7/4/23	362	7/6/23	791	4356	40	235	15	26	78	53	3.01	0.19	0.33	0.67	0.06	0.11	0.33	0.22
2105	TR.2 UN-B0 H2 S2 04.07.23 one each	Kelbadjar	2	2	2	B0	7/4/23	363	7/6/23	508	5216	41	160	18	10	111	32	1.43	0.16	0.09	0.29	0.11	0.06	0.70	0.20
2106	TR.2 UN-B0 H2 S2 04.07.23 one each	Gegham 1	2	2	2	B0	7/4/23	364	7/6/23	648	3816	33	202	14	23	73	51	2.79	0.19	0.31	0.70	0.07	0.11	0.36	0.25
2107	TR.2 UN-B0 H2 S2 04.07.23 one each	Kelbadjar	2	2	2	B0	7/4/23	365	7/6/23	461	4610	35	168	18	11	96	31	1.75	0.18	0.12	0.32	0.10	0.07	0.57	0.18
2108	TR.2 UN-B0 H2 S2 04.07.23 one each	Kelbadjar	2	2	2	B0	7/4/23	366	7/6/23	525	5154	36	161	17	11	105	33	1.53	0.16	0.11	0.32	0.10	0.07	0.65	0.21
2109	TR.2 UN-B0 H2 S2 04.07.23 one each	Kelbadjar	2	2	2	B0	7/4/23	367	7/6/23	526	5426	44	170	19	11	111	33	1.53	0.17	0.09	0.30	0.11	0.06	0.65	0.20
2110	TR.2 UN-B0 H2 S2 04.07.23 one each	Gegham 1	2	2	2	B0	7/4/23	368	7/6/23	763	4386	37	231	14	27	74	54	3.10	0.18	0.36	0.72	0.06	0.12	0.32	0.23
2111	TR.2 UN-B0 H2 S2 04.07.23 one each	Gegham 1	2	2	2	B0	7/4/23	369	7/6/23	700	3978	38	212	14	27	73	55	2.89	0.18	0.36	0.75	0.06	0.13	0.35	0.26
2112	TR.2 UN-B0 H2 S2 04.07.23 one each	Gegham 1	2	2	2	B0	7/4/23	370	7/6/23	873	4817	45	244	14	25	72	57	3.41	0.19	0.34	0.79	0.06	0.10	0.29	0.23
2113	TR.2 UN-B0 H2 S2 04.07.23 one each	Syunik	2	2	2	B0	7/4/23	371	7/6/23	451	5387	44	187	24	10	109	35	1.71	0.22	0.09	0.32	0.13	0.05	0.59	0.19
2114	TR.2 UN-B0 H2 S2 04.07.23 one each	Gegham 1	2	2	2	B0	7/4/23	372	7/6/23	1072	5173	51	255	15	24	73	59	3.47	0.20	0.32	0.81	0.06	0.09	0.29	0.23
2115	TR.2 H2 S2 A0 04.07.23 one each	Gegham 1	2	2	2	A0	7/4/23	374	7/6/23	661	3897	30	209	14	26	72	55	2.92	0.19	0.36	0.77	0.06	0.12	0.34	0.26
2116	TR.2 H2 S2 A0 04.07.23 one each	Gegham 1	2	2	2	A0	7/4/23	375	7/6/23	724	4051	32	220	11	23	73	53	2.99	0.16	0.31	0.71	0.05	0.10	0.33	0.24
2117	TR.2 H2 S2 A0 04.07.23 one each	Arteni	2	2	2	A0	7/4/23	376	7/6/23	636	4134	41	133	23	29	85	32	1.57	0.27	0.35	0.38	0.17	0.22	0.64	0.24
2118	TR.2 H2 S2 A0 04.07.23 one each	Gegham 1	2	2	2	A0	7/4/23	377	7/6/23	766	4262	39	232	15	26	73	57	3.15	0.20	0.35	0.77	0.06	0.11	0.32	0.24
2119	TR.2 H2 S2 A0 04.07.23 one each	Gegham 1	2	2	2	A0	7/4/23	378	7/6/23	945	5185	45	255	14	23	74	55	3.42	0.18	0.31	0.74	0.05	0.09	0.29	0.22
2120	TR.2 H2 S2 A0 04.07.23 one each	Arteni	2	2	2	A0	7/4/23	379	7/6/23	859	5255	57	172	23	30	91	35	1.88	0.25	0.33	0.38	0.13	0.18	0.53	0.20
2121	TR.2 H2 S2 A0 04.07.23 one each	Gegham 1	2	2	2	A0	7/4/23	381	7/6/23	1012	5253	44	259	15	27	75	55	3.43	0.19	0.35	0.73	0.06	0.10	0.29	0.21
2122	TR.2 H2 S2 A0 04.07.23 one each	Gegham 1	2	2	2	A0	7/4/23	382	7/6/23	968	5288	46	260	16	24	76	55	3.40	0.20	0.31	0.72	0.06	0.09	0.29	0.21
2123	TR-2 Unit-A1 H3 Sp1 06.07 one each	Kelbadjar	2	3	1	A1	7/6/23	385	7/6/23	521	5370	44	163	19	10	108	33	1.51	0.17	0.09	0.31	0.11	0.06	0.66	0.20
2124	TR-2 Unit-A1 H3 Sp1 06.07 one each	Gegham 1	2	3	1	A1	7/6/23	386	7/6/23	824	4776	39	245	15	26	76	54	3.21	0.19	0.34	0.71	0.06	0.10	0.31	0.22
2125	Tr2 C0 H3 Sp2 1 of 1	Gegham 1	2	3	2	C0	-	387	7/6/23	744	4082	35	216	13	27	73	53	2.94	0.17	0.37	0.71	0.06	0.13	0.34	0.24
2126	Tr2 Unit-A0 H5 Sp2 06.07 one each	Gegham 1	2	5	2	A0	7/6/23	388	7/6/23	637	3889	31	209	11	26	72	53	2.92	0.16	0.36	0.73	0.05	0.12	0.34	0.25
2127	Tr2 Unit-A0 H5 Sp2 06.07 one each	Gegham 1	2	5	2	A0	7/6/23	389	7/6/23	809	4374	36	230	14	26	74	54	3.08	0.18	0.34	0.72	0.06	0.11	0.32	0.23
2128	Tr-2 C1 H3 Sp2 06.07 one each	Gegham 1	2	3	2	C1	7/6/23	390	7/6/23	746	4197	35	223	14	27	74	57	2.99	0.18	0.36	0.76	0.06	0.12	0.33	0.25
2129	Tr-2 C1 H3 Sp2 06.07 one each	Arteni	2	3	2	C1	7/6/23	391	7/6/23	754	4668	47	157	22	32	85	35	1.85	0.25	0.38	0.41	0.14	0.21	0.54	0.22
2130	Tr-2 C1 H4 Sp1 one each	Gegham 1	2	4	1	C1	-	392	7/6/23	696	4078	36	220	15	27	73	57	2.99	0.20	0.36	0.77	0.07	0.12	0.33	0.26
2131	Tr-2 C1 H4 Sp1 one each	Syunik	2	4	1	C1	-	1	7/7/23	439	4873	34	181	26	11	103	33	1.76	0.25	0.10	0.32	0.14	0.06	0.57	0.18
2132	Tr-2 C1 H4 Sp1 one each	Gegham 1	2	4	1	C1	-	2	7/7/23	991	5395	51	265	17	23	75	58	3.51	0.22	0.30	0.77	0.06	0.09	0.28	0.22
2133	Tr-2 C1 H4 Sp1 one each	Gegham 1	2	4	1	C1	-	3	7/7/23	835	4613	37	238	17	28	75	57	3.16	0.22	0.38	0.75	0.07	0.12	0.32	0.24
2134	Tr2 Unit-B0 H4 Sp1 05.07 one each	Gegham 1	2	4	1	B0	7/5/23	4	7/7/23	701	3925	33	215	13	23	72	57	3.00	0.17	0.32	0.79	0.06	0.11	0.33	0.26
2135	Tr2 Unit-B0 H4 Sp1 05.07 one each	Gegham 1	2	4	1	B0	7/5/23	5	7/7/23	720	4060	31	218	13	25	74	55	2.93	0.17	0.33	0.74	0.06	0.11	0.34	0.25
2136	Tr2 Unit-B0 H4 Sp1 05.07 one each	Gegham 1	2	4	1	B0	7/5/23	6	7/7/23	670	3676	31	205	14	23	70	53	2.95	0.19	0.33	0.75	0.07	0.11	0.34	0.26
2137	Tr2 Unit-B0 H4 Sp1 05.07 one each	Gegham 1	2	4	1	B0	7/5/23	7	7/7/23	753	4138	36	223	14	26	73	54	3.07	0.19	0.35	0.74	0.06	0.11	0.33	0.24
2138	Tr2 Unit-A1 H4 Sp1 one each	Gegham 1	2	4	1	A1	-	8	7/7/23	768	4213	37	224	15	27	75	57	2.97	0.19	0.35	0.75	0.06	0.12	0.34	0.25

2139	Tr2 Unit-A1 H4 Sp1 one each	Syunik	2	4	1	A1	-	9	7/7/23	498	5528	40	184	24	11	106	36	1.74	0.22	0.10	0.34	0.13	0.06	0.57	0.20
2140	Tr2 Unit-A1 H4 Sp1 one each	Syunik	2	4	1	A1	-	10	7/7/23	456	4961	36	179	24	12	106	36	1.70	0.22	0.12	0.34	0.13	0.07	0.59	0.20
2141	Tr2 Unit-A1 H4 Sp1 one each	Arteni	2	4	1	A1	-	11	7/7/23	617	4861	42	133	38	26	91	28	1.47	0.41	0.28	0.31	0.28	0.19	0.68	0.21
2142	Tr2 A0 H4 Sp1 one each	Gegham 1	2	4	1	A0	-	13	7/7/23	806	4439	35	229	14	25	73	57	3.15	0.19	0.34	0.78	0.06	0.11	0.32	0.25
2143	Tr2 A0 H4 Sp1 one each	Gegham 1	2	4	1	A0	-	14	7/7/23	660	3766	35	208	13	25	73	53	2.87	0.17	0.34	0.72	0.06	0.12	0.35	0.25
2144	Tr2 A0 H4 Sp1 one each	Syunik	2	4	1	A0	-	15	7/7/23	637	5278	42	218	13	10	96	39	2.27	0.13	0.10	0.40	0.06	0.04	0.44	0.18
2145	TR.2 UN-A0 H2 Sp1 04.07.23 one each	Gegham 1	2	2	1	A0	7/4/23	17	7/7/23	711	4029	35	209	13	25	73	55	2.88	0.17	0.34	0.76	0.06	0.12	0.35	0.26
2146	TR.2 UN-A0 H2 Sp1 04.07.23 one each	Gegham 1	2	2	1	A0	7/4/23	18	7/7/23	754	4176	35	219	14	27	75	54	2.90	0.18	0.35	0.71	0.06	0.12	0.34	0.25
2147	TR.2 UN-A0 H2 Sp1 04.07.23 one each	Gegham 1	2	2	1	A0	7/4/23	19	7/7/23	712	4072	34	218	13	25	73	55	3.00	0.17	0.34	0.76	0.06	0.11	0.33	0.25
2148	TR.2 UN-A0 H2 Sp1 04.07.23 one each	Gegham 1	2	2	1	A0	7/4/23	20	7/7/23	650	3805	35	205	15	24	74	55	2.76	0.19	0.32	0.74	0.07	0.12	0.36	0.27
2149	TR.2 UN-A0 H2 Sp1 04.07.23 one each	Syunik	2	2	1	A0	7/4/23	21	7/7/23	574	4557	33	197	11	11	93	37	2.11	0.12	0.11	0.40	0.06	0.05	0.47	0.19
2150	TR.2 UN-A0 H2 Sp1 04.07.23 one each	Arteni	2	2	1	A0	7/4/23	22	7/7/23	619	4908	41	131	42	27	91	31	1.44	0.45	0.29	0.33	0.32	0.20	0.70	0.23
2151	TR.2 UN-A0 H2 Sp1 04.07.23 one each	Gegham 1	2	2	1	A0	7/4/23	23	7/7/23	705	4051	36	224	14	24	74	57	3.01	0.18	0.32	0.76	0.06	0.11	0.33	0.25
2152	TR.2 UN-A0 H2 Sp1 04.07.23 one each	Gegham 1	2	2	1	A0	7/4/23	24	7/7/23	825	4535	40	234	15	27	75	53	3.11	0.19	0.35	0.70	0.06	0.11	0.32	0.22
2153	TR.2 UN-A0 H2 Sp1 04.07.23 one each	Gegham 1	2	2	1	A0	7/4/23	25	7/7/23	870	4485	37	233	16	26	77	59	3.01	0.20	0.33	0.77	0.07	0.11	0.33	0.26
2154	TR.2 UN-A0 H2 Sp1 04.07.23 one each	Gegham 1	2	2	1	A0	7/4/23	26	7/7/23	788	4215	39	226	15	26	74	54	3.03	0.19	0.34	0.72	0.06	0.11	0.33	0.24
2155	TR.2 UN-A0 H2 Sp1 04.07.23 one each	Gegham 1	2	2	1	A0	7/4/23	27	7/7/23	939	4686	48	248	15	26	73	51	3.38	0.20	0.35	0.70	0.06	0.10	0.30	0.21
2156	TR.2 UN-A0 H2 Sp1 04.07.23 one each	Syunik	2	2	1	A0	7/4/23	28	7/7/23	579	5800	41	200	24	10	105	35	1.91	0.22	0.09	0.33	0.12	0.05	0.52	0.17
2157	TR.2 UN-A0 H2 Sp1 04.07.23 one each	Gegham 1	2	2	1	A0	7/4/23	29	7/7/23	828	4622	44	240	15	26	77	55	3.11	0.19	0.33	0.71	0.06	0.11	0.32	0.23
2158	TR.2 UN-A0 H2 Sp1 04.07.23 one each	Gegham 1	2	2	1	A0	7/4/23	30	7/7/23	951	5383	44	260	16	24	73	55	3.53	0.21	0.32	0.75	0.06	0.09	0.28	0.21
2159	TR.2 UN-A0 H2 Sp1 04.07.23 one each	Syunik	2	2	1	A0	7/4/23	31	7/7/23	592	6380	46	204	26	11	110	37	1.85	0.23	0.10	0.34	0.12	0.05	0.54	0.18
2160	TR.2 UN-A0 H2 Sp1 04.07.23 one each	Gegham 1	2	2	1	A0	7/4/23	32	7/7/23	793	4159	42	231	14	27	76	58	3.02	0.18	0.35	0.76	0.06	0.12	0.33	0.25
2161	TR.2 UN B0 H2 Sp1 04.07.23 one each	Gegham 1	2	2	1	B0	7/4/23	33	7/7/23	656	3762	28	209	10	25	78	57	2.67	0.13	0.32	0.72	0.05	0.12	0.37	0.27
2162	TR.2 UN B0 H2 Sp1 04.07.23 one each	Gegham 1	2	2	1	B0	7/4/23	34	7/7/23	660	3687	35	215	14	25	73	54	2.96	0.19	0.34	0.74	0.06	0.11	0.34	0.25
2163	TR.2 UN B0 H2 Sp1 04.07.23 one each	Kelbadjar	2	2	1	B0	7/4/23	35	7/7/23	491	5061	37	157	16	11	105	36	1.50	0.15	0.10	0.34	0.10	0.07	0.67	0.23
2164	TR.2 UN B0 H2 Sp1 04.07.23 one each	Gegham 1	2	2	1	B0	7/4/23	36	7/7/23	883	4772	45	246	15	25	75	57	3.26	0.19	0.33	0.75	0.06	0.10	0.31	0.23
2165	TR.2 UN-A1 H2 S1 04.07.23 one each	Kelbadjar	2	2	1	A1	7/4/23	37	7/7/23	504	5140	37	163	20	11	110	35	1.47	0.18	0.10	0.31	0.12	0.06	0.68	0.21
2166	TR.2 UN-A1 H2 S1 04.07.23 one each	Syunik	2	2	1	A1	7/4/23	38	7/7/23	554	5657	40	190	21	11	107	35	1.78	0.19	0.11	0.33	0.11	0.06	0.56	0.18
2167	TR.2 UN-A1 H2 S1 04.07.23 one each	Arteni	2	2	1	A1	7/4/23	39	7/7/23	645	4131	42	144	21	31	86	36	1.68	0.24	0.36	0.42	0.14	0.22	0.60	0.25
2168	TR.2 UN-A1 H2 S1 04.07.23 one each	Gegham 1	2	2	1	A1	7/4/23	40	7/7/23	663	3690	33	204	11	25	73	54	2.78	0.16	0.34	0.73	0.06	0.12	0.36	0.26
2169	TR.2 UN-A1 H2 S1 04.07.23 one each	Gegham 1	2	2	1	A1	7/4/23	41	7/7/23	675	3801	35	208	13	25	73	54	2.87	0.17	0.34	0.74	0.06	0.12	0.35	0.26
2170	TR.2 UN-A1 H2 S1 04.07.23 one each	Syunik	2	2	1	A1	7/4/23	42	7/7/23	514	5272	39	182	21	11	105	36	1.74	0.20	0.11	0.34	0.11	0.06	0.58	0.20
2171	TR.2 UN-A1 H2 S1 04.07.23 one each	Kelbadjar	2	2	1	A1	7/4/23	43	7/7/23	508	5099	35	162	18	11	110	36	1.46	0.16	0.10	0.33	0.11	0.07	0.68	0.22
2172	TR.2 UN-A1 H2 S1 04.07.23 one each	Gegham 1	2	2	1	A1	7/4/23	46	7/7/23	838	4561	39	240	13	25	74	54	3.23	0.17	0.33	0.72	0.05	0.10	0.31	0.22
2173	TR.2 UN-A1 H2 S1 04.07.23 one each	Kelbadjar	2	2	1	A1	7/4/23	47	7/7/23	663	6347	50	181	18	11	113	36	1.60	0.15	0.10	0.32	0.10	0.06	0.63	0.20
2174	TR.2 UN-A1 H2 S1 04.07.23 one each	Kelbadjar	2	2	1	A1	7/4/23	49	7/7/23	537	5491	40	163	18	12	113	33	1.44	0.15	0.11	0.29	0.11	0.08	0.70	0.20
2175	Tr-2 B1 H4 Sp1 06.07 one each	Gegham 1	2	4	1	B1	7/6/23	50	7/7/23	722	4323	37	216	15	25	72	54	3.02	0.20	0.34	0.75	0.07	0.11	0.33	0.25
2176	Tr-2 B1 H4 Sp1 06.07 one each	Hatis	2	4	1	B1	7/6/23	51	7/7/23	499	7113	36	106	127	16	99	17	1.07	1.28	0.16	0.17	1.19	0.15	0.93	0.16
2177	Tr-2 B1 H4 Sp1 06.07 one each	Gegham 1	2	4	1	B1	7/6/23	52	7/7/23	710	4127	37	216	13	27	73	55	2.94	0.17	0.36	0.75	0.06	0.12	0.34	0.26
2178	Tr-2 B1 H4 Sp1 06.07 one each	Gegham 1	2	4	1	B1	7/6/23	53	7/7/23	696	3895	39	220	14	24	72	57	3.07	0.19	0.33	0.79	0.06	0.11	0.33	0.26
2179	Tr-2 B1 H4 Sp1 06.07 one each	Gegham 1	2	4	1	B1	7/6/23	54	7/7/23	732	4006	36	215	14	24	71	54	3.04	0.19	0.34	0.76	0.06	0.11	0.33	0.25
2180	Tr-2 B1 H4 Sp1 06.07 one each	Syunik	2	4	1	B1	7/6/23	55	7/7/23	473	4995	34	177	22	11	109	36	1.62	0.20	0.10	0.33	0.12	0.06	0.62	0.20
2181	Tr-2 B1 H4 Sp1 06.07 one each	Gegham 1	2	4	1	B1	7/6/23	56	7/7/23	731	4207	35	222	15	25	79	55	2.80	0.18	0.31	0.70	0.07	0.11	0.36	0.25
2182	Tr-2 B1 H4 Sp1 06.07 one each	Arteni	2	4	1	B1	7/6/23	57	7/7/23	671	4650	42	145	30	26	82	32	1.77	0.36	0.31	0.39	0.20	0.18	0.57	0.22
2183	Tr-2 B1 H4 Sp1 06.07 one each	Gegham 1	2	4	1	B1	7/6/23	58	7/7/23	792	4586	37	238	13	23	74	55	3.20	0.17	0.31	0.74	0.05	0.10	0.31	0.23
2184	Tr-2 B1 H4 Sp1 06.07 one each	Gegham 1	2	4	1	B1	7/6/23	59	7/7/23	807	4718	42	239	15	25	73	59	3.26	0.20	0.34	0.81	0.06	0.10	0.31	0.25
2185	TR2 UN.C0 H2 Sp2 04.07.23 one each	Gegham 1	2	2	2	C0	7/4/23	60	7/7/23	727	3727	33	205	14	23	71	54	2.91	0.19	0.32	0.76	0.07	0.11	0.34	0.26
2186	TR2 UN.C0 H2 Sp2 04.07.23 one each	Gegham 1	2	2	2	C0	7/4/23	61	7/7/23	703	3947	39	219	14	23	73	57	3.02	0.19	0.31	0.78	0.06	0.10	0.33	0.26
2187	TR2 UN.C0 H2 Sp2 04.07.23 one each	Gegham 1	2	2	2	C0	7/4/23	62	7/7/23	655	3519	33	203	13	23	70	53	2.92	0.18	0.33	0.75	0.06	0.11	0.34	0.26
2188	TR2 UN.C0 H2 Sp2 04.07.23 one each	Gegham 1	2	2	2	C0	7/4/23	63	7/7/23	661	3762	35	211	13	24	72	55	2.95	0.17	0.33	0.77	0.06	0.11	0.34	0.26
2189	TR2 UN.C0 H2 Sp2 04.07.23 one each	Syunik	2	2	2	C0	7/4/23	64	7/7/23	530	5313	38	185	21	9	102	32	1.82	0.20	0.08	0.31	0.11	0.05	0.55	0.17

2190	TR2 UN.C0 H2 Sp2 04.07.23 one each	Gegham 1	2	2	2	C0	7/4/23	66	7/7/23	853	4813	41	239	15	25	76	57	3.14	0.19	0.32	0.74	0.06	0.10	0.32	0.24
2191	TR2 UN.C0 H2 Sp2 04.07.23 one each	Kelbadjar	2	2	2	C0	7/4/23	67	7/7/23	628	6405	49	184	20	11	117	36	1.57	0.17	0.10	0.31	0.11	0.06	0.64	0.20
2192	TR2 UN.C0 H2 Sp2 04.07.23 one each	Syunik	2	2	2	C0	7/4/23	68	7/7/23	540	5487	44	185	26	11	107	36	1.73	0.24	0.11	0.34	0.14	0.06	0.58	0.19
2193	TR2 UN.C0 H2 Sp2 04.07.23 one each	Gegham 1	2	2	2	C0	7/4/23	69	7/7/23	680	4012	36	211	14	26	73	55	2.87	0.18	0.35	0.75	0.06	0.12	0.35	0.26
2194	TR2 UN.C0 H2 Sp2 04.07.23 one each	Kelbadjar	2	2	2	C0	7/4/23	70	7/7/23	537	5410	44	164	18	14	110	39	1.49	0.16	0.13	0.35	0.11	0.09	0.67	0.24
2195	TR2 UN.C0 H2 Sp2 04.07.23 one each	Gegham 1	2	2	2	C0	7/4/23	71	7/7/23	849	4606	42	244	16	27	76	55	3.20	0.20	0.36	0.72	0.06	0.11	0.31	0.23
2196	TR2 UN.C0 H2 Sp2 04.07.23 one each	Kelbadjar	2	2	2	C0	7/4/23	72	7/7/23	567	5338	42	164	18	11	106	36	1.56	0.17	0.10	0.34	0.11	0.06	0.64	0.22
2197	TR2 UN.C0 H2 Sp2 04.07.23 one each	Gegham 1	2	2	2	C0	7/4/23	73	7/7/23	829	4611	43	235	15	24	73	58	3.25	0.20	0.33	0.80	0.06	0.10	0.31	0.25
2198	TR2 UN.C0 H2 Sp2 04.07.23 one each	Gegham 1	2	2	2	C0	7/4/23	74	7/7/23	837	4315	41	233	13	27	77	59	3.01	0.16	0.36	0.77	0.05	0.12	0.33	0.26
2199	TR2 UN.C0 H2 Sp2 04.07.23 one each	Gegham 1	2	2	2	C0	7/4/23	75	7/7/23	766	4094	35	227	13	23	73	59	3.08	0.17	0.31	0.81	0.06	0.10	0.32	0.26
2200	TR2 UN.C0 H2 Sp2 04.07.23 one each	Gegham 1	2	2	2	C0	7/4/23	76	7/7/23	873	4710	38	244	16	29	78	58	3.12	0.20	0.38	0.74	0.06	0.12	0.32	0.24
2201	TR2 UN.C0 H2 Sp2 04.07.23 one each	Gegham 1	2	2	2	C0	7/4/23	77	7/7/23	824	4317	38	231	15	24	72	57	3.22	0.20	0.33	0.79	0.06	0.10	0.31	0.25
2202	TR2 UN.C0 H2 Sp2 04.07.23 one each	Syunik	2	2	2	C0	7/4/23	78	7/7/23	736	7081	52	198	18	9	143	37	1.39	0.12	0.06	0.26	0.09	0.04	0.72	0.19
2203	TR.2 UN-A1 H2 Sp2 04.07.23 one each	Gegham 1	2	2	2	A1	7/4/23	82	7/7/23	761	4222	36	223	15	25	69	54	3.24	0.21	0.36	0.78	0.07	0.11	0.31	0.24
2204	TR.2 UN-A1 H2 Sp2 04.07.23 one each	Gegham 1	2	2	2	A1	7/4/23	83	7/7/23	663	3721	29	201	15	25	72	51	2.81	0.20	0.34	0.71	0.07	0.12	0.36	0.25
2205	TR.2 UN-A1 H2 Sp2 04.07.23 one each	Gegham 1	2	2	2	A1	7/4/23	84	7/7/23	759	4270	34	221	14	25	77	58	2.86	0.17	0.32	0.75	0.06	0.11	0.35	0.26
2206	TR.2 UN-A1 H2 Sp2 04.07.23 one each	Gegham 1	2	2	2	A1	7/4/23	85	7/7/23	753	4124	33	217	13	26	73	58	2.95	0.17	0.35	0.79	0.06	0.12	0.34	0.27
2207	TR.2 UN-A1 H2 Sp2 04.07.23 one each	Syunik	2	2	2	A1	7/4/23	86	7/7/23	503	5443	43	190	21	11	102	33	1.86	0.20	0.10	0.33	0.11	0.06	0.54	0.18
2208	TR.2 UN-A1 H2 Sp2 04.07.23 one each	Gegham 1	2	2	2	A1	7/4/23	88	7/7/23	859	4644	41	238	13	27	74	59	3.20	0.17	0.36	0.80	0.05	0.11	0.31	0.25
2209	TR.2 UN-A1 H2 Sp2 04.07.23 one each	Gegham 1	2	2	2	A1	7/4/23	90	7/7/23	786	4193	38	231	14	26	74	54	3.10	0.18	0.34	0.72	0.06	0.11	0.32	0.23
2210	TR.2 UN-A1 H2 Sp2 04.07.23 one each	Gegham 1	2	2	2	A1	7/4/23	92	7/7/23	701	3992	32	213	15	25	74	54	2.86	0.19	0.33	0.72	0.07	0.12	0.35	0.25
2211	TR.2 UN-A1 H2 Sp2 04.07.23 one each	Kelbadjar	2	2	2	A1	7/4/23	93	7/7/23	537	5270	41	160	18	11	110	36	1.45	0.16	0.10	0.33	0.11	0.07	0.69	0.23
2212	TR.2 UN-A1 H2 Sp2 04.07.23 one each	Gegham 1	2	2	2	A1	7/4/23	94	7/7/23	1204	6384	55	281	15	27	76	57	3.68	0.19	0.35	0.74	0.05	0.09	0.27	0.20
2213	TR.2 UN-B1 H2 S1 04.07.23 one each	Kelbadjar	2	2	1	B1	7/4/23	97	7/7/23	458	4716	34	153	21	10	107	31	1.43	0.19	0.09	0.29	0.13	0.06	0.70	0.20
2214	TR.2 UN-B1 H2 S1 04.07.23 one each	Gegham 1	2	2	1	B1	7/4/23	98	7/7/23	804	4518	37	234	17	24	73	55	3.19	0.22	0.32	0.75	0.07	0.10	0.31	0.24
2215	TR.2 UN-B1 H2 S1 04.07.23 one each	Kelbadjar	2	2	1	B1	7/4/23	99	7/7/23	459	4950	39	157	21	11	111	33	1.41	0.18	0.10	0.30	0.13	0.07	0.71	0.21
2216	TR.2 UN-B1 H2 S1 04.07.23 one each	Kelbadjar	2	2	1	B1	7/4/23	100	7/7/23	579	5776	43	171	24	11	117	36	1.46	0.20	0.09	0.31	0.14	0.06	0.68	0.21
2217	TR.2 UN-B1 H2 S1 04.07.23 one each	Gegham 1	2	2	1	B1	7/4/23	101	7/7/23	653	3951	38	210	14	26	72	53	2.94	0.19	0.36	0.73	0.06	0.12	0.34	0.25
2218	TR.2 UN-B1 H2 S1 04.07.23 one each	Kelbadjar	2	2	1	B1	7/4/23	102	7/7/23	542	5305	40	161	15	10	110	37	1.46	0.13	0.09	0.34	0.09	0.06	0.69	0.23
2219	TR.2 UN-B1 H2 S1 04.07.23 one each	Kelbadjar	2	2	1	B1	7/4/23	103	7/7/23	570	5576	46	167	17	11	110	35	1.52	0.15	0.10	0.31	0.10	0.06	0.66	0.21
2220	TR.2 UN-B1 H2 S1 04.07.23 one each	Kelbadjar	2	2	1	B1	7/4/23	104	7/7/23	523	5496	41	168	18	11	119	37	1.42	0.15	0.09	0.31	0.10	0.06	0.71	0.22
2221	TR.2 UN-B1 H2 S1 04.07.23 one each	Gegham 1	2	2	1	B1	7/4/23	105	7/7/23	787	4215	38	230	15	26	74	57	3.08	0.19	0.34	0.76	0.06	0.11	0.32	0.25
2222	TR.2 UN-B1 H2 S1 04.07.23 one each	Syunik	2	2	1	B1	7/4/23	106	7/7/23	646	6461	50	185	26	8	110	39	1.68	0.23	0.07	0.35	0.14	0.04	0.60	0.21
2223	TR.2 UN-B1 H2 S1 04.07.23 one each	Gegham 1	2	2	1	B1	7/4/23	107	7/7/23	834	4519	40	246	14	24	73	53	3.35	0.18	0.32	0.71	0.05	0.10	0.30	0.21
2224	TR.2 UN-B1 H2 S1 04.07.23 one each	Gegham 1	2	2	1	B1	7/4/23	108	7/7/23	688	3946	35	215	15	27	73	55	2.96	0.20	0.38	0.76	0.07	0.13	0.34	0.26
2225	TR.2 UN-B1 H2 S1 04.07.23 one each	Syunik	2	2	1	B1	7/4/23	109	7/7/23	528	5307	40	190	21	11	105	33	1.81	0.20	0.11	0.32	0.11	0.06	0.55	0.18
2226	TR.2 UN-B1 H2 S1 04.07.23 one each	Gegham 1	2	2	1	B1	7/4/23	110	7/7/23	754	4255	40	225	15	24	74	53	3.02	0.19	0.32	0.71	0.06	0.11	0.33	0.23
2227	TR.2 UN-B1 H2 S1 04.07.23 one each	Gegham 1	2	2	1	B1	7/4/23	111	7/7/23	897	4904	43	246	15	26	78	57	3.15	0.19	0.33	0.72	0.06	0.10	0.32	0.23
2228	TR.2 UN-B1 H2 S1 04.07.23 one each	Gegham 1	2	2	1	B1	7/4/23	112	7/7/23	767	4292	40	221	14	27	76	55	2.89	0.18	0.35	0.72	0.06	0.12	0.35	0.25
2229	TR.2 UN-B1 H2 S1 04.07.23 one each	Gegham 1	2	2	1	B1	7/4/23	113	7/7/23	743	4168	34	226	14	25	71	54	3.19	0.19	0.35	0.76	0.06	0.11	0.31	0.24
2230	TR.2 UN-B1 H2 S1 04.07.23 one each	Gegham 1	2	2	1	B1	7/4/23	115	7/7/23	729	4014	37	215	14	26	76	55	2.82	0.18	0.34	0.72	0.06	0.12	0.35	0.26
2231	TR.2 UN-B1 H2 S1 04.07.23 one each	Gegham 1	2	2	1	B1	7/4/23	116	7/7/23	890	4761	45	235	16	23	73	51	3.20	0.21	0.31	0.70	0.07	0.10	0.31	0.22
2232	TR.2 UN-B1 H2 S1 04.07.23 one each	Gegham 1	2	2	1	B1	7/4/23	117	7/7/23	822	4383	37	226	15	26	73	57	3.11	0.20	0.35	0.78	0.06	0.11	0.32	0.25
2233	TR.2 UN-B1 H2 S1 04.07.23 one each	Gegham 1	2	2	1	B1	7/4/23	118	7/7/23	906	5128	49	249	20	25	77	61	3.22	0.25	0.32	0.79	0.08	0.10	0.31	0.24
2234	TR.2 UN-B1 H2 S1 04.07.23 one each	Gegham 1	2	2	1	B1	7/4/23	119	7/7/23	929	4693	43	241	16	27	75	57	3.20	0.21	0.36	0.75	0.06	0.11	0.31	0.23
2235	TR.2 UN-B1 H2 S1 04.07.23 one each	Gegham 1	2	2	1	B1	7/4/23	120	7/7/23	686	4013	32	220	13	27	76	58	2.88	0.16	0.36	0.76	0.06	0.12	0.35	0.26
2236	TR.2 UN-B1 H2 S1 04.07.23 one each	Gegham 1	2	2	1	B1	7/4/23	121	7/7/23	978	4893	46	254	14	27	75	58	3.37	0.18	0.35	0.77	0.05	0.10	0.30	0.23
2237	TR.2 UN-B1 H2 S1 04.07.23 one each	Gegham 1	2	2	1	B1	7/4/23	122	7/7/23	746	4218	36	220	14	26	71	54	3.11	0.19	0.36	0.76	0.06	0.12	0.32	0.25
2238	TR.2 UN-B1 H2 S1 04.07.23 one each	Kelbadjar	2	2	1	B1	7/4/23	123	7/7/23	537	5534	47	165	20	14	109	35	1.51	0.18	0.13	0.32	0.12	0.09	0.66	0.21
2239	TR.2 UN-B1 H2 S1 04.07.23 one each	Gegham 1	2	2	1	B1	7/4/23	124	7/7/23	813	4371	41	233	13	27	74	55	3.12	0.17	0.36	0.74	0.05	0.11	0.32	0.24
2240	TR.2 UN-B1 H2 S1 04.07.23 one each	Gegham 2	2	2	1	B1	7/4/23	125	7/7/23	787	5956	55	154	43	28	98	32	1.57	0.43	0.29	0.33	0.28	0.18	0.64	0.21

2241	TR.2 UN-B1 H2 S1 04.07.23 one each	Gegham 1	2	2	1	B1	7/4/23	126	7/7/23	855	4625	41	238	23	24	75	59	3.16	0.30	0.31	0.79	0.09	0.10	0.32	0.25
2242	Tr2 TS-H1Sp1 U.A1-A4 collapse sample50 one each	Kelbadjar	2	0 - H1Sp1	collapse	A1-A4	-	128	7/7/23	571	5823	43	174	17	11	115	35	1.51	0.14	0.09	0.30	0.09	0.06	0.66	0.20
2243	Tr2 TS-H1Sp1 U.A1-A4 collapse sample50 one each	Gegham 1	2	0 - H1Sp1	collapse	A1-A4	-	129	7/7/23	713	3997	33	216	13	24	73	55	2.94	0.17	0.32	0.75	0.06	0.11	0.34	0.26
2244	Tr2 TS-H1Sp1 U.A1-A4 collapse sample50 one each	Gegham 1	2	0 - H1Sp1	collapse	A1-A4	-	130	7/7/23	738	4152	35	217	15	26	73	55	2.95	0.20	0.35	0.75	0.07	0.12	0.34	0.25
2245	Tr2 TS-H1Sp1 U.A1-A4 collapse sample50 one each	Gegham 1	2	0 - H1Sp1	collapse	A1-A4	-	131	7/7/23	793	4404	41	234	15	24	79	55	2.96	0.18	0.30	0.70	0.06	0.10	0.34	0.24
2246	Tr2 TS-H1Sp1 U.A1-A4 collapse sample50 one each	Gegham 1	2	0 - H1Sp1	collapse	A1-A4	-	132	7/7/23	761	4433	39	233	15	27	74	55	3.12	0.19	0.37	0.74	0.06	0.12	0.32	0.24
2247	Tr2 TS-H1Sp1 U.A1-A4 collapse sample50 one each	Gegham 1	2	0 - H1Sp1	collapse	A1-A4	-	133	7/7/23	850	4732	41	249	15	23	74	57	3.35	0.19	0.31	0.76	0.06	0.09	0.30	0.23
2248	Tr2 TS-H1Sp1 U.A1-A4 collapse sample50 one each	Gegham 1	2	0 - H1Sp1	collapse	A1-A4	-	134	7/7/23	727	4125	35	212	15	23	75	53	2.81	0.19	0.30	0.70	0.07	0.11	0.36	0.25
2249	Tr2 TS-H1Sp1 U.A1-A4 collapse sample50 one each	Kelbadjar	2	0 - H1Sp1	collapse	A1-A4	-	135	7/7/23	511	5012	40	156	16	11	108	36	1.44	0.14	0.11	0.33	0.10	0.07	0.70	0.23
2250	Tr2 TS-H1Sp1 U.A1-A4 collapse sample50 one each	Kelbadjar	2	0 - H1Sp1	collapse	A1-A4	-	136	7/7/23	541	5789	41	167	17	12	114	40	1.47	0.14	0.11	0.35	0.10	0.07	0.68	0.24
2251	Tr2 TS-H1Sp1 U.A1-A4 collapse sample50 one each	Gegham 1	2	0 - H1Sp1	collapse	A1-A4	-	137	7/7/23	673	3886	31	210	13	27	73	55	2.86	0.17	0.37	0.75	0.06	0.13	0.35	0.26
2252	Tr2 TS-H1Sp1 U.A1-A4 collapse sample50 one each	Gegham 1	2	0 - H1Sp1	collapse	A1-A4	-	138	7/7/23	877	4694	45	238	13	24	76	57	3.12	0.16	0.31	0.74	0.05	0.10	0.32	0.24
2253	Tr2 TS-H1Sp1 U.A1-A4 collapse sample50 one each	Syunik	2	0 - H1Sp1	collapse	A1-A4	-	139	7/7/23	493	5267	34	179	21	10	105	33	1.71	0.20	0.09	0.32	0.11	0.05	0.58	0.19
2254	Tr2 TS-H1Sp1 U.A1-A4 collapse sample50 one each	Gegham 1	2	0 - H1Sp1	collapse	A1-A4	-	140	7/7/23	811	4413	40	233	15	28	76	57	3.05	0.19	0.37	0.74	0.06	0.12	0.33	0.24
2255	Tr2 TS-H1Sp1 U.A1-A4 collapse sample50 one each	Gegham 1	2	0 - H1Sp1	collapse	A1-A4	-	141	7/7/23	713	4020	41	232	26	27	73	55	3.19	0.35	0.37	0.76	0.11	0.11	0.31	0.24
2256	Tr2 TS-H1Sp1 U.A1-A4 collapse sample50 one each	Gegham 1	2	0 - H1Sp1	collapse	A1-A4	-	142	7/7/23	721	4040	35	218	13	27	73	57	3.00	0.17	0.37	0.78	0.06	0.12	0.33	0.26
2257	Tr2 TS-H1Sp1 U.A1-A4 collapse sample50 one each	Kelbadjar	2	0 - H1Sp1	collapse	A1-A4	-	143	7/7/23	485	5214	39	160	22	11	113	32	1.41	0.19	0.10	0.28	0.13	0.07	0.71	0.20
2258	Tr2 TS-H1Sp1 U.A1-A4 collapse sample50 one each	Gegham 1	2	0 - H1Sp1	collapse	A1-A4	-	144	7/7/23	766	4374	37	230	13	26	73	55	3.12	0.17	0.35	0.75	0.05	0.11	0.32	0.24
2259	Tr2 TS-H1Sp1 U.A1-A4 collapse sample50 one each	Kelbadjar	2	0 - H1Sp1	collapse	A1-A4	-	145	7/7/23	607	6138	43	179	17	10	111	39	1.61	0.15	0.09	0.35	0.09	0.05	0.62	0.22
2260	Tr2 TS-H1Sp1 U.A1-A4 collapse sample50 one each	Gegham 1	2	0 - H1Sp1	collapse	A1-A4	-	146	7/7/23	763	4420	43	238	14	25	73	58	3.29	0.19	0.34	0.80	0.06	0.10	0.30	0.24
2261	Tr2 TS-H1Sp1 U.A1-A4 collapse sample50 one each	Kelbadjar	2	0 - H1Sp1	collapse	A1-A4	-	147	7/7/23	525	5406	42	166	18	13	111	33	1.50	0.16	0.12	0.30	0.11	0.08	0.67	0.20
2262	Tr2 TS-H1Sp1 U.A1-A4 collapse sample50 one each	Gegham 1	2	0 - H1Sp1	collapse	A1-A4	-	148	7/7/23	724	3966	42	212	15	21	72	53	2.96	0.20	0.29	0.73	0.07	0.10	0.34	0.25
2263	Tr2 TS-H1Sp1 U.A1-A4 collapse sample50 one each	Gegham 1	2	0 - H1Sp1	collapse	A1-A4	-	149	7/7/23	721	4032	40	210	14	23	72	55	2.94	0.19	0.32	0.77	0.06	0.11	0.34	0.26
2264	Tr2 TS-H1Sp1 U.A1-A4 collapse sample50 one each	Kelbadjar	2	0 - H1Sp1	collapse	A1-A4	-	150	7/7/23	534	5198	41	160	17	11	114	35	1.40	0.14	0.10	0.30	0.10	0.07	0.72	0.22
2265	Tr2 TS-H1Sp1 U.A1-A4 collapse sample50 one each	Kelbadjar	2	0 - H1Sp1	collapse	A1-A4	-	151	7/7/23	531	5471	43	166	17	11	113	33	1.47	0.15	0.09	0.29	0.10	0.06	0.68	0.20
2266	Tr2 TS-H1Sp1 U.A1-A4 collapse sample50 one each	Gegham 1	2	0 - H1Sp1	collapse	A1-A4	-	152	7/7/23	759	4246	39	229	15	27	74	57	3.07	0.19	0.36	0.76	0.06	0.12	0.33	0.25
2267	Tr2 TS-H1Sp1 U.A1-A4 collapse sample50 one each	Gegham 1	2	0 - H1Sp1	collapse	A1-A4	-	153	7/7/23	656	3849	31	206	13	25	77	55	2.67	0.16	0.32	0.71	0.06	0.12	0.37	0.27
2268	Tr2 TS-H1Sp1 U.A1-A4 collapse sample50 one each	Gegham 1	2	0 - H1Sp1	collapse	A1-A4	-	154	7/7/23	711	4062	38	218	14	27	75	57	2.89	0.18	0.35	0.75	0.06	0.12	0.35	0.26
2269	Tr2 TS-H1Sp1 U.A1-A4 collapse sample50 one each	Gegham 1	2	0 - H1Sp1	collapse	A1-A4	-	155	7/7/23	791	4234	34	228	11	26	75	58	3.02	0.15	0.34	0.77	0.05	0.11	0.33	0.25
2270	Tr2 TS-H1Sp1 U.A1-A4 collapse sample50 one each	Gegham 1	2	0 - H1Sp1	collapse	A1-A4	-	156	7/7/23	743	4164	35	221	13	26	76	57	2.89	0.16	0.34	0.74	0.06	0.12	0.35	0.26
2271	Tr2 TS-H1Sp1 U.A1-A4 collapse sample50 one each	Kelbadjar	2	0 - H1Sp1	collapse	A1-A4	-	157	7/7/23	533	5381	40	164	17	11	119	35	1.38	0.14	0.09	0.29	0.10	0.06	0.72	0.21
2272	Tr2 TS-H1Sp1 U.A1-A4 collapse sample50 one each	Gegham 1	2	0 - H1Sp1	collapse	A1-A4	-	159	7/7/23	941	5184	43	248	15	25	71	55	3.51	0.21	0.35	0.78	0.06	0.10	0.28	0.22
2273	Tr2 TS-H1Sp1 U.A1-A4 collapse sample50 one each	Syunik	2	0 - H1Sp1	collapse	A1-A4	-	160	7/7/23	579	6054	43	200	21	11	108	36	1.86	0.19	0.11	0.34	0.10	0.06	0.54	0.18
2274	Tr2 TS-H1Sp1 U.A1-A4 collapse sample50 one each	Gegham 1	2	0 - H1Sp1	collapse	A1-A4	-	161	7/7/23	753	4137	39	225	15	25	75	55	2.98	0.19	0.33	0.73	0.06	0.11	0.34	0.25
2275	Tr2 TS-H1Sp1 U.A1-A4 collapse sample50 one each	Gegham 1	2	0 - H1Sp1	collapse	A1-A4	-	162	7/7/23	741	4095	35	216	13	24	72	55	3.02	0.17	0.33	0.77	0.06	0.11	0.33	0.26
2276	Tr2 TS-H1Sp1 U.A1-A4 collapse sample50 one each	Gegham 1	2	0 - H1Sp1	collapse	A1-A4	-	163	7/7/23	763	3916	35	213	13	25	71	57	3.02	0.18	0.35	0.80	0.06	0.12	0.33	0.27
2277	Tr2 TS-H1Sp1 U.A1-A4 collapse sample50 one each	Syunik	2	0 - H1Sp1	collapse	A1-A4	-	164	7/7/23	481	5441	35	180	22	11	107	37	1.69	0.20	0.10	0.35	0.12	0.06	0.59	0.21
2278	Tr2 TS-H1Sp1 U.A1-A4 collapse sample50 one each	Gegham 1	2	0 - H1Sp1	collapse	A1-A4	-	165	7/7/23	756	4315	38	231	14	28	76	54	3.02	0.18	0.37	0.71	0.06	0.12	0.33	0.23
2279	Tr2 TS-H1Sp1 U.A1-A4 collapse sample50 one each	Gegham 1	2	0 - H1Sp1	collapse	A1-A4	-	166	7/7/23	808	4477	38	225	14	27	80	54	2.81	0.17	0.34	0.67	0.06	0.12	0.36	0.24
2280	Tr2 TS-H1Sp1 U.A1-A4 collapse sample50 one each	Syunik	2	0 - H1Sp1	collapse	A1-A4	-	167	7/7/23	458	5120	42	191	32	11	104	32	1.84	0.30	0.10	0.31	0.17	0.06	0.54	0.17
2281	Tr2 TS-H1Sp1 U.A1-A4 collapse sample50 one each	Gegham 1	2	0 - H1Sp1	collapse	A1-A4	-	168	7/7/23	711	4056	34	222	15	26	73	55	3.06	0.20	0.35	0.76	0.07	0.12	0.33	0.25
2282	Tr2 TS-H1Sp1 U.A1-A4 collapse sample50 one each	Gegham 1	2	0 - H1Sp1	collapse	A1-A4	-	169	7/7/23	733	3964	36	214	14	27	74	54	2.88	0.18	0.37	0.72	0.06	0.13	0.35	0.25
2283	Tr2 TS-H1Sp1 U.A1-A4 collapse sample50 one each	Gegham 1	2	0 - H1Sp1	collapse	A1-A4	-	170	7/7/23	776	4146	37	224	14	27	70	58	3.21	0.19	0.38	0.83	0.06	0.12	0.31	0.26
2284	Tr2 TS-H1Sp1 U.A1-A4 collapse sample50 one each	Gegham 1	2	0 - H1Sp1	collapse	A1-A4	-	171	7/7/23	862	4751	43	233	17	24	74	55	3.14	0.22	0.32	0.74	0.07	0.10	0.32	0.24
2285	Tr2 TS-H1Sp1 U.A1-A4 collapse sample50 one each	Gegham 1	2	0 - H1Sp1	collapse	A1-A4	-	172	7/7/23	754	4274	38	218	15	25	73	55	2.97	0.20	0.34	0.75	0.07	0.11	0.34	0.25
2286	Tr2 TS-H1Sp1 U.A1-A4 collapse sample50 one each	Gegham 1	2	0 - H1Sp1	collapse	A1-A4	-	173	7/7/23	807	4544	42	233	13	26	75	55	3.10	0.17	0.34	0.73	0.05	0.11	0.32	0.24
2287	Tr2 TS-H1Sp1 U.A1-A4 collapse sample50 one each	Kelbadjar	2	0 - H1Sp1	collapse	A1-A4	-	174	7/7/23	581	5916	46	174	18	11	112	33	1.55	0.16	0.09	0.30	0.10	0.06	0.64	0.19
2288	Tr2 TS-H1Sp1 U.A1-A4 collapse sample50 one each	Gegham 1	2	0 - H1Sp1	collapse	A1-A4	-	175	7/7/23	784	4314	36	230	14	26	73	54	3.12	0.18	0.35	0.73	0.06	0.11	0.32	0.23
2289	Tr2 TS-H1Sp1 U.A1-A4 collapse sample50 one each	Gegham 1	2	0 - H1Sp1	collapse	A1-A4	-	177	7/7/23	711	3998	35	219	14	27	75	58	2.90	0.18	0.35	0.77	0.06	0.12	0.34	0.26
2290	Tr-2 B1 H4 Sp1 07.07.23 one each	Gegham 1	2	4	1	B1	7/7/23	179	7/7/23	700	4052	35	212	13	26	73	55	2.92	0.17	0.35	0.76	0.06	0.12	0.34	0.26
2291	Tr-2 B1 H4 Sp1 07.07.23 one each	Gegham 1	2	4	1	B1	7/7/23	180	7/7/23	773	4318	39	223	13	22	73	55	3.07	0.17	0.30	0.76	0.06	0.10	0.33	0.25

2292	Tr-2 B1 H4 Sp1 07.07.23 one each	Gegham 1	2	4	1	B1	7/7/23	181	7/7/23	711	4252	45	227	19	24	69	51	3.30	0.27	0.34	0.74	0.08	0.10	0.30	0.23
2293	Tr-2 B1 H4 Sp1 07.07.23 one each	Gegham 1	2	4	1	B1	7/7/23	182	7/7/23	661	3898	33	209	15	25	72	51	2.92	0.20	0.34	0.71	0.07	0.12	0.34	0.24
2294	Tr-2 B1 H4 Sp1 07.07.23 one each	Gegham 1	2	4	1	B1	7/7/23	183	7/7/23	731	4059	35	213	14	27	75	54	2.83	0.18	0.35	0.71	0.06	0.12	0.35	0.25
2295	Tr-2 B1 H4 Sp1 07.07.23 one each	Gegham 1	2	4	1	B1	7/7/23	185	7/7/23	671	3655	31	203	13	27	70	54	2.92	0.18	0.38	0.77	0.06	0.13	0.34	0.26
2296	Tr-2 B1 H4 Sp1 07.07.23 one each	Gegham 1	2	4	1	B1	7/7/23	186	7/7/23	666	4069	45	210	20	24	73	51	2.90	0.27	0.33	0.70	0.09	0.11	0.35	0.24
2297	Tr-2 B1 H4 Sp1 07.07.23 one each	Gegham 1	2	4	1	B1	7/7/23	187	7/7/23	1031	5439	46	259	14	25	77	55	3.35	0.17	0.32	0.71	0.05	0.10	0.30	0.21
2298	Tr-2 B1 H4 Sp1 07.07.23 one each	Gegham 1	2	4	1	B1	7/7/23	188	7/7/23	706	3827	34	210	13	24	70	54	3.01	0.18	0.34	0.77	0.06	0.11	0.33	0.26
2299	Tr-2 B1 H4 Sp1 07.07.23 one each	Gegham 1	2	4	1	B1	7/7/23	189	7/7/23	630	3655	29	199	13	25	72	53	2.79	0.17	0.34	0.73	0.06	0.12	0.36	0.26
2300	Tr-2 B1 H4 Sp1 07.07.23 one each	Gegham 1	2	4	1	B1	7/7/23	190	7/7/23	766	4281	36	213	14	25	73	50	2.90	0.18	0.34	0.68	0.06	0.12	0.34	0.23
2301	Tr-2 B1 H4 Sp1 07.07.23 one each	Syunik	2	4	1	B1	7/7/23	191	7/7/23	575	5049	38	208	11	10	97	40	2.14	0.12	0.10	0.41	0.06	0.05	0.47	0.19
2302	Tr-2 B1 H4 Sp1 07.07.23 one each	Gegham 1	2	4	1	B1	7/7/23	193	7/7/23	749	4269	38	224	15	25	75	55	2.97	0.19	0.33	0.73	0.06	0.11	0.34	0.25
2303	Tr-2 B1 H4 Sp1 07.07.23 one each	Gegham 1	2	4	1	B1	7/7/23	194	7/7/23	945	5073	50	245	19	25	73	55	3.34	0.25	0.34	0.75	0.08	0.10	0.30	0.23
2304	Tr-2 B1 H4 Sp1 07.07.23 one each	Gegham 1	2	4	1	B1	7/7/23	195	7/7/23	778	4025	41	219	13	24	72	54	3.06	0.17	0.33	0.75	0.06	0.11	0.33	0.25
2305	Tr-2 B1 H4 Sp1 07.07.23 one each	Gegham 1	2	4	1	B1	7/7/23	196	7/7/23	930	4935	46	250	16	27	75	54	3.32	0.21	0.36	0.71	0.06	0.11	0.30	0.22
2306	T2 A1-C1 Hor.2 Sp.1 FL.5 one each	Gegham 1	2	2	1	A1-C1	-	199	7/7/23	779	4145	38	215	16	27	74	55	2.89	0.21	0.36	0.74	0.07	0.12	0.35	0.26
2307	T2 A1-C1 Hor.2 Sp.1 FL.5 one each	Kelbadjar	2	2	1	A1-C1	-	200	7/7/23	580	5862	48	170	19	11	112	33	1.52	0.16	0.10	0.30	0.11	0.07	0.66	0.20
2308	T2 A1-C1 Hor.2 Sp.1 FL.5 one each	Gegham 1	2	2	1	A1-C1	-	201	7/7/23	877	4732	41	237	15	27	73	55	3.23	0.20	0.37	0.75	0.06	0.12	0.31	0.23
2309	T2 A1-C1 Hor.2 Sp.1 FL.5 one each	Gegham 1	2	2	1	A1-C1	-	202	7/7/23	906	5072	43	254	15	27	76	54	3.33	0.19	0.35	0.71	0.06	0.10	0.30	0.21
2310	T2 A1-C1 Hor.2 Sp.1 FL.5 one each	Gegham 1	2	2	1	A1-C1	-	203	7/7/23	727	3954	33	221	13	24	73	51	3.04	0.17	0.33	0.70	0.06	0.11	0.33	0.23
2311	Tr-2 A0-C0 H2 Sp2 FL8 1 of 1	Gegham 1	2	2	2	A0-C0	-	204	7/7/23	682	4058	39	214	19	27	74	58	2.88	0.25	0.36	0.78	0.09	0.12	0.35	0.27
2312	Tr-2 A1-C1 H1 Sp2 FL.4 one each	Kelbadjar	2	1	2	A1-C1	-	208	7/7/23	577	5974	46	174	19	12	115	36	1.51	0.16	0.11	0.31	0.11	0.07	0.66	0.21
2313	Tr-2 A1-C1 H1 Sp2 FL.4 one each	Kelbadjar	2	1	2	A1-C1	-	210	7/7/23	607	6014	47	178	18	11	119	35	1.50	0.15	0.09	0.29	0.10	0.06	0.67	0.19
2314	Tr-2 B0 H4 Sp2 07.07.23 one each	Gegham 1	2	4	2	B0	7/7/23	211	7/7/23	738	4137	32	218	13	25	76	57	2.86	0.16	0.32	0.74	0.06	0.11	0.35	0.26
2315	Tr-2 B0 H4 Sp2 07.07.23 one each	Gegham 1	2	4	2	B0	7/7/23	212	7/7/23	707	3889	33	211	14	23	71	53	2.99	0.19	0.32	0.74	0.06	0.11	0.33	0.25
2316	Tr-2 B0 H4 Sp2 07.07.23 one each	Gegham 1	2	4	2	B0	7/7/23	213	7/7/23	680	3917	37	216	14	26	74	54	2.90	0.18	0.34	0.72	0.06	0.12	0.34	0.25
2317	Tr-2 A1 H4 Sp1 07.07.23 one each	Gegham 1	2	4	1	A1	7/7/23	214	7/7/23	697	3921	31	214	13	27	73	54	2.91	0.17	0.36	0.73	0.06	0.12	0.34	0.25
2318	Tr-2 A1 H4 Sp1 07.07.23 one each	Gegham 1	2	4	1	A1	7/7/23	215	7/7/23	687	3778	38	209	11	25	72	55	2.92	0.16	0.34	0.77	0.05	0.12	0.34	0.26
2319	Tr-2 A1 H4 Sp1 07.07.23 one each	Gegham 1	2	4	1	A1	7/7/23	216	7/7/23	736	4053	35	212	15	25	72	54	2.96	0.20	0.34	0.75	0.07	0.12	0.34	0.25
2320	Tr-2 A1 H4 Sp1 07.07.23 one each	Gegham 1	2	4	1	A1	7/7/23	217	7/7/23	710	4019	35	223	14	26	74	57	2.99	0.18	0.34	0.76	0.06	0.11	0.33	0.25
2321	Tr-2 A1 H4 Sp1 07.07.23 one each	Gegham 1	2	4	1	A1	7/7/23	218	7/7/23	746	4272	35	224	14	25	72	54	3.13	0.19	0.34	0.75	0.06	0.11	0.32	0.24
2322	Tr-2 A1 H4 Sp1 07.07.23 one each	Gegham 1	2	4	1	A1	7/7/23	219	7/7/23	784	4256	35	227	13	25	73	55	3.08	0.17	0.34	0.75	0.06	0.11	0.32	0.24
2323	Tr-2 A1 H4 Sp1 07.07.23 one each	Gegham 1	2	4	1	A1	7/7/23	220	7/7/23	832	4544	38	240	15	27	76	63	3.15	0.19	0.36	0.83	0.06	0.11	0.32	0.26
2324	Tr H5 Sp1 A5 2023 point tip	Gegham 1	2	5	1	A5	-	277	7/7/23	721	4053	36	221	14	27	75	58	2.93	0.18	0.36	0.77	0.06	0.12	0.34	0.26
2325	Tr-2 H3 Sp1 A0 (S6) 05.07.23 core frag?	Gegham 1	2	3	1	A0	7/5/23	9	7/8/23	630	4027	31	207	16	23	70	54	2.97	0.22	0.33	0.77	0.07	0.11	0.34	0.26
2326	Tr-2 H3 Sp1 A0 (S6) 05.07.23 core frag?	Gegham 1	2	3	1	A0	7/5/23	10	7/8/23	728	3978	35	215	15	23	73	55	2.96	0.20	0.31	0.76	0.07	0.11	0.34	0.26
2327	TR.2 UN A1-A4 H1.S1 29.06.23 one each	Kelbadjar	2	1	1	A1-A4	6/29/23	11	7/8/23	500	5009	41	159	17	11	104	33	1.53	0.16	0.10	0.32	0.10	0.07	0.65	0.21
2328	TR.2 UN A1-A4 H1.S1 29.06.23 one each	Gegham 1	2	1	1	A1-A4	6/29/23	12	7/8/23	681	3925	34	211	13	24	71	55	2.99	0.18	0.34	0.78	0.06	0.11	0.33	0.26
2329	TR.2 UN A1-A4 H1.S1 29.06.23 one each	Gegham 1	2	1	1	A1-A4	6/29/23	13	7/8/23	728	4103	36	219	14	27	73	55	2.98	0.18	0.36	0.75	0.06	0.12	0.34	0.25
2330	TR.2 UN A1-A4 H1.S1 29.06.23 one each	Kelbadjar	2	1	1	A1-A4	6/29/23	14	7/8/23	536	5229	42	164	18	13	108	35	1.53	0.16	0.12	0.32	0.11	0.08	0.65	0.21
2331	TR.2 UN A1-A4 H1.S1 29.06.23 one each	Gegham 1	2	1	1	A1-A4	6/29/23	15	7/8/23	849	4624	40	237	15	26	71	54	3.36	0.21	0.36	0.76	0.06	0.11	0.30	0.23
2332	TR.2 UN A1-A4 H1.S1 29.06.23 one each	Gegham 1	2	1	1	A1-A4	6/29/23	16	7/8/23	830	4387	40	239	15	26	77	57	3.10	0.19	0.33	0.73	0.06	0.11	0.32	0.24
2333	TR.2 UN A1-A4 H1.S1 29.06.23 one each	Kelbadjar	2	1	1	A1-A4	6/29/23	17	7/8/23	535	5572	45	169	18	12	119	39	1.42	0.15	0.10	0.33	0.10	0.07	0.70	0.23
2334	TR-2 H3 S2 A0 (S7) 05.07.23 1 of 1	Gegham 1	2	3	2	A0	7/5/23	18	7/8/23	658	3948	33	209	15	26	73	54	2.85	0.20	0.35	0.73	0.07	0.12	0.35	0.26
2335	TR-2 H2 Sp1 H2 Sp1 CO (S5) 04.07.23 1 of 1	Gegham 1	2	2	1	C0	7/4/23	19	7/8/23	663	3810	29	207	13	27	72	55	2.89	0.17	0.37	0.77	0.06	0.13	0.35	0.27
2336	TR.2 UN.C1 TS 29.06.23	Syunik	2	0	-	C1	6/29/23	20	7/8/23	475	5228	32	184	20	10	102	36	1.81	0.19	0.09	0.35	0.11	0.05	0.55	0.20
2337	Tr-2 H4 Sp2 A1 07.07.23 one each	Gegham 1	2	4	2	A1	7/7/23	21	7/8/23	720	3969	32	216	14	27	73	54	2.94	0.18	0.36	0.73	0.06	0.12	0.34	0.25
2338	Tr-2 H4 Sp2 A1 07.07.23 one each	Gegham 1	2	4	2	A1	7/7/23	22	7/8/23	807	4600	37	229	16	27	78	61	2.92	0.20	0.34	0.78	0.07	0.12	0.34	0.27
2339	Tr-2 H4 Sp2 A1 07.07.23 one each	Gegham 1	2	4	2	A1	7/7/23	23	7/8/23	717	4152	37	219	15	26	77	55	2.83	0.19	0.33	0.71	0.07	0.12	0.35	0.25
2340	Tr-2 H4 Sp2 A1 07.07.23 one each	Gegham 1	2	4	2	A1	7/7/23	24	7/8/23	782	4400	35	229	15	27	76	57	3.00	0.19	0.35	0.74	0.06	0.12	0.33	0.25
2341	Tr-2 H4 Sp2 A1 07.07.23 one each	Gegham 1	2	4	2	A1	7/7/23	25	7/8/23	742	4249	38	224	15	27	73	55	3.04	0.20	0.36	0.75	0.06	0.12	0.33	0.25
2342	Tr-2 H4 Sp2 A1 07.07.23 one each	Gegham 1	2	4	2	A1	7/7/23	26	7/8/23	768	4582	38	228	17	23	76	51	2.98	0.22	0.30	0.67	0.07	0.10	0.34	0.22

2343	Tr-2 H4 Sp2 A1 07.07.23 one each	Gegham 1	2	4	2	A1	7/7/23	27	7/8/23	890	4704	41	241	16	27	76	53	3.16	0.20	0.35	0.69	0.06	0.11	0.32	0.22
2344	Tr-2 H4 Sp2 A1 07.07.23 one each	Gegham 1	2	4	2	A1	7/7/23	28	7/8/23	843	4684	45	237	16	26	73	54	3.27	0.21	0.35	0.74	0.07	0.11	0.31	0.23
2345	Tr-2 H4 Sp2 A1 07.07.23 one each	Gegham 1	2	4	2	A1	7/7/23	30	7/8/23	865	4716	45	237	17	24	74	59	3.19	0.22	0.32	0.80	0.07	0.10	0.31	0.25
2346	TR.2 UN B0 H1 Sp1 (S1) 02.07.23 1 of 1	Syunik	2	1	1	B0	7/2/23	36	7/8/23	481	5273	36	180	21	12	103	33	1.75	0.20	0.12	0.32	0.11	0.07	0.57	0.18
2347	TR.2 B0 H2 Sp2 02.07.23 1 of 1	Gegham 1	2	2	2	B0	7/2/23	37	7/8/23	754	4362	40	231	16	27	74	55	3.10	0.21	0.36	0.74	0.07	0.12	0.32	0.24
2348	TR.2 UN A2-A3 30.06.23 on each	Kelbadjar	2	-	-	A2-A3	6/30/23	38	7/8/23	531	5177	41	160	19	11	114	36	1.40	0.16	0.09	0.32	0.12	0.07	0.72	0.23
2349	TR.2 UN A2-A3 30.06.23 on each	Gegham 1	2	-	-	A2-A3	6/30/23	39	7/8/23	712	3990	39	219	15	25	72	55	3.06	0.20	0.34	0.77	0.07	0.11	0.33	0.25
2350	TR.2 UN A2-A3 30.06.23 on each	Arteni	2	-	-	A2-A3	6/30/23	40	7/8/23	653	5154	46	137	40	24	89	26	1.55	0.45	0.27	0.30	0.29	0.17	0.65	0.19
2351	TR.2 UN A2-A3 30.06.23 on each	Gegham 1	2	-	-	A2-A3	6/30/23	41	7/8/23	663	3617	35	206	11	25	72	54	2.88	0.16	0.34	0.75	0.06	0.12	0.35	0.26
2352	TR.2 UN A2-A3 30.06.23 on each	Gegham 1	2	-	-	A2-A3	6/30/23	42	7/8/23	783	4431	44	236	15	24	73	55	3.22	0.20	0.32	0.75	0.06	0.10	0.31	0.23
2353	Tr-2 H4 Sp1 C1 07.07.23 one each	Gegham 1	2	4	1	C1	7/7/23	44	7/8/23	666	3826	32	208	14	25	73	55	2.87	0.19	0.34	0.76	0.06	0.12	0.35	0.27
2354	Tr-2 H4 Sp1 C1 07.07.23 one each	Gegham 1	2	4	1	C1	7/7/23	45	7/8/23	817	4539	36	236	17	23	71	57	3.35	0.23	0.32	0.80	0.07	0.10	0.30	0.24
2355	Tr-2 H4 Sp1 C1 07.07.23 one each	Gegham 1	2	4	1	C1	7/7/23	46	7/8/23	696	3882	36	208	15	25	73	55	2.87	0.20	0.34	0.76	0.07	0.12	0.35	0.27
2356	Tr-2 H4 Sp1 C1 07.07.23 one each	Gegham 1	2	4	1	C1	7/7/23	47	7/8/23	731	4133	35	214	13	26	72	54	2.99	0.17	0.36	0.75	0.06	0.12	0.33	0.25
2357	Tr-2 H4 Sp1 C1 07.07.23 one each	Gegham 1	2	4	1	C1	7/7/23	48	7/8/23	752	4261	39	224	16	26	73	57	3.04	0.21	0.35	0.77	0.07	0.11	0.33	0.25
2358	Tr-2 H4 Sp1 C1 07.07.23 one each	Gegham 1	2	4	1	C1	7/7/23	49	7/8/23	979	5646	48	267	17	24	76	55	3.50	0.22	0.31	0.72	0.06	0.09	0.29	0.21
2359	Tr-2 H4 Sp1 C1 07.07.23 one each	Gegham 1	2	4	1	C1	7/7/23	50	7/8/23	951	5209	46	257	16	25	79	55	3.24	0.20	0.31	0.70	0.06	0.10	0.31	0.22
2360	Tr-2 H4 Sp1 C1 07.07.23 one each	Gegham 1	2	4	1	C1	7/7/23	51	7/8/23	738	4181	33	226	13	24	74	55	3.03	0.17	0.32	0.74	0.06	0.11	0.33	0.24
2361	Tr-2 H4 Sp1 C1 07.07.23 one each	Gegham 1	2	4	1	C1	7/7/23	52	7/8/23	733	5025	39	221	17	25	73	57	3.00	0.22	0.34	0.77	0.07	0.11	0.33	0.26
2362	Tr-2 H4 Sp1 C1 07.07.23 one each	Gegham 1	2	4	1	C1	7/7/23	53	7/8/23	742	3992	35	220	14	25	77	57	2.85	0.17	0.32	0.73	0.06	0.11	0.35	0.26
2363	Tr-2 H4 Sp1 C1 07.07.23 one each	Gegham 1	2	4	1	C1	7/7/23	54	7/8/23	784	4237	36	228	11	27	70	55	3.27	0.16	0.38	0.79	0.05	0.12	0.31	0.24
2364	Tr-2 H4 Sp1 C1 07.07.23 one each	Arteni	2	4	1	C1	7/7/23	55	7/8/23	653	4731	37	138	32	27	86	31	1.61	0.37	0.31	0.36	0.23	0.19	0.62	0.22
2365	Tr-2 H4 Sp1 C1 07.07.23 one each	Gegham 1	2	4	1	C1	7/7/23	56	7/8/23	852	4575	41	231	13	26	75	58	3.06	0.17	0.34	0.77	0.05	0.11	0.33	0.25
2366	Tr-2 H4 Sp1 C1 07.07.23 one each	Gegham 1	2	4	1	C1	7/7/23	57	7/8/23	971	5230	46	252	15	25	73	58	3.47	0.20	0.34	0.80	0.06	0.10	0.29	0.23
2367	Tr-2 H4 Sp1 C1 07.07.23 one each	Gegham 1	2	4	1	C1	7/7/23	58	7/8/23	1140	5954	57	273	14	27	78	55	3.49	0.17	0.34	0.71	0.05	0.10	0.29	0.20
2368	Tr-2 H4 Sp2 C1 07.07.23 one each	Syunik	2	4	2	C1	7/7/23	59	7/8/23	597	4552	39	218	9	8	97	44	2.24	0.10	0.08	0.46	0.04	0.04	0.45	0.20
2369	Tr-2 H4 Sp2 C1 07.07.23 one each	Gegham 1	2	4	2	C1	7/7/23	60	7/8/23	728	4082	35	220	14	24	72	55	3.07	0.19	0.33	0.77	0.06	0.11	0.33	0.25
2370	Tr-2 H4 Sp2 C1 07.07.23 one each	Gegham 1	2	4	2	C1	7/7/23	61	7/8/23	688	3969	36	208	14	26	75	54	2.76	0.18	0.34	0.71	0.06	0.12	0.36	0.26
2371	Tr-2 H4 Sp2 C1 07.07.23 one each	Gegham 1	2	4	2	C1	7/7/23	62	7/8/23	687	3904	38	217	14	25	73	55	2.95	0.18	0.34	0.75	0.06	0.11	0.34	0.25
2372	Tr-2 H4 Sp2 C1 07.07.23 one each	Gegham 1	2	4	2	C1	7/7/23	63	7/8/23	661	3826	31	208	13	27	71	55	2.95	0.18	0.39	0.78	0.06	0.13	0.34	0.27
2373	Tr-2 H4 Sp2 C1 07.07.23 one each	Gegham 1	2	4	2	C1	7/7/23	64	7/8/23	682	3840	43	214	16	25	74	55	2.88	0.21	0.33	0.74	0.07	0.12	0.35	0.26
2374	Tr-2 H4 Sp2 C1 07.07.23 one each	Gegham 1	2	4	2	C1	7/7/23	65	7/8/23	823	4643	43	239	14	25	72	58	3.34	0.19	0.34	0.81	0.06	0.10	0.30	0.24
2375	Tr-2 H4 Sp2 C1 07.07.23 one each	Khorapor	2	4	2	C1	7/7/23	66	7/8/23	499	4278	31	227	7	16	82	39	2.76	0.09	0.20	0.47	0.03	0.07	0.36	0.17
2376	Tr-2 H4 Sp2 C1 07.07.23 one each	Gegham 1	2	4	2	C1	7/7/23	67	7/8/23	859	4859	41	246	16	27	76	53	3.22	0.20	0.35	0.69	0.06	0.11	0.31	0.21
2377	Tr-2 H4 Sp2 C1 07.07.23 one each	Gegham 1	2	4	2	C1	7/7/23	68	7/8/23	744	4300	36	225	13	24	77	55	2.91	0.16	0.31	0.71	0.06	0.11	0.34	0.25
2378	Tr-2 H4 Sp2 C1 07.07.23 one each	Gegham 1	2	4	2	C1	7/7/23	69	7/8/23	941	5269	47	259	14	23	73	53	3.57	0.19	0.31	0.72	0.05	0.09	0.28	0.20
2379	Tr-2 H4 Sp2 C1 07.07.23 one each	Gegham 1	2	4	2	C1	7/7/23	70	7/8/23	910	4948	45	249	16	24	73	55	3.39	0.21	0.32	0.75	0.06	0.10	0.30	0.22
2380	Tr-2 H4 Sp2 C1 07.07.23 one each	Gegham 1	2	4	2	C1	7/7/23	71	7/8/23	801	4362	37	233	14	26	73	57	3.16	0.18	0.35	0.77	0.06	0.11	0.32	0.24
2381	Tr-2 H4 Sp2 C1 07.07.23 one each	Gegham 1	2	4	2	C1	7/7/23	72	7/8/23	724	3869	33	219	14	24	73	54	3.02	0.19	0.33	0.74	0.06	0.11	0.33	0.25
2382	TR.2 A5 H1.S2 collapse 30.06 one each	Gegham 1	2	1	2	A5	6/30/23	73	7/8/23	611	3550	33	198	14	25	71	51	2.81	0.19	0.35	0.72	0.07	0.12	0.36	0.26
2383	TR.2 A5 H1.S2 collapse 30.06 one each	Gegham 1	2	1	2	A5	6/30/23	74	7/8/23	686	3956	32	213	15	25	73	55	2.90	0.20	0.34	0.75	0.07	0.12	0.34	0.26
2384	TR.2 A5 H1.S2 collapse 30.06 one each	Gegham 1	2	1	2	A5	6/30/23	75	7/8/23	729	4035	35	210	14	24	73	55	2.86	0.18	0.32	0.75	0.06	0.11	0.35	0.26
2385	TR.2 A5 H1.S2 collapse 30.06 one each	Kelbadjar	2	1	2	A5	6/30/23	76	7/8/23	536	5736	43	170	19	11	116	35	1.47	0.16	0.10	0.30	0.11	0.07	0.68	0.20
2386	TR.2 A5 H1.S2 collapse 30.06 one each	Gegham 1	2	1	2	A5	6/30/23	78	7/8/23	726	4133	40	227	14	23	73	57	3.08	0.18	0.31	0.77	0.06	0.10	0.32	0.25
2387	TR.2 A5 H1.S2 collapse 30.06 one each	Gegham 1	2	1	2	A5	6/30/23	79	7/8/23	753	4349	37	233	15	27	73	54	3.16	0.20	0.36	0.73	0.06	0.11	0.32	0.23
2388	TR.2 A5 H1.S2 collapse 30.06 one each	Gegham 1	2	1	2	A5	6/30/23	80	7/8/23	718	4158	33	216	13	25	73	55	2.94	0.17	0.34	0.75	0.06	0.11	0.34	0.26
2389	T2 A1 to C1 Hor.0 FL.1 1 of 1	Kelbadjar	2	0	-	A1-C1	-	81	7/8/23	604	6283	46	176	18	12	113	37	1.56	0.15	0.11	0.33	0.10	0.07	0.64	0.21
2390	T2 A1-C1 H2 S2 FL.7 one each	Hatis	2	2	2	A1-C1	-	82	7/8/23	662	10640	51	133	120	27	98	29	1.36	1.22	0.27	0.30	0.90	0.20	0.74	0.22
2391	T2 A1-C1 H2 S2 FL.7 one each	Kelbadjar	2	2	2	A1-C1	-	83	7/8/23	567	6123	48	172	19	11	117	35	1.47	0.16	0.09	0.30	0.11	0.06	0.68	0.20
2392	T2 A1-C1 H2 S2 FL.7 one each	Gegham 1	2	2	2	A1-C1	-	84	7/8/23	842	4475	47	235	15	27	74	55	3.16	0.19	0.36	0.74	0.06	0.11	0.32	0.23
2393	TR-2 A1-C1 H3 Sp1 Flot one each	Gegham 1	2	3	1	A1-C1	-	86	7/8/23	707	4046	32	216	15	27	76	57	2.83	0.19	0.35	0.74	0.07	0.12	0.35	0.26

2394	TR-2 A1-C1 H3 Sp1 Flot one each	Gegham 1	2	3	1	A1-C1	-	87	7/8/23	715	5221	41	220	18	27	73	51	3.03	0.24	0.37	0.70	0.08	0.12	0.33	0.23
2395	TR-2 A1-C1 H3 Sp1 Flot one each	Gegham 1	2	3	1	A1-C1	-	88	7/8/23	711	3891	35	232	15	27	74	55	3.11	0.19	0.36	0.74	0.06	0.11	0.32	0.24
2396	TR-2 A1-C1 H3 Sp1 Flot one each	Syunik	2	3	1	A1-C1	-	89	7/8/23	577	5879	44	198	23	9	106	33	1.88	0.21	0.08	0.32	0.11	0.04	0.53	0.17
2397	TR-2 A0-C-0 H1 Sp2 flt? one each	Gegham 1	2	1	2	A0-C0	-	91	7/8/23	681	4001	37	216	16	27	73	53	2.94	0.21	0.37	0.71	0.07	0.13	0.34	0.24
2398	TR-2 A0-C-0 H1 Sp2 flt? one each	Gegham 1	2	1	2	A0-C0	-	92	7/8/23	899	4664	46	245	14	27	81	59	3.02	0.17	0.33	0.73	0.06	0.11	0.33	0.24
2399	TR2 Unit A1 H5 08.07.23 one each	Gegham 1	2	5	-	A1	7/8/23	94	7/8/23	727	3857	35	205	11	25	71	57	2.91	0.16	0.35	0.80	0.06	0.12	0.34	0.28
2400	TR2 Unit A1 H5 08.07.23 one each	Gegham 1	2	5	-	A1	7/8/23	95	7/8/23	697	3994	35	216	14	27	73	54	2.98	0.19	0.37	0.74	0.06	0.12	0.34	0.25
2401	TR2 Unit A1 H5 08.07.23 one each	Gegham 1	2	5	-	A1	7/8/23	96	7/8/23	685	3807	34	209	13	25	73	54	2.85	0.17	0.34	0.73	0.06	0.12	0.35	0.26
2402	TR2 Unit A1 H5 08.07.23 one each	Gegham 1	2	5	-	A1	7/8/23	97	7/8/23	668	3915	32	212	14	26	69	57	3.08	0.20	0.37	0.82	0.06	0.12	0.32	0.27
2403	TR2 Unit A1 H5 08.07.23 one each	Gegham 1	2	5	-	A1	7/8/23	98	7/8/23	652	3885	31	211	11	24	72	58	2.95	0.16	0.33	0.81	0.05	0.11	0.34	0.27
2404	TR2 Unit A1 H5 08.07.23 one each	Gegham 1	2	5	-	A1	7/8/23	99	7/8/23	818	4440	38	237	15	25	73	57	3.27	0.20	0.34	0.78	0.06	0.10	0.31	0.24
2405	TR2 Unit A1 H5 08.07.23 one each	Gegham 1	2	5	-	A1	7/8/23	100	7/8/23	661	4526	35	204	18	23	79	47	2.58	0.22	0.29	0.59	0.09	0.11	0.39	0.23
2406	TR2 Unit A1 H5 08.07.23 one each	Gegham 1	2	5	-	A1	7/8/23	101	7/8/23	683	3992	34	214	14	25	73	57	2.95	0.19	0.34	0.78	0.06	0.12	0.34	0.26
2407	TR2 Unit A1 H5 08.07.23 one each	Gegham 1	2	5	-	A1	7/8/23	102	7/8/23	685	3807	32	209	13	26	72	55	2.92	0.17	0.36	0.77	0.06	0.12	0.34	0.26
2408	TR2 Unit A1 H5 08.07.23 one each	Gegham 1	2	5	-	A1	7/8/23	103	7/8/23	802	4407	39	237	13	24	75	54	3.15	0.17	0.31	0.71	0.05	0.10	0.32	0.23
2409	TR2 Unit A1 H5 08.07.23 one each	Gegham 1	2	5	-	A1	7/8/23	104	7/8/23	642	3755	34	203	14	27	76	55	2.66	0.18	0.35	0.72	0.07	0.13	0.38	0.27
2410	TR2 Unit A1 H5 08.07.23 one each	Gegham 1	2	5	-	A1	7/8/23	105	7/8/23	808	4349	34	230	14	27	74	57	3.08	0.18	0.37	0.76	0.06	0.12	0.32	0.25
2411	TR2 Unit A1 H5 08.07.23 one each	Gegham 1	2	5	-	A1	7/8/23	106	7/8/23	650	3642	36	210	15	24	68	53	3.10	0.21	0.35	0.77	0.07	0.11	0.32	0.25
2412	TR2 Unit A1 H5 08.07.23 one each	Gegham 1	2	5	-	A1	7/8/23	107	7/8/23	772	4317	38	227	14	24	73	54	3.08	0.18	0.32	0.73	0.06	0.10	0.32	0.24
2413	TR2 Unit A1 H5 08.07.23 one each	Gegham 1	2	5	-	A1	7/8/23	108	7/8/23	693	3897	33	209	13	24	68	55	3.08	0.18	0.35	0.81	0.06	0.11	0.32	0.26
2414	TR2 Unit A1 H5 08.07.23 one each	Gegham 1	2	5	-	A1	7/8/23	109	7/8/23	859	4953	42	243	14	25	73	53	3.31	0.18	0.34	0.71	0.06	0.10	0.30	0.22
2415	TR2 Unit A1 H5 08.07.23 one each	Gegham 1	2	5	-	A1	7/8/23	110	7/8/23	889	5076	45	250	16	26	76	57	3.28	0.20	0.34	0.74	0.06	0.10	0.31	0.23
2416	TR2 Unit A1 H5 08.07.23 one each	Gegham 1	2	5	-	A1	7/8/23	111	7/8/23	788	4218	36	233	15	25	76	55	3.05	0.19	0.32	0.72	0.06	0.11	0.33	0.24
2417	TR2 Unit A1 H5 08.07.23 one each	Gegham 1	2	5	-	A1	7/8/23	112	7/8/23	824	4572	39	238	16	25	73	54	3.24	0.21	0.34	0.73	0.07	0.10	0.31	0.23
2418	TR2 Unit A1 H5 08.07.23 one each	Gegham 1	2	5	-	A1	7/8/23	113	7/8/23	727	4028	34	216	13	25	71	54	3.06	0.18	0.35	0.76	0.06	0.11	0.33	0.25
2419	TR2 Unit A1 H5 08.07.23 one each	Gegham 1	2	5	-	A1	7/8/23	115	7/8/23	799	4521	41	237	15	27	75	54	3.15	0.19	0.35	0.71	0.06	0.11	0.32	0.23
2420	TR2 Unit A1 H5 08.07.23 one each	Gegham 1	2	5	-	A1	7/8/23	116	7/8/23	848	4126	35	228	13	24	78	57	2.91	0.16	0.30	0.72	0.05	0.10	0.34	0.25
2421	TR2 Unit A1 H5 08.07.23 one each	Gegham 1	2	5	-	A1	7/8/23	117	7/8/23	768	4126	39	228	15	27	73	55	3.10	0.20	0.37	0.75	0.06	0.12	0.32	0.24
2422	TR2 Unit A1 H5 08.07.23 one each	Gegham 1	2	5	-	A1	7/8/23	118	7/8/23	767	4358	38	225	14	26	76	55	2.94	0.18	0.34	0.72	0.06	0.11	0.34	0.25
2423	TR2 Unit A1 H5 08.07.23 one each	Gegham 1	2	5	-	A1	7/8/23	119	7/8/23	739	3917	32	218	14	26	73	55	3.00	0.19	0.35	0.76	0.06	0.12	0.33	0.25
2424	TR2 Unit A1 H5 08.07.23 one each	Gegham 1	2	5	-	A1	7/8/23	120	7/8/23	811	4483	41	235	15	28	73	55	3.20	0.20	0.39	0.75	0.06	0.12	0.31	0.23
2425	SR2 (TR2?) H5 B0 08.07.23 1 of 1	Gegham 1	2	5	-	B0	7/8/23	121	7/8/23	663	3770	35	207	14	24	73	51	2.82	0.18	0.32	0.70	0.07	0.11	0.35	0.25
2426	TR2 Unit B1 H5 08.07.23 one each	Gegham 1	2	5	-	B1	7/8/23	122	7/8/23	678	3982	36	209	13	25	73	54	2.88	0.17	0.34	0.74	0.06	0.12	0.35	0.26
2427	TR2 Unit B1 H5 08.07.23 one each	Gegham 1	2	5	-	B1	7/8/23	123	7/8/23	759	4338	40	231	14	26	74	53	3.10	0.18	0.34	0.71	0.06	0.11	0.32	0.23

XLSTAT 2022.4.1.1375 - Discriminant Analysis (DA) - Start time: 12/23/2023 at 14:37:24 / End time: 12/23/2023 at 14:37:39
Y / Qualitative: Workbook = Yegh1 2022-2023 arts and geo data 2023-Dec-23f.xlsx / Sheet = geo reference collection / Range = 'geo reference collection'!$C:$C / 261 rows and 1 column
X / Quantitative: Workbook = Yegh1 2022-2023 arts and geo data 2023-Dec-23f.xlsx / Sheet = geo reference collection / Range = 'geo reference collection'!$D:$S / 261 rows and 16 columns
Observation labels: Workbook = Yegh1 2022-2023 arts and geo data 2023-Dec-23f.xlsx / Sheet = geo reference collection / Range = 'geo reference collection'!$A:$A / 261 rows and 1 column
Prediction / X / Quantitative: Workbook = Yegh1 2022-2023 arts and geo data 2023-Dec-23f.xlsx / Sheet = artifacts - analyzed order / Range = 'artifacts - analyzed order'!$K:$Z / 2427 rows and 16 columns
Prediction / Observation labels: Workbook = Yegh1 2022-2023 arts and geo data 2023-Dec-23f.xlsx / Sheet = artifacts - analyzed order / Range = 'artifacts - analyzed order'!$A:$A / 2427 rows and 1 column
Filtering / N first rows: Number of observations: 100
Within-class covariance matrices are assumed to be equal
Prior probabilities are taken into account
Filter components:Maximum number: 5
Significance level (%): 5
Validation: Validation set: Random / Number of observations: 1
Classes weight correction: Automatic
Seed (random numbers): 1337947242

Summary statistics (Quantitative data)

Summary statistics (Quantitative data):

Variable	Observations	Obs. with missing data	Obs. without	Minimum	Maximum	Mean	Std. deviation
Mn	260	0	260	237.066	859.119	549.335	109.363
Fe	260	0	260	3518.240	12522.405	6083.603	2128.010
Zn	260	0	260	27.194	82.208	40.817	9.893
Rb	260	0	260	85.804	221.814	145.839	38.940
Sr	260	0	260	5.486	241.003	73.532	67.726
Y	260	0	260	5.817	56.648	19.325	10.978
Zr	260	0	260	50.800	288.121	116.741	53.515
Nb	260	0	260	4.486	56.618	28.859	11.997
Rb/Zr	260	0	260	0.410	3.582	1.492	0.770
Sr/Zr	260	0	260	0.050	1.766	0.624	0.544
Y/Zr	260	0	260	0.039	0.544	0.183	0.114
Nb/Zr	260	0	260	0.042	1.022	0.306	0.220
Sr/Rb	260	0	260	0.025	2.774	0.651	0.704
Y/Rb	260	0	260	0.030	0.368	0.138	0.071
Zr/Rb	260	0	260	0.279	2.442	0.873	0.467
Nb/Rb	260	0	260	0.033	0.306	0.196	0.056

Summary statistics (Qualitative data):

Variable	Categories	Counts	Frequencies	%
Group	Aghvorik	8	8	3.077
	Arteni	27	27	10.385
	Chikiani	25	25	9.615
	Gegham 1	21	21	8.077
	Gegham 2	6	6	2.308
	Gutansar	24	24	9.231
	Hatis	35	35	13.462
	Kars-Arpaçay	9	9	3.462
	Kelbadjar	3	3	1.154
	Khorapor	6	6	2.308
	Meydan Dağ	9	9	3.462
	Sarıkamış	13	13	5.000
	Syunik	47	47	18.077
	Tsaghkunyat	27	27	10.385

Summary statistics (Quantitative data / Validation):

Variable	Observations	Obs. with missing data	Obs. without	Minimum	Maximum	Mean	Std. deviation
Mn	1	0	1	448.988	448.988	448.988	

Fe	1	0	1	6189.583	6189.583	6189.583
Zn	1	0	1	29.944	29.944	29.944
Rb	1	0	1	93.576	93.576	93.576
Sr	1	0	1	174.858	174.858	174.858
Y	1	0	1	9.583	9.583	9.583
Zr	1	0	1	103.748	103.748	103.748
Nb	1	0	1	22.321	22.321	22.321
Rb/Zr	1	0	1	0.902	0.902	0.902
Sr/Zr	1	0	1	1.685	1.685	1.685
Y/Zr	1	0	1	0.092	0.092	0.092
Nb/Zr	1	0	1	0.215	0.215	0.215
Sr/Rb	1	0	1	1.869	1.869	1.869
Y/Rb	1	0	1	0.102	0.102	0.102
Zr/Rb	1	0	1	1.109	1.109	1.109
Nb/Rb	1	0	1	0.239	0.239	0.239

Summary statistics (Qualitative data / Validation):

Variable	Categories	Counts	Frequencies	%
Group	Aghvorik	0	0	0.000
	Arteni	0	0	0.000
	Chikiani	0	0	0.000
	Gegham 1	0	0	0.000
	Gegham 2	0	0	0.000
	Gutansar	0	0	0.000
	Hatis	0	0	0.000
	Kars-Arpaçay	0	0	0.000
	Kelbadjar	0	0	0.000
	Khorapor	0	0	0.000
	Meydan Dağ	0	0	0.000
	Sarıkamış	0	0	0.000
	Syunik	0	0	0.000
	Tsaghkunyat	1	1	100.000

Correlation matrix:

Variables	Mn	Fe	Zn	Rb	Sr	Y	Zr	Nb	Rb/Zr	Sr/Zr	Y/Zr	Nb/Zr	Sr/Rb	Y/Rb	Zr/Rb	Nb/Rb
Mn	**1**	-0.215	0.225	0.353	-0.342	0.441	-0.076	0.760	0.463	-0.385	0.663	0.697	-0.370	0.306	-0.229	0.744
Fe	-0.215	**1**	0.496	-0.471	0.651	0.241	0.847	-0.491	-0.809	0.410	-0.449	-0.690	0.600	0.397	0.917	-0.247
Zn	0.225	0.496	**1**	0.081	-0.104	0.754	0.754	-0.089	-0.377	-0.241	0.066	-0.303	-0.143	0.661	0.466	-0.156
Rb	0.353	-0.471	0.081	**1**	-0.790	0.117	-0.131	0.730	0.724	-0.808	0.193	0.601	-0.785	-0.290	-0.580	0.136
Sr	-0.342	0.651	-0.104	-0.790	**1**	-0.298	0.300	-0.538	-0.687	0.918	-0.475	-0.549	0.983	-0.021	0.682	-0.044
Y	0.441	0.241	0.754	0.117	-0.298	**1**	0.516	0.105	-0.145	-0.375	0.558	-0.013	-0.330	0.894	0.233	0.070
Zr	-0.076	0.847	0.754	-0.131	0.300	0.516	**1**	-0.274	-0.689	0.022	-0.345	-0.588	0.255	0.513	0.840	-0.219
Nb	0.760	-0.491	-0.089	0.730	-0.538	0.105	-0.274	**1**	0.736	-0.562	0.437	0.863	-0.542	-0.182	-0.532	0.764
Rb/Zr	0.463	-0.809	-0.377	0.724	-0.687	-0.145	-0.689	0.736	**1**	-0.559	0.523	0.921	-0.643	-0.411	-0.842	0.332
Sr/Zr	-0.385	0.410	-0.241	-0.808	0.918	-0.375	0.022	-0.562	-0.559	**1**	-0.385	-0.465	0.912	-0.095	0.421	-0.061
Y/Zr	0.663	-0.449	0.066	0.193	-0.475	0.558	-0.345	0.437	0.523	-0.385	**1**	0.639	-0.463	0.463	-0.417	0.400
Nb/Zr	0.697	-0.690	-0.303	0.601	-0.549	-0.013	-0.588	0.863	0.921	-0.465	0.639	**1**	-0.511	-0.244	-0.697	0.631
Sr/Rb	-0.370	0.600	-0.143	-0.785	0.983	-0.330	0.255	-0.542	-0.643	0.912	-0.463	-0.511	**1**	-0.052	0.665	-0.054
Y/Rb	0.306	0.397	0.661	-0.290	-0.021	0.894	0.513	-0.182	-0.411	-0.095	0.463	-0.244	-0.052	**1**	0.446	0.026
Zr/Rb	-0.229	0.917	0.466	-0.580	0.682	0.233	0.840	-0.532	-0.842	0.421	-0.417	-0.697	0.665	0.446	**1**	-0.211
Nb/Rb	0.744	-0.247	-0.156	0.136	-0.044	0.070	-0.219	0.764	0.332	-0.061	0.400	0.631	-0.054	0.026	-0.211	**1**

Discriminant Analysis:

Means by class:

Class \ Variable	Mn	Fe	Zn	Rb	Sr	Y	Zr	Nb	Rb/Zr	Sr/Zr	Y/Zr	Nb/Zr	Sr/Rb	Y/Rb	Zr/Rb	Nb/Rb
Aghvorik	487.009	12216.785	49.199	101.227	194.276	15.936	243.209	14.775	0.416	0.799	0.065	0.061	1.920	0.157	2.403	0.146
Arteni	660.633	3931.867	41.423	138.589	21.856	29.315	76.889	34.566	1.838	0.278	0.392	0.463	0.163	0.210	0.563	0.248

Chikiani	474.319	5558.993	44.285	128.006	86.063	14.891	95.881	15.900	1.352	0.899	0.157	0.169	0.674	0.116	0.751	0.124
Gegham 1	756.126	3659.517	34.704	195.352	11.356	23.568	61.155	53.287	3.220	0.187	0.389	0.884	0.058	0.121	0.313	0.274
Gegham 2	570.324	5956.049	43.087	112.197	53.258	26.055	114.306	28.266	0.982	0.466	0.228	0.247	0.475	0.232	1.019	0.252
Gutansar	636.601	8208.530	43.163	141.868	125.166	23.074	171.115	36.783	0.829	0.731	0.135	0.215	0.882	0.163	1.206	0.259
Hatis	511.852	7506.633	37.463	107.732	134.426	15.661	102.478	20.283	1.071	1.297	0.155	0.201	1.267	0.146	0.960	0.188
Kars-Arpaçay	660.217	8440.194	65.602	130.493	28.982	44.620	221.936	22.778	0.593	0.128	0.202	0.103	0.227	0.342	1.706	0.174
Kelbadjar	482.231	4969.487	40.642	151.542	15.508	10.524	106.900	33.296	1.420	0.145	0.099	0.312	0.102	0.069	0.706	0.220
Khorapor	453.559	3903.690	29.944	214.528	7.491	12.877	78.535	35.354	2.732	0.096	0.164	0.450	0.035	0.060	0.366	0.165
Meydan Dağ	557.719	9676.309	77.114	204.651	23.080	54.033	282.763	28.571	0.724	0.082	0.191	0.101	0.113	0.264	1.382	0.140
Sarıkamış	356.835	5994.360	34.035	135.052	25.068	24.354	111.021	9.024	1.254	0.233	0.224	0.083	0.186	0.180	0.820	0.067
Syunik	530.256	4515.069	36.948	193.765	11.393	8.982	93.247	37.383	2.086	0.121	0.096	0.403	0.060	0.047	0.484	0.193
Tsaghkunyats	446.356	6482.603	33.103	96.095	190.151	9.060	115.199	19.425	0.864	1.656	0.082	0.175	2.012	0.094	1.218	0.203

Unidimensional test of equality of the means of the classes:

	Lambda	F	DF1	DF2	p-value
Mn	0.134	122.214	13	246	<0.0001
Fe	0.065	273.916	13	246	<0.0001
Zn	0.050	360.628	13	246	<0.0001
Rb	0.034	530.859	13	246	<0.0001
Sr	0.036	512.889	13	246	<0.0001
Y	0.024	754.371	13	246	<0.0001
Zr	0.029	627.992	13	246	<0.0001
Nb	0.056	319.782	13	246	<0.0001
Rb/Zr	0.040	457.856	13	246	<0.0001
Sr/Zr	0.011	1650.657	13	246	<0.0001
Y/Zr	0.102	166.126	13	246	<0.0001
Nb/Zr	0.056	318.774	13	246	<0.0001
Sr/Rb	0.055	324.658	13	246	<0.0001
Y/Rb	0.019	957.361	13	246	<0.0001
Zr/Rb	0.044	411.091	13	246	<0.0001
Nb/Rb	0.079	220.265	13	246	<0.0001

Wilks' Lambda test (Rao's approximation):

Lambda	0.000	
F (Observed	79.939	
F (Critical val	1.175	
DF1	208	
DF2	2373.330	
p-value (Twc	**<0.0001**	***
alpha	0.05	

*Signification codes: 0 < "***" < 0.001 < "**" < 0.01 < "*" < 0.05 < "." < 0.1 < " " < 1*

Test interpretation:
H0: The means vectors of the 14 classes are equal.
Ha: At least one of the means vector is different from another.
As the computed p-value is lower than the significance level alpha=0.05, one should reject the null hypothesis H0, and accept the alternative hypothesis Ha.

Pillai's trace:

Trace	4.906	
F (Observed	9.207	
F (Critical val	1.173	
DF1	208	
DF2	3159.000	
p-value (Twc	**<0.0001**	***
alpha	0.05	

*Signification codes: 0 < "***" < 0.001 < "**" < 0.01 < "*" < 0.05 < "." < 0.1 < " " < 1*

Test interpretation:
H0: The means vectors of the 14 classes are equal.

Ha: At least one of the means vector is different from another.
As the computed p-value is lower than the significance level alpha=0.05, one should reject the null hypothesis H0, and accept the alternative hypothesis Ha.

Hotelling-Lawley trace:

Trace	442.654	
F (Observed	487.974	
F (Critical val	1.180	
DF1	208	
DF2	1552.602	
p-value (Twc	**<0.0001**	***
alpha	0.05	

Signification codes: 0 < "***" < 0.001 < "**" < 0.01 < "*" < 0.05 < "." < 0.1 < " " < 1

Test interpretation:
H0: The means vectors of the 14 classes are equal.
Ha: At least one of the means vector is different from another.
As the computed p-value is lower than the significance level alpha=0.05, one should reject the null hypothesis H0, and accept the alternative hypothesis Ha.

Roy's greatest root:

Root	206.688	
F (Observed	3139.081	
F (Critical val	1.685	
DF1	16	
DF2	243	
p-value (Twc	**<0.0001**	***
alpha	0.05	

Signification codes: 0 < "***" < 0.001 < "**" < 0.01 < "*" < 0.05 < "." < 0.1 < " " < 1

Test interpretation:
H0: The means vectors of the 14 classes are equal.
Ha: At least one of the means vector is different from another.
As the computed p-value is lower than the significance level alpha=0.05, one should reject the null hypothesis H0, and accept the alternative hypothesis Ha.

Sum of weights, prior probabilities and logarithms of determinants for each class:

Classes	Sum of weights	Prior probabilities	Log(Determinant)
Aghvorik	18.571	0.071	-380.253
Arteni	18.571	0.071	-78.674
Chikiani	18.571	0.071	-97.147
Gegham 1	18.571	0.071	-97.592
Gegham 2	18.571	0.071	
Gutansar	18.571	0.071	-117.577
Hatis	18.571	0.071	-80.091
Kars-Arpaçay	18.571	0.071	-309.693
Kelbadjar	18.571	0.071	
Khorapor	18.571	0.071	-441.357
Meydan Dağ	18.571	0.071	-352.101
Sarıkamış	18.571	0.071	
Syunik	18.571	0.071	-87.188
Tsaghkunyats	18.571	0.071	-74.378

Multicolinearity statistics:

Statistics	Mn	Fe	Zn	Rb	Sr	Y	Zr	Nb	Rb/Zr	Sr/Zr	Y/Zr	Nb/Zr	Sr/Rb	Y/Rb	Zr/Rb	Nb/Rb
Tolerance	0.088	0.038	0.082	0.002	0.003	0.001	0.001	0.002	0.002	0.013	0.013	0.003	0.003	0.003	0.001	0.011
VIF	11.317	26.151	12.268	410.874	337.181	749.758	1177.581	408.826	434.952	78.739	76.067	311.289	293.261	334.500	788.621	92.605

Between-classes covariance matrix:

	Mn	Fe	Zn	Rb	Sr	Y	Zr	Nb	Rb/Zr	Sr/Zr	Y/Zr	Nb/Zr	Sr/Rb	Y/Rb	Zr/Rb	Nb/Rb
Mn	11336.635	-25284.220	382.864	931.689	-1745.159	619.395	299.634	881.421	22.169	-14.617	6.688	13.092	-20.491	3.441	-4.349	4.565
Fe	-25284.220	6122425.33	20224.98	-38921.82	100606.79	11829.69	152124.84	-14631.285	-1648.760	339.964	-116.022	-388.340	966.944	96.772	1340.130	-46.679
Zn	382.864	20224.979	169.402	42.386	-75.237	145.940	768.564	-21.608	-5.878	-1.727	-0.027	-1.283	-1.042	0.818	4.278	-0.152
Rb	931.689	-38921.816	42.386	1595.278	-1961.296	104.503	-230.969	307.073	22.007	-14.314	0.995	4.767	-20.170	-0.748	-12.009	0.084
Sr	-1745.159	100606.790	-75.237	-1961.296	4568.598	-295.638	1218.579	-380.849	-31.920	30.028	-3.580	-6.862	46.016	-0.508	24.092	-0.124
Y	619.395	11829.694	145.940	104.503	-295.638	176.497	579.096	2.352	-3.263	-2.804	0.570	-0.587	-3.255	0.986	2.338	-0.074
Zr	299.634	152124.844	768.564	-230.969	1218.579	579.096	4686.360	-257.465	-40.487	-1.998	-2.295	-9.567	10.934	3.492	33.026	-1.241
Nb	881.421	-14631.285	-21.608	307.073	-380.849	2.352	-257.465	136.276	6.723	-2.601	0.527	2.212	-4.006	-0.226	-3.590	0.523
Rb/Zr	22.169	-1648.760	-5.878	22.007	-31.920	-3.263	-40.487	6.723	0.673	-0.166	0.042	0.164	-0.308	-0.036	-0.388	0.014
Sr/Zr	-14.617	339.964	-1.727	-14.314	30.028	-2.804	-1.998	-2.601	-0.166	0.249	-0.020	-0.036	0.307	-0.008	0.083	0.001
Y/Zr	6.688	-116.022	-0.027	0.995	-3.580	0.570	-2.295	0.527	0.042	-0.020	0.010	0.014	-0.036	0.003	-0.025	0.002
Nb/Zr	13.092	-388.340	-1.283	4.767	-6.862	-0.587	-9.567	2.212	0.164	-0.036	0.014	0.048	-0.066	-0.007	-0.088	0.008
Sr/Rb	-20.491	966.944	-1.042	-20.170	46.016	-3.255	10.934	-4.006	-0.308	0.307	-0.036	-0.066	0.474	-0.006	0.242	-0.002
Y/Rb	3.441	96.772	0.818	-0.748	-0.508	0.986	3.492	-0.226	-0.036	-0.008	0.003	-0.007	-0.006	0.007	0.024	0.000
Zr/Rb	-4.349	1340.130	4.278	-12.009	24.092	2.338	33.026	-3.590	-0.388	0.083	-0.025	-0.088	0.242	0.024	0.324	-0.009
Nb/Rb	4.565	-46.679	-0.152	0.084	-0.124	-0.074	-1.241	0.523	0.014	0.001	0.002	0.008	-0.002	0.000	-0.009	0.003

Within-class covariance matrix for class Aghvorik:

	Mn	Fe	Zn	Rb	Sr	Y	Zr	Nb	Rb/Zr	Sr/Zr	Y/Zr	Nb/Zr	Sr/Rb	Y/Rb	Zr/Rb	Nb/Rb
Mn	230.764	1368.717	-8.003	8.240	15.928	-0.620	13.859	-11.749	0.010	0.020	-0.006	-0.051	0.002	-0.020	-0.058	-0.127
Fe	1368.717	21825.037	-87.466	68.741	174.719	78.828	275.344	-88.110	-0.187	-0.186	0.251	-0.428	0.403	0.669	1.076	-0.967
Zn	-8.003	-87.466	4.443	-0.824	2.307	2.280	1.718	1.329	-0.006	0.004	0.009	0.005	0.039	0.024	0.037	0.014
Rb	8.240	68.741	-0.824	2.104	-0.209	0.091	2.609	-1.145	0.004	-0.009	0.000	-0.005	-0.042	-0.002	-0.024	-0.014
Sr	15.928	174.719	2.307	-0.209	4.760	2.461	3.443	-0.636	-0.007	0.008	0.009	-0.003	0.051	0.025	0.039	-0.006
Y	-0.620	78.828	2.280	0.091	2.461	2.283	3.057	0.171	-0.005	0.000	0.009	0.000	0.023	0.023	0.028	0.002
Zr	13.859	275.344	1.718	2.609	3.443	3.057	8.267	-1.028	-0.003	-0.013	0.010	-0.006	-0.015	0.026	0.020	-0.014
Nb	-11.749	-88.110	1.329	-1.145	-0.636	0.171	-1.028	1.492	-0.003	0.001	0.001	0.006	0.015	0.003	0.017	0.016
Rb/Zr	0.010	-0.187	-0.006	0.004	-0.007	-0.005	-0.003	-0.003	0.000	0.000	0.000	0.000	0.000	0.000	0.000	0.000
Sr/Zr	0.020	-0.186	0.004	-0.009	0.008	0.000	-0.013	0.001	0.000	0.000	0.000	0.000	0.000	0.000	0.000	0.000
Y/Zr	-0.006	0.251	0.009	0.000	0.009	0.009	0.010	0.001	0.000	0.000	0.000	0.000	0.000	0.000	0.000	0.000
Nb/Zr	-0.051	-0.428	0.005	-0.005	-0.003	0.000	-0.006	0.006	0.000	0.000	0.000	0.000	0.000	0.000	0.000	0.000
Sr/Rb	0.002	0.403	0.039	-0.042	0.051	0.023	-0.015	0.015	0.000	0.000	0.000	0.000	0.001	0.000	0.001	0.000
Y/Rb	-0.020	0.669	0.024	-0.002	0.025	0.023	0.026	0.003	0.000	0.000	0.000	0.000	0.000	0.000	0.000	0.000
Zr/Rb	-0.058	1.076	0.037	-0.024	0.039	0.028	0.020	0.017	0.000	0.000	0.000	0.000	0.001	0.000	0.001	0.000
Nb/Rb	-0.127	-0.967	0.014	-0.014	-0.006	0.002	-0.014	0.016	0.000	0.000	0.000	0.000	0.000	0.000	0.000	0.000

Within-class covariance matrix for class Arteni:

	Mn	Fe	Zn	Rb	Sr	Y	Zr	Nb	Rb/Zr	Sr/Zr	Y/Zr	Nb/Zr	Sr/Rb	Y/Rb	Zr/Rb	Nb/Rb
Mn	6755.990	-26358.620	296.203	891.682	-630.292	359.603	-637.285	487.537	27.874	-5.945	8.194	10.574	-5.632	1.232	-8.133	1.900
Fe	-26358.620	113889.161	-1190.629	-3734.750	2793.293	-1576.427	2501.507	-1990.933	-110.922	27.107	-33.939	-42.082	25.005	-5.709	33.224	-7.621
Zn	296.203	-1190.629	17.006	38.089	-29.160	15.896	-28.822	22.012	1.228	-0.279	0.366	0.477	-0.257	0.056	-0.359	0.090
Rb	891.682	-3734.750	38.089	128.865	-90.289	53.349	-85.157	67.076	3.810	-0.862	1.153	1.424	-0.814	0.189	-1.133	0.252
Sr	-630.292	2793.293	-29.160	-90.289	71.715	-38.356	58.743	-47.380	-2.626	0.709	-0.810	-0.993	0.641	-0.141	0.797	-0.180
Y	359.603	-1576.427	15.896	53.349	-38.356	24.590	-35.027	28.001	1.563	-0.372	0.508	0.589	-0.343	0.096	-0.467	0.105
Zr	-637.285	2501.507	-28.822	-85.157	58.743	-35.027	69.517	-47.161	-2.884	0.524	-0.838	-1.076	0.524	-0.123	0.835	-0.184
Nb	487.537	-1990.933	22.012	67.076	-47.380	28.001	-47.161	40.658	2.067	-0.451	0.623	0.843	-0.422	0.099	-0.607	0.171
Rb/Zr	27.874	-110.922	1.228	3.810	-2.626	1.563	-2.884	2.067	0.123	-0.024	0.036	0.046	-0.024	0.005	-0.036	0.008
Sr/Zr	-5.945	27.107	-0.279	-0.862	0.709	-0.372	0.524	-0.451	-0.024	0.007	-0.008	-0.009	0.006	-0.001	0.007	-0.002
Y/Zr	8.194	-33.939	0.366	1.153	-0.810	0.508	-0.838	0.623	0.036	-0.008	0.011	0.014	-0.007	0.002	-0.011	0.002
Nb/Zr	10.574	-42.082	0.477	1.424	-0.993	0.589	-1.076	0.843	0.046	-0.009	0.014	0.018	-0.009	0.002	-0.013	0.003
Sr/Rb	-5.632	25.005	-0.257	-0.814	0.641	-0.343	0.524	-0.422	-0.024	0.006	-0.007	-0.009	0.006	-0.001	0.007	-0.002
Y/Rb	1.232	-5.709	0.056	0.189	-0.141	0.096	-0.123	0.099	0.005	-0.001	0.002	0.002	-0.001	0.000	-0.002	0.000
Zr/Rb	-8.133	33.224	-0.359	-1.133	0.797	-0.467	0.835	-0.607	-0.036	0.007	-0.011	-0.013	0.007	-0.002	0.011	-0.002
Nb/Rb	1.900	-7.621	0.090	0.252	-0.180	0.105	-0.184	0.171	0.008	-0.002	0.002	0.003	-0.002	0.000	-0.002	0.001

Within-class covariance matrix for class Chikiani:

	Mn	Fe	Zn	Rb	Sr	Y	Zr	Nb	Rb/Zr	Sr/Zr	Y/Zr	Nb/Zr	Sr/Rb	Y/Rb	Zr/Rb	Nb/Rb
Mn	609.247	-4194.656	-7.254	72.267	-110.664	2.127	-144.528	17.023	3.013	0.225	0.292	0.455	-1.237	-0.051	-1.541	0.064
Fe	-4194.656	83150.611	-32.292	-827.146	2036.814	-87.442	2635.733	-298.266	-49.329	-3.766	-5.677	-8.332	20.058	0.083	25.161	-1.524
Zn	-7.254	-32.292	5.252	-0.340	-0.210	0.247	-0.362	0.472	0.007	0.001	0.004	0.005	0.002	0.002	0.001	0.004
Rb	72.267	-827.146	-0.340	14.850	-20.597	0.346	-28.250	2.506	0.582	0.053	0.054	0.080	-0.239	-0.011	-0.307	0.005
Sr	-110.664	2036.814	-0.210	-20.597	52.059	-1.452	66.228	-8.382	-1.237	-0.086	-0.134	-0.219	0.510	0.008	0.632	-0.045
Y	2.127	-87.442	0.247	0.346	-1.452	0.890	-1.538	-0.225	0.029	-0.001	0.013	0.001	-0.013	0.007	-0.013	-0.002
Zr	-144.528	2635.733	-0.362	-28.250	66.228	-1.538	90.684	-9.347	-1.689	-0.169	-0.178	-0.276	0.659	0.014	0.865	-0.046
Nb	17.023	-298.266	0.472	2.506	-8.382	-0.225	-9.347	3.456	0.168	0.001	0.014	0.055	-0.078	-0.004	-0.088	0.024
Rb/Zr	3.013	-49.329	0.007	0.582	-1.237	0.029	-1.689	0.168	0.032	0.003	0.003	0.005	-0.013	0.000	-0.016	0.001
Sr/Zr	0.225	-3.766	0.001	0.053	-0.086	-0.001	-0.169	0.001	0.003	0.001	0.000	0.000	-0.001	0.000	-0.002	0.000
Y/Zr	0.292	-5.677	0.004	0.054	-0.134	0.013	-0.178	0.014	0.003	0.000	0.000	0.000	-0.001	0.000	-0.002	0.000
Nb/Zr	0.455	-8.332	0.005	0.080	-0.219	0.001	-0.276	0.055	0.005	0.000	0.000	0.001	-0.002	0.000	-0.003	0.000
Sr/Rb	-1.237	20.058	0.002	-0.239	0.510	-0.013	0.659	-0.078	-0.013	-0.001	-0.001	-0.002	0.005	0.000	0.006	0.000
Y/Rb	-0.051	0.083	0.002	-0.011	0.008	0.007	0.014	-0.004	0.000	0.000	0.000	0.000	0.000	0.000	0.000	0.000
Zr/Rb	-1.541	25.161	0.001	-0.307	0.632	-0.013	0.865	-0.088	-0.016	-0.002	-0.002	-0.003	0.006	0.000	0.008	0.000
Nb/Rb	0.064	-1.524	0.004	0.005	-0.045	-0.002	-0.046	0.024	0.001	0.000	0.000	0.000	0.000	0.000	0.000	0.000

Within-class covariance matrix for class Gegham 1:

	Mn	Fe	Zn	Rb	Sr	Y	Zr	Nb	Rb/Zr	Sr/Zr	Y/Zr	Nb/Zr	Sr/Rb	Y/Rb	Zr/Rb	Nb/Rb
Mn	5683.738	-3516.965	127.679	-892.772	-36.398	-66.313	-542.170	-20.879	13.107	1.097	2.242	7.265	0.077	0.209	-1.367	1.147
Fe	-3516.965	6076.971	-94.368	258.133	54.012	12.346	413.594	-34.377	-15.978	-0.382	-2.224	-6.031	0.190	-0.097	1.668	-0.552
Zn	127.679	-94.368	6.145	-17.217	0.026	-0.931	-11.554	-0.588	0.314	0.036	0.056	0.153	0.005	0.006	-0.032	0.021
Rb	-892.772	258.133	-17.217	193.321	1.484	15.708	79.736	10.186	-1.077	-0.224	-0.252	-1.006	-0.046	-0.038	0.110	-0.216
Sr	-36.398	54.012	0.026	1.484	1.647	0.454	5.144	-0.573	-0.219	0.011	-0.022	-0.075	0.008	0.001	0.023	-0.005
Y	-66.313	12.346	-0.931	15.708	0.454	2.434	6.354	0.826	-0.088	-0.013	-0.001	-0.083	-0.002	0.003	0.009	-0.018
Zr	-542.170	413.594	-11.554	79.736	5.144	6.354	58.173	0.625	-1.662	-0.098	-0.252	-0.803	0.002	-0.016	0.171	-0.109
Nb	-20.879	-34.377	-0.588	10.186	-0.573	0.826	0.625	2.761	0.111	-0.011	0.007	0.030	-0.005	-0.002	-0.011	0.001
Rb/Zr	13.107	-15.978	0.314	-1.077	-0.219	-0.088	-1.662	0.111	0.065	0.002	0.008	0.024	-0.001	0.000	-0.007	0.002
Sr/Zr	1.097	-0.382	0.036	-0.224	0.011	-0.013	-0.098	-0.011	0.002	0.000	0.000	0.001	0.000	0.000	0.000	0.000
Y/Zr	2.242	-2.224	0.056	-0.252	-0.022	-0.001	-0.252	0.007	0.008	0.000	0.002	0.004	0.000	0.000	-0.001	0.000
Nb/Zr	7.265	-6.031	0.153	-1.006	-0.075	-0.083	-0.803	0.030	0.024	0.001	0.004	0.012	0.000	0.000	-0.003	0.002
Sr/Rb	0.077	0.190	0.005	-0.046	0.008	-0.002	0.002	-0.005	-0.001	0.000	0.000	0.000	0.000	0.000	0.000	0.000
Y/Rb	0.209	-0.097	0.006	-0.038	0.001	0.003	-0.016	-0.002	0.000	0.000	0.000	0.000	0.000	0.000	0.000	0.000
Zr/Rb	-1.367	1.668	-0.032	0.110	0.023	0.009	0.171	-0.011	-0.007	0.000	-0.001	-0.003	0.000	0.000	0.001	0.000
Nb/Rb	1.147	-0.552	0.021	-0.216	-0.005	-0.018	-0.109	0.001	0.002	0.000	0.000	0.002	0.000	0.000	0.000	0.000

Within-class covariance matrix for class Gegham 2:

	Mn	Fe	Zn	Rb	Sr	Y	Zr	Nb	Rb/Zr	Sr/Zr	Y/Zr	Nb/Zr	Sr/Rb	Y/Rb	Zr/Rb	Nb/Rb
Mn	687.457	-933.802	-7.181	-13.511	6.089	3.721	4.083	20.586	-0.154	0.038	0.024	0.170	0.112	0.061	0.159	0.212
Fe	-933.802	2192.682	29.444	46.898	7.993	-13.434	-9.129	-34.553	0.490	0.106	-0.099	-0.281	-0.130	-0.217	-0.508	-0.413
Zn	-7.181	29.444	2.863	-0.209	-0.378	-0.152	-0.814	0.295	0.005	0.000	0.000	0.004	-0.003	-0.001	-0.005	0.003
Rb	-13.511	46.898	-0.209	3.131	0.915	0.081	1.915	-1.487	0.011	0.000	-0.003	-0.017	-0.005	-0.006	-0.011	-0.020
Sr	6.089	7.993	-0.378	0.915	1.297	-0.166	0.389	-0.081	0.005	0.010	-0.002	-0.001	0.008	-0.003	-0.005	-0.003
Y	3.721	-13.434	-0.152	0.081	-0.166	0.234	0.549	0.000	-0.004	-0.004	0.001	-0.001	-0.002	0.002	0.004	0.000
Zr	4.083	-9.129	-0.814	1.915	0.389	0.549	2.336	-0.762	-0.003	-0.006	0.000	-0.012	-0.005	0.001	0.003	-0.011
Nb	20.586	-34.553	0.295	-1.487	-0.081	0.000	-0.762	1.105	-0.006	0.002	0.002	0.011	0.006	0.003	0.007	0.013
Rb/Zr	-0.154	0.490	0.005	0.011	0.005	-0.004	-0.003	-0.006	0.000	0.000	0.000	0.000	0.000	0.000	0.000	0.000
Sr/Zr	0.038	0.106	0.000	0.000	0.010	-0.004	-0.006	0.002	0.000	0.000	0.000	0.000	0.000	0.000	0.000	0.000
Y/Zr	0.024	-0.099	0.000	-0.003	-0.002	0.001	0.000	0.002	0.000	0.000	0.000	0.000	0.000	0.000	0.000	0.000
Nb/Zr	0.170	-0.281	0.004	-0.017	-0.001	-0.001	-0.012	0.011	0.000	0.000	0.000	0.000	0.000	0.000	0.000	0.000
Sr/Rb	0.112	-0.130	-0.003	-0.005	0.008	-0.002	-0.005	0.006	0.000	0.000	0.000	0.000	0.000	0.000	0.000	0.000
Y/Rb	0.061	-0.217	-0.001	-0.006	-0.003	0.002	0.001	0.003	0.000	0.000	0.000	0.000	0.000	0.000	0.000	0.000
Zr/Rb	0.159	-0.508	-0.005	-0.011	-0.005	0.004	0.003	0.007	0.000	0.000	0.000	0.000	0.000	0.000	0.000	0.000
Nb/Rb	0.212	-0.413	0.003	-0.020	-0.003	0.000	-0.011	0.013	0.000	0.000	0.000	0.000	0.000	0.000	0.000	0.000

Within-class covariance matrix for class Gutansar:

	Mn	Fe	Zn	Rb	Sr	Y	Zr	Nb	Rb/Zr	Sr/Zr	Y/Zr	Nb/Zr	Sr/Rb	Y/Rb	Zr/Rb	Nb/Rb
Mn	285.102	504.741	-9.111	1.573	6.987	1.137	13.132	-9.075	-0.054	-0.016	-0.005	-0.069	0.037	0.006	0.078	-0.066

Fe	504.741	42143.603	200.787	-5.648	326.217	64.916	455.843	18.647	-2.257	-0.047	0.015	-0.468	2.303	0.461	3.257	0.144
Zn	-9.111	200.787	9.990	0.428	0.641	1.242	1.775	3.744	-0.006	-0.004	0.006	0.020	0.002	0.008	0.009	0.026
Rb	1.573	-5.648	0.428	7.023	2.569	-0.470	4.156	1.162	0.021	-0.003	-0.006	0.002	-0.026	-0.011	-0.031	-0.005
Sr	6.987	326.217	0.641	2.569	15.031	-0.222	12.206	0.520	-0.044	0.036	-0.011	-0.012	0.090	-0.005	0.064	-0.001
Y	1.137	64.916	1.242	-0.470	-0.222	1.379	0.470	0.379	-0.005	-0.003	0.008	0.002	0.001	0.010	0.007	0.004
Zr	13.132	455.843	1.775	4.156	12.206	0.470	14.114	1.185	-0.044	0.011	-0.009	-0.011	0.060	-0.002	0.064	0.001
Nb	-9.075	18.647	3.744	1.162	0.520	0.379	1.185	3.975	0.001	-0.002	0.001	0.022	-0.004	0.001	-0.002	0.026
Rb/Zr	-0.054	-2.257	-0.006	0.021	-0.044	-0.005	-0.044	0.001	0.000	0.000	0.000	0.000	0.000	0.000	0.000	0.000
Sr/Zr	-0.016	-0.047	-0.004	-0.003	0.036	-0.003	0.011	-0.002	0.000	0.000	0.000	0.000	0.000	0.000	0.000	0.000
Y/Zr	-0.005	0.015	0.006	-0.006	-0.011	0.008	-0.009	0.001	0.000	0.000	0.000	0.000	0.000	0.000	0.000	0.000
Nb/Zr	-0.069	-0.468	0.020	0.002	-0.012	0.002	-0.011	0.022	0.000	0.000	0.000	0.000	0.000	0.000	0.000	0.000
Sr/Rb	0.037	2.303	0.002	-0.026	0.090	0.001	0.060	-0.004	0.000	0.000	0.000	0.000	0.001	0.000	0.001	0.000
Y/Rb	0.006	0.461	0.008	-0.011	-0.005	0.010	-0.002	0.001	0.000	0.000	0.000	0.000	0.000	0.000	0.000	0.000
Zr/Rb	0.078	3.257	0.009	-0.031	0.064	0.007	0.064	-0.002	0.000	0.000	0.000	0.000	0.001	0.000	0.001	0.000
Nb/Rb	-0.066	0.144	0.026	-0.005	-0.001	0.004	0.001	0.026	0.000	0.000	0.000	0.000	0.000	0.000	0.000	0.000

Within-class covariance matrix for class Hatis:

	Mn	Fe	Zn	Rb	Sr	Y	Zr	Nb	Rb/Zr	Sr/Zr	Y/Zr	Nb/Zr	Sr/Rb	Y/Rb	Zr/Rb	Nb/Rb
Mn	485.535	1540.988	3.969	-13.662	40.864	-1.129	9.081	-8.971	-0.180	0.267	-0.019	-0.090	0.591	0.010	0.237	-0.063
Fe	1540.988	2153801.669	2051.878	-8309.090	45504.434	-665.475	18378.449	-1308.109	-243.417	188.992	-31.173	-43.996	549.508	5.691	258.778	2.698
Zn	3.969	2051.878	6.591	-8.258	42.855	-0.396	18.153	-1.884	-0.247	0.172	-0.029	-0.051	0.518	0.008	0.254	-0.003
Rb	-13.662	-8309.090	-8.258	36.269	-173.825	2.619	-70.819	5.502	0.986	-0.719	0.122	0.175	-2.147	-0.027	-1.032	-0.013
Sr	40.864	45504.434	42.855	-173.825	965.536	-13.717	389.926	-27.437	-5.125	3.999	-0.655	-0.928	11.643	0.121	5.474	0.056
Y	-1.129	-665.475	-0.396	2.619	-13.717	1.677	-4.798	0.420	0.076	-0.072	0.024	0.014	-0.157	0.012	-0.067	-0.001
Zr	9.081	18378.449	18.153	-70.819	389.926	-4.798	161.701	-10.334	-2.112	1.556	-0.264	-0.373	4.720	0.057	2.261	0.030
Nb	-8.971	-1308.109	-1.884	5.502	-27.437	0.420	-10.334	4.930	0.149	-0.130	0.019	0.067	-0.332	-0.004	-0.152	0.036
Rb/Zr	-0.180	-243.417	-0.247	0.986	-5.125	0.076	-2.112	0.149	0.029	-0.021	0.004	0.005	-0.062	-0.001	-0.030	0.000
Sr/Zr	0.267	188.992	0.172	-0.719	3.999	-0.072	1.556	-0.130	-0.021	0.018	-0.003	-0.004	0.048	0.000	0.022	0.000
Y/Zr	-0.019	-31.173	-0.029	0.122	-0.655	0.024	-0.264	0.019	0.004	-0.003	0.001	0.001	-0.008	0.000	-0.004	0.000
Nb/Zr	-0.090	-43.996	-0.051	0.175	-0.928	0.014	-0.373	0.067	0.005	-0.004	0.001	0.001	-0.011	0.000	-0.005	0.000
Sr/Rb	0.591	549.508	0.518	-2.147	11.643	-0.157	4.720	-0.332	-0.062	0.048	-0.008	-0.011	0.141	0.002	0.067	0.001
Y/Rb	0.010	5.691	0.008	-0.027	0.121	0.012	0.057	-0.004	-0.001	0.000	0.000	0.000	0.002	0.000	0.001	0.000
Zr/Rb	0.237	258.778	0.254	-1.032	5.474	-0.067	2.261	-0.152	-0.030	0.022	-0.004	-0.005	0.067	0.001	0.032	0.000
Nb/Rb	-0.063	2.698	-0.003	-0.013	0.056	-0.001	0.030	0.036	0.000	0.000	0.000	0.000	0.001	0.000	0.000	0.000

Within-class covariance matrix for class Kars-Arpaçay:

	Mn	Fe	Zn	Rb	Sr	Y	Zr	Nb	Rb/Zr	Sr/Zr	Y/Zr	Nb/Zr	Sr/Rb	Y/Rb	Zr/Rb	Nb/Rb
Mn	2849.511	47267.351	188.248	155.590	434.312	15.725	576.085	-48.871	-0.715	1.662	-0.432	-0.490	2.967	-0.288	2.348	-0.553
Fe	47267.351	1835204.182	2114.939	-4779.761	19581.505	-2483.489	5780.206	-4487.208	-35.497	87.335	-16.371	-23.525	156.266	-6.327	105.319	-27.730
Zn	188.248	2114.939	21.018	23.118	15.372	4.077	34.004	1.526	0.022	0.049	-0.011	-0.008	0.068	-0.030	-0.043	-0.016
Rb	155.590	-4779.761	23.118	70.713	-75.692	20.404	26.212	25.043	0.246	-0.362	0.068	0.103	-0.707	-0.031	-0.720	0.101
Sr	434.312	19581.505	15.372	-75.692	285.705	-20.150	191.733	-50.107	-0.792	1.165	-0.254	-0.312	2.303	0.048	2.458	-0.279
Y	15.725	-2483.489	4.077	20.404	-20.150	9.735	36.999	10.818	0.001	-0.122	0.012	0.035	-0.187	0.021	0.023	0.056
Zr	576.085	5780.206	34.004	26.212	191.733	36.999	499.089	13.710	-1.064	0.496	-0.251	-0.144	1.415	0.217	3.505	0.079
Nb	-48.871	-4487.208	1.526	25.043	-50.107	10.818	13.710	15.914	0.079	-0.242	0.038	0.068	-0.425	0.017	-0.218	0.089
Rb/Zr	-0.715	-35.497	0.022	0.246	-0.792	0.001	-1.064	0.079	0.004	-0.003	0.001	0.001	-0.007	-0.001	-0.011	0.000
Sr/Zr	1.662	87.335	0.049	-0.362	1.165	-0.122	0.496	-0.242	-0.003	0.005	-0.001	-0.001	0.009	0.000	0.009	-0.001
Y/Zr	-0.432	-16.371	-0.011	0.068	-0.254	0.012	-0.251	0.038	0.001	-0.001	0.000	0.000	-0.002	0.000	-0.003	0.000
Nb/Zr	-0.490	-23.525	-0.008	0.103	-0.312	0.035	-0.144	0.068	0.001	-0.001	0.000	0.000	-0.003	0.000	-0.002	0.000
Sr/Rb	2.967	156.266	0.068	-0.707	2.303	-0.187	1.415	-0.425	-0.007	0.009	-0.002	-0.003	0.019	0.000	0.020	-0.002
Y/Rb	-0.288	-6.327	-0.030	-0.031	0.048	0.021	0.217	0.017	-0.001	0.000	0.000	0.000	0.000	0.000	0.002	0.000
Zr/Rb	2.348	105.319	-0.043	-0.720	2.458	0.023	3.505	-0.218	-0.011	0.009	-0.003	-0.002	0.020	0.002	0.036	-0.001
Nb/Rb	-0.553	-27.730	-0.016	0.101	-0.279	0.056	0.079	0.089	0.000	-0.001	0.000	0.000	-0.002	0.000	-0.001	0.001

Within-class covariance matrix for class Kelbadjar:

	Mn	Fe	Zn	Rb	Sr	Y	Zr	Nb	Rb/Zr	Sr/Zr	Y/Zr	Nb/Zr	Sr/Rb	Y/Rb	Zr/Rb	Nb/Rb
Mn	1095.332	2054.940	95.705	50.631	0.000	0.000	57.442	36.753	-0.299	-0.077	-0.053	0.169	-0.035	-0.024	0.131	0.169
Fe	2054.940	7805.427	157.034	228.779	0.000	0.000	-101.208	88.110	3.565	0.146	0.099	1.122	-0.156	-0.106	-1.750	0.253
Zn	95.705	157.034	8.491	3.661	0.000	0.000	6.210	3.102	-0.050	-0.008	-0.006	0.010	-0.003	-0.002	0.023	0.015
Rb	50.631	228.779	3.661	6.872	0.000	0.000	-4.423	2.348	0.126	0.006	0.004	0.035	-0.005	-0.003	-0.062	0.006

Sr	0.000	0.000	0.000	0.000	0.000	0.000	0.000	0.000	0.000	0.000	0.000	0.000	0.000	0.000	0.000	0.000
Y	0.000	0.000	0.000	0.000	0.000	0.000	0.000	0.000	0.000	0.000	0.000	0.000	0.000	0.000	0.000	0.000
Zr	57.442	-101.208	6.210	-4.423	0.000	0.000	14.068	0.914	-0.234	-0.019	-0.013	-0.034	0.003	0.002	0.112	0.012
Nb	36.753	88.110	3.102	2.348	0.000	0.000	0.914	1.326	0.010	-0.001	-0.001	0.010	-0.002	-0.001	-0.005	0.005
Rb/Zr	-0.299	3.565	-0.050	0.126	0.000	0.000	-0.234	0.010	0.004	0.000	0.000	0.001	0.000	0.000	-0.002	0.000
Sr/Zr	-0.077	0.146	-0.008	0.006	0.000	0.000	-0.019	-0.001	0.000	0.000	0.000	0.000	0.000	0.000	0.000	0.000
Y/Zr	-0.053	0.099	-0.006	0.004	0.000	0.000	-0.013	-0.001	0.000	0.000	0.000	0.000	0.000	0.000	0.000	0.000
Nb/Zr	0.169	1.122	0.010	0.035	0.000	0.000	-0.034	0.010	0.001	0.000	0.000	0.000	0.000	0.000	0.000	0.000
Sr/Rb	-0.035	-0.156	-0.003	-0.005	0.000	0.000	0.003	-0.002	0.000	0.000	0.000	0.000	0.000	0.000	0.000	0.000
Y/Rb	-0.024	-0.106	-0.002	-0.003	0.000	0.000	0.002	-0.001	0.000	0.000	0.000	0.000	0.000	0.000	0.000	0.000
Zr/Rb	0.131	-1.750	0.023	-0.062	0.000	0.000	0.112	-0.005	-0.002	0.000	0.000	0.000	0.000	0.000	0.001	0.000
Nb/Rb	0.169	0.253	0.015	0.006	0.000	0.000	0.012	0.005	0.000	0.000	0.000	0.000	0.000	0.000	0.000	0.000

Within-class covariance matrix for class Khorapor:

	Mn	Fe	Zn	Rb	Sr	Y	Zr	Nb	Rb/Zr	Sr/Zr	Y/Zr	Nb/Zr	Sr/Rb	Y/Rb	Zr/Rb	Nb/Rb
Mn	539.636	969.647	0.805	58.026	-8.583	7.441	-5.883	-10.544	0.933	-0.102	0.107	-0.097	-0.048	0.018	-0.125	-0.091
Fe	969.647	2540.665	2.117	167.710	-17.669	40.204	-3.374	25.483	2.244	-0.218	0.519	0.363	-0.108	0.141	-0.301	0.001
Zn	0.805	2.117	2.073	5.178	-1.619	1.672	1.833	2.437	0.002	-0.023	0.018	0.021	-0.008	0.006	0.000	0.008
Rb	58.026	167.710	5.178	27.183	-6.174	7.490	3.398	10.682	0.228	-0.084	0.089	0.118	-0.033	0.027	-0.030	0.030
Sr	-8.583	-17.669	-1.619	-6.174	1.769	-1.662	-1.002	-2.422	-0.044	0.024	-0.019	-0.025	0.009	-0.006	0.006	-0.007
Y	7.441	40.204	1.672	7.490	-1.662	2.419	1.411	3.298	0.047	-0.023	0.028	0.034	-0.009	0.009	-0.006	0.010
Zr	-5.883	-3.374	1.833	3.398	-1.002	1.411	2.100	2.513	-0.029	-0.016	0.014	0.020	-0.005	0.006	0.004	0.009
Nb	-10.544	25.483	2.437	10.682	-2.422	3.298	2.513	11.106	0.051	-0.034	0.037	0.128	-0.013	0.013	-0.007	0.044
Rb/Zr	0.933	2.244	0.002	0.228	-0.044	0.047	-0.029	0.051	0.004	-0.001	0.001	0.001	0.000	0.000	-0.001	0.000
Sr/Zr	-0.102	-0.218	-0.023	-0.084	0.024	-0.023	-0.016	-0.034	-0.001	0.000	0.000	0.000	0.000	0.000	0.000	0.000
Y/Zr	0.107	0.519	0.018	0.089	-0.019	0.028	0.014	0.037	0.001	0.000	0.000	0.000	0.000	0.000	0.000	0.000
Nb/Zr	-0.097	0.363	0.021	0.118	-0.025	0.034	0.020	0.128	0.001	0.000	0.000	0.002	0.000	0.000	0.000	0.001
Sr/Rb	-0.048	-0.108	-0.008	-0.033	0.009	-0.009	-0.005	-0.013	0.000	0.000	0.000	0.000	0.000	0.000	0.000	0.000
Y/Rb	0.018	0.141	0.006	0.027	-0.006	0.009	0.006	0.013	0.000	0.000	0.000	0.000	0.000	0.000	0.000	0.000
Zr/Rb	-0.125	-0.301	0.000	-0.030	0.006	-0.006	0.004	-0.007	-0.001	0.000	0.000	0.000	0.000	0.000	0.000	0.000
Nb/Rb	-0.091	0.001	0.008	0.030	-0.007	0.010	0.009	0.044	0.000	0.000	0.000	0.001	0.000	0.000	0.000	0.000

Within-class covariance matrix for class Meydan Dağ:

	Mn	Fe	Zn	Rb	Sr	Y	Zr	Nb	Rb/Zr	Sr/Zr	Y/Zr	Nb/Zr	Sr/Rb	Y/Rb	Zr/Rb	Nb/Rb
Mn	587.186	83.260	8.069	5.310	-36.108	12.479	-38.018	2.589	0.116	-0.116	0.070	0.022	-0.179	0.053	-0.222	0.008
Fe	83.260	1901.591	28.439	-42.684	13.088	29.810	21.697	35.193	-0.206	0.040	0.091	0.117	0.090	0.200	0.396	0.202
Zn	8.069	28.439	16.806	4.149	-0.348	3.964	2.511	0.410	0.008	-0.002	0.012	0.001	-0.004	0.014	-0.016	-0.001
Rb	5.310	-42.684	4.149	5.763	-0.076	0.573	2.050	-0.261	0.015	-0.001	0.001	-0.002	-0.004	-0.005	-0.029	-0.005
Sr	-36.108	13.088	-0.348	-0.076	3.093	-0.566	3.375	0.036	-0.009	0.010	-0.004	-0.001	0.015	-0.003	0.017	0.000
Y	12.479	29.810	3.964	0.573	-0.566	2.867	1.289	0.590	-0.001	-0.002	0.009	0.002	-0.003	0.013	0.002	0.002
Zr	-38.018	21.697	2.511	2.050	3.375	1.289	6.719	0.355	-0.010	0.010	0.000	-0.001	0.015	0.004	0.019	0.000
Nb	2.589	35.193	0.410	-0.261	0.036	0.590	0.355	0.933	-0.002	0.000	0.002	0.003	0.000	0.003	0.004	0.005
Rb/Zr	0.116	-0.206	0.008	0.015	-0.009	-0.001	-0.010	-0.002	0.000	0.000	0.000	0.000	0.000	0.000	0.000	0.000
Sr/Zr	-0.116	0.040	-0.002	-0.001	0.010	-0.002	0.010	0.000	0.000	0.000	0.000	0.000	0.000	0.000	0.000	0.000
Y/Zr	0.070	0.091	0.012	0.001	-0.004	0.009	0.000	0.002	0.000	0.000	0.000	0.000	0.000	0.000	0.000	0.000
Nb/Zr	0.022	0.117	0.001	-0.002	-0.001	0.002	-0.001	0.003	0.000	0.000	0.000	0.000	0.000	0.000	0.000	0.000
Sr/Rb	-0.179	0.090	-0.004	-0.004	0.015	-0.003	0.015	0.000	0.000	0.000	0.000	0.000	0.000	0.000	0.000	0.000
Y/Rb	0.053	0.200	0.014	-0.005	-0.003	0.013	0.004	0.003	0.000	0.000	0.000	0.000	0.000	0.000	0.000	0.000
Zr/Rb	-0.222	0.396	-0.016	-0.029	0.017	0.002	0.019	0.004	0.000	0.000	0.000	0.000	0.000	0.000	0.000	0.000
Nb/Rb	0.008	0.202	-0.001	-0.005	0.000	0.002	0.000	0.005	0.000	0.000	0.000	0.000	0.000	0.000	0.000	0.000

Within-class covariance matrix for class Sarıkamış:

	Mn	Fe	Zn	Rb	Sr	Y	Zr	Nb	Rb/Zr	Sr/Zr	Y/Zr	Nb/Zr	Sr/Rb	Y/Rb	Zr/Rb	Nb/Rb
Mn	1656.718	-22044.640	43.447	-90.160	-7.266	-77.436	-641.238	-4.214	5.201	0.993	0.501	0.417	0.062	-0.426	-3.969	0.024
Fe	-22044.640	1118006.866	443.041	3453.298	371.918	2262.778	24717.862	527.116	-186.118	-35.251	-22.291	-10.834	-1.848	11.495	154.141	2.057
Zn	43.447	443.041	5.279	1.510	0.730	-0.291	1.046	0.220	0.021	0.007	0.001	0.003	0.003	-0.004	0.000	0.001
Rb	-90.160	3453.298	1.510	30.752	0.883	10.391	76.604	5.443	-0.315	-0.098	-0.031	0.005	-0.036	0.032	0.347	0.025
Sr	-7.266	371.918	0.730	0.883	2.060	0.088	10.643	0.765	-0.113	-0.002	-0.022	-0.001	0.014	-0.001	0.071	0.005
Y	-77.436	2262.778	-0.291	10.391	0.088	6.683	53.456	1.284	-0.362	-0.081	-0.031	-0.023	-0.013	0.034	0.313	0.004
Zr	-641.238	24717.862	1.046	76.604	10.643	53.456	574.523	12.468	-4.456	-0.815	-0.531	-0.258	-0.022	0.277	3.603	0.050

Nb	-4.214	527.116	0.220	5.443	0.765	1.284	12.468	4.402	-0.044	-0.009	-0.009	0.035	-0.002	0.001	0.052	0.030
Rb/Zr	5.201	-186.118	0.021	-0.315	-0.113	-0.362	-4.456	-0.044	0.039	0.006	0.005	0.003	0.000	-0.002	-0.030	0.000
Sr/Zr	0.993	-35.251	0.007	-0.098	-0.002	-0.081	-0.815	-0.009	0.006	0.001	0.001	0.000	0.000	0.000	-0.005	0.000
Y/Zr	0.501	-22.291	0.001	-0.031	-0.022	-0.031	-0.531	-0.009	0.005	0.001	0.001	0.000	0.000	0.000	-0.004	0.000
Nb/Zr	0.417	-10.834	0.003	0.005	-0.001	-0.023	-0.258	0.035	0.003	0.000	0.000	0.001	0.000	0.000	-0.002	0.000
Sr/Rb	0.062	-1.848	0.003	-0.036	0.014	-0.013	-0.022	-0.002	0.000	0.000	0.000	0.000	0.000	0.000	0.000	0.000
Y/Rb	-0.426	11.495	-0.004	0.032	-0.001	0.034	0.277	0.001	-0.002	0.000	0.000	0.000	0.000	0.000	0.002	0.000
Zr/Rb	-3.969	154.141	0.000	0.347	0.071	0.313	3.603	0.052	-0.030	-0.005	-0.004	-0.002	0.000	0.002	0.023	0.000
Nb/Rb	0.024	2.057	0.001	0.025	0.005	0.004	0.050	0.030	0.000	0.000	0.000	0.000	0.000	0.000	0.000	0.000

Within-class covariance matrix for class Syunik:

	Mn	Fe	Zn	Rb	Sr	Y	Zr	Nb	Rb/Zr	Sr/Zr	Y/Zr	Nb/Zr	Sr/Rb	Y/Rb	Zr/Rb	Nb/Rb
Mn	2231.053	-7868.029	35.451	571.282	-165.253	-18.768	-122.296	130.727	8.603	-1.559	-0.081	1.874	-1.064	-0.239	-2.087	0.102
Fe	-7868.029	63128.816	-269.613	-2044.559	1160.713	173.256	885.334	-558.522	-39.882	10.972	1.001	-9.415	6.983	1.438	10.230	-0.869
Zn	35.451	-269.613	6.217	12.984	-4.666	-0.346	-4.714	2.422	0.234	-0.042	0.001	0.045	-0.030	-0.005	-0.059	-0.001
Rb	571.282	-2044.559	12.984	182.340	-43.907	-1.361	-34.090	38.487	2.632	-0.406	0.019	0.540	-0.293	-0.052	-0.639	0.016
Sr	-165.253	1160.713	-4.666	-43.907	23.230	3.114	17.952	-11.660	-0.834	0.219	0.016	-0.194	0.141	0.028	0.213	-0.017
Y	-18.768	173.256	-0.346	-1.361	3.114	2.288	3.238	-0.309	-0.086	0.029	0.022	-0.017	0.017	0.012	0.021	-0.001
Zr	-122.296	885.334	-4.714	-34.090	17.952	3.238	22.002	-7.237	-0.819	0.158	0.013	-0.164	0.110	0.026	0.210	-0.004
Nb	130.727	-558.522	2.422	38.487	-11.660	-0.309	-7.237	12.061	0.553	-0.111	0.004	0.156	-0.074	-0.011	-0.135	0.024
Rb/Zr	8.603	-39.882	0.234	2.632	-0.834	-0.086	-0.819	0.553	0.045	-0.008	0.000	0.009	-0.005	-0.001	-0.011	0.000
Sr/Zr	-1.559	10.972	-0.042	-0.406	0.219	0.029	0.158	-0.111	-0.008	0.002	0.000	-0.002	0.001	0.000	0.002	0.000
Y/Zr	-0.081	1.001	0.001	0.019	0.016	0.022	0.013	0.004	0.000	0.000	0.000	0.000	0.000	0.000	0.000	0.000
Nb/Zr	1.874	-9.415	0.045	0.540	-0.194	-0.017	-0.164	0.156	0.009	-0.002	0.000	0.002	-0.001	0.000	-0.002	0.000
Sr/Rb	-1.064	6.983	-0.030	-0.293	0.141	0.017	0.110	-0.074	-0.005	0.001	0.000	-0.001	0.001	0.000	0.001	0.000
Y/Rb	-0.239	1.438	-0.005	-0.052	0.028	0.012	0.026	-0.011	-0.001	0.000	0.000	0.000	0.000	0.000	0.000	0.000
Zr/Rb	-2.087	10.230	-0.059	-0.639	0.213	0.021	0.210	-0.135	-0.011	0.002	0.000	-0.002	0.001	0.000	0.003	0.000
Nb/Rb	0.102	-0.869	-0.001	0.016	-0.017	-0.001	-0.004	0.024	0.000	0.000	0.000	0.000	0.000	0.000	0.000	0.000

Within-class covariance matrix for class Tsaghkunyats:

	Mn	Fe	Zn	Rb	Sr	Y	Zr	Nb	Rb/Zr	Sr/Zr	Y/Zr	Nb/Zr	Sr/Rb	Y/Rb	Zr/Rb	Nb/Rb
Mn	420.508	-4038.609	-27.502	-12.953	-218.618	3.874	-189.983	38.070	0.841	0.806	0.115	0.535	-2.554	0.059	-2.166	0.424
Fe	-4038.609	359713.075	252.053	-3969.849	17720.881	-555.864	11913.876	-841.698	-110.857	-12.080	-11.850	-21.337	266.329	-1.970	175.545	-1.583
Zn	-27.502	252.053	9.961	6.098	14.492	0.462	16.651	-4.218	-0.014	-0.114	-0.001	-0.054	0.103	-0.002	0.144	-0.054
Rb	-12.953	-3969.849	6.098	72.137	-197.468	8.408	-114.262	3.380	1.462	-0.145	0.150	0.171	-3.344	0.017	-1.995	-0.096
Sr	-218.618	17720.881	14.492	-197.468	888.066	-29.325	596.350	-43.338	-5.533	-0.595	-0.607	-1.077	13.323	-0.115	8.776	-0.096
Y	3.874	-555.864	0.462	8.408	-29.325	3.353	-18.476	1.634	0.201	0.002	0.040	0.037	-0.464	0.027	-0.292	0.002
Zr	-189.983	11913.876	16.651	-114.262	596.350	-18.476	416.946	-35.475	-3.593	-0.652	-0.399	-0.796	8.716	-0.084	5.923	-0.164
Nb	38.070	-841.698	-4.218	3.380	-43.338	1.634	-35.475	6.680	0.229	0.127	0.032	0.099	-0.583	0.015	-0.453	0.064
Rb/Zr	0.841	-110.857	-0.014	1.462	-5.533	0.201	-3.593	0.229	0.037	0.002	0.004	0.006	-0.086	0.001	-0.055	0.000
Sr/Zr	0.806	-12.080	-0.114	-0.145	-0.595	0.002	-0.652	0.127	0.002	0.004	0.000	0.002	-0.005	0.000	-0.006	0.002
Y/Zr	0.115	-11.850	-0.001	0.150	-0.607	0.040	-0.399	0.032	0.004	0.000	0.001	0.001	-0.009	0.000	-0.006	0.000
Nb/Zr	0.535	-21.337	-0.054	0.171	-1.077	0.037	-0.796	0.099	0.006	0.002	0.001	0.002	-0.015	0.000	-0.011	0.001
Sr/Rb	-2.554	266.329	0.103	-3.344	13.323	-0.464	8.716	-0.583	-0.086	-0.005	-0.009	-0.015	0.205	-0.002	0.133	0.000
Y/Rb	0.059	-1.970	-0.002	0.017	-0.115	0.027	-0.084	0.015	0.001	0.000	0.000	0.000	-0.002	0.000	-0.001	0.000
Zr/Rb	-2.166	175.545	0.144	-1.995	8.776	-0.292	5.923	-0.453	-0.055	-0.006	-0.006	-0.011	0.133	-0.001	0.088	-0.001
Nb/Rb	0.424	-1.583	-0.054	-0.096	-0.096	0.002	-0.164	0.064	0.000	0.002	0.000	0.001	0.000	0.000	-0.001	0.001

Pooled within-class covariance matrix:

	Mn	Fe	Zn	Rb	Sr	Y	Zr	Nb	Rb/Zr	Sr/Zr	Y/Zr	Nb/Zr	Sr/Rb	Y/Rb	Zr/Rb	Nb/Rb
Mn	1722.699	-1083.263	52.895	56.539	-50.643	17.274	-117.694	44.213	4.164	-0.193	0.782	1.477	-0.493	0.043	-1.194	0.225
Fe	-1083.263	415098.597	257.526	-1392.138	6409.137	-194.285	4847.552	-639.088	-56.311	18.769	-8.689	-11.795	73.207	0.411	54.731	-2.565
Zn	52.895	257.526	8.724	4.883	2.860	1.980	2.688	2.234	0.108	-0.014	0.030	0.045	0.031	0.006	-0.003	0.008
Rb	56.539	-1392.138	4.883	55.809	-43.028	8.402	-10.023	12.066	0.624	-0.204	0.098	0.116	-0.553	0.006	-0.397	0.005
Sr	-50.643	6409.137	2.860	-43.028	165.426	-7.107	96.795	-13.621	-1.184	0.393	-0.180	-0.274	2.054	-0.003	1.326	-0.041
Y	17.274	-194.285	1.980	8.402	-7.107	4.345	3.356	3.349	0.098	-0.047	0.046	0.042	-0.082	0.019	-0.031	0.012
Zr	-117.694	4847.552	2.688	-10.023	96.795	3.356	138.588	-5.684	-1.329	0.069	-0.193	-0.281	1.155	0.029	1.257	-0.025
Nb	44.213	-639.088	2.234	12.066	-13.621	3.349	-5.684	7.914	0.240	-0.062	0.055	0.110	-0.137	0.010	-0.114	0.039
Rb/Zr	4.164	-56.311	0.108	0.624	-1.184	0.098	-1.329	0.240	0.027	-0.003	0.004	0.007	-0.014	0.000	-0.014	0.001
Sr/Zr	-0.193	18.769	-0.014	-0.204	0.393	-0.047	0.069	-0.062	-0.003	0.003	-0.001	-0.001	0.004	0.000	0.002	0.000

Y/Zr	0.782	-8.689	0.030	0.098	-0.180	0.046	-0.193	0.055	0.004	-0.001	0.001	0.001	-0.002	0.000	-0.002	0.000
Nb/Zr	1.477	-11.795	0.045	0.116	-0.274	0.042	-0.281	0.110	0.007	-0.001	0.001	0.003	-0.003	0.000	-0.003	0.001
Sr/Rb	-0.493	73.207	0.031	-0.553	2.054	-0.082	1.155	-0.137	-0.014	0.004	-0.002	-0.003	0.027	0.000	0.017	0.000
Y/Rb	0.043	0.411	0.006	0.006	-0.003	0.019	0.029	0.010	0.000	0.000	0.000	0.000	0.000	0.000	0.000	0.000
Zr/Rb	-1.194	54.731	-0.003	-0.397	1.326	-0.031	1.257	-0.114	-0.014	0.002	-0.002	-0.003	0.017	0.000	0.015	0.000
Nb/Rb	0.225	-2.565	0.008	0.005	-0.041	0.012	-0.025	0.039	0.001	0.000	0.000	0.001	0.000	0.000	0.000	0.000

Total covariance matrix:

	Mn	Fe	Zn	Rb	Sr	Y	Zr	Nb	Rb/Zr	Sr/Zr	Y/Zr	Nb/Zr	Sr/Rb	Y/Rb	Zr/Rb	Nb/Rb
Mn	12203.751	-24597.744	407.128	922.181	-1674.863	593.780	167.519	863.617	24.620	-13.809	6.977	13.607	-19.569	3.248	-5.188	4.469
Fe	-24597.74	6101323.00	19097.45	-37603.49	99868.73	10842.59	146408.42	-14245.659	-1590.387	334.727	-116.404	-373.197	970.876	90.597	1301.195	-45.948
Zn	407.128	19097.449	166.195	44.149	-67.416	137.920	718.975	-18.020	-5.376	-1.623	0.004	-1.153	-0.942	0.768	3.985	-0.134
Rb	922.181	-37603.491	44.149	1540.057	-1869.103	105.394	-224.820	297.700	21.107	-13.536	1.021	4.554	-19.326	-0.692	-11.571	0.083
Sr	-1674.863	99868.730	-67.416	-1869.103	4415.772	-282.331	1227.843	-367.949	-30.879	28.364	-3.508	-6.657	44.845	-0.476	23.717	-0.155
Y	593.780	10842.594	137.920	105.394	-282.331	168.650	542.996	5.374	-2.949	-2.658	0.575	-0.507	-3.112	0.937	2.149	-0.058
Zr	167.519	146408.424	718.975	-224.820	1227.843	542.996	4500.054	-245.397	-39.002	-1.797	-2.323	-9.185	11.289	3.283	31.979	-1.180
Nb	863.617	-14245.659	-18.020	297.700	-367.949	5.374	-245.397	134.548	6.496	-2.483	0.544	2.166	-3.864	-0.202	-3.455	0.524
Rb/Zr	24.620	-1590.387	-5.376	21.107	-30.879	-2.949	-39.002	6.496	0.653	-0.158	0.043	0.160	-0.301	-0.034	-0.376	0.014
Sr/Zr	-13.809	334.727	-1.623	-13.536	28.364	-2.658	-1.797	-2.483	-0.158	0.235	-0.019	-0.035	0.290	-0.008	0.079	0.000
Y/Zr	6.977	-116.404	0.004	1.021	-3.508	0.575	-2.323	0.544	0.043	-0.019	0.011	0.014	-0.035	0.003	-0.026	0.002
Nb/Zr	13.607	-373.197	-1.153	4.554	-6.657	-0.507	-9.185	2.166	0.160	-0.035	0.014	0.048	-0.065	-0.006	-0.085	0.008
Sr/Rb	-19.569	970.876	-0.942	-19.326	44.845	-3.112	11.289	-3.864	-0.301	0.290	-0.035	-0.065	0.468	-0.006	0.241	-0.002
Y/Rb	3.248	90.597	0.768	-0.692	-0.476	0.937	3.283	-0.202	-0.034	-0.008	0.003	-0.006	-0.006	0.007	0.023	0.000
Zr/Rb	-5.188	1301.195	3.985	-11.571	23.717	2.149	31.979	-3.455	-0.376	0.079	-0.026	-0.085	0.241	0.023	0.316	-0.008
Nb/Rb	4.469	-45.948	-0.134	0.083	-0.155	-0.058	-1.180	0.524	0.014	0.000	0.002	0.008	-0.002	0.000	-0.008	0.004

Eigenvalues:

	F1	F2	F3	F4	F5
Eigenvalue	206.688	109.352	67.121	30.749	28.744
Discriminatic	42.113	22.281	13.676	6.265	5.857
Cumulative 9	42.113	64.394	78.070	84.336	90.192

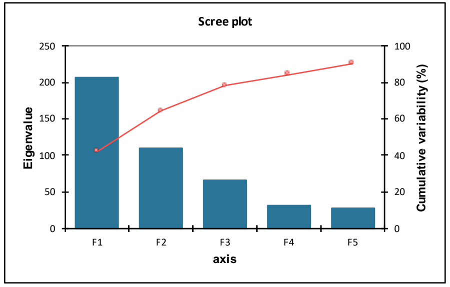

Bartlett's test for eigenvalue significancy:

	F1	F2	F3	F4	F5
Eigenvalue	206.688	109.352	67.121	30.749	28.744
Bartlett's sta	5151.202	3849.209	2701.512	1671.518	827.798
p-value	0.000	0.000	0.000	0.000	0.000

Variables/Factors correlations:

	F1	F2	F3	F4	F5
Mn	-0.207	0.106	0.341	-0.011	-0.183
Fe	0.062	0.686	0.088	0.466	0.446

Zn	-0.440	0.731	-0.214	0.293	0.126
Rb	-0.609	-0.531	-0.382	0.173	0.033
Sr	0.800	0.319	0.184	0.295	0.283
Y	-0.558	0.676	-0.202	0.031	-0.121
Zr	-0.265	0.713	-0.083	0.498	0.371
Nb	-0.274	-0.537	0.138	0.135	-0.208
Rb/Zr	-0.173	-0.814	-0.068	-0.322	-0.027
Sr/Zr	0.962	0.218	-0.023	0.066	0.016
Y/Zr	-0.276	-0.020	0.084	-0.477	-0.318
Nb/Zr	-0.099	-0.671	0.113	-0.254	-0.085
Sr/Rb	0.802	0.297	0.137	0.202	0.332
Y/Rb	-0.372	0.863	0.095	-0.142	-0.135
Zr/Rb	0.094	0.729	0.220	0.307	0.489
Nb/Rb	0.130	-0.223	0.438	0.097	-0.390

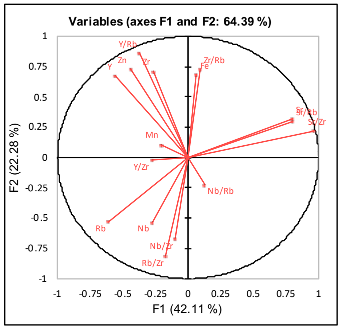

Classification functions:

	Aghvorik	Arteni	Chikiani	Gegham 1	Gegham 2	Gutansar	Hatis	Kars-Arpaçay	Kelbadjar	Khorapor	Meydan Dağ	Sarıkamış	Syunik	Tsaghkunyats
Intercept	-3924.287	-3101.275	-3418.011	-2978.498	-3358.878	-3415.839	-3591.499	-3513.215	-2967.024	-3630.469	-3522.386	-2880.761	-3279.315	-4363.361
Mn	0.270	0.346	0.210	0.218	0.426	0.480	0.282	0.603	0.105	-0.096	0.114	0.080	0.060	0.237
Fe	-0.085	-0.082	-0.124	-0.075	-0.082	-0.093	-0.130	-0.052	-0.059	-0.066	-0.056	-0.062	-0.069	-0.183
Zn	10.062	7.849	10.353	5.707	8.919	6.711	9.039	11.164	8.959	7.148	11.459	8.094	8.263	9.736
Rb	49.420	47.987	52.240	46.120	49.157	47.511	52.501	42.449	45.395	50.464	43.298	45.648	48.482	58.526
Sr	10.190	7.272	7.334	8.462	8.480	15.416	7.169	3.001	3.898	5.663	-2.211	2.813	3.613	7.550
Y	-11.083	-29.396	-19.950	-2.087	-24.407	-21.914	-18.392	-10.346	-49.190	-39.935	-36.814	-34.898	-57.503	-10.501
Zr	-22.069	-16.003	-18.461	-19.970	-18.676	-16.792	-18.882	-18.644	-10.960	-12.710	-5.010	-12.592	-9.701	-21.439
Nb	-143.905	-144.323	-153.082	-135.732	-147.282	-140.554	-150.520	-127.347	-133.531	-154.206	-139.979	-140.818	-140.685	-166.244
Rb/Zr	-847.783	-894.193	-1130.575	-749.307	-917.767	-865.884	-1204.242	-611.131	-744.700	-651.427	-639.573	-758.087	-778.400	-1522.967
Sr/Zr	1332.321	880.740	1550.766	937.181	919.475	840.742	1782.597	939.488	763.390	697.734	983.008	874.886	767.621	2317.068
Y/Zr	2886.354	2685.023	2592.771	1104.337	2328.377	2373.245	2595.142	1529.077	3113.424	2244.566	2630.987	2842.217	2922.071	2655.049
Nb/Zr	1735.078	1808.423	2786.138	2369.093	1796.947	1596.864	2939.502	1078.925	1392.129	1878.880	1472.948	1622.631	1767.977	4063.147
Sr/Rb	-1802.349	-1186.171	-1302.533	-1309.404	-1360.549	-1739.632	-1285.895	-1169.446	-928.201	-939.040	-402.880	-855.299	-794.239	-1346.259
Y/Rb	-2790.538	956.315	-845.829	-1627.527	889.112	-114.783	-770.348	624.180	1531.108	1534.117	2048.545	1377.159	2769.503	-2313.608
Zr/Rb	3874.837	2637.058	3086.705	3064.380	2948.023	2704.665	3113.719	3169.204	2209.240	2302.088	1605.549	2334.863	2003.002	3523.791
Nb/Rb	17326.749	17183.523	16582.616	15669.408	17754.022	17750.842	16240.736	15369.072	16568.235	18445.740	16085.822	16195.063	16898.360	16708.828

Prior and posterior classification, membership probabilities, scores and squared distances:

Observation	Prior	Posterior	Pr(Aghvorik)	Pr(Arteni)	Pr(Chikiani)	Pr(Gegham 1)	Pr(Gegham 2)	Pr(Gutansar)	Pr(Hatis)	Pr(Kars-Arpaçay)	Pr(Kelbadjar)	Pr(Khorapor)	Pr(Meydan Dağ)	Pr(Sarıkamış)	Pr(Syunik)	Pr(Tsaghkunyats)	F1	F2	F3	F4
Aghvorik AR6	Aghvorik	Aghvorik	1.000	0.000	0.000	0.000	0.000	0.000	0.000	0.000	0.000	0.000	0.000	0.000	0.000	0.000	6.404	5.385	11.614	5.244
Aghvorik AR6	Aghvorik	Aghvorik	1.000	0.000	0.000	0.000	0.000	0.000	0.000	0.000	0.000	0.000	0.000	0.000	0.000	0.000	5.647	6.187	10.436	5.624
Aghvorik AR6	Aghvorik	Aghvorik	1.000	0.000	0.000	0.000	0.000	0.000	0.000	0.000	0.000	0.000	0.000	0.000	0.000	0.000	5.947	5.297	10.780	4.757
Aghvorik AR6	Aghvorik	Aghvorik	1.000	0.000	0.000	0.000	0.000	0.000	0.000	0.000	0.000	0.000	0.000	0.000	0.000	0.000	5.948	6.563	11.920	4.041
Aghvorik AR6	Aghvorik	Aghvorik	1.000	0.000	0.000	0.000	0.000	0.000	0.000	0.000	0.000	0.000	0.000	0.000	0.000	0.000	5.441	7.639	11.172	3.169

Aghvorik AR	Aghvorik	Aghvorik	1.000	0.000	0.000	0.000	0.000	0.000	0.000	0.000	0.000	0.000	0.000	0.000	0.000	0.000	4.456	6.473	12.157	5.316
Aghvorik AR	Aghvorik	Aghvorik	1.000	0.000	0.000	0.000	0.000	0.000	0.000	0.000	0.000	0.000	0.000	0.000	0.000	0.000	4.879	6.855	11.342	4.259
Aghvorik AR	Aghvorik	Aghvorik	1.000	0.000	0.000	0.000	0.000	0.000	0.000	0.000	0.000	0.000	0.000	0.000	0.000	0.000	5.656	6.333	10.206	5.166
Mets Arteni	Arteni	Arteni	0.000	1.000	0.000	0.000	0.000	0.000	0.000	0.000	0.000	0.000	0.000	0.000	0.000	0.000	-3.480	0.744	5.464	-3.889
Mets Arteni	Arteni	Arteni	0.000	1.000	0.000	0.000	0.000	0.000	0.000	0.000	0.000	0.000	0.000	0.000	0.000	0.000	-4.086	2.776	4.334	-4.446
Mets Arteni	Arteni	Arteni	0.000	1.000	0.000	0.000	0.000	0.000	0.000	0.000	0.000	0.000	0.000	0.000	0.000	0.000	-2.362	-0.363	5.409	-3.770
Mets Arteni	Arteni	Arteni	0.000	1.000	0.000	0.000	0.000	0.000	0.000	0.000	0.000	0.000	0.000	0.000	0.000	0.000	-3.640	0.084	5.429	-3.869
Mets Arteni	Arteni	Arteni	0.000	1.000	0.000	0.000	0.000	0.000	0.000	0.000	0.000	0.000	0.000	0.000	0.000	0.000	-3.759	2.087	5.298	-4.142
Mets Arteni	Arteni	Arteni	0.000	1.000	0.000	0.000	0.000	0.000	0.000	0.000	0.000	0.000	0.000	0.000	0.000	0.000	-3.813	0.584	5.037	-3.898
Mets Arteni	Arteni	Arteni	0.000	1.000	0.000	0.000	0.000	0.000	0.000	0.000	0.000	0.000	0.000	0.000	0.000	0.000	-3.287	1.088	6.077	-4.164
Mets Arteni	Arteni	Arteni	0.000	1.000	0.000	0.000	0.000	0.000	0.000	0.000	0.000	0.000	0.000	0.000	0.000	0.000	-3.184	-0.599	4.075	-3.632
Mets Arteni	Arteni	Arteni	0.000	1.000	0.000	0.000	0.000	0.000	0.000	0.000	0.000	0.000	0.000	0.000	0.000	0.000	-3.821	1.332	4.445	-3.788
Mets Arteni	Arteni	Arteni	0.000	1.000	0.000	0.000	0.000	0.000	0.000	0.000	0.000	0.000	0.000	0.000	0.000	0.000	-1.456	-0.103	4.110	-3.779
Mets Arteni	Arteni	Arteni	0.000	1.000	0.000	0.000	0.000	0.000	0.000	0.000	0.000	0.000	0.000	0.000	0.000	0.000	-3.423	-0.358	4.611	-3.553
Pokr Arteni-1	Arteni	Arteni	0.000	1.000	0.000	0.000	0.000	0.000	0.000	0.000	0.000	0.000	0.000	0.000	0.000	0.000	-4.025	0.979	4.382	-3.615
Pokr Arteni-1	Arteni	Arteni	0.000	1.000	0.000	0.000	0.000	0.000	0.000	0.000	0.000	0.000	0.000	0.000	0.000	0.000	-4.399	0.539	4.845	-3.557
Pokr Arteni-1	Arteni	Arteni	0.000	1.000	0.000	0.000	0.000	0.000	0.000	0.000	0.000	0.000	0.000	0.000	0.000	0.000	-4.765	-0.152	4.132	-3.585
Pokr Arteni-1	Arteni	Arteni	0.000	1.000	0.000	0.000	0.000	0.000	0.000	0.000	0.000	0.000	0.000	0.000	0.000	0.000	-4.762	1.131	5.535	-3.606
Pokr Arteni-1	Arteni	Arteni	0.000	1.000	0.000	0.000	0.000	0.000	0.000	0.000	0.000	0.000	0.000	0.000	0.000	0.000	-3.926	0.390	5.226	-3.234
Pokr Arteni-1	Arteni	Arteni	0.000	1.000	0.000	0.000	0.000	0.000	0.000	0.000	0.000	0.000	0.000	0.000	0.000	0.000	-3.894	0.604	3.692	-3.480
Pokr Arteni-1	Arteni	Arteni	0.000	1.000	0.000	0.000	0.000	0.000	0.000	0.000	0.000	0.000	0.000	0.000	0.000	0.000	-4.619	0.967	4.343	-3.721
Pokr Arteni-1	Arteni	Arteni	0.000	1.000	0.000	0.000	0.000	0.000	0.000	0.000	0.000	0.000	0.000	0.000	0.000	0.000	-5.126	1.134	3.551	-4.280
Pokr Arteni-1	Arteni	Arteni	0.000	1.000	0.000	0.000	0.000	0.000	0.000	0.000	0.000	0.000	0.000	0.000	0.000	0.000	-4.799	1.691	4.006	-4.259
Pokr Arteni-1	Arteni	Arteni	0.000	1.000	0.000	0.000	0.000	0.000	0.000	0.000	0.000	0.000	0.000	0.000	0.000	0.000	-4.367	1.102	5.126	-3.632
Pokr Arteni-1	Arteni	Arteni	0.000	1.000	0.000	0.000	0.000	0.000	0.000	0.000	0.000	0.000	0.000	0.000	0.000	0.000	-3.488	0.829	4.847	-3.333
Pokr Arteni-2	Arteni	Arteni	0.000	1.000	0.000	0.000	0.000	0.000	0.000	0.000	0.000	0.000	0.000	0.000	0.000	0.000	-1.682	1.248	4.023	-3.732
Pokr Arteni-2	Arteni	Arteni	0.000	1.000	0.000	0.000	0.000	0.000	0.000	0.000	0.000	0.000	0.000	0.000	0.000	0.000	-1.265	1.211	3.185	-3.921
Pokr Arteni-2	Arteni	Arteni	0.000	1.000	0.000	0.000	0.000	0.000	0.000	0.000	0.000	0.000	0.000	0.000	0.000	0.000	-1.255	0.738	3.473	-3.630
Pokr Arteni-2	Arteni	Arteni	0.000	1.000	0.000	0.000	0.000	0.000	0.000	0.000	0.000	0.000	0.000	0.000	0.000	0.000	-0.733	-0.374	4.416	-2.808
Pokr Arteni-2	Arteni	Arteni	0.000	1.000	0.000	0.000	0.000	0.000	0.000	0.000	0.000	0.000	0.000	0.000	0.000	0.000	-1.561	0.464	4.313	-3.341
Chikiani-1 G	Chikiani	Chikiani	0.000	0.000	1.000	0.000	0.000	0.000	0.000	0.000	0.000	0.000	0.000	0.000	0.000	0.000	11.760	1.298	-3.398	-2.680
Chikiani-1 G	Chikiani	Chikiani	0.000	0.000	1.000	0.000	0.000	0.000	0.000	0.000	0.000	0.000	0.000	0.000	0.000	0.000	12.274	2.409	-4.791	-3.002
Chikiani-1 G	Chikiani	Chikiani	0.000	0.000	1.000	0.000	0.000	0.000	0.000	0.000	0.000	0.000	0.000	0.000	0.000	0.000	12.098	2.498	-4.649	-2.659
Chikiani-1 G	Chikiani	Chikiani	0.000	0.000	1.000	0.000	0.000	0.000	0.000	0.000	0.000	0.000	0.000	0.000	0.000	0.000	12.072	0.666	-3.853	-2.179
Chikiani-1 G	Chikiani	Chikiani	0.000	0.000	1.000	0.000	0.000	0.000	0.000	0.000	0.000	0.000	0.000	0.000	0.000	0.000	12.198	0.813	-3.935	-2.331
Chikiani-1 Sp	Chikiani	Chikiani	0.000	0.000	1.000	0.000	0.000	0.000	0.000	0.000	0.000	0.000	0.000	0.000	0.000	0.000	12.577	-0.006	-4.455	-2.347
Chikiani-2a G	Chikiani	Chikiani	0.000	0.000	1.000	0.000	0.000	0.000	0.000	0.000	0.000	0.000	0.000	0.000	0.000	0.000	13.224	1.603	-4.859	-1.832
Chikiani-2a G	Chikiani	Chikiani	0.000	0.000	1.000	0.000	0.000	0.000	0.000	0.000	0.000	0.000	0.000	0.000	0.000	0.000	12.708	0.102	-1.753	-0.372
Chikiani-2a G	Chikiani	Chikiani	0.000	0.000	1.000	0.000	0.000	0.000	0.000	0.000	0.000	0.000	0.000	0.000	0.000	0.000	11.796	1.058	-2.902	-1.266
Chikiani-2a S	Chikiani	Chikiani	0.000	0.000	1.000	0.000	0.000	0.000	0.000	0.000	0.000	0.000	0.000	0.000	0.000	0.000	13.308	1.280	-3.562	-1.080
Chikiani-2a S	Chikiani	Chikiani	0.000	0.000	1.000	0.000	0.000	0.000	0.000	0.000	0.000	0.000	0.000	0.000	0.000	0.000	12.202	1.061	-4.123	-1.935
Chikiani-2b G	Chikiani	Chikiani	0.000	0.000	1.000	0.000	0.000	0.000	0.000	0.000	0.000	0.000	0.000	0.000	0.000	0.000	13.365	1.777	-3.664	-1.388
Chikiani-2b G	Chikiani	Chikiani	0.000	0.000	1.000	0.000	0.000	0.000	0.000	0.000	0.000	0.000	0.000	0.000	0.000	0.000	12.767	1.066	-3.518	-0.841
Chikiani-2b G	Chikiani	Chikiani	0.000	0.000	1.000	0.000	0.000	0.000	0.000	0.000	0.000	0.000	0.000	0.000	0.000	0.000	12.791	1.192	-2.508	-0.792
Chikiani-2b G	Chikiani	Chikiani	0.000	0.000	1.000	0.000	0.000	0.000	0.000	0.000	0.000	0.000	0.000	0.000	0.000	0.000	11.951	1.888	-2.328	-0.630
Chikiani-2b G	Chikiani	Chikiani	0.000	0.000	1.000	0.000	0.000	0.000	0.000	0.000	0.000	0.000	0.000	0.000	0.000	0.000	13.602	0.938	-3.820	-0.985
Chikiani-2b G	Chikiani	Chikiani	0.000	0.000	1.000	0.000	0.000	0.000	0.000	0.000	0.000	0.000	0.000	0.000	0.000	0.000	12.655	1.670	-3.245	-1.187
Chikiani-2b G	Chikiani	Chikiani	0.000	0.000	1.000	0.000	0.000	0.000	0.000	0.000	0.000	0.000	0.000	0.000	0.000	0.000	12.411	1.955	-3.205	-2.050
Chikiani-2b G	Chikiani	Chikiani	0.000	0.000	1.000	0.000	0.000	0.000	0.000	0.000	0.000	0.000	0.000	0.000	0.000	0.000	13.309	2.441	-3.941	-1.945
Chikiani-2b G	Chikiani	Chikiani	0.000	0.000	1.000	0.000	0.000	0.000	0.000	0.000	0.000	0.000	0.000	0.000	0.000	0.000	12.317	2.123	-3.946	-2.274
Chikiani-2b S	Chikiani	Chikiani	0.000	0.000	1.000	0.000	0.000	0.000	0.000	0.000	0.000	0.000	0.000	0.000	0.000	0.000	12.016	2.173	-2.568	-1.149
Chikiani-2b S	Chikiani	Chikiani	0.000	0.000	1.000	0.000	0.000	0.000	0.000	0.000	0.000	0.000	0.000	0.000	0.000	0.000	13.014	1.808	-3.060	-1.205
Chikiani-2b S	Chikiani	Chikiani	0.000	0.000	1.000	0.000	0.000	0.000	0.000	0.000	0.000	0.000	0.000	0.000	0.000	0.000	12.446	1.667	-3.476	-1.749
Chikiani-3 G	Chikiani	Chikiani	0.000	0.000	1.000	0.000	0.000	0.000	0.000	0.000	0.000	0.000	0.000	0.000	0.000	0.000	12.301	1.851	-3.084	-1.598
Chikiani-3 G	Chikiani	Chikiani	0.000	0.000	1.000	0.000	0.000	0.000	0.000	0.000	0.000	0.000	0.000	0.000	0.000	0.000	13.492	2.433	-3.609	-1.259
Geghasar-1	Gegham 1	Gegham 1	0.000	0.000	0.000	1.000	0.000	0.000	0.000	0.000	0.000	0.000	0.000	0.000	0.000	0.000	-1.665	-13.587	1.567	-5.783
Geghasar-1	Gegham 1	Gegham 1	0.000	0.000	0.000	1.000	0.000	0.000	0.000	0.000	0.000	0.000	0.000	0.000	0.000	0.000	0.814	-12.349	1.755	-6.031
Geghasar-1	Gegham 1	Gegham 1	0.000	0.000	0.000	1.000	0.000	0.000	0.000	0.000	0.000	0.000	0.000	0.000	0.000	0.000	-1.206	-13.902	1.823	-6.031
Geghasar-1	Gegham 1	Gegham 1	0.000	0.000	0.000	1.000	0.000	0.000	0.000	0.000	0.000	0.000	0.000	0.000	0.000	0.000	-1.153	-10.632	3.090	-3.430
Geghasar-1	Gegham 1	Gegham 1	0.000	0.000	0.000	1.000	0.000	0.000	0.000	0.000	0.000	0.000	0.000	0.000	0.000	0.000	-1.819	-12.595	2.367	-4.567
Geghasar-1	Gegham 1	Gegham 1	0.000	0.000	0.000	1.000	0.000	0.000	0.000	0.000	0.000	0.000	0.000	0.000	0.000	0.000	-1.319	-10.705	2.517	-4.726
Geghasar-1	Gegham 1	Gegham 1	0.000	0.000	0.000	1.000	0.000	0.000	0.000	0.000	0.000	0.000	0.000	0.000	0.000	0.000	-1.799	-11.926	2.820	-4.455
Geghasar-1	Gegham 1	Gegham 1	0.000	0.000	0.000	1.000	0.000	0.000	0.000	0.000	0.000	0.000	0.000	0.000	0.000	0.000	-1.497	-11.774	2.810	-4.428
Geghasar-1	Gegham 1	Gegham 1	0.000	0.000	0.000	1.000	0.000	0.000	0.000	0.000	0.000	0.000	0.000	0.000	0.000	0.000	-2.269	-13.324	2.452	-4.572
Geghasar-1	Gegham 1	Gegham 1	0.000	0.000	0.000	1.000	0.000	0.000	0.000	0.000	0.000	0.000	0.000	0.000	0.000	0.000	-0.772	-10.908	2.580	-4.240
Spitakasar-1	Gegham 1	Gegham 1	0.000	0.000	0.000	1.000	0.000	0.000	0.000	0.000	0.000	0.000	0.000	0.000	0.000	0.000	-1.554	-11.936	3.263	-5.421
Spitakasar-1	Gegham 1	Gegham 1	0.000	0.000	0.000	1.000	0.000	0.000	0.000	0.000	0.000	0.000	0.000	0.000	0.000	0.000	-1.792	-10.065	3.198	-5.168

Spitakasar-1	Gegham 1	Gegham 1	0.000	0.000	0.000	1.000	0.000	0.000	0.000	0.000	0.000	0.000	0.000	0.000	0.000	0.000	-1.585	-10.504	3.337	-5.506
Spitakasar-1	Gegham 1	Gegham 1	0.000	0.000	0.000	1.000	0.000	0.000	0.000	0.000	0.000	0.000	0.000	0.000	0.000	0.000	-0.879	-11.150	2.896	-5.772
Spitakasar-1	Gegham 1	Gegham 1	0.000	0.000	0.000	1.000	0.000	0.000	0.000	0.000	0.000	0.000	0.000	0.000	0.000	0.000	-0.571	-12.874	2.235	-6.269
Spitakasar-1	Gegham 1	Gegham 1	0.000	0.000	0.000	1.000	0.000	0.000	0.000	0.000	0.000	0.000	0.000	0.000	0.000	0.000	0.418	-11.505	2.066	-6.148
Spitakasar-1	Gegham 1	Gegham 1	0.000	0.000	0.000	1.000	0.000	0.000	0.000	0.000	0.000	0.000	0.000	0.000	0.000	0.000	-2.341	-12.536	1.695	-5.818
Spitakasar-1	Gegham 1	Gegham 1	0.000	0.000	0.000	1.000	0.000	0.000	0.000	0.000	0.000	0.000	0.000	0.000	0.000	0.000	-2.156	-13.078	1.139	-5.958
Spitakasar-1	Gegham 1	Gegham 1	0.000	0.000	0.000	1.000	0.000	0.000	0.000	0.000	0.000	0.000	0.000	0.000	0.000	0.000	-1.567	-12.943	2.834	-5.975
Spitakasar-1	Gegham 1	Gegham 1	0.000	0.000	0.000	1.000	0.000	0.000	0.000	0.000	0.000	0.000	0.000	0.000	0.000	0.000	-0.858	-11.059	2.537	-5.799
Spitakasar-1	Gegham 1	Gegham 1	0.000	0.000	0.000	1.000	0.000	0.000	0.000	0.000	0.000	0.000	0.000	0.000	0.000	0.000	-0.266	-12.241	2.263	-6.255
Spitakasar/G	Gegham 2	Gegham 2	0.000	0.000	0.000	0.000	1.000	0.000	0.000	0.000	0.000	0.000	0.000	0.000	0.000	0.000	-3.289	4.917	7.571	-3.522
Spitakasar/G	Gegham 2	Gegham 2	0.000	0.000	0.000	0.000	1.000	0.000	0.000	0.000	0.000	0.000	0.000	0.000	0.000	0.000	-2.732	5.274	7.037	-3.627
Spitakasar/G	Gegham 2	Gegham 2	0.000	0.000	0.000	0.000	1.000	0.000	0.000	0.000	0.000	0.000	0.000	0.000	0.000	0.000	-3.154	5.604	8.743	-3.442
Spitakasar/G	Gegham 2	Gegham 2	0.000	0.000	0.000	0.000	1.000	0.000	0.000	0.000	0.000	0.000	0.000	0.000	0.000	0.000	-2.365	5.198	7.179	-3.033
Spitakasar/G	Gegham 2	Gegham 2	0.000	0.000	0.000	0.000	1.000	0.000	0.000	0.000	0.000	0.000	0.000	0.000	0.000	0.000	-2.275	5.109	8.863	-3.107
Spitakasar/G	Gegham 2	Gegham 2	0.000	0.000	0.000	0.000	1.000	0.000	0.000	0.000	0.000	0.000	0.000	0.000	0.000	0.000	-3.008	5.942	9.026	-3.269
Gutansar AR	Gutansar	Gutansar	0.000	0.000	0.000	0.000	0.000	1.000	0.000	0.000	0.000	0.000	0.000	0.000	0.000	0.000	4.574	0.567	11.656	13.308
Gutansar AR	Gutansar	Gutansar	0.000	0.000	0.000	0.000	0.000	1.000	0.000	0.000	0.000	0.000	0.000	0.000	0.000	0.000	5.396	0.628	11.475	13.189
Gutansar AR	Gutansar	Gutansar	0.000	0.000	0.000	0.000	0.000	1.000	0.000	0.000	0.000	0.000	0.000	0.000	0.000	0.000	5.297	-0.451	11.705	14.243
Gutansar AR	Gutansar	Gutansar	0.000	0.000	0.000	0.000	0.000	1.000	0.000	0.000	0.000	0.000	0.000	0.000	0.000	0.000	5.573	1.316	11.266	11.559
Gutansar AR	Gutansar	Gutansar	0.000	0.000	0.000	0.000	0.000	1.000	0.000	0.000	0.000	0.000	0.000	0.000	0.000	0.000	5.338	0.899	11.085	13.230
Gutansar AR	Gutansar	Gutansar	0.000	0.000	0.000	0.000	0.000	1.000	0.000	0.000	0.000	0.000	0.000	0.000	0.000	0.000	5.900	0.820	12.950	13.962
Gutansar AR	Gutansar	Gutansar	0.000	0.000	0.000	0.000	0.000	1.000	0.000	0.000	0.000	0.000	0.000	0.000	0.000	0.000	5.607	1.154	11.726	13.376
Gutansar AR	Gutansar	Gutansar	0.000	0.000	0.000	0.000	0.000	1.000	0.000	0.000	0.000	0.000	0.000	0.000	0.000	0.000	4.880	2.172	11.726	13.051
Gutansar AR	Gutansar	Gutansar	0.000	0.000	0.000	0.000	0.000	1.000	0.000	0.000	0.000	0.000	0.000	0.000	0.000	0.000	5.502	0.404	11.101	11.550
Gutansar AR	Gutansar	Gutansar	0.000	0.000	0.000	0.000	0.000	1.000	0.000	0.000	0.000	0.000	0.000	0.000	0.000	0.000	6.533	-0.424	11.118	13.176
Gutansar AR	Gutansar	Gutansar	0.000	0.000	0.000	0.000	0.000	1.000	0.000	0.000	0.000	0.000	0.000	0.000	0.000	0.000	4.732	1.062	11.395	12.443
Gutansar AR	Gutansar	Gutansar	0.000	0.000	0.000	0.000	0.000	1.000	0.000	0.000	0.000	0.000	0.000	0.000	0.000	0.000	5.053	0.671	11.095	11.529
Gutansar AR	Gutansar	Gutansar	0.000	0.000	0.000	0.000	0.000	1.000	0.000	0.000	0.000	0.000	0.000	0.000	0.000	0.000	4.982	1.210	11.370	12.129
Gutansar AR	Gutansar	Gutansar	0.000	0.000	0.000	0.000	0.000	1.000	0.000	0.000	0.000	0.000	0.000	0.000	0.000	0.000	4.858	1.111	12.662	13.782
Gutansar AR	Gutansar	Gutansar	0.000	0.000	0.000	0.000	0.000	1.000	0.000	0.000	0.000	0.000	0.000	0.000	0.000	0.000	3.568	2.104	12.449	11.744
Gutansar AR	Gutansar	Gutansar	0.000	0.000	0.000	0.000	0.000	1.000	0.000	0.000	0.000	0.000	0.000	0.000	0.000	0.000	5.189	0.513	12.816	14.216
Gutansar AR	Gutansar	Gutansar	0.000	0.000	0.000	0.000	0.000	1.000	0.000	0.000	0.000	0.000	0.000	0.000	0.000	0.000	3.391	1.702	12.191	11.313
Gutansar AR	Gutansar	Gutansar	0.000	0.000	0.000	0.000	0.000	1.000	0.000	0.000	0.000	0.000	0.000	0.000	0.000	0.000	5.413	0.492	11.358	11.919
Gutansar AR	Gutansar	Gutansar	0.000	0.000	0.000	0.000	0.000	1.000	0.000	0.000	0.000	0.000	0.000	0.000	0.000	0.000	5.473	0.041	11.437	12.884
Gutansar AR	Gutansar	Gutansar	0.000	0.000	0.000	0.000	0.000	1.000	0.000	0.000	0.000	0.000	0.000	0.000	0.000	0.000	5.873	2.006	11.505	10.369
Gutansar AR	Gutansar	Gutansar	0.000	0.000	0.000	0.000	0.000	1.000	0.000	0.000	0.000	0.000	0.000	0.000	0.000	0.000	7.260	-0.441	9.755	12.891
Gutansar AR	Gutansar	Gutansar	0.000	0.000	0.000	0.000	0.000	1.000	0.000	0.000	0.000	0.000	0.000	0.000	0.000	0.000	6.013	1.016	12.153	12.421
Gutansar AR	Gutansar	Gutansar	0.000	0.000	0.000	0.000	0.000	1.000	0.000	0.000	0.000	0.000	0.000	0.000	0.000	0.000	4.410	-0.657	13.275	15.910
Gutansar AR	Gutansar	Gutansar	0.000	0.000	0.000	0.000	0.000	1.000	0.000	0.000	0.000	0.000	0.000	0.000	0.000	0.000	5.831	0.220	10.827	14.002
Hatis-Alpha /	Hatis	Hatis	0.000	0.000	0.000	0.000	0.000	0.000	1.000	0.000	0.000	0.000	0.000	0.000	0.000	0.000	17.836	3.999	-2.786	-3.078
Hatis-Alpha /	Hatis	Hatis	0.000	0.000	0.000	0.000	0.000	0.000	1.000	0.000	0.000	0.000	0.000	0.000	0.000	0.000	18.629	3.495	-2.527	-1.985
Hatis-Alpha /	Hatis	Hatis	0.000	0.000	0.000	0.000	0.000	0.000	1.000	0.000	0.000	0.000	0.000	0.000	0.000	0.000	18.716	2.746	-2.283	-2.215
Hatis-Alpha /	Hatis	Hatis	0.000	0.000	0.000	0.000	0.000	0.000	1.000	0.000	0.000	0.000	0.000	0.000	0.000	0.000	17.535	2.672	-2.104	-1.877
Hatis-Alpha /	Hatis	Hatis	0.000	0.000	0.000	0.000	0.000	0.000	1.000	0.000	0.000	0.000	0.000	0.000	0.000	0.000	18.839	3.492	-4.402	-3.106
Hatis-Alpha /	Hatis	Hatis	0.000	0.000	0.000	0.000	0.000	0.000	1.000	0.000	0.000	0.000	0.000	0.000	0.000	0.000	17.503	3.853	-2.870	-2.688
Hatis-Alpha /	Hatis	Hatis	0.000	0.000	0.000	0.000	0.000	0.000	1.000	0.000	0.000	0.000	0.000	0.000	0.000	0.000	17.659	2.267	-1.248	-1.530
Hatis-Alpha /	Hatis	Hatis	0.000	0.000	0.000	0.000	0.000	0.000	1.000	0.000	0.000	0.000	0.000	0.000	0.000	0.000	18.375	2.777	-2.369	-2.055
Hatis-Alpha /	Hatis	Hatis	0.000	0.000	0.000	0.000	0.000	0.000	1.000	0.000	0.000	0.000	0.000	0.000	0.000	0.000	19.352	3.707	-4.333	-3.152
Hatis-Alpha /	Hatis	Hatis	0.000	0.000	0.000	0.000	0.000	0.000	1.000	0.000	0.000	0.000	0.000	0.000	0.000	0.000	19.545	3.633	-2.577	-2.054
Hatis-Alpha /	Hatis	Hatis	0.000	0.000	0.000	0.000	0.000	0.000	1.000	0.000	0.000	0.000	0.000	0.000	0.000	0.000	19.288	2.321	-3.196	-2.036
Hatis-Alpha /	Hatis	Hatis	0.000	0.000	0.000	0.000	0.000	0.000	1.000	0.000	0.000	0.000	0.000	0.000	0.000	0.000	18.290	3.405	-3.460	-2.669
Hatis-Alpha /	Hatis	Hatis	0.000	0.000	0.000	0.000	0.000	0.000	1.000	0.000	0.000	0.000	0.000	0.000	0.000	0.000	18.432	1.182	-1.471	-1.420
Hatis-Alpha /	Hatis	Hatis	0.000	0.000	0.000	0.000	0.000	0.000	1.000	0.000	0.000	0.000	0.000	0.000	0.000	0.000	18.454	3.220	-2.195	-2.053
Hatis-Alpha /	Hatis	Hatis	0.000	0.000	0.000	0.000	0.000	0.000	1.000	0.000	0.000	0.000	0.000	0.000	0.000	0.000	18.855	3.671	-3.839	-3.229
Hatis-Alpha /	Hatis	Hatis	0.000	0.000	0.000	0.000	0.000	0.000	1.000	0.000	0.000	0.000	0.000	0.000	0.000	0.000	17.716	2.369	-2.115	-1.989
Hatis-Beta A	Hatis	Hatis	0.000	0.000	0.000	0.000	0.000	0.000	1.000	0.000	0.000	0.000	0.000	0.000	0.000	0.000	18.654	5.100	-3.070	-3.405
Hatis-Beta A	Hatis	Hatis	0.000	0.000	0.000	0.000	0.000	0.000	1.000	0.000	0.000	0.000	0.000	0.000	0.000	0.000	18.484	5.473	-4.048	-2.800
Hatis-Beta A	Hatis	Hatis	0.000	0.000	0.000	0.000	0.000	0.000	1.000	0.000	0.000	0.000	0.000	0.000	0.000	0.000	20.858	3.740	-4.247	-2.060
Hatis-Beta A	Hatis	Hatis	0.000	0.000	0.000	0.000	0.000	0.000	1.000	0.000	0.000	0.000	0.000	0.000	0.000	0.000	20.899	5.210	-4.551	-2.872
Hatis-Beta G	Hatis	Hatis	0.000	0.000	0.000	0.000	0.000	0.000	1.000	0.000	0.000	0.000	0.000	0.000	0.000	0.000	19.390	3.331	-1.825	-1.473
Hatis-Beta G	Hatis	Hatis	0.000	0.000	0.000	0.000	0.000	0.000	1.000	0.000	0.000	0.000	0.000	0.000	0.000	0.000	18.732	3.304	-2.472	-0.982
Hatis-Beta G	Hatis	Hatis	0.000	0.000	0.000	0.000	0.000	0.000	1.000	0.000	0.000	0.000	0.000	0.000	0.000	0.000	20.093	2.914	-3.295	-1.507
Hatis-Beta G	Hatis	Hatis	0.000	0.000	0.000	0.000	0.000	0.000	1.000	0.000	0.000	0.000	0.000	0.000	0.000	0.000	20.452	4.142	-4.114	-2.312
Hatis-Beta G	Hatis	Hatis	0.000	0.000	0.000	0.000	0.000	0.000	1.000	0.000	0.000	0.000	0.000	0.000	0.000	0.000	19.551	4.072	-3.118	-2.577
Hatis-Beta G	Hatis	Hatis	0.000	0.000	0.000	0.000	0.000	0.000	1.000	0.000	0.000	0.000	0.000	0.000	0.000	0.000	20.254	5.149	-4.475	-3.006
Hatis-Delta 2	Hatis	Hatis	0.000	0.000	0.000	0.000	0.000	0.000	1.000	0.000	0.000	0.000	0.000	0.000	0.000	0.000	19.770	5.753	-4.091	-1.877
Hatis-Delta 2	Hatis	Hatis	0.000	0.000	0.000	0.000	0.000	0.000	1.000	0.000	0.000	0.000	0.000	0.000	0.000	0.000	22.016	5.184	-4.905	-0.832

Hatis-Delta 2	Hatis	Hatis	0.000	0.000	0.000	0.000	0.000	0.000	1.000	0.000	0.000	0.000	0.000	0.000	0.000	0.000	20.577	3.234	-2.927	0.915
Hatis-Gamm	Hatis	Hatis	0.000	0.000	0.000	0.000	0.000	0.000	1.000	0.000	0.000	0.000	0.000	0.000	0.000	0.000	21.816	3.337	-2.777	-0.068
Hatis-Gamm	Hatis	Hatis	0.000	0.000	0.000	0.000	0.000	0.000	1.000	0.000	0.000	0.000	0.000	0.000	0.000	0.000	21.937	3.521	-3.952	-0.878
Hatis-Gamm	Hatis	Hatis	0.000	0.000	0.000	0.000	0.000	0.000	1.000	0.000	0.000	0.000	0.000	0.000	0.000	0.000	23.024	3.479	-2.492	0.905
Hatis-Gamm	Hatis	Hatis	0.000	0.000	0.000	0.000	0.000	0.000	1.000	0.000	0.000	0.000	0.000	0.000	0.000	0.000	21.987	3.433	-2.639	0.536
Hatis-Gamm	Hatis	Hatis	0.000	0.000	0.000	0.000	0.000	0.000	1.000	0.000	0.000	0.000	0.000	0.000	0.000	0.000	22.116	3.612	-1.827	0.865
Hatis-Gamm	Hatis	Hatis	0.000	0.000	0.000	0.000	0.000	0.000	1.000	0.000	0.000	0.000	0.000	0.000	0.000	0.000	20.631	4.024	-4.118	-1.332
Kars-Arpaçay	Kars-Arpaçay	Kars-Arpaçay	0.000	0.000	0.000	0.000	0.000	0.000	0.000	1.000	0.000	0.000	0.000	0.000	0.000	0.000	-17.391	18.606	4.726	-7.264
Kars-Arpaçay	Kars-Arpaçay	Kars-Arpaçay	0.000	0.000	0.000	0.000	0.000	0.000	0.000	1.000	0.000	0.000	0.000	0.000	0.000	0.000	-17.599	19.986	1.935	-5.713
Kars-Arpaçay	Kars-Arpaçay	Kars-Arpaçay	0.000	0.000	0.000	0.000	0.000	0.000	0.000	1.000	0.000	0.000	0.000	0.000	0.000	0.000	-17.592	20.168	2.410	-6.296
Kars-Arpaçay	Kars-Arpaçay	Kars-Arpaçay	0.000	0.000	0.000	0.000	0.000	0.000	0.000	1.000	0.000	0.000	0.000	0.000	0.000	0.000	-16.315	18.176	4.371	-8.349
Kars-Arpaçay	Kars-Arpaçay	Kars-Arpaçay	0.000	0.000	0.000	0.000	0.000	0.000	0.000	1.000	0.000	0.000	0.000	0.000	0.000	0.000	-18.100	19.033	5.344	-5.443
Kars-Arpaçay	Kars-Arpaçay	Kars-Arpaçay	0.000	0.000	0.000	0.000	0.000	0.000	0.000	1.000	0.000	0.000	0.000	0.000	0.000	0.000	-17.225	19.304	4.253	-5.690
Kars-Arpaçay	Kars-Arpaçay	Kars-Arpaçay	0.000	0.000	0.000	0.000	0.000	0.000	0.000	1.000	0.000	0.000	0.000	0.000	0.000	0.000	-16.306	18.703	9.293	-11.314
Kars-Arpaçay	Kars-Arpaçay	Kars-Arpaçay	0.000	0.000	0.000	0.000	0.000	0.000	0.000	1.000	0.000	0.000	0.000	0.000	0.000	0.000	-17.434	22.679	6.256	-3.427
Kars-Arpaçay	Kars-Arpaçay	Kars-Arpaçay	0.000	0.000	0.000	0.000	0.000	0.000	0.000	1.000	0.000	0.000	0.000	0.000	0.000	0.000	-14.047	21.775	6.250	-5.777
Kelbadjar AZ	Kelbadjar	Kelbadjar	0.000	0.000	0.000	0.000	0.000	0.000	0.000	0.000	1.000	0.000	0.000	0.000	0.000	0.000	-9.006	-7.898	2.171	3.227
Kelbadjar AZ	Kelbadjar	Kelbadjar	0.000	0.000	0.000	0.000	0.000	0.000	0.000	0.000	1.000	0.000	0.000	0.000	0.000	0.000	-8.985	-9.616	1.468	2.137
Kelbadjar AZ	Kelbadjar	Kelbadjar	0.000	0.000	0.000	0.000	0.000	0.000	0.000	0.000	1.000	0.000	0.000	0.000	0.000	0.000	-8.652	-8.647	1.708	2.343
Khorapor AR	Khorapor	Khorapor	0.000	0.000	0.000	0.000	0.000	0.000	0.000	0.000	0.000	1.000	0.000	0.000	0.000	0.000	-8.821	-18.936	-3.588	-0.642
Khorapor AR	Khorapor	Khorapor	0.000	0.000	0.000	0.000	0.000	0.000	0.000	0.000	0.000	1.000	0.000	0.000	0.000	0.000	-9.973	-18.321	-4.602	-0.223
Khorapor AR(	Khorapor	Khorapor	0.000	0.000	0.000	0.000	0.000	0.000	0.000	0.000	0.000	1.000	0.000	0.000	0.000	0.000	-9.029	-17.712	-3.897	-0.875
Khorapor AR(	Khorapor	Khorapor	0.000	0.000	0.000	0.000	0.000	0.000	0.000	0.000	0.000	1.000	0.000	0.000	0.000	0.000	-8.257	-17.933	-4.354	-0.730
Khorapor AR(	Khorapor	Khorapor	0.000	0.000	0.000	0.000	0.000	0.000	0.000	0.000	0.000	1.000	0.000	0.000	0.000	0.000	-8.932	-18.835	-3.894	-1.126
Khorapor AR(	Khorapor	Khorapor	0.000	0.000	0.000	0.000	0.000	0.000	0.000	0.000	0.000	1.000	0.000	0.000	0.000	0.000	-8.248	-18.766	-3.888	-0.482
Meydan Dag	Meydan Dağ	Meydan Dağ	0.000	0.000	0.000	0.000	0.000	0.000	0.000	0.000	0.000	0.000	1.000	0.000	0.000	0.000	-18.281	14.069	-18.558	7.056
Meydan Dag	Meydan Dağ	Meydan Dağ	0.000	0.000	0.000	0.000	0.000	0.000	0.000	0.000	0.000	0.000	1.000	0.000	0.000	0.000	-17.661	14.831	-18.727	7.083
Meydan Dag	Meydan Dağ	Meydan Dağ	0.000	0.000	0.000	0.000	0.000	0.000	0.000	0.000	0.000	0.000	1.000	0.000	0.000	0.000	-17.299	11.403	-20.776	9.388
Meydan Dag	Meydan Dağ	Meydan Dağ	0.000	0.000	0.000	0.000	0.000	0.000	0.000	0.000	0.000	0.000	1.000	0.000	0.000	0.000	-18.312	12.902	-17.882	8.173
Meydan Dag	Meydan Dağ	Meydan Dağ	0.000	0.000	0.000	0.000	0.000	0.000	0.000	0.000	0.000	0.000	1.000	0.000	0.000	0.000	-17.565	12.712	-19.313	7.501
Meydan Dag	Meydan Dağ	Meydan Dağ	0.000	0.000	0.000	0.000	0.000	0.000	0.000	0.000	0.000	0.000	1.000	0.000	0.000	0.000	-18.268	14.419	-18.880	7.906
Meydan Dag	Meydan Dağ	Meydan Dağ	0.000	0.000	0.000	0.000	0.000	0.000	0.000	0.000	0.000	0.000	1.000	0.000	0.000	0.000	-18.332	13.020	-19.243	8.645
Meydan Dag	Meydan Dağ	Meydan Dağ	0.000	0.000	0.000	0.000	0.000	0.000	0.000	0.000	0.000	0.000	1.000	0.000	0.000	0.000	-18.425	10.852	-18.789	9.643
Meydan Dag	Meydan Dağ	Meydan Dağ	0.000	0.000	0.000	0.000	0.000	0.000	0.000	0.000	0.000	0.000	1.000	0.000	0.000	0.000	-17.075	12.848	-21.236	9.168
Sarıkamış-1	Sarıkamış	Sarıkamış	0.000	0.000	0.000	0.000	0.000	0.000	0.000	0.000	0.000	0.000	0.000	1.000	0.000	0.000	-8.406	1.270	-4.967	-7.071
Sarıkamış-1	Sarıkamış	Sarıkamış	0.000	0.000	0.000	0.000	0.000	0.000	0.000	0.000	0.000	0.000	0.000	1.000	0.000	0.000	-8.794	1.693	-1.956	-7.050
Sarıkamış-1	Sarıkamış	Sarıkamış	0.000	0.000	0.000	0.000	0.000	0.000	0.000	0.000	0.000	0.000	0.000	1.000	0.000	0.000	-7.712	1.326	-3.067	-5.813
Sarıkamış-1	Sarıkamış	Sarıkamış	0.000	0.000	0.000	0.000	0.000	0.000	0.000	0.000	0.000	0.000	0.000	1.000	0.000	0.000	-8.129	0.672	-3.267	-6.165
Sarıkamış-1	Sarıkamış	Sarıkamış	0.000	0.000	0.000	0.000	0.000	0.000	0.000	0.000	0.000	0.000	0.000	1.000	0.000	0.000	-9.508	2.268	-2.902	-8.272
Sarıkamış-1a	Sarıkamış	Sarıkamış	0.000	0.000	0.000	0.000	0.000	0.000	0.000	0.000	0.000	0.000	0.000	1.000	0.000	0.000	-11.331	1.610	-6.329	-4.578
Sarıkamış-1a	Sarıkamış	Sarıkamış	0.000	0.000	0.000	0.000	0.000	0.000	0.000	0.000	0.000	0.000	0.000	1.000	0.000	0.000	-14.082	3.450	-4.822	-4.086
Sarıkamış-2	Sarıkamış	Sarıkamış	0.000	0.000	0.000	0.000	0.000	0.000	0.000	0.000	0.000	0.000	0.000	1.000	0.000	0.000	-8.429	-0.046	-2.569	-5.481
Sarıkamış-2	Sarıkamış	Sarıkamış	0.000	0.000	0.000	0.000	0.000	0.000	0.000	0.000	0.000	0.000	0.000	1.000	0.000	0.000	-9.482	0.205	-3.500	-5.995
Sarıkamış-2	Sarıkamış	Sarıkamış	0.000	0.000	0.000	0.000	0.000	0.000	0.000	0.000	0.000	0.000	0.000	1.000	0.000	0.000	-9.389	1.224	-3.507	-6.307
Sarıkamış-2	Sarıkamış	Sarıkamış	0.000	0.000	0.000	0.000	0.000	0.000	0.000	0.000	0.000	0.000	0.000	1.000	0.000	0.000	-8.493	0.253	-2.010	-5.686
Sarıkamış-2	Sarıkamış	Sarıkamış	0.000	0.000	0.000	0.000	0.000	0.000	0.000	0.000	0.000	0.000	0.000	1.000	0.000	0.000	-7.625	-0.212	-3.014	-5.289
Sarıkamış-2	Sarıkamış	Sarıkamış	0.000	0.000	0.000	0.000	0.000	0.000	0.000	0.000	0.000	0.000	0.000	1.000	0.000	0.000	-8.667	-0.637	-2.437	-5.504
Bazenk AR38	Syunik	Syunik	0.000	0.000	0.000	0.000	0.000	0.000	0.000	0.000	0.000	0.000	0.000	0.000	1.000	0.000	-10.261	-16.005	-2.905	1.786
Bazenk AR58	Syunik	Syunik	0.000	0.000	0.000	0.000	0.000	0.000	0.000	0.000	0.000	0.000	0.000	0.000	1.000	0.000	-9.908	-15.988	-1.578	1.818
Bazenk AR58	Syunik	Syunik	0.000	0.000	0.000	0.000	0.000	0.000	0.000	0.000	0.000	0.000	0.000	0.000	1.000	0.000	-10.425	-17.519	-2.720	1.917
Bazenk AR58	Syunik	Syunik	0.000	0.000	0.000	0.000	0.000	0.000	0.000	0.000	0.000	0.000	0.000	0.000	1.000	0.000	-9.628	-16.265	-1.871	1.610
Bazenk AR58	Syunik	Syunik	0.000	0.000	0.000	0.000	0.000	0.000	0.000	0.000	0.000	0.000	0.000	0.000	1.000	0.000	-9.784	-16.193	-1.433	2.035
Bazenk AR58	Syunik	Syunik	0.000	0.000	0.000	0.000	0.000	0.000	0.000	0.000	0.000	0.000	0.000	0.000	1.000	0.000	-9.815	-16.224	-1.496	2.226
Bazenk AR58	Syunik	Syunik	0.000	0.000	0.000	0.000	0.000	0.000	0.000	0.000	0.000	0.000	0.000	0.000	1.000	0.000	-9.899	-16.179	-1.974	1.592
Bazenk AR58	Syunik	Syunik	0.000	0.000	0.000	0.000	0.000	0.000	0.000	0.000	0.000	0.000	0.000	0.000	1.000	0.000	-10.709	-17.662	-2.849	1.959
Bazenk AR82	Syunik	Syunik	0.000	0.000	0.000	0.000	0.000	0.000	0.000	0.000	0.000	0.000	0.000	0.000	1.000	0.000	-8.958	-14.786	-3.116	2.510
Bazenk AR82	Syunik	Syunik	0.000	0.000	0.000	0.000	0.000	0.000	0.000	0.000	0.000	0.000	0.000	0.000	1.000	0.000	-9.290	-16.213	-2.552	2.197
Bazenk AR82	Syunik	Syunik	0.000	0.000	0.000	0.000	0.000	0.000	0.000	0.000	0.000	0.000	0.000	0.000	1.000	0.000	-10.233	-15.709	-1.938	1.282
Bazenk AR82	Syunik	Syunik	0.000	0.000	0.000	0.000	0.000	0.000	0.000	0.000	0.000	0.000	0.000	0.000	1.000	0.000	-8.882	-14.825	-2.574	1.324
Bazenk AR82	Syunik	Syunik	0.000	0.000	0.000	0.000	0.000	0.000	0.000	0.000	0.000	0.000	0.000	0.000	1.000	0.000	-10.019	-16.360	-2.380	1.692
Bazenk AR82	Syunik	Syunik	0.000	0.000	0.000	0.000	0.000	0.000	0.000	0.000	0.000	0.000	0.000	0.000	1.000	0.000	-9.537	-16.108	-2.468	1.307
Satanakar-1	Syunik	Syunik	0.000	0.000	0.000	0.000	0.000	0.000	0.000	0.000	0.000	0.000	0.000	0.000	1.000	0.000	-7.752	-15.728	-2.870	1.933
Satanakar-1	Syunik	Syunik	0.000	0.000	0.000	0.000	0.000	0.000	0.000	0.000	0.000	0.000	0.000	0.000	1.000	0.000	-8.128	-16.268	-2.256	2.236
Satanakar-1	Syunik	Syunik	0.000	0.000	0.000	0.000	0.000	0.000	0.000	0.000	0.000	0.000	0.000	0.000	1.000	0.000	-9.065	-15.734	-3.376	1.876
Satanakar-1	Syunik	Syunik	0.000	0.000	0.000	0.000	0.000	0.000	0.000	0.000	0.000	0.000	0.000	0.000	1.000	0.000	-8.801	-15.025	-1.483	1.090
Satanakar-1	Syunik	Syunik	0.000	0.000	0.000	0.000	0.000	0.000	0.000	0.000	0.000	0.000	0.000	0.000	1.000	0.000	-8.409	-14.444	-2.593	1.474
Satanakar-1	Syunik	Syunik	0.000	0.000	0.000	0.000	0.000	0.000	0.000	0.000	0.000	0.000	0.000	0.000	1.000	0.000	-8.357	-16.258	-2.878	1.994

Satanakar-1	Syunik	Syunik	0.000	0.000	0.000	0.000	0.000	0.000	0.000	0.000	0.000	0.000	0.000	0.000	1.000	0.000	-8.571	-15.344	-2.786	1.575
Satanakar-1	Syunik	Syunik	0.000	0.000	0.000	0.000	0.000	0.000	0.000	0.000	0.000	0.000	0.000	0.000	1.000	0.000	-8.720	-15.080	-3.148	1.454
Satanakar-1	Syunik	Syunik	0.000	0.000	0.000	0.000	0.000	0.000	0.000	0.000	0.000	0.000	0.000	0.000	1.000	0.000	-7.848	-16.464	-2.836	2.276
Satanakar-1	Syunik	Syunik	0.000	0.000	0.000	0.000	0.000	0.000	0.000	0.000	0.000	0.000	0.000	0.000	1.000	0.000	-8.334	-15.326	-2.998	1.924
Satanakar-1	Syunik	Syunik	0.000	0.000	0.000	0.000	0.000	0.000	0.000	0.000	0.000	0.000	0.000	0.000	1.000	0.000	-8.053	-14.384	-2.490	1.772
Satanakar-1	Syunik	Syunik	0.000	0.000	0.000	0.000	0.000	0.000	0.000	0.000	0.000	0.000	0.000	0.000	1.000	0.000	-8.501	-16.131	-2.511	1.440
Satanakar-2	Syunik	Syunik	0.000	0.000	0.000	0.000	0.000	0.000	0.000	0.000	0.000	0.000	0.000	0.000	1.000	0.000	-7.180	-16.409	-2.640	2.172
Satanakar-2	Syunik	Syunik	0.000	0.000	0.000	0.000	0.000	0.000	0.000	0.000	0.000	0.000	0.000	0.000	1.000	0.000	-8.423	-18.019	-2.946	1.880
Satanakar-2	Syunik	Syunik	0.000	0.000	0.000	0.000	0.000	0.000	0.000	0.000	0.000	0.000	0.000	0.000	1.000	0.000	-7.578	-16.870	-2.699	1.353
Satanakar-2	Syunik	Syunik	0.000	0.000	0.000	0.000	0.000	0.000	0.000	0.000	0.000	0.000	0.000	0.000	1.000	0.000	-8.843	-18.280	-3.942	1.996
Satanakar-3	Syunik	Syunik	0.000	0.000	0.000	0.000	0.000	0.000	0.000	0.000	0.000	0.000	0.000	0.000	1.000	0.000	-8.483	-17.982	-2.974	2.534
Satanakar-3	Syunik	Syunik	0.000	0.000	0.000	0.000	0.000	0.000	0.000	0.000	0.000	0.000	0.000	0.000	1.000	0.000	-7.609	-16.089	-2.426	1.730
Satanakar-3	Syunik	Syunik	0.000	0.000	0.000	0.000	0.000	0.000	0.000	0.000	0.000	0.000	0.000	0.000	1.000	0.000	-8.967	-18.538	-3.378	1.849
Satanakar-3	Syunik	Syunik	0.000	0.000	0.000	0.000	0.000	0.000	0.000	0.000	0.000	0.000	0.000	0.000	1.000	0.000	-7.774	-16.697	-4.193	2.361
Sevkar AR33	Syunik	Syunik	0.000	0.000	0.000	0.000	0.000	0.000	0.000	0.000	0.000	0.000	0.000	0.000	1.000	0.000	-7.433	-14.051	-0.867	2.742
Sevkar AR33	Syunik	Syunik	0.000	0.000	0.000	0.000	0.000	0.000	0.000	0.000	0.000	0.000	0.000	0.000	1.000	0.000	-6.447	-12.208	-2.069	3.480
Sevkar AR35	Syunik	Syunik	0.000	0.000	0.000	0.000	0.000	0.000	0.000	0.000	0.000	0.000	0.000	0.000	1.000	0.000	-6.761	-13.276	-2.104	3.214
Sevkar AR57	Syunik	Syunik	0.000	0.000	0.000	0.000	0.000	0.000	0.000	0.000	0.000	0.000	0.000	0.000	1.000	0.000	-7.724	-13.603	-1.696	3.171
Sevkar AR57	Syunik	Syunik	0.000	0.000	0.000	0.000	0.000	0.000	0.000	0.000	0.000	0.000	0.000	0.000	1.000	0.000	-6.406	-12.529	-1.704	2.411
Sevkar AR81	Syunik	Syunik	0.000	0.000	0.000	0.000	0.000	0.000	0.000	0.000	0.000	0.000	0.000	0.000	1.000	0.000	-7.968	-13.918	-1.603	2.372
Sevkar AR81	Syunik	Syunik	0.000	0.000	0.000	0.000	0.000	0.000	0.000	0.000	0.000	0.000	0.000	0.000	1.000	0.000	-7.486	-13.768	-1.253	2.185
Sevkar AR81	Syunik	Syunik	0.000	0.000	0.000	0.000	0.000	0.000	0.000	0.000	0.000	0.000	0.000	0.000	1.000	0.000	-7.383	-14.410	-2.157	2.621
Sevkar AR81	Syunik	Syunik	0.000	0.000	0.000	0.000	0.000	0.000	0.000	0.000	0.000	0.000	0.000	0.000	1.000	0.000	-6.929	-12.695	-3.404	2.309
Sevkar GPS 5	Syunik	Syunik	0.000	0.000	0.000	0.000	0.000	0.000	0.000	0.000	0.014	0.000	0.000	0.000	0.986	0.000	-7.012	-12.960	-1.076	3.421
Sevkar GPS 5	Syunik	Syunik	0.000	0.000	0.000	0.000	0.000	0.000	0.000	0.000	0.000	0.000	0.000	0.000	1.000	0.000	-7.235	-12.316	-1.874	3.116
Sevkar GPS 5	Syunik	Syunik	0.000	0.000	0.000	0.000	0.000	0.000	0.000	0.000	0.000	0.000	0.000	0.000	1.000	0.000	-6.445	-12.386	-1.194	2.504
Sevkar GPS 5	Syunik	Syunik	0.000	0.000	0.000	0.000	0.000	0.000	0.000	0.000	0.000	0.000	0.000	0.000	1.000	0.000	-6.893	-12.498	-2.564	2.504
Tsaghk-1 Kar	Tsaghkunyat	Tsaghkunyats	0.000	0.000	0.000	0.000	0.000	0.000	0.000	0.000	0.000	0.000	0.000	0.000	0.000	1.000	32.679	2.633	-9.002	-2.648
Tsaghk-1 Kar	Tsaghkunyat	Tsaghkunyats	0.000	0.000	0.000	0.000	0.000	0.000	0.000	0.000	0.000	0.000	0.000	0.000	0.000	1.000	34.417	1.422	-8.010	-0.260
Tsaghk-1 Kar	Tsaghkunyat	Tsaghkunyats	0.000	0.000	0.000	0.000	0.000	0.000	0.000	0.000	0.000	0.000	0.000	0.000	0.000	1.000	34.261	0.762	-6.492	0.981
Tsaghk-1 Kar	Tsaghkunyat	Tsaghkunyats	0.000	0.000	0.000	0.000	0.000	0.000	0.000	0.000	0.000	0.000	0.000	0.000	0.000	1.000	34.652	1.464	-8.156	0.054
Tsaghk-1 Kar	Tsaghkunyat	Tsaghkunyats	0.000	0.000	0.000	0.000	0.000	0.000	0.000	0.000	0.000	0.000	0.000	0.000	0.000	1.000	34.611	2.345	-10.707	-3.872
Tsaghk-1 Kar	Tsaghkunyat	Tsaghkunyats	0.000	0.000	0.000	0.000	0.000	0.000	0.000	0.000	0.000	0.000	0.000	0.000	0.000	1.000	32.812	1.836	-7.104	0.174
Tsaghk-1 Kar	Tsaghkunyat	Tsaghkunyats	0.000	0.000	0.000	0.000	0.000	0.000	0.000	0.000	0.000	0.000	0.000	0.000	0.000	1.000	33.335	2.712	-8.699	-1.719
Tsaghk-2 Tta	Tsaghkunyat	Tsaghkunyats	0.000	0.000	0.000	0.000	0.000	0.000	0.000	0.000	0.000	0.000	0.000	0.000	0.000	1.000	35.607	2.935	-11.262	-3.024
Tsaghk-2 Tta	Tsaghkunyat	Tsaghkunyats	0.000	0.000	0.000	0.000	0.000	0.000	0.000	0.000	0.000	0.000	0.000	0.000	0.000	1.000	36.825	1.633	-8.868	-0.567
Tsaghk-2 Tta	Tsaghkunyat	Tsaghkunyats	0.000	0.000	0.000	0.000	0.000	0.000	0.000	0.000	0.000	0.000	0.000	0.000	0.000	1.000	35.253	2.864	-10.023	-2.264
Tsaghk-2 Tta	Tsaghkunyat	Tsaghkunyats	0.000	0.000	0.000	0.000	0.000	0.000	0.000	0.000	0.000	0.000	0.000	0.000	0.000	1.000	36.173	1.684	-8.234	-1.313
Tsaghk-2 Tta	Tsaghkunyat	Tsaghkunyats	0.000	0.000	0.000	0.000	0.000	0.000	0.000	0.000	0.000	0.000	0.000	0.000	0.000	1.000	35.077	2.961	-9.172	-1.734
Tsaghk-2 Tta	Tsaghkunyat	Tsaghkunyats	0.000	0.000	0.000	0.000	0.000	0.000	0.000	0.000	0.000	0.000	0.000	0.000	0.000	1.000	33.556	2.236	-8.228	-1.873
Tsaghk-2 Tta	Tsaghkunyat	Tsaghkunyats	0.000	0.000	0.000	0.000	0.000	0.000	0.000	0.000	0.000	0.000	0.000	0.000	0.000	1.000	34.976	2.034	-9.308	-2.195
Tsaghk-2 Tta	Tsaghkunyat	Tsaghkunyats	0.000	0.000	0.000	0.000	0.000	0.000	0.000	0.000	0.000	0.000	0.000	0.000	0.000	1.000	36.400	2.253	-9.859	-1.897
Tsaghk-2 Tta	Tsaghkunyat	Tsaghkunyats	0.000	0.000	0.000	0.000	0.000	0.000	0.000	0.000	0.000	0.000	0.000	0.000	0.000	1.000	35.322	2.491	-9.867	-1.576
Tsaghk-2 Tta	Tsaghkunyat	Tsaghkunyats	0.000	0.000	0.000	0.000	0.000	0.000	0.000	0.000	0.000	0.000	0.000	0.000	0.000	1.000	36.403	3.877	-11.589	-2.892
Tsaghk-2 Tta	Tsaghkunyat	Tsaghkunyats	0.000	0.000	0.000	0.000	0.000	0.000	0.000	0.000	0.000	0.000	0.000	0.000	0.000	1.000	35.000	2.161	-9.818	-2.529
Tsaghk-2 Tta	Tsaghkunyat	Tsaghkunyats	0.000	0.000	0.000	0.000	0.000	0.000	0.000	0.000	0.000	0.000	0.000	0.000	0.000	1.000	34.772	1.676	-8.194	-0.717
Tsaghk-2 Tta	Tsaghkunyat	Tsaghkunyats	0.000	0.000	0.000	0.000	0.000	0.000	0.000	0.000	0.000	0.000	0.000	0.000	0.000	1.000	33.949	3.365	-9.286	-2.513
Tsaghk-2 Tta	Tsaghkunyat	Tsaghkunyats	0.000	0.000	0.000	0.000	0.000	0.000	0.000	0.000	0.000	0.000	0.000	0.000	0.000	1.000	34.031	2.535	-7.837	-0.931
Tsaghk-2 Tta	Tsaghkunyat	Tsaghkunyats	0.000	0.000	0.000	0.000	0.000	0.000	0.000	0.000	0.000	0.000	0.000	0.000	0.000	1.000	35.450	3.664	-11.469	-3.738
Tsaghk-3 Da	Tsaghkunyat	Tsaghkunyats	0.000	0.000	0.000	0.000	0.000	0.000	0.000	0.000	0.000	0.000	0.000	0.000	0.000	1.000	34.581	1.625	-7.894	-0.165
Tsaghk-3 Da	Tsaghkunyat	Tsaghkunyats	0.000	0.000	0.000	0.000	0.000	0.000	0.000	0.000	0.000	0.000	0.000	0.000	0.000	1.000	34.523	1.741	-8.653	-0.358
Tsaghk-3 Da	Tsaghkunyat	Tsaghkunyats	0.000	0.000	0.000	0.000	0.000	0.000	0.000	0.000	0.000	0.000	0.000	0.000	0.000	1.000	33.751	1.687	-7.086	0.210
Tsaghk-3 Da	Tsaghkunyat	Tsaghkunyats	0.000	0.000	0.000	0.000	0.000	0.000	0.000	0.000	0.000	0.000	0.000	0.000	0.000	1.000	33.151	2.667	-7.999	-0.060
Tsaghk-3 Da	Tsaghkunyat	Tsaghkunyats	0.000	0.000	0.000	0.000	0.000	0.000	0.000	0.000	0.000	0.000	0.000	0.000	0.000	1.000	32.157	3.713	-9.251	-2.021
Tsaghk-2 Tta	Tsaghkunyat	Tsaghkunyats	0.000	0.000	0.000	0.000	0.000	0.000	0.000	0.000	0.000	0.000	0.000	0.000	0.000	1.000	34.424	2.259	-9.208	-2.225

The results corresponding to the validation set are displayed in the second part of the table

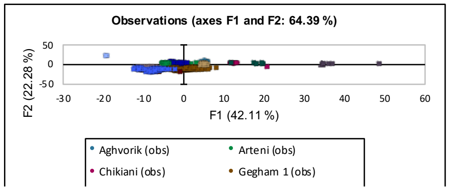

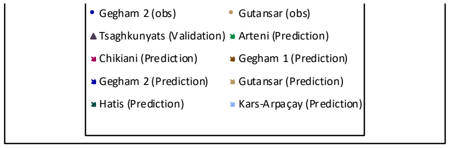

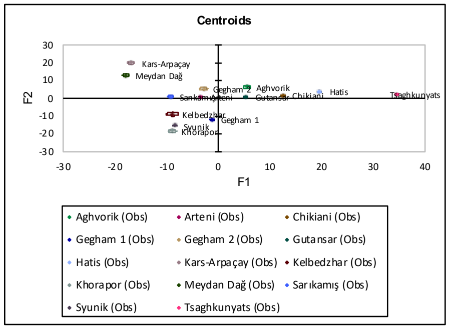

Confusion matrix for the training sample:

from \ to	Aghvorik	Arteni	Chikiani	Gegham 1	Gegham 2	Gutansar	Hatis	Kars-Arpaçay	Kelbadjar	Khorapor	Meydan Dağ	Sarıkamış	Syunik	Tsaghkunyats	Total	% correct
Aghvorik	19	0	0	0	0	0	0	0	0	0	0	0	0	0	19	100.00%
Arteni	0	19	0	0	0	0	0	0	0	0	0	0	0	0	19	100.00%
Chikiani	0	0	19	0	0	0	0	0	0	0	0	0	0	0	19	100.00%
Gegham 1	0	0	0	19	0	0	0	0	0	0	0	0	0	0	19	100.00%
Gegham 2	0	0	0	0	19	0	0	0	0	0	0	0	0	0	19	100.00%
Gutansar	0	0	0	0	0	19	0	0	0	0	0	0	0	0	19	100.00%
Hatis	0	0	0	0	0	0	19	0	0	0	0	0	0	0	19	100.00%
Kars-Arpaçay	0	0	0	0	0	0	0	19	0	0	0	0	0	0	19	100.00%
Kelbadjar	0	0	0	0	0	0	0	0	19	0	0	0	0	0	19	100.00%
Khorapor	0	0	0	0	0	0	0	0	0	19	0	0	0	0	19	100.00%
Meydan Dağ	0	0	0	0	0	0	0	0	0	0	19	0	0	0	19	100.00%
Sarıkamış	0	0	0	0	0	0	0	0	0	0	0	19	0	0	19	100.00%
Syunik	0	0	0	0	0	0	0	0	0	0	0	0	19	0	19	100.00%
Tsaghkunyats	0	0	0	0	0	0	0	0	0	0	0	0	0	19	19	100.00%
Total	19	19	19	19	19	19	19	19	19	19	19	19	19	19	260	100.00%

Confusion matrix for the validation sample:

from \ to	Aghvorik	Arteni	Chikiani	Gegham 1	Gegham 2	Gutansar	Hatis	Kars-Arpaçay	Kelbadjar	Khorapor	Meydan Dağ	Sarıkamış	Syunik	Tsaghkunyats	Total	% correct
Aghvorik	0	0	0	0	0	0	0	0	0	0	0	0	0	0	0	0.00%
Arteni	0	0	0	0	0	0	0	0	0	0	0	0	0	0	0	0.00%
Chikiani	0	0	0	0	0	0	0	0	0	0	0	0	0	0	0	0.00%
Gegham 1	0	0	0	0	0	0	0	0	0	0	0	0	0	0	0	0.00%
Gegham 2	0	0	0	0	0	0	0	0	0	0	0	0	0	0	0	0.00%
Gutansar	0	0	0	0	0	0	0	0	0	0	0	0	0	0	0	0.00%
Hatis	0	0	0	0	0	0	0	0	0	0	0	0	0	0	0	0.00%
Kars-Arpaçay	0	0	0	0	0	0	0	0	0	0	0	0	0	0	0	0.00%
Kelbadjar	0	0	0	0	0	0	0	0	0	0	0	0	0	0	0	0.00%
Khorapor	0	0	0	0	0	0	0	0	0	0	0	0	0	0	0	0.00%
Meydan Dağ	0	0	0	0	0	0	0	0	0	0	0	0	0	0	0	0.00%
Sarıkamış	0	0	0	0	0	0	0	0	0	0	0	0	0	0	0	0.00%
Syunik	0	0	0	0	0	0	0	0	0	0	0	0	0	0	0	0.00%
Tsaghkunyats	0	0	0	0	0	0	0	0	0	0	0	0	0	1	1	100.00%

Total	0	0	0	0	0	0	0	0	0	0	0	0	0	1	1	100.00%

Cross-validation: Prior and posterior classification, membership probabilities, scores and squared distances:

Observation	Prior	Posterior	Aghvorik	Arteni	Chikiani	Gegham 1	Gegham 2	Gutansar	Hatis	Kars-Arpaçay	Kelbadjar	Khorapor	Meydan Dağ	Sarıkamış	Syunik	Tsaghkunyats
Aghvorik AR6	Aghvorik	Aghvorik	1.000	0.000	0.000	0.000	0.000	0.000	0.000	0.000	0.000	0.000	0.000	0.000	0.000	0.000
Aghvorik AR6	Aghvorik	Aghvorik	1.000	0.000	0.000	0.000	0.000	0.000	0.000	0.000	0.000	0.000	0.000	0.000	0.000	0.000
Aghvorik AR6	Aghvorik	Aghvorik	1.000	0.000	0.000	0.000	0.000	0.000	0.000	0.000	0.000	0.000	0.000	0.000	0.000	0.000
Aghvorik AR6	Aghvorik	Aghvorik	1.000	0.000	0.000	0.000	0.000	0.000	0.000	0.000	0.000	0.000	0.000	0.000	0.000	0.000
Aghvorik AR6	Aghvorik	Aghvorik	1.000	0.000	0.000	0.000	0.000	0.000	0.000	0.000	0.000	0.000	0.000	0.000	0.000	0.000
Aghvorik AR6	Aghvorik	Aghvorik	1.000	0.000	0.000	0.000	0.000	0.000	0.000	0.000	0.000	0.000	0.000	0.000	0.000	0.000
Aghvorik AR6	Aghvorik	Aghvorik	1.000	0.000	0.000	0.000	0.000	0.000	0.000	0.000	0.000	0.000	0.000	0.000	0.000	0.000
Aghvorik AR6	Aghvorik	Aghvorik	1.000	0.000	0.000	0.000	0.000	0.000	0.000	0.000	0.000	0.000	0.000	0.000	0.000	0.000
Mets Arteni	Arteni	Arteni	0.000	1.000	0.000	0.000	0.000	0.000	0.000	0.000	0.000	0.000	0.000	0.000	0.000	0.000
Mets Arteni	Arteni	Arteni	0.000	1.000	0.000	0.000	0.000	0.000	0.000	0.000	0.000	0.000	0.000	0.000	0.000	0.000
Mets Arteni	Arteni	Arteni	0.000	1.000	0.000	0.000	0.000	0.000	0.000	0.000	0.000	0.000	0.000	0.000	0.000	0.000
Mets Arteni	Arteni	Arteni	0.000	1.000	0.000	0.000	0.000	0.000	0.000	0.000	0.000	0.000	0.000	0.000	0.000	0.000
Mets Arteni	Arteni	Arteni	0.000	1.000	0.000	0.000	0.000	0.000	0.000	0.000	0.000	0.000	0.000	0.000	0.000	0.000
Mets Arteni	Arteni	Arteni	0.000	1.000	0.000	0.000	0.000	0.000	0.000	0.000	0.000	0.000	0.000	0.000	0.000	0.000
Mets Arteni	Arteni	Arteni	0.000	1.000	0.000	0.000	0.000	0.000	0.000	0.000	0.000	0.000	0.000	0.000	0.000	0.000
Mets Arteni	Arteni	Arteni	0.000	1.000	0.000	0.000	0.000	0.000	0.000	0.000	0.000	0.000	0.000	0.000	0.000	0.000
Mets Arteni	Arteni	Arteni	0.000	1.000	0.000	0.000	0.000	0.000	0.000	0.000	0.000	0.000	0.000	0.000	0.000	0.000
Mets Arteni	Arteni	Arteni	0.000	1.000	0.000	0.000	0.000	0.000	0.000	0.000	0.000	0.000	0.000	0.000	0.000	0.000
Mets Arteni	Arteni	Arteni	0.000	1.000	0.000	0.000	0.000	0.000	0.000	0.000	0.000	0.000	0.000	0.000	0.000	0.000
Pokr Arteni-1	Arteni	Arteni	0.000	1.000	0.000	0.000	0.000	0.000	0.000	0.000	0.000	0.000	0.000	0.000	0.000	0.000
Pokr Arteni-1	Arteni	Arteni	0.000	1.000	0.000	0.000	0.000	0.000	0.000	0.000	0.000	0.000	0.000	0.000	0.000	0.000
Pokr Arteni-1	Arteni	Arteni	0.000	1.000	0.000	0.000	0.000	0.000	0.000	0.000	0.000	0.000	0.000	0.000	0.000	0.000
Pokr Arteni-1	Arteni	Arteni	0.000	1.000	0.000	0.000	0.000	0.000	0.000	0.000	0.000	0.000	0.000	0.000	0.000	0.000
Pokr Arteni-1	Arteni	Arteni	0.000	1.000	0.000	0.000	0.000	0.000	0.000	0.000	0.000	0.000	0.000	0.000	0.000	0.000
Pokr Arteni-1	Arteni	Arteni	0.000	1.000	0.000	0.000	0.000	0.000	0.000	0.000	0.000	0.000	0.000	0.000	0.000	0.000
Pokr Arteni-1	Arteni	Arteni	0.000	1.000	0.000	0.000	0.000	0.000	0.000	0.000	0.000	0.000	0.000	0.000	0.000	0.000
Pokr Arteni-1	Arteni	Arteni	0.000	1.000	0.000	0.000	0.000	0.000	0.000	0.000	0.000	0.000	0.000	0.000	0.000	0.000
Pokr Arteni-1	Arteni	Arteni	0.000	1.000	0.000	0.000	0.000	0.000	0.000	0.000	0.000	0.000	0.000	0.000	0.000	0.000
Pokr Arteni-1	Arteni	Arteni	0.000	1.000	0.000	0.000	0.000	0.000	0.000	0.000	0.000	0.000	0.000	0.000	0.000	0.000
Pokr Arteni-1	Arteni	Arteni	0.000	1.000	0.000	0.000	0.000	0.000	0.000	0.000	0.000	0.000	0.000	0.000	0.000	0.000
Pokr Arteni-2	Arteni	Arteni	0.000	1.000	0.000	0.000	0.000	0.000	0.000	0.000	0.000	0.000	0.000	0.000	0.000	0.000
Pokr Arteni-2	Arteni	Arteni	0.000	1.000	0.000	0.000	0.000	0.000	0.000	0.000	0.000	0.000	0.000	0.000	0.000	0.000
Pokr Arteni-2	Arteni	Arteni	0.000	1.000	0.000	0.000	0.000	0.000	0.000	0.000	0.000	0.000	0.000	0.000	0.000	0.000
Pokr Arteni-2	Arteni	Arteni	0.000	1.000	0.000	0.000	0.000	0.000	0.000	0.000	0.000	0.000	0.000	0.000	0.000	0.000
Pokr Arteni-2	Arteni	Arteni	0.000	1.000	0.000	0.000	0.000	0.000	0.000	0.000	0.000	0.000	0.000	0.000	0.000	0.000
Chikiani-1 GE	Chikiani	Chikiani	0.000	0.000	1.000	0.000	0.000	0.000	0.000	0.000	0.000	0.000	0.000	0.000	0.000	0.000
Chikiani-1 GE	Chikiani	Chikiani	0.000	0.000	1.000	0.000	0.000	0.000	0.000	0.000	0.000	0.000	0.000	0.000	0.000	0.000
Chikiani-1 GE	Chikiani	Chikiani	0.000	0.000	1.000	0.000	0.000	0.000	0.000	0.000	0.000	0.000	0.000	0.000	0.000	0.000
Chikiani-1 GE	Chikiani	Chikiani	0.000	0.000	1.000	0.000	0.000	0.000	0.000	0.000	0.000	0.000	0.000	0.000	0.000	0.000
Chikiani-1 GE	Chikiani	Chikiani	0.000	0.000	1.000	0.000	0.000	0.000	0.000	0.000	0.000	0.000	0.000	0.000	0.000	0.000
Chikiani-1 Sp	Chikiani	Chikiani	0.000	0.000	1.000	0.000	0.000	0.000	0.000	0.000	0.000	0.000	0.000	0.000	0.000	0.000
Chikiani-2a G	Chikiani	Chikiani	0.000	0.000	1.000	0.000	0.000	0.000	0.000	0.000	0.000	0.000	0.000	0.000	0.000	0.000
Chikiani-2a G	Chikiani	Chikiani	0.000	0.000	1.000	0.000	0.000	0.000	0.000	0.000	0.000	0.000	0.000	0.000	0.000	0.000
Chikiani-2a G	Chikiani	Chikiani	0.000	0.000	1.000	0.000	0.000	0.000	0.000	0.000	0.000	0.000	0.000	0.000	0.000	0.000
Chikiani-2a S	Chikiani	Chikiani	0.000	0.000	1.000	0.000	0.000	0.000	0.000	0.000	0.000	0.000	0.000	0.000	0.000	0.000
Chikiani-2a S	Chikiani	Chikiani	0.000	0.000	1.000	0.000	0.000	0.000	0.000	0.000	0.000	0.000	0.000	0.000	0.000	0.000
Chikiani-2b G	Chikiani	Chikiani	0.000	0.000	1.000	0.000	0.000	0.000	0.000	0.000	0.000	0.000	0.000	0.000	0.000	0.000
Chikiani-2b G	Chikiani	Chikiani	0.000	0.000	1.000	0.000	0.000	0.000	0.000	0.000	0.000	0.000	0.000	0.000	0.000	0.000
Chikiani-2b G	Chikiani	Chikiani	0.000	0.000	1.000	0.000	0.000	0.000	0.000	0.000	0.000	0.000	0.000	0.000	0.000	0.000
Chikiani-2b G	Chikiani	Chikiani	0.000	0.000	1.000	0.000	0.000	0.000	0.000	0.000	0.000	0.000	0.000	0.000	0.000	0.000
Chikiani-2b G	Chikiani	Chikiani	0.000	0.000	1.000	0.000	0.000	0.000	0.000	0.000	0.000	0.000	0.000	0.000	0.000	0.000
Chikiani-2b G	Chikiani	Chikiani	0.000	0.000	1.000	0.000	0.000	0.000	0.000	0.000	0.000	0.000	0.000	0.000	0.000	0.000
Chikiani-2b G	Chikiani	Chikiani	0.000	0.000	1.000	0.000	0.000	0.000	0.000	0.000	0.000	0.000	0.000	0.000	0.000	0.000
Chikiani-2b G	Chikiani	Chikiani	0.000	0.000	1.000	0.000	0.000	0.000	0.000	0.000	0.000	0.000	0.000	0.000	0.000	0.000
Chikiani-2b G	Chikiani	Chikiani	0.000	0.000	1.000	0.000	0.000	0.000	0.000	0.000	0.000	0.000	0.000	0.000	0.000	0.000
Chikiani-2b S	Chikiani	Chikiani	0.000	0.000	1.000	0.000	0.000	0.000	0.000	0.000	0.000	0.000	0.000	0.000	0.000	0.000
Chikiani-2b S	Chikiani	Chikiani	0.000	0.000	1.000	0.000	0.000	0.000	0.000	0.000	0.000	0.000	0.000	0.000	0.000	0.000
Chikiani-2b S	Chikiani	Chikiani	0.000	0.000	1.000	0.000	0.000	0.000	0.000	0.000	0.000	0.000	0.000	0.000	0.000	0.000
Chikiani-3 GE	Chikiani	Chikiani	0.000	0.000	1.000	0.000	0.000	0.000	0.000	0.000	0.000	0.000	0.000	0.000	0.000	0.000

Chikiani-3 G	Chikiani	Chikiani	0.000	0.000	1.000	0.000	0.000	0.000	0.000	0.000	0.000	0.000	0.000	0.000	0.000	0.000
Geghasar-1 /	Gegham 1	Gegham 1	0.000	0.000	0.000	1.000	0.000	0.000	0.000	0.000	0.000	0.000	0.000	0.000	0.000	0.000
Geghasar-1 /	Gegham 1	Gegham 1	0.000	0.000	0.000	1.000	0.000	0.000	0.000	0.000	0.000	0.000	0.000	0.000	0.000	0.000
Geghasar-1 /	Gegham 1	Gegham 1	0.000	0.000	0.000	1.000	0.000	0.000	0.000	0.000	0.000	0.000	0.000	0.000	0.000	0.000
Geghasar-1 /	Gegham 1	Gegham 1	0.000	0.000	0.000	1.000	0.000	0.000	0.000	0.000	0.000	0.000	0.000	0.000	0.000	0.000
Geghasar-1 /	Gegham 1	Gegham 1	0.000	0.000	0.000	1.000	0.000	0.000	0.000	0.000	0.000	0.000	0.000	0.000	0.000	0.000
Geghasar-1 /	Gegham 1	Gegham 1	0.000	0.000	0.000	1.000	0.000	0.000	0.000	0.000	0.000	0.000	0.000	0.000	0.000	0.000
Geghasar-1 /	Gegham 1	Gegham 1	0.000	0.000	0.000	1.000	0.000	0.000	0.000	0.000	0.000	0.000	0.000	0.000	0.000	0.000
Geghasar-1 /	Gegham 1	Gegham 1	0.000	0.000	0.000	1.000	0.000	0.000	0.000	0.000	0.000	0.000	0.000	0.000	0.000	0.000
Geghasar-1 /	Gegham 1	Gegham 1	0.000	0.000	0.000	1.000	0.000	0.000	0.000	0.000	0.000	0.000	0.000	0.000	0.000	0.000
Geghasar-1 /	Gegham 1	Gegham 1	0.000	0.000	0.000	1.000	0.000	0.000	0.000	0.000	0.000	0.000	0.000	0.000	0.000	0.000
Spitakasar-1	Gegham 1	Gegham 1	0.000	0.000	0.000	1.000	0.000	0.000	0.000	0.000	0.000	0.000	0.000	0.000	0.000	0.000
Spitakasar-1	Gegham 1	Gegham 1	0.000	0.000	0.000	1.000	0.000	0.000	0.000	0.000	0.000	0.000	0.000	0.000	0.000	0.000
Spitakasar-1	Gegham 1	Gegham 1	0.000	0.000	0.000	1.000	0.000	0.000	0.000	0.000	0.000	0.000	0.000	0.000	0.000	0.000
Spitakasar-1	Gegham 1	Gegham 1	0.000	0.000	0.000	1.000	0.000	0.000	0.000	0.000	0.000	0.000	0.000	0.000	0.000	0.000
Spitakasar-1	Gegham 1	Gegham 1	0.000	0.000	0.000	1.000	0.000	0.000	0.000	0.000	0.000	0.000	0.000	0.000	0.000	0.000
Spitakasar-1	Gegham 1	Gegham 1	0.000	0.000	0.000	1.000	0.000	0.000	0.000	0.000	0.000	0.000	0.000	0.000	0.000	0.000
Spitakasar-1	Gegham 1	Gegham 1	0.000	0.000	0.000	1.000	0.000	0.000	0.000	0.000	0.000	0.000	0.000	0.000	0.000	0.000
Spitakasar-1	Gegham 1	Gegham 1	0.000	0.000	0.000	1.000	0.000	0.000	0.000	0.000	0.000	0.000	0.000	0.000	0.000	0.000
Spitakasar-1	Gegham 1	Gegham 1	0.000	0.000	0.000	1.000	0.000	0.000	0.000	0.000	0.000	0.000	0.000	0.000	0.000	0.000
Spitakasar-1	Gegham 1	Gegham 1	0.000	0.000	0.000	1.000	0.000	0.000	0.000	0.000	0.000	0.000	0.000	0.000	0.000	0.000
Spitakasar-1	Gegham 1	Gegham 1	0.000	0.000	0.000	1.000	0.000	0.000	0.000	0.000	0.000	0.000	0.000	0.000	0.000	0.000
Spitakasar/G	Gegham 2	Gegham 2	0.000	0.000	0.000	0.000	1.000	0.000	0.000	0.000	0.000	0.000	0.000	0.000	0.000	0.000
Spitakasar/G	Gegham 2	Gegham 2	0.000	0.000	0.000	0.000	1.000	0.000	0.000	0.000	0.000	0.000	0.000	0.000	0.000	0.000
Spitakasar/G	Gegham 2	Gegham 2	0.000	0.000	0.000	0.000	1.000	0.000	0.000	0.000	0.000	0.000	0.000	0.000	0.000	0.000
Spitakasar/G	Gegham 2	Gegham 2	0.000	0.000	0.000	0.000	1.000	0.000	0.000	0.000	0.000	0.000	0.000	0.000	0.000	0.000
Spitakasar/G	Gegham 2	Gegham 2	0.000	0.000	0.000	0.000	1.000	0.000	0.000	0.000	0.000	0.000	0.000	0.000	0.000	0.000
Spitakasar/G	Gegham 2	Gegham 2	0.000	0.000	0.000	0.000	1.000	0.000	0.000	0.000	0.000	0.000	0.000	0.000	0.000	0.000
Gutansar AR	Gutansar	Gutansar	0.000	0.000	0.000	0.000	0.000	1.000	0.000	0.000	0.000	0.000	0.000	0.000	0.000	0.000
Gutansar AR	Gutansar	Gutansar	0.000	0.000	0.000	0.000	0.000	1.000	0.000	0.000	0.000	0.000	0.000	0.000	0.000	0.000
Gutansar AR	Gutansar	Gutansar	0.000	0.000	0.000	0.000	0.000	1.000	0.000	0.000	0.000	0.000	0.000	0.000	0.000	0.000
Gutansar AR	Gutansar	Gutansar	0.000	0.000	0.000	0.000	0.000	1.000	0.000	0.000	0.000	0.000	0.000	0.000	0.000	0.000
Gutansar AR	Gutansar	Gutansar	0.000	0.000	0.000	0.000	0.000	1.000	0.000	0.000	0.000	0.000	0.000	0.000	0.000	0.000
Gutansar AR	Gutansar	Gutansar	0.000	0.000	0.000	0.000	0.000	1.000	0.000	0.000	0.000	0.000	0.000	0.000	0.000	0.000
Gutansar AR	Gutansar	Gutansar	0.000	0.000	0.000	0.000	0.000	1.000	0.000	0.000	0.000	0.000	0.000	0.000	0.000	0.000
Gutansar AR	Gutansar	Gutansar	0.000	0.000	0.000	0.000	0.000	1.000	0.000	0.000	0.000	0.000	0.000	0.000	0.000	0.000
Gutansar AR	Gutansar	Gutansar	0.000	0.000	0.000	0.000	0.000	1.000	0.000	0.000	0.000	0.000	0.000	0.000	0.000	0.000
Gutansar AR	Gutansar	Gutansar	0.000	0.000	0.000	0.000	0.000	1.000	0.000	0.000	0.000	0.000	0.000	0.000	0.000	0.000
Gutansar AR	Gutansar	Gutansar	0.000	0.000	0.000	0.000	0.000	1.000	0.000	0.000	0.000	0.000	0.000	0.000	0.000	0.000
Gutansar AR	Gutansar	Gutansar	0.000	0.000	0.000	0.000	0.000	1.000	0.000	0.000	0.000	0.000	0.000	0.000	0.000	0.000
Gutansar AR	Gutansar	Gutansar	0.000	0.000	0.000	0.000	0.000	1.000	0.000	0.000	0.000	0.000	0.000	0.000	0.000	0.000
Gutansar AR	Gutansar	Gutansar	0.000	0.000	0.000	0.000	0.000	1.000	0.000	0.000	0.000	0.000	0.000	0.000	0.000	0.000
Gutansar AR	Gutansar	Gutansar	0.000	0.000	0.000	0.000	0.000	1.000	0.000	0.000	0.000	0.000	0.000	0.000	0.000	0.000
Gutansar AR	Gutansar	Gutansar	0.000	0.000	0.000	0.000	0.000	1.000	0.000	0.000	0.000	0.000	0.000	0.000	0.000	0.000
Gutansar AR	Gutansar	Gutansar	0.000	0.000	0.000	0.000	0.000	1.000	0.000	0.000	0.000	0.000	0.000	0.000	0.000	0.000
Gutansar AR	Gutansar	Gutansar	0.000	0.000	0.000	0.000	0.000	1.000	0.000	0.000	0.000	0.000	0.000	0.000	0.000	0.000
Gutansar AR	Gutansar	Gutansar	0.000	0.000	0.000	0.000	0.000	1.000	0.000	0.000	0.000	0.000	0.000	0.000	0.000	0.000
Gutansar AR	Gutansar	Gutansar	0.000	0.000	0.000	0.000	0.000	1.000	0.000	0.000	0.000	0.000	0.000	0.000	0.000	0.000
Gutansar AR	Gutansar	Gutansar	0.000	0.000	0.000	0.000	0.000	1.000	0.000	0.000	0.000	0.000	0.000	0.000	0.000	0.000
Gutansar AR	Gutansar	Gutansar	0.000	0.000	0.000	0.000	0.000	1.000	0.000	0.000	0.000	0.000	0.000	0.000	0.000	0.000
Gutansar AR	Gutansar	Gutansar	0.000	0.000	0.000	0.000	0.000	1.000	0.000	0.000	0.000	0.000	0.000	0.000	0.000	0.000
Gutansar AR	Gutansar	Gutansar	0.000	0.000	0.000	0.000	0.000	1.000	0.000	0.000	0.000	0.000	0.000	0.000	0.000	0.000
Hatis-Alpha /	Hatis	Hatis	0.000	0.000	0.000	0.000	0.000	0.000	1.000	0.000	0.000	0.000	0.000	0.000	0.000	0.000
Hatis-Alpha /	Hatis	Hatis	0.000	0.000	0.000	0.000	0.000	0.000	1.000	0.000	0.000	0.000	0.000	0.000	0.000	0.000
Hatis-Alpha /	Hatis	Hatis	0.000	0.000	0.000	0.000	0.000	0.000	1.000	0.000	0.000	0.000	0.000	0.000	0.000	0.000
Hatis-Alpha /	Hatis	Hatis	0.000	0.000	0.000	0.000	0.000	0.000	1.000	0.000	0.000	0.000	0.000	0.000	0.000	0.000
Hatis-Alpha /	Hatis	Hatis	0.000	0.000	0.000	0.000	0.000	0.000	1.000	0.000	0.000	0.000	0.000	0.000	0.000	0.000
Hatis-Alpha /	Hatis	Hatis	0.000	0.000	0.000	0.000	0.000	0.000	1.000	0.000	0.000	0.000	0.000	0.000	0.000	0.000
Hatis-Alpha /	Hatis	Hatis	0.000	0.000	0.000	0.000	0.000	0.000	1.000	0.000	0.000	0.000	0.000	0.000	0.000	0.000
Hatis-Alpha /	Hatis	Hatis	0.000	0.000	0.000	0.000	0.000	0.000	1.000	0.000	0.000	0.000	0.000	0.000	0.000	0.000
Hatis-Alpha /	Hatis	Hatis	0.000	0.000	0.000	0.000	0.000	0.000	1.000	0.000	0.000	0.000	0.000	0.000	0.000	0.000
Hatis-Alpha /	Hatis	Hatis	0.000	0.000	0.000	0.000	0.000	0.000	1.000	0.000	0.000	0.000	0.000	0.000	0.000	0.000
Hatis-Alpha /	Hatis	Hatis	0.000	0.000	0.000	0.000	0.000	0.000	1.000	0.000	0.000	0.000	0.000	0.000	0.000	0.000
Hatis-Alpha /	Hatis	Hatis	0.000	0.000	0.000	0.000	0.000	0.000	1.000	0.000	0.000	0.000	0.000	0.000	0.000	0.000
Hatis-Alpha /	Hatis	Hatis	0.000	0.000	0.000	0.000	0.000	0.000	1.000	0.000	0.000	0.000	0.000	0.000	0.000	0.000
Hatis-Alpha /	Hatis	Hatis	0.000	0.000	0.000	0.000	0.000	0.000	1.000	0.000	0.000	0.000	0.000	0.000	0.000	0.000
Hatis-Alpha /	Hatis	Hatis	0.000	0.000	0.000	0.000	0.000	0.000	1.000	0.000	0.000	0.000	0.000	0.000	0.000	0.000

Hatis-Alpha	Hatis	Hatis	0.000	0.000	0.000	0.000	0.000	0.000	1.000	0.000	0.000	0.000	0.000	0.000	0.000	0.000
Hatis-Beta A	Hatis	Hatis	0.000	0.000	0.000	0.000	0.000	0.000	1.000	0.000	0.000	0.000	0.000	0.000	0.000	0.000
Hatis-Beta A	Hatis	Hatis	0.000	0.000	0.000	0.000	0.000	0.000	1.000	0.000	0.000	0.000	0.000	0.000	0.000	0.000
Hatis-Beta A	Hatis	Hatis	0.000	0.000	0.000	0.000	0.000	0.000	1.000	0.000	0.000	0.000	0.000	0.000	0.000	0.000
Hatis-Beta A	Hatis	Hatis	0.000	0.000	0.000	0.000	0.000	0.000	1.000	0.000	0.000	0.000	0.000	0.000	0.000	0.000
Hatis-Beta G	Hatis	Hatis	0.000	0.000	0.000	0.000	0.000	0.000	1.000	0.000	0.000	0.000	0.000	0.000	0.000	0.000
Hatis-Beta G	Hatis	Hatis	0.000	0.000	0.000	0.000	0.000	0.000	1.000	0.000	0.000	0.000	0.000	0.000	0.000	0.000
Hatis-Beta G	Hatis	Hatis	0.000	0.000	0.000	0.000	0.000	0.000	1.000	0.000	0.000	0.000	0.000	0.000	0.000	0.000
Hatis-Beta G	Hatis	Hatis	0.000	0.000	0.000	0.000	0.000	0.000	1.000	0.000	0.000	0.000	0.000	0.000	0.000	0.000
Hatis-Beta G	Hatis	Hatis	0.000	0.000	0.000	0.000	0.000	0.000	1.000	0.000	0.000	0.000	0.000	0.000	0.000	0.000
Hatis-Beta G	Hatis	Hatis	0.000	0.000	0.000	0.000	0.000	0.000	1.000	0.000	0.000	0.000	0.000	0.000	0.000	0.000
Hatis-Delta 2	Hatis	Hatis	0.000	0.000	0.000	0.000	0.000	0.000	1.000	0.000	0.000	0.000	0.000	0.000	0.000	0.000
Hatis-Delta 2	Hatis	Hatis	0.000	0.000	0.000	0.000	0.000	0.000	1.000	0.000	0.000	0.000	0.000	0.000	0.000	0.000
Hatis-Delta 2	Hatis	Hatis	0.000	0.000	0.000	0.000	0.000	0.000	1.000	0.000	0.000	0.000	0.000	0.000	0.000	0.000
Hatis-Gamm	Hatis	Hatis	0.000	0.000	0.000	0.000	0.000	0.000	1.000	0.000	0.000	0.000	0.000	0.000	0.000	0.000
Hatis-Gamm	Hatis	Hatis	0.000	0.000	0.000	0.000	0.000	0.000	1.000	0.000	0.000	0.000	0.000	0.000	0.000	0.000
Hatis-Gamm	Hatis	Hatis	0.000	0.000	0.000	0.000	0.000	0.000	1.000	0.000	0.000	0.000	0.000	0.000	0.000	0.000
Hatis-Gamm	Hatis	Hatis	0.000	0.000	0.000	0.000	0.000	0.000	1.000	0.000	0.000	0.000	0.000	0.000	0.000	0.000
Hatis-Gamm	Hatis	Hatis	0.000	0.000	0.000	0.000	0.000	0.000	1.000	0.000	0.000	0.000	0.000	0.000	0.000	0.000
Hatis-Gamm	Hatis	Hatis	0.000	0.000	0.000	0.000	0.000	0.000	1.000	0.000	0.000	0.000	0.000	0.000	0.000	0.000
Kars-Arpaçay	Kars-Arpaça	Kars-Arpaçay	0.000	0.000	0.000	0.000	0.000	0.000	0.000	1.000	0.000	0.000	0.000	0.000	0.000	0.000
Kars-Arpaçay	Kars-Arpaça	Kars-Arpaçay	0.000	0.000	0.000	0.000	0.000	0.000	0.000	1.000	0.000	0.000	0.000	0.000	0.000	0.000
Kars-Arpaçay	Kars-Arpaça	Kars-Arpaçay	0.000	0.000	0.000	0.000	0.000	0.000	0.000	1.000	0.000	0.000	0.000	0.000	0.000	0.000
Kars-Arpaçay	Kars-Arpaça	Kars-Arpaçay	0.000	0.000	0.000	0.000	0.000	0.000	0.000	1.000	0.000	0.000	0.000	0.000	0.000	0.000
Kars-Arpaçay	Kars-Arpaça	Kars-Arpaçay	0.000	0.000	0.000	0.000	0.000	0.000	0.000	1.000	0.000	0.000	0.000	0.000	0.000	0.000
Kars-Arpaçay	Kars-Arpaça	Kars-Arpaçay	0.000	0.000	0.000	0.000	0.000	0.000	0.000	1.000	0.000	0.000	0.000	0.000	0.000	0.000
Kars-Arpaçay	Kars-Arpaça	Kars-Arpaçay	0.000	0.000	0.000	0.000	0.000	0.000	0.000	1.000	0.000	0.000	0.000	0.000	0.000	0.000
Kars-Arpaçay	Kars-Arpaça	Kars-Arpaçay	0.000	0.000	0.000	0.000	0.000	0.000	0.000	1.000	0.000	0.000	0.000	0.000	0.000	0.000
Kars-Arpaçay	Kars-Arpaça	Kars-Arpaçay	0.000	0.000	0.000	0.000	0.000	0.000	0.000	1.000	0.000	0.000	0.000	0.000	0.000	0.000
Kelbadjar AZ	Kelbadjar	Kelbadjar	0.000	0.000	0.000	0.000	0.000	0.000	0.000	0.000	1.000	0.000	0.000	0.000	0.000	0.000
Kelbadjar AZ	Kelbadjar	Kelbadjar	0.000	0.000	0.000	0.000	0.000	0.000	0.000	0.000	1.000	0.000	0.000	0.000	0.000	0.000
Kelbadjar AZ	Kelbadjar	Kelbadjar	0.000	0.000	0.000	0.000	0.000	0.000	0.000	0.000	1.000	0.000	0.000	0.000	0.000	0.000
Khorapor AR	Khorapor	Khorapor	0.000	0.000	0.000	0.000	0.000	0.000	0.000	0.000	0.000	1.000	0.000	0.000	0.000	0.000
Khorapor AR	Khorapor	Khorapor	0.000	0.000	0.000	0.000	0.000	0.000	0.000	0.000	0.000	1.000	0.000	0.000	0.000	0.000
Khorapor AR	Khorapor	Khorapor	0.000	0.000	0.000	0.000	0.000	0.000	0.000	0.000	0.000	1.000	0.000	0.000	0.000	0.000
Khorapor AR	Khorapor	Khorapor	0.000	0.000	0.000	0.000	0.000	0.000	0.000	0.000	0.000	1.000	0.000	0.000	0.000	0.000
Khorapor AR	Khorapor	Khorapor	0.000	0.000	0.000	0.000	0.000	0.000	0.000	0.000	0.000	1.000	0.000	0.000	0.000	0.000
Khorapor AR	Khorapor	Khorapor	0.000	0.000	0.000	0.000	0.000	0.000	0.000	0.000	0.000	1.000	0.000	0.000	0.000	0.000
Meydan Dag	Meydan Dağ	Meydan Dağ	0.000	0.000	0.000	0.000	0.000	0.000	0.000	0.000	0.000	0.000	1.000	0.000	0.000	0.000
Meydan Dag	Meydan Dağ	Meydan Dağ	0.000	0.000	0.000	0.000	0.000	0.000	0.000	0.000	0.000	0.000	1.000	0.000	0.000	0.000
Meydan Dag	Meydan Dağ	Meydan Dağ	0.000	0.000	0.000	0.000	0.000	0.000	0.000	0.000	0.000	0.000	1.000	0.000	0.000	0.000
Meydan Dag	Meydan Dağ	Meydan Dağ	0.000	0.000	0.000	0.000	0.000	0.000	0.000	0.000	0.000	0.000	1.000	0.000	0.000	0.000
Meydan Dag	Meydan Dağ	Meydan Dağ	0.000	0.000	0.000	0.000	0.000	0.000	0.000	0.000	0.000	0.000	1.000	0.000	0.000	0.000
Meydan Dag	Meydan Dağ	Meydan Dağ	0.000	0.000	0.000	0.000	0.000	0.000	0.000	0.000	0.000	0.000	1.000	0.000	0.000	0.000
Meydan Dag	Meydan Dağ	Meydan Dağ	0.000	0.000	0.000	0.000	0.000	0.000	0.000	0.000	0.000	0.000	1.000	0.000	0.000	0.000
Meydan Dag	Meydan Dağ	Meydan Dağ	0.000	0.000	0.000	0.000	0.000	0.000	0.000	0.000	0.000	0.000	1.000	0.000	0.000	0.000
Meydan Dag	Meydan Dağ	Meydan Dağ	0.000	0.000	0.000	0.000	0.000	0.000	0.000	0.000	0.000	0.000	1.000	0.000	0.000	0.000
Sarıkamış-1	Sarıkamış	Sarıkamış	0.000	0.000	0.000	0.000	0.000	0.000	0.000	0.000	0.000	0.000	0.000	1.000	0.000	0.000
Sarıkamış-1	Sarıkamış	Sarıkamış	0.000	0.000	0.000	0.000	0.000	0.000	0.000	0.000	0.000	0.000	0.000	1.000	0.000	0.000
Sarıkamış-1	Sarıkamış	Sarıkamış	0.000	0.000	0.000	0.000	0.000	0.000	0.000	0.000	0.000	0.000	0.000	1.000	0.000	0.000
Sarıkamış-1	Sarıkamış	Sarıkamış	0.000	0.000	0.000	0.000	0.000	0.000	0.000	0.000	0.000	0.000	0.000	1.000	0.000	0.000
Sarıkamış-1	Sarıkamış	Sarıkamış	0.000	0.000	0.000	0.000	0.000	0.000	0.000	0.000	0.000	0.000	0.000	1.000	0.000	0.000
Sarıkamış-1a	Sarıkamış	Sarıkamış	0.000	0.000	0.000	0.000	0.000	0.000	0.000	0.000	0.000	0.000	0.000	1.000	0.000	0.000
Sarıkamış-1a	Sarıkamış	Sarıkamış	0.000	0.000	0.000	0.000	0.000	0.000	0.000	0.000	0.000	0.000	0.000	1.000	0.000	0.000
Sarıkamış-2	Sarıkamış	Sarıkamış	0.000	0.000	0.000	0.000	0.000	0.000	0.000	0.000	0.000	0.000	0.000	1.000	0.000	0.000
Sarıkamış-2	Sarıkamış	Sarıkamış	0.000	0.000	0.000	0.000	0.000	0.000	0.000	0.000	0.000	0.000	0.000	1.000	0.000	0.000
Sarıkamış-2	Sarıkamış	Sarıkamış	0.000	0.000	0.000	0.000	0.000	0.000	0.000	0.000	0.000	0.000	0.000	1.000	0.000	0.000
Sarıkamış-2	Sarıkamış	Sarıkamış	0.000	0.000	0.000	0.000	0.000	0.000	0.000	0.000	0.000	0.000	0.000	1.000	0.000	0.000
Sarıkamış-2	Sarıkamış	Sarıkamış	0.000	0.000	0.000	0.000	0.000	0.000	0.000	0.000	0.000	0.000	0.000	1.000	0.000	0.000
Sarıkamış-2	Sarıkamış	Sarıkamış	0.000	0.000	0.000	0.000	0.000	0.000	0.000	0.000	0.000	0.000	0.000	1.000	0.000	0.000
Bazenk AR38	Syunik	Syunik	0.000	0.000	0.000	0.000	0.000	0.000	0.000	0.000	0.000	0.000	0.000	0.000	1.000	0.000
Bazenk AR58	Syunik	Syunik	0.000	0.000	0.000	0.000	0.000	0.000	0.000	0.000	0.000	0.000	0.000	0.000	1.000	0.000
Bazenk AR58	Syunik	Syunik	0.000	0.000	0.000	0.000	0.000	0.000	0.000	0.000	0.000	0.000	0.000	0.000	1.000	0.000
Bazenk AR58	Syunik	Syunik	0.000	0.000	0.000	0.000	0.000	0.000	0.000	0.000	0.000	0.000	0.000	0.000	1.000	0.000
Bazenk AR58	Syunik	Syunik	0.000	0.000	0.000	0.000	0.000	0.000	0.000	0.000	0.000	0.000	0.000	0.000	1.000	0.000
Bazenk AR58	Syunik	Syunik	0.000	0.000	0.000	0.000	0.000	0.000	0.000	0.000	0.000	0.000	0.000	0.000	1.000	0.000
Bazenk AR58	Syunik	Syunik	0.000	0.000	0.000	0.000	0.000	0.000	0.000	0.000	0.000	0.000	0.000	0.000	1.000	0.000

Bazenk AR58	Syunik	Syunik	0.000	0.000	0.000	0.000	0.000	0.000	0.000	0.000	0.000	0.000	0.000	0.000	1.000	0.000
Bazenk AR82	Syunik	Syunik	0.000	0.000	0.000	0.000	0.000	0.000	0.000	0.000	0.000	0.000	0.000	0.000	1.000	0.000
Bazenk AR82	Syunik	Syunik	0.000	0.000	0.000	0.000	0.000	0.000	0.000	0.000	0.000	0.000	0.000	0.000	1.000	0.000
Bazenk AR82	Syunik	Syunik	0.000	0.000	0.000	0.000	0.000	0.000	0.000	0.000	0.000	0.000	0.000	0.000	1.000	0.000
Bazenk AR82	Syunik	Syunik	0.000	0.000	0.000	0.000	0.000	0.000	0.000	0.000	0.000	0.000	0.000	0.000	1.000	0.000
Bazenk AR82	Syunik	Syunik	0.000	0.000	0.000	0.000	0.000	0.000	0.000	0.000	0.000	0.000	0.000	0.000	1.000	0.000
Bazenk AR82	Syunik	Syunik	0.000	0.000	0.000	0.000	0.000	0.000	0.000	0.000	0.000	0.000	0.000	0.000	1.000	0.000
Satanakar-1	Syunik	Syunik	0.000	0.000	0.000	0.000	0.000	0.000	0.000	0.000	0.000	0.000	0.000	0.000	1.000	0.000
Satanakar-1	Syunik	Syunik	0.000	0.000	0.000	0.000	0.000	0.000	0.000	0.000	0.000	0.000	0.000	0.000	1.000	0.000
Satanakar-1	Syunik	Syunik	0.000	0.000	0.000	0.000	0.000	0.000	0.000	0.000	0.000	0.000	0.000	0.000	1.000	0.000
Satanakar-1	Syunik	Syunik	0.000	0.000	0.000	0.000	0.000	0.000	0.000	0.000	0.000	0.000	0.000	0.000	1.000	0.000
Satanakar-1	Syunik	Syunik	0.000	0.000	0.000	0.000	0.000	0.000	0.000	0.000	0.000	0.000	0.000	0.000	1.000	0.000
Satanakar-1	Syunik	Syunik	0.000	0.000	0.000	0.000	0.000	0.000	0.000	0.000	0.000	0.000	0.000	0.000	1.000	0.000
Satanakar-1	Syunik	Syunik	0.000	0.000	0.000	0.000	0.000	0.000	0.000	0.000	0.000	0.000	0.000	0.000	1.000	0.000
Satanakar-1	Syunik	Syunik	0.000	0.000	0.000	0.000	0.000	0.000	0.000	0.000	0.000	0.000	0.000	0.000	1.000	0.000
Satanakar-1	Syunik	Syunik	0.000	0.000	0.000	0.000	0.000	0.000	0.000	0.000	0.000	0.000	0.000	0.000	1.000	0.000
Satanakar-1	Syunik	Syunik	0.000	0.000	0.000	0.000	0.000	0.000	0.000	0.000	0.000	0.000	0.000	0.000	1.000	0.000
Satanakar-1	Syunik	Syunik	0.000	0.000	0.000	0.000	0.000	0.000	0.000	0.000	0.000	0.000	0.000	0.000	1.000	0.000
Satanakar-1	Syunik	Syunik	0.000	0.000	0.000	0.000	0.000	0.000	0.000	0.000	0.000	0.000	0.000	0.000	1.000	0.000
Satanakar-2	Syunik	Syunik	0.000	0.000	0.000	0.000	0.000	0.000	0.000	0.000	0.000	0.000	0.000	0.000	1.000	0.000
Satanakar-2	Syunik	Syunik	0.000	0.000	0.000	0.000	0.000	0.000	0.000	0.000	0.000	0.000	0.000	0.000	1.000	0.000
Satanakar-2	Syunik	Syunik	0.000	0.000	0.000	0.000	0.000	0.000	0.000	0.000	0.000	0.000	0.000	0.000	1.000	0.000
Satanakar-2	Syunik	Syunik	0.000	0.000	0.000	0.000	0.000	0.000	0.000	0.000	0.000	0.000	0.000	0.000	1.000	0.000
Satanakar-3	Syunik	Syunik	0.000	0.000	0.000	0.000	0.000	0.000	0.000	0.000	0.000	0.000	0.000	0.000	1.000	0.000
Satanakar-3	Syunik	Syunik	0.000	0.000	0.000	0.000	0.000	0.000	0.000	0.000	0.000	0.000	0.000	0.000	1.000	0.000
Satanakar-3	Syunik	Syunik	0.000	0.000	0.000	0.000	0.000	0.000	0.000	0.000	0.000	0.000	0.000	0.000	1.000	0.000
Satanakar-3	Syunik	Syunik	0.000	0.000	0.000	0.000	0.000	0.000	0.000	0.000	0.000	0.000	0.000	0.000	1.000	0.000
Sevkar AR33	Syunik	Syunik	0.000	0.000	0.000	0.000	0.000	0.000	0.000	0.000	0.000	0.000	0.000	0.000	1.000	0.000
Sevkar AR33	Syunik	Syunik	0.000	0.000	0.000	0.000	0.000	0.000	0.000	0.000	0.000	0.000	0.000	0.000	1.000	0.000
Sevkar AR35	Syunik	Syunik	0.000	0.000	0.000	0.000	0.000	0.000	0.000	0.000	0.000	0.000	0.000	0.000	1.000	0.000
Sevkar AR57	Syunik	Syunik	0.000	0.000	0.000	0.000	0.000	0.000	0.000	0.000	0.000	0.000	0.000	0.000	1.000	0.000
Sevkar AR57	Syunik	Syunik	0.000	0.000	0.000	0.000	0.000	0.000	0.000	0.000	0.000	0.000	0.000	0.000	1.000	0.000
Sevkar AR81	Syunik	Syunik	0.000	0.000	0.000	0.000	0.000	0.000	0.000	0.000	0.000	0.000	0.000	0.000	1.000	0.000
Sevkar AR81	Syunik	Syunik	0.000	0.000	0.000	0.000	0.000	0.000	0.000	0.000	0.000	0.000	0.000	0.000	1.000	0.000
Sevkar AR81	Syunik	Syunik	0.000	0.000	0.000	0.000	0.000	0.000	0.000	0.000	0.000	0.000	0.000	0.000	1.000	0.000
Sevkar AR81	Syunik	Syunik	0.000	0.000	0.000	0.000	0.000	0.000	0.000	0.000	0.000	0.000	0.000	0.000	1.000	0.000
Sevkar GPS 5	Syunik	Syunik	0.000	0.000	0.000	0.000	0.000	0.000	0.000	0.000	0.347	0.000	0.000	0.000	0.653	0.000
Sevkar GPS 5	Syunik	Syunik	0.000	0.000	0.000	0.000	0.000	0.000	0.000	0.000	0.000	0.000	0.000	0.000	1.000	0.000
Sevkar GPS 5	Syunik	Syunik	0.000	0.000	0.000	0.000	0.000	0.000	0.000	0.000	0.001	0.000	0.000	0.000	0.999	0.000
Sevkar GPS 5	Syunik	Syunik	0.000	0.000	0.000	0.000	0.000	0.000	0.000	0.000	0.000	0.000	0.000	0.000	1.000	0.000
Tsaghk-1 Kar	Tsaghkunyat	Tsaghkunyats	0.000	0.000	0.000	0.000	0.000	0.000	0.000	0.000	0.000	0.000	0.000	0.000	0.000	1.000
Tsaghk-1 Kar	Tsaghkunyat	Tsaghkunyats	0.000	0.000	0.000	0.000	0.000	0.000	0.000	0.000	0.000	0.000	0.000	0.000	0.000	1.000
Tsaghk-1 Kar	Tsaghkunyat	Tsaghkunyats	0.000	0.000	0.000	0.000	0.000	0.000	0.000	0.000	0.000	0.000	0.000	0.000	0.000	1.000
Tsaghk-1 Kar	Tsaghkunyat	Tsaghkunyats	0.000	0.000	0.000	0.000	0.000	0.000	0.000	0.000	0.000	0.000	0.000	0.000	0.000	1.000
Tsaghk-1 Kar	Tsaghkunyat	Tsaghkunyats	0.000	0.000	0.000	0.000	0.000	0.000	0.000	0.000	0.000	0.000	0.000	0.000	0.000	1.000
Tsaghk-1 Kar	Tsaghkunyat	Tsaghkunyats	0.000	0.000	0.000	0.000	0.000	0.000	0.000	0.000	0.000	0.000	0.000	0.000	0.000	1.000
Tsaghk-1 Kar	Tsaghkunyat	Tsaghkunyats	0.000	0.000	0.000	0.000	0.000	0.000	0.000	0.000	0.000	0.000	0.000	0.000	0.000	1.000
Tsaghk-2 Tta	Tsaghkunyat	Tsaghkunyats	0.000	0.000	0.000	0.000	0.000	0.000	0.000	0.000	0.000	0.000	0.000	0.000	0.000	1.000
Tsaghk-2 Tta	Tsaghkunyat	Tsaghkunyats	0.000	0.000	0.000	0.000	0.000	0.000	0.000	0.000	0.000	0.000	0.000	0.000	0.000	1.000
Tsaghk-2 Tta	Tsaghkunyat	Tsaghkunyats	0.000	0.000	0.000	0.000	0.000	0.000	0.000	0.000	0.000	0.000	0.000	0.000	0.000	1.000
Tsaghk-2 Tta	Tsaghkunyat	Tsaghkunyats	0.000	0.000	0.000	0.000	0.000	0.000	0.000	0.000	0.000	0.000	0.000	0.000	0.000	1.000
Tsaghk-2 Tta	Tsaghkunyat	Tsaghkunyats	0.000	0.000	0.000	0.000	0.000	0.000	0.000	0.000	0.000	0.000	0.000	0.000	0.000	1.000
Tsaghk-2 Tta	Tsaghkunyat	Tsaghkunyats	0.000	0.000	0.000	0.000	0.000	0.000	0.000	0.000	0.000	0.000	0.000	0.000	0.000	1.000
Tsaghk-2 Tta	Tsaghkunyat	Tsaghkunyats	0.000	0.000	0.000	0.000	0.000	0.000	0.000	0.000	0.000	0.000	0.000	0.000	0.000	1.000
Tsaghk-2 Tta	Tsaghkunyat	Tsaghkunyats	0.000	0.000	0.000	0.000	0.000	0.000	0.000	0.000	0.000	0.000	0.000	0.000	0.000	1.000
Tsaghk-2 Tta	Tsaghkunyat	Tsaghkunyats	0.000	0.000	0.000	0.000	0.000	0.000	0.000	0.000	0.000	0.000	0.000	0.000	0.000	1.000
Tsaghk-2 Tta	Tsaghkunyat	Tsaghkunyats	0.000	0.000	0.000	0.000	0.000	0.000	0.000	0.000	0.000	0.000	0.000	0.000	0.000	1.000
Tsaghk-2 Tta	Tsaghkunyat	Tsaghkunyats	0.000	0.000	0.000	0.000	0.000	0.000	0.000	0.000	0.000	0.000	0.000	0.000	0.000	1.000
Tsaghk-2 Tta	Tsaghkunyat	Tsaghkunyats	0.000	0.000	0.000	0.000	0.000	0.000	0.000	0.000	0.000	0.000	0.000	0.000	0.000	1.000
Tsaghk-2 Tta	Tsaghkunyat	Tsaghkunyats	0.000	0.000	0.000	0.000	0.000	0.000	0.000	0.000	0.000	0.000	0.000	0.000	0.000	1.000
Tsaghk-2 Tta	Tsaghkunyat	Tsaghkunyats	0.000	0.000	0.000	0.000	0.000	0.000	0.000	0.000	0.000	0.000	0.000	0.000	0.000	1.000
Tsaghk-2 Tta	Tsaghkunyat	Tsaghkunyats	0.000	0.000	0.000	0.000	0.000	0.000	0.000	0.000	0.000	0.000	0.000	0.000	0.000	1.000
Tsaghk-2 Tta	Tsaghkunyat	Tsaghkunyats	0.000	0.000	0.000	0.000	0.000	0.000	0.000	0.000	0.000	0.000	0.000	0.000	0.000	1.000
Tsaghk-2 Tta	Tsaghkunyat	Tsaghkunyats	0.000	0.000	0.000	0.000	0.000	0.000	0.000	0.000	0.000	0.000	0.000	0.000	0.000	1.000
Tsaghk-3 Dar	Tsaghkunyat	Tsaghkunyats	0.000	0.000	0.000	0.000	0.000	0.000	0.000	0.000	0.000	0.000	0.000	0.000	0.000	1.000
Tsaghk-3 Dar	Tsaghkunyat	Tsaghkunyats	0.000	0.000	0.000	0.000	0.000	0.000	0.000	0.000	0.000	0.000	0.000	0.000	0.000	1.000
Tsaghk-3 Dar	Tsaghkunyat	Tsaghkunyats	0.000	0.000	0.000	0.000	0.000	0.000	0.000	0.000	0.000	0.000	0.000	0.000	0.000	1.000
Tsaghk-3 Dar	Tsaghkunyat	Tsaghkunyats	0.000	0.000	0.000	0.000	0.000	0.000	0.000	0.000	0.000	0.000	0.000	0.000	0.000	1.000
Tsaghk-3 Dar	Tsaghkunyat	Tsaghkunyats	0.000	0.000	0.000	0.000	0.000	0.000	0.000	0.000	0.000	0.000	0.000	0.000	0.000	1.000

The results corresponding to the validation set are displayed in the second part of the table

Confusion matrix for the cross-validation results:

from \ to	Aghvorik	Arteni	Chikiani	Gegham 1	Gegham 2	Gutansar	Hatis	Kars-Arpaçay	Kelbadjar	Khorapor	Meydan Dağ	Sarıkamış	Syunik	Tsaghkunyats	Total	% correct
Aghvorik	19	0	0	0	0	0	0	0	0	0	0	0	0	0	19	100.00%
Arteni	0	19	0	0	0	0	0	0	0	0	0	0	0	0	19	100.00%
Chikiani	0	0	19	0	0	0	0	0	0	0	0	0	0	0	19	100.00%
Gegham 1	0	0	0	19	0	0	0	0	0	0	0	0	0	0	19	100.00%
Gegham 2	0	0	0	0	19	0	0	0	0	0	0	0	0	0	19	100.00%
Gutansar	0	0	0	0	0	19	0	0	0	0	0	0	0	0	19	100.00%
Hatis	0	0	0	0	0	0	19	0	0	0	0	0	0	0	19	100.00%
Kars-Arpaçay	0	0	0	0	0	0	0	19	0	0	0	0	0	0	19	100.00%
Kelbadjar	0	0	0	0	0	0	0	0	19	0	0	0	0	0	19	100.00%
Khorapor	0	0	0	0	0	0	0	0	0	19	0	0	0	0	19	100.00%
Meydan Dağ	0	0	0	0	0	0	0	0	0	0	19	0	0	0	19	100.00%
Sarıkamış	0	0	0	0	0	0	0	0	0	0	0	19	0	0	19	100.00%
Syunik	0	0	0	0	0	0	0	0	0	0	0	0	19	0	19	100.00%
Tsaghkunyat	0	0	0	0	0	0	0	0	0	0	0	0	0	19	19	100.00%
Total	19	19	19	19	19	19	19	19	19	19	19	19	19	19	260	100.00%

Results for the prediction sample:

	Predicted class	Pr(Aghvorik)	Pr(Arteni)	Pr(Chikiani)	Pr(Gegham 1)	Pr(Gegham 2)	Pr(Gutansar)	Pr(Hatis)	Pr(Kars-Arpaçay)	Pr(Kelbadjar)	Pr(Khorapor)	Pr(Meydan Dağ)	Pr(Sarıkamış)	Pr(Syunik)	Pr(Tsaghkunyats)	F1	F2	F3	F4	F5
Point Tr-2 H-	Gegham 1	0.000	0.000	0.000	1.000	0.000	0.000	0.000	0.000	0.000	0.000	0.000	0.000	0.000	0.000	1.314	-8.892	1.129	-3.508	2.857
Point Tr-2 H-	Gegham 1	0.000	0.000	0.000	1.000	0.000	0.000	0.000	0.000	0.000	0.000	0.000	0.000	0.000	0.000	0.540	-9.929	2.388	-3.796	1.484
Pit 2 Hor1 S.1	Gegham 1	0.000	0.000	0.000	1.000	0.000	0.000	0.000	0.000	0.000	0.000	0.000	0.000	0.000	0.000	1.242	-10.668	2.675	-4.120	3.469
Pit 2 Hor1 S.1	Gegham 1	0.000	0.000	0.000	1.000	0.000	0.000	0.000	0.000	0.000	0.000	0.000	0.000	0.000	0.000	-1.094	-11.375	1.929	-4.395	3.827
Pit 2 Hor1 S.1	Kelbadjar	0.000	0.000	0.000	0.000	0.000	0.000	0.000	0.000	1.000	0.000	0.000	0.000	0.000	0.000	-8.968	-8.451	0.665	2.106	-1.041
Pit 2 Hor1 S.1	Kelbadjar	0.000	0.000	0.000	0.000	0.000	0.000	0.000	0.000	1.000	0.000	0.000	0.000	0.000	0.000	-9.889	-9.883	1.128	4.345	-1.626
Pit 2 Hor1 S.1	Kelbadjar	0.000	0.000	0.000	0.000	0.000	0.000	0.000	0.000	1.000	0.000	0.000	0.000	0.000	0.000	-9.606	-9.674	0.556	3.873	-1.530
Tr2 Hor1 Sp1	Gegham 1	0.000	0.000	0.000	1.000	0.000	0.000	0.000	0.000	0.000	0.000	0.000	0.000	0.000	0.000	-1.526	-11.627	2.917	-4.620	3.475
Tr2 Hor1 Sp1	Gegham 1	0.000	0.000	0.000	1.000	0.000	0.000	0.000	0.000	0.000	0.000	0.000	0.000	0.000	0.000	-1.170	-11.300	3.062	-4.858	3.504
Tr2 Hor1 Sp1	Gutansar	0.000	0.000	0.000	0.000	0.000	1.000	0.000	0.000	0.000	0.000	0.000	0.000	0.000	0.000	4.575	1.149	12.837	14.338	-7.628
Tr2 Hor1 Sp1	Gutansar	0.000	0.000	0.000	0.000	0.000	1.000	0.000	0.000	0.000	0.000	0.000	0.000	0.000	0.000	5.246	0.106	11.706	15.743	-7.537
Tr2 Hor1 Sp1	Gegham 1	0.000	0.000	0.000	1.000	0.000	0.000	0.000	0.000	0.000	0.000	0.000	0.000	0.000	0.000	-0.503	-10.651	3.137	-4.229	3.405
Tr2 Hor1 Sp1	Gegham 1	0.000	0.000	0.000	1.000	0.000	0.000	0.000	0.000	0.000	0.000	0.000	0.000	0.000	0.000	0.511	-10.850	2.711	-4.761	4.701
Tr2 Hor1 Sp1	Gegham 1	0.000	0.000	0.000	1.000	0.000	0.000	0.000	0.000	0.000	0.000	0.000	0.000	0.000	0.000	-0.999	-11.932	3.095	-4.411	4.238
Hor1 Tr1 Sp2	Gegham 1	0.000	0.000	0.000	1.000	0.000	0.000	0.000	0.000	0.000	0.000	0.000	0.000	0.000	0.000	-0.549	-11.017	3.248	-4.840	2.887
Hor1 Tr1 Sp2	Gegham 1	0.000	0.000	0.000	1.000	0.000	0.000	0.000	0.000	0.000	0.000	0.000	0.000	0.000	0.000	-2.270	-12.004	2.017	-4.227	3.085
Hor1 Tr1 Sp2	Gegham 1	0.000	0.000	0.000	1.000	0.000	0.000	0.000	0.000	0.000	0.000	0.000	0.000	0.000	0.000	-0.844	-11.473	3.001	-4.659	3.793
Hor1 Tr1 Sp2	Gegham 1	0.000	0.000	0.000	1.000	0.000	0.000	0.000	0.000	0.000	0.000	0.000	0.000	0.000	0.000	-1.314	-11.798	2.996	-4.284	2.725
Tr2 Hor5 sou	Arteni	0.000	1.000	0.000	0.000	0.000	0.000	0.000	0.000	0.000	0.000	0.000	0.000	0.000	0.000	-2.307	0.496	4.155	-3.228	-6.589
Tr2 Hor5 sou	Arteni	0.000	1.000	0.000	0.000	0.000	0.000	0.000	0.000	0.000	0.000	0.000	0.000	0.000	0.000	-2.875	1.014	4.580	-3.201	-6.665
Tr2 Hor5 sou	Gegham 1	0.000	0.000	0.000	1.000	0.000	0.000	0.000	0.000	0.000	0.000	0.000	0.000	0.000	0.000	-1.637	-11.131	2.933	-4.200	2.356
Tr2 Hor5 sou	Gegham 1	0.000	0.000	0.000	1.000	0.000	0.000	0.000	0.000	0.000	0.000	0.000	0.000	0.000	0.000	-1.457	-10.636	3.254	-4.475	3.378
Pit1 TopSoil	Gegham 1	0.000	0.000	0.000	1.000	0.000	0.000	0.000	0.000	0.000	0.000	0.000	0.000	0.000	0.000	-0.628	-11.473	2.575	-5.011	3.997
Pit1 TopSoil	Gegham 1	0.000	0.000	0.000	1.000	0.000	0.000	0.000	0.000	0.000	0.000	0.000	0.000	0.000	0.000	-1.584	-12.292	2.196	-4.400	3.283
Pit1 TopSoil	Gegham 1	0.000	0.000	0.000	1.000	0.000	0.000	0.000	0.000	0.000	0.000	0.000	0.000	0.000	0.000	-1.597	-12.234	2.552	-4.380	4.129
Pit1 TopSoil	Gegham 1	0.000	0.000	0.000	1.000	0.000	0.000	0.000	0.000	0.000	0.000	0.000	0.000	0.000	0.000	-1.507	-11.198	3.339	-3.875	2.124
Trench2 TopS	Kelbadjar	0.000	0.000	0.000	0.000	0.000	0.000	0.000	0.000	1.000	0.000	0.000	0.000	0.000	0.000	-9.393	-10.417	0.506	3.648	-1.122
Trench2 TopS	Kelbadjar	0.000	0.000	0.000	0.000	0.000	0.000	0.000	0.000	1.000	0.000	0.000	0.000	0.000	0.000	-8.973	-9.543	1.000	2.992	-1.453
Trench2 Hor4	Gegham 1	0.000	0.000	0.000	1.000	0.000	0.000	0.000	0.000	0.000	0.000	0.000	0.000	0.000	0.000	-0.728	-11.308	3.643	-4.516	3.212
Trench2 Hor4	Gegham 1	0.000	0.000	0.000	1.000	0.000	0.000	0.000	0.000	0.000	0.000	0.000	0.000	0.000	0.000	-1.477	-10.428	3.441	-4.399	3.040
Trench1 Hor1	Gegham 1	0.000	0.000	0.000	1.000	0.000	0.000	0.000	0.000	0.000	0.000	0.000	0.000	0.000	0.000	-0.343	-10.656	3.830	-4.741	2.898
Trench1 Hor1	Gegham 1	0.000	0.000	0.000	1.000	0.000	0.000	0.000	0.000	0.000	0.000	0.000	0.000	0.000	0.000	-0.679	-11.099	3.691	-4.322	2.028
Pit1 TopSoil	Gegham 1	0.000	0.000	0.000	1.000	0.000	0.000	0.000	0.000	0.000	0.000	0.000	0.000	0.000	0.000	-1.289	-11.447	2.327	-4.347	3.434
Pit1 TopSoil	Gegham 1	0.000	0.000	0.000	1.000	0.000	0.000	0.000	0.000	0.000	0.000	0.000	0.000	0.000	0.000	-0.281	-11.861	2.984	-4.966	3.688
Pit2 Hor1 S.1	Kelbadjar	0.000	0.000	0.000	0.000	0.000	0.000	0.000	0.000	1.000	0.000	0.000	0.000	0.000	0.000	-9.228	-9.306	0.810	3.667	-1.498
Pit2 Hor1 S.1	Kelbadjar	0.000	0.000	0.000	0.000	0.000	0.000	0.000	0.000	1.000	0.000	0.000	0.000	0.000	0.000	-9.685	-9.759	0.729	4.163	-1.864
Pit 2 Hor1 S.1	Arteni	0.000	1.000	0.000	0.000	0.000	0.000	0.000	0.000	0.000	0.000	0.000	0.000	0.000	0.000	-5.369	1.716	5.098	-3.693	-7.647
Pit 2 Hor1 S.1	Arteni	0.000	1.000	0.000	0.000	0.000	0.000	0.000	0.000	0.000	0.000	0.000	0.000	0.000	0.000	-4.943	1.043	5.538	-3.595	-7.543

Trench1 Hor1	Syunik	0.000	0.000	0.000	0.000	0.000	0.000	0.000	0.000	0.052	0.000	0.000	0.000	0.948	0.000	-6.222	-11.438	-1.131	2.677	-1.388
Trench1 Hor1	Syunik	0.000	0.000	0.000	0.000	0.000	0.000	0.000	0.000	0.000	0.000	0.000	0.000	1.000	0.000	-6.635	-12.372	-1.828	2.473	-0.994
Tr-1 Hor-2 Sp	Gegham 1	0.000	0.000	0.000	1.000	0.000	0.000	0.000	0.000	0.000	0.000	0.000	0.000	0.000	0.000	-1.301	-10.790	3.557	-4.304	2.461
Tr-1 Hor-2 Sp	Gegham 1	0.000	0.000	0.000	1.000	0.000	0.000	0.000	0.000	0.000	0.000	0.000	0.000	0.000	0.000	-1.648	-10.407	3.145	-4.297	2.775
Tr-2 Hor-4 Sp	Gegham 1	0.000	0.000	0.000	1.000	0.000	0.000	0.000	0.000	0.000	0.000	0.000	0.000	0.000	0.000	-0.691	-11.463	3.229	-4.306	4.407
Tr-2 Hor-4 Sp	Gegham 1	0.000	0.000	0.000	1.000	0.000	0.000	0.000	0.000	0.000	0.000	0.000	0.000	0.000	0.000	-1.146	-10.529	3.307	-4.723	5.692
Tr-2 Hor-4 Sp	Gegham 1	0.000	0.000	0.000	1.000	0.000	0.000	0.000	0.000	0.000	0.000	0.000	0.000	0.000	0.000	0.296	-11.078	3.138	-5.017	5.868
smalls Trenc	Kelbadjar	0.000	0.000	0.000	0.000	0.000	0.000	0.000	0.000	1.000	0.000	0.000	0.000	0.000	0.000	-8.790	-9.926	2.219	3.111	-1.813
smalls Trenc	Kelbadjar	0.000	0.000	0.000	0.000	0.000	0.000	0.000	0.000	1.000	0.000	0.000	0.000	0.000	0.000	-8.818	-9.172	1.855	2.403	-1.473
smalls Trenc	Kelbadjar	0.000	0.000	0.000	0.000	0.000	0.000	0.000	0.000	1.000	0.000	0.000	0.000	0.000	0.000	-9.921	-9.863	0.822	3.934	-1.557
smalls Trenc	Gegham 1	0.000	0.000	0.000	1.000	0.000	0.000	0.000	0.000	0.000	0.000	0.000	0.000	0.000	0.000	-1.415	-12.685	2.025	-4.675	3.187
smalls Trenc	Gegham 1	0.000	0.000	0.000	1.000	0.000	0.000	0.000	0.000	0.000	0.000	0.000	0.000	0.000	0.000	-1.310	-11.487	2.010	-4.337	4.090
smalls Trenc	Gegham 1	0.000	0.000	0.000	1.000	0.000	0.000	0.000	0.000	0.000	0.000	0.000	0.000	0.000	0.000	-0.677	-10.150	3.734	-4.473	3.608
smalls Trenc	Gegham 1	0.000	0.000	0.000	1.000	0.000	0.000	0.000	0.000	0.000	0.000	0.000	0.000	0.000	0.000	-1.531	-11.293	2.708	-4.340	3.748
smalls Trenc	Gegham 1	0.000	0.000	0.000	1.000	0.000	0.000	0.000	0.000	0.000	0.000	0.000	0.000	0.000	0.000	-1.668	-12.219	3.317	-3.900	1.935
smalls Trenc	Syunik	0.000	0.000	0.000	0.000	0.000	0.000	0.000	0.000	0.000	0.000	0.000	0.000	1.000	0.000	-6.427	-11.408	-1.803	3.982	-1.515
smalls Trenc	Kelbadjar	0.000	0.000	0.000	0.000	0.000	0.000	0.000	0.000	1.000	0.000	0.000	0.000	0.000	0.000	-10.157	-8.584	1.684	5.271	-2.138
smalls Trenc	Kelbadjar	0.000	0.000	0.000	0.000	0.000	0.000	0.000	0.000	1.000	0.000	0.000	0.000	0.000	0.000	-8.647	-10.537	-0.903	3.232	-0.969
smalls Trenc	Gegham 1	0.000	0.000	0.000	1.000	0.000	0.000	0.000	0.000	0.000	0.000	0.000	0.000	0.000	0.000	-1.016	-10.753	2.200	-3.985	3.459
smalls Trenc	Gegham 1	0.000	0.000	0.000	1.000	0.000	0.000	0.000	0.000	0.000	0.000	0.000	0.000	0.000	0.000	-0.900	-11.996	3.242	-4.522	3.219
smalls Trenc	Kelbadjar	0.000	0.000	0.000	0.000	0.000	0.000	0.000	0.000	1.000	0.000	0.000	0.000	0.000	0.000	-9.597	-9.155	-0.095	3.630	-1.318
smalls Trenc	Kelbadjar	0.000	0.000	0.000	0.000	0.000	0.000	0.000	0.000	1.000	0.000	0.000	0.000	0.000	0.000	-8.366	-7.946	-0.133	4.498	-1.518
smalls Trenc	Kelbadjar	0.000	0.000	0.000	0.000	0.000	0.000	0.000	0.000	1.000	0.000	0.000	0.000	0.000	0.000	-9.505	-7.896	-0.794	4.830	-2.065
smalls Trenc	Gegham 1	0.000	0.000	0.000	1.000	0.000	0.000	0.000	0.000	0.000	0.000	0.000	0.000	0.000	0.000	-1.413	-10.293	3.346	-4.492	4.087
smalls Trenc	Arteni	0.000	0.993	0.000	0.000	0.007	0.000	0.000	0.000	0.000	0.000	0.000	0.000	0.000	0.000	-0.043	2.710	3.904	-3.057	-5.648
smalls Trenc	Kelbadjar	0.000	0.000	0.000	0.000	0.000	0.000	0.000	0.000	1.000	0.000	0.000	0.000	0.000	0.000	-10.876	-9.783	0.279	5.889	-2.267
smalls Trenc	Gegham 1	0.000	0.000	0.000	1.000	0.000	0.000	0.000	0.000	0.000	0.000	0.000	0.000	0.000	0.000	-0.841	-10.689	3.014	-4.456	4.437
smalls Trenc	Gegham 1	0.000	0.000	0.000	1.000	0.000	0.000	0.000	0.000	0.000	0.000	0.000	0.000	0.000	0.000	-1.025	-10.697	2.859	-4.733	4.578
smalls Trenc	Syunik	0.000	0.000	0.000	0.000	0.000	0.000	0.000	0.000	0.000	0.000	0.000	0.000	1.000	0.000	-6.640	-11.927	-2.297	3.134	-1.233
smalls Trenc	Kelbadjar	0.000	0.000	0.000	0.000	0.000	0.000	0.000	0.000	1.000	0.000	0.000	0.000	0.000	0.000	-9.790	-10.659	-0.250	4.494	-1.569
smalls Trenc	Kelbadjar	0.000	0.000	0.000	0.000	0.000	0.000	0.000	0.000	0.988	0.000	0.000	0.000	0.012	0.000	-10.900	-9.438	-1.733	6.462	-2.331
smalls Trenc	Gegham 1	0.000	0.000	0.000	1.000	0.000	0.000	0.000	0.000	0.000	0.000	0.000	0.000	0.000	0.000	-1.595	-11.285	2.423	-4.047	2.984
smalls Trenc	Kelbadjar	0.000	0.000	0.000	0.000	0.000	0.000	0.000	0.000	0.986	0.000	0.000	0.000	0.014	0.000	-11.217	-10.674	-0.504	5.273	-1.446
smalls Trenc	Gegham 1	0.000	0.000	0.000	1.000	0.000	0.000	0.000	0.000	0.000	0.000	0.000	0.000	0.000	0.000	-1.160	-10.199	2.956	-4.388	5.125
smalls Trenc	Gegham 1	0.000	0.000	0.000	1.000	0.000	0.000	0.000	0.000	0.000	0.000	0.000	0.000	0.000	0.000	-0.280	-11.060	3.495	-4.818	7.976
smalls Trenc	Kelbadjar	0.000	0.000	0.000	0.000	0.000	0.000	0.000	0.000	1.000	0.000	0.000	0.000	0.000	0.000	-9.919	-9.595	0.288	4.718	-1.538
smalls Pit2 H	Gegham 1	0.000	0.000	0.000	1.000	0.000	0.000	0.000	0.000	0.000	0.000	0.000	0.000	0.000	0.000	-0.742	-12.099	3.713	-4.797	3.015
smalls Pit2 H	Syunik	0.000	0.000	0.000	0.000	0.000	0.000	0.000	0.000	0.000	0.000	0.000	0.000	1.000	0.000	-6.506	-12.892	-1.065	4.036	-1.217
smalls Pit2 H	Gegham 1	0.000	0.000	0.000	1.000	0.000	0.000	0.000	0.000	0.000	0.000	0.000	0.000	0.000	0.000	-0.369	-11.898	2.339	-4.799	4.532
smalls Pit2 H	Gegham 1	0.000	0.000	0.000	1.000	0.000	0.000	0.000	0.000	0.000	0.000	0.000	0.000	0.000	0.000	-1.338	-11.917	3.064	-4.473	2.715
smalls Pit2 H	Kelbadjar	0.000	0.000	0.000	0.000	0.000	0.000	0.000	0.000	1.000	0.000	0.000	0.000	0.000	0.000	-8.960	-8.578	0.678	2.634	-1.365
smalls Pit2 H	Syunik	0.000	0.000	0.000	0.000	0.000	0.000	0.000	0.000	0.027	0.000	0.000	0.000	0.973	0.000	-6.656	-12.359	-0.482	3.410	-1.230
smalls Pit2 H	Gegham 1	0.000	0.000	0.000	1.000	0.000	0.000	0.000	0.000	0.000	0.000	0.000	0.000	0.000	0.000	-3.107	-9.427	2.393	-2.116	-0.383
smalls Pit2 H	Gegham 1	0.000	0.000	0.000	1.000	0.000	0.000	0.000	0.000	0.000	0.000	0.000	0.000	0.000	0.000	-0.758	-11.660	4.012	-4.524	3.446
smalls Pit2 H	Gegham 1	0.000	0.000	0.000	1.000	0.000	0.000	0.000	0.000	0.000	0.000	0.000	0.000	0.000	0.000	-1.610	-13.016	2.310	-4.179	3.242
smalls Pit2 H	Syunik	0.000	0.000	0.000	0.000	0.000	0.000	0.000	0.000	0.000	0.000	0.000	0.000	1.000	0.000	-6.758	-13.042	-1.575	3.225	-1.227
smalls Pit2 H	Gegham 1	0.000	0.000	0.000	1.000	0.000	0.000	0.000	0.000	0.000	0.000	0.000	0.000	0.000	0.000	-1.879	-12.326	1.660	-3.954	2.418
smalls Pit2 H	Gegham 1	0.000	0.000	0.000	1.000	0.000	0.000	0.000	0.000	0.000	0.000	0.000	0.000	0.000	0.000	-2.464	-11.723	2.901	-4.446	3.536
smalls Pit2 H	Gegham 1	0.000	0.000	0.000	1.000	0.000	0.000	0.000	0.000	0.000	0.000	0.000	0.000	0.000	0.000	-1.821	-11.505	2.678	-3.840	3.059
smalls Pit2 H	Gegham 1	0.000	0.000	0.000	1.000	0.000	0.000	0.000	0.000	0.000	0.000	0.000	0.000	0.000	0.000	-0.264	-11.887	3.783	-4.679	3.774
smalls Pit2 H	Gegham 1	0.000	0.000	0.000	1.000	0.000	0.000	0.000	0.000	0.000	0.000	0.000	0.000	0.000	0.000	-1.096	-11.500	3.292	-4.810	3.754
smalls Pit2 H	Gegham 1	0.000	0.000	0.000	1.000	0.000	0.000	0.000	0.000	0.000	0.000	0.000	0.000	0.000	0.000	-0.250	-11.007	2.748	-4.091	2.753
smalls Pit2 H	Syunik	0.000	0.000	0.000	0.000	0.000	0.000	0.000	0.000	0.000	0.000	0.000	0.000	1.000	0.000	-7.682	-17.696	-4.215	2.455	0.585
smalls Pit2 H	Gegham 1	0.000	0.000	0.000	1.000	0.000	0.000	0.000	0.000	0.000	0.000	0.000	0.000	0.000	0.000	-1.246	-10.896	2.637	-4.475	3.524
smalls Pit2 H	Gegham 1	0.000	0.000	0.000	1.000	0.000	0.000	0.000	0.000	0.000	0.000	0.000	0.000	0.000	0.000	-1.140	-10.947	3.413	-4.478	2.709
smalls Pit2 H	Gutansar	0.000	0.000	0.000	0.000	0.000	1.000	0.000	0.000	0.000	0.000	0.000	0.000	0.000	0.000	5.861	-0.428	12.746	16.380	-8.303
smalls Pit2 H	Khorapor	0.000	0.000	0.000	0.000	0.000	0.000	0.000	0.000	0.000	1.000	0.000	0.000	0.000	0.000	-0.775	-18.120	-0.535	-5.395	14.111
smalls Pit2 H	Gegham 1	0.000	0.000	0.000	1.000	0.000	0.000	0.000	0.000	0.000	0.000	0.000	0.000	0.000	0.000	-0.584	-11.272	3.117	-4.861	5.671
smalls Pit2 H	Gegham 1	0.000	0.000	0.000	1.000	0.000	0.000	0.000	0.000	0.000	0.000	0.000	0.000	0.000	0.000	0.062	-11.113	2.595	-4.682	5.809
smalls Pit2 H	Gegham 1	0.000	0.000	0.000	1.000	0.000	0.000	0.000	0.000	0.000	0.000	0.000	0.000	0.000	0.000	-1.265	-11.585	3.078	-4.491	3.977
smalls Pit2 H	Kelbadjar	0.000	0.000	0.000	0.000	0.000	0.000	0.000	0.000	1.000	0.000	0.000	0.000	0.000	0.000	-9.503	-10.130	1.235	3.190	-0.778
smalls Pit2 H	Gegham 1	0.000	0.000	0.000	1.000	0.000	0.000	0.000	0.000	0.000	0.000	0.000	0.000	0.000	0.000	-0.262	-10.258	2.955	-4.883	3.957
smalls Pit2 H	Syunik	0.000	0.000	0.000	0.000	0.000	0.000	0.000	0.000	0.000	0.000	0.000	0.000	1.000	0.000	-6.350	-13.996	-1.880	2.655	-0.081
smalls Pit2 H	Syunik	0.000	0.000	0.000	0.000	0.000	0.000	0.000	0.000	0.000	0.000	0.000	0.000	1.000	0.000	-4.842	-11.444	-3.090	4.475	0.221
smalls Pit2 H	Syunik	0.000	0.000	0.000	0.000	0.000	0.000	0.000	0.000	0.000	0.000	0.000	0.000	1.000	0.000	-7.168	-14.034	-1.533	4.582	-1.268
smalls Pit2 H	Gegham 1	0.000	0.000	0.000	1.000	0.000	0.000	0.000	0.000	0.000	0.000	0.000	0.000	0.000	0.000	-0.234	-11.579	3.211	-4.804	3.403
smalls Pit2 H	Gegham 1	0.000	0.000	0.000	1.000	0.000	0.000	0.000	0.000	0.000	0.000	0.000	0.000	0.000	0.000	-4.044	-10.692	4.434	-4.759	8.515

smalls bag3	Gegham 1	0.000	0.000	0.000	1.000	0.000	0.000	0.000	0.000	0.000	0.000	0.000	0.000	0.000	0.000	-0.545	-11.172	3.686	-4.738	4.569
smalls bag3	Gegham 1	0.000	0.000	0.000	1.000	0.000	0.000	0.000	0.000	0.000	0.000	0.000	0.000	0.000	0.000	-0.395	-9.269	4.011	-4.889	4.656
smalls bag3	Gegham 1	0.000	0.000	0.000	1.000	0.000	0.000	0.000	0.000	0.000	0.000	0.000	0.000	0.000	0.000	-0.596	-10.966	2.925	-4.574	5.388
smalls bag4	Kelbadjar	0.000	0.000	0.000	0.000	0.000	0.000	0.000	0.000	1.000	0.000	0.000	0.000	0.000	0.000	-10.190	-10.350	0.418	4.529	-1.552
smalls bag4	Kelbadjar	0.000	0.000	0.000	0.000	0.000	0.000	0.000	0.000	1.000	0.000	0.000	0.000	0.000	0.000	-8.922	-9.536	1.435	3.123	-1.098
smalls bag4	Kelbadjar	0.000	0.000	0.000	0.000	0.000	0.000	0.000	0.000	1.000	0.000	0.000	0.000	0.000	0.000	-8.891	-9.665	0.897	3.708	-1.711
smalls bag4	Gegham 1	0.000	0.000	0.000	1.000	0.000	0.000	0.000	0.000	0.000	0.000	0.000	0.000	0.000	0.000	-1.357	-11.155	3.069	-3.995	2.647
smalls bag4	Gegham 1	0.000	0.000	0.000	1.000	0.000	0.000	0.000	0.000	0.000	0.000	0.000	0.000	0.000	0.000	-0.649	-11.175	3.180	-4.563	3.433
smalls bag4	Gegham 1	0.000	0.000	0.000	1.000	0.000	0.000	0.000	0.000	0.000	0.000	0.000	0.000	0.000	0.000	-1.142	-10.094	2.915	-3.832	1.057
smalls bag4	Kelbadjar	0.000	0.000	0.000	0.000	0.000	0.000	0.000	0.000	1.000	0.000	0.000	0.000	0.000	0.000	-9.710	-9.692	1.932	4.165	-1.628
smalls bag4	Gegham 1	0.000	0.000	0.000	1.000	0.000	0.000	0.000	0.000	0.000	0.000	0.000	0.000	0.000	0.000	-0.948	-11.263	3.118	-4.419	3.287
smalls bag4	Gegham 1	0.000	0.000	0.000	1.000	0.000	0.000	0.000	0.000	0.000	0.000	0.000	0.000	0.000	0.000	-0.686	-10.745	3.224	-4.486	2.455
smalls bag4	Gegham 1	0.000	0.000	0.000	1.000	0.000	0.000	0.000	0.000	0.000	0.000	0.000	0.000	0.000	0.000	-1.249	-11.780	2.465	-4.353	3.156
smalls bag4	Kelbadjar	0.000	0.000	0.000	0.000	0.000	0.000	0.000	0.000	1.000	0.000	0.000	0.000	0.000	0.000	-9.094	-8.873	1.537	3.247	-1.576
smalls bag4	Gegham 1	0.000	0.000	0.000	1.000	0.000	0.000	0.000	0.000	0.000	0.000	0.000	0.000	0.000	0.000	-0.866	-10.704	2.926	-4.764	4.781
smalls bag4	Kelbadjar	0.000	0.000	0.000	0.000	0.000	0.000	0.000	0.000	1.000	0.000	0.000	0.000	0.000	0.000	-9.353	-10.181	-0.786	3.889	-0.959
smalls bag4	Gegham 1	0.000	0.000	0.000	1.000	0.000	0.000	0.000	0.000	0.000	0.000	0.000	0.000	0.000	0.000	0.754	-9.317	3.068	-3.990	3.474
smalls bag4	Gegham 1	0.000	0.000	0.000	1.000	0.000	0.000	0.000	0.000	0.000	0.000	0.000	0.000	0.000	0.000	-2.118	-11.281	2.341	-4.219	3.927
smalls bag4	Gegham 1	0.000	0.000	0.000	1.000	0.000	0.000	0.000	0.000	0.000	0.000	0.000	0.000	0.000	0.000	-0.051	-9.335	3.457	-4.373	2.409
smalls bag4	Kelbadjar	0.000	0.000	0.000	0.000	0.000	0.000	0.000	0.000	1.000	0.000	0.000	0.000	0.000	0.000	-9.251	-11.047	0.020	4.053	-0.879
smalls bag4	Gegham 1	0.000	0.000	0.000	1.000	0.000	0.000	0.000	0.000	0.000	0.000	0.000	0.000	0.000	0.000	-0.652	-10.958	3.242	-4.463	3.781
smalls bag4	Gegham 1	0.000	0.000	0.000	1.000	0.000	0.000	0.000	0.000	0.000	0.000	0.000	0.000	0.000	0.000	-0.816	-8.793	2.877	-3.407	3.096
smalls bag4	Gegham 1	0.000	0.000	0.000	1.000	0.000	0.000	0.000	0.000	0.000	0.000	0.000	0.000	0.000	0.000	-0.168	-9.264	3.462	-4.487	3.305
smalls bag4	Kelbadjar	0.000	0.000	0.000	0.000	0.000	0.000	0.000	0.000	1.000	0.000	0.000	0.000	0.000	0.000	-9.815	-9.438	1.480	5.121	-1.991
smalls bag4	Gegham 1	0.000	0.000	0.000	1.000	0.000	0.000	0.000	0.000	0.000	0.000	0.000	0.000	0.000	0.000	-1.695	-11.730	3.149	-4.225	3.324
smalls bag4	Gegham 1	0.000	0.000	0.000	1.000	0.000	0.000	0.000	0.000	0.000	0.000	0.000	0.000	0.000	0.000	-1.189	-11.582	3.182	-3.685	1.638
smalls bag4	Kelbadjar	0.000	0.000	0.000	0.000	0.000	0.000	0.000	0.000	1.000	0.000	0.000	0.000	0.000	0.000	-9.662	-9.851	-0.877	3.900	-1.358
smalls bag4	Gegham 1	0.000	0.000	0.000	1.000	0.000	0.000	0.000	0.000	0.000	0.000	0.000	0.000	0.000	0.000	-0.568	-10.050	2.757	-4.953	5.718
smalls bag4	Gegham 1	0.000	0.000	0.000	1.000	0.000	0.000	0.000	0.000	0.000	0.000	0.000	0.000	0.000	0.000	0.211	-10.513	2.711	-4.330	4.629
smalls bag4	Gegham 1	0.000	0.000	0.000	1.000	0.000	0.000	0.000	0.000	0.000	0.000	0.000	0.000	0.000	0.000	-1.014	-12.432	2.547	-4.220	2.924
smalls bag4	Gegham 1	0.000	0.000	0.000	1.000	0.000	0.000	0.000	0.000	0.000	0.000	0.000	0.000	0.000	0.000	-2.032	-11.748	3.107	-4.388	3.980
smalls bag4	Gegham 1	0.000	0.000	0.000	1.000	0.000	0.000	0.000	0.000	0.000	0.000	0.000	0.000	0.000	0.000	-0.669	-11.628	3.394	-5.154	6.546
smalls bag4	Gegham 1	0.000	0.000	0.000	1.000	0.000	0.000	0.000	0.000	0.000	0.000	0.000	0.000	0.000	0.000	-0.882	-11.334	3.024	-4.303	2.821
smalls bag4	Gegham 1	0.000	0.000	0.000	1.000	0.000	0.000	0.000	0.000	0.000	0.000	0.000	0.000	0.000	0.000	0.337	-11.235	3.941	-5.157	5.142
smalls bag4	Gegham 1	0.000	0.000	0.000	1.000	0.000	0.000	0.000	0.000	0.000	0.000	0.000	0.000	0.000	0.000	-1.427	-11.568	3.041	-4.431	5.616
smalls bag4	Gegham 1	0.000	0.000	0.000	1.000	0.000	0.000	0.000	0.000	0.000	0.000	0.000	0.000	0.000	0.000	-0.330	-9.489	3.175	-4.529	5.081
smalls bag4	Gegham 1	0.000	0.000	0.000	1.000	0.000	0.000	0.000	0.000	0.000	0.000	0.000	0.000	0.000	0.000	-1.056	-11.096	3.209	-4.193	5.558
smalls bag4	Gegham 1	0.000	0.000	0.000	1.000	0.000	0.000	0.000	0.000	0.000	0.000	0.000	0.000	0.000	0.000	-1.495	-11.709	3.985	-5.234	6.474
smalls bag4	Gegham 1	0.000	0.000	0.000	1.000	0.000	0.000	0.000	0.000	0.000	0.000	0.000	0.000	0.000	0.000	0.074	-9.979	3.845	-4.846	4.457
smalls bag4	Kelbadjar	0.000	0.000	0.000	0.000	0.000	0.000	0.000	0.000	1.000	0.000	0.000	0.000	0.000	0.000	-9.525	-10.293	-0.736	6.038	-1.800
smalls bag4	Gegham 1	0.000	0.000	0.000	1.000	0.000	0.000	0.000	0.000	0.000	0.000	0.000	0.000	0.000	0.000	-0.311	-11.174	4.100	-4.798	5.459
smalls bag4	Gegham 1	0.000	0.000	0.000	1.000	0.000	0.000	0.000	0.000	0.000	0.000	0.000	0.000	0.000	0.000	0.494	-9.925	3.229	-4.891	5.488
smalls bag4	Gegham 1	0.000	0.000	0.000	1.000	0.000	0.000	0.000	0.000	0.000	0.000	0.000	0.000	0.000	0.000	-0.144	-9.664	3.516	-4.278	3.582
smalls bag5	Gegham 1	0.000	0.000	0.000	1.000	0.000	0.000	0.000	0.000	0.000	0.000	0.000	0.000	0.000	0.000	0.332	-10.040	4.160	-4.822	5.518
smalls bag5	Gegham 1	0.000	0.000	0.000	1.000	0.000	0.000	0.000	0.000	0.000	0.000	0.000	0.000	0.000	0.000	0.305	-10.694	3.827	-4.829	4.619
smalls bag5	Gegham 1	0.000	0.000	0.000	1.000	0.000	0.000	0.000	0.000	0.000	0.000	0.000	0.000	0.000	0.000	-0.583	-9.830	3.743	-4.678	3.256
smalls bag5	Gegham 1	0.000	0.000	0.000	1.000	0.000	0.000	0.000	0.000	0.000	0.000	0.000	0.000	0.000	0.000	-0.109	-10.152	3.466	-4.689	2.714
smalls bag5	Gegham 1	0.000	0.000	0.000	1.000	0.000	0.000	0.000	0.000	0.000	0.000	0.000	0.000	0.000	0.000	-0.433	-10.306	3.720	-4.799	3.714
smalls bag5	Arteni	0.000	1.000	0.000	0.000	0.000	0.000	0.000	0.000	0.000	0.000	0.000	0.000	0.000	0.000	-2.488	2.738	3.880	-2.768	-5.202
smalls bag5	Syunik	0.000	0.000	0.000	0.000	0.000	0.000	0.000	0.000	0.000	0.000	0.000	0.000	1.000	0.000	-4.967	-12.422	-3.009	3.857	-0.483
smalls bag5	Kelbadjar	0.000	0.000	0.000	0.000	0.000	0.000	0.000	0.000	0.990	0.000	0.000	0.000	0.010	0.000	-8.733	-9.823	-2.181	5.575	-1.614
smalls bag5	Kelbadjar	0.000	0.000	0.000	0.000	0.000	0.000	0.000	0.000	1.000	0.000	0.000	0.000	0.000	0.000	-10.469	-10.201	-0.357	4.522	-1.679
smalls bag5	Gegham 1	0.000	0.000	0.000	1.000	0.000	0.000	0.000	0.000	0.000	0.000	0.000	0.000	0.000	0.000	-0.832	-10.551	3.489	-4.569	3.547
smalls bag5	Gegham 1	0.000	0.000	0.000	1.000	0.000	0.000	0.000	0.000	0.000	0.000	0.000	0.000	0.000	0.000	0.402	-11.417	3.232	-4.478	3.592
smalls bag5	Syunik	0.000	0.000	0.000	0.000	0.000	0.000	0.000	0.000	0.000	0.000	0.000	0.000	1.000	0.000	-9.550	-19.306	-5.320	3.740	0.084
smalls bag5	Gegham 1	0.000	0.000	0.000	1.000	0.000	0.000	0.000	0.000	0.000	0.000	0.000	0.000	0.000	0.000	0.045	-11.960	3.204	-4.777	3.817
smalls bag5	Gegham 1	0.000	0.000	0.000	1.000	0.000	0.000	0.000	0.000	0.000	0.000	0.000	0.000	0.000	0.000	0.292	-10.228	4.310	-5.479	4.431
smalls bag5	Gegham 1	0.000	0.000	0.000	1.000	0.000	0.000	0.000	0.000	0.000	0.000	0.000	0.000	0.000	0.000	-1.032	-9.430	4.339	-5.042	7.441
smalls bag5	Gegham 1	0.000	0.000	0.000	1.000	0.000	0.000	0.000	0.000	0.000	0.000	0.000	0.000	0.000	0.000	-0.696	-11.013	3.452	-4.802	5.451
smalls bag5	Gegham 1	0.000	0.000	0.000	1.000	0.000	0.000	0.000	0.000	0.000	0.000	0.000	0.000	0.000	0.000	-1.019	-11.287	3.231	-4.261	3.131
smalls bag5	Gegham 1	0.000	0.000	0.000	1.000	0.000	0.000	0.000	0.000	0.000	0.000	0.000	0.000	0.000	0.000	-0.660	-11.432	3.173	-4.538	4.163
smalls bag5	Gegham 1	0.000	0.000	0.000	1.000	0.000	0.000	0.000	0.000	0.000	0.000	0.000	0.000	0.000	0.000	-0.987	-11.596	3.662	-4.646	4.858
smalls bag5	Kelbadjar	0.000	0.000	0.000	0.000	0.000	0.000	0.000	0.000	1.000	0.000	0.000	0.000	0.000	0.000	-9.322	-9.411	0.416	4.569	-1.771
smalls bag5	Kelbadjar	0.000	0.000	0.000	0.000	0.000	0.000	0.000	0.000	0.989	0.000	0.000	0.000	0.011	0.000	-10.123	-9.942	-1.771	3.797	-1.099
smalls bag5	Gegham 1	0.000	0.000	0.000	1.000	0.000	0.000	0.000	0.000	0.000	0.000	0.000	0.000	0.000	0.000	-0.895	-11.093	3.237	-4.367	3.141
smalls bag5	Gegham 1	0.000	0.000	0.000	1.000	0.000	0.000	0.000	0.000	0.000	0.000	0.000	0.000	0.000	0.000	-1.880	-9.100	3.954	-4.491	6.643
smalls bag5	Gegham 1	0.000	0.000	0.000	1.000	0.000	0.000	0.000	0.000	0.000	0.000	0.000	0.000	0.000	0.000	-1.163	-8.959	4.642	-5.120	6.583

smalls bag5	Gegham 1	0.000	0.000	0.000	1.000	0.000	0.000	0.000	0.000	0.000	0.000	0.000	0.000	0.000	0.000	-0.693	-10.442	3.200	-4.849	5.574
smalls bag5	Gegham 1	0.000	0.000	0.000	1.000	0.000	0.000	0.000	0.000	0.000	0.000	0.000	0.000	0.000	0.000	-1.681	-12.563	2.420	-4.659	6.867
smalls bag5	Gegham 1	0.000	0.000	0.000	1.000	0.000	0.000	0.000	0.000	0.000	0.000	0.000	0.000	0.000	0.000	-0.974	-10.751	3.199	-4.398	4.073
smalls bag5	Gegham 1	0.000	0.000	0.000	1.000	0.000	0.000	0.000	0.000	0.000	0.000	0.000	0.000	0.000	0.000	-1.082	-10.027	4.309	-5.007	4.853
smalls bag5	Gegham 1	0.000	0.000	0.000	1.000	0.000	0.000	0.000	0.000	0.000	0.000	0.000	0.000	0.000	0.000	-0.530	-11.534	2.432	-4.569	5.482
smalls bag5	Gegham 1	0.000	0.000	0.000	1.000	0.000	0.000	0.000	0.000	0.000	0.000	0.000	0.000	0.000	0.000	-0.872	-10.810	3.110	-4.233	3.725
smalls bag5	Syunik	0.000	0.000	0.000	0.000	0.000	0.000	0.000	0.000	0.058	0.000	0.000	0.000	0.942	0.000	-10.753	-9.994	-2.023	6.235	-1.462
smalls bag5	Gegham 1	0.000	0.000	0.000	1.000	0.000	0.000	0.000	0.000	0.000	0.000	0.000	0.000	0.000	0.000	-0.405	-10.752	4.019	-4.892	3.610
smalls bag5	Syunik	0.000	0.000	0.000	0.000	0.000	0.000	0.000	0.000	0.000	0.000	0.000	0.000	1.000	0.000	-11.584	-8.872	-2.117	6.523	-1.743
smalls bag5	Kelbadjar	0.000	0.000	0.000	0.000	0.000	0.000	0.000	0.000	1.000	0.000	0.000	0.000	0.000	0.000	-8.395	-8.791	-0.938	4.771	-0.684
smalls bag5	Gegham 1	0.000	0.000	0.000	1.000	0.000	0.000	0.000	0.000	0.000	0.000	0.000	0.000	0.000	0.000	-0.848	-12.588	3.058	-4.769	4.937
smalls bag5	Syunik	0.000	0.000	0.000	0.000	0.000	0.000	0.000	0.000	0.000	0.000	0.000	0.000	1.000	0.000	-6.602	-13.688	-3.386	4.453	-0.757
smalls bag5	Gegham 1	0.000	0.000	0.000	1.000	0.000	0.000	0.000	0.000	0.000	0.000	0.000	0.000	0.000	0.000	0.562	-9.222	4.090	-4.904	3.860
smalls bag5	Kelbadjar	0.000	0.000	0.000	0.000	0.000	0.000	0.000	0.000	0.937	0.000	0.000	0.000	0.063	0.000	-10.561	-8.818	-1.245	5.556	-1.496
smalls bag5	Gegham 1	0.000	0.000	0.000	1.000	0.000	0.000	0.000	0.000	0.000	0.000	0.000	0.000	0.000	0.000	-1.233	-10.718	3.734	-4.139	5.848
smalls bag5	Kelbadjar	0.000	0.000	0.000	0.000	0.000	0.000	0.000	0.000	0.998	0.000	0.000	0.000	0.002	0.000	-11.218	-9.211	-1.506	6.374	-2.148
smalls bag5	Gegham 2	0.000	0.227	0.000	0.000	0.773	0.000	0.000	0.000	0.000	0.000	0.000	0.000	0.000	0.000	-0.008	4.355	4.533	-2.275	-4.884
smalls bag5	Gegham 1	0.000	0.000	0.000	1.000	0.000	0.000	0.000	0.000	0.000	0.000	0.000	0.000	0.000	0.000	-0.892	-0.844	2.406	-3.544	5.493
smalls bag5	Gegham 1	0.000	0.000	0.000	1.000	0.000	0.000	0.000	0.000	0.000	0.000	0.000	0.000	0.000	0.000	-0.384	-8.924	5.070	-4.566	6.246
smalls bag5	Kelbadjar	0.000	0.000	0.000	0.000	0.000	0.000	0.000	0.000	1.000	0.000	0.000	0.000	0.000	0.000	-9.670	-9.197	-0.978	5.718	-1.922
smalls bag5	Gegham 1	0.000	0.000	0.000	1.000	0.000	0.000	0.000	0.000	0.000	0.000	0.000	0.000	0.000	0.000	-1.037	-9.781	2.784	-4.627	5.504
smalls bag5	Gegham 1	0.000	0.000	0.000	1.000	0.000	0.000	0.000	0.000	0.000	0.000	0.000	0.000	0.000	0.000	0.379	-10.984	3.668	-4.561	5.440
Trench1 Hor	Gegham 1	0.000	0.000	0.000	1.000	0.000	0.000	0.000	0.000	0.000	0.000	0.000	0.000	0.000	0.000	-1.087	-10.082	1.654	-4.241	3.907
Trench1 Hor	Gegham 1	0.000	0.000	0.000	1.000	0.000	0.000	0.000	0.000	0.000	0.000	0.000	0.000	0.000	0.000	-0.265	-10.911	3.437	-4.708	3.491
Trench1 Hor	Gegham 1	0.000	0.000	0.000	1.000	0.000	0.000	0.000	0.000	0.000	0.000	0.000	0.000	0.000	0.000	-1.900	-10.719	4.015	-4.423	1.415
Trench1 Hor	Gegham 1	0.000	0.000	0.000	1.000	0.000	0.000	0.000	0.000	0.000	0.000	0.000	0.000	0.000	0.000	-0.935	-9.463	2.143	-4.547	3.570
Trench1 Hor	Gegham 1	0.000	0.000	0.000	1.000	0.000	0.000	0.000	0.000	0.000	0.000	0.000	0.000	0.000	0.000	-1.771	-12.499	1.724	-4.282	3.376
Trench1 Hor	Gegham 1	0.000	0.000	0.000	1.000	0.000	0.000	0.000	0.000	0.000	0.000	0.000	0.000	0.000	0.000	-1.587	-12.151	3.093	-4.562	3.856
Trench1 Hor	Gegham 1	0.000	0.000	0.000	1.000	0.000	0.000	0.000	0.000	0.000	0.000	0.000	0.000	0.000	0.000	-0.864	-11.311	3.155	-4.604	2.958
Trench1 Hor	Kelbadjar	0.000	0.000	0.000	0.000	0.000	0.000	0.000	0.000	1.000	0.000	0.000	0.000	0.000	0.000	-9.402	-10.575	1.106	4.163	-1.108
Trench1 Hor	Gegham 1	0.000	0.000	0.000	1.000	0.000	0.000	0.000	0.000	0.000	0.000	0.000	0.000	0.000	0.000	-0.912	-11.647	2.696	-4.460	3.676
Trench1 Hor	Gegham 1	0.000	0.000	0.000	1.000	0.000	0.000	0.000	0.000	0.000	0.000	0.000	0.000	0.000	0.000	0.056	-10.456	3.485	-4.386	2.943
Trench1 Hor	Gegham 1	0.000	0.000	0.000	1.000	0.000	0.000	0.000	0.000	0.000	0.000	0.000	0.000	0.000	0.000	-0.962	-12.472	2.757	-4.592	3.627
Trench1 Hor	Gegham 1	0.000	0.000	0.000	1.000	0.000	0.000	0.000	0.000	0.000	0.000	0.000	0.000	0.000	0.000	0.032	-11.465	2.570	-4.075	3.820
Trench1 Hor	Gegham 1	0.000	0.000	0.000	1.000	0.000	0.000	0.000	0.000	0.000	0.000	0.000	0.000	0.000	0.000	-0.787	-13.337	2.125	-4.325	4.143
Trench1 Hor	Gegham 1	0.000	0.000	0.000	1.000	0.000	0.000	0.000	0.000	0.000	0.000	0.000	0.000	0.000	0.000	-1.459	-11.088	3.273	-4.138	2.025
Trench1 Hor	Gegham 1	0.000	0.000	0.000	1.000	0.000	0.000	0.000	0.000	0.000	0.000	0.000	0.000	0.000	0.000	-2.004	-10.832	2.337	-4.409	4.220
Trench1 Hor	Gegham 1	0.000	0.000	0.000	1.000	0.000	0.000	0.000	0.000	0.000	0.000	0.000	0.000	0.000	0.000	0.388	-11.746	2.357	-4.024	4.036
Trench1 Hor	Gegham 1	0.000	0.000	0.000	1.000	0.000	0.000	0.000	0.000	0.000	0.000	0.000	0.000	0.000	0.000	-1.078	-8.830	-0.232	-4.173	6.955
Trench1 Hor	Gegham 1	0.000	0.000	0.000	1.000	0.000	0.000	0.000	0.000	0.000	0.000	0.000	0.000	0.000	0.000	-0.020	-10.869	3.748	-4.534	2.443
Trench1 Hor	Gegham 1	0.000	0.000	0.000	1.000	0.000	0.000	0.000	0.000	0.000	0.000	0.000	0.000	0.000	0.000	-0.473	-11.977	2.947	-4.779	3.932
Trench1 Hor	Gegham 1	0.000	0.000	0.000	1.000	0.000	0.000	0.000	0.000	0.000	0.000	0.000	0.000	0.000	0.000	-0.414	-11.479	3.743	-4.648	3.743
Trench1 Hor	Gegham 1	0.000	0.000	0.000	1.000	0.000	0.000	0.000	0.000	0.000	0.000	0.000	0.000	0.000	0.000	1.892	-10.822	1.603	-3.999	3.936
Trench1 Hor	Gegham 1	0.000	0.000	0.000	1.000	0.000	0.000	0.000	0.000	0.000	0.000	0.000	0.000	0.000	0.000	-0.775	-12.109	2.372	-4.479	3.898
Trench1 Hor	Gegham 1	0.000	0.000	0.000	1.000	0.000	0.000	0.000	0.000	0.000	0.000	0.000	0.000	0.000	0.000	0.246	-10.186	2.397	-4.430	2.174
Trench1 Hor	Gegham 1	0.000	0.000	0.000	1.000	0.000	0.000	0.000	0.000	0.000	0.000	0.000	0.000	0.000	0.000	-1.692	-12.273	2.326	-4.834	5.190
Trench1 Hor	Gegham 1	0.000	0.000	0.000	1.000	0.000	0.000	0.000	0.000	0.000	0.000	0.000	0.000	0.000	0.000	0.055	-10.852	3.084	-4.431	2.592
Trench1 Hor	Gegham 1	0.000	0.000	0.000	1.000	0.000	0.000	0.000	0.000	0.000	0.000	0.000	0.000	0.000	0.000	-0.467	-10.917	2.374	-3.912	2.484
Trench1 Hor	Gegham 1	0.000	0.000	0.000	1.000	0.000	0.000	0.000	0.000	0.000	0.000	0.000	0.000	0.000	0.000	-1.014	-11.101	3.094	-4.431	4.336
Trench1 Hor	Gegham 1	0.000	0.000	0.000	1.000	0.000	0.000	0.000	0.000	0.000	0.000	0.000	0.000	0.000	0.000	-0.695	-9.949	3.642	-4.614	3.115
Trench1 Hor	Gegham 1	0.000	0.000	0.000	1.000	0.000	0.000	0.000	0.000	0.000	0.000	0.000	0.000	0.000	0.000	-1.286	-10.396	2.860	-4.753	4.686
Trench1 Hor	Gegham 1	0.000	0.000	0.000	1.000	0.000	0.000	0.000	0.000	0.000	0.000	0.000	0.000	0.000	0.000	-0.887	-11.004	3.141	-4.682	3.014
Trench1 Hor	Gegham 1	0.000	0.000	0.000	1.000	0.000	0.000	0.000	0.000	0.000	0.000	0.000	0.000	0.000	0.000	-0.655	-10.932	3.458	-4.622	3.281
Trench1 Hor	Gegham 1	0.000	0.000	0.000	1.000	0.000	0.000	0.000	0.000	0.000	0.000	0.000	0.000	0.000	0.000	0.071	-10.786	3.573	-4.425	3.654
Trench1 Hor	Gegham 1	0.000	0.000	0.000	1.000	0.000	0.000	0.000	0.000	0.000	0.000	0.000	0.000	0.000	0.000	-0.205	-10.329	3.169	-4.500	3.132
Trench1 Hor	Kelbadjar	0.000	0.000	0.000	0.000	0.000	0.000	0.000	0.000	0.664	0.000	0.000	0.000	0.336	0.000	-5.933	-10.817	-0.982	2.687	-1.150
Trench1 Hor	Syunik	0.000	0.000	0.000	0.000	0.000	0.000	0.000	0.000	0.000	0.000	0.000	0.000	1.000	0.000	-6.936	-12.557	-1.649	3.128	-1.201
Trench1 Hor	Gegham 1	0.000	0.000	0.000	1.000	0.000	0.000	0.000	0.000	0.000	0.000	0.000	0.000	0.000	0.000	-0.408	-11.293	3.358	-4.081	2.158
Trench1 Hor	Gegham 1	0.000	0.000	0.000	1.000	0.000	0.000	0.000	0.000	0.000	0.000	0.000	0.000	0.000	0.000	-0.070	-11.345	1.667	-3.951	3.267
Trench1 Hor	Gegham 1	0.000	0.000	0.000	1.000	0.000	0.000	0.000	0.000	0.000	0.000	0.000	0.000	0.000	0.000	-1.127	-12.418	3.337	-4.524	4.151
Trench1 Hor	Gegham 1	0.000	0.000	0.000	1.000	0.000	0.000	0.000	0.000	0.000	0.000	0.000	0.000	0.000	0.000	0.085	-11.242	2.438	-4.461	6.776
Trench1 Hor	Syunik	0.000	0.000	0.000	0.000	0.000	0.000	0.000	0.000	0.441	0.000	0.000	0.000	0.559	0.000	-10.458	-9.956	-0.716	5.084	-1.975
Trench1 Hor	Syunik	0.000	0.000	0.000	0.000	0.000	0.000	0.000	0.000	0.000	0.000	0.000	0.000	1.000	0.000	-6.595	-13.668	-2.602	4.877	-1.076
Trench1 Hor	Gegham 1	0.000	0.000	0.000	1.000	0.000	0.000	0.000	0.000	0.000	0.000	0.000	0.000	0.000	0.000	-1.117	-10.410	3.325	-4.295	3.817
Trench1 Hor	Gegham 1	0.000	0.000	0.000	1.000	0.000	0.000	0.000	0.000	0.000	0.000	0.000	0.000	0.000	0.000	-0.284	-10.023	3.264	-4.636	4.468
obs obtool fr	Gegham 1	0.000	0.000	0.000	1.000	0.000	0.000	0.000	0.000	0.000	0.000	0.000	0.000	0.000	0.000	-0.731	-9.401	3.417	-4.251	1.845
obs obtool fr	Gegham 1	0.000	0.000	0.000	1.000	0.000	0.000	0.000	0.000	0.000	0.000	0.000	0.000	0.000	0.000	-2.118	-10.609	3.715	-4.284	2.127

Tr-1 Hor2 lar	Gegham 1	0.000	0.000	0.000	1.000	0.000	0.000	0.000	0.000	0.000	0.000	0.000	0.000	0.000	0.000	-1.752	-10.249	2.734	-4.335	4.137
Tr-1 Hor2 lar	Gegham 1	0.000	0.000	0.000	1.000	0.000	0.000	0.000	0.000	0.000	0.000	0.000	0.000	0.000	0.000	-0.703	-11.038	3.146	-4.419	3.418
Tr-1 Hor2 sm	Gegham 1	0.000	0.000	0.000	1.000	0.000	0.000	0.000	0.000	0.000	0.000	0.000	0.000	0.000	0.000	-1.519	-11.683	3.310	-4.617	3.521
Tr-1 Hor2 sm	Gegham 1	0.000	0.000	0.000	1.000	0.000	0.000	0.000	0.000	0.000	0.000	0.000	0.000	0.000	0.000	-0.729	-12.014	3.050	-4.773	3.437
Trench-2 Hor	Gegham 1	0.000	0.000	0.000	1.000	0.000	0.000	0.000	0.000	0.000	0.000	0.000	0.000	0.000	0.000	-1.777	-12.223	2.776	-4.171	2.639
Trench-2 Hor	Gegham 1	0.000	0.000	0.000	1.000	0.000	0.000	0.000	0.000	0.000	0.000	0.000	0.000	0.000	0.000	-1.414	-10.443	1.883	-3.903	4.443
Trench-2 Hor	Kelbadjar	0.000	0.000	0.000	0.000	0.000	0.000	0.000	0.000	0.679	0.000	0.000	0.000	0.321	0.000	-6.346	-11.481	-1.051	3.420	-1.274
Trench-2 Hor	Syunik	0.000	0.000	0.000	0.000	0.000	0.000	0.000	0.000	0.000	0.000	0.000	0.000	1.000	0.000	-7.032	-13.227	-1.870	3.936	-0.753
Trench-2 Hor	Syunik	0.000	0.000	0.000	0.000	0.000	0.000	0.000	0.000	0.000	0.000	0.000	0.000	1.000	0.000	-6.624	-12.225	-1.847	4.591	-1.279
Trench-2 Hor	Syunik	0.000	0.000	0.000	0.000	0.000	0.000	0.000	0.000	0.000	0.000	0.000	0.000	1.000	0.000	-6.325	-11.236	-1.516	3.794	-1.272
Trench-2 Hor	Gegham 1	0.000	0.000	0.000	1.000	0.000	0.000	0.000	0.000	0.000	0.000	0.000	0.000	0.000	0.000	-1.356	-11.718	2.322	-4.259	4.336
Trench-2 Hor	Gegham 1	0.000	0.000	0.000	1.000	0.000	0.000	0.000	0.000	0.000	0.000	0.000	0.000	0.000	0.000	-1.083	-11.121	2.213	-4.150	4.063
Trench-2 Hor	Gegham 1	0.000	0.000	0.000	1.000	0.000	0.000	0.000	0.000	0.000	0.000	0.000	0.000	0.000	0.000	-0.047	-9.345	3.760	-4.411	2.652
Trench-2 Hor	Gegham 1	0.000	0.000	0.000	1.000	0.000	0.000	0.000	0.000	0.000	0.000	0.000	0.000	0.000	0.000	-0.077	-11.094	3.666	-3.999	2.600
Trench-2 Hor	Gegham 1	0.000	0.000	0.000	1.000	0.000	0.000	0.000	0.000	0.000	0.000	0.000	0.000	0.000	0.000	-0.253	-12.027	2.404	-4.289	4.298
Trench-2 Hor	Gegham 1	0.000	0.000	0.000	1.000	0.000	0.000	0.000	0.000	0.000	0.000	0.000	0.000	0.000	0.000	-1.608	-12.408	2.391	-4.284	4.190
Trench-2 Hor	Gegham 1	0.000	0.000	0.000	1.000	0.000	0.000	0.000	0.000	0.000	0.000	0.000	0.000	0.000	0.000	0.745	-10.766	4.190	-5.041	3.421
PIT1. HOR1.	Gegham 1	0.000	0.000	0.000	1.000	0.000	0.000	0.000	0.000	0.000	0.000	0.000	0.000	0.000	0.000	-0.331	-11.501	3.380	-5.068	4.152
PIT1. HOR1.	Gegham 1	0.000	0.000	0.000	1.000	0.000	0.000	0.000	0.000	0.000	0.000	0.000	0.000	0.000	0.000	-0.272	-11.329	3.047	-4.346	3.389
PIT1. HOR1.	Gegham 1	0.000	0.000	0.000	1.000	0.000	0.000	0.000	0.000	0.000	0.000	0.000	0.000	0.000	0.000	-2.074	-11.882	2.239	-3.541	2.539
PIT1. HOR1.	Gegham 1	0.000	0.000	0.000	1.000	0.000	0.000	0.000	0.000	0.000	0.000	0.000	0.000	0.000	0.000	-1.622	-10.605	2.238	-3.673	2.146
PIT1. HOR1.	Gegham 1	0.000	0.000	0.000	1.000	0.000	0.000	0.000	0.000	0.000	0.000	0.000	0.000	0.000	0.000	-1.576	-10.950	2.413	-4.385	3.451
PIT1. HOR1.	Gegham 1	0.000	0.000	0.000	1.000	0.000	0.000	0.000	0.000	0.000	0.000	0.000	0.000	0.000	0.000	-2.197	-10.532	2.152	-4.388	3.111
PIT1. HOR1.	Gegham 1	0.000	0.000	0.000	1.000	0.000	0.000	0.000	0.000	0.000	0.000	0.000	0.000	0.000	0.000	-0.949	-10.745	3.284	-3.906	2.075
PIT1. HOR1.	Gegham 1	0.000	0.000	0.000	1.000	0.000	0.000	0.000	0.000	0.000	0.000	0.000	0.000	0.000	0.000	-1.007	-12.135	2.571	-4.244	2.422
PIT1. HOR1.	Arteni	0.000	1.000	0.000	0.000	0.000	0.000	0.000	0.000	0.000	0.000	0.000	0.000	0.000	0.000	-2.265	3.023	5.533	-3.960	-7.736
PIT1. HOR1.	Arteni	0.000	1.000	0.000	0.000	0.000	0.000	0.000	0.000	0.000	0.000	0.000	0.000	0.000	0.000	-4.017	1.518	4.768	-3.783	-8.349
PIT1. HOR1.	Syunik	0.000	0.000	0.000	0.000	0.000	0.000	0.000	0.000	0.000	0.000	0.000	0.000	1.000	0.000	-7.104	-12.674	-0.907	3.756	-1.651
PIT1. HOR1.	Syunik	0.000	0.000	0.000	0.000	0.000	0.000	0.000	0.000	0.000	0.000	0.000	0.000	1.000	0.000	-6.826	-12.848	-1.597	4.489	-1.051
PIT1. HOR1.	Syunik	0.000	0.000	0.000	0.000	0.000	0.000	0.000	0.000	0.000	0.000	0.000	0.000	1.000	0.000	-4.892	-11.603	-3.462	3.237	-0.869
PIT1. HOR1.	Syunik	0.000	0.000	0.000	0.000	0.000	0.000	0.000	0.000	0.000	0.000	0.000	0.000	1.000	0.000	-5.544	-12.899	-2.638	3.518	-0.986
PIT1. HOR1.	Kars-Arpaçay	0.000	0.000	0.000	0.000	0.000	0.000	0.000	1.000	0.000	0.000	0.000	0.000	0.000	0.000	-19.100	21.987	-3.857	-4.852	-0.037
PIT1. HOR1.	Kars-Arpaçay	0.000	0.000	0.000	0.000	0.000	0.000	0.000	1.000	0.000	0.000	0.000	0.000	0.000	0.000	-19.366	22.305	-4.311	-4.390	0.522
PIT1. HOR1.	Gegham 1	0.000	0.000	0.000	1.000	0.000	0.000	0.000	0.000	0.000	0.000	0.000	0.000	0.000	0.000	-0.350	-11.254	3.154	-4.582	5.547
PIT1. HOR1.	Gegham 1	0.000	0.000	0.000	1.000	0.000	0.000	0.000	0.000	0.000	0.000	0.000	0.000	0.000	0.000	-0.343	-9.610	4.240	-5.198	5.676
PIT1. HOR1.	Gegham 1	0.000	0.000	0.000	1.000	0.000	0.000	0.000	0.000	0.000	0.000	0.000	0.000	0.000	0.000	-0.932	-10.900	2.960	-4.432	4.959
PIT1. HOR1.	Gegham 1	0.000	0.000	0.000	1.000	0.000	0.000	0.000	0.000	0.000	0.000	0.000	0.000	0.000	0.000	-0.181	-10.942	3.318	-4.971	5.949
Tr-2 H-3 Sp-1	Kelbadjar	0.000	0.000	0.000	0.000	0.000	0.000	0.000	0.000	1.000	0.000	0.000	0.000	0.000	0.000	-8.593	-10.893	2.114	3.142	-1.305
Tr-2 H-3 Sp-1	Gegham 1	0.000	0.000	0.000	1.000	0.000	0.000	0.000	0.000	0.000	0.000	0.000	0.000	0.000	0.000	-0.949	-9.917	3.428	-4.460	3.038
Tr-2 H-3 Sp-1	Gegham 1	0.000	0.000	0.000	1.000	0.000	0.000	0.000	0.000	0.000	0.000	0.000	0.000	0.000	0.000	-1.589	-9.925	3.210	-3.803	2.141
Tr-2 H-3 Sp-1	Gegham 1	0.000	0.000	0.000	1.000	0.000	0.000	0.000	0.000	0.000	0.000	0.000	0.000	0.000	0.000	-0.032	-10.545	3.805	-4.714	3.317
Tr-2 H-3 Sp-1	Syunik	0.000	0.000	0.000	0.000	0.000	0.000	0.000	0.000	0.000	0.000	0.000	0.000	1.000	0.000	-5.737	-12.700	-1.347	3.177	-1.111
Tr-2 H-3 Sp-1	Syunik	0.000	0.000	0.000	0.000	0.000	0.000	0.000	0.000	0.000	0.000	0.000	0.000	1.000	0.000	-5.953	-11.292	-1.567	4.046	-1.545
Tr-2 H-3 Sp-1	Gegham 1	0.000	0.000	0.000	1.000	0.000	0.000	0.000	0.000	0.000	0.000	0.000	0.000	0.000	0.000	-0.314	-11.062	1.970	-3.982	5.144
Tr-2 H-3 Sp-1	Gegham 1	0.000	0.000	0.000	1.000	0.000	0.000	0.000	0.000	0.000	0.000	0.000	0.000	0.000	0.000	-1.673	-11.407	3.031	-3.942	2.490
Tr-2 H-3 Sp-1	Gegham 1	0.000	0.000	0.000	1.000	0.000	0.000	0.000	0.000	0.000	0.000	0.000	0.000	0.000	0.000	-0.774	-10.651	3.642	-4.372	2.664
Tr-2 H-3 Sp-1	Arteni	0.000	1.000	0.000	0.000	0.000	0.000	0.000	0.000	0.000	0.000	0.000	0.000	0.000	0.000	-1.459	2.928	3.793	-3.899	-6.912
Tr-2 H-3 Sp-1	Kelbadjar	0.000	0.000	0.000	0.000	0.000	0.000	0.000	0.000	1.000	0.000	0.000	0.000	0.000	0.000	-9.483	-9.264	0.852	4.274	-2.164
Tr-2 H-3 Sp-1	Gegham 1	0.000	0.000	0.000	1.000	0.000	0.000	0.000	0.000	0.000	0.000	0.000	0.000	0.000	0.000	-0.926	-10.214	3.009	-3.897	3.705
Tr-2 H-3 Sp-1	Gegham 1	0.000	0.000	0.000	1.000	0.000	0.000	0.000	0.000	0.000	0.000	0.000	0.000	0.000	0.000	-0.524	-10.790	3.815	-4.237	2.802
Tr-2 H-3 Sp-1	Gegham 1	0.000	0.000	0.000	1.000	0.000	0.000	0.000	0.000	0.000	0.000	0.000	0.000	0.000	0.000	-1.469	-11.687	2.827	-4.110	3.136
Tr-2 H-3 Sp-1	Arteni	0.000	1.000	0.000	0.000	0.000	0.000	0.000	0.000	0.000	0.000	0.000	0.000	0.000	0.000	-1.675	2.660	3.689	-2.886	-5.327
Tr-2 H-3 Sp-1	Gegham 1	0.000	0.000	0.000	1.000	0.000	0.000	0.000	0.000	0.000	0.000	0.000	0.000	0.000	0.000	-2.155	-10.937	2.733	-4.331	2.374
Tr-2 H-3 Sp-1	Gegham 1	0.000	0.000	0.000	1.000	0.000	0.000	0.000	0.000	0.000	0.000	0.000	0.000	0.000	0.000	-0.575	-11.497	2.636	-4.654	4.412
Trench-2 Hor	Kelbadjar	0.000	0.000	0.000	0.000	0.000	0.000	0.000	0.000	1.000	0.000	0.000	0.000	0.000	0.000	-8.551	-10.148	1.617	3.685	-1.235
Trench-2 Hor	Syunik	0.000	0.000	0.000	0.000	0.000	0.000	0.000	0.000	0.043	0.000	0.000	0.000	0.957	0.000	-5.986	-11.415	-0.448	3.399	-1.579
Trench-2 Hor	Gegham 1	0.000	0.000	0.000	1.000	0.000	0.000	0.000	0.000	0.000	0.000	0.000	0.000	0.000	0.000	-1.363	-10.761	2.023	-3.191	2.220
Trench-2 Hor	Gegham 1	0.000	0.000	0.000	1.000	0.000	0.000	0.000	0.000	0.000	0.000	0.000	0.000	0.000	0.000	-0.780	-10.228	4.158	-3.946	2.673
Trench-2 Hor	Syunik	0.000	0.000	0.000	0.000	0.000	0.000	0.000	0.000	0.000	0.000	0.000	0.000	1.000	0.000	-7.978	-16.912	-3.347	1.901	-0.124
Trench-2 Hor	Gegham 1	0.000	0.000	0.000	1.000	0.000	0.000	0.000	0.000	0.000	0.000	0.000	0.000	0.000	0.000	-1.284	-9.992	2.988	-4.415	4.398
Trench-2 Hor	Gegham 1	0.000	0.000	0.000	1.000	0.000	0.000	0.000	0.000	0.000	0.000	0.000	0.000	0.000	0.000	-0.212	-11.620	4.734	-4.533	3.040
Trench-2 Hor	Gegham 1	0.000	0.000	0.000	1.000	0.000	0.000	0.000	0.000	0.000	0.000	0.000	0.000	0.000	0.000	-1.276	-13.208	2.711	-4.234	3.535
Trench-2 Hor	Gegham 1	0.000	0.000	0.000	1.000	0.000	0.000	0.000	0.000	0.000	0.000	0.000	0.000	0.000	0.000	-1.080	-10.002	2.579	-4.342	5.868
Trench-2 Hor	Gegham 1	0.000	0.000	0.000	1.000	0.000	0.000	0.000	0.000	0.000	0.000	0.000	0.000	0.000	0.000	-0.990	-11.530	3.845	-4.494	2.417
Trench-2 Hor	Syunik	0.000	0.000	0.000	0.000	0.000	0.000	0.000	0.000	0.000	0.000	0.000	0.000	1.000	0.000	-7.003	-14.277	-3.860	4.716	-1.173
Trench-2 Hor	Kelbadjar	0.000	0.000	0.000	0.000	0.000	0.000	0.000	0.000	1.000	0.000	0.000	0.000	0.000	0.000	-9.310	-8.626	1.270	3.611	-1.535
Trench-2 Hor	Gegham 1	0.000	0.000	0.000	1.000	0.000	0.000	0.000	0.000	0.000	0.000	0.000	0.000	0.000	0.000	-0.488	-10.214	3.548	-4.094	3.123

Trench-2 Hor	Gegham 1	0.000	0.000	0.000	1.000	0.000	0.000	0.000	0.000	0.000	0.000	0.000	0.000	0.000	0.000	-0.344	-8.642	3.969	-5.077	5.165
Trench-2 Hor	Gegham 1	0.000	0.000	0.000	1.000	0.000	0.000	0.000	0.000	0.000	0.000	0.000	0.000	0.000	0.000	-1.267	-11.220	4.241	-4.373	2.850
Trench-2 Hor	Syunik	0.000	0.000	0.000	0.000	0.000	0.000	0.000	0.000	0.000	0.000	0.000	0.000	1.000	0.000	-7.000	-12.654	-1.193	2.450	-0.799
Trench-2 Hor	Gegham 1	0.000	0.000	0.000	1.000	0.000	0.000	0.000	0.000	0.000	0.000	0.000	0.000	0.000	0.000	0.198	-10.593	4.168	-4.801	2.778
Trench-2 Hor	Gegham 1	0.000	0.000	0.000	1.000	0.000	0.000	0.000	0.000	0.000	0.000	0.000	0.000	0.000	0.000	-1.285	-12.420	2.539	-4.547	2.949
Trench-2 Hor	Gegham 1	0.000	0.000	0.000	1.000	0.000	0.000	0.000	0.000	0.000	0.000	0.000	0.000	0.000	0.000	-0.690	-11.689	3.280	-4.597	3.655
Trench-2 Hor	Arteni	0.000	1.000	0.000	0.000	0.000	0.000	0.000	0.000	0.000	0.000	0.000	0.000	0.000	0.000	-3.592	2.691	4.722	-3.756	-4.652
Trench-2 Hor	Gegham 1	0.000	0.000	0.000	1.000	0.000	0.000	0.000	0.000	0.000	0.000	0.000	0.000	0.000	0.000	-0.082	-12.314	4.106	-4.788	3.056
Trench-2 Hor	Gegham 1	0.000	0.000	0.000	1.000	0.000	0.000	0.000	0.000	0.000	0.000	0.000	0.000	0.000	0.000	-0.454	-11.406	3.337	-4.103	3.034
Trench-2 Hor	Gegham 1	0.000	0.000	0.000	1.000	0.000	0.000	0.000	0.000	0.000	0.000	0.000	0.000	0.000	0.000	1.803	-9.516	2.800	-3.505	7.481
Trench-2 Hor	Gegham 1	0.000	0.000	0.000	1.000	0.000	0.000	0.000	0.000	0.000	0.000	0.000	0.000	0.000	0.000	-0.868	-10.188	3.927	-4.854	5.436
Trench-2 Hor	Gegham 1	0.000	0.000	0.000	1.000	0.000	0.000	0.000	0.000	0.000	0.000	0.000	0.000	0.000	0.000	1.492	-9.973	3.232	-4.404	3.586
Trench-2 Hor	Gegham 1	0.000	0.000	0.000	1.000	0.000	0.000	0.000	0.000	0.000	0.000	0.000	0.000	0.000	0.000	-0.837	-10.697	3.244	-4.643	4.016
Trench-2 Hor	Syunik	0.000	0.000	0.000	0.000	0.000	0.000	0.000	0.000	0.000	0.000	0.000	0.000	1.000	0.000	-6.215	-12.776	-2.929	3.999	-1.538
Trench-2 Hor	Gegham 1	0.000	0.000	0.000	1.000	0.000	0.000	0.000	0.000	0.000	0.000	0.000	0.000	0.000	0.000	-1.434	-11.194	3.754	-5.037	7.342
Trench-2 Hor	Gegham 1	0.000	0.000	0.000	1.000	0.000	0.000	0.000	0.000	0.000	0.000	0.000	0.000	0.000	0.000	-0.256	-11.498	2.070	-4.698	7.089
Trench-2 Hor	Gegham 1	0.000	0.000	0.000	1.000	0.000	0.000	0.000	0.000	0.000	0.000	0.000	0.000	0.000	0.000	-1.773	-10.953	3.156	-4.411	5.676
Trench-2 Hor	Gegham 1	0.000	0.000	0.000	1.000	0.000	0.000	0.000	0.000	0.000	0.000	0.000	0.000	0.000	0.000	-1.161	-11.030	3.462	-5.000	6.756
Trench-2 Hor	Gegham 1	0.000	0.000	0.000	1.000	0.000	0.000	0.000	0.000	0.000	0.000	0.000	0.000	0.000	0.000	-1.461	-11.382	3.868	-4.011	3.398
Trench-2 Hor	Gegham 1	0.000	0.000	0.000	1.000	0.000	0.000	0.000	0.000	0.000	0.000	0.000	0.000	0.000	0.000	-1.509	-10.434	2.255	-4.237	4.372
Trench-2 Hor	Gegham 1	0.000	0.000	0.000	1.000	0.000	0.000	0.000	0.000	0.000	0.000	0.000	0.000	0.000	0.000	-1.696	-11.234	3.213	-4.536	6.606
Trench-2 Hor	Arteni	0.000	1.000	0.000	0.000	0.000	0.000	0.000	0.000	0.000	0.000	0.000	0.000	0.000	0.000	-1.092	3.448	3.993	-3.684	-7.064
Tr-2 Hor 5 Sc	Gegham 1	0.000	0.000	0.000	1.000	0.000	0.000	0.000	0.000	0.000	0.000	0.000	0.000	0.000	0.000	-0.995	-10.433	3.254	-4.689	2.959
Tr-2 Hor 5 Sc	Syunik	0.000	0.000	0.000	0.000	0.000	0.000	0.000	0.000	0.000	0.000	0.000	0.000	1.000	0.000	-6.784	-13.233	-1.511	3.109	-1.415
Tr-2 Hor 5 Sc	Gegham 1	0.000	0.000	0.000	1.000	0.000	0.000	0.000	0.000	0.000	0.000	0.000	0.000	0.000	0.000	-1.141	-12.204	3.040	-4.313	1.659
Tr-2 Hor 5 Sc	Gegham 1	0.000	0.000	0.000	1.000	0.000	0.000	0.000	0.000	0.000	0.000	0.000	0.000	0.000	0.000	-1.438	-10.612	2.997	-4.577	3.835
Tr-2 Hor 5 Sc	Gegham 1	0.000	0.000	0.000	1.000	0.000	0.000	0.000	0.000	0.000	0.000	0.000	0.000	0.000	0.000	0.493	-9.548	3.584	-4.744	3.327
Tr-2 Hor 5 Sc	Gegham 1	0.000	0.000	0.000	1.000	0.000	0.000	0.000	0.000	0.000	0.000	0.000	0.000	0.000	0.000	-0.855	-11.248	3.432	-4.340	3.603
Tr-2 Hor 5 Sc	Syunik	0.000	0.000	0.000	0.000	0.000	0.000	0.000	0.000	0.000	0.000	0.000	0.000	1.000	0.000	-6.681	-11.369	-1.630	3.140	-1.261
Tr-2 Hor 5 Sc	Gegham 1	0.000	0.000	0.000	1.000	0.000	0.000	0.000	0.000	0.000	0.000	0.000	0.000	0.000	0.000	-1.257	-9.686	3.393	-4.338	1.654
Tr-2 Hor 5 Sc	Gegham 1	0.000	0.000	0.000	1.000	0.000	0.000	0.000	0.000	0.000	0.000	0.000	0.000	0.000	0.000	-0.457	-11.917	3.084	-4.838	5.145
Tr-2 Hor 5 Sc	Gegham 1	0.000	0.000	0.000	1.000	0.000	0.000	0.000	0.000	0.000	0.000	0.000	0.000	0.000	0.000	-0.607	-11.173	2.807	-4.496	3.041
Tr-2 Hor 5 Sc	Gegham 1	0.000	0.000	0.000	1.000	0.000	0.000	0.000	0.000	0.000	0.000	0.000	0.000	0.000	0.000	-1.213	-11.715	3.672	-4.535	3.063
Tr-2 Hor 5 Sc	Gegham 1	0.000	0.000	0.000	1.000	0.000	0.000	0.000	0.000	0.000	0.000	0.000	0.000	0.000	0.000	-0.304	-11.454	2.856	-4.187	3.401
Tr-2 Hor 5 Sc	Gegham 1	0.000	0.000	0.000	1.000	0.000	0.000	0.000	0.000	0.000	0.000	0.000	0.000	0.000	0.000	-0.322	-11.274	2.746	-4.271	2.972
Tr-2 Hor 5 Sc	Gegham 1	0.000	0.000	0.000	1.000	0.000	0.000	0.000	0.000	0.000	0.000	0.000	0.000	0.000	0.000	-1.014	-11.911	2.628	-4.221	3.776
Tr-2 Hor 5 Sc	Gegham 1	0.000	0.000	0.000	1.000	0.000	0.000	0.000	0.000	0.000	0.000	0.000	0.000	0.000	0.000	-2.061	-11.480	3.033	-4.218	2.920
Tr-2 Hor 5 Sc	Gegham 1	0.000	0.000	0.000	1.000	0.000	0.000	0.000	0.000	0.000	0.000	0.000	0.000	0.000	0.000	0.409	-11.127	3.445	-4.652	4.487
Tr-2 Hor 5 Sc	Gegham 1	0.000	0.000	0.000	1.000	0.000	0.000	0.000	0.000	0.000	0.000	0.000	0.000	0.000	0.000	0.320	-11.294	2.497	-4.701	4.637
Tr-2 Hor 5 Sc	Gegham 1	0.000	0.000	0.000	1.000	0.000	0.000	0.000	0.000	0.000	0.000	0.000	0.000	0.000	0.000	-0.751	-10.545	2.613	-3.159	1.878
Tr-2 Hor 5 Sc	Gegham 1	0.000	0.000	0.000	1.000	0.000	0.000	0.000	0.000	0.000	0.000	0.000	0.000	0.000	0.000	1.666	-12.038	1.687	-4.751	5.548
Tr-2 Hor-4 Sp	Gegham 1	0.000	0.000	0.000	1.000	0.000	0.000	0.000	0.000	0.000	0.000	0.000	0.000	0.000	0.000	-0.760	-11.259	3.337	-4.576	4.037
Tr-2 Hor-4 Sp	Gegham 1	0.000	0.000	0.000	1.000	0.000	0.000	0.000	0.000	0.000	0.000	0.000	0.000	0.000	0.000	-0.626	-12.274	3.833	-4.423	5.531
Tr-2 Hor-4 Sp	Arteni	0.000	1.000	0.000	0.000	0.000	0.000	0.000	0.000	0.000	0.000	0.000	0.000	0.000	0.000	-4.227	3.022	4.813	-3.344	-5.648
Tr-2 Hor-4 Sp	Gegham 1	0.000	0.000	0.000	1.000	0.000	0.000	0.000	0.000	0.000	0.000	0.000	0.000	0.000	0.000	-0.727	-9.081	4.574	-4.659	5.896
Tr-2 Hor-4 Sp	Gegham 1	0.000	0.000	0.000	1.000	0.000	0.000	0.000	0.000	0.000	0.000	0.000	0.000	0.000	0.000	-0.052	-10.018	2.913	-4.475	3.493
Tr-2 Hor-4 Sp	Syunik	0.000	0.000	0.000	0.000	0.000	0.000	0.000	0.000	0.000	0.000	0.000	0.000	1.000	0.000	-6.469	-12.551	-2.388	3.523	-0.588
Tr-2 Hor-4 Sp	Gegham 1	0.000	0.000	0.000	1.000	0.000	0.000	0.000	0.000	0.000	0.000	0.000	0.000	0.000	0.000	0.505	-9.514	4.280	-4.929	3.189
Tr-2 Hor-4 Sp	Gegham 1	0.000	0.000	0.000	1.000	0.000	0.000	0.000	0.000	0.000	0.000	0.000	0.000	0.000	0.000	-0.386	-10.342	2.831	-4.033	2.995
Tr-2 Hor-4 Sp	Syunik	0.000	0.000	0.000	0.000	0.000	0.000	0.000	0.000	0.000	0.000	0.000	0.000	1.000	0.000	-8.187	-16.281	-2.310	2.419	-0.088
Tr-2 Hor-4 Sp	Kelbadjar	0.000	0.000	0.000	0.000	0.000	0.000	0.000	0.000	1.000	0.000	0.000	0.000	0.000	0.000	-8.309	-7.891	2.822	2.279	-1.358
Tr-2 Hor-4 Sp	Arteni	0.000	0.984	0.000	0.000	0.016	0.000	0.000	0.000	0.000	0.000	0.000	0.000	0.000	0.000	-0.587	2.043	3.909	-3.458	-6.383
Tr-2 Hor-4 Sp	Gegham 1	0.000	0.000	0.000	1.000	0.000	0.000	0.000	0.000	0.000	0.000	0.000	0.000	0.000	0.000	-0.381	-9.622	4.117	-4.132	3.233
Tr-2 Hor-4 Sp	Syunik	0.000	0.000	0.000	0.000	0.000	0.000	0.000	0.000	0.000	0.000	0.000	0.000	1.000	0.000	-7.982	-17.206	-3.043	2.530	-0.843
Tr-2 Hor-4 Sp	Gegham 1	0.000	0.000	0.000	1.000	0.000	0.000	0.000	0.000	0.000	0.000	0.000	0.000	0.000	0.000	-1.333	-10.300	2.722	-4.166	2.886
Tr-2 Hor-4 Sp	Kelbadjar	0.000	0.000	0.000	0.000	0.000	0.000	0.000	0.000	1.000	0.000	0.000	0.000	0.000	0.000	-8.932	-10.122	-0.253	3.463	-1.164
Tr-2 Hor-4 Sp	Gegham 1	0.000	0.000	0.000	1.000	0.000	0.000	0.000	0.000	0.000	0.000	0.000	0.000	0.000	0.000	-0.258	-10.647	3.909	-4.040	3.266
Tr-2 Hor-4 Sp	Gegham 1	0.000	0.000	0.000	1.000	0.000	0.000	0.000	0.000	0.000	0.000	0.000	0.000	0.000	0.000	3.036	-9.835	2.903	-4.342	6.633
Tr-2 Hor-4 Sp	Arteni	0.000	1.000	0.000	0.000	0.000	0.000	0.000	0.000	0.000	0.000	0.000	0.000	0.000	0.000	-4.756	3.082	6.502	-3.564	-7.440
Tr-2 Hor-4 Sp	Syunik	0.000	0.000	0.000	0.000	0.000	0.000	0.000	0.000	0.000	0.000	0.000	0.000	1.000	0.000	-7.429	-13.316	-1.329	3.974	-1.531
Tr-2 Hor-4 Sp	Gegham 1	0.000	0.000	0.000	1.000	0.000	0.000	0.000	0.000	0.000	0.000	0.000	0.000	0.000	0.000	-1.453	-10.155	3.749	-3.750	1.487
Tr-2 Hor-4 Sp	Gegham 1	0.000	0.000	0.000	1.000	0.000	0.000	0.000	0.000	0.000	0.000	0.000	0.000	0.000	0.000	-1.679	-10.666	3.208	-4.332	5.798
Tr-2 Hor-4 Sp	Arteni	0.000	1.000	0.000	0.000	0.000	0.000	0.000	0.000	0.000	0.000	0.000	0.000	0.000	0.000	-4.692	1.990	5.970	-3.589	-7.878
Tr-2 Hor-4 Sp	Gegham 1	0.000	0.000	0.000	1.000	0.000	0.000	0.000	0.000	0.000	0.000	0.000	0.000	0.000	0.000	0.024	-9.586	2.816	-5.017	7.121
Tr-2 Hor-4 Sp	Gegham 1	0.000	0.000	0.000	1.000	0.000	0.000	0.000	0.000	0.000	0.000	0.000	0.000	0.000	0.000	4.047	-9.940	1.310	-4.005	5.467
Tr-2 Hor-4 Sp	Gegham 1	0.000	0.000	0.000	1.000	0.000	0.000	0.000	0.000	0.000	0.000	0.000	0.000	0.000	0.000	-0.352	-11.638	2.131	-4.457	4.936
Tr-2 Hor-4 Sp	Gegham 1	0.000	0.000	0.000	1.000	0.000	0.000	0.000	0.000	0.000	0.000	0.000	0.000	0.000	0.000	-1.530	-11.167	3.294	-4.687	5.153

Tr-2 Hor-2 Sp	Kelbadjar	0.000	0.000	0.000	0.000	0.000	0.000	0.000	0.000	1.000	0.000	0.000	0.000	0.000	0.000	-9.332	-11.013	0.296	4.483	-1.446
Tr-2 Hor-2 Sp	Gegham 1	0.000	0.000	0.000	1.000	0.000	0.000	0.000	0.000	0.000	0.000	0.000	0.000	0.000	0.000	-1.349	-12.576	3.206	-4.759	2.648
Tr-2 Hor-2 Sp	Gegham 1	0.000	0.000	0.000	1.000	0.000	0.000	0.000	0.000	0.000	0.000	0.000	0.000	0.000	0.000	0.059	-9.404	3.895	-4.455	3.879
Tr-2 Hor-2 Sp	Gegham 1	0.000	0.000	0.000	1.000	0.000	0.000	0.000	0.000	0.000	0.000	0.000	0.000	0.000	0.000	-0.967	-10.037	3.417	-4.496	3.406
Tr-2 Hor-2 Sp	Kelbadjar	0.000	0.000	0.000	0.000	0.000	0.000	0.000	0.000	1.000	0.000	0.000	0.000	0.000	0.000	-9.722	-10.242	0.028	4.538	-1.365
Tr-2 Hor-2 Sp	Gegham 1	0.000	0.000	0.000	1.000	0.000	0.000	0.000	0.000	0.000	0.000	0.000	0.000	0.000	0.000	-0.998	-12.174	2.283	-3.951	4.661
Tr-2 Hor-2 Sp	Gegham 1	0.000	0.000	0.000	1.000	0.000	0.000	0.000	0.000	0.000	0.000	0.000	0.000	0.000	0.000	-1.160	-11.509	2.492	-4.467	2.876
Tr-2 Hor-2 Sp	Kelbadjar	0.000	0.000	0.000	0.000	0.000	0.000	0.000	0.000	1.000	0.000	0.000	0.000	0.000	0.000	-8.928	-9.347	0.730	4.284	-2.066
Tr-2 Hor-2 Sp	Gegham 1	0.000	0.000	0.000	1.000	0.000	0.000	0.000	0.000	0.000	0.000	0.000	0.000	0.000	0.000	1.364	-11.495	2.551	-4.010	3.148
Tr-2 Hor-2 Sp	Gegham 1	0.000	0.000	0.000	1.000	0.000	0.000	0.000	0.000	0.000	0.000	0.000	0.000	0.000	0.000	-1.615	-11.544	2.915	-4.626	5.843
Tr-2 Hor-2 Sp	Gegham 1	0.000	0.000	0.000	1.000	0.000	0.000	0.000	0.000	0.000	0.000	0.000	0.000	0.000	0.000	0.071	-11.110	2.890	-4.508	6.264
Tr-2 Hor-2 Sp	Gegham 1	0.000	0.000	0.000	1.000	0.000	0.000	0.000	0.000	0.000	0.000	0.000	0.000	0.000	0.000	-1.248	-11.486	3.083	-4.279	3.690
Tr-2 Hor-2 Sp	Gegham 1	0.000	0.000	0.000	1.000	0.000	0.000	0.000	0.000	0.000	0.000	0.000	0.000	0.000	0.000	-1.144	-12.190	2.968	-4.607	4.182
Tr-2 Hor-2 Sp	Gegham 1	0.000	0.000	0.000	1.000	0.000	0.000	0.000	0.000	0.000	0.000	0.000	0.000	0.000	0.000	0.127	-11.110	3.185	-4.715	5.227
Tr-2 Hor-2 Sp	Gegham 1	0.000	0.000	0.000	1.000	0.000	0.000	0.000	0.000	0.000	0.000	0.000	0.000	0.000	0.000	-1.176	-9.485	4.351	-4.429	2.134
Trench-2 Hor	Arteni	0.000	1.000	0.000	0.000	0.000	0.000	0.000	0.000	0.000	0.000	0.000	0.000	0.000	0.000	-3.165	1.318	5.468	-4.051	-6.214
Trench-2 Hor	Kelbadjar	0.000	0.000	0.000	0.000	0.000	0.000	0.000	0.000	1.000	0.000	0.000	0.000	0.000	0.000	-5.758	-11.302	-0.786	2.677	-0.517
Trench-2 Hor	Gegham 1	0.000	0.000	0.000	1.000	0.000	0.000	0.000	0.000	0.000	0.000	0.000	0.000	0.000	0.000	-0.692	-10.003	2.771	-4.230	3.232
Trench-2 Hor	Gegham 1	0.000	0.000	0.000	1.000	0.000	0.000	0.000	0.000	0.000	0.000	0.000	0.000	0.000	0.000	-1.198	-10.610	2.960	-4.360	2.806
Trench-2 Hor	Gegham 1	0.000	0.000	0.000	1.000	0.000	0.000	0.000	0.000	0.000	0.000	0.000	0.000	0.000	0.000	-1.086	-12.222	2.873	-4.269	2.927
Trench-2 Hor	Gegham 1	0.000	0.000	0.000	1.000	0.000	0.000	0.000	0.000	0.000	0.000	0.000	0.000	0.000	0.000	-0.670	-10.647	2.963	-4.198	4.156
Trench-2 Hor	Gegham 1	0.000	0.000	0.000	1.000	0.000	0.000	0.000	0.000	0.000	0.000	0.000	0.000	0.000	0.000	-0.996	-10.884	2.786	-4.477	3.534
Trench-2 Hor	Syunik	0.000	0.000	0.000	0.000	0.000	0.000	0.000	0.000	0.000	0.000	0.000	0.000	1.000	0.000	-7.786	-14.044	-2.320	4.143	-0.651
Trench-2 Hor	Gegham 1	0.000	0.000	0.000	1.000	0.000	0.000	0.000	0.000	0.000	0.000	0.000	0.000	0.000	0.000	0.099	-10.271	4.275	-4.425	2.470
Trench-2 Hor	Gegham 1	0.000	0.000	0.000	1.000	0.000	0.000	0.000	0.000	0.000	0.000	0.000	0.000	0.000	0.000	-1.245	-11.626	2.868	-4.825	4.637
Trench-2 Hor	Gegham 1	0.000	0.000	0.000	1.000	0.000	0.000	0.000	0.000	0.000	0.000	0.000	0.000	0.000	0.000	2.636	-8.415	1.775	-3.997	5.099
Trench-2 Hor	Gegham 1	0.000	0.000	0.000	1.000	0.000	0.000	0.000	0.000	0.000	0.000	0.000	0.000	0.000	0.000	0.657	-10.953	2.417	-4.319	3.130
Trench-2 Hor	Gegham 1	0.000	0.000	0.000	1.000	0.000	0.000	0.000	0.000	0.000	0.000	0.000	0.000	0.000	0.000	0.702	-11.306	2.717	-3.969	3.525
Trench-2 Hor	Gegham 1	0.000	0.000	0.000	1.000	0.000	0.000	0.000	0.000	0.000	0.000	0.000	0.000	0.000	0.000	0.035	-10.901	3.387	-4.815	4.642
Trench-2 Hor	Gegham 1	0.000	0.000	0.000	1.000	0.000	0.000	0.000	0.000	0.000	0.000	0.000	0.000	0.000	0.000	-0.598	-10.316	2.552	-4.182	3.766
Trench-2 Hor	Gegham 1	0.000	0.000	0.000	1.000	0.000	0.000	0.000	0.000	0.000	0.000	0.000	0.000	0.000	0.000	-0.789	-11.004	3.320	-4.522	2.991
Trench-2 Hor	Gegham 1	0.000	0.000	0.000	1.000	0.000	0.000	0.000	0.000	0.000	0.000	0.000	0.000	0.000	0.000	-1.785	-10.727	2.249	-2.417	3.116
Trench-2 Hor	Gegham 1	0.000	0.000	0.000	1.000	0.000	0.000	0.000	0.000	0.000	0.000	0.000	0.000	0.000	0.000	-1.171	-11.680	2.190	-3.919	5.807
Trench-2 Hor	Gegham 1	0.000	0.000	0.000	1.000	0.000	0.000	0.000	0.000	0.000	0.000	0.000	0.000	0.000	0.000	-0.293	-11.451	4.239	-4.408	4.595
Trench-2 Hor	Gegham 2	0.000	0.059	0.000	0.000	0.941	0.000	0.000	0.000	0.000	0.000	0.000	0.000	0.000	0.000	-0.588	3.431	4.221	-1.049	-1.254
Trench-2 Hor	Gegham 1	0.000	0.000	0.000	1.000	0.000	0.000	0.000	0.000	0.000	0.000	0.000	0.000	0.000	0.000	-1.328	-10.669	2.787	-4.134	3.719
Trench-2 Hor	Gegham 1	0.000	0.000	0.000	1.000	0.000	0.000	0.000	0.000	0.000	0.000	0.000	0.000	0.000	0.000	0.846	-9.876	3.946	-4.653	8.033
Trench-2 Hor	Gegham 1	0.000	0.000	0.000	1.000	0.000	0.000	0.000	0.000	0.000	0.000	0.000	0.000	0.000	0.000	0.379	-9.773	3.867	-4.568	4.051
Trench-2 Hor	Gegham 1	0.000	0.000	0.000	1.000	0.000	0.000	0.000	0.000	0.000	0.000	0.000	0.000	0.000	0.000	0.548	-11.840	0.867	-4.161	6.991
Trench-2 Hor	Gegham 1	0.000	0.000	0.000	1.000	0.000	0.000	0.000	0.000	0.000	0.000	0.000	0.000	0.000	0.000	-0.672	-11.443	3.500	-4.325	4.004
Trench-2 Hor	Gegham 1	0.000	0.000	0.000	1.000	0.000	0.000	0.000	0.000	0.000	0.000	0.000	0.000	0.000	0.000	-1.339	-9.597	2.524	-4.033	4.623
Trench-2 Hor	Gegham 1	0.000	0.000	0.000	1.000	0.000	0.000	0.000	0.000	0.000	0.000	0.000	0.000	0.000	0.000	-1.169	-10.699	3.666	-4.730	4.139
Trench-2 Hor	Gegham 1	0.000	0.000	0.000	1.000	0.000	0.000	0.000	0.000	0.000	0.000	0.000	0.000	0.000	0.000	-0.850	-11.492	2.969	-4.230	4.759
Trench-2 Hor	Gegham 1	0.000	0.000	0.000	1.000	0.000	0.000	0.000	0.000	0.000	0.000	0.000	0.000	0.000	0.000	-1.512	-8.776	3.448	-2.490	1.054
Trench-2 Hor	Gegham 1	0.000	0.000	0.000	1.000	0.000	0.000	0.000	0.000	0.000	0.000	0.000	0.000	0.000	0.000	-0.670	-11.099	3.171	-4.493	5.679
Trench-2 Hor	Gegham 1	0.000	0.000	0.000	1.000	0.000	0.000	0.000	0.000	0.000	0.000	0.000	0.000	0.000	0.000	-1.056	-9.573	4.329	-4.885	4.914
Trench-2 Hor	Gegham 1	0.000	0.000	0.000	1.000	0.000	0.000	0.000	0.000	0.000	0.000	0.000	0.000	0.000	0.000	-0.364	-10.077	3.606	-4.633	4.923
Trench-2 Hor	Gegham 1	0.000	0.000	0.000	1.000	0.000	0.000	0.000	0.000	0.000	0.000	0.000	0.000	0.000	0.000	-0.536	-12.092	2.942	-4.497	3.762
Trench-2 Hor	Gegham 1	0.000	0.000	0.000	1.000	0.000	0.000	0.000	0.000	0.000	0.000	0.000	0.000	0.000	0.000	0.067	-10.505	3.700	-4.710	5.843
Trench-2 Hor	Gegham 1	0.000	0.000	0.000	1.000	0.000	0.000	0.000	0.000	0.000	0.000	0.000	0.000	0.000	0.000	0.543	-9.595	3.964	-4.993	6.048
Trench-2 Hor	Gegham 1	0.000	0.000	0.000	1.000	0.000	0.000	0.000	0.000	0.000	0.000	0.000	0.000	0.000	0.000	-0.669	-10.129	3.316	-4.307	4.457
Trench-2 Hor	Gegham 1	0.000	0.000	0.000	1.000	0.000	0.000	0.000	0.000	0.000	0.000	0.000	0.000	0.000	0.000	-2.976	-12.624	1.787	-3.829	5.010
Trench-2 Hor	Gegham 1	0.000	0.000	0.000	1.000	0.000	0.000	0.000	0.000	0.000	0.000	0.000	0.000	0.000	0.000	-3.170	-11.836	2.957	-4.108	2.986
smaller bag	Gegham 1	0.000	0.000	0.000	1.000	0.000	0.000	0.000	0.000	0.000	0.000	0.000	0.000	0.000	0.000	-0.057	-11.016	2.109	-3.868	3.927
smaller bag	Gegham 1	0.000	0.000	0.000	1.000	0.000	0.000	0.000	0.000	0.000	0.000	0.000	0.000	0.000	0.000	-1.251	-10.296	2.728	-4.388	4.174
smaller bag	Syunik	0.000	0.000	0.000	0.000	0.000	0.000	0.000	0.000	0.000	0.000	0.000	0.000	1.000	0.000	-7.324	-12.337	-1.313	4.252	-1.729
smaller bag	Gegham 1	0.000	0.000	0.000	1.000	0.000	0.000	0.000	0.000	0.000	0.000	0.000	0.000	0.000	0.000	-4.267	-10.452	3.759	-4.216	4.241
smaller bag	Gegham 1	0.000	0.000	0.000	1.000	0.000	0.000	0.000	0.000	0.000	0.000	0.000	0.000	0.000	0.000	-0.790	-10.838	3.384	-4.745	3.803
smaller bag	Gegham 1	0.000	0.000	0.000	1.000	0.000	0.000	0.000	0.000	0.000	0.000	0.000	0.000	0.000	0.000	-1.168	-11.054	3.124	-4.734	4.214
smaller bag	Gegham 1	0.000	0.000	0.000	1.000	0.000	0.000	0.000	0.000	0.000	0.000	0.000	0.000	0.000	0.000	0.083	-11.231	2.239	-3.835	3.951
smaller bag	Tsaghkunyat	0.000	0.000	0.000	0.000	0.000	0.000	0.000	0.000	0.000	0.000	0.000	0.000	0.000	1.000	34.521	1.280	-5.435	1.798	-1.070
smaller bag	Gegham 1	0.000	0.000	0.000	1.000	0.000	0.000	0.000	0.000	0.000	0.000	0.000	0.000	0.000	0.000	-1.266	-10.882	2.270	-4.269	3.828
Tr-1 H-1 Sp-2	Gegham 1	0.000	0.000	0.000	1.000	0.000	0.000	0.000	0.000	0.000	0.000	0.000	0.000	0.000	0.000	-0.978	-10.191	3.765	-4.335	2.492
Tr-1 H-1 Sp-2	Arteni	0.000	1.000	0.000	0.000	0.000	0.000	0.000	0.000	0.000	0.000	0.000	0.000	0.000	0.000	-1.514	0.574	3.946	-3.127	-5.370
Tr-1 H-1 Sp-2	Kelbadjar	0.000	0.000	0.000	0.000	0.000	0.000	0.000	0.000	1.000	0.000	0.000	0.000	0.000	0.000	-9.004	-9.899	2.745	2.711	-1.662
Tr-1 H-1 Sp-2	Gegham 1	0.000	0.000	0.000	1.000	0.000	0.000	0.000	0.000	0.000	0.000	0.000	0.000	0.000	0.000	-0.892	-11.566	3.687	-4.479	3.494
Tr-1 H-1 Sp-2	Gegham 1	0.000	0.000	0.000	1.000	0.000	0.000	0.000	0.000	0.000	0.000	0.000	0.000	0.000	0.000	0.040	-10.390	3.821	-4.747	2.809

Tr-1 H-1 Sp-2	Arteni	0.000	1.000	0.000	0.000	0.000	0.000	0.000	0.000	0.000	0.000	0.000	0.000	0.000	0.000	-3.720	0.996	4.397	-3.519	-6.788
Tr-1 H-1 Sp-2	Gegham 1	0.000	0.000	0.000	1.000	0.000	0.000	0.000	0.000	0.000	0.000	0.000	0.000	0.000	0.000	-0.879	-10.980	3.084	-4.385	3.444
Tr-1 H-1 Sp-2	Gegham 1	0.000	0.000	0.000	1.000	0.000	0.000	0.000	0.000	0.000	0.000	0.000	0.000	0.000	0.000	-1.354	-11.015	2.799	-4.150	3.062
Tr-1 H-1 Sp-2	Arteni	0.000	1.000	0.000	0.000	0.000	0.000	0.000	0.000	0.000	0.000	0.000	0.000	0.000	0.000	-3.990	1.019	4.067	-3.512	-6.229
Tr-1 H-1 Sp-2	Gegham 1	0.000	0.000	0.000	1.000	0.000	0.000	0.000	0.000	0.000	0.000	0.000	0.000	0.000	0.000	-1.408	-12.163	2.414	-4.405	4.281
Tr-1 H-1 Sp-2	Gegham 1	0.000	0.000	0.000	1.000	0.000	0.000	0.000	0.000	0.000	0.000	0.000	0.000	0.000	0.000	-1.483	-12.701	2.565	-4.546	3.329
Tr-1 H-1 Sp-2	Gegham 1	0.000	0.000	0.000	1.000	0.000	0.000	0.000	0.000	0.000	0.000	0.000	0.000	0.000	0.000	-1.631	-11.027	2.539	-3.646	5.508
Tr-1 H-1 Sp-2	Gegham 1	0.000	0.000	0.000	1.000	0.000	0.000	0.000	0.000	0.000	0.000	0.000	0.000	0.000	0.000	-1.233	-10.893	3.148	-4.240	3.159
Tr-1 H-1 Sp-2	Kelbadjar	0.000	0.000	0.000	0.000	0.000	0.000	0.000	0.000	1.000	0.000	0.000	0.000	0.000	0.000	-10.212	-10.232	0.612	4.960	-1.488
Tr-1 H-1 Sp-2	Gegham 1	0.000	0.000	0.000	1.000	0.000	0.000	0.000	0.000	0.000	0.000	0.000	0.000	0.000	0.000	-0.657	-11.045	3.535	-4.739	2.478
Tr-1 H-1 Sp-2	Gegham 1	0.000	0.000	0.000	1.000	0.000	0.000	0.000	0.000	0.000	0.000	0.000	0.000	0.000	0.000	-0.148	-9.890	3.458	-4.954	6.086
Tr-1 H-1 Sp-2	Kelbadjar	0.000	0.000	0.000	0.000	0.000	0.000	0.000	0.000	1.000	0.000	0.000	0.000	0.000	0.000	-9.419	-10.273	0.354	5.310	-2.255
Tr-1 H-1 Sp-2	Gegham 1	0.000	0.000	0.000	1.000	0.000	0.000	0.000	0.000	0.000	0.000	0.000	0.000	0.000	0.000	0.318	-10.779	3.608	-5.230	7.506
Tr-1 H-1 Sp-2	Gegham 1	0.000	0.000	0.000	1.000	0.000	0.000	0.000	0.000	0.000	0.000	0.000	0.000	0.000	0.000	0.637	-11.930	3.776	-4.249	3.001
Tr-1 H-1 Sp-2	Gegham 1	0.000	0.000	0.000	1.000	0.000	0.000	0.000	0.000	0.000	0.000	0.000	0.000	0.000	0.000	-0.534	-11.050	2.514	-3.799	4.446
Tr-1 H-1 Sp-2	Kelbadjar	0.000	0.000	0.000	0.000	0.000	0.000	0.000	0.000	1.000	0.000	0.000	0.000	0.000	0.000	-8.564	-9.313	-0.139	3.301	-1.625
Pit 1 10-JUN	Gegham 1	0.000	0.000	0.000	1.000	0.000	0.000	0.000	0.000	0.000	0.000	0.000	0.000	0.000	0.000	-1.079	-11.101	2.164	-4.718	3.701
Pit 1 10-JUN	Syunik	0.000	0.000	0.000	0.000	0.000	0.000	0.000	0.000	0.008	0.000	0.000	0.000	0.992	0.000	-6.105	-10.686	-1.225	3.345	-1.717
Pit 1 10-JUN	Gegham 1	0.000	0.000	0.000	1.000	0.000	0.000	0.000	0.000	0.000	0.000	0.000	0.000	0.000	0.000	-1.254	-11.449	3.546	-4.845	3.472
Pit 1 10-JUN	Gegham 1	0.000	0.000	0.000	1.000	0.000	0.000	0.000	0.000	0.000	0.000	0.000	0.000	0.000	0.000	-1.009	-10.594	2.715	-4.414	2.779
Pit 1 10-JUN	Gegham 1	0.000	0.000	0.000	1.000	0.000	0.000	0.000	0.000	0.000	0.000	0.000	0.000	0.000	0.000	-0.983	-10.205	1.370	-3.551	2.990
Pit 1 10-JUN	Gegham 1	0.000	0.000	0.000	1.000	0.000	0.000	0.000	0.000	0.000	0.000	0.000	0.000	0.000	0.000	1.136	-9.494	3.375	-3.930	5.263
Pit 1 10-JUN	Syunik	0.000	0.000	0.000	0.000	0.000	0.000	0.000	0.000	0.000	0.000	0.000	0.000	1.000	0.000	-6.281	-15.199	-4.133	2.851	1.358
Pit 1 10-JUN	Gegham 1	0.000	0.000	0.000	1.000	0.000	0.000	0.000	0.000	0.000	0.000	0.000	0.000	0.000	0.000	-1.522	-11.986	2.993	-4.101	1.801
Pit 1 10-JUN	Gegham 1	0.000	0.000	0.000	1.000	0.000	0.000	0.000	0.000	0.000	0.000	0.000	0.000	0.000	0.000	-1.311	-10.504	2.515	-4.338	3.581
Trench 2 17-	Gegham 1	0.000	0.000	0.000	1.000	0.000	0.000	0.000	0.000	0.000	0.000	0.000	0.000	0.000	0.000	-0.159	-12.049	2.479	-4.694	3.176
Trench 2 17-	Gegham 1	0.000	0.000	0.000	1.000	0.000	0.000	0.000	0.000	0.000	0.000	0.000	0.000	0.000	0.000	-0.648	-11.100	3.224	-4.361	1.787
Trench 2 17-	Gegham 1	0.000	0.000	0.000	1.000	0.000	0.000	0.000	0.000	0.000	0.000	0.000	0.000	0.000	0.000	-0.533	-10.640	3.379	-4.365	2.772
Trench 2 17-	Gegham 1	0.000	0.000	0.000	1.000	0.000	0.000	0.000	0.000	0.000	0.000	0.000	0.000	0.000	0.000	-2.304	-12.895	2.174	-4.169	3.675
Trench 2 17-	Syunik	0.000	0.000	0.000	0.000	0.000	0.000	0.000	0.000	0.000	0.000	0.000	0.000	1.000	0.000	-6.940	-12.453	-1.821	3.402	-1.400
Trench 2 17-	Gegham 1	0.000	0.000	0.000	1.000	0.000	0.000	0.000	0.000	0.000	0.000	0.000	0.000	0.000	0.000	-1.253	-11.203	2.624	-4.461	3.357
Trench 2 17-	Gegham 1	0.000	0.000	0.000	1.000	0.000	0.000	0.000	0.000	0.000	0.000	0.000	0.000	0.000	0.000	-0.504	-10.886	3.706	-4.421	2.683
Trench 2 17-	Gegham 1	0.000	0.000	0.000	1.000	0.000	0.000	0.000	0.000	0.000	0.000	0.000	0.000	0.000	0.000	-0.824	-12.078	2.931	-4.658	4.062
Trench 2 17-	Gegham 1	0.000	0.000	0.000	1.000	0.000	0.000	0.000	0.000	0.000	0.000	0.000	0.000	0.000	0.000	-1.189	-10.187	3.467	-3.933	2.165
Trench 2 17-	Gegham 1	0.000	0.000	0.000	1.000	0.000	0.000	0.000	0.000	0.000	0.000	0.000	0.000	0.000	0.000	-0.951	-12.835	2.893	-4.784	3.982
Trench 2 17-	Gegham 1	0.000	0.000	0.000	1.000	0.000	0.000	0.000	0.000	0.000	0.000	0.000	0.000	0.000	0.000	-0.046	-10.399	3.067	-4.688	2.546
Trench 2 17-	Gegham 1	0.000	0.000	0.000	1.000	0.000	0.000	0.000	0.000	0.000	0.000	0.000	0.000	0.000	0.000	0.038	-12.204	3.215	-4.764	4.676
Trench 2 17-	Gegham 1	0.000	0.000	0.000	1.000	0.000	0.000	0.000	0.000	0.000	0.000	0.000	0.000	0.000	0.000	-0.579	-11.026	3.418	-4.395	2.949
Trench 2 17-	Syunik	0.000	0.000	0.000	0.000	0.000	0.000	0.000	0.000	0.000	0.000	0.000	0.000	1.000	0.000	-6.650	-13.202	-2.749	4.449	-0.833
Trench 2 17-	Gegham 1	0.000	0.000	0.000	1.000	0.000	0.000	0.000	0.000	0.000	0.000	0.000	0.000	0.000	0.000	-1.217	-12.863	3.374	-4.551	2.620
Trench 2 17-	Syunik	0.000	0.000	0.000	0.000	0.000	0.000	0.000	0.000	0.000	0.000	0.000	0.000	1.000	0.000	-6.487	-12.382	-2.831	2.828	-0.584
Trench 2 17-	Gegham 1	0.000	0.000	0.000	1.000	0.000	0.000	0.000	0.000	0.000	0.000	0.000	0.000	0.000	0.000	-0.140	-11.279	2.993	-4.287	3.693
Trench 2 17-	Arteni	0.000	1.000	0.000	0.000	0.000	0.000	0.000	0.000	0.000	0.000	0.000	0.000	0.000	0.000	-1.676	1.253	3.700	-2.710	-5.261
Trench 2 17-	Gegham 1	0.000	0.000	0.000	1.000	0.000	0.000	0.000	0.000	0.000	0.000	0.000	0.000	0.000	0.000	-0.513	-11.763	3.137	-4.713	5.294
Trench 2 17-	Gegham 1	0.000	0.000	0.000	1.000	0.000	0.000	0.000	0.000	0.000	0.000	0.000	0.000	0.000	0.000	-0.416	-10.985	3.303	-4.780	5.667
Trench 2 17-	Gegham 1	0.000	0.000	0.000	1.000	0.000	0.000	0.000	0.000	0.000	0.000	0.000	0.000	0.000	0.000	-1.119	-10.371	3.259	-4.149	2.250
Trench 2 17-	Syunik	0.000	0.000	0.000	0.000	0.000	0.000	0.000	0.000	0.000	0.000	0.000	0.000	1.000	0.000	-6.770	-12.392	-1.528	3.504	-1.551
Trench 2 17-	Gegham 1	0.000	0.000	0.000	1.000	0.000	0.000	0.000	0.000	0.000	0.000	0.000	0.000	0.000	0.000	-1.038	-12.013	3.279	-4.052	2.748
Trench 2 17-	Arteni	0.000	0.974	0.000	0.000	0.026	0.000	0.000	0.000	0.000	0.000	0.000	0.000	0.000	0.000	-1.202	3.682	4.263	-3.260	-6.467
Trench 2 17-	Arteni	0.000	1.000	0.000	0.000	0.000	0.000	0.000	0.000	0.000	0.000	0.000	0.000	0.000	0.000	-2.408	0.233	4.100	-3.476	-6.739
Trench 2 17-	Gegham 1	0.000	0.000	0.000	1.000	0.000	0.000	0.000	0.000	0.000	0.000	0.000	0.000	0.000	0.000	-1.434	-11.000	3.417	-3.814	3.029
Trench 2 17-	Gegham 1	0.000	0.000	0.000	1.000	0.000	0.000	0.000	0.000	0.000	0.000	0.000	0.000	0.000	0.000	-0.010	-11.509	2.402	-4.733	4.890
Trench 2 17-	Gegham 1	0.000	0.000	0.000	1.000	0.000	0.000	0.000	0.000	0.000	0.000	0.000	0.000	0.000	0.000	-1.167	-11.514	3.678	-4.199	2.563
Trench 2 17-	Gegham 1	0.000	0.000	0.000	1.000	0.000	0.000	0.000	0.000	0.000	0.000	0.000	0.000	0.000	0.000	-0.666	-7.577	2.473	-3.549	4.281
Trench 2 17-	Syunik	0.000	0.000	0.000	0.000	0.000	0.000	0.000	0.000	0.000	0.000	0.000	0.000	1.000	0.000	-6.196	-12.924	-1.377	4.024	-1.235
Trench 2 17-	Gegham 1	0.000	0.000	0.000	1.000	0.000	0.000	0.000	0.000	0.000	0.000	0.000	0.000	0.000	0.000	0.791	-7.978	2.623	-4.132	3.455
Trench 2 17-	Gegham 1	0.000	0.000	0.000	1.000	0.000	0.000	0.000	0.000	0.000	0.000	0.000	0.000	0.000	0.000	0.240	-11.710	2.725	-4.590	3.720
Trench 2 17-	Syunik	0.000	0.000	0.000	0.000	0.000	0.000	0.000	0.000	0.000	0.000	0.000	0.000	1.000	0.000	-6.038	-13.101	-2.235	4.069	-0.951
Trench 2 17-	Gegham 1	0.000	0.000	0.000	1.000	0.000	0.000	0.000	0.000	0.000	0.000	0.000	0.000	0.000	0.000	-0.660	-10.906	3.289	-4.604	4.473
Trench 2 17-	Kelbadjar	0.000	0.000	0.000	0.000	0.000	0.000	0.000	0.000	1.000	0.000	0.000	0.000	0.000	0.000	-9.538	-9.318	-0.807	6.176	-2.393
Trench 2 17-	Gegham 1	0.000	0.000	0.000	1.000	0.000	0.000	0.000	0.000	0.000	0.000	0.000	0.000	0.000	0.000	-0.979	-12.918	2.945	-4.594	5.254
Trench 2 17-	Gegham 1	0.000	0.000	0.000	1.000	0.000	0.000	0.000	0.000	0.000	0.000	0.000	0.000	0.000	0.000	-1.538	-11.387	2.854	-4.396	4.633
Trench 2 17-	Gegham 1	0.000	0.000	0.000	1.000	0.000	0.000	0.000	0.000	0.000	0.000	0.000	0.000	0.000	0.000	-0.981	-12.065	3.137	-4.560	4.652
Trench 2 17-	Gegham 1	0.000	0.000	0.000	1.000	0.000	0.000	0.000	0.000	0.000	0.000	0.000	0.000	0.000	0.000	-0.944	-10.249	3.446	-4.797	4.668
Trench 2 17-	Gegham 1	0.000	0.000	0.000	1.000	0.000	0.000	0.000	0.000	0.000	0.000	0.000	0.000	0.000	0.000	-1.298	-11.501	3.263	-4.515	7.047
Trench 2 17-	Syunik	0.000	0.000	0.000	0.000	0.000	0.000	0.000	0.000	0.000	0.000	0.000	0.000	1.000	0.000	-10.592	-9.516	-2.515	5.552	-2.248
Trench 2 17-	Gegham 1	0.000	0.000	0.000	1.000	0.000	0.000	0.000	0.000	0.000	0.000	0.000	0.000	0.000	0.000	0.053	-10.193	3.715	-4.615	3.808

Trench 2 17-	Gegham 1	0.000	0.000	0.000	1.000	0.000	0.000	0.000	0.000	0.000	0.000	0.000	0.000	0.000	0.000	-0.036	-9.712	4.165	-4.807	3.081
Trench 2 17-	Gegham 1	0.000	0.000	0.000	1.000	0.000	0.000	0.000	0.000	0.000	0.000	0.000	0.000	0.000	0.000	2.327	-11.470	3.596	-4.658	7.727
Tr-2 Hor-3 Sp	Gegham 1	0.000	0.000	0.000	1.000	0.000	0.000	0.000	0.000	0.000	0.000	0.000	0.000	0.000	0.000	-1.993	-11.383	2.181	-3.312	2.727
Tr-2 Hor-3 Sp	Kelbadjar	0.000	0.000	0.000	0.000	0.000	0.000	0.000	0.000	1.000	0.000	0.000	0.000	0.000	0.000	-8.415	-6.854	-2.054	3.609	0.420
Tr-2 Hor-3 Sp	Gegham 1	0.000	0.000	0.000	1.000	0.000	0.000	0.000	0.000	0.000	0.000	0.000	0.000	0.000	0.000	0.759	-11.267	3.684	-4.984	3.161
Tr-2 Hor-3 Sp	Kelbadjar	0.000	0.000	0.000	0.000	0.000	0.000	0.000	0.000	1.000	0.000	0.000	0.000	0.000	0.000	-9.920	-9.437	0.423	4.698	-2.224
Tr-2 Hor-3 Sp	Gegham 1	0.000	0.000	0.000	1.000	0.000	0.000	0.000	0.000	0.000	0.000	0.000	0.000	0.000	0.000	0.349	-11.905	3.321	-5.071	7.276
Tr-2 Hor-3 Sp	Gegham 1	0.000	0.000	0.000	1.000	0.000	0.000	0.000	0.000	0.000	0.000	0.000	0.000	0.000	0.000	-0.574	-6.927	4.688	-4.546	4.207
Tr-2 Hor-3 Sp	Gegham 1	0.000	0.000	0.000	1.000	0.000	0.000	0.000	0.000	0.000	0.000	0.000	0.000	0.000	0.000	-1.333	-11.404	3.321	-4.796	8.053
Trench-2 Hor	Kelbadjar	0.000	0.000	0.000	0.000	0.000	0.000	0.000	0.000	1.000	0.000	0.000	0.000	0.000	0.000	-10.542	-6.983	-4.026	3.896	3.332
Trench-2 Hor	Gegham 1	0.000	0.000	0.000	1.000	0.000	0.000	0.000	0.000	0.000	0.000	0.000	0.000	0.000	0.000	-0.982	-10.842	3.482	-4.813	3.177
Trench-2 Hor	Kelbadjar	0.000	0.000	0.000	0.000	0.000	0.000	0.000	0.000	1.000	0.000	0.000	0.000	0.000	0.000	-9.399	-9.161	0.100	3.069	-1.032
Trench-2 Hor	Gegham 1	0.000	0.000	0.000	1.000	0.000	0.000	0.000	0.000	0.000	0.000	0.000	0.000	0.000	0.000	-0.284	-10.411	2.912	-4.690	4.598
Trench-2 Hor	Gegham 1	0.000	0.000	0.000	1.000	0.000	0.000	0.000	0.000	0.000	0.000	0.000	0.000	0.000	0.000	-1.108	-11.494	3.416	-4.301	2.145
Trench-2 Hor	Gegham 1	0.000	0.000	0.000	1.000	0.000	0.000	0.000	0.000	0.000	0.000	0.000	0.000	0.000	0.000	-0.726	-11.117	3.714	-4.641	4.466
Trench-2 Hor	Gegham 1	0.000	0.000	0.000	1.000	0.000	0.000	0.000	0.000	0.000	0.000	0.000	0.000	0.000	0.000	-1.661	-11.215	2.988	-4.085	4.023
Trench-2 Hor	Gegham 1	0.000	0.000	0.000	1.000	0.000	0.000	0.000	0.000	0.000	0.000	0.000	0.000	0.000	0.000	-1.109	-12.304	2.936	-4.448	3.977
Trench-2 Hor	Gegham 1	0.000	0.000	0.000	1.000	0.000	0.000	0.000	0.000	0.000	0.000	0.000	0.000	0.000	0.000	-0.818	-10.323	3.162	-4.541	2.502
Trench-2 Hor	Gegham 1	0.000	0.000	0.000	1.000	0.000	0.000	0.000	0.000	0.000	0.000	0.000	0.000	0.000	0.000	-1.233	-9.225	2.929	-3.400	1.338
Trench-2 Hor	Gegham 1	0.000	0.000	0.000	1.000	0.000	0.000	0.000	0.000	0.000	0.000	0.000	0.000	0.000	0.000	-0.446	-10.698	2.897	-4.664	3.595
Trench-2 Hor	Gegham 1	0.000	0.000	0.000	1.000	0.000	0.000	0.000	0.000	0.000	0.000	0.000	0.000	0.000	0.000	2.005	-10.023	2.141	-3.544	2.867
Trench-2 Hor	Gegham 1	0.000	0.000	0.000	1.000	0.000	0.000	0.000	0.000	0.000	0.000	0.000	0.000	0.000	0.000	-0.839	-10.312	3.844	-4.394	1.893
Trench-2 Hor	Gegham 2	0.000	0.000	0.000	0.000	1.000	0.000	0.000	0.000	0.000	0.000	0.000	0.000	0.000	0.000	-0.703	4.336	5.776	-2.974	-7.298
Trench-2 Hor	Gegham 1	0.000	0.000	0.000	1.000	0.000	0.000	0.000	0.000	0.000	0.000	0.000	0.000	0.000	0.000	-0.927	-10.589	3.454	-4.440	4.080
Trench-2 Hor	Gegham 1	0.000	0.000	0.000	1.000	0.000	0.000	0.000	0.000	0.000	0.000	0.000	0.000	0.000	0.000	-0.331	-11.757	3.344	-4.494	3.191
Trench-2 Hor	Gegham 1	0.000	0.000	0.000	1.000	0.000	0.000	0.000	0.000	0.000	0.000	0.000	0.000	0.000	0.000	-1.151	-11.427	2.791	-4.199	4.377
Trench-2 Hor	Arteni	0.000	1.000	0.000	0.000	0.000	0.000	0.000	0.000	0.000	0.000	0.000	0.000	0.000	0.000	-1.487	2.974	3.252	-3.543	-5.512
Trench-2 Hor	Gegham 1	0.000	0.000	0.000	1.000	0.000	0.000	0.000	0.000	0.000	0.000	0.000	0.000	0.000	0.000	-0.661	-12.496	2.608	-3.630	2.682
Trench-2 Hor	Gegham 1	0.000	0.000	0.000	1.000	0.000	0.000	0.000	0.000	0.000	0.000	0.000	0.000	0.000	0.000	-1.110	-10.619	2.911	-4.628	3.884
Trench-2 Hor	Gegham 1	0.000	0.000	0.000	1.000	0.000	0.000	0.000	0.000	0.000	0.000	0.000	0.000	0.000	0.000	1.952	-11.082	3.045	-4.366	3.227
Trench-2 Hor	Gegham 1	0.000	0.000	0.000	1.000	0.000	0.000	0.000	0.000	0.000	0.000	0.000	0.000	0.000	0.000	0.927	-9.560	3.530	-4.102	3.792
Trench-2 Hor	Gegham 1	0.000	0.000	0.000	1.000	0.000	0.000	0.000	0.000	0.000	0.000	0.000	0.000	0.000	0.000	-0.613	-10.870	2.687	-4.574	3.429
Trench-2 Hor	Gegham 1	0.000	0.000	0.000	1.000	0.000	0.000	0.000	0.000	0.000	0.000	0.000	0.000	0.000	0.000	-0.979	-11.157	3.408	-4.600	5.134
Trench-2 Hor	Gegham 1	0.000	0.000	0.000	1.000	0.000	0.000	0.000	0.000	0.000	0.000	0.000	0.000	0.000	0.000	-0.631	-10.539	2.869	-4.710	3.951
Trench-2 Hor	Arteni	0.000	1.000	0.000	0.000	0.000	0.000	0.000	0.000	0.000	0.000	0.000	0.000	0.000	0.000	-5.213	1.364	5.656	-3.719	-7.496
Trench-2 Hor	Gegham 1	0.000	0.000	0.000	1.000	0.000	0.000	0.000	0.000	0.000	0.000	0.000	0.000	0.000	0.000	-0.537	-10.905	2.613	-4.349	4.010
Trench-2 Hor	Arteni	0.000	1.000	0.000	0.000	0.000	0.000	0.000	0.000	0.000	0.000	0.000	0.000	0.000	0.000	-3.341	1.792	4.556	-3.112	-5.324
Trench-2 Hor	Gegham 1	0.000	0.000	0.000	1.000	0.000	0.000	0.000	0.000	0.000	0.000	0.000	0.000	0.000	0.000	-0.297	-11.461	3.433	-4.575	5.672
Trench-2 Hor	Arteni	0.000	1.000	0.000	0.000	0.000	0.000	0.000	0.000	0.000	0.000	0.000	0.000	0.000	0.000	-1.905	1.722	3.668	-3.463	-5.976
Trench-2 Hor	Gegham 1	0.000	0.000	0.000	1.000	0.000	0.000	0.000	0.000	0.000	0.000	0.000	0.000	0.000	0.000	-2.533	-8.476	5.060	-5.122	10.649
Trench-2 Hor	Gegham 1	0.000	0.000	0.000	1.000	0.000	0.000	0.000	0.000	0.000	0.000	0.000	0.000	0.000	0.000	-1.233	-11.548	2.372	-4.426	5.570
Trench-2 Hor	Gegham 1	0.000	0.000	0.000	1.000	0.000	0.000	0.000	0.000	0.000	0.000	0.000	0.000	0.000	0.000	0.027	-10.382	3.299	-4.698	3.245
Trench 2 Top	Gegham 1	0.000	0.000	0.000	1.000	0.000	0.000	0.000	0.000	0.000	0.000	0.000	0.000	0.000	0.000	-0.824	-11.540	2.455	-4.576	4.536
Trench 2 Top	Gegham 1	0.000	0.000	0.000	1.000	0.000	0.000	0.000	0.000	0.000	0.000	0.000	0.000	0.000	0.000	-1.809	-13.601	2.228	-4.468	3.644
Trench 2 Top	Gegham 1	0.000	0.000	0.000	1.000	0.000	0.000	0.000	0.000	0.000	0.000	0.000	0.000	0.000	0.000	-1.430	-10.234	4.175	-4.832	2.305
Trench 2 Top	Gegham 1	0.000	0.000	0.000	1.000	0.000	0.000	0.000	0.000	0.000	0.000	0.000	0.000	0.000	0.000	-0.146	-10.619	3.097	-4.656	3.436
Trench 2 Top	Gegham 1	0.000	0.000	0.000	1.000	0.000	0.000	0.000	0.000	0.000	0.000	0.000	0.000	0.000	0.000	-1.108	-12.581	1.913	-4.066	3.648
Trench 2 Top	Gegham 1	0.000	0.000	0.000	1.000	0.000	0.000	0.000	0.000	0.000	0.000	0.000	0.000	0.000	0.000	-1.262	-9.906	2.691	-4.671	3.652
Trench 2 Top	Gegham 1	0.000	0.000	0.000	1.000	0.000	0.000	0.000	0.000	0.000	0.000	0.000	0.000	0.000	0.000	-1.192	-11.583	3.047	-4.352	4.024
Trench 2 Top	Gegham 1	0.000	0.000	0.000	1.000	0.000	0.000	0.000	0.000	0.000	0.000	0.000	0.000	0.000	0.000	-1.988	-11.764	1.972	-4.328	4.162
Trench 2 Top	Gegham 1	0.000	0.000	0.000	1.000	0.000	0.000	0.000	0.000	0.000	0.000	0.000	0.000	0.000	0.000	-1.132	-10.596	2.886	-4.030	3.318
Trench 2 Top	Gegham 1	0.000	0.000	0.000	1.000	0.000	0.000	0.000	0.000	0.000	0.000	0.000	0.000	0.000	0.000	-0.842	-11.260	4.100	-4.063	1.935
Trench 2 Top	Gegham 1	0.000	0.000	0.000	1.000	0.000	0.000	0.000	0.000	0.000	0.000	0.000	0.000	0.000	0.000	-1.090	-10.230	2.800	-2.929	2.634
Trench 2 Top	Kelbadjar	0.000	0.000	0.000	0.000	0.000	0.000	0.000	0.000	1.000	0.000	0.000	0.000	0.000	0.000	-9.097	-8.376	0.585	2.465	-1.063
Trench 2 Top	Gegham 1	0.000	0.000	0.000	1.000	0.000	0.000	0.000	0.000	0.000	0.000	0.000	0.000	0.000	0.000	-0.840	-10.846	3.773	-4.684	2.907
Trench 2 Top	Kelbadjar	0.000	0.000	0.000	0.000	0.000	0.000	0.000	0.000	1.000	0.000	0.000	0.000	0.000	0.000	-8.317	-8.355	2.669	2.791	-1.398
Trench 2 Top	Gegham 1	0.000	0.000	0.000	1.000	0.000	0.000	0.000	0.000	0.000	0.000	0.000	0.000	0.000	0.000	-0.865	-10.031	3.320	-4.647	3.094
Trench 2 Top	Syunik	0.000	0.000	0.000	0.000	0.000	0.000	0.000	0.000	0.000	0.000	0.000	0.000	1.000	0.000	-6.386	-12.033	-1.106	4.043	-1.747
Trench 2 Top	Kelbadjar	0.000	0.000	0.000	0.000	0.000	0.000	0.000	0.000	1.000	0.000	0.000	0.000	0.000	0.000	-9.594	-9.311	2.364	3.817	-1.943
Trench 2 Top	Gegham 1	0.000	0.000	0.000	1.000	0.000	0.000	0.000	0.000	0.000	0.000	0.000	0.000	0.000	0.000	-1.790	-12.584	2.728	-4.094	2.602
Trench 2 Top	Gegham 1	0.000	0.000	0.000	1.000	0.000	0.000	0.000	0.000	0.000	0.000	0.000	0.000	0.000	0.000	-2.225	-12.955	2.923	-4.319	2.702
Trench 2 Top	Kelbadjar	0.000	0.000	0.000	0.000	0.000	0.000	0.000	0.000	1.000	0.000	0.000	0.000	0.000	0.000	-9.367	-9.999	2.319	4.082	-1.378
Trench 2 Top	Gegham 1	0.000	0.000	0.000	1.000	0.000	0.000	0.000	0.000	0.000	0.000	0.000	0.000	0.000	0.000	-0.921	-11.444	3.753	-4.099	2.730
Trench 2 Top	Gegham 1	0.000	0.000	0.000	1.000	0.000	0.000	0.000	0.000	0.000	0.000	0.000	0.000	0.000	0.000	-1.121	-11.133	2.710	-3.870	2.477
Trench 2 Top	Kelbadjar	0.000	0.000	0.000	0.000	0.000	0.000	0.000	0.000	1.000	0.000	0.000	0.000	0.000	0.000	-9.335	-9.722	-0.224	4.904	-1.845
Trench 2 Top	Kelbadjar	0.000	0.000	0.000	0.000	0.000	0.000	0.000	0.000	1.000	0.000	0.000	0.000	0.000	0.000	-9.579	-9.356	0.995	4.857	-1.854
Trench 2 Top	Gegham 1	0.000	0.000	0.000	1.000	0.000	0.000	0.000	0.000	0.000	0.000	0.000	0.000	0.000	0.000	0.156	-10.261	0.871	-3.344	3.377

Trench 2 Top	Gegham 1	0.000	0.000	0.000	1.000	0.000	0.000	0.000	0.000	0.000	0.000	0.000	0.000	0.000	0.000	-1.323	-10.509	2.711	-3.527	1.261
Trench 2 Top	Gegham 1	0.000	0.000	0.000	1.000	0.000	0.000	0.000	0.000	0.000	0.000	0.000	0.000	0.000	0.000	-1.442	-11.807	2.617	-4.428	4.505
Trench 2 Top	Gegham 1	0.000	0.000	0.000	1.000	0.000	0.000	0.000	0.000	0.000	0.000	0.000	0.000	0.000	0.000	-0.896	-11.258	3.612	-4.439	3.806
Trench 2 Top	Gegham 1	0.000	0.000	0.000	1.000	0.000	0.000	0.000	0.000	0.000	0.000	0.000	0.000	0.000	0.000	0.658	-9.634	3.480	-4.554	3.573
Trench 2 Top	Gegham 1	0.000	0.000	0.000	1.000	0.000	0.000	0.000	0.000	0.000	0.000	0.000	0.000	0.000	0.000	2.086	-9.688	2.051	-3.859	4.287
Trench 2 Top	Gegham 1	0.000	0.000	0.000	1.000	0.000	0.000	0.000	0.000	0.000	0.000	0.000	0.000	0.000	0.000	-0.878	-9.096	4.137	-4.150	1.684
Trench 2 Top	Gegham 1	0.000	0.000	0.000	1.000	0.000	0.000	0.000	0.000	0.000	0.000	0.000	0.000	0.000	0.000	0.385	-8.748	4.502	-4.749	2.266
Trench 2 Top	Gegham 1	0.000	0.000	0.000	1.000	0.000	0.000	0.000	0.000	0.000	0.000	0.000	0.000	0.000	0.000	-0.128	-9.981	4.673	-4.666	3.046
Trench 2 Top	Kelbadjar	0.000	0.000	0.000	0.000	0.000	0.000	0.000	0.000	1.000	0.000	0.000	0.000	0.000	0.000	-8.881	-9.581	0.869	4.522	-1.288
Trench 2 Top	Kelbadjar	0.000	0.000	0.000	0.000	0.000	0.000	0.000	0.000	1.000	0.000	0.000	0.000	0.000	0.000	-8.932	-9.401	1.022	3.703	-1.746
Trench 2 Top	Gegham 1	0.000	0.000	0.000	1.000	0.000	0.000	0.000	0.000	0.000	0.000	0.000	0.000	0.000	0.000	-1.026	-10.178	3.218	-4.492	2.608
Trench 2 Top	Gegham 1	0.000	0.000	0.000	1.000	0.000	0.000	0.000	0.000	0.000	0.000	0.000	0.000	0.000	0.000	-0.492	-8.582	3.356	-4.288	3.508
Trench 2 Top	Gegham 1	0.000	0.000	0.000	1.000	0.000	0.000	0.000	0.000	0.000	0.000	0.000	0.000	0.000	0.000	-0.291	-10.942	2.935	-4.875	4.556
Trench 2 Top	Gegham 1	0.000	0.000	0.000	1.000	0.000	0.000	0.000	0.000	0.000	0.000	0.000	0.000	0.000	0.000	-0.443	-11.752	2.744	-4.499	3.281
Trench 2 Top	Kelbadjar	0.000	0.000	0.000	0.000	0.000	0.000	0.000	0.000	1.000	0.000	0.000	0.000	0.000	0.000	-8.954	-7.963	0.696	3.486	-1.957
Trench 2 Top	Kelbadjar	0.000	0.000	0.000	0.000	0.000	0.000	0.000	0.000	0.997	0.000	0.000	0.000	0.003	0.000	-10.233	-9.438	-1.607	4.566	-1.642
Trench 2 Top	Gegham 1	0.000	0.000	0.000	1.000	0.000	0.000	0.000	0.000	0.000	0.000	0.000	0.000	0.000	0.000	-0.411	-11.927	3.055	-4.767	3.432
Trench 2 Top	Gegham 1	0.000	0.000	0.000	1.000	0.000	0.000	0.000	0.000	0.000	0.000	0.000	0.000	0.000	0.000	-1.039	-11.645	2.963	-4.696	4.694
Trench 2 Top	Gegham 1	0.000	0.000	0.000	1.000	0.000	0.000	0.000	0.000	0.000	0.000	0.000	0.000	0.000	0.000	-0.568	-10.488	3.073	-4.648	4.798
Trench 2 Top	Kelbadjar	0.000	0.000	0.000	0.000	0.000	0.000	0.000	0.000	1.000	0.000	0.000	0.000	0.000	0.000	-8.225	-8.702	-0.658	4.777	-1.893
Trench 2 Top	Kelbadjar	0.000	0.000	0.000	0.000	0.000	0.000	0.000	0.000	1.000	0.000	0.000	0.000	0.000	0.000	-9.019	-8.272	1.282	4.706	-1.460
Trench 2 Top	Gegham 1	0.000	0.000	0.000	1.000	0.000	0.000	0.000	0.000	0.000	0.000	0.000	0.000	0.000	0.000	-0.934	-11.832	2.607	-4.755	4.970
Trench 2 Top	Kelbadjar	0.000	0.000	0.000	0.000	0.000	0.000	0.000	0.000	1.000	0.000	0.000	0.000	0.000	0.000	-9.578	-10.173	-0.064	3.965	-1.490
Trench 2 Top	Gegham 1	0.000	0.000	0.000	1.000	0.000	0.000	0.000	0.000	0.000	0.000	0.000	0.000	0.000	0.000	-0.481	-11.130	3.170	-4.650	3.994
Trench 2 Top	Gegham 1	0.000	0.000	0.000	1.000	0.000	0.000	0.000	0.000	0.000	0.000	0.000	0.000	0.000	0.000	-0.849	-10.994	2.990	-4.467	5.947
Trench 2 Top	Gegham 1	0.000	0.000	0.000	1.000	0.000	0.000	0.000	0.000	0.000	0.000	0.000	0.000	0.000	0.000	-1.657	-11.749	2.782	-4.643	5.023
Trench 2 Top	Gegham 1	0.000	0.000	0.000	1.000	0.000	0.000	0.000	0.000	0.000	0.000	0.000	0.000	0.000	0.000	-1.003	-11.088	2.789	-4.762	4.214
Trench 2 Top	Gegham 1	0.000	0.000	0.000	1.000	0.000	0.000	0.000	0.000	0.000	0.000	0.000	0.000	0.000	0.000	-0.817	-11.050	3.222	-4.798	2.652
Trench 2 Top	Kelbadjar	0.000	0.000	0.000	0.000	0.000	0.000	0.000	0.000	1.000	0.000	0.000	0.000	0.000	0.000	-9.460	-11.156	0.534	5.538	-1.526
Trench 2 Top	Gegham 1	0.000	0.000	0.000	1.000	0.000	0.000	0.000	0.000	0.000	0.000	0.000	0.000	0.000	0.000	0.369	-11.117	2.307	-4.523	4.245
Trench 2 Top	Gegham 1	0.000	0.000	0.000	1.000	0.000	0.000	0.000	0.000	0.000	0.000	0.000	0.000	0.000	0.000	-1.007	-12.652	3.407	-4.707	3.379
Trench 2 Top	Gegham 1	0.000	0.000	0.000	1.000	0.000	0.000	0.000	0.000	0.000	0.000	0.000	0.000	0.000	0.000	-0.603	-10.951	3.018	-4.547	3.401
Trench 2 Top	Kelbadjar	0.000	0.000	0.000	0.000	0.000	0.000	0.000	0.000	1.000	0.000	0.000	0.000	0.000	0.000	-10.516	-9.996	-0.444	5.104	-1.095
Trench 2 Top	Syunik	0.000	0.000	0.000	0.000	0.000	0.000	0.000	0.000	0.000	0.000	0.000	0.000	1.000	0.000	-6.449	-13.448	-2.517	4.248	-1.188
Trench 2 Top	Gegham 1	0.000	0.000	0.000	1.000	0.000	0.000	0.000	0.000	0.000	0.000	0.000	0.000	0.000	0.000	-0.556	-10.616	3.191	-4.265	2.123
Tr-2 H-2 Sp-2	Gegham 1	0.000	0.000	0.000	1.000	0.000	0.000	0.000	0.000	0.000	0.000	0.000	0.000	0.000	0.000	-1.203	-11.490	2.703	-4.654	3.920
Tr-2 H-2 Sp-2	Gegham 1	0.000	0.000	0.000	1.000	0.000	0.000	0.000	0.000	0.000	0.000	0.000	0.000	0.000	0.000	-0.285	-10.656	3.912	-4.493	1.752
Tr-2 H-2 Sp-2	Kelbadjar	0.000	0.000	0.000	0.000	0.000	0.000	0.000	0.000	1.000	0.000	0.000	0.000	0.000	0.000	-9.315	-9.087	0.966	4.437	-1.354
Tr-2 H-2 Sp-2	Kelbadjar	0.000	0.000	0.000	0.000	0.000	0.000	0.000	0.000	1.000	0.000	0.000	0.000	0.000	0.000	-8.803	-9.733	1.090	3.473	-0.552
Tr-2 H-2 Sp-2	Syunik	0.000	0.000	0.000	0.000	0.000	0.000	0.000	0.000	0.000	0.000	0.000	0.000	1.000	0.000	-7.007	-12.743	-0.731	2.945	-1.723
Tr-2 H-2 Sp-2	Kelbadjar	0.000	0.000	0.000	0.000	0.000	0.000	0.000	0.000	1.000	0.000	0.000	0.000	0.000	0.000	-9.649	-10.269	2.357	4.664	-1.129
Tr-2 H-2 Sp-2	Kelbadjar	0.000	0.000	0.000	0.000	0.000	0.000	0.000	0.000	1.000	0.000	0.000	0.000	0.000	0.000	-8.242	-8.778	1.157	5.303	-1.579
Tr-2 H-2 Sp-2	Gegham 1	0.000	0.000	0.000	1.000	0.000	0.000	0.000	0.000	0.000	0.000	0.000	0.000	0.000	0.000	-1.384	-10.996	3.387	-4.258	2.686
Tr-2 H-2 Sp-2	Kelbadjar	0.000	0.000	0.000	0.000	0.000	0.000	0.000	0.000	1.000	0.000	0.000	0.000	0.000	0.000	-9.044	-10.465	0.982	2.865	-0.922
Tr-2 H-2 Sp-2	Gegham 1	0.000	0.000	0.000	1.000	0.000	0.000	0.000	0.000	0.000	0.000	0.000	0.000	0.000	0.000	-1.113	-11.876	2.357	-4.254	2.965
Tr-2 H-2 Sp-2	Kelbadjar	0.000	0.000	0.000	0.000	0.000	0.000	0.000	0.000	1.000	0.000	0.000	0.000	0.000	0.000	-9.601	-9.550	0.156	3.389	-1.425
Tr-2 H-2 Sp-2	Kelbadjar	0.000	0.000	0.000	0.000	0.000	0.000	0.000	0.000	1.000	0.000	0.000	0.000	0.000	0.000	-9.658	-9.678	0.543	4.498	-1.761
Tr-2 H-2 Sp-2	Kelbadjar	0.000	0.000	0.000	0.000	0.000	0.000	0.000	0.000	1.000	0.000	0.000	0.000	0.000	0.000	-9.602	-9.669	-0.605	4.067	-1.756
Tr-2 H-2 Sp-2	Kelbadjar	0.000	0.000	0.000	0.000	0.000	0.000	0.000	0.000	1.000	0.000	0.000	0.000	0.000	0.000	-9.116	-8.812	1.040	2.486	-1.381
Tr-2 H-2 Sp-2	Gegham 1	0.000	0.000	0.000	1.000	0.000	0.000	0.000	0.000	0.000	0.000	0.000	0.000	0.000	0.000	-0.619	-10.756	3.713	-4.941	3.827
Tr-2 H-2 Sp-2	Gegham 1	0.000	0.000	0.000	1.000	0.000	0.000	0.000	0.000	0.000	0.000	0.000	0.000	0.000	0.000	0.661	-9.367	3.266	-4.363	1.968
Tr-2 H-2 Sp-2	Kelbadjar	0.000	0.000	0.000	0.000	0.000	0.000	0.000	0.000	1.000	0.000	0.000	0.000	0.000	0.000	-9.572	-10.093	0.956	4.993	-1.310
Tr-2 H-2 Sp-2	Gegham 1	0.000	0.000	0.000	1.000	0.000	0.000	0.000	0.000	0.000	0.000	0.000	0.000	0.000	0.000	-1.185	-10.437	3.304	-4.031	2.678
Tr-2 H-2 Sp-2	Gegham 1	0.000	0.000	0.000	1.000	0.000	0.000	0.000	0.000	0.000	0.000	0.000	0.000	0.000	0.000	0.218	-11.505	3.097	-4.360	3.426
Tr-2 H-2 Sp-2	Gegham 1	0.000	0.000	0.000	1.000	0.000	0.000	0.000	0.000	0.000	0.000	0.000	0.000	0.000	0.000	-1.666	-11.932	2.206	-4.231	3.424
Tr-2 H-2 Sp-2	Gegham 1	0.000	0.000	0.000	1.000	0.000	0.000	0.000	0.000	0.000	0.000	0.000	0.000	0.000	0.000	-1.200	-10.850	2.399	-3.831	3.580
Tr-2 H-2 Sp-2	Kelbadjar	0.000	0.000	0.000	0.000	0.000	0.000	0.000	0.000	1.000	0.000	0.000	0.000	0.000	0.000	-9.236	-9.439	0.406	2.779	-0.981
Tr-2 H-2 Sp-2	Kelbadjar	0.000	0.000	0.000	0.000	0.000	0.000	0.000	0.000	1.000	0.000	0.000	0.000	0.000	0.000	-9.408	-9.653	0.932	4.361	-1.757
Tr-2 H-2 Sp-2	Gegham 1	0.000	0.000	0.000	1.000	0.000	0.000	0.000	0.000	0.000	0.000	0.000	0.000	0.000	0.000	-1.000	-10.999	2.557	-3.993	2.181
Tr-2 H-2 Sp-2	Gegham 1	0.000	0.000	0.000	1.000	0.000	0.000	0.000	0.000	0.000	0.000	0.000	0.000	0.000	0.000	-0.355	-11.809	2.274	-4.456	4.223
Tr-2 H-2 Sp-2	Kelbadjar	0.000	0.000	0.000	0.000	0.000	0.000	0.000	0.000	1.000	0.000	0.000	0.000	0.000	0.000	-9.070	-10.348	0.205	3.148	-1.157
Tr-2 H-2 Sp-2	Gegham 1	0.000	0.000	0.000	1.000	0.000	0.000	0.000	0.000	0.000	0.000	0.000	0.000	0.000	0.000	-2.783	-10.902	2.985	-4.583	4.142
Tr-2 H-2 Sp-2	Gegham 1	0.000	0.000	0.000	1.000	0.000	0.000	0.000	0.000	0.000	0.000	0.000	0.000	0.000	0.000	0.638	-9.522	4.288	-4.804	4.399
Tr-2 H-2 Sp-2	Gegham 1	0.000	0.000	0.000	1.000	0.000	0.000	0.000	0.000	0.000	0.000	0.000	0.000	0.000	0.000	0.312	-10.490	2.725	-4.476	4.304
Tr-2 H-2 Sp-2	Gegham 1	0.000	0.000	0.000	1.000	0.000	0.000	0.000	0.000	0.000	0.000	0.000	0.000	0.000	0.000	4.101	-6.872	4.381	-4.191	6.248
Tr-2 H-2 Sp-2	Gegham 1	0.000	0.000	0.000	1.000	0.000	0.000	0.000	0.000	0.000	0.000	0.000	0.000	0.000	0.000	-0.371	-11.397	2.461	-4.203	4.390
Tr-2 H-2 Sp-2	Gegham 1	0.000	0.000	0.000	1.000	0.000	0.000	0.000	0.000	0.000	0.000	0.000	0.000	0.000	0.000	-1.499	-9.170	3.687	-3.273	0.863

Tr-2 H-2 Sp-	Kelbadjar	0.000	0.000	0.000	0.000	0.000	0.000	0.000	0.000	1.000	0.000	0.000	0.000	0.000	0.000	-8.973	-9.558	-0.118	3.792	-1.721
Tr-2 H-2 Sp-	Gegham 1	0.000	0.000	0.000	1.000	0.000	0.000	0.000	0.000	0.000	0.000	0.000	0.000	0.000	0.000	0.741	-11.519	2.854	-4.084	3.829
Tr-2 H-2 Sp-	Gegham 1	0.000	0.000	0.000	1.000	0.000	0.000	0.000	0.000	0.000	0.000	0.000	0.000	0.000	0.000	0.231	-10.268	3.382	-4.650	2.575
Tr-2 H-2 Sp-	Kelbadjar	0.000	0.000	0.000	0.000	0.000	0.000	0.000	0.000	0.877	0.000	0.000	0.000	0.123	0.000	-10.266	-9.931	-1.049	4.375	-1.796
Tr-2 H-2 Sp-	Gegham 1	0.000	0.000	0.000	1.000	0.000	0.000	0.000	0.000	0.000	0.000	0.000	0.000	0.000	0.000	-1.361	-12.089	3.027	-4.423	3.867
Tr-2 H-2 Sp-	Kelbadjar	0.000	0.000	0.000	0.000	0.000	0.000	0.000	0.000	1.000	0.000	0.000	0.000	0.000	0.000	-9.093	-8.984	0.091	4.965	-1.957
Tr-2 H-2 Sp-	Gegham 1	0.000	0.000	0.000	1.000	0.000	0.000	0.000	0.000	0.000	0.000	0.000	0.000	0.000	0.000	-0.909	-11.684	2.690	-4.176	3.788
Tr-2 H-2 Sp-	Kelbadjar	0.000	0.000	0.000	0.000	0.000	0.000	0.000	0.000	1.000	0.000	0.000	0.000	0.000	0.000	-9.267	-9.002	1.158	5.028	-1.872
Tr-2 H-2 Sp-	Gegham 1	0.000	0.000	0.000	1.000	0.000	0.000	0.000	0.000	0.000	0.000	0.000	0.000	0.000	0.000	-1.117	-11.339	2.533	-3.883	3.836
Tr-2 H-2 Sp-	Kelbadjar	0.000	0.000	0.000	0.000	0.000	0.000	0.000	0.000	1.000	0.000	0.000	0.000	0.000	0.000	-9.542	-8.319	0.581	4.201	-2.372
Tr-2 H-2 Sp-	Gegham 1	0.000	0.000	0.000	1.000	0.000	0.000	0.000	0.000	0.000	0.000	0.000	0.000	0.000	0.000	-0.571	-9.439	3.169	-4.651	2.690
Tr-2 H-2 Sp-	Gegham 1	0.000	0.000	0.000	1.000	0.000	0.000	0.000	0.000	0.000	0.000	0.000	0.000	0.000	0.000	-1.252	-11.112	3.397	-4.159	4.144
Tr-2 H-2 Sp-	Kelbadjar	0.000	0.000	0.000	0.000	0.000	0.000	0.000	0.000	1.000	0.000	0.000	0.000	0.000	0.000	-9.433	-7.567	1.423	3.128	-2.167
Tr-2 H-2 Sp-	Gegham 1	0.000	0.000	0.000	1.000	0.000	0.000	0.000	0.000	0.000	0.000	0.000	0.000	0.000	0.000	-1.117	-11.116	3.204	-4.475	4.516
Tr-2 H-2 Sp-	Gegham 1	0.000	0.000	0.000	1.000	0.000	0.000	0.000	0.000	0.000	0.000	0.000	0.000	0.000	0.000	-0.604	-12.544	2.408	-4.405	3.266
Tr-2 H-2 Sp-	Gegham 1	0.000	0.000	0.000	1.000	0.000	0.000	0.000	0.000	0.000	0.000	0.000	0.000	0.000	0.000	-1.824	-11.401	2.821	-4.350	4.050
Tr-2 H-2 Sp-	Gegham 1	0.000	0.000	0.000	1.000	0.000	0.000	0.000	0.000	0.000	0.000	0.000	0.000	0.000	0.000	-0.944	-12.237	2.870	-4.662	4.250
Tr-2 H-2 Sp-	Kelbadjar	0.000	0.000	0.000	0.000	0.000	0.000	0.000	0.000	1.000	0.000	0.000	0.000	0.000	0.000	-10.263	-10.154	-0.774	4.888	-1.937
Tr-2 H-2 Sp-	Gegham 1	0.000	0.000	0.000	1.000	0.000	0.000	0.000	0.000	0.000	0.000	0.000	0.000	0.000	0.000	-0.570	-10.353	3.751	-5.006	3.755
Tr-2 H-2 Sp-	Gegham 1	0.000	0.000	0.000	1.000	0.000	0.000	0.000	0.000	0.000	0.000	0.000	0.000	0.000	0.000	-0.717	-11.838	3.136	-4.905	3.636
Tr-2 H-2 Sp-	Kelbadjar	0.000	0.000	0.000	0.000	0.000	0.000	0.000	0.000	1.000	0.000	0.000	0.000	0.000	0.000	-8.817	-9.786	0.759	3.035	-1.038
Tr-2 H-2 Sp-	Gegham 1	0.000	0.000	0.000	1.000	0.000	0.000	0.000	0.000	0.000	0.000	0.000	0.000	0.000	0.000	-1.884	-12.029	2.905	-4.393	3.424
Tr-2 H-2 Sp-	Kelbadjar	0.000	0.000	0.000	0.000	0.000	0.000	0.000	0.000	1.000	0.000	0.000	0.000	0.000	0.000	-9.133	-8.988	1.591	3.323	-1.667
Tr-2 H-2 Sp-	Syunik	0.000	0.000	0.000	0.000	0.000	0.000	0.000	0.000	0.000	0.000	0.000	0.000	1.000	0.000	-6.871	-12.535	-1.098	3.857	-1.407
Tr-2 H-2 Sp-	Gegham 1	0.000	0.000	0.000	1.000	0.000	0.000	0.000	0.000	0.000	0.000	0.000	0.000	0.000	0.000	-0.481	-11.170	3.025	-4.913	4.774
Tr-2 H-2 Sp-	Gegham 1	0.000	0.000	0.000	1.000	0.000	0.000	0.000	0.000	0.000	0.000	0.000	0.000	0.000	0.000	-0.624	-9.946	3.833	-5.050	4.758
Tr-2 H-2 Sp-	Gegham 1	0.000	0.000	0.000	1.000	0.000	0.000	0.000	0.000	0.000	0.000	0.000	0.000	0.000	0.000	-0.583	-9.833	4.269	-4.598	2.453
Tr-2 H-2 Sp-	Gegham 1	0.000	0.000	0.000	1.000	0.000	0.000	0.000	0.000	0.000	0.000	0.000	0.000	0.000	0.000	-0.576	-9.693	3.600	-4.441	2.301
Tr-2 H-2 Sp-	Kelbadjar	0.000	0.000	0.000	0.000	0.000	0.000	0.000	0.000	1.000	0.000	0.000	0.000	0.000	0.000	-9.482	-8.009	0.293	4.209	-1.616
Tr-2 H-2 Sp-	Gegham 1	0.000	0.000	0.000	1.000	0.000	0.000	0.000	0.000	0.000	0.000	0.000	0.000	0.000	0.000	-0.552	-10.552	3.511	-4.609	3.849
Tr-2 H-2 Sp-	Syunik	0.000	0.000	0.000	0.000	0.000	0.000	0.000	0.000	0.000	0.000	0.000	0.000	1.000	0.000	-5.834	-11.428	-2.840	3.876	-1.192
Tr-2 H-2 Sp-	Kelbadjar	0.000	0.000	0.000	0.000	0.000	0.000	0.000	0.000	1.000	0.000	0.000	0.000	0.000	0.000	-9.417	-9.377	0.745	3.757	-1.054
Tr-2 H-2 Sp-	Gegham 1	0.000	0.000	0.000	1.000	0.000	0.000	0.000	0.000	0.000	0.000	0.000	0.000	0.000	0.000	-0.877	-10.517	3.433	-4.382	3.883
Tr-2 H-2 Sp-	Gegham 1	0.000	0.000	0.000	1.000	0.000	0.000	0.000	0.000	0.000	0.000	0.000	0.000	0.000	0.000	-1.045	-10.201	3.512	-4.601	3.994
Tr-2 H-2 Sp-	Kelbadjar	0.000	0.000	0.000	0.000	0.000	0.000	0.000	0.000	1.000	0.000	0.000	0.000	0.000	0.000	-9.151	-10.922	1.363	3.871	-1.267
Tr-2 H-2 Sp-	Kelbadjar	0.000	0.000	0.000	0.000	0.000	0.000	0.000	0.000	1.000	0.000	0.000	0.000	0.000	0.000	-9.274	-10.167	-0.910	4.339	-1.494
Tr-2 H-2 Sp-	Gegham 1	0.000	0.000	0.000	1.000	0.000	0.000	0.000	0.000	0.000	0.000	0.000	0.000	0.000	0.000	0.738	-10.156	3.636	-4.061	2.366
Tr-2 H-2 Sp-	Gegham 1	0.000	0.000	0.000	1.000	0.000	0.000	0.000	0.000	0.000	0.000	0.000	0.000	0.000	0.000	-0.197	-11.693	2.503	-4.740	3.844
Tr-2 H-2 Sp-	Gegham 1	0.000	0.000	0.000	1.000	0.000	0.000	0.000	0.000	0.000	0.000	0.000	0.000	0.000	0.000	-0.911	-10.600	2.892	-4.590	7.308
Tr-2 H-2 Sp-	Kelbadjar	0.000	0.000	0.000	0.000	0.000	0.000	0.000	0.000	1.000	0.000	0.000	0.000	0.000	0.000	-9.371	-9.482	0.849	5.179	-1.800
Tr-2 H-2 Sp-	Kelbadjar	0.000	0.000	0.000	0.000	0.000	0.000	0.000	0.000	1.000	0.000	0.000	0.000	0.000	0.000	-8.787	-8.845	1.697	3.855	-1.946
Tr-2 H-2 Sp-	Gegham 1	0.000	0.000	0.000	1.000	0.000	0.000	0.000	0.000	0.000	0.000	0.000	0.000	0.000	0.000	-0.470	-10.671	3.987	-5.004	4.343
Tr-2 H-2 Sp-	Gegham 1	0.000	0.000	0.000	1.000	0.000	0.000	0.000	0.000	0.000	0.000	0.000	0.000	0.000	0.000	-0.825	-10.237	3.993	-4.634	3.273
Tr-2 H-2 Sp-	Kelbadjar	0.000	0.000	0.000	0.000	0.000	0.000	0.000	0.000	1.000	0.000	0.000	0.000	0.000	0.000	-9.549	-10.114	0.636	4.093	-1.541
Tr-2 H-2 Sp-	Kelbadjar	0.000	0.000	0.000	0.000	0.000	0.000	0.000	0.000	0.999	0.000	0.000	0.000	0.001	0.000	-10.268	-11.239	-0.678	4.739	-1.359
Tr-2 H-2 Sp-	Gegham 1	0.000	0.000	0.000	1.000	0.000	0.000	0.000	0.000	0.000	0.000	0.000	0.000	0.000	0.000	-0.026	-10.779	2.590	-4.458	4.114
Tr-2 H-2 Sp-	Syunik	0.000	0.000	0.000	0.000	0.000	0.000	0.000	0.000	0.000	0.000	0.000	0.000	1.000	0.000	-5.419	-12.099	-1.244	4.733	-1.056
Tr-2 H-2 Sp-	Gegham 1	0.000	0.000	0.000	1.000	0.000	0.000	0.000	0.000	0.000	0.000	0.000	0.000	0.000	0.000	-2.159	-10.975	2.964	-4.684	4.686
Tr-2 H-2 Sp-	Gegham 1	0.000	0.000	0.000	1.000	0.000	0.000	0.000	0.000	0.000	0.000	0.000	0.000	0.000	0.000	-0.685	-11.705	2.896	-4.728	4.452
Tr-2 H-2 Sp-	Gegham 1	0.000	0.000	0.000	1.000	0.000	0.000	0.000	0.000	0.000	0.000	0.000	0.000	0.000	0.000	-0.392	-10.756	3.549	-4.817	4.224
Tr-2 H-2 Sp-	Gegham 1	0.000	0.000	0.000	1.000	0.000	0.000	0.000	0.000	0.000	0.000	0.000	0.000	0.000	0.000	0.328	-11.376	3.882	-4.311	2.636
Tr-2 H-2 Sp-	Kelbadjar	0.000	0.000	0.000	0.000	0.000	0.000	0.000	0.000	1.000	0.000	0.000	0.000	0.000	0.000	-9.381	-10.337	-1.378	4.661	-0.933
Tr-2 H-2 Sp-	Kelbadjar	0.000	0.000	0.000	0.000	0.000	0.000	0.000	0.000	1.000	0.000	0.000	0.000	0.000	0.000	-9.285	-8.913	0.300	4.223	-1.883
Tr-2 H-2 Sp-	Kelbadjar	0.000	0.000	0.000	0.000	0.000	0.000	0.000	0.000	1.000	0.000	0.000	0.000	0.000	0.000	-9.168	-9.265	0.720	3.707	-1.597
Tr-2 H-2 Sp-	Syunik	0.000	0.000	0.000	0.000	0.000	0.000	0.000	0.000	0.001	0.000	0.000	0.000	0.999	0.000	-5.727	-11.579	-0.890	4.223	-2.140
Tr-2 H-2 Sp-	Gegham 1	0.000	0.000	0.000	1.000	0.000	0.000	0.000	0.000	0.000	0.000	0.000	0.000	0.000	0.000	-1.574	-11.409	2.776	-2.888	1.715
Tr-2 H-2 Sp-	Gegham 1	0.000	0.000	0.000	1.000	0.000	0.000	0.000	0.000	0.000	0.000	0.000	0.000	0.000	0.000	-0.344	-11.000	3.238	-4.761	6.758
Tr-2 H-2 Sp-	Gegham 1	0.000	0.000	0.000	1.000	0.000	0.000	0.000	0.000	0.000	0.000	0.000	0.000	0.000	0.000	-0.835	-9.236	3.658	-4.458	3.098
Trench-2 Hor	Kelbadjar	0.000	0.000	0.000	0.000	0.000	0.000	0.000	0.000	1.000	0.000	0.000	0.000	0.000	0.000	-7.701	-8.666	1.338	2.717	-0.998
Trench-2 Hor	Kelbadjar	0.000	0.000	0.000	0.000	0.000	0.000	0.000	0.000	1.000	0.000	0.000	0.000	0.000	0.000	-9.590	-9.438	-0.034	4.840	-1.538
Trench-2 Hor	Gegham 1	0.000	0.000	0.000	1.000	0.000	0.000	0.000	0.000	0.000	0.000	0.000	0.000	0.000	0.000	-1.269	-10.797	3.201	-4.484	3.417
Trench-2 Hor	Kelbadjar	0.000	0.000	0.000	0.000	0.000	0.000	0.000	0.000	1.000	0.000	0.000	0.000	0.000	0.000	-9.570	-10.233	0.482	3.371	-0.861
Trench-2 Hor	Kelbadjar	0.000	0.000	0.000	0.000	0.000	0.000	0.000	0.000	1.000	0.000	0.000	0.000	0.000	0.000	-8.307	-8.830	1.475	2.667	-1.524
Trench-2 Hor	Gegham 1	0.000	0.000	0.000	1.000	0.000	0.000	0.000	0.000	0.000	0.000	0.000	0.000	0.000	0.000	0.213	-11.500	3.806	-4.865	3.260
Trench-2 Hor	Syunik	0.000	0.000	0.000	0.000	0.000	0.000	0.000	0.000	0.000	0.000	0.000	0.000	1.000	0.000	-4.857	-11.971	-1.402	2.839	-1.084
Trench-2 Hor	Syunik	0.000	0.000	0.000	0.000	0.000	0.000	0.000	0.000	0.000	0.000	0.000	0.000	1.000	0.000	-8.830	-18.738	-4.599	2.308	0.436
Trench-2 Hor	Kelbadjar	0.000	0.000	0.000	0.000	0.000	0.000	0.000	0.000	1.000	0.000	0.000	0.000	0.000	0.000	-7.439	-7.971	-1.377	5.858	-1.317

Trench-2 Hor	Gegham 1	0.000	0.000	0.000	1.000	0.000	0.000	0.000	0.000	0.000	0.000	0.000	0.000	0.000	0.000	-0.512	-11.217	2.975	-4.476	3.186
Trench-2 Hor	Kelbadjar	0.000	0.000	0.000	0.000	0.000	0.000	0.000	0.000	1.000	0.000	0.000	0.000	0.000	0.000	-7.064	-8.934	2.319	2.990	-1.171
Trench-2 Hor	Gegham 1	0.000	0.000	0.000	1.000	0.000	0.000	0.000	0.000	0.000	0.000	0.000	0.000	0.000	0.000	-0.692	-11.546	3.193	-4.473	2.100
Trench-2 Hor	Kelbadjar	0.000	0.000	0.000	0.000	0.000	0.000	0.000	0.000	1.000	0.000	0.000	0.000	0.000	0.000	-9.411	-8.330	1.512	3.903	-1.569
Trench-2 Hor	Gegham 1	0.000	0.000	0.000	1.000	0.000	0.000	0.000	0.000	0.000	0.000	0.000	0.000	0.000	0.000	-0.806	-10.443	4.068	-4.468	2.938
Trench-2 Hor	Gegham 1	0.000	0.000	0.000	1.000	0.000	0.000	0.000	0.000	0.000	0.000	0.000	0.000	0.000	0.000	-0.761	-11.067	3.500	-4.178	3.282
Trench-2 Hor	Gegham 1	0.000	0.000	0.000	1.000	0.000	0.000	0.000	0.000	0.000	0.000	0.000	0.000	0.000	0.000	-0.698	-10.707	3.591	-4.560	1.904
Trench-2 Hor	Syunik	0.000	0.000	0.000	0.000	0.000	0.000	0.000	0.000	0.000	0.000	0.000	0.000	1.000	0.000	-6.152	-12.626	-0.394	3.764	-1.576
Trench-2 Hor	Kelbadjar	0.000	0.000	0.000	0.000	0.000	0.000	0.000	0.000	1.000	0.000	0.000	0.000	0.000	0.000	-9.289	-9.068	0.742	3.560	-1.329
Trench-2 Hor	Gegham 1	0.000	0.000	0.000	1.000	0.000	0.000	0.000	0.000	0.000	0.000	0.000	0.000	0.000	0.000	0.181	-11.679	2.013	-3.815	2.415
Trench-2 Hor	Kelbadjar	0.000	0.000	0.000	0.000	0.000	0.000	0.000	0.000	1.000	0.000	0.000	0.000	0.000	0.000	-9.501	-10.244	1.784	3.593	-1.191
Trench-2 Hor	Gegham 1	0.000	0.000	0.000	1.000	0.000	0.000	0.000	0.000	0.000	0.000	0.000	0.000	0.000	0.000	-1.530	-12.717	2.486	-3.900	2.781
Trench-2 Hor	Kelbadjar	0.000	0.000	0.000	0.000	0.000	0.000	0.000	0.000	1.000	0.000	0.000	0.000	0.000	0.000	-9.292	-9.786	0.468	2.822	-0.639
Trench-2 Hor	Gegham 1	0.000	0.000	0.000	1.000	0.000	0.000	0.000	0.000	0.000	0.000	0.000	0.000	0.000	0.000	-0.696	-11.993	2.802	-4.168	2.936
Trench-2 Hor	Gegham 1	0.000	0.000	0.000	1.000	0.000	0.000	0.000	0.000	0.000	0.000	0.000	0.000	0.000	0.000	-1.334	-11.781	2.613	-4.535	5.468
Trench-2 Hor	Kelbadjar	0.000	0.000	0.000	0.000	0.000	0.000	0.000	0.000	1.000	0.000	0.000	0.000	0.000	0.000	-8.235	-9.868	1.397	2.655	-1.347
Trench-2 Hor	Kelbadjar	0.000	0.000	0.000	0.000	0.000	0.000	0.000	0.000	1.000	0.000	0.000	0.000	0.000	0.000	-8.718	-9.922	1.887	3.540	-1.836
Trench-2 Hor	Gegham 1	0.000	0.000	0.000	1.000	0.000	0.000	0.000	0.000	0.000	0.000	0.000	0.000	0.000	0.000	-2.661	-13.280	2.539	-4.591	7.443
Trench-2 Hor	Kelbadjar	0.000	0.000	0.000	0.000	0.000	0.000	0.000	0.000	1.000	0.000	0.000	0.000	0.000	0.000	-7.797	-9.972	0.749	4.243	-1.872
Trench-2 Hor	Kelbadjar	0.000	0.000	0.000	0.000	0.000	0.000	0.000	0.000	1.000	0.000	0.000	0.000	0.000	0.000	-8.923	-10.534	-0.075	4.164	-1.917
Trench-2 Hor	Kelbadjar	0.000	0.000	0.000	0.000	0.000	0.000	0.000	0.000	1.000	0.000	0.000	0.000	0.000	0.000	-9.206	-9.751	0.510	4.568	-1.437
Trench-2 Hor	Gegham 1	0.000	0.000	0.000	1.000	0.000	0.000	0.000	0.000	0.000	0.000	0.000	0.000	0.000	0.000	-0.601	-10.639	2.746	-4.700	4.018
Trench-2 Hor	Gegham 1	0.000	0.000	0.000	1.000	0.000	0.000	0.000	0.000	0.000	0.000	0.000	0.000	0.000	0.000	-1.218	-12.476	2.869	-4.278	3.094
Trench-2 Hor	Gutansar	0.000	0.000	0.000	0.000	0.000	1.000	0.000	0.000	0.000	0.000	0.000	0.000	0.000	0.000	5.453	2.131	10.445	14.084	-6.359
Trench-2 Hor	Kelbadjar	0.000	0.000	0.000	0.000	0.000	0.000	0.000	0.000	1.000	0.000	0.000	0.000	0.000	0.000	-10.013	-9.886	-0.405	6.322	-2.405
Trench-2 Hor	Gegham 1	0.000	0.000	0.000	1.000	0.000	0.000	0.000	0.000	0.000	0.000	0.000	0.000	0.000	0.000	-1.477	-10.691	3.866	-4.580	3.881
Trench-2 Hor	Kelbadjar	0.000	0.000	0.000	0.000	0.000	0.000	0.000	0.000	1.000	0.000	0.000	0.000	0.000	0.000	-10.387	-10.092	-0.074	4.413	-1.760
Trench-2 Hor	Gegham 1	0.000	0.000	0.000	1.000	0.000	0.000	0.000	0.000	0.000	0.000	0.000	0.000	0.000	0.000	-0.190	-12.292	3.569	-4.667	3.307
Trench-2 Hor	Kelbadjar	0.000	0.000	0.000	0.000	0.000	0.000	0.000	0.000	1.000	0.000	0.000	0.000	0.000	0.000	-9.268	-9.173	0.548	3.672	-1.576
Trench-2 Hor	Kelbadjar	0.000	0.000	0.000	0.000	0.000	0.000	0.000	0.000	1.000	0.000	0.000	0.000	0.000	0.000	-9.600	-9.504	0.024	3.958	-1.736
Trench-2 Hor	Gegham 1	0.000	0.000	0.000	1.000	0.000	0.000	0.000	0.000	0.000	0.000	0.000	0.000	0.000	0.000	-0.716	-10.899	3.147	-4.489	2.858
Trench-2 Hor	Kelbadjar	0.000	0.000	0.000	0.000	0.000	0.000	0.000	0.000	1.000	0.000	0.000	0.000	0.000	0.000	-9.440	-11.106	1.233	5.035	-2.137
Trench-2 Hor	Gegham 1	0.000	0.000	0.000	1.000	0.000	0.000	0.000	0.000	0.000	0.000	0.000	0.000	0.000	0.000	-1.006	-11.186	3.050	-4.131	2.932
Trench-2 Hor	Gegham 1	0.000	0.000	0.000	1.000	0.000	0.000	0.000	0.000	0.000	0.000	0.000	0.000	0.000	0.000	-0.287	-9.737	3.735	-4.773	5.069
Trench-2 Hor	Syunik	0.000	0.000	0.000	0.000	0.000	0.000	0.000	0.000	0.000	0.000	0.000	0.000	1.000	0.000	-6.923	-12.963	-3.506	4.633	-1.072
Trench-2 Hor	Kelbadjar	0.000	0.000	0.000	0.000	0.000	0.000	0.000	0.000	1.000	0.000	0.000	0.000	0.000	0.000	-9.820	-8.592	0.785	6.474	-2.105
Trench-2 Hor	Gegham 1	0.000	0.000	0.000	1.000	0.000	0.000	0.000	0.000	0.000	0.000	0.000	0.000	0.000	0.000	-0.760	-11.154	3.610	-4.531	4.212
Trench-2 Hor	Gegham 1	0.000	0.000	0.000	1.000	0.000	0.000	0.000	0.000	0.000	0.000	0.000	0.000	0.000	0.000	-0.880	-10.581	3.469	-4.511	2.489
Trench-2 Hor	Gegham 1	0.000	0.000	0.000	1.000	0.000	0.000	0.000	0.000	0.000	0.000	0.000	0.000	0.000	0.000	-0.266	-11.726	3.037	-4.645	5.350
Trench-2 Hor	Kelbadjar	0.000	0.000	0.000	0.000	0.000	0.000	0.000	0.000	1.000	0.000	0.000	0.000	0.000	0.000	-9.326	-9.122	1.070	3.977	-1.195
Trench-2 Hor	Kelbadjar	0.000	0.000	0.000	0.000	0.000	0.000	0.000	0.000	1.000	0.000	0.000	0.000	0.000	0.000	-9.327	-10.659	-0.375	5.273	-1.787
Trench-2 Hor	Syunik	0.000	0.000	0.000	0.000	0.000	0.000	0.000	0.000	0.034	0.000	0.000	0.000	0.966	0.000	-10.445	-10.339	-1.362	5.798	-2.034
Trench-2 Hor	Gegham 1	0.000	0.000	0.000	1.000	0.000	0.000	0.000	0.000	0.000	0.000	0.000	0.000	0.000	0.000	0.004	-10.085	3.741	-4.626	3.331
Trench-2 Hor	Gegham 1	0.000	0.000	0.000	1.000	0.000	0.000	0.000	0.000	0.000	0.000	0.000	0.000	0.000	0.000	-1.244	-11.050	3.013	-4.216	3.333
Trench-2 Hor	Gegham 1	0.000	0.000	0.000	1.000	0.000	0.000	0.000	0.000	0.000	0.000	0.000	0.000	0.000	0.000	-0.929	-11.340	3.073	-4.526	3.426
Trench-2 Hor	Arteni	0.000	1.000	0.000	0.000	0.000	0.000	0.000	0.000	0.000	0.000	0.000	0.000	0.000	0.000	-2.027	0.897	3.990	-3.279	-6.377
Trench 2 Hor	Gegham 1	0.000	0.000	0.000	1.000	0.000	0.000	0.000	0.000	0.000	0.000	0.000	0.000	0.000	0.000	-1.259	-11.096	3.224	-4.239	1.112
Trench 2 Hor	Kelbadjar	0.000	0.000	0.000	0.000	0.000	0.000	0.000	0.000	1.000	0.000	0.000	0.000	0.000	0.000	-9.700	-9.583	1.333	4.698	-1.654
Trench 2 Hor	Kelbadjar	0.000	0.000	0.000	0.000	0.000	0.000	0.000	0.000	1.000	0.000	0.000	0.000	0.000	0.000	-9.498	-8.221	0.445	4.372	-1.742
Trench 2 Hor	Gegham 1	0.000	0.000	0.000	1.000	0.000	0.000	0.000	0.000	0.000	0.000	0.000	0.000	0.000	0.000	-1.282	-11.790	1.885	-4.167	3.648
Trench 2 Hor	Syunik	0.000	0.000	0.000	0.000	0.000	0.000	0.000	0.000	0.000	0.000	0.000	0.000	1.000	0.000	-5.197	-12.233	-2.617	2.337	0.318
Trench 2 Hor	Kelbadjar	0.000	0.000	0.000	0.000	0.000	0.000	0.000	0.000	1.000	0.000	0.000	0.000	0.000	0.000	-8.973	-9.811	1.479	3.696	-1.070
Trench 2 Hor	Gegham 1	0.000	0.000	0.000	1.000	0.000	0.000	0.000	0.000	0.000	0.000	0.000	0.000	0.000	0.000	-1.584	-12.023	3.125	-4.184	2.409
Trench 2 Hor	Kelbadjar	0.000	0.000	0.000	0.000	0.000	0.000	0.000	0.000	1.000	0.000	0.000	0.000	0.000	0.000	-8.497	-10.030	1.177	3.277	-1.151
Trench 2 Hor	Kelbadjar	0.000	0.000	0.000	0.000	0.000	0.000	0.000	0.000	1.000	0.000	0.000	0.000	0.000	0.000	-8.013	-9.028	1.236	2.921	-1.665
Trench 2 Hor	Kelbadjar	0.000	0.000	0.000	0.000	0.000	0.000	0.000	0.000	1.000	0.000	0.000	0.000	0.000	0.000	-9.080	-9.747	0.323	3.007	-1.168
Trench 2 Hor	Gegham 1	0.000	0.000	0.000	1.000	0.000	0.000	0.000	0.000	0.000	0.000	0.000	0.000	0.000	0.000	0.088	-10.348	2.140	-4.557	3.342
Trench 2 Hor	Kelbadjar	0.000	0.000	0.000	0.000	0.000	0.000	0.000	0.000	1.000	0.000	0.000	0.000	0.000	0.000	-8.066	-9.256	1.166	2.034	-0.811
Trench 2 Hor	Kelbadjar	0.000	0.000	0.000	0.000	0.000	0.000	0.000	0.000	1.000	0.000	0.000	0.000	0.000	0.000	-10.191	-8.373	-0.234	3.394	-1.156
Trench 2 Hor	Kelbadjar	0.000	0.000	0.000	0.000	0.000	0.000	0.000	0.000	1.000	0.000	0.000	0.000	0.000	0.000	-9.200	-9.275	0.076	3.638	-1.244
Trench 2 Hor	Kelbadjar	0.000	0.000	0.000	0.000	0.000	0.000	0.000	0.000	1.000	0.000	0.000	0.000	0.000	0.000	-8.920	-8.231	1.062	3.461	-1.567
Trench 2 Hor	Kelbadjar	0.000	0.000	0.000	0.000	0.000	0.000	0.000	0.000	1.000	0.000	0.000	0.000	0.000	0.000	-9.884	-9.694	1.862	3.629	-1.740
Trench 2 Hor	Gegham 1	0.000	0.000	0.000	1.000	0.000	0.000	0.000	0.000	0.000	0.000	0.000	0.000	0.000	0.000	0.354	-9.319	4.187	-4.666	2.729
Trench 2 Hor	Kelbadjar	0.000	0.000	0.000	0.000	0.000	0.000	0.000	0.000	1.000	0.000	0.000	0.000	0.000	0.000	-9.448	-10.149	1.142	4.076	-1.669
Trench 2 Hor	Gegham 1	0.000	0.000	0.000	1.000	0.000	0.000	0.000	0.000	0.000	0.000	0.000	0.000	0.000	0.000	1.283	-10.208	2.802	-4.278	2.627
Trench 2 Hor	Gegham 1	0.000	0.000	0.000	1.000	0.000	0.000	0.000	0.000	0.000	0.000	0.000	0.000	0.000	0.000	-0.173	-9.857	3.600	-4.665	2.563
Trench 2 Hor	Gegham 1	0.000	0.000	0.000	1.000	0.000	0.000	0.000	0.000	0.000	0.000	0.000	0.000	0.000	0.000	-0.440	-10.428	2.967	-4.500	4.021

Trench 2 Hor	Kelbadjar	0.000	0.000	0.000	0.000	0.000	0.000	0.000	0.000	1.000	0.000	0.000	0.000	0.000	0.000	-8.956	-8.934	-0.175	4.942	-1.302
Trench 2 Hor	Arteni	0.000	1.000	0.000	0.000	0.000	0.000	0.000	0.000	0.000	0.000	0.000	0.000	0.000	0.000	-3.809	0.534	4.158	-3.424	-5.596
Trench 2 Hor	Gegham 1	0.000	0.000	0.000	1.000	0.000	0.000	0.000	0.000	0.000	0.000	0.000	0.000	0.000	0.000	-0.313	-12.743	2.648	-4.705	3.615
Trench 2 Hor	Gegham 1	0.000	0.000	0.000	1.000	0.000	0.000	0.000	0.000	0.000	0.000	0.000	0.000	0.000	0.000	-0.035	-11.041	3.156	-4.443	2.789
Trench 2 Hor	Kelbadjar	0.000	0.000	0.000	0.000	0.000	0.000	0.000	0.000	1.000	0.000	0.000	0.000	0.000	0.000	-8.842	-9.815	0.421	3.681	-1.499
Trench 2 Hor	Gegham 1	0.000	0.000	0.000	1.000	0.000	0.000	0.000	0.000	0.000	0.000	0.000	0.000	0.000	0.000	-0.636	-9.450	3.869	-4.273	1.467
Trench 2 Hor	Kelbadjar	0.000	0.000	0.000	0.000	0.000	0.000	0.000	0.000	1.000	0.000	0.000	0.000	0.000	0.000	-9.871	-9.109	0.732	4.285	-1.721
Trench 2 Hor	Kelbadjar	0.000	0.000	0.000	0.000	0.000	0.000	0.000	0.000	1.000	0.000	0.000	0.000	0.000	0.000	-8.397	-9.530	-0.773	3.170	-0.566
Trench 2 Hor	Kelbadjar	0.000	0.000	0.000	0.000	0.000	0.000	0.000	0.000	1.000	0.000	0.000	0.000	0.000	0.000	-9.024	-7.574	1.011	3.317	-1.589
Trench 2 Hor	Gegham 1	0.000	0.000	0.000	1.000	0.000	0.000	0.000	0.000	0.000	0.000	0.000	0.000	0.000	0.000	-2.709	-12.406	2.271	-3.541	1.994
Trench 2 Hor	Gegham 1	0.000	0.000	0.000	1.000	0.000	0.000	0.000	0.000	0.000	0.000	0.000	0.000	0.000	0.000	8.229	-8.847	0.558	-3.779	5.725
Trench 2 Hor	Syunik	0.000	0.000	0.000	0.000	0.000	0.000	0.000	0.000	0.001	0.000	0.000	0.000	0.999	0.000	-6.043	-11.597	-2.147	3.051	-0.327
Trench 2 Hor	Gegham 1	0.000	0.000	0.000	1.000	0.000	0.000	0.000	0.000	0.000	0.000	0.000	0.000	0.000	0.000	-0.977	-11.263	3.077	-4.339	3.271
Trench 2 Hor	Kelbadjar	0.000	0.000	0.000	0.000	0.000	0.000	0.000	0.000	1.000	0.000	0.000	0.000	0.000	0.000	-8.971	-9.041	1.340	3.309	-1.593
Trench 2 Hor	Kelbadjar	0.000	0.000	0.000	0.000	0.000	0.000	0.000	0.000	1.000	0.000	0.000	0.000	0.000	0.000	-9.653	-11.022	0.060	4.655	-2.043
Trench 2 Hor	Gegham 1	0.000	0.000	0.000	1.000	0.000	0.000	0.000	0.000	0.000	0.000	0.000	0.000	0.000	0.000	0.216	-10.353	3.542	-4.952	5.126
Trench 2 Hor	Gegham 1	0.000	0.000	0.000	1.000	0.000	0.000	0.000	0.000	0.000	0.000	0.000	0.000	0.000	0.000	-0.460	-11.922	1.467	-3.958	3.120
Trench 2 Hor	Kelbadjar	0.000	0.000	0.000	0.000	0.000	0.000	0.000	0.000	1.000	0.000	0.000	0.000	0.000	0.000	-9.543	-9.506	0.693	4.053	-1.762
Trench 2 Hor	Kelbadjar	0.000	0.000	0.000	0.000	0.000	0.000	0.000	0.000	1.000	0.000	0.000	0.000	0.000	0.000	-8.903	-10.878	0.908	4.943	-1.309
Trench 2 Hor	Kelbadjar	0.000	0.000	0.000	0.000	0.000	0.000	0.000	0.000	1.000	0.000	0.000	0.000	0.000	0.000	-9.076	-9.100	1.326	3.540	-1.353
Trench 2 Hor	Gegham 1	0.000	0.000	0.000	1.000	0.000	0.000	0.000	0.000	0.000	0.000	0.000	0.000	0.000	0.000	0.576	-10.584	3.637	-4.526	3.040
Trench 2 Hor	Gegham 1	0.000	0.000	0.000	1.000	0.000	0.000	0.000	0.000	0.000	0.000	0.000	0.000	0.000	0.000	-1.245	-10.367	3.357	-3.750	1.981
Trench 2 Hor	Kelbadjar	0.000	0.000	0.000	0.000	0.000	0.000	0.000	0.000	1.000	0.000	0.000	0.000	0.000	0.000	-9.130	-9.652	0.504	2.401	-1.285
Trench 2 Hor	Gegham 1	0.000	0.000	0.000	1.000	0.000	0.000	0.000	0.000	0.000	0.000	0.000	0.000	0.000	0.000	-0.106	-11.801	3.959	-4.042	2.199
Trench 2 Hor	Gegham 1	0.000	0.000	0.000	1.000	0.000	0.000	0.000	0.000	0.000	0.000	0.000	0.000	0.000	0.000	-0.995	-11.326	3.095	-4.273	3.497
Trench 2 Hor	Gegham 1	0.000	0.000	0.000	1.000	0.000	0.000	0.000	0.000	0.000	0.000	0.000	0.000	0.000	0.000	-1.730	-10.769	3.439	-4.895	4.520
Trench 2 Hor	Kelbadjar	0.000	0.000	0.000	0.000	0.000	0.000	0.000	0.000	1.000	0.000	0.000	0.000	0.000	0.000	-8.612	-8.832	2.272	2.920	-1.796
Trench 2 Hor	Gegham 1	0.000	0.000	0.000	1.000	0.000	0.000	0.000	0.000	0.000	0.000	0.000	0.000	0.000	0.000	-1.395	-9.939	3.089	-3.892	2.599
Trench 2 Hor	Kelbadjar	0.000	0.000	0.000	0.000	0.000	0.000	0.000	0.000	1.000	0.000	0.000	0.000	0.000	0.000	-9.566	-10.147	-0.299	4.508	-1.430
Trench 2 Hor	Gegham 1	0.000	0.000	0.000	1.000	0.000	0.000	0.000	0.000	0.000	0.000	0.000	0.000	0.000	0.000	-2.005	-12.123	2.975	-4.400	3.803
Trench 2 Hor	Kelbadjar	0.000	0.000	0.000	0.000	0.000	0.000	0.000	0.000	1.000	0.000	0.000	0.000	0.000	0.000	-9.596	-9.064	0.752	2.713	-1.366
Trench 2 Hor	Syunik	0.000	0.000	0.000	0.000	0.000	0.000	0.000	0.000	0.000	0.000	0.000	0.000	1.000	0.000	-6.817	-13.143	-0.990	3.729	-1.515
Trench 2 Hor	Syunik	0.000	0.000	0.000	0.000	0.000	0.000	0.000	0.000	0.011	0.000	0.000	0.000	0.989	0.000	-6.911	-11.720	-0.302	2.609	-1.212
Trench 2 Hor	Arteni	0.000	1.000	0.000	0.000	0.000	0.000	0.000	0.000	0.000	0.000	0.000	0.000	0.000	0.000	-4.305	1.744	4.787	-3.486	-6.459
Trench 2 Hor	Kelbadjar	0.000	0.000	0.000	0.000	0.000	0.000	0.000	0.000	1.000	0.000	0.000	0.000	0.000	0.000	-8.713	-8.911	0.297	2.476	-0.773
Trench 2 Hor	Gegham 1	0.000	0.000	0.000	1.000	0.000	0.000	0.000	0.000	0.000	0.000	0.000	0.000	0.000	0.000	4.959	-10.639	1.983	-4.505	5.102
Trench 2 Hor	Gegham 1	0.000	0.000	0.000	1.000	0.000	0.000	0.000	0.000	0.000	0.000	0.000	0.000	0.000	0.000	-0.752	-10.648	2.943	-4.624	3.185
Trench 2 Hor	Kelbadjar	0.000	0.000	0.000	0.000	0.000	0.000	0.000	0.000	1.000	0.000	0.000	0.000	0.000	0.000	-9.575	-8.576	0.711	4.730	-1.941
Trench 2 Hor	Gegham 1	0.000	0.000	0.000	1.000	0.000	0.000	0.000	0.000	0.000	0.000	0.000	0.000	0.000	0.000	-1.068	-10.457	2.811	-4.123	3.238
Trench 2 Hor	Syunik	0.000	0.000	0.000	0.000	0.000	0.000	0.000	0.000	0.000	0.000	0.000	0.000	1.000	0.000	-6.506	-13.015	-1.661	3.614	-1.391
Trench 2 Hor	Gegham 1	0.000	0.000	0.000	1.000	0.000	0.000	0.000	0.000	0.000	0.000	0.000	0.000	0.000	0.000	-0.853	-11.091	3.198	-4.546	3.622
Trench 2 Hor	Kelbadjar	0.000	0.000	0.000	0.000	0.000	0.000	0.000	0.000	1.000	0.000	0.000	0.000	0.000	0.000	-11.287	-10.137	-0.800	6.299	-2.386
Trench 2 Hor	Gegham 1	0.000	0.000	0.000	1.000	0.000	0.000	0.000	0.000	0.000	0.000	0.000	0.000	0.000	0.000	-1.415	-9.845	3.004	-3.812	1.437
Trench 2 Hor	Gegham 1	0.000	0.000	0.000	1.000	0.000	0.000	0.000	0.000	0.000	0.000	0.000	0.000	0.000	0.000	-1.211	-12.104	3.364	-4.781	4.172
Trench 2 Hor	Kelbadjar	0.000	0.000	0.000	0.000	0.000	0.000	0.000	0.000	1.000	0.000	0.000	0.000	0.000	0.000	-9.196	-8.627	0.682	3.741	-1.491
Trench 2 Hor	Gegham 1	0.000	0.000	0.000	1.000	0.000	0.000	0.000	0.000	0.000	0.000	0.000	0.000	0.000	0.000	0.087	-10.385	3.542	-4.641	3.972
Trench 2 Hor	Kelbadjar	0.000	0.000	0.000	0.000	0.000	0.000	0.000	0.000	1.000	0.000	0.000	0.000	0.000	0.000	-9.144	-8.494	1.147	3.421	-2.335
Trench 2 Hor	Kelbadjar	0.000	0.000	0.000	0.000	0.000	0.000	0.000	0.000	1.000	0.000	0.000	0.000	0.000	0.000	-8.944	-9.210	1.340	4.245	-2.008
Trench 2 Hor	Kelbadjar	0.000	0.000	0.000	0.000	0.000	0.000	0.000	0.000	1.000	0.000	0.000	0.000	0.000	0.000	-8.535	-9.109	0.826	3.625	-1.973
Trench 2 Hor	Gegham 1	0.000	0.000	0.000	1.000	0.000	0.000	0.000	0.000	0.000	0.000	0.000	0.000	0.000	0.000	-0.612	-11.719	3.092	-3.820	2.279
Trench 2 Hor	Kelbadjar	0.000	0.000	0.000	0.000	0.000	0.000	0.000	0.000	1.000	0.000	0.000	0.000	0.000	0.000	-9.316	-9.665	0.822	3.995	-1.762
Trench 2 Hor	Kelbadjar	0.000	0.000	0.000	0.000	0.000	0.000	0.000	0.000	1.000	0.000	0.000	0.000	0.000	0.000	-9.091	-9.149	1.703	3.876	-1.953
Trench 2 Hor	Gegham 1	0.000	0.000	0.000	1.000	0.000	0.000	0.000	0.000	0.000	0.000	0.000	0.000	0.000	0.000	-0.096	-9.824	3.982	-4.261	1.916
Trench 2 Hor	Kelbadjar	0.000	0.000	0.000	0.000	0.000	0.000	0.000	0.000	1.000	0.000	0.000	0.000	0.000	0.000	-8.794	-9.021	0.944	3.598	-1.628
Trench 2 Hor	Syunik	0.000	0.000	0.000	0.000	0.000	0.000	0.000	0.000	0.000	0.000	0.000	0.000	1.000	0.000	-7.055	-12.773	-1.651	4.321	-1.560
Hor-2 Tr-2 Sp	Gegham 1	0.000	0.000	0.000	1.000	0.000	0.000	0.000	0.000	0.000	0.000	0.000	0.000	0.000	0.000	-2.294	-13.277	1.992	-4.286	4.004
Hor-2 Tr-2 Sp	Gegham 1	0.000	0.000	0.000	1.000	0.000	0.000	0.000	0.000	0.000	0.000	0.000	0.000	0.000	0.000	-1.849	-12.638	2.598	-4.316	3.620
Hor-2 Tr-2 Sp	Gegham 1	0.000	0.000	0.000	1.000	0.000	0.000	0.000	0.000	0.000	0.000	0.000	0.000	0.000	0.000	-0.519	-11.632	3.012	-4.684	3.505
Hor-2 Tr-2 Sp	Gegham 1	0.000	0.000	0.000	1.000	0.000	0.000	0.000	0.000	0.000	0.000	0.000	0.000	0.000	0.000	-0.739	-10.405	3.702	-3.931	2.781
Hor-2 Tr-2 Sp	Gegham 1	0.000	0.000	0.000	1.000	0.000	0.000	0.000	0.000	0.000	0.000	0.000	0.000	0.000	0.000	-0.274	-10.168	2.670	-4.603	3.677
Hor-2 Tr-2 Sp	Gegham 1	0.000	0.000	0.000	1.000	0.000	0.000	0.000	0.000	0.000	0.000	0.000	0.000	0.000	0.000	-1.356	-9.957	3.092	-4.630	4.107
Hor-2 Tr-2 Sp	Kelbadjar	0.000	0.000	0.000	0.000	0.000	0.000	0.000	0.000	1.000	0.000	0.000	0.000	0.000	0.000	-9.761	-9.629	0.008	5.237	-2.012
Hor-2 Tr-2 Sp	Syunik	0.000	0.000	0.000	0.000	0.000	0.000	0.000	0.000	0.000	0.000	0.000	0.000	1.000	0.000	-6.842	-13.717	-1.027	3.273	-1.074
Hor-2 Tr-2 Sp	Kelbadjar	0.000	0.000	0.000	0.000	0.000	0.000	0.000	0.000	1.000	0.000	0.000	0.000	0.000	0.000	-8.866	-8.986	0.537	3.057	-0.541
Hor-2 Tr-2 Sp	Kelbadjar	0.000	0.000	0.000	0.000	0.000	0.000	0.000	0.000	1.000	0.000	0.000	0.000	0.000	0.000	-9.977	-9.051	0.520	4.523	-1.459
Hor-2 Tr-2 Sp	Gegham 1	0.000	0.000	0.000	1.000	0.000	0.000	0.000	0.000	0.000	0.000	0.000	0.000	0.000	0.000	-0.978	-10.180	3.537	-4.662	3.511
Hor-2 Tr-2 Sp	Syunik	0.000	0.000	0.000	0.000	0.000	0.000	0.000	0.000	0.000	0.000	0.000	0.000	1.000	0.000	-7.666	-12.927	-1.175	4.280	-1.606

Hor-2 Tr-2 Sp	Kelbadjar	0.000	0.000	0.000	0.000	0.000	0.000	0.000	0.000	1.000	0.000	0.000	0.000	0.000	0.000	-8.149	-8.357	1.489	3.565	-2.081
Hor-2 Tr-2 Sp	Kelbadjar	0.000	0.000	0.000	0.000	0.000	0.000	0.000	0.000	1.000	0.000	0.000	0.000	0.000	0.000	-9.723	-9.714	1.508	4.230	-1.423
Hor-2 Tr-2 Sp	Kelbadjar	0.000	0.000	0.000	0.000	0.000	0.000	0.000	0.000	1.000	0.000	0.000	0.000	0.000	0.000	-8.908	-8.864	0.377	3.371	-0.958
Hor-2 Tr-2 Sp	Gegham 1	0.000	0.000	0.000	1.000	0.000	0.000	0.000	0.000	0.000	0.000	0.000	0.000	0.000	0.000	-1.184	-10.852	3.164	-4.534	2.920
Hor-2 Tr-2 Sp	Gegham 1	0.000	0.000	0.000	1.000	0.000	0.000	0.000	0.000	0.000	0.000	0.000	0.000	0.000	0.000	-1.363	-11.305	3.579	-4.556	2.248
Hor-2 Tr-2 Sp	Kelbadjar	0.000	0.000	0.000	0.000	0.000	0.000	0.000	0.000	1.000	0.000	0.000	0.000	0.000	0.000	-9.196	-9.085	0.517	5.271	-1.964
Hor-2 Tr-2 Sp	Kelbadjar	0.000	0.000	0.000	0.000	0.000	0.000	0.000	0.000	1.000	0.000	0.000	0.000	0.000	0.000	-8.397	-9.884	0.678	3.318	-1.231
Hor-2 Tr-2 Sp	Arteni	0.000	1.000	0.000	0.000	0.000	0.000	0.000	0.000	0.000	0.000	0.000	0.000	0.000	0.000	-2.927	1.323	4.606	-3.623	-7.184
Hor-2 Tr-2 Sp	Gegham 1	0.000	0.000	0.000	1.000	0.000	0.000	0.000	0.000	0.000	0.000	0.000	0.000	0.000	0.000	-0.916	-11.703	3.345	-4.966	4.181
Hor-2 Tr-2 Sp	Syunik	0.000	0.000	0.000	0.000	0.000	0.000	0.000	0.000	0.000	0.000	0.000	0.000	1.000	0.000	-8.484	-16.086	-2.647	2.362	0.050
Hor-2 Tr-2 Sp	Gegham 1	0.000	0.000	0.000	1.000	0.000	0.000	0.000	0.000	0.000	0.000	0.000	0.000	0.000	0.000	-0.509	-9.913	3.687	-5.087	4.816
Hor-2 Tr-2 Sp	Kelbadjar	0.000	0.000	0.000	0.000	0.000	0.000	0.000	0.000	1.000	0.000	0.000	0.000	0.000	0.000	-9.302	-9.448	0.021	4.305	-1.948
Hor-2 Tr-2 Sp	Arteni	0.000	1.000	0.000	0.000	0.000	0.000	0.000	0.000	0.000	0.000	0.000	0.000	0.000	0.000	-4.148	0.368	3.731	-3.672	-6.741
Hor-2 Tr-2 Sp	Gegham 1	0.000	0.000	0.000	1.000	0.000	0.000	0.000	0.000	0.000	0.000	0.000	0.000	0.000	0.000	-0.311	-12.162	3.082	-4.412	3.406
Hor-2 Tr-2 Sp	Kelbadjar	0.000	0.000	0.000	0.000	0.000	0.000	0.000	0.000	1.000	0.000	0.000	0.000	0.000	0.000	-9.333	-9.268	1.413	3.274	-0.732
Hor-2 Tr-2 Sp	Gegham 1	0.000	0.000	0.000	1.000	0.000	0.000	0.000	0.000	0.000	0.000	0.000	0.000	0.000	0.000	-0.358	-11.852	3.121	-5.041	3.241
Hor-2 Tr-2 Sp	Syunik	0.000	0.000	0.000	0.000	0.000	0.000	0.000	0.000	0.000	0.000	0.000	0.000	1.000	0.000	-6.875	-12.378	-2.171	3.448	-1.405
Hor-2 Tr-2 Sp	Kelbadjar	0.000	0.000	0.000	0.000	0.000	0.000	0.000	0.000	1.000	0.000	0.000	0.000	0.000	0.000	-9.524	-9.357	1.066	3.259	-1.462
Hor-2 Tr-2 Sp	Syunik	0.000	0.000	0.000	0.000	0.000	0.000	0.000	0.000	0.000	0.000	0.000	0.000	1.000	0.000	-7.508	-12.190	-1.553	3.164	-1.349
Hor-2 Tr-2 Sp	Gegham 1	0.000	0.000	0.000	1.000	0.000	0.000	0.000	0.000	0.000	0.000	0.000	0.000	0.000	0.000	-0.725	-10.579	2.597	-4.516	3.369
Hor-2 Tr-2 Sp	Kelbadjar	0.000	0.000	0.000	0.000	0.000	0.000	0.000	0.000	1.000	0.000	0.000	0.000	0.000	0.000	-8.911	-9.745	2.396	2.558	-1.399
Hor-2 Tr-2 Sp	Syunik	0.000	0.000	0.000	0.000	0.000	0.000	0.000	0.000	0.000	0.000	0.000	0.000	1.000	0.000	-6.756	-12.934	-1.698	4.564	-1.535
Hor-2 Tr-2 Sp	Kelbadjar	0.000	0.000	0.000	0.000	0.000	0.000	0.000	0.000	1.000	0.000	0.000	0.000	0.000	0.000	-9.310	-9.625	-0.071	4.336	-1.279
Hor-2 Tr-2 Sp	Syunik	0.000	0.000	0.000	0.000	0.000	0.000	0.000	0.000	0.000	0.000	0.000	0.000	1.000	0.000	-7.697	-12.337	-0.653	3.667	-1.905
Hor-2 Tr-2 Sp	Kelbadjar	0.000	0.000	0.000	0.000	0.000	0.000	0.000	0.000	1.000	0.000	0.000	0.000	0.000	0.000	-8.693	-8.252	0.609	2.288	-1.653
Hor-2 Tr-2 Sp	Gegham 1	0.000	0.000	0.000	1.000	0.000	0.000	0.000	0.000	0.000	0.000	0.000	0.000	0.000	0.000	-0.575	-10.386	3.051	-4.462	2.732
Hor-2 Tr-2 Sp	Gegham 1	0.000	0.000	0.000	1.000	0.000	0.000	0.000	0.000	0.000	0.000	0.000	0.000	0.000	0.000	-0.546	-11.430	3.191	-4.742	4.405
Hor-2 Tr-2 Sp	Gegham 1	0.000	0.000	0.000	1.000	0.000	0.000	0.000	0.000	0.000	0.000	0.000	0.000	0.000	0.000	0.186	-10.592	2.948	-4.446	4.738
Hor-2 Tr-2 Sp	Arteni	0.000	1.000	0.000	0.000	0.000	0.000	0.000	0.000	0.000	0.000	0.000	0.000	0.000	0.000	-0.002	1.681	3.363	-3.294	-5.508
Hor-2 Tr-2 Sp	Gegham 1	0.000	0.000	0.000	1.000	0.000	0.000	0.000	0.000	0.000	0.000	0.000	0.000	0.000	0.000	-0.648	-11.810	1.977	-4.243	4.055
Hor-2 Tr-2 Sp	Kelbadjar	0.000	0.000	0.000	0.000	0.000	0.000	0.000	0.000	1.000	0.000	0.000	0.000	0.000	0.000	-10.025	-9.483	1.467	4.343	-1.835
Hor-2 Tr-2 Sp	Gegham 1	0.000	0.000	0.000	1.000	0.000	0.000	0.000	0.000	0.000	0.000	0.000	0.000	0.000	0.000	0.826	-11.252	2.980	-4.611	3.221
Hor-2 Tr-2 Sp	Syunik	0.000	0.000	0.000	0.000	0.000	0.000	0.000	0.000	0.000	0.000	0.000	0.000	1.000	0.000	-7.481	-13.406	-2.454	4.312	-1.239
Hor-2 Tr-2 Sp	Arteni	0.000	1.000	0.000	0.000	0.000	0.000	0.000	0.000	0.000	0.000	0.000	0.000	0.000	0.000	-5.277	1.192	4.330	-3.541	-6.570
Hor-2 Tr-2 Sp	Gegham 1	0.000	0.000	0.000	1.000	0.000	0.000	0.000	0.000	0.000	0.000	0.000	0.000	0.000	0.000	0.002	-11.088	3.322	-4.299	2.168
Hor-2 Tr-2 Sp	Syunik	0.000	0.000	0.000	0.000	0.000	0.000	0.000	0.000	0.000	0.000	0.000	0.000	1.000	0.000	-7.151	-12.184	-1.550	3.029	-1.478
Hor-2 Tr-2 Sp	Kelbadjar	0.000	0.000	0.000	0.000	0.000	0.000	0.000	0.000	1.000	0.000	0.000	0.000	0.000	0.000	-9.125	-10.250	1.449	3.704	-1.222
Hor-2 Tr-2 Sp	Kelbadjar	0.000	0.000	0.000	0.000	0.000	0.000	0.000	0.000	1.000	0.000	0.000	0.000	0.000	0.000	-9.060	-9.447	0.118	5.017	-1.655
Hor-2 Tr-2 Sp	Gegham 1	0.000	0.000	0.000	1.000	0.000	0.000	0.000	0.000	0.000	0.000	0.000	0.000	0.000	0.000	-1.595	-11.774	3.032	-4.484	3.633
Hor-2 Tr-2 Sp	Kelbadjar	0.000	0.000	0.000	0.000	0.000	0.000	0.000	0.000	1.000	0.000	0.000	0.000	0.000	0.000	-9.060	-8.053	-0.294	4.682	-1.601
Hor-2 Tr-2 Sp	Gegham 1	0.000	0.000	0.000	1.000	0.000	0.000	0.000	0.000	0.000	0.000	0.000	0.000	0.000	0.000	-0.262	-11.848	2.850	-4.201	1.214
Hor-2 Tr-2 Sp	Kelbadjar	0.000	0.000	0.000	0.000	0.000	0.000	0.000	0.000	1.000	0.000	0.000	0.000	0.000	0.000	-9.775	-9.642	-0.266	5.380	-1.513
Hor-2 Tr-2 Sp	Kelbadjar	0.000	0.000	0.000	0.000	0.000	0.000	0.000	0.000	1.000	0.000	0.000	0.000	0.000	0.000	-9.090	-8.864	0.298	3.677	-1.240
Hor-2 Tr-2 Sp	Gegham 1	0.000	0.000	0.000	1.000	0.000	0.000	0.000	0.000	0.000	0.000	0.000	0.000	0.000	0.000	-0.888	-10.856	3.379	-4.400	3.230
Hor-2 Tr-2 Sp	Gegham 1	0.000	0.000	0.000	1.000	0.000	0.000	0.000	0.000	0.000	0.000	0.000	0.000	0.000	0.000	-0.335	-11.864	3.225	-4.371	3.779
Hor-2 Tr-2 Sp	Syunik	0.000	0.000	0.000	0.000	0.000	0.000	0.000	0.000	0.000	0.000	0.000	0.000	1.000	0.000	-5.577	-11.729	-2.093	4.065	-1.509
Hor-2 Tr-2 Sp	Kelbadjar	0.000	0.000	0.000	0.000	0.000	0.000	0.000	0.000	1.000	0.000	0.000	0.000	0.000	0.000	-8.328	-10.283	0.668	3.653	-1.065
Hor-2 Tr-2 Sp	Gegham 1	0.000	0.000	0.000	1.000	0.000	0.000	0.000	0.000	0.000	0.000	0.000	0.000	0.000	0.000	-1.467	-12.916	2.381	-4.739	4.300
Hor-2 Tr-2 Sp	Arteni	0.000	1.000	0.000	0.000	0.000	0.000	0.000	0.000	0.000	0.000	0.000	0.000	0.000	0.000	-3.776	0.580	3.481	-3.912	-7.042
Hor-2 Tr-2 Sp	Gegham 1	0.000	0.000	0.000	1.000	0.000	0.000	0.000	0.000	0.000	0.000	0.000	0.000	0.000	0.000	-1.263	-11.834	3.132	-4.216	2.697
Hor-2 Tr-2 Sp	Kelbadjar	0.000	0.000	0.000	0.000	0.000	0.000	0.000	0.000	1.000	0.000	0.000	0.000	0.000	0.000	-8.667	-9.016	1.290	4.057	-2.442
Hor-2 Tr-2 Sp	Arteni	0.000	1.000	0.000	0.000	0.000	0.000	0.000	0.000	0.000	0.000	0.000	0.000	0.000	0.000	-5.387	1.850	5.927	-3.858	-7.770
Hor-2 Tr-2 Sp	Gegham 1	0.000	0.000	0.000	1.000	0.000	0.000	0.000	0.000	0.000	0.000	0.000	0.000	0.000	0.000	0.793	-11.667	2.000	-4.162	3.946
Hor-2 Tr-2 Sp	Gegham 1	0.000	0.000	0.000	1.000	0.000	0.000	0.000	0.000	0.000	0.000	0.000	0.000	0.000	0.000	-0.653	-10.667	3.646	-4.206	2.955
Hor-2 Tr-2 Sp	Kelbadjar	0.000	0.000	0.000	0.000	0.000	0.000	0.000	0.000	1.000	0.000	0.000	0.000	0.000	0.000	-8.868	-10.540	2.754	3.294	-1.547
Hor-2 Tr-2 Sp	Gegham 1	0.000	0.000	0.000	1.000	0.000	0.000	0.000	0.000	0.000	0.000	0.000	0.000	0.000	0.000	-2.233	-11.653	2.963	-4.124	3.200
Hor-2 Tr-2 Sp	Gegham 1	0.000	0.000	0.000	1.000	0.000	0.000	0.000	0.000	0.000	0.000	0.000	0.000	0.000	0.000	-0.850	-11.422	2.885	-4.409	2.672
Hor-2 Tr-2 Sp	Gegham 1	0.000	0.000	0.000	1.000	0.000	0.000	0.000	0.000	0.000	0.000	0.000	0.000	0.000	0.000	-1.432	-11.412	2.829	-4.361	3.933
Hor-2 Tr-2 Sp	Kelbadjar	0.000	0.000	0.000	0.000	0.000	0.000	0.000	0.000	1.000	0.000	0.000	0.000	0.000	0.000	-9.417	-9.928	0.535	4.950	-1.801
Hor-2 Tr-2 Sp	Kelbadjar	0.000	0.000	0.000	0.000	0.000	0.000	0.000	0.000	1.000	0.000	0.000	0.000	0.000	0.000	-9.345	-9.590	0.580	4.622	-1.562
Hor-2 Tr-2 Sp	Kelbadjar	0.000	0.000	0.000	0.000	0.000	0.000	0.000	0.000	1.000	0.000	0.000	0.000	0.000	0.000	-8.832	-9.311	1.730	3.788	-1.599
Hor-2 Tr-2 Sp	Hatis	0.000	0.000	0.000	0.000	0.000	0.000	1.000	0.000	0.000	0.000	0.000	0.000	0.000	0.000	17.240	4.173	-1.544	-2.066	-4.451
Hor-2 Tr-2 Sp	Gegham 1	0.000	0.000	0.000	1.000	0.000	0.000	0.000	0.000	0.000	0.000	0.000	0.000	0.000	0.000	-1.039	-10.658	3.310	-3.446	1.919
Hor-2 Tr-2 Sp	Gegham 1	0.000	0.000	0.000	1.000	0.000	0.000	0.000	0.000	0.000	0.000	0.000	0.000	0.000	0.000	0.789	-11.301	3.006	-4.555	5.983
Hor-2 Tr-2 Sp	Gegham 1	0.000	0.000	0.000	1.000	0.000	0.000	0.000	0.000	0.000	0.000	0.000	0.000	0.000	0.000	0.198	-9.709	3.696	-4.197	2.165
Hor-2 Tr-2 Sp	Gegham 1	0.000	0.000	0.000	1.000	0.000	0.000	0.000	0.000	0.000	0.000	0.000	0.000	0.000	0.000	-0.228	-10.702	3.718	-4.811	3.434
Hor-2 Tr-2 Sp	Syunik	0.000	0.000	0.000	0.000	0.000	0.000	0.000	0.000	0.000	0.000	0.000	0.000	1.000	0.000	-8.053	-12.700	-2.233	4.547	-1.454

Hor-2 Tr-2 Sp	Kelbadjar	0.000	0.000	0.000	0.000	0.000	0.000	0.000	0.000	1.000	0.000	0.000	0.000	0.000	0.000	-9.463	-9.357	0.118	4.476	-1.657
Hor-2 Tr-2 Sp	Kelbadjar	0.000	0.000	0.000	0.000	0.000	0.000	0.000	0.000	1.000	0.000	0.000	0.000	0.000	0.000	-9.861	-8.317	0.392	3.612	-1.833
Hor-2 Tr-2 Sp	Arteni	0.000	1.000	0.000	0.000	0.000	0.000	0.000	0.000	0.000	0.000	0.000	0.000	0.000	0.000	-1.868	2.111	4.781	-3.724	-7.927
Hor-2 Tr-2 Sp	Gegham 1	0.000	0.000	0.000	1.000	0.000	0.000	0.000	0.000	0.000	0.000	0.000	0.000	0.000	0.000	-1.235	-11.519	3.087	-4.468	3.854
Hor-2 Tr-2 Sp	Kelbadjar	0.000	0.000	0.000	0.000	0.000	0.000	0.000	0.000	1.000	0.000	0.000	0.000	0.000	0.000	-8.837	-9.707	1.195	4.216	-2.034
Hor-2 Tr-2 Sp	Syunik	0.000	0.000	0.000	0.000	0.000	0.000	0.000	0.000	0.000	0.000	0.000	0.000	1.000	0.000	-6.679	-12.047	-0.956	3.612	-1.398
Hor-2 Tr-2 Sp	Gegham 1	0.000	0.000	0.000	1.000	0.000	0.000	0.000	0.000	0.000	0.000	0.000	0.000	0.000	0.000	0.048	-11.321	3.974	-4.857	3.484
Hor-2 Tr-2 Sp	Gegham 1	0.000	0.000	0.000	1.000	0.000	0.000	0.000	0.000	0.000	0.000	0.000	0.000	0.000	0.000	-1.283	-10.992	2.623	-4.536	4.382
Hor-2 Tr-2 Sp	Syunik	0.000	0.000	0.000	0.000	0.000	0.000	0.000	0.000	0.000	0.000	0.000	0.000	1.000	0.000	-4.973	-12.723	-1.217	3.763	-0.402
Hor-2 Tr-2 Sp	Gegham 1	0.000	0.000	0.000	1.000	0.000	0.000	0.000	0.000	0.000	0.000	0.000	0.000	0.000	0.000	-0.965	-10.189	2.272	-4.156	2.843
Hor-2 Tr-2 Sp	Kelbadjar	0.000	0.000	0.000	0.000	0.000	0.000	0.000	0.000	1.000	0.000	0.000	0.000	0.000	0.000	-9.218	-9.126	-0.090	3.547	-1.766
Hor-2 Tr-2 Sp	Gegham 1	0.000	0.000	0.000	1.000	0.000	0.000	0.000	0.000	0.000	0.000	0.000	0.000	0.000	0.000	-0.774	-11.167	3.376	-4.169	3.728
Hor-2 Tr-2 Sp	Gegham 1	0.000	0.000	0.000	1.000	0.000	0.000	0.000	0.000	0.000	0.000	0.000	0.000	0.000	0.000	-1.435	-12.491	2.427	-3.862	3.766
Hor-2 Tr-2 Sp	Gegham 1	0.000	0.000	0.000	1.000	0.000	0.000	0.000	0.000	0.000	0.000	0.000	0.000	0.000	0.000	-1.036	-9.888	3.015	-4.157	3.721
Hor-2 Tr-2 Sp	Gegham 1	0.000	0.000	0.000	1.000	0.000	0.000	0.000	0.000	0.000	0.000	0.000	0.000	0.000	0.000	-1.251	-10.467	2.965	-4.104	2.292
Hor-2 Tr-2 Sp	Gegham 1	0.000	0.000	0.000	1.000	0.000	0.000	0.000	0.000	0.000	0.000	0.000	0.000	0.000	0.000	-0.179	-11.848	2.688	-4.860	4.850
Hor-2 Tr-2 Sp	Gegham 1	0.000	0.000	0.000	1.000	0.000	0.000	0.000	0.000	0.000	0.000	0.000	0.000	0.000	0.000	-0.776	-11.013	3.074	-4.445	2.335
Hor-2 Tr-2 Sp	Gegham 1	0.000	0.000	0.000	1.000	0.000	0.000	0.000	0.000	0.000	0.000	0.000	0.000	0.000	0.000	0.161	-9.936	3.020	-4.428	3.510
Hor-2 Tr-2 Sp	Gegham 1	0.000	0.000	0.000	1.000	0.000	0.000	0.000	0.000	0.000	0.000	0.000	0.000	0.000	0.000	1.782	-9.842	2.473	-4.334	6.242
Hor-2 Tr-2 Sp	Kelbadjar	0.000	0.000	0.000	0.000	0.000	0.000	0.000	0.000	1.000	0.000	0.000	0.000	0.000	0.000	-9.145	-10.565	2.138	4.235	-1.509
Hor-2 Tr-2 Sp	Syunik	0.000	0.000	0.000	0.000	0.000	0.000	0.000	0.000	0.000	0.000	0.000	0.000	1.000	0.000	-7.199	-13.069	-1.422	3.228	-1.141
Hor-2 Tr-2 Sp	Kelbadjar	0.000	0.000	0.000	0.000	0.000	0.000	0.000	0.000	1.000	0.000	0.000	0.000	0.000	0.000	-8.578	-9.461	-0.215	3.842	-1.553
Hor-2 Tr-2 Sp	Kelbadjar	0.000	0.000	0.000	0.000	0.000	0.000	0.000	0.000	1.000	0.000	0.000	0.000	0.000	0.000	-9.646	-8.743	-0.812	6.249	-1.980
Hor-2 Tr-2 Sp	Arteni	0.000	1.000	0.000	0.000	0.000	0.000	0.000	0.000	0.000	0.000	0.000	0.000	0.000	0.000	-4.771	2.462	5.493	-3.127	-6.019
Hor-2 Tr-2 Sp	Gegham 1	0.000	0.000	0.000	1.000	0.000	0.000	0.000	0.000	0.000	0.000	0.000	0.000	0.000	0.000	-0.910	-12.404	2.782	-4.307	4.827
Hor-2 Tr-2 Sp	Gegham 1	0.000	0.000	0.000	1.000	0.000	0.000	0.000	0.000	0.000	0.000	0.000	0.000	0.000	0.000	-1.392	-12.795	1.702	-4.687	6.692
Hor-2 Tr-2 Sp	Gegham 1	0.000	0.000	0.000	1.000	0.000	0.000	0.000	0.000	0.000	0.000	0.000	0.000	0.000	0.000	0.143	-10.918	4.089	-4.805	2.429
Hor-2 Tr-2 Sp	Gegham 1	0.000	0.000	0.000	1.000	0.000	0.000	0.000	0.000	0.000	0.000	0.000	0.000	0.000	0.000	-1.177	-11.161	2.568	-3.531	3.644
Hor-2 Tr-2 Sp	Gegham 1	0.000	0.000	0.000	1.000	0.000	0.000	0.000	0.000	0.000	0.000	0.000	0.000	0.000	0.000	-1.643	-11.158	3.304	-4.359	2.747
Hor-2 Tr-2 Sp	Gegham 1	0.000	0.000	0.000	1.000	0.000	0.000	0.000	0.000	0.000	0.000	0.000	0.000	0.000	0.000	-0.520	-11.278	3.151	-4.157	2.687
Hor-2 Tr-2 Sp	Gegham 1	0.000	0.000	0.000	1.000	0.000	0.000	0.000	0.000	0.000	0.000	0.000	0.000	0.000	0.000	-0.943	-11.477	3.160	-4.172	2.387
Hor-2 Tr-2 Sp	Gegham 1	0.000	0.000	0.000	1.000	0.000	0.000	0.000	0.000	0.000	0.000	0.000	0.000	0.000	0.000	-0.533	-11.123	3.208	-4.546	4.468
Hor-2 Tr-2 Sp	Gegham 1	0.000	0.000	0.000	1.000	0.000	0.000	0.000	0.000	0.000	0.000	0.000	0.000	0.000	0.000	-1.205	-11.515	2.951	-4.789	5.076
Hor-2 Tr-2 Sp	Gegham 1	0.000	0.000	0.000	1.000	0.000	0.000	0.000	0.000	0.000	0.000	0.000	0.000	0.000	0.000	-0.364	-11.054	3.120	-4.287	2.485
Hor-2 Tr-2 Sp	Gegham 1	0.000	0.000	0.000	1.000	0.000	0.000	0.000	0.000	0.000	0.000	0.000	0.000	0.000	0.000	-0.682	-10.515	3.193	-4.517	2.883
Hor-2 Tr-2 Sp	Gegham 1	0.000	0.000	0.000	1.000	0.000	0.000	0.000	0.000	0.000	0.000	0.000	0.000	0.000	0.000	-0.364	-10.666	2.997	-4.489	3.249
Hor-2 Tr-2 Sp	Gegham 1	0.000	0.000	0.000	1.000	0.000	0.000	0.000	0.000	0.000	0.000	0.000	0.000	0.000	0.000	0.015	-9.048	4.871	-4.761	3.769
Hor-2 Tr-2 Sp	Gegham 1	0.000	0.000	0.000	1.000	0.000	0.000	0.000	0.000	0.000	0.000	0.000	0.000	0.000	0.000	-0.475	-10.478	4.399	-4.233	2.632
Hor-2 Tr-2 Sp	Gegham 1	0.000	0.000	0.000	1.000	0.000	0.000	0.000	0.000	0.000	0.000	0.000	0.000	0.000	0.000	1.478	-8.411	3.713	-4.610	4.315
Hor-2 Tr-2 Sp	Gegham 2	0.000	0.011	0.000	0.000	0.989	0.000	0.000	0.000	0.000	0.000	0.000	0.000	0.000	0.000	-1.492	5.622	5.414	-2.399	-4.634
Hor-2 Tr-2 Sp	Kelbadjar	0.000	0.000	0.000	0.000	0.000	0.000	0.000	0.000	1.000	0.000	0.000	0.000	0.000	0.000	-10.351	-9.568	0.249	5.302	-2.368
Hor-2 Tr-2 Sp	Kelbadjar	0.000	0.000	0.000	0.000	0.000	0.000	0.000	0.000	1.000	0.000	0.000	0.000	0.000	0.000	-9.145	-9.996	-0.613	4.959	-1.829
Hor-2 Tr-2 Sp	Gegham 1	0.000	0.000	0.000	1.000	0.000	0.000	0.000	0.000	0.000	0.000	0.000	0.000	0.000	0.000	-0.661	-12.582	2.761	-4.662	6.484
Hor-2 Tr-2 Sp	Kelbadjar	0.000	0.000	0.000	0.000	0.000	0.000	0.000	0.000	1.000	0.000	0.000	0.000	0.000	0.000	-7.872	-8.712	0.153	3.061	-1.709
Hor-2 Tr-2 Sp	Gegham 1	0.000	0.000	0.000	1.000	0.000	0.000	0.000	0.000	0.000	0.000	0.000	0.000	0.000	0.000	-0.706	-11.805	3.182	-3.661	2.345
Tr-2 Hor-0 U	Kelbadjar	0.000	0.000	0.000	0.000	0.000	0.000	0.000	0.000	1.000	0.000	0.000	0.000	0.000	0.000	-8.088	-9.482	1.439	2.579	-1.553
Tr-2 Hor-0 U	Gegham 1	0.000	0.000	0.000	1.000	0.000	0.000	0.000	0.000	0.000	0.000	0.000	0.000	0.000	0.000	-0.026	-11.559	2.945	-4.316	2.945
Tr-2 Hor-0 U	Gegham 1	0.000	0.000	0.000	1.000	0.000	0.000	0.000	0.000	0.000	0.000	0.000	0.000	0.000	0.000	-0.023	-11.143	3.566	-4.377	3.158
Tr-2 Hor-0 U	Gegham 1	0.000	0.000	0.000	1.000	0.000	0.000	0.000	0.000	0.000	0.000	0.000	0.000	0.000	0.000	-0.565	-12.429	1.836	-4.554	4.906
Tr-2 Hor-0 U	Gegham 1	0.000	0.000	0.000	1.000	0.000	0.000	0.000	0.000	0.000	0.000	0.000	0.000	0.000	0.000	0.399	-9.682	3.918	-4.745	3.244
Tr-2 Hor-0 U	Gegham 1	0.000	0.000	0.000	1.000	0.000	0.000	0.000	0.000	0.000	0.000	0.000	0.000	0.000	0.000	-0.260	-9.806	3.980	-4.863	6.571
Tr-2 Hor-0 U	Kelbadjar	0.000	0.000	0.000	0.000	0.000	0.000	0.000	0.000	1.000	0.000	0.000	0.000	0.000	0.000	-8.705	-9.576	2.474	3.937	-1.929
Tr-2 Hor-0 U	Gegham 1	0.000	0.000	0.000	1.000	0.000	0.000	0.000	0.000	0.000	0.000	0.000	0.000	0.000	0.000	0.449	-9.199	3.668	-3.971	1.941
Tr-2 Hor-0 U	Gegham 1	0.000	0.000	0.000	1.000	0.000	0.000	0.000	0.000	0.000	0.000	0.000	0.000	0.000	0.000	-0.839	-12.231	2.440	-4.630	3.859
Tr-2 Hor-0 U	Kelbadjar	0.000	0.000	0.000	0.000	0.000	0.000	0.000	0.000	1.000	0.000	0.000	0.000	0.000	0.000	-4.948	-10.229	-0.591	3.620	-1.295
Tr-2 Hor-0 U	Syunik	0.000	0.000	0.000	0.000	0.000	0.000	0.000	0.000	0.000	0.000	0.000	0.000	1.000	0.000	-5.813	-12.246	-1.945	3.325	-0.429
Tr-2 Hor-0 U	Kelbadjar	0.000	0.000	0.000	0.000	0.000	0.000	0.000	0.000	1.000	0.000	0.000	0.000	0.000	0.000	-7.441	-8.791	1.332	3.347	-1.806
Tr-2 Hor-0 U	Gegham 1	0.000	0.000	0.000	1.000	0.000	0.000	0.000	0.000	0.000	0.000	0.000	0.000	0.000	0.000	-0.787	-9.610	5.056	-4.970	3.058
Tr-2 Hor-0 U	Kelbadjar	0.000	0.000	0.000	0.000	0.000	0.000	0.000	0.000	1.000	0.000	0.000	0.000	0.000	0.000	-8.920	-9.343	1.637	3.044	-1.454
Tr-2 Hor-0 U	Kelbadjar	0.000	0.000	0.000	0.000	0.000	0.000	0.000	0.000	1.000	0.000	0.000	0.000	0.000	0.000	-9.686	-9.081	1.306	3.109	-1.840
Tr-2 Hor-0 U	Kelbadjar	0.000	0.000	0.000	0.000	0.000	0.000	0.000	0.000	1.000	0.000	0.000	0.000	0.000	0.000	-10.408	-10.332	1.009	4.743	-1.683
Tr.2 UN-A2-3	Gegham 1	0.000	0.000	0.000	1.000	0.000	0.000	0.000	0.000	0.000	0.000	0.000	0.000	0.000	0.000	-1.035	-9.841	3.769	-4.625	3.341
Tr.2 UN-A2-3	Kelbadjar	0.000	0.000	0.000	0.000	0.000	0.000	0.000	0.000	1.000	0.000	0.000	0.000	0.000	0.000	-9.621	-10.071	2.003	4.611	-1.876
Tr.2 UN-A2-3	Gegham 1	0.000	0.000	0.000	1.000	0.000	0.000	0.000	0.000	0.000	0.000	0.000	0.000	0.000	0.000	-0.770	-11.083	3.267	-4.886	4.724
Tr.2 UN-A2-3	Gegham 1	0.000	0.000	0.000	1.000	0.000	0.000	0.000	0.000	0.000	0.000	0.000	0.000	0.000	0.000	-1.176	-11.945	3.268	-5.394	6.841
Tr.2 UN-A2-3	Kelbadjar	0.000	0.000	0.000	0.000	0.000	0.000	0.000	0.000	1.000	0.000	0.000	0.000	0.000	0.000	-9.129	-9.973	0.751	3.708	-1.230
Tr.2 UN-A2-3	Gegham 1	0.000	0.000	0.000	1.000	0.000	0.000	0.000	0.000	0.000	0.000	0.000	0.000	0.000	0.000	-0.418	-9.830	3.040	-4.292	3.404

Tr.2 UN-A2-3	Kelbadjar	0.000	0.000	0.000	0.000	0.000	0.000	0.000	0.000	1.000	0.000	0.000	0.000	0.000	0.000	-10.271	-10.477	-0.381	4.654	-1.701
Tr.2 UN-A2-3	Syunik	0.000	0.000	0.000	0.000	0.000	0.000	0.000	0.000	0.000	0.000	0.000	0.000	1.000	0.000	-7.109	-12.155	-2.696	3.666	-1.325
Tr.2 UN-A2-3	Kelbadjar	0.000	0.000	0.000	0.000	0.000	0.000	0.000	0.000	1.000	0.000	0.000	0.000	0.000	0.000	-9.861	-9.602	0.397	4.182	-1.855
Tr.2 UN-A2-3	Gegham 1	0.000	0.000	0.000	1.000	0.000	0.000	0.000	0.000	0.000	0.000	0.000	0.000	0.000	0.000	-0.194	-10.095	4.094	-4.743	2.281
Tr.2 UN-A2-3	Kelbadjar	0.000	0.000	0.000	0.000	0.000	0.000	0.000	0.000	1.000	0.000	0.000	0.000	0.000	0.000	-9.235	-10.064	0.922	4.372	-1.442
Tr.2 UN-A2-3	Arteni	0.000	0.993	0.000	0.000	0.007	0.000	0.000	0.000	0.000	0.000	0.000	0.000	0.000	0.000	-0.958	4.073	4.486	-2.351	-4.189
Tr.2 UN-A2-3	Kelbadjar	0.000	0.000	0.000	0.000	0.000	0.000	0.000	0.000	1.000	0.000	0.000	0.000	0.000	0.000	-10.038	-8.756	-0.560	5.225	-1.724
Tr.2 UN-A2-3	Kelbadjar	0.000	0.000	0.000	0.000	0.000	0.000	0.000	0.000	1.000	0.000	0.000	0.000	0.000	0.000	-9.390	-9.312	1.437	5.209	-2.393
Tr.2 UN-A2-3	Kelbadjar	0.000	0.000	0.000	0.000	0.000	0.000	0.000	0.000	1.000	0.000	0.000	0.000	0.000	0.000	-8.818	-10.547	-0.118	5.678	-1.938
Tr.2 UN-A2-3	Kelbadjar	0.000	0.000	0.000	0.000	0.000	0.000	0.000	0.000	1.000	0.000	0.000	0.000	0.000	0.000	-9.767	-9.678	1.281	4.001	-1.650
Tr.2 UN-A2-3	Gegham 1	0.000	0.000	0.000	1.000	0.000	0.000	0.000	0.000	0.000	0.000	0.000	0.000	0.000	0.000	-1.511	-12.896	2.748	-4.193	3.420
Tr.2 UN-A2-3	Gegham 1	0.000	0.000	0.000	1.000	0.000	0.000	0.000	0.000	0.000	0.000	0.000	0.000	0.000	0.000	-1.685	-11.044	4.258	-4.534	5.253
Tr.2 UN-A2-3	Gegham 1	0.000	0.000	0.000	1.000	0.000	0.000	0.000	0.000	0.000	0.000	0.000	0.000	0.000	0.000	-0.749	-12.326	3.161	-4.719	5.092
Tr.2 UN-A2-3	Gegham 1	0.000	0.000	0.000	1.000	0.000	0.000	0.000	0.000	0.000	0.000	0.000	0.000	0.000	0.000	-0.634	-10.386	3.604	-4.615	3.507
Tr.2 UN-A2-3	Kelbadjar	0.000	0.000	0.000	0.000	0.000	0.000	0.000	0.000	1.000	0.000	0.000	0.000	0.000	0.000	-9.676	-9.258	1.162	4.008	-2.073
Tr.2 UN-A2-3	Kelbadjar	0.000	0.000	0.000	0.000	0.000	0.000	0.000	0.000	1.000	0.000	0.000	0.000	0.000	0.000	-9.307	-10.133	0.596	3.902	-1.695
Tr.2 UN-A2-3	Syunik	0.000	0.000	0.000	0.000	0.000	0.000	0.000	0.000	0.000	0.000	0.000	0.000	1.000	0.000	-8.128	-14.049	-4.025	4.925	-0.805
Tr.2 UN-A2-3	Gegham 1	0.000	0.000	0.000	1.000	0.000	0.000	0.000	0.000	0.000	0.000	0.000	0.000	0.000	0.000	-1.288	-12.430	2.710	-4.513	5.899
Tr.2 UN-A2-3	Gegham 1	0.000	0.000	0.000	1.000	0.000	0.000	0.000	0.000	0.000	0.000	0.000	0.000	0.000	0.000	-0.814	-11.222	2.904	-4.415	3.847
Tr.2 UN-A2-3	Gegham 1	0.000	0.000	0.000	1.000	0.000	0.000	0.000	0.000	0.000	0.000	0.000	0.000	0.000	0.000	-1.527	-12.613	2.526	-4.832	5.194
Tr.2 UN-A2-3	Kelbadjar	0.000	0.000	0.000	0.000	0.000	0.000	0.000	0.000	1.000	0.000	0.000	0.000	0.000	0.000	-9.964	-9.598	-1.262	5.730	-1.697
Tr.2 UN-A2-3	Gegham 1	0.000	0.000	0.000	1.000	0.000	0.000	0.000	0.000	0.000	0.000	0.000	0.000	0.000	0.000	-0.824	-10.927	2.926	-4.212	4.497
Tr.2 UN-A2-3	Kelbadjar	0.000	0.000	0.000	0.000	0.000	0.000	0.000	0.000	1.000	0.000	0.000	0.000	0.000	0.000	-9.728	-9.914	0.045	5.204	-1.852
Tr.2 UN-A2-3	Kelbadjar	0.000	0.000	0.000	0.000	0.000	0.000	0.000	0.000	0.584	0.000	0.000	0.000	0.416	0.000	-10.392	-10.472	-2.062	5.426	-1.697
Tr.2 UN-A2-3	Gegham 1	0.000	0.000	0.000	1.000	0.000	0.000	0.000	0.000	0.000	0.000	0.000	0.000	0.000	0.000	-1.030	-8.913	4.214	-4.591	4.455
Tr.2 UN-A2-3	Kelbadjar	0.000	0.000	0.000	0.000	0.000	0.000	0.000	0.000	1.000	0.000	0.000	0.000	0.000	0.000	-9.174	-8.364	0.976	3.294	-1.847
Tr.2 UN-A2-3	Kelbadjar	0.000	0.000	0.000	0.000	0.000	0.000	0.000	0.000	1.000	0.000	0.000	0.000	0.000	0.000	-10.100	-10.460	0.243	5.605	-1.607
Tr.2 UN-A2-3	Gegham 1	0.000	0.000	0.000	1.000	0.000	0.000	0.000	0.000	0.000	0.000	0.000	0.000	0.000	0.000	0.191	-11.678	3.026	-5.169	5.240
Tr.2 UN-A2-3	Kelbadjar	0.000	0.000	0.000	0.000	0.000	0.000	0.000	0.000	1.000	0.000	0.000	0.000	0.000	0.000	-9.467	-9.266	1.069	4.583	-2.203
Tr.2 UN-A2-3	Kelbadjar	0.000	0.000	0.000	0.000	0.000	0.000	0.000	0.000	1.000	0.000	0.000	0.000	0.000	0.000	-10.336	-10.153	-1.294	6.385	-1.878
Tr.2 UN-A2-3	Kelbadjar	0.000	0.000	0.000	0.000	0.000	0.000	0.000	0.000	1.000	0.000	0.000	0.000	0.000	0.000	-10.938	-10.205	-0.659	5.759	-1.832
Tr.2 UN-A2-3	Gegham 1	0.000	0.000	0.000	1.000	0.000	0.000	0.000	0.000	0.000	0.000	0.000	0.000	0.000	0.000	-0.618	-9.816	3.788	-4.788	5.088
Tr.2 UN-A2-3	Gegham 1	0.000	0.000	0.000	1.000	0.000	0.000	0.000	0.000	0.000	0.000	0.000	0.000	0.000	0.000	-2.093	-11.362	2.419	-4.380	6.941
Tr.2 UN.C1 H	Kelbadjar	0.000	0.000	0.000	0.000	0.000	0.000	0.000	0.000	1.000	0.000	0.000	0.000	0.000	0.000	-10.250	-9.687	1.357	3.894	-1.661
Tr.2 UN.C1 H	Gegham 1	0.000	0.000	0.000	1.000	0.000	0.000	0.000	0.000	0.000	0.000	0.000	0.000	0.000	0.000	0.455	-11.243	3.793	-4.673	4.317
Tr.2 UN.C1 H	Gegham 1	0.000	0.000	0.000	1.000	0.000	0.000	0.000	0.000	0.000	0.000	0.000	0.000	0.000	0.000	-0.276	-11.541	3.028	-4.601	4.024
Tr.2 UN.C1 H	Gegham 1	0.000	0.000	0.000	1.000	0.000	0.000	0.000	0.000	0.000	0.000	0.000	0.000	0.000	0.000	-1.919	-11.150	2.561	-3.543	2.146
Tr.2 UN.C1 H	Gegham 1	0.000	0.000	0.000	1.000	0.000	0.000	0.000	0.000	0.000	0.000	0.000	0.000	0.000	0.000	4.350	-10.532	2.801	-4.365	5.139
Trench-2 H3	Gegham 1	0.000	0.000	0.000	1.000	0.000	0.000	0.000	0.000	0.000	0.000	0.000	0.000	0.000	0.000	-1.440	-11.174	3.432	-4.304	2.082
Trench-2 H3	Gegham 1	0.000	0.000	0.000	1.000	0.000	0.000	0.000	0.000	0.000	0.000	0.000	0.000	0.000	0.000	-1.761	-10.597	3.301	-4.446	2.413
Trench-2 H3	Gegham 1	0.000	0.000	0.000	1.000	0.000	0.000	0.000	0.000	0.000	0.000	0.000	0.000	0.000	0.000	-1.076	-10.942	3.109	-4.720	4.841
Trench-2 H3	Gegham 1	0.000	0.000	0.000	1.000	0.000	0.000	0.000	0.000	0.000	0.000	0.000	0.000	0.000	0.000	-0.713	-12.566	3.341	-4.079	4.304
Trench-2 H3	Gegham 1	0.000	0.000	0.000	1.000	0.000	0.000	0.000	0.000	0.000	0.000	0.000	0.000	0.000	0.000	-0.607	-11.431	2.842	-4.358	4.026
Trench-2 H3	Kelbadjar	0.000	0.000	0.000	0.000	0.000	0.000	0.000	0.000	1.000	0.000	0.000	0.000	0.000	0.000	-9.130	-8.849	0.985	3.014	-1.767
Trench-2 H3	Gegham 1	0.000	0.000	0.000	1.000	0.000	0.000	0.000	0.000	0.000	0.000	0.000	0.000	0.000	0.000	0.719	-10.726	3.003	-4.240	4.377
Trench-2 H3	Syunik	0.000	0.000	0.000	0.000	0.000	0.000	0.000	0.000	0.000	0.000	0.000	0.000	1.000	0.000	-12.209	-9.336	-2.383	6.463	-1.970
Tr.2 UN A2-4	Kelbadjar	0.000	0.000	0.000	0.000	0.000	0.000	0.000	0.000	1.000	0.000	0.000	0.000	0.000	0.000	-10.093	-10.181	-0.998	4.614	-1.864
Tr.2 UN A2-4	Gegham 1	0.000	0.000	0.000	1.000	0.000	0.000	0.000	0.000	0.000	0.000	0.000	0.000	0.000	0.000	0.362	-12.290	3.508	-4.506	3.704
Tr-2 Hor-0 U	Syunik	0.000	0.000	0.000	0.000	0.000	0.000	0.000	0.000	0.204	0.000	0.000	0.000	0.796	0.000	-5.837	-12.043	-1.044	3.209	-0.977
Tr-2 Hor-0 U	Gegham 1	0.000	0.000	0.000	1.000	0.000	0.000	0.000	0.000	0.000	0.000	0.000	0.000	0.000	0.000	-1.057	-13.180	2.525	-4.134	3.080
Tr-2 Hor-0 U	Kelbadjar	0.000	0.000	0.000	0.000	0.000	0.000	0.000	0.000	1.000	0.000	0.000	0.000	0.000	0.000	-8.261	-10.307	0.106	4.273	-1.193
Tr-2 Hor-0 U	Arteni	0.000	1.000	0.000	0.000	0.000	0.000	0.000	0.000	0.000	0.000	0.000	0.000	0.000	0.000	-3.057	1.480	4.952	-3.203	-6.857
Tr-2 Hor-0 U	Gegham 1	0.000	0.000	0.000	1.000	0.000	0.000	0.000	0.000	0.000	0.000	0.000	0.000	0.000	0.000	-0.122	-11.457	3.202	-4.991	5.905
Tr-2 Hor-0 U	Gegham 1	0.000	0.000	0.000	1.000	0.000	0.000	0.000	0.000	0.000	0.000	0.000	0.000	0.000	0.000	3.162	-10.227	3.135	-3.712	2.690
Tr-2 Hor-0 U	Gegham 1	0.000	0.000	0.000	1.000	0.000	0.000	0.000	0.000	0.000	0.000	0.000	0.000	0.000	0.000	-0.143	-10.360	3.463	-4.487	4.398
Tr-2 Hor-0 U	Gegham 1	0.000	0.000	0.000	1.000	0.000	0.000	0.000	0.000	0.000	0.000	0.000	0.000	0.000	0.000	0.532	-9.619	3.741	-4.515	4.970
Tr.2 UN-A4	Gegham 1	0.000	0.000	0.000	1.000	0.000	0.000	0.000	0.000	0.000	0.000	0.000	0.000	0.000	0.000	-1.405	-12.294	2.720	-4.281	2.318
Tr.2 UN-A4	Gegham 1	0.000	0.000	0.000	1.000	0.000	0.000	0.000	0.000	0.000	0.000	0.000	0.000	0.000	0.000	-0.070	-10.276	2.379	-3.130	2.129
Tr.2 UN-A4	Gegham 1	0.000	0.000	0.000	1.000	0.000	0.000	0.000	0.000	0.000	0.000	0.000	0.000	0.000	0.000	0.033	-12.809	2.484	-4.288	3.857
Tr.2 UN-A4	Kelbadjar	0.000	0.000	0.000	0.000	0.000	0.000	0.000	0.000	1.000	0.000	0.000	0.000	0.000	0.000	-9.016	-10.106	1.628	4.168	-1.602
Tr.2 UN-A4	Gegham 1	0.000	0.000	0.000	1.000	0.000	0.000	0.000	0.000	0.000	0.000	0.000	0.000	0.000	0.000	-0.034	-11.560	2.328	-4.545	3.700
Tr.2 UN-A4	Gegham 1	0.000	0.000	0.000	1.000	0.000	0.000	0.000	0.000	0.000	0.000	0.000	0.000	0.000	0.000	-1.021	-10.851	3.967	-4.615	3.347
Tr.2 UN-A4	Kelbadjar	0.000	0.000	0.000	0.000	0.000	0.000	0.000	0.000	1.000	0.000	0.000	0.000	0.000	0.000	-9.904	-10.137	0.062	4.418	-1.258
Tr.2 UN-A4	Kelbadjar	0.000	0.000	0.000	0.000	0.000	0.000	0.000	0.000	1.000	0.000	0.000	0.000	0.000	0.000	-8.892	-9.490	2.085	4.712	-1.815
Tr.2 UN-A4	Gegham 1	0.000	0.000	0.000	1.000	0.000	0.000	0.000	0.000	0.000	0.000	0.000	0.000	0.000	0.000	-2.034	-11.312	3.278	-4.385	5.179
Tr.2 UN-A4	Gegham 1	0.000	0.000	0.000	1.000	0.000	0.000	0.000	0.000	0.000	0.000	0.000	0.000	0.000	0.000	-0.420	-10.529	3.761	-4.369	4.542
Tr.2 UN-A4	Kelbadjar	0.000	0.000	0.000	0.000	0.000	0.000	0.000	0.000	1.000	0.000	0.000	0.000	0.000	0.000	-8.854	-9.283	2.741	4.149	-2.394

Tr.2 UN-A4	Kelbadjar	0.000	0.000	0.000	0.000	0.000	0.000	0.000	0.000	1.000	0.000	0.000	0.000	0.000	0.000	-9.824	-9.795	-0.338	6.124	-1.917
TR.2 UN.C1 I	Gegham 1	0.000	0.000	0.000	1.000	0.000	0.000	0.000	0.000	0.000	0.000	0.000	0.000	0.000	0.000	0.971	-11.494	2.568	-4.938	4.468
TR.2 UN.C1 I	Gegham 1	0.000	0.000	0.000	1.000	0.000	0.000	0.000	0.000	0.000	0.000	0.000	0.000	0.000	0.000	-0.477	-10.710	3.050	-4.671	3.862
TR.2 UN.C1 I	Gutansar	0.000	0.000	0.000	0.000	0.000	1.000	0.000	0.000	0.000	0.000	0.000	0.000	0.000	0.000	4.039	0.178	12.843	16.322	-8.323
TR.2 UN.C1 I	Syunik	0.000	0.000	0.000	0.000	0.000	0.000	0.000	0.000	0.000	0.000	0.000	0.000	1.000	0.000	-6.217	-12.010	-1.159	3.446	-1.509
TR.2 UN.C1 I	Hatis	0.000	0.000	0.000	0.000	0.000	0.000	1.000	0.000	0.000	0.000	0.000	0.000	0.000	0.000	18.068	2.409	-0.308	-0.866	-3.657
TR.2 UN.C1 I	Gegham 1	0.000	0.000	0.000	1.000	0.000	0.000	0.000	0.000	0.000	0.000	0.000	0.000	0.000	0.000	-0.137	-11.084	3.430	-4.583	4.027
TR.2 UN.C1 I	Syunik	0.000	0.000	0.000	0.000	0.000	0.000	0.000	0.000	0.000	0.000	0.000	0.000	1.000	0.000	-6.821	-11.453	-1.728	3.683	-1.177
TR.2 UN.C1 I	Gutansar	0.000	0.000	0.000	0.000	0.000	1.000	0.000	0.000	0.000	0.000	0.000	0.000	0.000	0.000	5.288	0.278	12.242	15.409	-8.389
TR.2 UN.C1 I	Kelbadjar	0.000	0.000	0.000	0.000	0.000	0.000	0.000	0.000	1.000	0.000	0.000	0.000	0.000	0.000	-10.000	-9.902	-0.480	5.226	-2.037
TR.2 UN-21 T	Gegham 1	0.000	0.000	0.000	1.000	0.000	0.000	0.000	0.000	0.000	0.000	0.000	0.000	0.000	0.000	-0.787	-11.794	2.929	-4.530	2.909
TR.2 UN-21 T	Syunik	0.000	0.000	0.000	0.000	0.000	0.000	0.000	0.000	0.001	0.000	0.000	0.000	0.999	0.000	-7.530	-12.739	0.101	2.949	-1.672
TR.2 UN-21 T	Gegham 1	0.000	0.000	0.000	1.000	0.000	0.000	0.000	0.000	0.000	0.000	0.000	0.000	0.000	0.000	-1.085	-11.907	3.301	-4.906	5.191
TR.2 UN-21 T	Kelbadjar	0.000	0.000	0.000	0.000	0.000	0.000	0.000	0.000	1.000	0.000	0.000	0.000	0.000	0.000	-9.039	-10.344	0.607	2.997	-1.105
TR.2 UN-21 T	Gegham 1	0.000	0.000	0.000	1.000	0.000	0.000	0.000	0.000	0.000	0.000	0.000	0.000	0.000	0.000	-0.387	-9.257	3.932	-4.511	5.328
Tr-2 Hor4 Sp	Syunik	0.000	0.000	0.000	0.000	0.000	0.000	0.000	0.000	0.000	0.000	0.000	0.000	1.000	0.000	-7.938	-13.507	-1.969	2.533	-0.901
Tr-2 Hor4 Sp	Gegham 1	0.000	0.000	0.000	1.000	0.000	0.000	0.000	0.000	0.000	0.000	0.000	0.000	0.000	0.000	-0.480	-10.394	3.681	-4.623	2.659
Tr-2 Hor4 Sp	Gegham 1	0.000	0.000	0.000	1.000	0.000	0.000	0.000	0.000	0.000	0.000	0.000	0.000	0.000	0.000	-0.932	-11.335	3.176	-4.497	2.243
Tr-2 Hor4 Sp	Gegham 1	0.000	0.000	0.000	1.000	0.000	0.000	0.000	0.000	0.000	0.000	0.000	0.000	0.000	0.000	-0.862	-11.529	2.209	-4.489	3.090
Tr-2 Hor4 Sp	Kelbadjar	0.000	0.000	0.000	0.000	0.000	0.000	0.000	0.000	0.999	0.000	0.000	0.000	0.001	0.000	-6.236	-10.537	-0.928	2.244	-1.291
Tr-2 Hor4 Sp	Kelbadjar	0.000	0.000	0.000	0.000	0.000	0.000	0.000	0.000	1.000	0.000	0.000	0.000	0.000	0.000	-9.796	-9.964	0.077	4.243	-1.158
Tr-2 Hor4 Sp	Gegham 1	0.000	0.000	0.000	1.000	0.000	0.000	0.000	0.000	0.000	0.000	0.000	0.000	0.000	0.000	0.304	-8.942	4.326	-4.814	3.961
Tr-2 Hor4 Sp	Gegham 1	0.000	0.000	0.000	1.000	0.000	0.000	0.000	0.000	0.000	0.000	0.000	0.000	0.000	0.000	-0.993	-11.753	3.199	-4.772	3.849
Tr-2 Hor4 Sp	Gegham 1	0.000	0.000	0.000	1.000	0.000	0.000	0.000	0.000	0.000	0.000	0.000	0.000	0.000	0.000	-2.087	-9.700	4.002	-4.514	7.059
Tr-2 Hor4 Sp	Gegham 1	0.000	0.000	0.000	1.000	0.000	0.000	0.000	0.000	0.000	0.000	0.000	0.000	0.000	0.000	-0.678	-10.534	4.076	-4.364	3.542
Tr-2 Hor4 Sp	Gegham 1	0.000	0.000	0.000	1.000	0.000	0.000	0.000	0.000	0.000	0.000	0.000	0.000	0.000	0.000	-0.319	-11.522	3.305	-5.069	4.563
Tr-2 Hor4 Sp	Gegham 1	0.000	0.000	0.000	1.000	0.000	0.000	0.000	0.000	0.000	0.000	0.000	0.000	0.000	0.000	-1.129	-11.937	2.847	-4.592	4.730
Tr-2 Hor4 Sp	Syunik	0.000	0.000	0.000	0.000	0.000	0.000	0.000	0.000	0.000	0.000	0.000	0.000	1.000	0.000	-6.986	-13.135	-2.940	6.564	-1.751
Tr-2 Hor4 Sp	Syunik	0.000	0.000	0.000	0.000	0.000	0.000	0.000	0.000	0.000	0.000	0.000	0.000	1.000	0.000	-5.752	-12.909	-2.824	5.184	-1.868
Tr-2 Hor4 Sp	Syunik	0.000	0.000	0.000	0.000	0.000	0.000	0.000	0.000	0.000	0.000	0.000	0.000	1.000	0.000	-7.412	-13.431	-2.149	4.003	-1.101
TR.2 UN-A1	Kelbadjar	0.000	0.000	0.000	0.000	0.000	0.000	0.000	0.000	1.000	0.000	0.000	0.000	0.000	0.000	-9.226	-11.431	0.735	5.730	-1.431
TR.2 UN-A1	Kelbadjar	0.000	0.000	0.000	0.000	0.000	0.000	0.000	0.000	1.000	0.000	0.000	0.000	0.000	0.000	-9.339	-9.974	-0.216	4.417	-1.847
TR.2 UN-A1	Kelbadjar	0.000	0.000	0.000	0.000	0.000	0.000	0.000	0.000	1.000	0.000	0.000	0.000	0.000	0.000	-10.380	-9.992	0.480	3.918	-1.686
TR.2 UN-A1	Gegham 1	0.000	0.000	0.000	1.000	0.000	0.000	0.000	0.000	0.000	0.000	0.000	0.000	0.000	0.000	-0.645	-10.387	4.124	-4.868	4.415
TR.2 UN-A1	Gegham 1	0.000	0.000	0.000	1.000	0.000	0.000	0.000	0.000	0.000	0.000	0.000	0.000	0.000	0.000	-0.187	-10.161	4.887	-5.030	5.079
TR.2 UN-C! T	Syunik	0.000	0.000	0.000	0.000	0.000	0.000	0.000	0.000	0.000	0.000	0.000	0.000	1.000	0.000	-8.019	-12.942	-2.515	5.259	-1.576
TR.2 UN-A5 T	Kelbadjar	0.000	0.000	0.000	0.000	0.000	0.000	0.000	0.000	1.000	0.000	0.000	0.000	0.000	0.000	-8.720	-8.530	2.399	3.539	-1.905
TR.2 UN.A2-	Gegham 1	0.000	0.000	0.000	1.000	0.000	0.000	0.000	0.000	0.000	0.000	0.000	0.000	0.000	0.000	-1.548	-10.134	3.303	-4.341	1.275
TR.2 UN.A2-	Kelbadjar	0.000	0.000	0.000	0.000	0.000	0.000	0.000	0.000	1.000	0.000	0.000	0.000	0.000	0.000	-8.716	-9.591	0.114	2.881	-0.714
TR2 UN.A4 H	Kelbadjar	0.000	0.000	0.000	0.000	0.000	0.000	0.000	0.000	1.000	0.000	0.000	0.000	0.000	0.000	-9.001	-9.695	0.824	3.088	-1.348
TR2 UN.A4 H	Gegham 1	0.000	0.000	0.000	1.000	0.000	0.000	0.000	0.000	0.000	0.000	0.000	0.000	0.000	0.000	-0.422	-9.991	3.383	-4.898	4.935
TR2 UN.A4 H	Gegham 1	0.000	0.000	0.000	1.000	0.000	0.000	0.000	0.000	0.000	0.000	0.000	0.000	0.000	0.000	-0.402	-9.665	2.568	-4.640	5.614
TR.2 UN-A2-	Kelbadjar	0.000	0.000	0.000	0.000	0.000	0.000	0.000	0.000	0.818	0.000	0.000	0.000	0.182	0.000	-11.338	-10.235	-1.976	7.309	-2.598
TR.2 UN-A2-	Gegham 1	0.000	0.000	0.000	1.000	0.000	0.000	0.000	0.000	0.000	0.000	0.000	0.000	0.000	0.000	0.405	-9.147	4.061	-4.838	3.613
TR.2 UN-A2-	Kelbadjar	0.000	0.000	0.000	0.000	0.000	0.000	0.000	0.000	1.000	0.000	0.000	0.000	0.000	0.000	-7.842	-9.065	0.491	4.435	-0.897
TR.2 UN-A2-	Gegham 1	0.000	0.000	0.000	1.000	0.000	0.000	0.000	0.000	0.000	0.000	0.000	0.000	0.000	0.000	-0.993	-10.513	3.750	-4.693	3.725
TR.2 UN-A1-	Gegham 1	0.000	0.000	0.000	1.000	0.000	0.000	0.000	0.000	0.000	0.000	0.000	0.000	0.000	0.000	-1.270	-11.822	3.262	-3.988	4.058
TR.2 UN-A1-	Gegham 1	0.000	0.000	0.000	1.000	0.000	0.000	0.000	0.000	0.000	0.000	0.000	0.000	0.000	0.000	0.626	-10.167	3.637	-5.110	3.784
Tr-2 Hor4 Sp	Gegham 1	0.000	0.000	0.000	1.000	0.000	0.000	0.000	0.000	0.000	0.000	0.000	0.000	0.000	0.000	-0.729	-12.648	2.915	-4.315	2.371
Tr-2 Hor4 Sp	Gegham 1	0.000	0.000	0.000	1.000	0.000	0.000	0.000	0.000	0.000	0.000	0.000	0.000	0.000	0.000	-0.516	-9.939	3.057	-4.475	2.415
Tr-2 Hor4 Sp	Gegham 1	0.000	0.000	0.000	1.000	0.000	0.000	0.000	0.000	0.000	0.000	0.000	0.000	0.000	0.000	0.821	-10.254	3.860	-4.838	5.747
Tr-2 Hor4 Sp	Gegham 1	0.000	0.000	0.000	1.000	0.000	0.000	0.000	0.000	0.000	0.000	0.000	0.000	0.000	0.000	-0.355	-10.108	3.452	-4.391	5.563
Tr-2 Hor4 Sp	Gegham 1	0.000	0.000	0.000	1.000	0.000	0.000	0.000	0.000	0.000	0.000	0.000	0.000	0.000	0.000	-0.730	-9.993	3.948	-4.622	5.780
Tr-2 Hor3 Sp	Gegham 1	0.000	0.000	0.000	1.000	0.000	0.000	0.000	0.000	0.000	0.000	0.000	0.000	0.000	0.000	-1.531	-13.197	2.258	-4.406	3.118
Tr-2 Hor3 Sp	Gegham 1	0.000	0.000	0.000	1.000	0.000	0.000	0.000	0.000	0.000	0.000	0.000	0.000	0.000	0.000	-1.302	-11.130	2.558	-4.549	3.420
Tr-2 Hor3 Sp	Gegham 1	0.000	0.000	0.000	1.000	0.000	0.000	0.000	0.000	0.000	0.000	0.000	0.000	0.000	0.000	-2.331	-12.333	2.999	-4.374	2.261
Tr-2 Hor3 Sp	Kelbadjar	0.000	0.000	0.000	0.000	0.000	0.000	0.000	0.000	1.000	0.000	0.000	0.000	0.000	0.000	-9.444	-7.628	2.357	4.441	-1.874
Tr-2 Hor3 Sp	Gegham 1	0.000	0.000	0.000	1.000	0.000	0.000	0.000	0.000	0.000	0.000	0.000	0.000	0.000	0.000	-0.693	-11.387	2.866	-4.512	2.623
Tr-2 Hor3 Sp	Gegham 1	0.000	0.000	0.000	1.000	0.000	0.000	0.000	0.000	0.000	0.000	0.000	0.000	0.000	0.000	-0.683	-10.055	3.361	-4.542	3.454
Tr-2 Hor3 Sp	Kelbadjar	0.000	0.000	0.000	0.000	0.000	0.000	0.000	0.000	1.000	0.000	0.000	0.000	0.000	0.000	-9.467	-10.661	0.652	5.096	-1.526
Tr-2 Hor3 Sp	Kelbadjar	0.000	0.000	0.000	0.000	0.000	0.000	0.000	0.000	0.992	0.000	0.000	0.000	0.008	0.000	-10.062	-11.766	-0.652	5.191	-1.712
Tr-2 Hor3 Sp	Gegham 1	0.000	0.000	0.000	1.000	0.000	0.000	0.000	0.000	0.000	0.000	0.000	0.000	0.000	0.000	0.014	-11.365	2.348	-4.675	6.086
Tr-2 Hor3 Sp	Gegham 1	0.000	0.000	0.000	1.000	0.000	0.000	0.000	0.000	0.000	0.000	0.000	0.000	0.000	0.000	-1.853	-11.069	3.547	-4.487	3.494
Tr-2 Hor3 Sp	Gegham 1	0.000	0.000	0.000	1.000	0.000	0.000	0.000	0.000	0.000	0.000	0.000	0.000	0.000	0.000	-0.612	-10.819	3.223	-4.501	4.135
Tr-2 Hor3 Sp	Syunik	0.000	0.000	0.000	0.000	0.000	0.000	0.000	0.000	0.000	0.000	0.000	0.000	1.000	0.000	-6.762	-13.866	-1.325	4.404	-1.088
Tr-2 Hor3 Sp	Gegham 1	0.000	0.000	0.000	1.000	0.000	0.000	0.000	0.000	0.000	0.000	0.000	0.000	0.000	0.000	-0.162	-12.303	3.222	-4.863	4.282
Tr-2 Hor3 Sp	Kelbadjar	0.000	0.000	0.000	0.000	0.000	0.000	0.000	0.000	1.000	0.000	0.000	0.000	0.000	0.000	-7.884	-9.462	-0.729	5.463	-1.736

Tr-2 Hor3 Sp	Gegham 1	0.000	0.000	0.000	1.000	0.000	0.000	0.000	0.000	0.000	0.000	0.000	0.000	0.000	0.000	-1.056	-10.218	3.218	-4.528	4.042
Tr-2 Hor3 Sp	Gegham 1	0.000	0.000	0.000	1.000	0.000	0.000	0.000	0.000	0.000	0.000	0.000	0.000	0.000	0.000	-0.771	-11.618	2.827	-4.066	2.880
Tr-2 Hor3 Sp	Syunik	0.000	0.000	0.000	0.000	0.000	0.000	0.000	0.000	0.000	0.000	0.000	0.000	1.000	0.000	-6.122	-12.280	-1.430	3.854	-1.309
Tr-2 Hor3 Sp	Gegham 1	0.000	0.000	0.000	1.000	0.000	0.000	0.000	0.000	0.000	0.000	0.000	0.000	0.000	0.000	-0.935	-11.337	2.393	-4.711	5.194
Tr-2 Hor3 Sp	Gegham 1	0.000	0.000	0.000	1.000	0.000	0.000	0.000	0.000	0.000	0.000	0.000	0.000	0.000	0.000	-1.550	-11.993	2.764	-4.608	4.298
Tr-2 Hor3 Sp	Gegham 1	0.000	0.000	0.000	1.000	0.000	0.000	0.000	0.000	0.000	0.000	0.000	0.000	0.000	0.000	-0.327	-10.685	3.295	-4.709	3.946
Tr-2 Hor3 Sp	Gegham 1	0.000	0.000	0.000	1.000	0.000	0.000	0.000	0.000	0.000	0.000	0.000	0.000	0.000	0.000	-0.544	-11.904	3.017	-4.796	3.761
Tr-2 Hor3 Sp	Kelbadjar	0.000	0.000	0.000	0.000	0.000	0.000	0.000	0.000	1.000	0.000	0.000	0.000	0.000	0.000	-8.054	-8.267	0.767	4.219	-2.176
Tr-2 Hor3 Sp	Gegham 1	0.000	0.000	0.000	1.000	0.000	0.000	0.000	0.000	0.000	0.000	0.000	0.000	0.000	0.000	-0.544	-11.175	2.810	-4.052	3.226
Tr-2 Hor3 Sp	Arteni	0.000	1.000	0.000	0.000	0.000	0.000	0.000	0.000	0.000	0.000	0.000	0.000	0.000	0.000	-4.390	0.961	5.217	-3.418	-7.263
Tr-2 Hor3 Sp	Gegham 1	0.000	0.000	0.000	1.000	0.000	0.000	0.000	0.000	0.000	0.000	0.000	0.000	0.000	0.000	-0.082	-11.917	1.778	-4.602	4.266
Tr-2 Hor3 Sp	Gegham 1	0.000	0.000	0.000	1.000	0.000	0.000	0.000	0.000	0.000	0.000	0.000	0.000	0.000	0.000	-0.778	-10.994	3.476	-4.153	2.888
Tr-2 Hor3 Sp	Gegham 1	0.000	0.000	0.000	1.000	0.000	0.000	0.000	0.000	0.000	0.000	0.000	0.000	0.000	0.000	2.911	-10.770	2.670	-3.993	4.411
Tr-2 Hor3 Sp	Gegham 1	0.000	0.000	0.000	1.000	0.000	0.000	0.000	0.000	0.000	0.000	0.000	0.000	0.000	0.000	-0.583	-10.042	3.910	-4.117	2.037
Tr-2 Hor3 Sp	Kelbadjar	0.000	0.000	0.000	0.000	0.000	0.000	0.000	0.000	1.000	0.000	0.000	0.000	0.000	0.000	-8.440	-9.579	-0.524	3.781	-1.631
Tr-2 Hor3 Sp	Gegham 1	0.000	0.000	0.000	1.000	0.000	0.000	0.000	0.000	0.000	0.000	0.000	0.000	0.000	0.000	0.339	-10.126	3.213	-4.333	4.171
Tr-2 Hor3 Sp	Gutansar	0.000	0.000	0.000	0.000	0.000	1.000	0.000	0.000	0.000	0.000	0.000	0.000	0.000	0.000	4.387	1.031	12.878	14.157	-7.870
Tr-2 Hor3 Sp	Gegham 1	0.000	0.000	0.000	1.000	0.000	0.000	0.000	0.000	0.000	0.000	0.000	0.000	0.000	0.000	-0.174	-10.214	3.447	-4.477	2.995
Tr-2 Hor3 Sp	Gegham 1	0.000	0.000	0.000	1.000	0.000	0.000	0.000	0.000	0.000	0.000	0.000	0.000	0.000	0.000	-1.098	-11.419	2.699	-4.783	4.930
Tr-2 Hor3 Sp	Gegham 1	0.000	0.000	0.000	1.000	0.000	0.000	0.000	0.000	0.000	0.000	0.000	0.000	0.000	0.000	0.310	-10.649	3.829	-4.887	4.538
Tr-2 Hor3 Sp	Gegham 1	0.000	0.000	0.000	1.000	0.000	0.000	0.000	0.000	0.000	0.000	0.000	0.000	0.000	0.000	0.028	-10.066	4.555	-5.050	4.520
Tr-2 Hor3 Sp	Kelbadjar	0.000	0.000	0.000	0.000	0.000	0.000	0.000	0.000	1.000	0.000	0.000	0.000	0.000	0.000	-10.323	-9.719	-1.168	5.139	-1.371
Tr-2 Hor3 Sp	Gegham 1	0.000	0.000	0.000	1.000	0.000	0.000	0.000	0.000	0.000	0.000	0.000	0.000	0.000	0.000	-1.141	-10.207	3.133	-4.449	3.075
Tr-2 Hor3 Sp	Gegham 1	0.000	0.000	0.000	1.000	0.000	0.000	0.000	0.000	0.000	0.000	0.000	0.000	0.000	0.000	-1.654	-11.251	2.435	-4.579	8.131
Tr-2 Hor3 Sp	Kelbadjar	0.000	0.000	0.000	0.000	0.000	0.000	0.000	0.000	1.000	0.000	0.000	0.000	0.000	0.000	-9.231	-9.889	-1.352	5.110	-1.350
Tr-2 Hor3 Sp	Gegham 1	0.000	0.000	0.000	1.000	0.000	0.000	0.000	0.000	0.000	0.000	0.000	0.000	0.000	0.000	0.096	-11.843	3.248	-4.589	6.086
Tr-2 Hor3 Sp	Gegham 1	0.000	0.000	0.000	1.000	0.000	0.000	0.000	0.000	0.000	0.000	0.000	0.000	0.000	0.000	0.275	-10.375	3.517	-4.934	5.017
Tr-2 Hor3 Sp	Syunik	0.000	0.000	0.000	0.000	0.000	0.000	0.000	0.000	0.134	0.000	0.000	0.000	0.866	0.000	-10.823	-10.116	-1.435	5.481	-1.962
Tr-2 Hor3 Sp	Syunik	0.000	0.000	0.000	0.000	0.000	0.000	0.000	0.000	0.000	0.000	0.000	0.000	1.000	0.000	-9.306	-14.414	-4.898	6.043	0.205
Tr-2 Hor3 Sp	Gegham 1	0.000	0.000	0.000	1.000	0.000	0.000	0.000	0.000	0.000	0.000	0.000	0.000	0.000	0.000	-1.222	-10.332	3.413	-4.693	5.905
Tr-2 Hor3 Sp	Gegham 1	0.000	0.000	0.000	1.000	0.000	0.000	0.000	0.000	0.000	0.000	0.000	0.000	0.000	0.000	0.354	-10.149	3.956	-4.629	4.690
Tr-2 Hor3 Sp	Gegham 1	0.000	0.000	0.000	1.000	0.000	0.000	0.000	0.000	0.000	0.000	0.000	0.000	0.000	0.000	1.071	-9.228	4.131	-4.818	5.593
Tr-2 Hor3 Sp	Gegham 1	0.000	0.000	0.000	1.000	0.000	0.000	0.000	0.000	0.000	0.000	0.000	0.000	0.000	0.000	-0.835	-11.479	2.370	-4.449	3.417
Tr-2 Hor3 Sp	Gegham 1	0.000	0.000	0.000	1.000	0.000	0.000	0.000	0.000	0.000	0.000	0.000	0.000	0.000	0.000	-0.062	-11.670	3.571	-4.502	3.783
Tr-2 Hor3 Sp	Gegham 1	0.000	0.000	0.000	1.000	0.000	0.000	0.000	0.000	0.000	0.000	0.000	0.000	0.000	0.000	0.516	-10.501	3.717	-4.527	3.354
TR2 U A2-A3	Gegham 1	0.000	0.000	0.000	1.000	0.000	0.000	0.000	0.000	0.000	0.000	0.000	0.000	0.000	0.000	-0.433	-12.176	2.937	-4.381	2.834
TR2 U A2-A3	Kelbadjar	0.000	0.000	0.000	0.000	0.000	0.000	0.000	0.000	1.000	0.000	0.000	0.000	0.000	0.000	-9.726	-8.992	1.129	4.453	-1.342
TR2 U A2-A3	Gegham 1	0.000	0.000	0.000	1.000	0.000	0.000	0.000	0.000	0.000	0.000	0.000	0.000	0.000	0.000	-1.128	-10.991	3.238	-4.128	2.675
TR2 U A2-A3	Gegham 1	0.000	0.000	0.000	1.000	0.000	0.000	0.000	0.000	0.000	0.000	0.000	0.000	0.000	0.000	-0.593	-11.963	3.024	-4.889	4.527
TR2 U A2-A3	Gegham 1	0.000	0.000	0.000	1.000	0.000	0.000	0.000	0.000	0.000	0.000	0.000	0.000	0.000	0.000	-0.324	-9.911	3.509	-4.622	3.071
TR2 U A2-A3	Gegham 1	0.000	0.000	0.000	1.000	0.000	0.000	0.000	0.000	0.000	0.000	0.000	0.000	0.000	0.000	-0.077	-11.103	3.968	-4.792	3.940
TR2 U A2-A3	Gegham 1	0.000	0.000	0.000	1.000	0.000	0.000	0.000	0.000	0.000	0.000	0.000	0.000	0.000	0.000	6.533	-8.784	2.292	-4.734	7.172
TR2 U A2-A3	Gegham 1	0.000	0.000	0.000	1.000	0.000	0.000	0.000	0.000	0.000	0.000	0.000	0.000	0.000	0.000	-0.719	-10.303	3.677	-4.442	3.349
TR2 U A2-A3	Gegham 1	0.000	0.000	0.000	1.000	0.000	0.000	0.000	0.000	0.000	0.000	0.000	0.000	0.000	0.000	-0.734	-10.957	2.578	-4.439	2.705
TR2 U A2-A3	Gegham 1	0.000	0.000	0.000	1.000	0.000	0.000	0.000	0.000	0.000	0.000	0.000	0.000	0.000	0.000	-0.415	-11.845	2.387	-4.445	4.047
TR2 U A2-A3	Kelbadjar	0.000	0.000	0.000	0.000	0.000	0.000	0.000	0.000	1.000	0.000	0.000	0.000	0.000	0.000	-8.792	-9.333	-0.776	3.364	-1.183
TR2 U A2-A3	Gegham 1	0.000	0.000	0.000	1.000	0.000	0.000	0.000	0.000	0.000	0.000	0.000	0.000	0.000	0.000	-0.536	-10.897	3.161	-4.253	1.716
TR2 U A2-A3	Kelbadjar	0.000	0.000	0.000	0.000	0.000	0.000	0.000	0.000	1.000	0.000	0.000	0.000	0.000	0.000	-9.700	-9.394	-3.019	7.813	-2.497
TR2 U A2-A3	Kelbadjar	0.000	0.000	0.000	0.000	0.000	0.000	0.000	0.000	1.000	0.000	0.000	0.000	0.000	0.000	-7.669	-9.618	1.324	4.760	-1.482
TR2 U A2-A3	Gegham 1	0.000	0.000	0.000	1.000	0.000	0.000	0.000	0.000	0.000	0.000	0.000	0.000	0.000	0.000	0.515	-9.095	3.774	-4.715	4.954
TR2 U A2-A3	Gegham 1	0.000	0.000	0.000	1.000	0.000	0.000	0.000	0.000	0.000	0.000	0.000	0.000	0.000	0.000	-0.471	-10.138	4.141	-4.966	8.154
TR2 U A2-A3	Gegham 1	0.000	0.000	0.000	1.000	0.000	0.000	0.000	0.000	0.000	0.000	0.000	0.000	0.000	0.000	0.054	-9.551	3.675	-4.224	4.157
TR2 U A2-A3	Gegham 1	0.000	0.000	0.000	1.000	0.000	0.000	0.000	0.000	0.000	0.000	0.000	0.000	0.000	0.000	-0.353	-10.674	3.073	-4.936	4.984
TR2 U A2-A3	Syunik	0.000	0.000	0.000	0.000	0.000	0.000	0.000	0.000	0.000	0.000	0.000	0.000	1.000	0.000	-7.456	-12.279	-1.507	4.551	-1.297
TR2 U A2-A3	Gegham 1	0.000	0.000	0.000	1.000	0.000	0.000	0.000	0.000	0.000	0.000	0.000	0.000	0.000	0.000	1.089	-8.429	3.884	-5.267	4.559
TR2 U A2-A3	Gegham 1	0.000	0.000	0.000	1.000	0.000	0.000	0.000	0.000	0.000	0.000	0.000	0.000	0.000	0.000	0.938	-8.841	3.710	-4.499	4.494
TR2 U A2-A3	Gegham 1	0.000	0.000	0.000	1.000	0.000	0.000	0.000	0.000	0.000	0.000	0.000	0.000	0.000	0.000	-0.940	-11.631	2.498	-4.293	4.724
TR.2 UN B1	Kelbadjar	0.000	0.000	0.000	0.000	0.000	0.000	0.000	0.000	1.000	0.000	0.000	0.000	0.000	0.000	-10.289	-10.376	-0.943	5.810	-2.065
TR.2 UN B1	Gegham 1	0.000	0.000	0.000	1.000	0.000	0.000	0.000	0.000	0.000	0.000	0.000	0.000	0.000	0.000	-0.942	-11.138	2.932	-4.216	3.020
TR.2 UN B1	Kelbadjar	0.000	0.000	0.000	0.000	0.000	0.000	0.000	0.000	1.000	0.000	0.000	0.000	0.000	0.000	-6.956	-9.097	1.365	4.102	-1.494
TR.2 UN B1	Kelbadjar	0.000	0.000	0.000	0.000	0.000	0.000	0.000	0.000	1.000	0.000	0.000	0.000	0.000	0.000	-8.656	-10.339	1.050	3.416	-1.885
TR.2 UN B1	Gegham 1	0.000	0.000	0.000	1.000	0.000	0.000	0.000	0.000	0.000	0.000	0.000	0.000	0.000	0.000	-2.087	-11.022	3.210	-4.152	3.422
TR.2 UN B1	Arteni	0.000	0.998	0.000	0.000	0.002	0.000	0.000	0.000	0.000	0.000	0.000	0.000	0.000	0.000	2.995	3.718	2.806	-2.132	-4.908
TR.2 UN B1	Kelbadjar	0.000	0.000	0.000	0.000	0.000	0.000	0.000	0.000	1.000	0.000	0.000	0.000	0.000	0.000	-8.968	-9.318	0.092	4.092	-1.350
TR.2 UN B1	Gegham 1	0.000	0.000	0.000	1.000	0.000	0.000	0.000	0.000	0.000	0.000	0.000	0.000	0.000	0.000	-1.094	-10.607	3.969	-4.705	2.281
TR.2 UN B1	Kelbadjar	0.000	0.000	0.000	0.000	0.000	0.000	0.000	0.000	1.000	0.000	0.000	0.000	0.000	0.000	-8.744	-9.029	1.449	4.076	-1.783
TR.2 UN B1	Gegham 1	0.000	0.000	0.000	1.000	0.000	0.000	0.000	0.000	0.000	0.000	0.000	0.000	0.000	0.000	-0.855	-12.001	2.672	-4.773	6.950

TR.2 UN B1	Gegham 1	0.000	0.000	0.000	1.000	0.000	0.000	0.000	0.000	0.000	0.000	0.000	0.000	0.000	0.000	-0.711	-8.021	4.590	-5.244	4.473
TR.2 UN B1	Gegham 1	0.000	0.000	0.000	1.000	0.000	0.000	0.000	0.000	0.000	0.000	0.000	0.000	0.000	0.000	0.230	-11.161	3.277	-4.858	4.807
TR.2 UN B1	Gegham 1	0.000	0.000	0.000	1.000	0.000	0.000	0.000	0.000	0.000	0.000	0.000	0.000	0.000	0.000	-0.419	-9.641	3.770	-4.637	3.809
TR.2 UN B1	Gegham 1	0.000	0.000	0.000	1.000	0.000	0.000	0.000	0.000	0.000	0.000	0.000	0.000	0.000	0.000	-0.804	-9.068	4.204	-4.760	8.122
TR.2 UN B1	Gegham 1	0.000	0.000	0.000	1.000	0.000	0.000	0.000	0.000	0.000	0.000	0.000	0.000	0.000	0.000	-0.179	-10.677	4.619	-5.134	6.296
TR.2 UN B1	Gegham 1	0.000	0.000	0.000	1.000	0.000	0.000	0.000	0.000	0.000	0.000	0.000	0.000	0.000	0.000	1.145	-10.253	3.464	-4.481	5.252
TR.2 UN B1	Gegham 1	0.000	0.000	0.000	1.000	0.000	0.000	0.000	0.000	0.000	0.000	0.000	0.000	0.000	0.000	-0.137	-12.166	3.164	-4.716	5.551
TR.2 UN B1	Gegham 1	0.000	0.000	0.000	1.000	0.000	0.000	0.000	0.000	0.000	0.000	0.000	0.000	0.000	0.000	-0.001	-6.665	3.155	1.303	-3.582
TR.2 UN B1	Syunik	0.000	0.000	0.000	0.000	0.000	0.000	0.000	0.000	0.000	0.000	0.000	0.000	1.000	0.000	-5.782	-12.516	-4.636	6.741	-1.475
TR2 UN-A1 H	Gegham 1	0.000	0.000	0.000	1.000	0.000	0.000	0.000	0.000	0.000	0.000	0.000	0.000	0.000	0.000	-1.626	-11.788	3.000	-4.555	4.126
TR2 UN-A1 H	Syunik	0.000	0.000	0.000	0.000	0.000	0.000	0.000	0.000	0.002	0.000	0.000	0.000	0.998	0.000	-5.771	-12.006	-1.945	3.152	-0.674
TR2 UN-A1 H	Kelbadjar	0.000	0.000	0.000	0.000	0.000	0.000	0.000	0.000	1.000	0.000	0.000	0.000	0.000	0.000	-8.037	-9.091	0.248	3.622	-1.062
TR2 UN-A1 H	Gegham 1	0.000	0.000	0.000	1.000	0.000	0.000	0.000	0.000	0.000	0.000	0.000	0.000	0.000	0.000	-0.042	-12.011	2.918	-4.274	3.834
TR2 UN-A1 H	Syunik	0.000	0.000	0.000	0.000	0.000	0.000	0.000	0.000	0.000	0.000	0.000	0.000	1.000	0.000	-6.718	-13.095	-2.859	3.910	-0.878
TR2 UN-A1 H	Gegham 1	0.000	0.000	0.000	1.000	0.000	0.000	0.000	0.000	0.000	0.000	0.000	0.000	0.000	0.000	-0.392	-11.692	3.500	-4.541	2.633
TR2 UN-A1 H	Kelbadjar	0.000	0.000	0.000	0.000	0.000	0.000	0.000	0.000	1.000	0.000	0.000	0.000	0.000	0.000	-8.566	-8.211	1.306	2.897	-2.029
TR2 UN-A1 H	Gegham 1	0.000	0.000	0.000	1.000	0.000	0.000	0.000	0.000	0.000	0.000	0.000	0.000	0.000	0.000	-1.371	-10.776	3.235	-4.164	2.173
TR2 UN-A1 H	Kelbadjar	0.000	0.000	0.000	0.000	0.000	0.000	0.000	0.000	1.000	0.000	0.000	0.000	0.000	0.000	-9.564	-9.995	0.424	4.954	-1.846
TR2 UN-A1 H	Syunik	0.000	0.000	0.000	0.000	0.000	0.000	0.000	0.000	0.000	0.000	0.000	0.000	1.000	0.000	-5.733	-12.301	-1.329	4.307	-1.183
TR2 UN-A1 H	Syunik	0.000	0.000	0.000	0.000	0.000	0.000	0.000	0.000	0.000	0.000	0.000	0.000	1.000	0.000	-11.360	-10.492	-2.689	7.352	-2.567
TR2 UN-A1 H	Kelbadjar	0.000	0.000	0.000	0.000	0.000	0.000	0.000	0.000	1.000	0.000	0.000	0.000	0.000	0.000	-8.476	-9.789	0.282	4.937	-1.509
TR2 UN-A1 H	Gegham 1	0.000	0.000	0.000	1.000	0.000	0.000	0.000	0.000	0.000	0.000	0.000	0.000	0.000	0.000	-1.841	-10.709	2.983	-3.456	2.331
TR2 UN-A1 H	Gegham 1	0.000	0.000	0.000	1.000	0.000	0.000	0.000	0.000	0.000	0.000	0.000	0.000	0.000	0.000	0.105	-10.219	2.637	-4.676	6.104
TR2 UN-A1 H	Gegham 1	0.000	0.000	0.000	1.000	0.000	0.000	0.000	0.000	0.000	0.000	0.000	0.000	0.000	0.000	-0.582	-11.802	1.935	-4.444	4.947
TR2 UN-A1 H	Kelbadjar	0.000	0.000	0.000	0.000	0.000	0.000	0.000	0.000	1.000	0.000	0.000	0.000	0.000	0.000	-9.686	-10.359	0.068	4.391	-1.412
TR2 UN-A1 H	Kelbadjar	0.000	0.000	0.000	0.000	0.000	0.000	0.000	0.000	1.000	0.000	0.000	0.000	0.000	0.000	-8.697	-8.159	0.202	4.128	-1.715
TR2 UN-A1 H	Syunik	0.000	0.000	0.000	0.000	0.000	0.000	0.000	0.000	0.000	0.000	0.000	0.000	1.000	0.000	-7.090	-13.609	-2.436	5.392	-2.217
TR2 UN-A1 H	Gegham 1	0.000	0.000	0.000	1.000	0.000	0.000	0.000	0.000	0.000	0.000	0.000	0.000	0.000	0.000	-1.112	-11.804	3.372	-4.885	5.453
TR2 UN-A1 H	Gegham 1	0.000	0.000	0.000	1.000	0.000	0.000	0.000	0.000	0.000	0.000	0.000	0.000	0.000	0.000	0.739	-10.134	4.001	-4.766	3.622
TR2 UN-A1 H	Kelbadjar	0.000	0.000	0.000	0.000	0.000	0.000	0.000	0.000	1.000	0.000	0.000	0.000	0.000	0.000	-9.287	-9.856	-1.648	7.610	-2.327
TR2 UN-A1 H	Gegham 1	0.000	0.000	0.000	1.000	0.000	0.000	0.000	0.000	0.000	0.000	0.000	0.000	0.000	0.000	-0.056	-11.767	2.683	-4.452	5.347
TR2 UN-A1 H	Gegham 1	0.000	0.000	0.000	1.000	0.000	0.000	0.000	0.000	0.000	0.000	0.000	0.000	0.000	0.000	-0.716	-8.832	4.280	-4.770	5.269
TR2 UN-A1 H	Kelbadjar	0.000	0.000	0.000	0.000	0.000	0.000	0.000	0.000	1.000	0.000	0.000	0.000	0.000	0.000	-8.303	-8.584	0.581	3.379	-1.744
TR2 UN-A1 H	Kelbadjar	0.000	0.000	0.000	0.000	0.000	0.000	0.000	0.000	1.000	0.000	0.000	0.000	0.000	0.000	-9.631	-9.631	-0.423	4.797	-1.608
TR2 UN-A1 H	Gegham 1	0.000	0.000	0.000	1.000	0.000	0.000	0.000	0.000	0.000	0.000	0.000	0.000	0.000	0.000	2.914	-8.598	3.876	-4.901	5.813
TR2 UN-A1 H	Kelbadjar	0.000	0.000	0.000	0.000	0.000	0.000	0.000	0.000	1.000	0.000	0.000	0.000	0.000	0.000	-9.772	-8.332	-3.447	7.863	-2.641
Tr-2 H1S2 - H	Kelbadjar	0.000	0.000	0.000	0.000	0.000	0.000	0.000	0.000	1.000	0.000	0.000	0.000	0.000	0.000	-6.306	-9.450	-0.834	5.429	-0.189
Tr-2 H1S2 - H	Gegham 1	0.000	0.000	0.000	1.000	0.000	0.000	0.000	0.000	0.000	0.000	0.000	0.000	0.000	0.000	-0.748	-11.683	3.479	-4.747	4.529
Tr-2 H1S2 - H	Kelbadjar	0.000	0.000	0.000	0.000	0.000	0.000	0.000	0.000	0.998	0.000	0.000	0.000	0.002	0.000	-6.079	-9.866	-0.791	3.530	-1.889
Tr-2 H1S2 - H	Gegham 1	0.000	0.000	0.000	1.000	0.000	0.000	0.000	0.000	0.000	0.000	0.000	0.000	0.000	0.000	-0.088	-9.458	4.460	-4.300	2.431
Tr-2 H1S2 - H	Kelbadjar	0.000	0.000	0.000	0.000	0.000	0.000	0.000	0.000	1.000	0.000	0.000	0.000	0.000	0.000	-9.682	-10.167	0.682	4.465	-2.068
Tr-2 H1S2 - H	Gegham 1	0.000	0.000	0.000	1.000	0.000	0.000	0.000	0.000	0.000	0.000	0.000	0.000	0.000	0.000	0.206	-9.538	4.293	-4.616	2.924
Tr-2 H1S2 - H	Gegham 1	0.000	0.000	0.000	1.000	0.000	0.000	0.000	0.000	0.000	0.000	0.000	0.000	0.000	0.000	-1.857	-11.039	2.693	-4.344	3.645
Tr-2 H1S2 - H	Gutansar	0.000	0.000	0.000	0.000	0.000	1.000	0.000	0.000	0.000	0.000	0.000	0.000	0.000	0.000	5.118	-0.747	13.258	18.859	-8.928
Tr-2 H1S2 - H	Kelbadjar	0.000	0.000	0.000	0.000	0.000	0.000	0.000	0.000	1.000	0.000	0.000	0.000	0.000	0.000	-9.859	-9.428	0.975	4.195	-1.656
Tr-2 H1S2 - H	Kelbadjar	0.000	0.000	0.000	0.000	0.000	0.000	0.000	0.000	1.000	0.000	0.000	0.000	0.000	0.000	-9.276	-9.855	1.462	4.117	-1.715
Tr-2 H1S2 - H	Kelbadjar	0.000	0.000	0.000	0.000	0.000	0.000	0.000	0.000	1.000	0.000	0.000	0.000	0.000	0.000	-7.929	-9.400	0.987	5.255	-1.716
Tr-2 H1S2 - H	Gegham 1	0.000	0.000	0.000	1.000	0.000	0.000	0.000	0.000	0.000	0.000	0.000	0.000	0.000	0.000	0.092	-11.135	3.451	-4.976	5.010
Tr-2 H1S2 - H	Gegham 1	0.000	0.000	0.000	1.000	0.000	0.000	0.000	0.000	0.000	0.000	0.000	0.000	0.000	0.000	0.336	-10.130	3.825	-4.528	2.126
Tr-2 H1S2 - H	Syunik	0.000	0.000	0.000	0.000	0.000	0.000	0.000	0.000	0.000	0.000	0.000	0.000	1.000	0.000	-7.382	-17.896	-3.981	2.613	0.004
Tr-2 H1S2 - H	Kelbadjar	0.000	0.000	0.000	0.000	0.000	0.000	0.000	0.000	1.000	0.000	0.000	0.000	0.000	0.000	-9.130	-9.082	0.151	4.658	-2.235
Tr-2 H1S2 - H	Kelbadjar	0.000	0.000	0.000	0.000	0.000	0.000	0.000	0.000	1.000	0.000	0.000	0.000	0.000	0.000	-9.573	-7.905	-0.560	3.900	-1.526
Tr-2 H1S2 - H	Gegham 1	0.000	0.000	0.000	1.000	0.000	0.000	0.000	0.000	0.000	0.000	0.000	0.000	0.000	0.000	-0.318	-8.863	3.679	-4.988	6.487
Tr-2 H1S2 - H	Kelbadjar	0.000	0.000	0.000	0.000	0.000	0.000	0.000	0.000	1.000	0.000	0.000	0.000	0.000	0.000	-9.796	-10.303	-0.661	4.832	-1.522
Tr-2 H1S2 - H	Gegham 1	0.000	0.000	0.000	1.000	0.000	0.000	0.000	0.000	0.000	0.000	0.000	0.000	0.000	0.000	-0.168	-11.429	3.567	-5.294	7.406
Tr-2 H1S2 - H	Syunik	0.000	0.000	0.000	0.000	0.000	0.000	0.000	0.000	0.000	0.000	0.000	0.000	1.000	0.000	-6.946	-13.763	-2.077	5.079	-1.329
Tr-2 H1S2 - H	Gegham 1	0.000	0.000	0.000	1.000	0.000	0.000	0.000	0.000	0.000	0.000	0.000	0.000	0.000	0.000	0.604	-10.335	3.501	-4.566	5.323
Tr-2 H1S2 - H	Arteni	0.000	1.000	0.000	0.000	0.000	0.000	0.000	0.000	0.000	0.000	0.000	0.000	0.000	0.000	-0.641	2.012	4.191	-3.557	-7.489
Tr-2 H1S2 - H	Syunik	0.000	0.000	0.000	0.000	0.000	0.000	0.000	0.000	0.000	0.000	0.000	0.000	1.000	0.000	-6.175	-12.578	-3.362	5.432	-1.352
Tr-2 H1S2 - H	Kelbadjar	0.000	0.000	0.000	0.000	0.000	0.000	0.000	0.000	1.000	0.000	0.000	0.000	0.000	0.000	-11.143	-10.275	-0.961	5.934	-1.670
Tr-2 H1S2 - H	Arteni	0.000	0.558	0.000	0.000	0.442	0.000	0.000	0.000	0.000	0.000	0.000	0.000	0.000	0.000	-5.139	6.597	5.867	-3.839	-2.255
Tr-2 H1S2 - H	Syunik	0.000	0.000	0.000	0.000	0.000	0.000	0.000	0.000	0.000	0.000	0.000	0.000	1.000	0.000	-7.980	-13.205	-3.075	6.680	-1.446
Tr-2 Hor 2 Sp	Kelbadjar	0.000	0.000	0.000	0.000	0.000	0.000	0.000	0.000	1.000	0.000	0.000	0.000	0.000	0.000	-8.549	-9.704	1.653	2.902	-0.943
Tr-2 Hor 2 Sp	Arteni	0.000	1.000	0.000	0.000	0.000	0.000	0.000	0.000	0.000	0.000	0.000	0.000	0.000	0.000	-4.092	1.271	5.242	-3.257	-6.906
Tr-2 Hor 2 Sp	Gegham 1	0.000	0.000	0.000	1.000	0.000	0.000	0.000	0.000	0.000	0.000	0.000	0.000	0.000	0.000	-0.801	-10.016	4.243	-4.376	2.070
Tr-2 Hor 2 Sp	Gegham 1	0.000	0.000	0.000	1.000	0.000	0.000	0.000	0.000	0.000	0.000	0.000	0.000	0.000	0.000	-0.500	-9.838	3.859	-4.575	3.109
Tr-2 Hor 2 Sp	Gegham 1	0.000	0.000	0.000	1.000	0.000	0.000	0.000	0.000	0.000	0.000	0.000	0.000	0.000	0.000	0.498	-10.211	1.947	-4.293	4.050

Tr-2 Hor 2 Sp	Gegham 1	0.000	0.000	0.000	1.000	0.000	0.000	0.000	0.000	0.000	0.000	0.000	0.000	0.000	0.000	0.072	-9.437	4.499	-4.470	3.198
Tr-2 Hor 2 Sp	Kelbadjar	0.000	0.000	0.000	0.000	0.000	0.000	0.000	0.000	1.000	0.000	0.000	0.000	0.000	0.000	-9.783	-11.390	-0.634	4.106	-1.213
Tr-2 Hor 2 Sp	Kelbadjar	0.000	0.000	0.000	0.000	0.000	0.000	0.000	0.000	1.000	0.000	0.000	0.000	0.000	0.000	-10.019	-8.026	-1.261	6.282	-2.962
Tr-2 Hor 2 Sp	Gegham 1	0.000	0.000	0.000	1.000	0.000	0.000	0.000	0.000	0.000	0.000	0.000	0.000	0.000	0.000	-2.873	-11.989	2.713	-4.031	3.581
Tr-2 Hor 2 Sp	Gegham 1	0.000	0.000	0.000	1.000	0.000	0.000	0.000	0.000	0.000	0.000	0.000	0.000	0.000	0.000	-0.921	-10.593	3.425	-4.665	3.501
Tr-2 Hor 2 Sp	Gegham 1	0.000	0.000	0.000	1.000	0.000	0.000	0.000	0.000	0.000	0.000	0.000	0.000	0.000	0.000	-0.645	-11.376	2.850	-4.774	4.434
Tr-2 Hor 2 Sp	Gegham 1	0.000	0.000	0.000	1.000	0.000	0.000	0.000	0.000	0.000	0.000	0.000	0.000	0.000	0.000	-0.196	-11.031	3.375	-4.529	4.177
Tr-2 Hor 2 Sp	Gegham 1	0.000	0.000	0.000	1.000	0.000	0.000	0.000	0.000	0.000	0.000	0.000	0.000	0.000	0.000	-0.052	-10.224	4.061	-4.710	6.064
Tr-2 Hor 2 Sp	Kelbadjar	0.000	0.000	0.000	0.000	0.000	0.000	0.000	0.000	1.000	0.000	0.000	0.000	0.000	0.000	-11.452	-7.866	1.129	5.333	-2.133
Tr-2 Hor 2 Sp	Gegham 1	0.000	0.000	0.000	1.000	0.000	0.000	0.000	0.000	0.000	0.000	0.000	0.000	0.000	0.000	-0.028	-10.163	3.418	-4.704	3.856
Tr-2 Hor 2 Sp	Gegham 1	0.000	0.000	0.000	1.000	0.000	0.000	0.000	0.000	0.000	0.000	0.000	0.000	0.000	0.000	0.166	-9.402	4.689	-5.229	7.499
Tr-2 Hor 2 Sp	Gegham 1	0.000	0.000	0.000	1.000	0.000	0.000	0.000	0.000	0.000	0.000	0.000	0.000	0.000	0.000	-1.275	-10.957	3.093	-4.641	4.708
Tr-2 Hor 2 Sp	Gegham 1	0.000	0.000	0.000	1.000	0.000	0.000	0.000	0.000	0.000	0.000	0.000	0.000	0.000	0.000	0.225	-11.012	3.356	-4.958	7.674
Tr-2 Hor 2 Sp	Gegham 1	0.000	0.000	0.000	1.000	0.000	0.000	0.000	0.000	0.000	0.000	0.000	0.000	0.000	0.000	-0.274	-10.928	2.904	-4.639	5.766
Tr-2 Hor 2 Sp	Gegham 1	0.000	0.000	0.000	1.000	0.000	0.000	0.000	0.000	0.000	0.000	0.000	0.000	0.000	0.000	-0.664	-11.882	3.543	-5.172	8.312
Tr-2 Hor 2 Sp	Kelbadjar	0.000	0.000	0.000	0.000	0.000	0.000	0.000	0.000	1.000	0.000	0.000	0.000	0.000	0.000	-11.068	-10.215	-0.741	5.607	-1.624
Tr-2 Hor 2 Sp	Gegham 1	0.000	0.000	0.000	1.000	0.000	0.000	0.000	0.000	0.000	0.000	0.000	0.000	0.000	0.000	-1.108	-11.463	2.949	-4.277	7.667
Tr-2 Hor 2 Sp	Kelbadjar	0.000	0.000	0.000	0.000	0.000	0.000	0.000	0.000	1.000	0.000	0.000	0.000	0.000	0.000	-9.709	-9.400	-1.522	5.013	-1.767
Tr-2 Hor 2 Sp	Syunik	0.000	0.000	0.000	0.000	0.000	0.000	0.000	0.000	0.000	0.000	0.000	0.000	1.000	0.000	-8.246	-19.872	-10.191	8.324	-2.817
Tr-2 Hor 2 Sp	Gegham 1	0.000	0.000	0.000	1.000	0.000	0.000	0.000	0.000	0.000	0.000	0.000	0.000	0.000	0.000	-0.248	-10.675	3.321	-4.695	4.141
Tr-2 Hor 2 Sp	Kelbadjar	0.000	0.000	0.000	0.000	0.000	0.000	0.000	0.000	1.000	0.000	0.000	0.000	0.000	0.000	-10.075	-9.695	0.555	4.850	-1.567
Tr-2 Hor 2 Sp	Gegham 1	0.000	0.000	0.000	1.000	0.000	0.000	0.000	0.000	0.000	0.000	0.000	0.000	0.000	0.000	0.020	-9.849	4.217	-4.288	2.663
Tr-2 Hor 2 Sp	Gegham 1	0.000	0.000	0.000	1.000	0.000	0.000	0.000	0.000	0.000	0.000	0.000	0.000	0.000	0.000	1.033	-10.068	3.522	-4.548	5.077
TR.2 A1 H1S1	Arteni	0.000	1.000	0.000	0.000	0.000	0.000	0.000	0.000	0.000	0.000	0.000	0.000	0.000	0.000	-4.303	-0.031	3.543	-4.201	-6.360
Tr2 A4 H3 S2	Gegham 1	0.000	0.000	0.000	1.000	0.000	0.000	0.000	0.000	0.000	0.000	0.000	0.000	0.000	0.000	-1.114	-11.146	3.038	-4.420	3.101
Tr2 A4 H3 S2	Gegham 1	0.000	0.000	0.000	1.000	0.000	0.000	0.000	0.000	0.000	0.000	0.000	0.000	0.000	0.000	0.557	-10.600	2.229	-3.811	3.727
Tr2 A4 H3 S2	Gegham 1	0.000	0.000	0.000	1.000	0.000	0.000	0.000	0.000	0.000	0.000	0.000	0.000	0.000	0.000	-1.459	-11.667	3.657	-4.521	3.189
Tr2 A4 H3 S2	Gegham 1	0.000	0.000	0.000	1.000	0.000	0.000	0.000	0.000	0.000	0.000	0.000	0.000	0.000	0.000	-0.985	-10.984	3.709	-4.197	1.375
Tr2 A4 H3 S2	Kelbadjar	0.000	0.000	0.000	0.000	0.000	0.000	0.000	0.000	1.000	0.000	0.000	0.000	0.000	0.000	-9.428	-10.194	-1.065	4.832	-1.338
Tr2 A4 H3 S2	Gegham 1	0.000	0.000	0.000	1.000	0.000	0.000	0.000	0.000	0.000	0.000	0.000	0.000	0.000	0.000	-1.571	-10.744	3.571	-4.288	2.832
Tr2 A4 H3 S2	Gegham 1	0.000	0.000	0.000	1.000	0.000	0.000	0.000	0.000	0.000	0.000	0.000	0.000	0.000	0.000	0.531	-11.054	3.011	-4.761	4.630
Tr2 A4 H3 S2	Syunik	0.000	0.000	0.000	0.000	0.000	0.000	0.000	0.000	0.000	0.000	0.000	0.000	1.000	0.000	-7.449	-12.282	-1.633	3.507	-1.322
Tr2 A4 H3 S2	Gegham 1	0.000	0.000	0.000	1.000	0.000	0.000	0.000	0.000	0.000	0.000	0.000	0.000	0.000	0.000	-1.404	-10.633	3.259	-4.326	2.881
Tr2 A4 H3 S2	Gegham 1	0.000	0.000	0.000	1.000	0.000	0.000	0.000	0.000	0.000	0.000	0.000	0.000	0.000	0.000	-0.161	-10.322	3.145	-4.197	1.931
Tr2 A4 H3 S2	Kelbadjar	0.000	0.000	0.000	0.000	0.000	0.000	0.000	0.000	1.000	0.000	0.000	0.000	0.000	0.000	-9.945	-10.190	-1.263	3.987	-0.785
Tr2 A4 H3 S2	Gegham 1	0.000	0.000	0.000	1.000	0.000	0.000	0.000	0.000	0.000	0.000	0.000	0.000	0.000	0.000	0.451	-10.843	2.989	-5.151	4.433
Tr2 A4 H3 S2	Gegham 1	0.000	0.000	0.000	1.000	0.000	0.000	0.000	0.000	0.000	0.000	0.000	0.000	0.000	0.000	-0.216	-10.269	3.757	-3.934	1.620
Tr2 A4 H3 S2	Kelbadjar	0.000	0.000	0.000	0.000	0.000	0.000	0.000	0.000	1.000	0.000	0.000	0.000	0.000	0.000	-9.140	-10.265	1.304	4.173	-1.636
Tr2 A4 H3 S2	Gegham 1	0.000	0.000	0.000	1.000	0.000	0.000	0.000	0.000	0.000	0.000	0.000	0.000	0.000	0.000	0.503	-10.607	5.253	-5.205	5.059
TR.2 collapse	Kelbadjar	0.000	0.000	0.000	0.000	0.000	0.000	0.000	0.000	1.000	0.000	0.000	0.000	0.000	0.000	-8.079	-8.389	2.944	1.656	-1.567
TR.2 collapse	Kelbadjar	0.000	0.000	0.000	0.000	0.000	0.000	0.000	0.000	1.000	0.000	0.000	0.000	0.000	0.000	-8.460	-8.976	2.579	2.647	-1.919
TR.2 collapse	Kelbadjar	0.000	0.000	0.000	0.000	0.000	0.000	0.000	0.000	1.000	0.000	0.000	0.000	0.000	0.000	-5.080	-10.226	-0.167	2.233	-1.650
TR.2 collapse	Arteni	0.000	1.000	0.000	0.000	0.000	0.000	0.000	0.000	0.000	0.000	0.000	0.000	0.000	0.000	-3.074	0.023	3.922	-3.366	-6.281
TR.2 collapse	Gegham 1	0.000	0.000	0.000	1.000	0.000	0.000	0.000	0.000	0.000	0.000	0.000	0.000	0.000	0.000	-1.299	-11.854	2.890	-4.105	3.923
TR.2 A2-A3 H	Gegham 1	0.000	0.000	0.000	1.000	0.000	0.000	0.000	0.000	0.000	0.000	0.000	0.000	0.000	0.000	-0.933	-10.902	2.641	-4.651	3.079
TR.2 A2-A3 H	Gegham 1	0.000	0.000	0.000	1.000	0.000	0.000	0.000	0.000	0.000	0.000	0.000	0.000	0.000	0.000	-1.373	-11.222	2.728	-4.768	3.373
TR.2 A2-A3 H	Syunik	0.000	0.000	0.000	0.000	0.000	0.000	0.000	0.000	0.000	0.000	0.000	0.000	1.000	0.000	-6.430	-12.849	-2.604	2.611	0.003
TR.2 A2-A3 H	Gegham 1	0.000	0.000	0.000	1.000	0.000	0.000	0.000	0.000	0.000	0.000	0.000	0.000	0.000	0.000	-0.727	-11.764	3.094	-4.683	3.948
TR.2 A2-A3 H	Gegham 1	0.000	0.000	0.000	1.000	0.000	0.000	0.000	0.000	0.000	0.000	0.000	0.000	0.000	0.000	0.286	-10.148	3.832	-5.244	5.082
TR.2 A2-A3 H	Gegham 1	0.000	0.000	0.000	1.000	0.000	0.000	0.000	0.000	0.000	0.000	0.000	0.000	0.000	0.000	-0.794	-9.959	3.809	-4.378	1.152
TR.2 A2-A3 H	Gegham 1	0.000	0.000	0.000	1.000	0.000	0.000	0.000	0.000	0.000	0.000	0.000	0.000	0.000	0.000	0.260	-9.855	3.724	-4.816	5.404
TR.2 A2-A3 H	Gegham 1	0.000	0.000	0.000	1.000	0.000	0.000	0.000	0.000	0.000	0.000	0.000	0.000	0.000	0.000	0.269	-9.460	4.490	-4.645	3.040
TR.2 A2-A3 H	Gegham 1	0.000	0.000	0.000	1.000	0.000	0.000	0.000	0.000	0.000	0.000	0.000	0.000	0.000	0.000	-0.071	-10.695	3.946	-4.971	4.126
TR.2 A2-A3 H	Gegham 1	0.000	0.000	0.000	1.000	0.000	0.000	0.000	0.000	0.000	0.000	0.000	0.000	0.000	0.000	-0.995	-11.729	3.608	-4.933	4.827
TR.2 A2-A3 H	Gegham 1	0.000	0.000	0.000	1.000	0.000	0.000	0.000	0.000	0.000	0.000	0.000	0.000	0.000	0.000	0.237	-9.840	4.007	-4.467	4.367
TR.2 A2-A3 H	Gegham 1	0.000	0.000	0.000	1.000	0.000	0.000	0.000	0.000	0.000	0.000	0.000	0.000	0.000	0.000	-0.435	-10.080	3.117	-4.456	4.854
TR.2 A2-A3 H	Gegham 1	0.000	0.000	0.000	1.000	0.000	0.000	0.000	0.000	0.000	0.000	0.000	0.000	0.000	0.000	0.106	-10.603	2.651	-4.470	2.349
Tr2 A2-A3 H5	Gegham 1	0.000	0.000	0.000	1.000	0.000	0.000	0.000	0.000	0.000	0.000	0.000	0.000	0.000	0.000	-0.289	-10.639	4.314	-4.897	3.301
Tr2 A2-A3 H5	Gegham 1	0.000	0.000	0.000	1.000	0.000	0.000	0.000	0.000	0.000	0.000	0.000	0.000	0.000	0.000	-0.336	-10.660	3.476	-4.472	4.797
Tr2 A2-A3 H5	Gegham 1	0.000	0.000	0.000	1.000	0.000	0.000	0.000	0.000	0.000	0.000	0.000	0.000	0.000	0.000	0.894	-9.513	4.130	-4.814	5.439
Tr2 A2-A3 H5	Gegham 1	0.000	0.000	0.000	1.000	0.000	0.000	0.000	0.000	0.000	0.000	0.000	0.000	0.000	0.000	-0.886	-9.309	3.388	-4.845	8.189
Tr2 A2-A3 H5	Gegham 1	0.000	0.000	0.000	1.000	0.000	0.000	0.000	0.000	0.000	0.000	0.000	0.000	0.000	0.000	0.229	-10.073	4.160	-5.378	6.753
Tr2 A2-A3 H5	Kelbadjar	0.000	0.000	0.000	0.000	0.000	0.000	0.000	0.000	1.000	0.000	0.000	0.000	0.000	0.000	-8.194	-8.878	-0.041	5.128	-1.513
TR.2 A4 H5 S	Gegham 1	0.000	0.000	0.000	1.000	0.000	0.000	0.000	0.000	0.000	0.000	0.000	0.000	0.000	0.000	-0.652	-10.407	3.466	-4.278	4.620
TR.2 A4 H5 S	Syunik	0.000	0.000	0.000	0.000	0.000	0.000	0.000	0.000	0.000	0.000	0.000	0.000	1.000	0.000	-7.912	-14.967	-3.277	5.910	-1.577
TR.2 A4 H5 S	Gegham 1	0.000	0.000	0.000	1.000	0.000	0.000	0.000	0.000	0.000	0.000	0.000	0.000	0.000	0.000	-1.225	-11.902	3.046	-5.017	6.273
TR.2 A4 H5 S	Gegham 1	0.000	0.000	0.000	1.000	0.000	0.000	0.000	0.000	0.000	0.000	0.000	0.000	0.000	0.000	0.002	-10.584	2.739	-4.104	3.759

TR.2 A4 H5 S	Gegham 1	0.000	0.000	0.000	1.000	0.000	0.000	0.000	0.000	0.000	0.000	0.000	0.000	0.000	0.000	0.141	-8.700	5.215	-5.453	5.861
TR.2 A4 H5 S	Syunik	0.000	0.000	0.000	0.000	0.000	0.000	0.000	0.000	0.000	0.000	0.000	0.000	1.000	0.000	-7.876	-11.026	-4.409	5.617	-0.269
TR.2 A4 H5 S	Syunik	0.000	0.000	0.000	0.000	0.000	0.000	0.000	0.000	0.000	0.000	0.000	0.000	1.000	0.000	-8.486	-14.582	-4.100	4.696	0.940
Tr2 Unit A4 H	Syunik	0.000	0.000	0.000	0.000	0.000	0.000	0.000	0.000	0.000	0.000	0.000	0.000	1.000	0.000	-6.606	-12.778	-1.447	4.363	-1.583
Tr2 Unit A4 H	Gegham 1	0.000	0.000	0.000	1.000	0.000	0.000	0.000	0.000	0.000	0.000	0.000	0.000	0.000	0.000	0.137	-10.520	3.327	-4.323	3.446
Tr2 Unit A4 H	Gegham 1	0.000	0.000	0.000	1.000	0.000	0.000	0.000	0.000	0.000	0.000	0.000	0.000	0.000	0.000	-0.365	-11.118	3.163	-4.419	5.794
Tr2 Unit A4 H	Gegham 1	0.000	0.000	0.000	1.000	0.000	0.000	0.000	0.000	0.000	0.000	0.000	0.000	0.000	0.000	-0.030	-10.395	3.726	-4.974	5.751
Tr2 Unit A4 H	Arteni	0.000	1.000	0.000	0.000	0.000	0.000	0.000	0.000	0.000	0.000	0.000	0.000	0.000	0.000	-0.988	2.285	3.772	-3.289	-6.370
Tr2 Unit A4 H	Gegham 1	0.000	0.000	0.000	1.000	0.000	0.000	0.000	0.000	0.000	0.000	0.000	0.000	0.000	0.000	-0.759	-8.575	3.477	-4.649	6.093
Tr.2 Unit B1	Gegham 1	0.000	0.000	0.000	1.000	0.000	0.000	0.000	0.000	0.000	0.000	0.000	0.000	0.000	0.000	-0.859	-12.153	2.134	-4.235	3.423
Tr.2 Unit B1	Kelbadjar	0.000	0.000	0.000	0.000	0.000	0.000	0.000	0.000	1.000	0.000	0.000	0.000	0.000	0.000	-8.647	-9.204	0.386	3.655	-1.823
Tr.2 Unit B1	Gegham 1	0.000	0.000	0.000	1.000	0.000	0.000	0.000	0.000	0.000	0.000	0.000	0.000	0.000	0.000	-0.123	-10.565	3.794	-5.021	4.330
Tr.2 Unit B1	Gegham 1	0.000	0.000	0.000	1.000	0.000	0.000	0.000	0.000	0.000	0.000	0.000	0.000	0.000	0.000	-1.210	-12.936	2.015	-6.337	8.122
Tr.2 Unit B1	Kelbadjar	0.000	0.000	0.000	0.000	0.000	0.000	0.000	0.000	1.000	0.000	0.000	0.000	0.000	0.000	-7.765	-8.861	0.925	2.575	-0.930
Tr.2 Unit B1	Kelbadjar	0.000	0.000	0.000	0.000	0.000	0.000	0.000	0.000	1.000	0.000	0.000	0.000	0.000	0.000	-9.609	-10.051	0.810	3.643	-1.205
Tr.2 Unit B1	Kelbadjar	0.000	0.000	0.000	0.000	0.000	0.000	0.000	0.000	1.000	0.000	0.000	0.000	0.000	0.000	-7.633	-10.273	0.765	3.203	-0.953
Tr.2 Unit B1	Kelbadjar	0.000	0.000	0.000	0.000	0.000	0.000	0.000	0.000	1.000	0.000	0.000	0.000	0.000	0.000	-6.555	-8.570	1.206	4.613	-1.317
Tr.2 Unit B1	Kelbadjar	0.000	0.000	0.000	0.000	0.000	0.000	0.000	0.000	1.000	0.000	0.000	0.000	0.000	0.000	-10.073	-10.163	0.322	4.634	-1.478
Tr.2 Unit B1	Gegham 1	0.000	0.000	0.000	1.000	0.000	0.000	0.000	0.000	0.000	0.000	0.000	0.000	0.000	0.000	0.261	-10.494	3.574	-4.857	4.471
Tr.2 Unit B1	Gegham 1	0.000	0.000	0.000	1.000	0.000	0.000	0.000	0.000	0.000	0.000	0.000	0.000	0.000	0.000	-2.145	-13.349	1.857	-4.082	7.564
Tr.2 Unit B1	Gegham 1	0.000	0.000	0.000	1.000	0.000	0.000	0.000	0.000	0.000	0.000	0.000	0.000	0.000	0.000	0.124	-10.365	3.472	-4.636	3.202
Tr.2 Unit B1	Gegham 1	0.000	0.000	0.000	1.000	0.000	0.000	0.000	0.000	0.000	0.000	0.000	0.000	0.000	0.000	-0.964	-12.855	2.925	-4.467	3.653
Tr.2 Unit B1	Gegham 1	0.000	0.000	0.000	1.000	0.000	0.000	0.000	0.000	0.000	0.000	0.000	0.000	0.000	0.000	-0.605	-9.832	3.715	-4.773	2.953
Tr.2 Unit B1	Syunik	0.000	0.000	0.000	0.000	0.000	0.000	0.000	0.000	0.079	0.000	0.000	0.000	0.921	0.000	-10.624	-11.684	-1.088	5.851	-1.825
Tr.2 Unit B1	Gegham 1	0.000	0.000	0.000	1.000	0.000	0.000	0.000	0.000	0.000	0.000	0.000	0.000	0.000	0.000	-0.551	-11.721	3.288	-3.488	3.642
Tr.2 Unit B1	Gegham 1	0.000	0.000	0.000	1.000	0.000	0.000	0.000	0.000	0.000	0.000	0.000	0.000	0.000	0.000	-0.372	-10.453	3.737	-4.712	3.057
Tr.2 Unit B1	Gegham 1	0.000	0.000	0.000	1.000	0.000	0.000	0.000	0.000	0.000	0.000	0.000	0.000	0.000	0.000	-1.063	-8.662	3.889	-4.579	5.031
Tr.2 Unit B1	Kelbadjar	0.000	0.000	0.000	0.000	0.000	0.000	0.000	0.000	1.000	0.000	0.000	0.000	0.000	0.000	-9.772	-10.169	0.853	4.034	-1.328
Tr.2 Unit B1	Gegham 1	0.000	0.000	0.000	1.000	0.000	0.000	0.000	0.000	0.000	0.000	0.000	0.000	0.000	0.000	0.235	-10.056	3.952	-4.842	4.798
Tr.2 Unit B1	Gegham 1	0.000	0.000	0.000	1.000	0.000	0.000	0.000	0.000	0.000	0.000	0.000	0.000	0.000	0.000	-1.699	-11.507	3.548	-4.741	6.799
Tr.2 Unit B1	Gegham 1	0.000	0.000	0.000	1.000	0.000	0.000	0.000	0.000	0.000	0.000	0.000	0.000	0.000	0.000	-0.576	-10.510	3.666	-3.796	2.126
Tr.2 Unit B1	Gegham 1	0.000	0.000	0.000	1.000	0.000	0.000	0.000	0.000	0.000	0.000	0.000	0.000	0.000	0.000	-0.861	-11.074	3.551	-4.960	6.480
Tr.2 Unit B1	Gegham 1	0.000	0.000	0.000	1.000	0.000	0.000	0.000	0.000	0.000	0.000	0.000	0.000	0.000	0.000	-0.449	-10.320	3.689	-5.026	5.786
Tr.2 Unit B1	Gegham 1	0.000	0.000	0.000	1.000	0.000	0.000	0.000	0.000	0.000	0.000	0.000	0.000	0.000	0.000	-0.103	-9.971	4.084	-4.795	3.126
Tr.2 Unit B1	Gegham 1	0.000	0.000	0.000	1.000	0.000	0.000	0.000	0.000	0.000	0.000	0.000	0.000	0.000	0.000	-0.696	-10.967	3.365	-4.893	5.695
Tr.2 Unit B1	Gegham 1	0.000	0.000	0.000	1.000	0.000	0.000	0.000	0.000	0.000	0.000	0.000	0.000	0.000	0.000	-0.466	-10.568	2.868	-4.847	5.233
Tr.2 Unit B1	Kelbadjar	0.000	0.000	0.000	0.000	0.000	0.000	0.000	0.000	1.000	0.000	0.000	0.000	0.000	0.000	-10.322	-10.104	0.307	5.336	-2.122
Tr.2 Unit B1	Syunik	0.000	0.000	0.000	0.000	0.000	0.000	0.000	0.000	0.000	0.000	0.000	0.000	1.000	0.000	-6.351	-13.641	-3.224	4.098	-1.285
Tr.2 Unit B1	Gegham 1	0.000	0.000	0.000	1.000	0.000	0.000	0.000	0.000	0.000	0.000	0.000	0.000	0.000	0.000	-1.453	-11.806	2.841	-4.539	4.904
Tr.2 Unit B1	Gegham 1	0.000	0.000	0.000	1.000	0.000	0.000	0.000	0.000	0.000	0.000	0.000	0.000	0.000	0.000	-0.667	-9.774	3.808	-4.829	6.872
Tr.2 Unit B1	Gegham 1	0.000	0.000	0.000	1.000	0.000	0.000	0.000	0.000	0.000	0.000	0.000	0.000	0.000	0.000	0.032	-11.421	2.791	-4.091	5.774
Tr.2 Unit B1	Gegham 1	0.000	0.000	0.000	1.000	0.000	0.000	0.000	0.000	0.000	0.000	0.000	0.000	0.000	0.000	-1.355	-10.215	3.731	-4.792	4.420
Tr.2 Unit B1	Gegham 1	0.000	0.000	0.000	1.000	0.000	0.000	0.000	0.000	0.000	0.000	0.000	0.000	0.000	0.000	0.325	-9.854	4.583	-5.329	7.379
Tr.2 Unit B1	Gegham 1	0.000	0.000	0.000	1.000	0.000	0.000	0.000	0.000	0.000	0.000	0.000	0.000	0.000	0.000	-0.926	-11.797	2.391	-4.208	4.635
Tr.2 Unit B1	Gegham 1	0.000	0.000	0.000	1.000	0.000	0.000	0.000	0.000	0.000	0.000	0.000	0.000	0.000	0.000	-0.012	-9.550	4.410	-4.794	5.104
Tr.2 Unit B1	Gegham 1	0.000	0.000	0.000	1.000	0.000	0.000	0.000	0.000	0.000	0.000	0.000	0.000	0.000	0.000	-0.543	-9.963	3.656	-4.576	6.134
Tr.2 Unit B1	Gegham 1	0.000	0.000	0.000	1.000	0.000	0.000	0.000	0.000	0.000	0.000	0.000	0.000	0.000	0.000	-0.468	-9.836	3.296	-4.176	4.723
Tr.2 Unit B1	Gegham 1	0.000	0.000	0.000	1.000	0.000	0.000	0.000	0.000	0.000	0.000	0.000	0.000	0.000	0.000	1.253	-9.372	3.461	-4.709	6.386
Tr.2 Unit B1	Gegham 1	0.000	0.000	0.000	1.000	0.000	0.000	0.000	0.000	0.000	0.000	0.000	0.000	0.000	0.000	-1.053	-11.382	2.678	-4.946	7.614
Tr2 A5 H5 S2	Syunik	0.000	0.000	0.000	0.000	0.000	0.000	0.000	0.000	0.000	0.000	0.000	0.000	1.000	0.000	-5.943	-13.021	-2.528	4.441	-1.448
Tr2 A5 H5 S2	Arteni	0.000	1.000	0.000	0.000	0.000	0.000	0.000	0.000	0.000	0.000	0.000	0.000	0.000	0.000	-4.124	-0.115	5.093	-3.101	-6.565
Tr2 A5 H5 S2	Gegham 1	0.000	0.000	0.000	1.000	0.000	0.000	0.000	0.000	0.000	0.000	0.000	0.000	0.000	0.000	1.566	-9.651	2.682	-3.952	2.419
Tr2 A5 H5 S2	Syunik	0.000	0.000	0.000	0.000	0.000	0.000	0.000	0.000	0.000	0.000	0.000	0.000	1.000	0.000	-6.056	-12.189	-2.609	3.679	-1.336
Tr2 A5 H5 S2	Gegham 1	0.000	0.000	0.000	1.000	0.000	0.000	0.000	0.000	0.000	0.000	0.000	0.000	0.000	0.000	-0.244	-10.669	3.790	-3.734	1.134
Tr2 A5 H5 S2	Syunik	0.000	0.000	0.000	0.000	0.000	0.000	0.000	0.000	0.000	0.000	0.000	0.000	1.000	0.000	-5.602	-11.477	-1.497	4.088	-1.699
Tr2 A5 H5 S2	Syunik	0.000	0.000	0.000	0.000	0.000	0.000	0.000	0.000	0.000	0.000	0.000	0.000	1.000	0.000	-5.613	-10.307	-2.913	4.111	-1.180
Tr2 A5 H5 S2	Kelbadjar	0.000	0.000	0.000	0.000	0.000	0.000	0.000	0.000	1.000	0.000	0.000	0.000	0.000	0.000	-9.482	-10.776	-0.160	4.945	-1.640
Tr2 A5 H5 S2	Syunik	0.000	0.000	0.000	0.000	0.000	0.000	0.000	0.000	0.000	0.000	0.000	0.000	1.000	0.000	-7.483	-13.485	-4.494	5.999	-0.109
Tr2 A5 H2 S2	Gegham 1	0.000	0.000	0.000	1.000	0.000	0.000	0.000	0.000	0.000	0.000	0.000	0.000	0.000	0.000	-0.339	-11.194	3.458	-3.966	2.172
Tr2 A5 H2 S2	Gegham 1	0.000	0.000	0.000	1.000	0.000	0.000	0.000	0.000	0.000	0.000	0.000	0.000	0.000	0.000	0.165	-10.022	4.583	-4.656	4.335
Tr2 A5 H2 S2	Gegham 1	0.000	0.000	0.000	1.000	0.000	0.000	0.000	0.000	0.000	0.000	0.000	0.000	0.000	0.000	-1.452	-11.926	2.821	-4.363	6.008
Tr2 A5 H2 S2	Arteni	0.000	1.000	0.000	0.000	0.000	0.000	0.000	0.000	0.000	0.000	0.000	0.000	0.000	0.000	-4.467	2.371	6.155	-3.344	-6.095
Tr2 A5 H2 S2	Gegham 1	0.000	0.000	0.000	1.000	0.000	0.000	0.000	0.000	0.000	0.000	0.000	0.000	0.000	0.000	-0.985	-12.218	3.711	-4.812	5.553
Tr2 A5 H2 S2	Gegham 1	0.000	0.000	0.000	1.000	0.000	0.000	0.000	0.000	0.000	0.000	0.000	0.000	0.000	0.000	0.565	-10.162	3.657	-5.065	4.395
Tr2 A5 H2 S2	Gegham 1	0.000	0.000	0.000	1.000	0.000	0.000	0.000	0.000	0.000	0.000	0.000	0.000	0.000	0.000	-0.543	-9.550	4.056	-4.824	5.751
Tr2 A5 H2 S2	Kelbadjar	0.000	0.000	0.000	0.000	0.000	0.000	0.000	0.000	1.000	0.000	0.000	0.000	0.000	0.000	-9.746	-9.323	-2.215	5.694	-1.875
TR.2 Unit-B0	Gegham 1	0.000	0.000	0.000	1.000	0.000	0.000	0.000	0.000	0.000	0.000	0.000	0.000	0.000	0.000	2.262	-9.797	3.572	-4.532	3.286

TR.2 Unit-B0	Gegham 1	0.000	0.000	0.000	1.000	0.000	0.000	0.000	0.000	0.000	0.000	0.000	0.000	0.000	0.000	-1.065	-11.147	2.878	-4.179	2.157
TR.2 Unit-B0	Syunik	0.000	0.000	0.000	0.000	0.000	0.000	0.000	0.000	0.000	0.000	0.000	0.000	1.000	0.000	-5.996	-12.628	-2.103	4.081	-1.322
TR.2 Unit-B0	Gegham 1	0.000	0.000	0.000	1.000	0.000	0.000	0.000	0.000	0.000	0.000	0.000	0.000	0.000	0.000	0.344	-11.636	3.168	-4.230	2.182
TR.2 Unit-B0	Gegham 1	0.000	0.000	0.000	1.000	0.000	0.000	0.000	0.000	0.000	0.000	0.000	0.000	0.000	0.000	1.075	-9.385	3.296	-5.065	4.151
TR.2 Unit-B0	Kelbadjar	0.000	0.000	0.000	0.000	0.000	0.000	0.000	0.000	1.000	0.000	0.000	0.000	0.000	0.000	-9.723	-9.115	0.795	3.950	-1.591
TR.2 Unit-B0	Khorapor	0.000	0.000	0.000	0.000	0.000	0.000	0.000	0.000	0.000	1.000	0.000	0.000	0.000	0.000	-6.322	-15.621	-4.781	-0.828	3.910
TR.2 Unit-B0	Gegham 1	0.000	0.000	0.000	1.000	0.000	0.000	0.000	0.000	0.000	0.000	0.000	0.000	0.000	0.000	0.478	-10.104	4.022	-4.985	6.032
Tr2 A5 H5 S1	Syunik	0.000	0.000	0.000	0.000	0.000	0.000	0.000	0.000	0.004	0.000	0.000	0.000	0.996	0.000	-6.615	-11.466	-1.041	5.570	-2.279
Tr2 A5 H5 S1	Gegham 1	0.000	0.000	0.000	1.000	0.000	0.000	0.000	0.000	0.000	0.000	0.000	0.000	0.000	0.000	-0.706	-9.718	3.125	-4.237	3.007
Tr2 A5 H5 S1	Gegham 1	0.000	0.000	0.000	1.000	0.000	0.000	0.000	0.000	0.000	0.000	0.000	0.000	0.000	0.000	-1.553	-11.560	3.303	-4.659	5.485
Tr2 A5 H5 S1	Gegham 1	0.000	0.000	0.000	1.000	0.000	0.000	0.000	0.000	0.000	0.000	0.000	0.000	0.000	0.000	-0.900	-11.591	3.218	-4.364	3.645
Tr2 A5 H5 S1	Kelbadjar	0.000	0.000	0.000	0.000	0.000	0.000	0.000	0.000	0.999	0.000	0.000	0.000	0.001	0.000	-6.365	-11.190	-0.688	2.673	-1.212
Tr2 A5 H5 S1	Syunik	0.000	0.000	0.000	0.000	0.000	0.000	0.000	0.000	0.000	0.000	0.000	0.000	1.000	0.000	-6.900	-11.636	-3.289	2.928	-0.517
Tr2 A5 H5 S1	Kelbadjar	0.000	0.000	0.000	0.000	0.000	0.000	0.000	0.000	0.877	0.000	0.000	0.000	0.123	0.000	-5.327	-9.939	-2.128	2.843	-0.973
Tr2 A5 H5 S1	Gegham 1	0.000	0.000	0.000	1.000	0.000	0.000	0.000	0.000	0.000	0.000	0.000	0.000	0.000	0.000	0.304	-8.931	4.546	-5.060	1.983
Tr2 A5 H5 S1	Gegham 1	0.000	0.000	0.000	1.000	0.000	0.000	0.000	0.000	0.000	0.000	0.000	0.000	0.000	0.000	-1.714	-11.356	2.771	-3.823	3.765
Tr2 A5 H5 S1	Tsaghkunyat	0.000	0.000	0.000	0.000	0.000	0.000	0.000	0.000	0.000	0.000	0.000	0.000	0.000	1.000	34.528	0.679	-6.169	4.088	-1.144
Tr2 A5 H5 S1	Gegham 1	0.000	0.000	0.000	1.000	0.000	0.000	0.000	0.000	0.000	0.000	0.000	0.000	0.000	0.000	-0.547	-9.335	4.506	-5.298	6.940
Tr2 A5 H5 S1	Gegham 1	0.000	0.000	0.000	1.000	0.000	0.000	0.000	0.000	0.000	0.000	0.000	0.000	0.000	0.000	-0.718	-10.460	3.156	-3.924	4.172
Tr2 A5 H5 S1	Gegham 1	0.000	0.000	0.000	1.000	0.000	0.000	0.000	0.000	0.000	0.000	0.000	0.000	0.000	0.000	-1.179	-10.836	4.076	-4.644	6.630
Tr2 A5 H5 S1	Syunik	0.000	0.000	0.000	0.000	0.000	0.000	0.000	0.000	0.000	0.000	0.000	0.000	1.000	0.000	-6.772	-11.516	-2.053	4.437	-1.158
TR.2 A4 H5 S	Gegham 1	0.000	0.000	0.000	1.000	0.000	0.000	0.000	0.000	0.000	0.000	0.000	0.000	0.000	0.000	-0.251	-12.407	2.728	-4.796	3.937
TR.2 A4 H5 S	Gegham 1	0.000	0.000	0.000	1.000	0.000	0.000	0.000	0.000	0.000	0.000	0.000	0.000	0.000	0.000	-1.175	-12.049	3.177	-4.256	3.103
TR.2 A4 H5 S	Gegham 1	0.000	0.000	0.000	1.000	0.000	0.000	0.000	0.000	0.000	0.000	0.000	0.000	0.000	0.000	-0.436	-9.730	3.731	-4.376	1.825
TR.2 A4 H5 S	Syunik	0.000	0.000	0.000	0.000	0.000	0.000	0.000	0.000	0.000	0.000	0.000	0.000	1.000	0.000	-7.018	-12.145	-2.206	2.509	-1.082
TR.2 A4 H5 S	Gegham 1	0.000	0.000	0.000	1.000	0.000	0.000	0.000	0.000	0.000	0.000	0.000	0.000	0.000	0.000	-0.534	-10.104	3.697	-4.677	3.966
TR.2 A4 H5 S	Gegham 1	0.000	0.000	0.000	1.000	0.000	0.000	0.000	0.000	0.000	0.000	0.000	0.000	0.000	0.000	-0.993	-10.503	2.818	-4.433	4.037
TR.2 A4 H5 S	Arteni	0.000	1.000	0.000	0.000	0.000	0.000	0.000	0.000	0.000	0.000	0.000	0.000	0.000	0.000	-4.766	0.453	5.338	-3.380	-6.691
TR.2 A4 H5 S	Gegham 1	0.000	0.000	0.000	1.000	0.000	0.000	0.000	0.000	0.000	0.000	0.000	0.000	0.000	0.000	-0.842	-10.669	3.376	-4.600	3.135
TR.2 A4 H5 S	Gegham 1	0.000	0.000	0.000	1.000	0.000	0.000	0.000	0.000	0.000	0.000	0.000	0.000	0.000	0.000	-1.000	-11.116	3.643	-4.533	4.340
TR.2 A4 H5 S	Gegham 1	0.000	0.000	0.000	1.000	0.000	0.000	0.000	0.000	0.000	0.000	0.000	0.000	0.000	0.000	-1.142	-11.305	3.260	-4.271	2.423
TR.2 A4 H5 S	Gegham 1	0.000	0.000	0.000	1.000	0.000	0.000	0.000	0.000	0.000	0.000	0.000	0.000	0.000	0.000	-0.448	-10.149	3.708	-4.413	3.242
TR.2 A4 H5 S	Kelbadjar	0.000	0.000	0.000	0.000	0.000	0.000	0.000	0.000	1.000	0.000	0.000	0.000	0.000	0.000	-8.323	-8.981	2.226	4.762	-1.695
TR.2 A4 H5 S	Gegham 1	0.000	0.000	0.000	1.000	0.000	0.000	0.000	0.000	0.000	0.000	0.000	0.000	0.000	0.000	-0.191	-11.969	3.298	-4.212	2.838
TR.2 A4 H5 S	Gegham 1	0.000	0.000	0.000	1.000	0.000	0.000	0.000	0.000	0.000	0.000	0.000	0.000	0.000	0.000	-1.004	-11.454	3.320	-4.252	2.146
TR.2 A4 H5 S	Gegham 1	0.000	0.000	0.000	1.000	0.000	0.000	0.000	0.000	0.000	0.000	0.000	0.000	0.000	0.000	-0.595	-12.053	2.532	-4.672	6.370
TR.2 A4 H5 S	Gegham 1	0.000	0.000	0.000	1.000	0.000	0.000	0.000	0.000	0.000	0.000	0.000	0.000	0.000	0.000	-1.332	-11.141	3.431	-4.542	3.314
TR.2 A4 H5 S	Gegham 1	0.000	0.000	0.000	1.000	0.000	0.000	0.000	0.000	0.000	0.000	0.000	0.000	0.000	0.000	-1.446	-11.820	3.219	-4.226	5.212
TR.2 A4 H5 S	Syunik	0.000	0.000	0.000	0.000	0.000	0.000	0.000	0.000	0.000	0.000	0.000	0.000	1.000	0.000	-7.827	-17.803	-3.368	2.351	-0.034
TR.2 A4 H5 S	Gegham 1	0.000	0.000	0.000	1.000	0.000	0.000	0.000	0.000	0.000	0.000	0.000	0.000	0.000	0.000	-0.046	-11.172	3.442	-4.694	3.350
TR.2 A4 H5 S	Gegham 1	0.000	0.000	0.000	1.000	0.000	0.000	0.000	0.000	0.000	0.000	0.000	0.000	0.000	0.000	-1.771	-11.660	2.952	-4.587	3.504
TR.2 A4 H5 S	Gegham 1	0.000	0.000	0.000	1.000	0.000	0.000	0.000	0.000	0.000	0.000	0.000	0.000	0.000	0.000	-1.074	-10.230	3.255	-4.581	4.077
TR.2 A4 H5 S	Gegham 1	0.000	0.000	0.000	1.000	0.000	0.000	0.000	0.000	0.000	0.000	0.000	0.000	0.000	0.000	0.177	-10.995	3.503	-4.823	3.719
TR.2 A4 H5 S	Gegham 1	0.000	0.000	0.000	1.000	0.000	0.000	0.000	0.000	0.000	0.000	0.000	0.000	0.000	0.000	1.178	-10.487	3.730	-4.933	4.338
TR.2 A4 H5 S	Kelbadjar	0.000	0.000	0.000	0.000	0.000	0.000	0.000	0.000	1.000	0.000	0.000	0.000	0.000	0.000	-9.460	-8.778	0.511	4.290	-1.517
TR.2 A4 H5 S	Gegham 1	0.000	0.000	0.000	1.000	0.000	0.000	0.000	0.000	0.000	0.000	0.000	0.000	0.000	0.000	-0.124	-8.848	3.910	-4.658	3.142
TR.2 A4 H5 S	Gegham 1	0.000	0.000	0.000	1.000	0.000	0.000	0.000	0.000	0.000	0.000	0.000	0.000	0.000	0.000	-1.016	-10.216	4.117	-4.958	8.122
TR.2 A4 H5 S	Arteni	0.000	1.000	0.000	0.000	0.000	0.000	0.000	0.000	0.000	0.000	0.000	0.000	0.000	0.000	-3.325	1.148	4.566	-3.510	-6.763
TR.2 A4 H5 S	Kelbadjar	0.000	0.000	0.000	0.000	0.000	0.000	0.000	0.000	1.000	0.000	0.000	0.000	0.000	0.000	-9.920	-9.076	-0.865	5.060	-2.126
TR.2 A4 H5 S	Gegham 1	0.000	0.000	0.000	1.000	0.000	0.000	0.000	0.000	0.000	0.000	0.000	0.000	0.000	0.000	0.353	-11.125	3.851	-4.706	5.123
Tr2 CO TS 01	Gegham 1	0.000	0.000	0.000	1.000	0.000	0.000	0.000	0.000	0.000	0.000	0.000	0.000	0.000	0.000	0.737	-10.552	2.435	-4.493	3.618
Tr2 CO TS 01	Kelbadjar	0.000	0.000	0.000	0.000	0.000	0.000	0.000	0.000	1.000	0.000	0.000	0.000	0.000	0.000	-9.994	-9.045	-0.807	5.584	-1.010
TR.2 B1 H1 S	Gegham 1	0.000	0.000	0.000	1.000	0.000	0.000	0.000	0.000	0.000	0.000	0.000	0.000	0.000	0.000	-0.937	-10.147	2.879	-4.605	3.545
TR.2 B1 H1 S	Kelbadjar	0.000	0.000	0.000	0.000	0.000	0.000	0.000	0.000	1.000	0.000	0.000	0.000	0.000	0.000	-8.047	-10.099	0.766	3.982	-0.913
TR.2 B1 H1 S	Kelbadjar	0.000	0.000	0.000	0.000	0.000	0.000	0.000	0.000	1.000	0.000	0.000	0.000	0.000	0.000	-9.754	-8.700	1.223	3.387	-1.642
TR.2 B1 H1 S	Gegham 1	0.000	0.000	0.000	1.000	0.000	0.000	0.000	0.000	0.000	0.000	0.000	0.000	0.000	0.000	-0.658	-11.788	3.594	-4.583	4.634
TR.2 B1 H1 S	Kelbadjar	0.000	0.000	0.000	0.000	0.000	0.000	0.000	0.000	1.000	0.000	0.000	0.000	0.000	0.000	-9.568	-9.517	0.726	3.771	-1.121
TR.2 B1 H1 S	Kelbadjar	0.000	0.000	0.000	0.000	0.000	0.000	0.000	0.000	1.000	0.000	0.000	0.000	0.000	0.000	-8.496	-8.448	0.711	2.421	-1.871
TR.2 B1 H1 S	Kelbadjar	0.000	0.000	0.000	0.000	0.000	0.000	0.000	0.000	1.000	0.000	0.000	0.000	0.000	0.000	-9.091	-8.907	0.856	4.930	-1.699
TR.2 B1 H1 S	Gegham 1	0.000	0.000	0.000	1.000	0.000	0.000	0.000	0.000	0.000	0.000	0.000	0.000	0.000	0.000	0.101	-12.031	2.777	-4.475	3.718
TR.2 B1 H1 S	Kelbadjar	0.000	0.000	0.000	0.000	0.000	0.000	0.000	0.000	1.000	0.000	0.000	0.000	0.000	0.000	-9.062	-8.980	2.220	3.212	-1.370
TR.2 B1 H1 S	Gegham 1	0.000	0.000	0.000	1.000	0.000	0.000	0.000	0.000	0.000	0.000	0.000	0.000	0.000	0.000	1.170	-9.331	4.258	-5.181	6.561
TR.2 B1 H1 S	Gegham 1	0.000	0.000	0.000	1.000	0.000	0.000	0.000	0.000	0.000	0.000	0.000	0.000	0.000	0.000	-2.083	-11.820	2.984	-4.200	3.994
TR.2 B1 H1 S	Kelbadjar	0.000	0.000	0.000	0.000	0.000	0.000	0.000	0.000	1.000	0.000	0.000	0.000	0.000	0.000	-9.722	-10.281	-0.486	5.049	-1.986
TR.2 B1 H1 S	Syunik	0.000	0.000	0.000	0.000	0.000	0.000	0.000	0.000	0.000	0.000	0.000	0.000	1.000	0.000	-6.760	-11.206	-1.933	2.303	-1.368
TR.2 B1 H1 S	Gegham 1	0.000	0.000	0.000	1.000	0.000	0.000	0.000	0.000	0.000	0.000	0.000	0.000	0.000	0.000	-0.311	-11.646	3.095	-4.477	4.511
TR.2 B1 H1 S	Kelbadjar	0.000	0.000	0.000	0.000	0.000	0.000	0.000	0.000	1.000	0.000	0.000	0.000	0.000	0.000	-9.669	-8.628	1.214	3.937	-1.404

TR.2 B1 H1 S	Kelbadjar	0.000	0.000	0.000	0.000	0.000	0.000	0.000	0.000	1.000	0.000	0.000	0.000	0.000	0.000	-9.357	-7.957	-0.081	4.365	-2.015
TR.2 B1 H1 S	Gegham 1	0.000	0.000	0.000	1.000	0.000	0.000	0.000	0.000	0.000	0.000	0.000	0.000	0.000	0.000	-0.221	-11.166	3.262	-4.317	5.953
TR.2 B1 H1 S	Arteni	0.000	1.000	0.000	0.000	0.000	0.000	0.000	0.000	0.000	0.000	0.000	0.000	0.000	0.000	-0.309	2.569	3.891	-1.754	-4.528
TR.2 B1 H1 S	Gegham 1	0.000	0.000	0.000	1.000	0.000	0.000	0.000	0.000	0.000	0.000	0.000	0.000	0.000	0.000	-0.354	-10.213	4.491	-5.203	7.348
TR.2 B1 H1 S	Gegham 1	0.000	0.000	0.000	1.000	0.000	0.000	0.000	0.000	0.000	0.000	0.000	0.000	0.000	0.000	-1.202	-9.118	6.628	-5.067	7.873
Tr2 A4 H5 S2	Gegham 1	0.000	0.000	0.000	1.000	0.000	0.000	0.000	0.000	0.000	0.000	0.000	0.000	0.000	0.000	-1.280	-10.917	3.337	-4.543	2.925
Tr2 A4 H5 S2	Gegham 1	0.000	0.000	0.000	1.000	0.000	0.000	0.000	0.000	0.000	0.000	0.000	0.000	0.000	0.000	0.240	-11.035	3.705	-4.852	3.167
Tr2 A4 H5 S2	Gegham 1	0.000	0.000	0.000	1.000	0.000	0.000	0.000	0.000	0.000	0.000	0.000	0.000	0.000	0.000	-1.421	-10.943	2.323	-4.219	4.330
Tr2 A4 H5 S2	Gegham 1	0.000	0.000	0.000	1.000	0.000	0.000	0.000	0.000	0.000	0.000	0.000	0.000	0.000	0.000	-0.271	-9.682	3.054	-4.719	4.207
Tr2 A4 H5 S2	Gegham 1	0.000	0.000	0.000	1.000	0.000	0.000	0.000	0.000	0.000	0.000	0.000	0.000	0.000	0.000	-1.239	-12.819	2.305	-4.377	4.144
Tr2 A4 H5 S2	Khorapor	0.000	0.000	0.000	0.000	0.000	0.000	0.000	0.000	0.000	1.000	0.000	0.000	0.000	0.000	-6.164	-17.356	-4.502	-0.538	3.912
Tr2 A4 H5 S2	Gegham 1	0.000	0.000	0.000	1.000	0.000	0.000	0.000	0.000	0.000	0.000	0.000	0.000	0.000	0.000	-1.801	-11.653	2.366	-4.375	3.900
Tr2 A4 H5 S2	Gegham 1	0.000	0.000	0.000	1.000	0.000	0.000	0.000	0.000	0.000	0.000	0.000	0.000	0.000	0.000	-0.664	-11.866	2.271	-4.399	4.242
Tr2 A4 H5 S2	Gegham 1	0.000	0.000	0.000	1.000	0.000	0.000	0.000	0.000	0.000	0.000	0.000	0.000	0.000	0.000	1.181	-10.271	3.740	-4.116	2.767
Tr2 HOR.3 S	Kelbadjar	0.000	0.000	0.000	0.000	0.000	0.000	0.000	0.000	1.000	0.000	0.000	0.000	0.000	0.000	-9.625	-9.955	1.872	2.519	-1.586
Tr2 HOR.3 S	Gegham 1	0.000	0.000	0.000	1.000	0.000	0.000	0.000	0.000	0.000	0.000	0.000	0.000	0.000	0.000	-0.407	-11.695	2.683	-4.787	3.415
Tr2 HOR.3 S	Kelbadjar	0.000	0.000	0.000	0.000	0.000	0.000	0.000	0.000	1.000	0.000	0.000	0.000	0.000	0.000	-9.882	-8.245	0.086	4.954	-1.876
Tr2 HOR.3 S	Kelbadjar	0.000	0.000	0.000	0.000	0.000	0.000	0.000	0.000	1.000	0.000	0.000	0.000	0.000	0.000	-8.431	-9.383	1.091	3.618	-1.266
Tr2 HOR.3 S	Gegham 1	0.000	0.000	0.000	1.000	0.000	0.000	0.000	0.000	0.000	0.000	0.000	0.000	0.000	0.000	-1.235	-9.746	3.716	-3.984	3.109
Tr2 HOR.3 S	Gegham 1	0.000	0.000	0.000	1.000	0.000	0.000	0.000	0.000	0.000	0.000	0.000	0.000	0.000	0.000	-0.728	-9.457	3.474	-4.618	3.388
Tr2 HOR.3 S	Gegham 1	0.000	0.000	0.000	1.000	0.000	0.000	0.000	0.000	0.000	0.000	0.000	0.000	0.000	0.000	-0.103	-12.275	3.032	-4.412	3.227
Tr2 HOR.3 S	Gegham 1	0.000	0.000	0.000	1.000	0.000	0.000	0.000	0.000	0.000	0.000	0.000	0.000	0.000	0.000	0.135	-11.160	2.848	-4.728	5.278
Tr2 HOR.3 S	Gegham 1	0.000	0.000	0.000	1.000	0.000	0.000	0.000	0.000	0.000	0.000	0.000	0.000	0.000	0.000	-1.256	-11.494	2.939	-4.029	3.402
Tr2 HOR.3 S	Syunik	0.000	0.000	0.000	0.000	0.000	0.000	0.000	0.000	0.000	0.000	0.000	0.000	1.000	0.000	-6.000	-11.727	-2.212	4.741	-1.805
Tr2 HOR.3 S	Kelbadjar	0.000	0.000	0.000	0.000	0.000	0.000	0.000	0.000	1.000	0.000	0.000	0.000	0.000	0.000	-10.242	-9.115	0.387	4.746	-1.973
Tr2 HOR.3 S	Syunik	0.000	0.000	0.000	0.000	0.000	0.000	0.000	0.000	0.000	0.000	0.000	0.000	1.000	0.000	-7.706	-13.835	-2.132	5.258	-1.928
Tr2 HOR.3 S	Gegham 1	0.000	0.000	0.000	1.000	0.000	0.000	0.000	0.000	0.000	0.000	0.000	0.000	0.000	0.000	-0.002	-10.114	3.031	-5.138	7.079
Tr2 A4 H3 S2	Kelbadjar	0.000	0.000	0.000	0.000	0.000	0.000	0.000	0.000	1.000	0.000	0.000	0.000	0.000	0.000	-9.275	-9.442	0.468	3.669	-1.603
Tr2 A4 H3 S2	Kelbadjar	0.000	0.000	0.000	0.000	0.000	0.000	0.000	0.000	1.000	0.000	0.000	0.000	0.000	0.000	-10.153	-9.397	1.913	4.790	-2.074
Tr2 A4 H3 S2	Gegham 1	0.000	0.000	0.000	1.000	0.000	0.000	0.000	0.000	0.000	0.000	0.000	0.000	0.000	0.000	-0.065	-11.032	3.610	-4.198	2.800
Tr2 A4 H3 S2	Syunik	0.000	0.000	0.000	0.000	0.000	0.000	0.000	0.000	0.000	0.000	0.000	0.000	1.000	0.000	-5.426	-13.276	-1.413	3.107	-0.520
Tr2 A4 H3 S2	Kelbadjar	0.000	0.000	0.000	0.000	0.000	0.000	0.000	0.000	1.000	0.000	0.000	0.000	0.000	0.000	-10.852	-9.440	-0.594	5.452	-1.879
Tr2 A4 H3 S2	Kelbadjar	0.000	0.000	0.000	0.000	0.000	0.000	0.000	0.000	1.000	0.000	0.000	0.000	0.000	0.000	-9.497	-10.875	1.826	4.322	-1.425
Tr2 A4 H3 S2	Gegham 1	0.000	0.000	0.000	1.000	0.000	0.000	0.000	0.000	0.000	0.000	0.000	0.000	0.000	0.000	-0.202	-10.725	3.615	-4.190	3.373
Tr2 A4 H3 S2	Arteni	0.000	1.000	0.000	0.000	0.000	0.000	0.000	0.000	0.000	0.000	0.000	0.000	0.000	0.000	-2.602	2.596	5.357	-2.688	-6.096
Tr2 A4 H3 S2	Arteni	0.000	1.000	0.000	0.000	0.000	0.000	0.000	0.000	0.000	0.000	0.000	0.000	0.000	0.000	-3.219	1.578	4.580	-3.308	-6.533
Tr2 A4 H3 S2	Gegham 1	0.000	0.000	0.000	1.000	0.000	0.000	0.000	0.000	0.000	0.000	0.000	0.000	0.000	0.000	0.545	-9.580	3.754	-5.074	4.528
Tr2 A4 H3 S2	Gegham 1	0.000	0.000	0.000	1.000	0.000	0.000	0.000	0.000	0.000	0.000	0.000	0.000	0.000	0.000	-1.551	-9.778	4.118	-4.513	4.245
Tr2 A4 H3 S2	Syunik	0.000	0.000	0.000	0.000	0.000	0.000	0.000	0.000	0.000	0.000	0.000	0.000	1.000	0.000	-8.547	-15.278	-5.350	7.234	-0.289
Tr2 A4 H3 S2	Gegham 1	0.000	0.000	0.000	1.000	0.000	0.000	0.000	0.000	0.000	0.000	0.000	0.000	0.000	0.000	-1.445	-9.978	3.638	-4.767	8.131
Tr2 A4 H3 S2	Kelbadjar	0.000	0.000	0.000	0.000	0.000	0.000	0.000	0.000	1.000	0.000	0.000	0.000	0.000	0.000	-8.805	-10.671	1.185	4.777	-1.427
Tr2 A4 H3 S2	Gegham 1	0.000	0.000	0.000	1.000	0.000	0.000	0.000	0.000	0.000	0.000	0.000	0.000	0.000	0.000	-1.327	-11.972	3.411	-4.312	4.158
Tr2 A4 H2 S2	Gegham 1	0.000	0.000	0.000	1.000	0.000	0.000	0.000	0.000	0.000	0.000	0.000	0.000	0.000	0.000	-0.603	-10.417	3.569	-4.884	2.744
Tr2 A4 H2 S2	Gegham 1	0.000	0.000	0.000	1.000	0.000	0.000	0.000	0.000	0.000	0.000	0.000	0.000	0.000	0.000	2.265	-10.311	2.465	-4.143	5.076
Tr2 A4 H2 S2	Gegham 1	0.000	0.000	0.000	1.000	0.000	0.000	0.000	0.000	0.000	0.000	0.000	0.000	0.000	0.000	-1.212	-12.576	2.268	-4.371	7.317
Tr2 A4 H2 S2	Chikiani	0.000	0.000	1.000	0.000	0.000	0.000	0.000	0.000	0.000	0.000	0.000	0.000	0.000	0.000	-0.990	3.796	-1.539	-2.463	6.897
Tr2 A4 H2 S2	Gegham 1	0.000	0.000	0.000	1.000	0.000	0.000	0.000	0.000	0.000	0.000	0.000	0.000	0.000	0.000	-0.719	-11.667	2.914	-4.250	3.941
Tr2 A4 H2 S2	Gegham 1	0.000	0.000	0.000	1.000	0.000	0.000	0.000	0.000	0.000	0.000	0.000	0.000	0.000	0.000	2.309	-9.615	2.830	-4.510	2.579
Tr2 A4 H2 S2	Gegham 1	0.000	0.000	0.000	1.000	0.000	0.000	0.000	0.000	0.000	0.000	0.000	0.000	0.000	0.000	0.887	-9.558	4.208	-4.689	4.402
Tr2 A4 H2 S2	Kelbadjar	0.000	0.000	0.000	0.000	0.000	0.000	0.000	0.000	1.000	0.000	0.000	0.000	0.000	0.000	-9.243	-9.706	0.748	3.793	-1.790
Tr2 A4 H2 S2	Gegham 1	0.000	0.000	0.000	1.000	0.000	0.000	0.000	0.000	0.000	0.000	0.000	0.000	0.000	0.000	-0.478	-11.449	3.499	-4.367	3.646
Tr2 A4 H2 S2	Gegham 1	0.000	0.000	0.000	1.000	0.000	0.000	0.000	0.000	0.000	0.000	0.000	0.000	0.000	0.000	0.450	-8.548	4.556	-5.347	4.967
Tr2 A4 H2 S2	Kelbadjar	0.000	0.000	0.000	0.000	0.000	0.000	0.000	0.000	1.000	0.000	0.000	0.000	0.000	0.000	-9.086	-8.380	1.170	3.632	-2.279
Tr2 A4 H2 S2	Gegham 1	0.000	0.000	0.000	1.000	0.000	0.000	0.000	0.000	0.000	0.000	0.000	0.000	0.000	0.000	-0.775	-10.901	3.539	-4.464	3.320
Tr2 A4 H2 S2	Gegham 1	0.000	0.000	0.000	1.000	0.000	0.000	0.000	0.000	0.000	0.000	0.000	0.000	0.000	0.000	-0.675	-10.500	3.478	-4.114	3.484
Tr2 A4 H2 S2	Gegham 1	0.000	0.000	0.000	1.000	0.000	0.000	0.000	0.000	0.000	0.000	0.000	0.000	0.000	0.000	0.397	-9.622	4.666	-4.733	2.823
Tr2 A4 H2 S2	Gegham 1	0.000	0.000	0.000	1.000	0.000	0.000	0.000	0.000	0.000	0.000	0.000	0.000	0.000	0.000	1.967	-11.009	2.995	-4.655	3.860
Tr2 A4 H2 S2	Gegham 1	0.000	0.000	0.000	1.000	0.000	0.000	0.000	0.000	0.000	0.000	0.000	0.000	0.000	0.000	-0.760	-10.737	3.330	-4.794	5.623
Tr2 A4 H2 S2	Gegham 1	0.000	0.000	0.000	1.000	0.000	0.000	0.000	0.000	0.000	0.000	0.000	0.000	0.000	0.000	1.255	-10.142	3.459	-5.257	5.879
Tr2 A4 H2 S2	Gegham 1	0.000	0.000	0.000	1.000	0.000	0.000	0.000	0.000	0.000	0.000	0.000	0.000	0.000	0.000	0.015	-9.120	4.013	-4.740	4.497
Tr2 A4 H2 S2	Gegham 1	0.000	0.000	0.000	1.000	0.000	0.000	0.000	0.000	0.000	0.000	0.000	0.000	0.000	0.000	0.294	-11.338	4.580	-4.561	4.626
Tr2 A4 H2 S2	Gegham 1	0.000	0.000	0.000	1.000	0.000	0.000	0.000	0.000	0.000	0.000	0.000	0.000	0.000	0.000	-0.208	-8.645	3.917	-5.124	6.750
Tr2 A4 H2 S2	Gegham 1	0.000	0.000	0.000	1.000	0.000	0.000	0.000	0.000	0.000	0.000	0.000	0.000	0.000	0.000	-0.543	-10.483	3.349	-3.962	3.351
Tr2 A4 H2 S2	Gegham 1	0.000	0.000	0.000	1.000	0.000	0.000	0.000	0.000	0.000	0.000	0.000	0.000	0.000	0.000	0.276	-9.936	3.125	-4.245	3.966
TR.2 Un.5 H1	Gegham 1	0.000	0.000	0.000	1.000	0.000	0.000	0.000	0.000	0.000	0.000	0.000	0.000	0.000	0.000	-1.377	-12.300	2.968	-4.136	2.599
TR.2 Un.5 H1	Syunik	0.000	0.000	0.000	0.000	0.000	0.000	0.000	0.000	0.000	0.000	0.000	0.000	1.000	0.000	-6.400	-11.724	-1.175	4.393	-0.970
TR.2 Un.5 H1	Gegham 1	0.000	0.000	0.000	1.000	0.000	0.000	0.000	0.000	0.000	0.000	0.000	0.000	0.000	0.000	-1.365	-10.798	2.931	-3.583	1.718

TR.2 Un.5 H1	Gegham 1	0.000	0.000	0.000	1.000	0.000	0.000	0.000	0.000	0.000	0.000	0.000	0.000	0.000	0.000	-0.385	-11.798	1.029	-4.163	4.195
TR.2 Un.5 H1	Syunik	0.000	0.000	0.000	0.000	0.000	0.000	0.000	0.000	0.000	0.000	0.000	0.000	1.000	0.000	-5.183	-11.473	-1.549	3.038	-1.142
TR.2 Un.5 H1	Syunik	0.000	0.000	0.000	0.000	0.000	0.000	0.000	0.000	0.001	0.000	0.000	0.000	0.999	0.000	-6.409	-12.176	-1.456	3.517	-0.894
TR.2 Un.5 H1	Kelbadjar	0.000	0.000	0.000	0.000	0.000	0.000	0.000	0.000	1.000	0.000	0.000	0.000	0.000	0.000	-8.897	-10.679	1.463	4.202	-1.712
TR.2 Un.5 H1	Kelbadjar	0.000	0.000	0.000	0.000	0.000	0.000	0.000	0.000	1.000	0.000	0.000	0.000	0.000	0.000	-9.280	-9.685	0.550	4.082	-0.989
TR.2 Un.5 H1	Kelbadjar	0.000	0.000	0.000	0.000	0.000	0.000	0.000	0.000	1.000	0.000	0.000	0.000	0.000	0.000	-8.826	-8.886	1.915	2.934	-1.715
TR.2 Un.5 H1	Gegham 1	0.000	0.000	0.000	1.000	0.000	0.000	0.000	0.000	0.000	0.000	0.000	0.000	0.000	0.000	-0.691	-11.655	3.372	-4.616	2.304
TR.2 Un.5 H1	Gegham 1	0.000	0.000	0.000	1.000	0.000	0.000	0.000	0.000	0.000	0.000	0.000	0.000	0.000	0.000	-1.184	-10.276	2.953	-4.030	2.405
TR.2 Un.5 H1	Kelbadjar	0.000	0.000	0.000	0.000	0.000	0.000	0.000	0.000	1.000	0.000	0.000	0.000	0.000	0.000	-8.535	-9.978	0.386	3.268	-0.973
TR.2 Un.5 H1	Kelbadjar	0.000	0.000	0.000	0.000	0.000	0.000	0.000	0.000	1.000	0.000	0.000	0.000	0.000	0.000	-10.030	-8.991	2.059	4.654	-2.342
TR.2 Un.5 H1	Gegham 1	0.000	0.000	0.000	1.000	0.000	0.000	0.000	0.000	0.000	0.000	0.000	0.000	0.000	0.000	-0.570	-10.908	4.399	-4.548	2.857
TR.2 Un.5 H1	Gegham 1	0.000	0.000	0.000	1.000	0.000	0.000	0.000	0.000	0.000	0.000	0.000	0.000	0.000	0.000	-1.682	-11.333	2.803	-4.088	2.767
TR.2 Un.5 H1	Gegham 1	0.000	0.000	0.000	1.000	0.000	0.000	0.000	0.000	0.000	0.000	0.000	0.000	0.000	0.000	-2.054	-13.177	1.895	-3.622	3.162
TR.2 Un.5 H1	Kelbadjar	0.000	0.000	0.000	0.000	0.000	0.000	0.000	0.000	1.000	0.000	0.000	0.000	0.000	0.000	-9.824	-10.744	2.043	4.446	-1.389
TR.2 Un.5 H1	Gegham 1	0.000	0.000	0.000	1.000	0.000	0.000	0.000	0.000	0.000	0.000	0.000	0.000	0.000	0.000	-1.017	-9.914	3.916	-4.632	2.919
TR.2 Un.5 H1	Kelbadjar	0.000	0.000	0.000	0.000	0.000	0.000	0.000	0.000	1.000	0.000	0.000	0.000	0.000	0.000	-9.604	-9.546	0.282	5.068	-2.084
TR.2 Un.5 H1	Gegham 1	0.000	0.000	0.000	1.000	0.000	0.000	0.000	0.000	0.000	0.000	0.000	0.000	0.000	0.000	-0.505	-9.512	3.927	-4.616	4.109
TR.2 Un.5 H1	Gutansar	0.000	0.000	0.000	0.000	0.000	1.000	0.000	0.000	0.000	0.000	0.000	0.000	0.000	0.000	5.182	0.775	11.872	13.585	-7.578
TR.2 Un.5 H1	Kelbadjar	0.000	0.000	0.000	0.000	0.000	0.000	0.000	0.000	1.000	0.000	0.000	0.000	0.000	0.000	-9.722	-9.355	1.044	3.129	-1.558
TR.2 Un.5 H1	Kelbadjar	0.000	0.000	0.000	0.000	0.000	0.000	0.000	0.000	0.979	0.000	0.000	0.000	0.021	0.000	-6.128	-10.745	-0.368	3.597	-1.936
TR.2 Un.5 H1	Gegham 1	0.000	0.000	0.000	1.000	0.000	0.000	0.000	0.000	0.000	0.000	0.000	0.000	0.000	0.000	-1.300	-12.414	2.342	-4.502	3.133
TR.2 Un.5 H1	Arteni	0.000	0.973	0.000	0.000	0.027	0.000	0.000	0.000	0.000	0.000	0.000	0.000	0.000	0.000	-0.929	3.412	3.747	-4.168	-6.574
TR.2 Un.5 H1	Gegham 1	0.000	0.000	0.000	1.000	0.000	0.000	0.000	0.000	0.000	0.000	0.000	0.000	0.000	0.000	-0.947	-10.733	2.944	-4.242	2.908
TR.2 Un.5 H1	Arteni	0.000	1.000	0.000	0.000	0.000	0.000	0.000	0.000	0.000	0.000	0.000	0.000	0.000	0.000	-4.344	0.097	5.244	-3.650	-8.545
TR.2 Un.5 H1	Gegham 1	0.000	0.000	0.000	1.000	0.000	0.000	0.000	0.000	0.000	0.000	0.000	0.000	0.000	0.000	-0.665	-11.779	2.978	-4.365	3.874
TR.2 Un.5 H1	Syunik	0.000	0.000	0.000	0.000	0.000	0.000	0.000	0.000	0.000	0.000	0.000	0.000	1.000	0.000	-6.315	-13.338	-1.599	2.709	-0.525
TR.2 Un.5 H1	Gegham 1	0.000	0.000	0.000	1.000	0.000	0.000	0.000	0.000	0.000	0.000	0.000	0.000	0.000	0.000	-1.605	-10.435	3.343	-4.121	1.797
TR.2 Un.5 H1	Kelbadjar	0.000	0.000	0.000	0.000	0.000	0.000	0.000	0.000	1.000	0.000	0.000	0.000	0.000	0.000	-8.366	-9.039	1.400	3.643	-1.024
TR.2 Un.5 H1	Gegham 1	0.000	0.000	0.000	1.000	0.000	0.000	0.000	0.000	0.000	0.000	0.000	0.000	0.000	0.000	-0.497	-10.642	2.708	-4.884	5.976
TR.2 Un.5 H1	Gegham 1	0.000	0.000	0.000	1.000	0.000	0.000	0.000	0.000	0.000	0.000	0.000	0.000	0.000	0.000	-0.540	-10.542	3.435	-4.353	2.521
TR.2 Un.5 H1	Gegham 1	0.000	0.000	0.000	1.000	0.000	0.000	0.000	0.000	0.000	0.000	0.000	0.000	0.000	0.000	-1.031	-11.063	3.330	-4.867	5.636
TR.2 Un.5 H1	Gegham 1	0.000	0.000	0.000	1.000	0.000	0.000	0.000	0.000	0.000	0.000	0.000	0.000	0.000	0.000	-1.474	-12.296	2.635	-4.114	2.870
TR.2 Un.5 H1	Kelbadjar	0.000	0.000	0.000	0.000	0.000	0.000	0.000	0.000	1.000	0.000	0.000	0.000	0.000	0.000	-10.176	-9.325	1.115	6.695	-2.281
TR.2 Un.5 H1	Kelbadjar	0.000	0.000	0.000	0.000	0.000	0.000	0.000	0.000	1.000	0.000	0.000	0.000	0.000	0.000	-8.397	-8.771	2.311	2.656	-1.799
TR.2 Un.5 H1	Kelbadjar	0.000	0.000	0.000	0.000	0.000	0.000	0.000	0.000	1.000	0.000	0.000	0.000	0.000	0.000	-8.252	-9.241	2.521	2.533	-1.293
TR.2 Un.5 H1	Kelbadjar	0.000	0.000	0.000	0.000	0.000	0.000	0.000	0.000	1.000	0.000	0.000	0.000	0.000	0.000	-8.162	-9.785	0.660	3.201	-0.582
TR.2 Un.5 H1	Gegham 1	0.000	0.000	0.000	1.000	0.000	0.000	0.000	0.000	0.000	0.000	0.000	0.000	0.000	0.000	-1.092	-10.871	4.030	-3.994	2.544
TR.2 Un.5 H1	Kelbadjar	0.000	0.000	0.000	0.000	0.000	0.000	0.000	0.000	1.000	0.000	0.000	0.000	0.000	0.000	-9.023	-9.544	0.183	4.025	-1.163
TR.2 Un.5 H1	Kelbadjar	0.000	0.000	0.000	0.000	0.000	0.000	0.000	0.000	0.545	0.000	0.000	0.000	0.455	0.000	-8.478	-10.248	-1.798	5.038	-1.879
TR.2 Un.5 H1	Gegham 1	0.000	0.000	0.000	1.000	0.000	0.000	0.000	0.000	0.000	0.000	0.000	0.000	0.000	0.000	-2.066	-11.157	2.778	-4.061	3.511
TR.2 Un.5 H1	Gegham 1	0.000	0.000	0.000	1.000	0.000	0.000	0.000	0.000	0.000	0.000	0.000	0.000	0.000	0.000	-1.834	-10.820	2.452	-3.975	1.713
TR.2 Un.5 H1	Gegham 1	0.000	0.000	0.000	1.000	0.000	0.000	0.000	0.000	0.000	0.000	0.000	0.000	0.000	0.000	-0.572	-10.666	3.054	-4.367	3.161
TR.2 Un.5 H1	Kelbadjar	0.000	0.000	0.000	0.000	0.000	0.000	0.000	0.000	1.000	0.000	0.000	0.000	0.000	0.000	-8.798	-9.228	1.179	2.254	-0.796
TR.2 Un.5 H1	Kelbadjar	0.000	0.000	0.000	0.000	0.000	0.000	0.000	0.000	1.000	0.000	0.000	0.000	0.000	0.000	-9.029	-9.829	0.867	3.531	-0.992
TR.2 Un.5 H1	Gegham 1	0.000	0.000	0.000	1.000	0.000	0.000	0.000	0.000	0.000	0.000	0.000	0.000	0.000	0.000	-1.449	-12.490	2.112	-4.380	4.468
TR.2 Un.5 H1	Kelbadjar	0.000	0.000	0.000	0.000	0.000	0.000	0.000	0.000	1.000	0.000	0.000	0.000	0.000	0.000	-7.988	-8.516	-0.317	4.233	-1.382
TR.2 Un.5 H1	Gegham 1	0.000	0.000	0.000	1.000	0.000	0.000	0.000	0.000	0.000	0.000	0.000	0.000	0.000	0.000	-0.289	-10.291	2.923	-4.917	4.575
TR.2 Un.5 H1	Kelbadjar	0.000	0.000	0.000	0.000	0.000	0.000	0.000	0.000	1.000	0.000	0.000	0.000	0.000	0.000	-7.903	-8.574	0.834	3.546	-1.399
TR.2 Un.5 H1	Gegham 1	0.000	0.000	0.000	1.000	0.000	0.000	0.000	0.000	0.000	0.000	0.000	0.000	0.000	0.000	3.631	-10.228	4.561	-4.699	4.383
TR.2 Un.5 H1	Kelbadjar	0.000	0.000	0.000	0.000	0.000	0.000	0.000	0.000	1.000	0.000	0.000	0.000	0.000	0.000	-9.885	-9.988	-0.144	4.192	-1.691
TR.2 Un.5 H1	Gegham 1	0.000	0.000	0.000	1.000	0.000	0.000	0.000	0.000	0.000	0.000	0.000	0.000	0.000	0.000	-0.185	-9.824	3.701	-4.406	2.898
TR.2 Un.5 H1	Gegham 1	0.000	0.000	0.000	1.000	0.000	0.000	0.000	0.000	0.000	0.000	0.000	0.000	0.000	0.000	0.206	-7.862	4.107	-5.277	6.718
TR.2 Un.5 H1	Gegham 1	0.000	0.000	0.000	1.000	0.000	0.000	0.000	0.000	0.000	0.000	0.000	0.000	0.000	0.000	-0.394	-11.250	3.816	-4.758	2.246
TR.2 Un.5 H1	Gegham 1	0.000	0.000	0.000	1.000	0.000	0.000	0.000	0.000	0.000	0.000	0.000	0.000	0.000	0.000	0.281	-9.228	4.594	-5.055	3.523
TR.2 Un.5 H1	Kelbadjar	0.000	0.000	0.000	0.000	0.000	0.000	0.000	0.000	1.000	0.000	0.000	0.000	0.000	0.000	-8.962	-9.795	-0.123	4.082	-1.963
TR.2 Un.5 H1	Gegham 1	0.000	0.000	0.000	1.000	0.000	0.000	0.000	0.000	0.000	0.000	0.000	0.000	0.000	0.000	-1.969	-11.362	3.451	-4.515	5.406
TR.2 Un.5 H1	Gegham 1	0.000	0.000	0.000	1.000	0.000	0.000	0.000	0.000	0.000	0.000	0.000	0.000	0.000	0.000	0.910	-9.543	3.203	-4.946	5.745
TR.2 Un.5 H1	Gegham 1	0.000	0.000	0.000	1.000	0.000	0.000	0.000	0.000	0.000	0.000	0.000	0.000	0.000	0.000	0.322	-8.802	2.958	-4.777	4.785
TR.2 Un.5 H1	Gegham 1	0.000	0.000	0.000	1.000	0.000	0.000	0.000	0.000	0.000	0.000	0.000	0.000	0.000	0.000	-1.638	-12.035	2.562	-3.674	2.399
TR.2 Un.5 H1	Kelbadjar	0.000	0.000	0.000	0.000	0.000	0.000	0.000	0.000	1.000	0.000	0.000	0.000	0.000	0.000	-9.579	-9.114	-0.261	4.717	-2.181
TR.2 Un.5 H1	Gegham 1	0.000	0.000	0.000	1.000	0.000	0.000	0.000	0.000	0.000	0.000	0.000	0.000	0.000	0.000	1.346	-11.430	2.172	-4.534	6.323
TR.2 Un.5 H1	Kelbadjar	0.000	0.000	0.000	0.000	0.000	0.000	0.000	0.000	1.000	0.000	0.000	0.000	0.000	0.000	-8.434	-8.432	1.241	4.757	-2.132
TR.2 Un.5 H1	Gegham 1	0.000	0.000	0.000	1.000	0.000	0.000	0.000	0.000	0.000	0.000	0.000	0.000	0.000	0.000	-1.513	-11.042	3.231	-4.447	4.082
TR.2 Un.5 H1	Gegham 1	0.000	0.000	0.000	1.000	0.000	0.000	0.000	0.000	0.000	0.000	0.000	0.000	0.000	0.000	1.683	-10.933	2.381	-4.693	5.607
TR.2 Un.5 H1	Kelbadjar	0.000	0.000	0.000	0.000	0.000	0.000	0.000	0.000	1.000	0.000	0.000	0.000	0.000	0.000	-10.685	-9.868	-0.621	5.482	-1.680
TR.2 Un.5 H1	Gegham 1	0.000	0.000	0.000	1.000	0.000	0.000	0.000	0.000	0.000	0.000	0.000	0.000	0.000	0.000	-0.817	-10.285	3.214	-4.531	6.944
TR2 UN A1-A	Kelbadjar	0.000	0.000	0.000	0.000	0.000	0.000	0.000	0.000	1.000	0.000	0.000	0.000	0.000	0.000	-9.654	-11.059	0.610	4.176	-0.971

TR2 UN A1-A	Kelbadjar	0.000	0.000	0.000	0.000	0.000	0.000	0.000	0.000	1.000	0.000	0.000	0.000	0.000	0.000	-9.017	-9.817	1.335	3.797	-1.715
TR2 UN A1-A	Kelbadjar	0.000	0.000	0.000	0.000	0.000	0.000	0.000	0.000	1.000	0.000	0.000	0.000	0.000	0.000	-7.886	-9.466	1.105	2.254	-0.837
TR2 UN A1-A	Gegham 1	0.000	0.000	0.000	1.000	0.000	0.000	0.000	0.000	0.000	0.000	0.000	0.000	0.000	0.000	-1.402	-10.693	2.711	-4.423	3.433
TR2 UN A1-A	Kelbadjar	0.000	0.000	0.000	0.000	0.000	0.000	0.000	0.000	1.000	0.000	0.000	0.000	0.000	0.000	-8.762	-9.459	1.360	3.336	-1.307
TR2 UN A1-A	Gegham 1	0.000	0.000	0.000	1.000	0.000	0.000	0.000	0.000	0.000	0.000	0.000	0.000	0.000	0.000	-1.305	-11.998	3.074	-4.239	2.414
TR2 UN A1-A	Gegham 1	0.000	0.000	0.000	1.000	0.000	0.000	0.000	0.000	0.000	0.000	0.000	0.000	0.000	0.000	-1.240	-11.275	3.100	-4.610	3.536
TR2 UN A1-A	Gegham 1	0.000	0.000	0.000	1.000	0.000	0.000	0.000	0.000	0.000	0.000	0.000	0.000	0.000	0.000	-1.448	-11.452	2.570	-4.854	4.915
TR2 UN A1-A	Kelbadjar	0.000	0.000	0.000	0.000	0.000	0.000	0.000	0.000	1.000	0.000	0.000	0.000	0.000	0.000	-10.010	-9.343	0.346	4.915	-2.191
TR2 UN A1-A	Gegham 1	0.000	0.000	0.000	1.000	0.000	0.000	0.000	0.000	0.000	0.000	0.000	0.000	0.000	0.000	-1.028	-10.659	4.069	-3.431	2.101
TR2 UN A1-A	Kelbadjar	0.000	0.000	0.000	0.000	0.000	0.000	0.000	0.000	1.000	0.000	0.000	0.000	0.000	0.000	-9.421	-11.318	0.188	5.033	-1.089
TR2 UN A1-A	Syunik	0.000	0.000	0.000	0.000	0.000	0.000	0.000	0.000	0.000	0.000	0.000	0.000	1.000	0.000	-6.012	-11.875	-1.817	4.463	-1.473
TR2 UN A1-A	Kelbadjar	0.000	0.000	0.000	0.000	0.000	0.000	0.000	0.000	1.000	0.000	0.000	0.000	0.000	0.000	-9.609	-9.874	0.034	3.851	-1.178
TR2 UN A1-A	Gegham 1	0.000	0.000	0.000	1.000	0.000	0.000	0.000	0.000	0.000	0.000	0.000	0.000	0.000	0.000	0.262	-11.128	3.217	-4.922	3.408
TR2 UN A1-A	Syunik	0.000	0.000	0.000	0.000	0.000	0.000	0.000	0.000	0.000	0.000	0.000	0.000	1.000	0.000	-7.188	-12.720	-2.542	3.893	-1.283
TR2 UN A1-A	Gegham 1	0.000	0.000	0.000	1.000	0.000	0.000	0.000	0.000	0.000	0.000	0.000	0.000	0.000	0.000	-1.083	-10.177	3.037	-4.147	3.336
TR2 UN A1-A	Arteni	0.000	1.000	0.000	0.000	0.000	0.000	0.000	0.000	0.000	0.000	0.000	0.000	0.000	0.000	-1.041	1.328	3.966	-4.259	-7.714
TR2 UN A1-A	Gegham 1	0.000	0.000	0.000	1.000	0.000	0.000	0.000	0.000	0.000	0.000	0.000	0.000	0.000	0.000	-2.135	-11.488	2.371	-4.100	2.276
TR2 UN A1-A	Gegham 1	0.000	0.000	0.000	1.000	0.000	0.000	0.000	0.000	0.000	0.000	0.000	0.000	0.000	0.000	-1.564	-9.421	4.011	-4.832	3.721
TR2 UN A1-A	Kelbadjar	0.000	0.000	0.000	0.000	0.000	0.000	0.000	0.000	1.000	0.000	0.000	0.000	0.000	0.000	-8.718	-9.381	1.603	2.689	-1.100
TR2 UN A1-A	Kelbadjar	0.000	0.000	0.000	0.000	0.000	0.000	0.000	0.000	1.000	0.000	0.000	0.000	0.000	0.000	-9.096	-9.895	2.019	3.050	-1.494
TR2 UN A1-A	Gegham 1	0.000	0.000	0.000	1.000	0.000	0.000	0.000	0.000	0.000	0.000	0.000	0.000	0.000	0.000	-0.574	-9.979	3.662	-4.628	2.832
TR2 UN A1-A	Arteni	0.000	1.000	0.000	0.000	0.000	0.000	0.000	0.000	0.000	0.000	0.000	0.000	0.000	0.000	-4.667	-0.177	4.809	-3.155	-6.241
TR2 UN A1-A	Gegham 1	0.000	0.000	0.000	1.000	0.000	0.000	0.000	0.000	0.000	0.000	0.000	0.000	0.000	0.000	0.187	-10.273	3.162	-4.462	5.349
TR2 UN A1-A	Gegham 1	0.000	0.000	0.000	1.000	0.000	0.000	0.000	0.000	0.000	0.000	0.000	0.000	0.000	0.000	-1.803	-12.216	2.841	-4.617	3.953
TR2 UN A1-A	Kelbadjar	0.000	0.000	0.000	0.000	0.000	0.000	0.000	0.000	1.000	0.000	0.000	0.000	0.000	0.000	-8.397	-9.522	-0.387	3.062	-1.310
TR2 UN A1-A	Gegham 1	0.000	0.000	0.000	1.000	0.000	0.000	0.000	0.000	0.000	0.000	0.000	0.000	0.000	0.000	-1.094	-10.470	3.436	-4.570	3.473
TR2 UN A1-A	Arteni	0.000	1.000	0.000	0.000	0.000	0.000	0.000	0.000	0.000	0.000	0.000	0.000	0.000	0.000	-4.670	0.597	6.875	-3.160	-9.358
TR2 UN A1-A	Kelbadjar	0.000	0.000	0.000	0.000	0.000	0.000	0.000	0.000	1.000	0.000	0.000	0.000	0.000	0.000	-9.398	-10.413	0.235	4.128	-1.280
TR2 UN A1-A	Gegham 1	0.000	0.000	0.000	1.000	0.000	0.000	0.000	0.000	0.000	0.000	0.000	0.000	0.000	0.000	0.276	-10.138	4.031	-4.648	4.062
TR2 UN A1-A	Gegham 1	0.000	0.000	0.000	1.000	0.000	0.000	0.000	0.000	0.000	0.000	0.000	0.000	0.000	0.000	-1.598	-11.642	2.923	-4.606	6.370
TR2 UN A1-A	Kelbadjar	0.000	0.000	0.000	0.000	0.000	0.000	0.000	0.000	1.000	0.000	0.000	0.000	0.000	0.000	-10.523	-9.582	0.614	4.073	-1.615
TR2 UN A1-A	Gegham 1	0.000	0.000	0.000	1.000	0.000	0.000	0.000	0.000	0.000	0.000	0.000	0.000	0.000	0.000	-0.953	-11.860	2.740	-4.514	5.818
TR2 UN A1-A	Gegham 1	0.000	0.000	0.000	1.000	0.000	0.000	0.000	0.000	0.000	0.000	0.000	0.000	0.000	0.000	1.299	-10.917	3.433	-4.337	3.782
TR2 UN A1-A	Gegham 1	0.000	0.000	0.000	1.000	0.000	0.000	0.000	0.000	0.000	0.000	0.000	0.000	0.000	0.000	0.309	-10.847	3.733	-5.303	4.917
TR2 UN A1-A	Kelbadjar	0.000	0.000	0.000	0.000	0.000	0.000	0.000	0.000	1.000	0.000	0.000	0.000	0.000	0.000	-9.033	-9.524	0.686	4.865	-1.894
TR2 UN A1-A	Gegham 1	0.000	0.000	0.000	1.000	0.000	0.000	0.000	0.000	0.000	0.000	0.000	0.000	0.000	0.000	-1.099	-10.563	4.187	-4.473	1.559
TR2 UN A1-A	Gegham 1	0.000	0.000	0.000	1.000	0.000	0.000	0.000	0.000	0.000	0.000	0.000	0.000	0.000	0.000	-0.740	-10.675	3.968	-4.523	2.954
TR2 UN A1-A	Gegham 1	0.000	0.000	0.000	1.000	0.000	0.000	0.000	0.000	0.000	0.000	0.000	0.000	0.000	0.000	-0.914	-12.057	3.492	-4.738	4.522
TR2 UN A1-A	Gegham 1	0.000	0.000	0.000	1.000	0.000	0.000	0.000	0.000	0.000	0.000	0.000	0.000	0.000	0.000	-1.764	-10.673	2.642	-4.053	4.363
TR2 UN A1-A	Kelbadjar	0.000	0.000	0.000	0.000	0.000	0.000	0.000	0.000	1.000	0.000	0.000	0.000	0.000	0.000	-9.047	-10.715	1.797	4.222	-1.739
TR2 UN A1-A	Gegham 1	0.000	0.000	0.000	1.000	0.000	0.000	0.000	0.000	0.000	0.000	0.000	0.000	0.000	0.000	-0.625	-11.443	3.420	-4.668	3.792
TR2 UN A1-A	Gegham 1	0.000	0.000	0.000	1.000	0.000	0.000	0.000	0.000	0.000	0.000	0.000	0.000	0.000	0.000	-0.757	-10.027	3.056	-4.431	4.784
TR2 UN A1-A	Gegham 1	0.000	0.000	0.000	1.000	0.000	0.000	0.000	0.000	0.000	0.000	0.000	0.000	0.000	0.000	0.272	-8.644	4.872	-4.556	3.433
TR2 UN A1-A	Arteni	0.000	0.677	0.000	0.000	0.323	0.000	0.000	0.000	0.000	0.000	0.000	0.000	0.000	0.000	1.671	2.918	3.239	-3.163	-6.767
TR2 UN A1-A	Gegham 1	0.000	0.000	0.000	1.000	0.000	0.000	0.000	0.000	0.000	0.000	0.000	0.000	0.000	0.000	-0.957	-10.785	2.996	-4.008	2.641
TR2 UN A1-A	Gegham 1	0.000	0.000	0.000	1.000	0.000	0.000	0.000	0.000	0.000	0.000	0.000	0.000	0.000	0.000	-0.464	-10.560	3.987	-5.006	4.029
TR2 UN A1-A	Gegham 1	0.000	0.000	0.000	1.000	0.000	0.000	0.000	0.000	0.000	0.000	0.000	0.000	0.000	0.000	0.610	-9.806	4.280	-4.947	3.661
TR2 UN A1-A	Kelbadjar	0.000	0.000	0.000	0.000	0.000	0.000	0.000	0.000	1.000	0.000	0.000	0.000	0.000	0.000	-8.717	-8.900	1.270	3.668	-1.299
TR2 UN A1-A	Gegham 1	0.000	0.000	0.000	1.000	0.000	0.000	0.000	0.000	0.000	0.000	0.000	0.000	0.000	0.000	-1.576	-12.032	2.394	-4.425	4.617
TR2 UN A1-A	Gegham 1	0.000	0.000	0.000	1.000	0.000	0.000	0.000	0.000	0.000	0.000	0.000	0.000	0.000	0.000	-0.834	-10.392	3.182	-4.118	2.412
TR2 UN A1-A	Gegham 1	0.000	0.000	0.000	1.000	0.000	0.000	0.000	0.000	0.000	0.000	0.000	0.000	0.000	0.000	0.322	-9.966	3.130	-4.766	3.477
TR2 UN A1-A	Gegham 1	0.000	0.000	0.000	1.000	0.000	0.000	0.000	0.000	0.000	0.000	0.000	0.000	0.000	0.000	0.463	-11.335	3.597	-4.678	3.812
TR2 UN A1-A	Gegham 1	0.000	0.000	0.000	1.000	0.000	0.000	0.000	0.000	0.000	0.000	0.000	0.000	0.000	0.000	0.933	-10.220	2.695	-4.819	3.731
TR2 UN A1-A	Syunik	0.000	0.000	0.000	0.000	0.000	0.000	0.000	0.000	0.000	0.000	0.000	0.000	1.000	0.000	-7.299	-13.539	-3.659	5.157	-1.801
TR2 UN A1-A	Gegham 1	0.000	0.000	0.000	1.000	0.000	0.000	0.000	0.000	0.000	0.000	0.000	0.000	0.000	0.000	-0.794	-10.601	3.531	-4.368	1.327
TR2 UN A1-A	Kelbadjar	0.000	0.000	0.000	0.000	0.000	0.000	0.000	0.000	1.000	0.000	0.000	0.000	0.000	0.000	-9.953	-10.132	-0.398	3.945	-1.622
TR2 UN A1-A	Gegham 1	0.000	0.000	0.000	1.000	0.000	0.000	0.000	0.000	0.000	0.000	0.000	0.000	0.000	0.000	-0.286	-9.000	3.727	-4.099	4.063
TR2 UN A1-A	Gegham 1	0.000	0.000	0.000	1.000	0.000	0.000	0.000	0.000	0.000	0.000	0.000	0.000	0.000	0.000	-1.842	-10.493	2.759	-4.214	4.936
TR2 UN A1-A	Arteni	0.000	0.989	0.000	0.000	0.011	0.000	0.000	0.000	0.000	0.000	0.000	0.000	0.000	0.000	-2.149	3.526	5.409	-2.295	-5.616
TR2 UN A1-A	Kelbadjar	0.000	0.000	0.000	0.000	0.000	0.000	0.000	0.000	1.000	0.000	0.000	0.000	0.000	0.000	-9.550	-9.411	0.065	4.816	-2.158
TR2 UN A1-A	Gegham 1	0.000	0.000	0.000	1.000	0.000	0.000	0.000	0.000	0.000	0.000	0.000	0.000	0.000	0.000	1.548	-11.672	2.488	-5.140	8.403
TR2 UN A1-A	Gegham 1	0.000	0.000	0.000	1.000	0.000	0.000	0.000	0.000	0.000	0.000	0.000	0.000	0.000	0.000	0.390	-9.488	3.907	-4.367	3.778
TR2 UN A1-A	Kelbadjar	0.000	0.000	0.000	0.000	0.000	0.000	0.000	0.000	1.000	0.000	0.000	0.000	0.000	0.000	-10.965	-8.926	0.006	6.053	-2.287
TR2 UN A1-A	Gegham 1	0.000	0.000	0.000	1.000	0.000	0.000	0.000	0.000	0.000	0.000	0.000	0.000	0.000	0.000	0.891	-8.549	4.476	-4.914	3.486
TR2 UN A1-A	Gegham 1	0.000	0.000	0.000	1.000	0.000	0.000	0.000	0.000	0.000	0.000	0.000	0.000	0.000	0.000	-1.011	-10.498	3.238	-4.504	4.293
TR2 UN A1-A	Kelbadjar	0.000	0.000	0.000	0.000	0.000	0.000	0.000	0.000	1.000	0.000	0.000	0.000	0.000	0.000	-9.999	-9.793	-1.229	4.804	-1.208
TR2 UN A1-A	Kelbadjar	0.000	0.000	0.000	0.000	0.000	0.000	0.000	0.000	0.997	0.000	0.000	0.000	0.003	0.000	-9.980	-10.758	-1.721	5.235	-1.142

TR2 UN A1-A	Kelbadjar	0.000	0.000	0.000	0.000	0.000	0.000	0.000	0.000	1.000	0.000	0.000	0.000	0.000	0.000	-9.549	-9.076	0.691	3.306	-1.583
TR2 UN A1-A	Gegham 1	0.000	0.000	0.000	1.000	0.000	0.000	0.000	0.000	0.000	0.000	0.000	0.000	0.000	0.000	-0.553	-10.461	4.241	-4.951	5.843
TR2 UN A1-A	Kelbadjar	0.000	0.000	0.000	0.000	0.000	0.000	0.000	0.000	0.986	0.000	0.000	0.000	0.014	0.000	-10.449	-10.296	-1.100	4.642	-1.479
TR2 UN A1-A	Gegham 1	0.000	0.000	0.000	1.000	0.000	0.000	0.000	0.000	0.000	0.000	0.000	0.000	0.000	0.000	-0.114	-10.001	4.048	-4.354	3.323
TR2 UN A1-A	Gegham 1	0.000	0.000	0.000	1.000	0.000	0.000	0.000	0.000	0.000	0.000	0.000	0.000	0.000	0.000	-1.466	-9.042	3.621	-4.746	7.038
TR2 UN A1-A	Gegham 1	0.000	0.000	0.000	1.000	0.000	0.000	0.000	0.000	0.000	0.000	0.000	0.000	0.000	0.000	-1.593	-12.425	2.575	-3.755	3.807
TR2 UN A1-A	Gegham 1	0.000	0.000	0.000	1.000	0.000	0.000	0.000	0.000	0.000	0.000	0.000	0.000	0.000	0.000	2.449	-9.502	2.673	-4.670	4.362
TR2 UN A1-A	Gegham 1	0.000	0.000	0.000	1.000	0.000	0.000	0.000	0.000	0.000	0.000	0.000	0.000	0.000	0.000	-1.549	-11.286	3.015	-3.973	2.727
TR2 UN A1-A	Kelbadjar	0.000	0.000	0.000	0.000	0.000	0.000	0.000	0.000	1.000	0.000	0.000	0.000	0.000	0.000	-10.639	-9.250	-0.858	4.421	-1.691
TR2 UN A1-A	Gegham 1	0.000	0.000	0.000	1.000	0.000	0.000	0.000	0.000	0.000	0.000	0.000	0.000	0.000	0.000	-0.183	-10.964	4.665	-4.732	5.549
TR.2 A5 H5 S	Gegham 1	0.000	0.000	0.000	1.000	0.000	0.000	0.000	0.000	0.000	0.000	0.000	0.000	0.000	0.000	-1.083	-12.525	3.074	-4.516	3.254
TR.2 A5 H5 S	Gegham 1	0.000	0.000	0.000	1.000	0.000	0.000	0.000	0.000	0.000	0.000	0.000	0.000	0.000	0.000	-2.345	-11.964	3.291	-4.239	3.270
TR.2 A5 H5 S	Gegham 1	0.000	0.000	0.000	1.000	0.000	0.000	0.000	0.000	0.000	0.000	0.000	0.000	0.000	0.000	-2.623	-10.639	1.820	-4.069	3.046
TR.2 A5 H5 S	Gegham 1	0.000	0.000	0.000	1.000	0.000	0.000	0.000	0.000	0.000	0.000	0.000	0.000	0.000	0.000	-0.999	-10.857	2.963	-4.152	3.392
TR.2 A5 H5 S	Gegham 1	0.000	0.000	0.000	1.000	0.000	0.000	0.000	0.000	0.000	0.000	0.000	0.000	0.000	0.000	-2.335	-12.242	2.310	-3.670	1.839
TR.2 A5 H5 S	Gegham 1	0.000	0.000	0.000	1.000	0.000	0.000	0.000	0.000	0.000	0.000	0.000	0.000	0.000	0.000	-1.665	-11.372	3.294	-4.263	2.252
TR.2 A5 H5 S	Gegham 1	0.000	0.000	0.000	1.000	0.000	0.000	0.000	0.000	0.000	0.000	0.000	0.000	0.000	0.000	-1.038	-10.718	3.135	-4.660	3.029
TR.2 A5 H5 S	Gegham 1	0.000	0.000	0.000	1.000	0.000	0.000	0.000	0.000	0.000	0.000	0.000	0.000	0.000	0.000	-0.883	-11.163	2.053	-4.318	3.281
TR.2 A5 H5 S	Gegham 1	0.000	0.000	0.000	1.000	0.000	0.000	0.000	0.000	0.000	0.000	0.000	0.000	0.000	0.000	-1.000	-10.879	3.176	-4.479	2.468
TR.2 A5 H5 S	Syunik	0.000	0.000	0.000	0.000	0.000	0.000	0.000	0.000	0.000	0.000	0.000	0.000	1.000	0.000	-7.181	-12.581	-2.037	3.669	-1.097
TR.2 A5 H5 S	Gegham 1	0.000	0.000	0.000	1.000	0.000	0.000	0.000	0.000	0.000	0.000	0.000	0.000	0.000	0.000	3.494	-10.397	2.118	-4.176	3.715
TR.2 A5 H5 S	Syunik	0.000	0.000	0.000	0.000	0.000	0.000	0.000	0.000	0.000	0.000	0.000	0.000	1.000	0.000	-8.569	-17.295	-2.458	2.309	-0.373
TR.2 A5 H5 S	Gegham 1	0.000	0.000	0.000	1.000	0.000	0.000	0.000	0.000	0.000	0.000	0.000	0.000	0.000	0.000	-0.685	-10.346	3.622	-4.059	3.015
TR.2 A5 H5 S	Gegham 1	0.000	0.000	0.000	1.000	0.000	0.000	0.000	0.000	0.000	0.000	0.000	0.000	0.000	0.000	-0.272	-11.517	3.198	-4.592	4.377
TR.2 A5 H5 S	Gegham 1	0.000	0.000	0.000	1.000	0.000	0.000	0.000	0.000	0.000	0.000	0.000	0.000	0.000	0.000	0.215	-10.124	3.737	-4.928	3.604
TR.2 A5 H5 S	Gegham 1	0.000	0.000	0.000	1.000	0.000	0.000	0.000	0.000	0.000	0.000	0.000	0.000	0.000	0.000	-0.977	-11.521	2.835	-4.581	2.606
TR.2 A5 H5 S	Gegham 1	0.000	0.000	0.000	1.000	0.000	0.000	0.000	0.000	0.000	0.000	0.000	0.000	0.000	0.000	1.286	-9.267	2.414	-4.601	4.477
TR.2 A5 H5 S	Gegham 1	0.000	0.000	0.000	1.000	0.000	0.000	0.000	0.000	0.000	0.000	0.000	0.000	0.000	0.000	-1.119	-12.291	2.286	-4.599	3.953
TR.2 A5 H5 S	Gegham 1	0.000	0.000	0.000	1.000	0.000	0.000	0.000	0.000	0.000	0.000	0.000	0.000	0.000	0.000	-1.674	-11.818	2.888	-4.344	4.045
TR.2 A5 H5 S	Gegham 1	0.000	0.000	0.000	1.000	0.000	0.000	0.000	0.000	0.000	0.000	0.000	0.000	0.000	0.000	-0.186	-10.460	4.070	-5.043	4.136
TR.2 A5 H5 S	Gegham 1	0.000	0.000	0.000	1.000	0.000	0.000	0.000	0.000	0.000	0.000	0.000	0.000	0.000	0.000	3.026	-11.044	2.018	-4.489	3.884
TR.2 A5 H5 S	Gegham 1	0.000	0.000	0.000	1.000	0.000	0.000	0.000	0.000	0.000	0.000	0.000	0.000	0.000	0.000	-0.360	-9.935	3.179	-4.383	3.653
TR.2 A5 H5 S	Gegham 1	0.000	0.000	0.000	1.000	0.000	0.000	0.000	0.000	0.000	0.000	0.000	0.000	0.000	0.000	0.072	-10.512	4.044	-4.846	3.303
TR.2 A5 H5 S	Gegham 1	0.000	0.000	0.000	1.000	0.000	0.000	0.000	0.000	0.000	0.000	0.000	0.000	0.000	0.000	-1.100	-10.723	3.048	-4.492	5.443
TR.2 A5 H5 S	Gegham 1	0.000	0.000	0.000	1.000	0.000	0.000	0.000	0.000	0.000	0.000	0.000	0.000	0.000	0.000	0.704	-9.282	3.973	-4.574	4.078
TR.2 A5 H5 S	Syunik	0.000	0.000	0.000	0.000	0.000	0.000	0.000	0.000	0.000	0.000	0.000	0.000	1.000	0.000	-8.085	-14.600	-2.421	1.701	0.290
TR.2 A5 H5 S	Syunik	0.000	0.000	0.000	0.000	0.000	0.000	0.000	0.000	0.000	0.000	0.000	0.000	1.000	0.000	-8.597	-16.728	-5.242	3.138	0.149
TR.2 A5 H5 S	Gegham 1	0.000	0.000	0.000	1.000	0.000	0.000	0.000	0.000	0.000	0.000	0.000	0.000	0.000	0.000	0.167	-9.743	3.703	-4.407	4.687
TR.2 A5 H5 S	Kelbadjar	0.000	0.000	0.000	0.000	0.000	0.000	0.000	0.000	1.000	0.000	0.000	0.000	0.000	0.000	-9.865	-8.702	0.298	5.260	-2.458
TR.2 A5 H5 S	Gegham 1	0.000	0.000	0.000	1.000	0.000	0.000	0.000	0.000	0.000	0.000	0.000	0.000	0.000	0.000	0.028	-9.857	4.731	-4.641	5.045
TR.2 Unit A0	Syunik	0.000	0.000	0.000	0.000	0.000	0.000	0.000	0.000	0.000	0.000	0.000	0.000	1.000	0.000	-6.394	-11.821	-1.254	3.264	-2.003
TR.2 Unit A0	Arteni	0.000	1.000	0.000	0.000	0.000	0.000	0.000	0.000	0.000	0.000	0.000	0.000	0.000	0.000	-1.887	3.483	3.050	-4.725	-7.896
TR.2 Unit A0	Syunik	0.000	0.000	0.000	0.000	0.000	0.000	0.000	0.000	0.000	0.000	0.000	0.000	1.000	0.000	-7.102	-13.079	-1.773	4.661	-2.055
TR.2 Unit A0	Kelbadjar	0.000	0.000	0.000	0.000	0.000	0.000	0.000	0.000	1.000	0.000	0.000	0.000	0.000	0.000	-9.361	-8.631	0.098	3.474	-1.281
TR.2 Unit A0	Gegham 1	0.000	0.000	0.000	1.000	0.000	0.000	0.000	0.000	0.000	0.000	0.000	0.000	0.000	0.000	-0.787	-11.037	2.878	-4.618	3.797
TR.2 Unit A0	Gegham 1	0.000	0.000	0.000	1.000	0.000	0.000	0.000	0.000	0.000	0.000	0.000	0.000	0.000	0.000	0.261	-11.183	2.964	-4.550	4.687
TR.2 Unit A0	Syunik	0.000	0.000	0.000	0.000	0.000	0.000	0.000	0.000	0.000	0.000	0.000	0.000	1.000	0.000	-7.989	-15.892	-3.686	5.609	-0.678
TR.2 Unit A0	Gegham 1	0.000	0.000	0.000	1.000	0.000	0.000	0.000	0.000	0.000	0.000	0.000	0.000	0.000	0.000	-0.553	-11.760	2.390	-4.543	4.088
TR.2 Unit A0	Gegham 1	0.000	0.000	0.000	1.000	0.000	0.000	0.000	0.000	0.000	0.000	0.000	0.000	0.000	0.000	0.334	-11.302	2.732	-4.462	4.282
TR.2 Unit A0	Kelbadjar	0.000	0.000	0.000	0.000	0.000	0.000	0.000	0.000	1.000	0.000	0.000	0.000	0.000	0.000	-9.786	-9.456	0.144	5.065	-2.594
TR.2 Unit A0	Gegham 1	0.000	0.000	0.000	1.000	0.000	0.000	0.000	0.000	0.000	0.000	0.000	0.000	0.000	0.000	-0.200	-11.157	3.407	-4.975	4.268
TR.2 Unit A0	Gegham 1	0.000	0.000	0.000	1.000	0.000	0.000	0.000	0.000	0.000	0.000	0.000	0.000	0.000	0.000	-0.329	-9.415	3.491	-4.631	4.597
TR.2 Unit A0	Syunik	0.000	0.000	0.000	0.000	0.000	0.000	0.000	0.000	0.000	0.000	0.000	0.000	1.000	0.000	-7.266	-10.646	-1.652	4.174	-1.191
Tr-2 B0 H1 S	Gegham 1	0.000	0.000	0.000	1.000	0.000	0.000	0.000	0.000	0.000	0.000	0.000	0.000	0.000	0.000	-2.205	-12.504	2.739	-4.266	3.162
Tr-2 B0 H1 S	Kelbadjar	0.000	0.000	0.000	0.000	0.000	0.000	0.000	0.000	1.000	0.000	0.000	0.000	0.000	0.000	-9.614	-10.065	0.524	4.392	-1.178
Tr-2 B0 H1 S	Gegham 1	0.000	0.000	0.000	1.000	0.000	0.000	0.000	0.000	0.000	0.000	0.000	0.000	0.000	0.000	1.924	-10.680	2.709	-4.454	3.350
Tr-2 B0 H1 S	Gegham 1	0.000	0.000	0.000	1.000	0.000	0.000	0.000	0.000	0.000	0.000	0.000	0.000	0.000	0.000	2.324	-11.762	2.196	-4.346	5.257
Tr-2 B0 H1 S	Kelbadjar	0.000	0.000	0.000	0.000	0.000	0.000	0.000	0.000	1.000	0.000	0.000	0.000	0.000	0.000	-9.811	-9.801	-0.263	4.842	-1.215
Tr-2 B0 H1 S	Syunik	0.000	0.000	0.000	0.000	0.000	0.000	0.000	0.000	0.000	0.000	0.000	0.000	1.000	0.000	-7.577	-14.246	-2.894	4.390	-1.352
Tr-2 B0 H1 S	Gegham 1	0.000	0.000	0.000	1.000	0.000	0.000	0.000	0.000	0.000	0.000	0.000	0.000	0.000	0.000	-0.245	-11.684	2.721	-4.558	3.796
Tr-2 B0 H1 S	Gegham 1	0.000	0.000	0.000	1.000	0.000	0.000	0.000	0.000	0.000	0.000	0.000	0.000	0.000	0.000	-0.372	-9.594	4.695	-5.100	5.477
Tr-2 B0 H1 S	Gegham 1	0.000	0.000	0.000	1.000	0.000	0.000	0.000	0.000	0.000	0.000	0.000	0.000	0.000	0.000	-0.473	-11.010	3.526	-4.396	3.322
Tr-2 B0 H1 S	Kelbadjar	0.000	0.000	0.000	0.000	0.000	0.000	0.000	0.000	1.000	0.000	0.000	0.000	0.000	0.000	-11.588	-7.291	-3.489	11.025	-3.301
Tr-2 B0 H1 S	Gegham 1	0.000	0.000	0.000	1.000	0.000	0.000	0.000	0.000	0.000	0.000	0.000	0.000	0.000	0.000	-0.719	-10.731	2.611	-4.508	3.840
Tr-2 B0 H1 S	Kelbadjar	0.000	0.000	0.000	0.000	0.000	0.000	0.000	0.000	1.000	0.000	0.000	0.000	0.000	0.000	-9.405	-11.085	0.427	4.833	-1.710
Tr-2 B0 H1 S	Syunik	0.000	0.000	0.000	0.000	0.000	0.000	0.000	0.000	0.000	0.000	0.000	0.000	1.000	0.000	-3.650	-10.604	-2.387	4.248	-1.220
Tr-2 B0 H1 S	Gegham 1	0.000	0.000	0.000	1.000	0.000	0.000	0.000	0.000	0.000	0.000	0.000	0.000	0.000	0.000	0.809	-10.236	3.610	-5.106	4.670

Tr-2 B0 H1 S	Gegham 1	0.000	0.000	0.000	1.000	0.000	0.000	0.000	0.000	0.000	0.000	0.000	0.000	0.000	0.000	-0.262	-8.704	5.421	-5.078	6.199
Tr-2 B0 H1 S	Gegham 1	0.000	0.000	0.000	1.000	0.000	0.000	0.000	0.000	0.000	0.000	0.000	0.000	0.000	0.000	-0.018	-9.443	3.841	-4.882	6.546
Tr-2 B0 H1 S	Gegham 1	0.000	0.000	0.000	1.000	0.000	0.000	0.000	0.000	0.000	0.000	0.000	0.000	0.000	0.000	-0.103	-10.050	3.295	-4.771	5.100
TR.2 C0 H1 S	Gegham 1	0.000	0.000	0.000	1.000	0.000	0.000	0.000	0.000	0.000	0.000	0.000	0.000	0.000	0.000	-0.569	-11.558	3.091	-4.463	5.030
TR.2 C0 H1 S	Gegham 1	0.000	0.000	0.000	1.000	0.000	0.000	0.000	0.000	0.000	0.000	0.000	0.000	0.000	0.000	-1.915	-12.338	2.953	-4.322	3.889
TR.2 C0 H1 S	Syunik	0.000	0.000	0.000	0.000	0.000	0.000	0.000	0.000	0.000	0.000	0.000	0.000	1.000	0.000	-7.340	-12.153	-0.792	3.777	-1.493
TR.2 C0 H1 S	Syunik	0.000	0.000	0.000	0.000	0.000	0.000	0.000	0.000	0.000	0.000	0.000	0.000	1.000	0.000	-9.972	-10.453	-2.450	7.418	-2.526
TR.2 C0 H1 S	Syunik	0.000	0.000	0.000	0.000	0.000	0.000	0.000	0.000	0.000	0.000	0.000	0.000	1.000	0.000	-5.786	-12.251	-2.027	4.621	-1.839
TR.2 C1 H1 S	Gegham 1	0.000	0.000	0.000	1.000	0.000	0.000	0.000	0.000	0.000	0.000	0.000	0.000	0.000	0.000	-0.945	-11.267	3.382	-4.754	2.561
TR.2 C1 H1 S	Gegham 1	0.000	0.000	0.000	1.000	0.000	0.000	0.000	0.000	0.000	0.000	0.000	0.000	0.000	0.000	-1.372	-13.103	2.552	-4.776	4.320
TR.2 C1 H1 S	Gegham 1	0.000	0.000	0.000	1.000	0.000	0.000	0.000	0.000	0.000	0.000	0.000	0.000	0.000	0.000	-1.126	-9.716	3.963	-4.148	2.531
TR.2 C1 H1 S	Syunik	0.000	0.000	0.000	0.000	0.000	0.000	0.000	0.000	0.000	0.000	0.000	0.000	1.000	0.000	-6.957	-13.093	-2.305	5.011	-1.446
TR.2 C1 H1 S	Gegham 1	0.000	0.000	0.000	1.000	0.000	0.000	0.000	0.000	0.000	0.000	0.000	0.000	0.000	0.000	-1.727	-9.233	3.658	-4.257	3.066
TR.2 C1 H1 S	Kelbadjar	0.000	0.000	0.000	0.000	0.000	0.000	0.000	0.000	1.000	0.000	0.000	0.000	0.000	0.000	-9.585	-8.979	0.061	4.334	-1.800
TR2 Unit-C0	Syunik	0.000	0.000	0.000	0.000	0.000	0.000	0.000	0.000	0.000	0.000	0.000	0.000	1.000	0.000	-5.577	-11.647	-0.653	3.623	-1.775
TR2 Unit-C0	Tsaghkunyat	0.000	0.000	0.000	0.000	0.000	0.000	0.000	0.000	0.000	0.000	0.000	0.000	0.000	1.000	35.941	1.246	-6.504	1.535	-1.227
TR2 Unit-C0	Gegham 1	0.000	0.000	0.000	1.000	0.000	0.000	0.000	0.000	0.000	0.000	0.000	0.000	0.000	0.000	-1.226	-10.726	3.470	-4.876	4.951
TR2 Unit-C0	Gegham 1	0.000	0.000	0.000	1.000	0.000	0.000	0.000	0.000	0.000	0.000	0.000	0.000	0.000	0.000	-0.184	-9.280	3.182	-4.476	3.774
TR2 Unit-C0	Gegham 1	0.000	0.000	0.000	1.000	0.000	0.000	0.000	0.000	0.000	0.000	0.000	0.000	0.000	0.000	-1.401	-11.090	3.446	-4.655	5.252
TR2 Unit-C0	Syunik	0.000	0.000	0.000	0.000	0.000	0.000	0.000	0.000	0.000	0.000	0.000	0.000	1.000	0.000	-6.300	-11.395	-2.071	3.839	-1.210
TR2 Unit-C0	Gegham 1	0.000	0.000	0.000	1.000	0.000	0.000	0.000	0.000	0.000	0.000	0.000	0.000	0.000	0.000	-0.868	-11.349	2.945	-4.477	3.778
TR2 Unit-C0	Gegham 1	0.000	0.000	0.000	1.000	0.000	0.000	0.000	0.000	0.000	0.000	0.000	0.000	0.000	0.000	1.112	-10.019	3.462	-4.228	2.127
TR2 Unit-C0	Gegham 1	0.000	0.000	0.000	1.000	0.000	0.000	0.000	0.000	0.000	0.000	0.000	0.000	0.000	0.000	-0.122	-10.692	4.192	-5.314	8.158
TR2 Unit-C0	Kelbadjar	0.000	0.000	0.000	0.000	0.000	0.000	0.000	0.000	1.000	0.000	0.000	0.000	0.000	0.000	-8.633	-9.364	-1.553	5.865	-2.200
TR2 Unit-C0	Syunik	0.000	0.000	0.000	0.000	0.000	0.000	0.000	0.000	0.000	0.000	0.000	0.000	1.000	0.000	-6.417	-13.604	-3.603	5.418	-1.339
TR2 Unit-C0	Gegham 1	0.000	0.000	0.000	1.000	0.000	0.000	0.000	0.000	0.000	0.000	0.000	0.000	0.000	0.000	-1.980	-12.130	2.920	-4.478	6.328
TR.2 A1 H2 S	Kelbadjar	0.000	0.000	0.000	0.000	0.000	0.000	0.000	0.000	1.000	0.000	0.000	0.000	0.000	0.000	-8.215	-9.545	1.188	3.708	-1.449
TR.2 A1 H2 S	Gegham 1	0.000	0.000	0.000	1.000	0.000	0.000	0.000	0.000	0.000	0.000	0.000	0.000	0.000	0.000	-0.860	-11.864	3.440	-4.255	2.888
TR.2 A1 H2 S	Kelbadjar	0.000	0.000	0.000	0.000	0.000	0.000	0.000	0.000	1.000	0.000	0.000	0.000	0.000	0.000	-8.895	-9.839	0.685	3.532	-1.495
TR.2 A1 H2 S	Kelbadjar	0.000	0.000	0.000	0.000	0.000	0.000	0.000	0.000	1.000	0.000	0.000	0.000	0.000	0.000	-8.524	-9.562	0.855	3.477	-1.815
TR.2 A1 H2 S	Kelbadjar	0.000	0.000	0.000	0.000	0.000	0.000	0.000	0.000	1.000	0.000	0.000	0.000	0.000	0.000	-9.050	-10.236	0.972	4.077	-1.906
TR.2 A1 H2 S	Kelbadjar	0.000	0.000	0.000	0.000	0.000	0.000	0.000	0.000	1.000	0.000	0.000	0.000	0.000	0.000	-9.056	-9.862	1.173	3.729	-1.619
TR.2 A1 H2 S	Arteni	0.000	1.000	0.000	0.000	0.000	0.000	0.000	0.000	0.000	0.000	0.000	0.000	0.000	0.000	-1.084	3.508	3.552	-3.077	-5.129
TR.2 A1 H2 S	Kelbadjar	0.000	0.000	0.000	0.000	0.000	0.000	0.000	0.000	1.000	0.000	0.000	0.000	0.000	0.000	-9.750	-9.677	0.109	4.079	-1.415
TR.2 A1 H2 S	Kelbadjar	0.000	0.000	0.000	0.000	0.000	0.000	0.000	0.000	1.000	0.000	0.000	0.000	0.000	0.000	-9.105	-9.517	1.636	3.598	-1.908
TR.2 A1 H2 S	Arteni	0.000	1.000	0.000	0.000	0.000	0.000	0.000	0.000	0.000	0.000	0.000	0.000	0.000	0.000	-4.009	1.488	4.691	-3.353	-6.014
TR.2 A1 H2 S	Kelbadjar	0.000	0.000	0.000	0.000	0.000	0.000	0.000	0.000	1.000	0.000	0.000	0.000	0.000	0.000	-10.236	-9.906	-0.772	5.688	-1.675
TR.2 A1 H2 S	Kelbadjar	0.000	0.000	0.000	0.000	0.000	0.000	0.000	0.000	1.000	0.000	0.000	0.000	0.000	0.000	-9.868	-9.128	0.993	5.568	-1.431
TR.2 A1 H2 S	Gegham 1	0.000	0.000	0.000	1.000	0.000	0.000	0.000	0.000	0.000	0.000	0.000	0.000	0.000	0.000	-0.751	-12.109	3.111	-4.598	2.665
TR.2 A1 H2 S	Kelbadjar	0.000	0.000	0.000	0.000	0.000	0.000	0.000	0.000	1.000	0.000	0.000	0.000	0.000	0.000	-8.705	-8.666	0.753	3.623	-1.450
TR.2 A1 H2 S	Arteni	0.000	1.000	0.000	0.000	0.000	0.000	0.000	0.000	0.000	0.000	0.000	0.000	0.000	0.000	-1.866	2.240	4.700	-3.568	-7.051
TR.2 A1 H2 S	Kelbadjar	0.000	0.000	0.000	0.000	0.000	0.000	0.000	0.000	1.000	0.000	0.000	0.000	0.000	0.000	-9.548	-10.132	1.024	4.079	-1.427
TR.2 A1 H2 S	Syunik	0.000	0.000	0.000	0.000	0.000	0.000	0.000	0.000	0.000	0.000	0.000	0.000	1.000	0.000	-10.834	-9.764	-2.743	6.478	-2.064
TR.2 A1 H2 S	Arteni	0.000	1.000	0.000	0.000	0.000	0.000	0.000	0.000	0.000	0.000	0.000	0.000	0.000	0.000	-0.869	3.321	3.047	-3.557	-5.967
TR.2 A1 H2 S	Kelbadjar	0.000	0.000	0.000	0.000	0.000	0.000	0.000	0.000	1.000	0.000	0.000	0.000	0.000	0.000	-9.061	-10.128	-0.599	4.180	-1.083
TR.2 A1 H2 S	Syunik	0.000	0.000	0.000	0.000	0.000	0.000	0.000	0.000	0.253	0.000	0.000	0.000	0.747	0.000	-11.082	-9.833	-1.879	5.879	-1.821
TR.2 A1 H2 S	Kelbadjar	0.000	0.000	0.000	0.000	0.000	0.000	0.000	0.000	1.000	0.000	0.000	0.000	0.000	0.000	-10.023	-8.368	-0.849	3.940	-1.822
TR.2 A1 H2 S	Tsaghkunyat	0.000	0.000	0.000	0.000	0.000	0.000	0.000	0.000	0.000	0.000	0.000	0.000	0.000	1.000	48.606	3.080	-2.013	9.814	-1.534
Tr2 A2-A3 H2	Gegham 1	0.000	0.000	0.000	1.000	0.000	0.000	0.000	0.000	0.000	0.000	0.000	0.000	0.000	0.000	2.053	-11.706	1.923	-4.433	4.270
Tr2 A2-A3 H2	Syunik	0.000	0.000	0.000	0.000	0.000	0.000	0.000	0.000	0.002	0.000	0.000	0.000	0.998	0.000	-5.795	-11.570	-0.968	3.343	-1.199
Tr2 A2-A3 H2	Gegham 1	0.000	0.000	0.000	1.000	0.000	0.000	0.000	0.000	0.000	0.000	0.000	0.000	0.000	0.000	0.408	-12.056	3.562	-4.979	3.493
Tr2 A2-A3 H2	Gegham 1	0.000	0.000	0.000	1.000	0.000	0.000	0.000	0.000	0.000	0.000	0.000	0.000	0.000	0.000	-0.399	-10.126	3.409	-4.821	3.403
Tr2 A2-A3 H2	Gegham 1	0.000	0.000	0.000	1.000	0.000	0.000	0.000	0.000	0.000	0.000	0.000	0.000	0.000	0.000	-1.089	-9.809	3.215	-4.837	5.282
Tr2 A2-A3 H2	Gegham 1	0.000	0.000	0.000	1.000	0.000	0.000	0.000	0.000	0.000	0.000	0.000	0.000	0.000	0.000	-0.226	-9.891	4.468	-4.755	2.484
Tr2 A2-A3 H2	Gegham 1	0.000	0.000	0.000	1.000	0.000	0.000	0.000	0.000	0.000	0.000	0.000	0.000	0.000	0.000	1.057	-10.556	2.519	-4.344	2.896
Tr2 A2-A3 H2	Kelbadjar	0.000	0.000	0.000	0.000	0.000	0.000	0.000	0.000	1.000	0.000	0.000	0.000	0.000	0.000	-8.071	-9.218	2.227	2.905	-1.570
Tr2 A2-A3 H2	Gegham 1	0.000	0.000	0.000	1.000	0.000	0.000	0.000	0.000	0.000	0.000	0.000	0.000	0.000	0.000	0.524	-10.449	1.828	-3.828	3.541
Tr2 A2-A3 H2	Gegham 1	0.000	0.000	0.000	1.000	0.000	0.000	0.000	0.000	0.000	0.000	0.000	0.000	0.000	0.000	-0.811	-10.187	1.948	-4.299	2.719
Tr2 A2-A3 H2	Gegham 1	0.000	0.000	0.000	1.000	0.000	0.000	0.000	0.000	0.000	0.000	0.000	0.000	0.000	0.000	-0.733	-10.497	3.560	-4.251	2.532
Tr2 A2-A3 H2	Kelbadjar	0.000	0.000	0.000	0.000	0.000	0.000	0.000	0.000	1.000	0.000	0.000	0.000	0.000	0.000	-9.729	-8.991	2.154	4.410	-2.280
Tr2 A2-A3 H2	Gegham 1	0.000	0.000	0.000	1.000	0.000	0.000	0.000	0.000	0.000	0.000	0.000	0.000	0.000	0.000	-0.944	-10.847	2.882	-4.125	2.509
Tr2 A2-A3 H2	Gegham 1	0.000	0.000	0.000	1.000	0.000	0.000	0.000	0.000	0.000	0.000	0.000	0.000	0.000	0.000	-1.053	-12.098	3.061	-4.180	3.567
Tr2 A2-A3 H2	Kelbadjar	0.000	0.000	0.000	0.000	0.000	0.000	0.000	0.000	1.000	0.000	0.000	0.000	0.000	0.000	-10.177	-10.803	0.181	5.753	-1.762
Tr2 A2-A3 H2	Gegham 1	0.000	0.000	0.000	1.000	0.000	0.000	0.000	0.000	0.000	0.000	0.000	0.000	0.000	0.000	-0.872	-10.870	3.821	-4.783	4.179
Tr2 A2-A3 H2	Gegham 1	0.000	0.000	0.000	1.000	0.000	0.000	0.000	0.000	0.000	0.000	0.000	0.000	0.000	0.000	-1.517	-11.512	3.252	-4.540	4.830
Tr2 A2-A3 H2	Gegham 1	0.000	0.000	0.000	1.000	0.000	0.000	0.000	0.000	0.000	0.000	0.000	0.000	0.000	0.000	-0.311	-12.157	3.026	-4.613	5.070
Tr2 A2-A3 H2	Kelbadjar	0.000	0.000	0.000	0.000	0.000	0.000	0.000	0.000	0.997	0.000	0.000	0.000	0.003	0.000	-9.311	-11.848	-0.734	4.865	-1.153

Tr2 A2-A3 H2	Syunik	0.000	0.000	0.000	0.000	0.000	0.000	0.000	0.000	0.315	0.000	0.000	0.000	0.685	0.000	-6.677	-11.886	-0.366	2.593	-1.578
Tr2 A2-A3 H2	Chikiani	0.000	0.000	1.000	0.000	0.000	0.000	0.000	0.000	0.000	0.000	0.000	0.000	0.000	0.000	20.755	-4.078	-2.829	2.760	0.279
Tr2 A2-A3 H2	Gegham 1	0.000	0.000	0.000	1.000	0.000	0.000	0.000	0.000	0.000	0.000	0.000	0.000	0.000	0.000	-0.096	-9.854	3.767	-4.308	2.546
Tr2 A2-A3 H2	Kelbadjar	0.000	0.000	0.000	0.000	0.000	0.000	0.000	0.000	1.000	0.000	0.000	0.000	0.000	0.000	-9.270	-9.744	0.794	5.147	-2.066
Tr2 A2-A3 H2	Gegham 1	0.000	0.000	0.000	1.000	0.000	0.000	0.000	0.000	0.000	0.000	0.000	0.000	0.000	0.000	-0.572	-10.400	3.693	-4.552	4.474
Tr2 A2-A3 H2	Syunik	0.000	0.000	0.000	0.000	0.000	0.000	0.000	0.000	0.000	0.000	0.000	0.000	1.000	0.000	-6.249	-12.855	-2.380	5.178	-0.986
TR.2 B0 H2 S	Gegham 1	0.000	0.000	0.000	1.000	0.000	0.000	0.000	0.000	0.000	0.000	0.000	0.000	0.000	0.000	-1.154	-11.632	2.802	-4.484	2.938
TR.2 B0 H2 S	Gegham 1	0.000	0.000	0.000	1.000	0.000	0.000	0.000	0.000	0.000	0.000	0.000	0.000	0.000	0.000	0.719	-11.278	2.811	-4.650	4.141
TR.2 B0 H2 S	Syunik	0.000	0.000	0.000	0.000	0.000	0.000	0.000	0.000	0.000	0.000	0.000	0.000	1.000	0.000	-7.165	-12.930	-1.434	3.510	-1.274
TR.2 B0 H2 S	Kelbadjar	0.000	0.000	0.000	0.000	0.000	0.000	0.000	0.000	1.000	0.000	0.000	0.000	0.000	0.000	-8.178	-6.412	1.741	2.045	-1.353
TR.2 B0 H2 S	Gegham 1	0.000	0.000	0.000	1.000	0.000	0.000	0.000	0.000	0.000	0.000	0.000	0.000	0.000	0.000	-2.001	-11.934	2.685	-4.173	2.345
TR.2 B0 H2 S	Gegham 1	0.000	0.000	0.000	1.000	0.000	0.000	0.000	0.000	0.000	0.000	0.000	0.000	0.000	0.000	-1.162	-12.550	1.996	-4.077	3.063
TR.2 B0 H2 S	Gegham 1	0.000	0.000	0.000	1.000	0.000	0.000	0.000	0.000	0.000	0.000	0.000	0.000	0.000	0.000	-0.147	-10.027	2.894	-4.412	2.046
TR.2 B0 H2 S	Kelbadjar	0.000	0.000	0.000	0.000	0.000	0.000	0.000	0.000	0.998	0.000	0.000	0.000	0.002	0.000	-10.445	-10.198	-0.979	5.066	-1.512
TR.2 B0 H2 S	Gegham 1	0.000	0.000	0.000	1.000	0.000	0.000	0.000	0.000	0.000	0.000	0.000	0.000	0.000	0.000	-0.334	-11.739	2.395	-4.719	3.640
TR.2 B0 H2 S	Syunik	0.000	0.000	0.000	0.000	0.000	0.000	0.000	0.000	0.000	0.000	0.000	0.000	1.000	0.000	-5.781	-12.119	-3.331	4.264	-1.359
TR.2 B0 H2 S	Kelbadjar	0.000	0.000	0.000	0.000	0.000	0.000	0.000	0.000	1.000	0.000	0.000	0.000	0.000	0.000	-9.332	-8.705	1.366	5.014	-2.220
TR.2 B0 H2 S	Gegham 1	0.000	0.000	0.000	1.000	0.000	0.000	0.000	0.000	0.000	0.000	0.000	0.000	0.000	0.000	-0.614	-10.912	3.262	-4.099	2.172
TR.2 B0 H2 S	Gegham 1	0.000	0.000	0.000	1.000	0.000	0.000	0.000	0.000	0.000	0.000	0.000	0.000	0.000	0.000	-0.510	-11.712	2.501	-4.615	3.607
TR.2 B0 H2 S	Gegham 1	0.000	0.000	0.000	1.000	0.000	0.000	0.000	0.000	0.000	0.000	0.000	0.000	0.000	0.000	1.110	-11.212	2.737	-4.441	4.000
TR.2 B0 H2 S	Kelbadjar	0.000	0.000	0.000	0.000	0.000	0.000	0.000	0.000	1.000	0.000	0.000	0.000	0.000	0.000	-9.449	-10.776	-0.062	3.204	-1.061
TR.2 B0 H2 S	Syunik	0.000	0.000	0.000	0.000	0.000	0.000	0.000	0.000	0.000	0.000	0.000	0.000	1.000	0.000	-5.116	-12.779	-2.534	4.314	-1.276
TR.2 B0 H2 S	Kelbadjar	0.000	0.000	0.000	0.000	0.000	0.000	0.000	0.000	1.000	0.000	0.000	0.000	0.000	0.000	-9.487	-9.240	0.453	5.558	-2.252
TR.2 B0 H2 S	Gegham 1	0.000	0.000	0.000	1.000	0.000	0.000	0.000	0.000	0.000	0.000	0.000	0.000	0.000	0.000	-1.356	-12.266	3.057	-4.714	3.736
TR.2 B0 H2 S	Gegham 1	0.000	0.000	0.000	1.000	0.000	0.000	0.000	0.000	0.000	0.000	0.000	0.000	0.000	0.000	-0.370	-11.091	4.112	-4.878	3.304
TR.2 B0 H2 S	Gegham 1	0.000	0.000	0.000	1.000	0.000	0.000	0.000	0.000	0.000	0.000	0.000	0.000	0.000	0.000	-0.600	-10.125	3.175	-4.115	2.254
TR.2 B0 H2 S	Gegham 1	0.000	0.000	0.000	1.000	0.000	0.000	0.000	0.000	0.000	0.000	0.000	0.000	0.000	0.000	0.014	-11.544	2.403	-4.432	5.695
TR.2 B0 H2 S	Syunik	0.000	0.000	0.000	0.000	0.000	0.000	0.000	0.000	0.000	0.000	0.000	0.000	1.000	0.000	-11.211	-10.580	-4.024	8.780	-3.217
TR.2 B0 H2 S	Gegham 1	0.000	0.000	0.000	1.000	0.000	0.000	0.000	0.000	0.000	0.000	0.000	0.000	0.000	0.000	-0.625	-11.294	2.721	-4.344	3.777
TR.2 B0 H2 S	Arteni	0.000	1.000	0.000	0.000	0.000	0.000	0.000	0.000	0.000	0.000	0.000	0.000	0.000	0.000	-0.753	2.802	3.344	-2.979	-5.263
TR.2 B0 H2 S	Gegham 1	0.000	0.000	0.000	1.000	0.000	0.000	0.000	0.000	0.000	0.000	0.000	0.000	0.000	0.000	-0.225	-10.078	3.606	-4.500	4.883
TR.2 B0 H2 S	Syunik	0.000	0.000	0.000	0.000	0.000	0.000	0.000	0.000	0.000	0.000	0.000	0.000	1.000	0.000	-7.905	-14.198	-1.735	1.488	-0.039
TR.2 B0 H2 S	Gegham 1	0.000	0.000	0.000	1.000	0.000	0.000	0.000	0.000	0.000	0.000	0.000	0.000	0.000	0.000	-0.170	-10.898	3.418	-5.075	4.450
TR.2 B0 H2 S	Gegham 1	0.000	0.000	0.000	1.000	0.000	0.000	0.000	0.000	0.000	0.000	0.000	0.000	0.000	0.000	-0.776	-8.759	5.913	-5.466	8.198
TR.2 B0 H2 S	Gegham 1	0.000	0.000	0.000	1.000	0.000	0.000	0.000	0.000	0.000	0.000	0.000	0.000	0.000	0.000	0.605	-8.835	3.798	-4.890	4.365
TR.2 B0 H2 S	Kelbadjar	0.000	0.000	0.000	0.000	0.000	0.000	0.000	0.000	1.000	0.000	0.000	0.000	0.000	0.000	-8.085	-9.894	-0.441	3.607	-0.813
T2 B1 Hor.3 $	Gegham 1	0.000	0.000	0.000	1.000	0.000	0.000	0.000	0.000	0.000	0.000	0.000	0.000	0.000	0.000	-2.141	-10.429	2.671	-3.942	1.878
T2 B1 Hor.3 $	Gegham 1	0.000	0.000	0.000	1.000	0.000	0.000	0.000	0.000	0.000	0.000	0.000	0.000	0.000	0.000	-0.206	-10.566	3.147	-4.943	3.906
T2 B1 Hor.3 $	Syunik	0.000	0.000	0.000	0.000	0.000	0.000	0.000	0.000	0.000	0.000	0.000	0.000	1.000	0.000	-6.454	-12.455	-1.324	3.277	-0.917
T2 B1 Hor.3 $	Gegham 1	0.000	0.000	0.000	1.000	0.000	0.000	0.000	0.000	0.000	0.000	0.000	0.000	0.000	0.000	-1.503	-11.603	2.058	-4.373	4.615
T2 B1 Hor.3 $	Syunik	0.000	0.000	0.000	0.000	0.000	0.000	0.000	0.000	0.000	0.000	0.000	0.000	1.000	0.000	-6.752	-11.171	-2.026	4.405	-1.792
T2 B1 Hor.3 $	Gegham 1	0.000	0.000	0.000	1.000	0.000	0.000	0.000	0.000	0.000	0.000	0.000	0.000	0.000	0.000	0.033	-10.489	3.252	-4.926	4.024
T2 B1 Hor.3 $	Syunik	0.000	0.000	0.000	0.000	0.000	0.000	0.000	0.000	0.000	0.000	0.000	0.000	1.000	0.000	-6.717	-12.067	-2.407	4.796	-0.785
T2 B1 Hor.3 $	Arteni	0.000	1.000	0.000	0.000	0.000	0.000	0.000	0.000	0.000	0.000	0.000	0.000	0.000	0.000	-3.712	2.100	6.790	-3.162	-6.318
T2 B1 Hor.3 $	Gegham 1	0.000	0.000	0.000	1.000	0.000	0.000	0.000	0.000	0.000	0.000	0.000	0.000	0.000	0.000	0.584	-10.131	4.054	-4.722	6.528
T2 B1 Hor.3 $	Kelbadjar	0.000	0.000	0.000	0.000	0.000	0.000	0.000	0.000	1.000	0.000	0.000	0.000	0.000	0.000	-9.794	-8.978	-0.439	4.381	-2.182
T.2 C1 Hor.3	Gegham 1	0.000	0.000	0.000	1.000	0.000	0.000	0.000	0.000	0.000	0.000	0.000	0.000	0.000	0.000	-1.355	-11.809	1.709	-4.120	3.076
T.2 C1 Hor.3	Syunik	0.000	0.000	0.000	0.000	0.000	0.000	0.000	0.000	0.000	0.000	0.000	0.000	1.000	0.000	-6.776	-10.473	-1.044	4.262	-1.563
T.2 B0 Hor.3	Gegham 1	0.000	0.000	0.000	1.000	0.000	0.000	0.000	0.000	0.000	0.000	0.000	0.000	0.000	0.000	0.283	-11.506	3.323	-4.363	3.528
T.2 B0 Hor.3	Gegham 1	0.000	0.000	0.000	1.000	0.000	0.000	0.000	0.000	0.000	0.000	0.000	0.000	0.000	0.000	0.678	-11.089	3.242	-4.621	3.838
T.2 B0 Hor.3	Gegham 1	0.000	0.000	0.000	1.000	0.000	0.000	0.000	0.000	0.000	0.000	0.000	0.000	0.000	0.000	-1.891	-13.039	2.425	-4.194	5.038
T.2 B0 Hor.3	Gegham 1	0.000	0.000	0.000	1.000	0.000	0.000	0.000	0.000	0.000	0.000	0.000	0.000	0.000	0.000	-0.199	-9.702	3.481	-4.313	2.495
Tr-2 Unit-A0	Gegham 1	0.000	0.000	0.000	1.000	0.000	0.000	0.000	0.000	0.000	0.000	0.000	0.000	0.000	0.000	-1.248	-11.901	2.948	-4.321	2.297
Tr-2 Unit-A0	Gegham 1	0.000	0.000	0.000	1.000	0.000	0.000	0.000	0.000	0.000	0.000	0.000	0.000	0.000	0.000	-0.421	-11.484	3.183	-4.967	4.646
Tr-2 Unit-A0	Hatis	0.000	0.000	0.000	0.000	0.000	0.000	1.000	0.000	0.000	0.000	0.000	0.000	0.000	0.000	19.741	3.061	-1.238	-0.488	-3.419
Tr-2 Unit-A0	Gegham 1	0.000	0.000	0.000	1.000	0.000	0.000	0.000	0.000	0.000	0.000	0.000	0.000	0.000	0.000	-0.308	-13.102	1.860	-4.347	4.189
Tr-2 Unit-A0	Gegham 1	0.000	0.000	0.000	1.000	0.000	0.000	0.000	0.000	0.000	0.000	0.000	0.000	0.000	0.000	-0.904	-11.266	2.949	-4.358	4.143
Tr-2 Unit-A0	Kelbadjar	0.000	0.000	0.000	0.000	0.000	0.000	0.000	0.000	1.000	0.000	0.000	0.000	0.000	0.000	-11.255	-8.855	-1.700	6.783	-2.249
Tr-2 Unit-A0	Kelbadjar	0.000	0.000	0.000	0.000	0.000	0.000	0.000	0.000	1.000	0.000	0.000	0.000	0.000	0.000	-9.184	-9.417	-0.522	4.134	-1.793
Tr-2 Unit-A0	Gegham 1	0.000	0.000	0.000	1.000	0.000	0.000	0.000	0.000	0.000	0.000	0.000	0.000	0.000	0.000	3.576	-10.217	0.960	-3.727	4.206
Tr-2 Unit-A0	Gegham 1	0.000	0.000	0.000	1.000	0.000	0.000	0.000	0.000	0.000	0.000	0.000	0.000	0.000	0.000	-2.393	-9.245	4.917	-4.330	6.059
Tr-2 Unit-A0	Gegham 1	0.000	0.000	0.000	1.000	0.000	0.000	0.000	0.000	0.000	0.000	0.000	0.000	0.000	0.000	-1.481	-9.673	4.041	-4.686	8.064
T2 B0 Bor.3 $	Gegham 1	0.000	0.000	0.000	1.000	0.000	0.000	0.000	0.000	0.000	0.000	0.000	0.000	0.000	0.000	0.094	-11.265	3.599	-4.547	2.198
T2 B0 Bor.3 $	Kelbadjar	0.000	0.000	0.000	0.000	0.000	0.000	0.000	0.000	1.000	0.000	0.000	0.000	0.000	0.000	-9.472	-9.309	1.635	3.852	-1.884
T2 B0 Bor.3 $	Gegham 1	0.000	0.000	0.000	1.000	0.000	0.000	0.000	0.000	0.000	0.000	0.000	0.000	0.000	0.000	0.187	-9.478	3.561	-4.915	3.812
T2 B0 Bor.3 $	Gegham 1	0.000	0.000	0.000	1.000	0.000	0.000	0.000	0.000	0.000	0.000	0.000	0.000	0.000	0.000	-0.113	-10.150	3.962	-5.265	7.964
T2 B0 Hor 2 $	Gegham 1	0.000	0.000	0.000	1.000	0.000	0.000	0.000	0.000	0.000	0.000	0.000	0.000	0.000	0.000	-1.544	-11.881	3.164	-4.629	3.738

T2 B0 Hor 2 S	Gegham 1	0.000	0.000	0.000	1.000	0.000	0.000	0.000	0.000	0.000	0.000	0.000	0.000	0.000	0.000	1.224	-10.822	2.975	-5.055	3.835
T2 B0 Hor 2 S	Kelbadjar	0.000	0.000	0.000	0.000	0.000	0.000	0.000	0.000	1.000	0.000	0.000	0.000	0.000	0.000	-10.495	-9.750	-0.713	5.589	-1.970
T2 B0 Hor 2 S	Kelbadjar	0.000	0.000	0.000	0.000	0.000	0.000	0.000	0.000	1.000	0.000	0.000	0.000	0.000	0.000	-10.587	-7.775	-2.127	6.460	-2.189
T2 C0 Hor 3 S	Kelbadjar	0.000	0.000	0.000	0.000	0.000	0.000	0.000	0.000	1.000	0.000	0.000	0.000	0.000	0.000	-9.119	-9.901	-0.471	4.098	-1.287
T2 C0 Hor 3 S	Gegham 1	0.000	0.000	0.000	1.000	0.000	0.000	0.000	0.000	0.000	0.000	0.000	0.000	0.000	0.000	-0.347	-11.133	2.725	-4.219	2.329
T2 C0 Hor 3 S	Syunik	0.000	0.000	0.000	0.000	0.000	0.000	0.000	0.000	0.000	0.000	0.000	0.000	1.000	0.000	-7.351	-13.108	-3.265	5.890	-1.330
T2 C0 Hor.2 S	Kelbadjar	0.000	0.000	0.000	0.000	0.000	0.000	0.000	0.000	1.000	0.000	0.000	0.000	0.000	0.000	-8.538	-9.567	0.463	4.062	-1.331
T2 C0 Hor.2 S	Gegham 1	0.000	0.000	0.000	1.000	0.000	0.000	0.000	0.000	0.000	0.000	0.000	0.000	0.000	0.000	-0.975	-10.804	3.512	-4.663	3.350
T2 C0 Hor.2 S	Gegham 1	0.000	0.000	0.000	1.000	0.000	0.000	0.000	0.000	0.000	0.000	0.000	0.000	0.000	0.000	0.650	-10.791	3.066	-5.128	6.206
Tr-2 Unit-A0	Gegham 1	0.000	0.000	0.000	1.000	0.000	0.000	0.000	0.000	0.000	0.000	0.000	0.000	0.000	0.000	-0.316	-11.774	2.424	-5.100	4.974
Tr-2 Unit-A0	Gegham 1	0.000	0.000	0.000	1.000	0.000	0.000	0.000	0.000	0.000	0.000	0.000	0.000	0.000	0.000	3.203	-8.783	3.504	-4.946	4.032
Tr-2 Unit-A1	Arteni	0.000	1.000	0.000	0.000	0.000	0.000	0.000	0.000	0.000	0.000	0.000	0.000	0.000	0.000	-4.688	2.577	5.783	-3.801	-7.964
Tr-2 Unit-A1	Syunik	0.000	0.000	0.000	0.000	0.000	0.000	0.000	0.000	0.000	0.000	0.000	0.000	1.000	0.000	-8.151	-14.269	-3.242	3.855	-0.981
Tr-2 Unit-A1	Gegham 1	0.000	0.000	0.000	1.000	0.000	0.000	0.000	0.000	0.000	0.000	0.000	0.000	0.000	0.000	-1.083	-12.142	2.970	-4.612	3.405
Tr-2 Unit-A1	Gegham 1	0.000	0.000	0.000	1.000	0.000	0.000	0.000	0.000	0.000	0.000	0.000	0.000	0.000	0.000	-1.584	-10.930	3.661	-4.091	2.528
Tr-2 Unit-A1	Gegham 1	0.000	0.000	0.000	1.000	0.000	0.000	0.000	0.000	0.000	0.000	0.000	0.000	0.000	0.000	-0.388	-11.799	2.298	-4.645	4.202
Tr-2 Unit-A1	Gegham 1	0.000	0.000	0.000	1.000	0.000	0.000	0.000	0.000	0.000	0.000	0.000	0.000	0.000	0.000	-0.860	-10.144	3.222	-4.110	2.209
Tr-2 Unit-A1	Gegham 1	0.000	0.000	0.000	1.000	0.000	0.000	0.000	0.000	0.000	0.000	0.000	0.000	0.000	0.000	2.653	-8.731	3.291	-5.459	7.389
Tr-2 Unit-A1	Arteni	0.000	1.000	0.000	0.000	0.000	0.000	0.000	0.000	0.000	0.000	0.000	0.000	0.000	0.000	-4.825	0.751	4.711	-3.429	-5.734
Tr-2 Unit-A1	Gegham 1	0.000	0.000	0.000	1.000	0.000	0.000	0.000	0.000	0.000	0.000	0.000	0.000	0.000	0.000	-1.176	-10.934	2.981	-5.067	7.458
Tr-2 Unit-C1	Gegham 1	0.000	0.000	0.000	1.000	0.000	0.000	0.000	0.000	0.000	0.000	0.000	0.000	0.000	0.000	-1.912	-11.458	2.702	-4.353	3.262
Tr-2 Unit-C1	Kelbadjar	0.000	0.000	0.000	0.000	0.000	0.000	0.000	0.000	1.000	0.000	0.000	0.000	0.000	0.000	-9.793	-9.893	0.167	4.431	-1.270
Tr-2 Unit-C1	Gegham 1	0.000	0.000	0.000	1.000	0.000	0.000	0.000	0.000	0.000	0.000	0.000	0.000	0.000	0.000	0.203	-11.474	3.060	-4.820	2.888
Tr-2 Unit-C1	Gegham 1	0.000	0.000	0.000	1.000	0.000	0.000	0.000	0.000	0.000	0.000	0.000	0.000	0.000	0.000	-0.315	-10.340	3.416	-5.003	3.667
Tr-2 Unit-C1	Hatis	0.000	0.000	0.000	0.000	0.000	0.000	1.000	0.000	0.000	0.000	0.000	0.000	0.000	0.000	19.377	2.954	-1.626	-0.392	-3.098
Tr-2 Unit-C1	Gegham 1	0.000	0.000	0.000	1.000	0.000	0.000	0.000	0.000	0.000	0.000	0.000	0.000	0.000	0.000	-1.797	-12.564	2.457	-3.903	3.150
Tr-2 Unit-C1	Gegham 1	0.000	0.000	0.000	1.000	0.000	0.000	0.000	0.000	0.000	0.000	0.000	0.000	0.000	0.000	-0.088	-9.735	3.529	-4.596	3.357
Tr-2 Unit-C1	Gegham 1	0.000	0.000	0.000	1.000	0.000	0.000	0.000	0.000	0.000	0.000	0.000	0.000	0.000	0.000	-1.570	-9.635	3.826	-4.560	3.561
Tr-2 Unit-C1	Syunik	0.000	0.000	0.000	0.000	0.000	0.000	0.000	0.000	0.000	0.000	0.000	0.000	1.000	0.000	-7.237	-17.108	-2.343	1.909	-0.130
Tr-2 Unit-C1	Gegham 1	0.000	0.000	0.000	1.000	0.000	0.000	0.000	0.000	0.000	0.000	0.000	0.000	0.000	0.000	-2.390	-8.903	3.950	-3.425	-1.985
Tr-2 Unit-C1	Gegham 1	0.000	0.000	0.000	1.000	0.000	0.000	0.000	0.000	0.000	0.000	0.000	0.000	0.000	0.000	-1.565	-12.204	2.670	-4.659	4.868
Tr-2 Unit-C1	Gegham 1	0.000	0.000	0.000	1.000	0.000	0.000	0.000	0.000	0.000	0.000	0.000	0.000	0.000	0.000	-0.308	-10.841	3.562	-4.932	5.563
Tr-2 Unit-C1	Gegham 1	0.000	0.000	0.000	1.000	0.000	0.000	0.000	0.000	0.000	0.000	0.000	0.000	0.000	0.000	-0.421	-11.051	2.770	-4.271	4.452
Tr-2 Unit-C1	Gegham 1	0.000	0.000	0.000	1.000	0.000	0.000	0.000	0.000	0.000	0.000	0.000	0.000	0.000	0.000	-0.664	-10.748	3.298	-4.849	3.862
Tr-2 Unit-C1	Gegham 1	0.000	0.000	0.000	1.000	0.000	0.000	0.000	0.000	0.000	0.000	0.000	0.000	0.000	0.000	1.199	-9.056	4.525	-4.756	4.533
Tr-2 Unit-C1	Gegham 1	0.000	0.000	0.000	1.000	0.000	0.000	0.000	0.000	0.000	0.000	0.000	0.000	0.000	0.000	-1.493	-10.862	3.041	-4.691	4.134
Tr-2 Unit-C1	Gegham 1	0.000	0.000	0.000	1.000	0.000	0.000	0.000	0.000	0.000	0.000	0.000	0.000	0.000	0.000	0.520	-11.005	3.128	-5.289	5.904
Tr-2 Unit-C1	Gegham 1	0.000	0.000	0.000	1.000	0.000	0.000	0.000	0.000	0.000	0.000	0.000	0.000	0.000	0.000	-1.217	-11.457	3.109	-4.008	3.956
Tr-2 Unit-C1	Gegham 1	0.000	0.000	0.000	1.000	0.000	0.000	0.000	0.000	0.000	0.000	0.000	0.000	0.000	0.000	0.096	-9.099	3.493	-4.694	5.158
Tr-2 Unit-C1	Gegham 1	0.000	0.000	0.000	1.000	0.000	0.000	0.000	0.000	0.000	0.000	0.000	0.000	0.000	0.000	-0.702	-10.171	3.174	-4.908	5.532
Tr-2 Unit-C1	Arteni	0.000	0.983	0.000	0.000	0.017	0.000	0.000	0.000	0.000	0.000	0.000	0.000	0.000	0.000	-1.466	3.593	4.949	-2.034	-4.725
Tr-2 Unit-A1	Gegham 1	0.000	0.000	0.000	1.000	0.000	0.000	0.000	0.000	0.000	0.000	0.000	0.000	0.000	0.000	-1.687	-11.143	2.463	-4.334	2.298
Tr-2 Unit-A1	Gegham 1	0.000	0.000	0.000	1.000	0.000	0.000	0.000	0.000	0.000	0.000	0.000	0.000	0.000	0.000	-0.803	-12.236	2.489	-4.487	4.555
Tr-2 Unit-A1	Gegham 1	0.000	0.000	0.000	1.000	0.000	0.000	0.000	0.000	0.000	0.000	0.000	0.000	0.000	0.000	0.365	-11.835	2.633	-4.328	2.889
Tr-2 Unit-A1	Arteni	0.000	1.000	0.000	0.000	0.000	0.000	0.000	0.000	0.000	0.000	0.000	0.000	0.000	0.000	-0.478	3.105	3.514	-2.846	-5.234
Tr-2 Unit-A1	Gegham 1	0.000	0.000	0.000	1.000	0.000	0.000	0.000	0.000	0.000	0.000	0.000	0.000	0.000	0.000	-0.420	-10.980	3.221	-4.676	3.981
Tr-2 Unit-A1	Gegham 1	0.000	0.000	0.000	1.000	0.000	0.000	0.000	0.000	0.000	0.000	0.000	0.000	0.000	0.000	-0.749	-12.516	2.341	-4.437	6.628
Tr-2 Unit-A1	Arteni	0.000	0.997	0.000	0.000	0.003	0.000	0.000	0.000	0.000	0.000	0.000	0.000	0.000	0.000	1.234	2.953	4.822	-3.075	-7.975
Tr-2 Unit-A1	Gegham 1	0.000	0.000	0.000	1.000	0.000	0.000	0.000	0.000	0.000	0.000	0.000	0.000	0.000	0.000	-1.565	-10.077	2.869	-3.650	2.481
Tr-2 Unit-A1	Gegham 1	0.000	0.000	0.000	1.000	0.000	0.000	0.000	0.000	0.000	0.000	0.000	0.000	0.000	0.000	-0.763	-12.100	3.200	-4.974	5.657
Tr-2 Unit-A1	Gegham 1	0.000	0.000	0.000	1.000	0.000	0.000	0.000	0.000	0.000	0.000	0.000	0.000	0.000	0.000	-1.045	-11.534	2.317	-4.456	3.943
Tr-2 Unit-A1	Arteni	0.000	1.000	0.000	0.000	0.000	0.000	0.000	0.000	0.000	0.000	0.000	0.000	0.000	0.000	-3.675	3.486	6.188	-3.798	-5.324
Tr-2 Unit-A1	Arteni	0.000	1.000	0.000	0.000	0.000	0.000	0.000	0.000	0.000	0.000	0.000	0.000	0.000	0.000	-0.676	2.799	3.443	-3.247	-5.294
Tr-2 Unit-A1	Gegham 1	0.000	0.000	0.000	1.000	0.000	0.000	0.000	0.000	0.000	0.000	0.000	0.000	0.000	0.000	-1.163	-10.364	4.784	-5.463	9.632
Tr-2 Unit-A1	Kelbadjar	0.000	0.000	0.000	0.000	0.000	0.000	0.000	0.000	1.000	0.000	0.000	0.000	0.000	0.000	-9.684	-7.793	1.208	3.565	-1.703
TR2 B1 Hor.3	Arteni	0.000	1.000	0.000	0.000	0.000	0.000	0.000	0.000	0.000	0.000	0.000	0.000	0.000	0.000	-1.147	1.343	4.245	-2.714	-5.947
TR2 B1 Hor.3	Gegham 1	0.000	0.000	0.000	1.000	0.000	0.000	0.000	0.000	0.000	0.000	0.000	0.000	0.000	0.000	-1.024	-12.288	2.339	-4.312	3.668
TR2 B1 Hor.3	Gegham 1	0.000	0.000	0.000	1.000	0.000	0.000	0.000	0.000	0.000	0.000	0.000	0.000	0.000	0.000	-1.429	-11.675	3.217	-4.428	2.798
TR2 B1 Hor.3	Gegham 1	0.000	0.000	0.000	1.000	0.000	0.000	0.000	0.000	0.000	0.000	0.000	0.000	0.000	0.000	-0.631	-11.822	3.563	-4.756	3.669
TR2 B1 Hor.3	Gegham 1	0.000	0.000	0.000	1.000	0.000	0.000	0.000	0.000	0.000	0.000	0.000	0.000	0.000	0.000	-1.558	-10.733	2.332	-4.017	2.643
TR2 B1 Hor.3	Gegham 1	0.000	0.000	0.000	1.000	0.000	0.000	0.000	0.000	0.000	0.000	0.000	0.000	0.000	0.000	-1.248	-11.378	2.836	-4.584	3.845
TR2 B1 Hor.3	Arteni	0.000	1.000	0.000	0.000	0.000	0.000	0.000	0.000	0.000	0.000	0.000	0.000	0.000	0.000	-2.900	1.154	3.440	-3.537	-6.356
TR2 B1 Hor.3	Gegham 1	0.000	0.000	0.000	1.000	0.000	0.000	0.000	0.000	0.000	0.000	0.000	0.000	0.000	0.000	-2.094	-11.388	3.119	-3.507	1.839
TR2 B1 Hor.3	Gegham 1	0.000	0.000	0.000	1.000	0.000	0.000	0.000	0.000	0.000	0.000	0.000	0.000	0.000	0.000	-1.598	-12.038	2.393	-4.395	2.711
TR2 B1 Hor.3	Gegham 1	0.000	0.000	0.000	1.000	0.000	0.000	0.000	0.000	0.000	0.000	0.000	0.000	0.000	0.000	-0.950	-11.427	2.698	-4.595	4.481
TR2 B1 Hor.3	Gegham 1	0.000	0.000	0.000	1.000	0.000	0.000	0.000	0.000	0.000	0.000	0.000	0.000	0.000	0.000	0.064	-10.634	3.429	-4.777	5.856
TR2 B1 Hor.3	Gegham 1	0.000	0.000	0.000	1.000	0.000	0.000	0.000	0.000	0.000	0.000	0.000	0.000	0.000	0.000	-0.610	-12.546	2.940	-5.248	7.073

TR2 B1 Hor.3	Gegham 1	0.000	0.000	0.000	1.000	0.000	0.000	0.000	0.000	0.000	0.000	0.000	0.000	0.000	0.000	-1.189	-11.348	2.501	-4.381	3.611
TR2 B1 Hor.3	Gegham 1	0.000	0.000	0.000	1.000	0.000	0.000	0.000	0.000	0.000	0.000	0.000	0.000	0.000	0.000	0.557	-9.029	3.561	-5.004	6.746
TR.2 A0 H2 S	Gegham 1	0.000	0.000	0.000	1.000	0.000	0.000	0.000	0.000	0.000	0.000	0.000	0.000	0.000	0.000	-0.617	-11.311	3.599	-4.578	3.269
TR.2 A0 H2 S	Gegham 1	0.000	0.000	0.000	1.000	0.000	0.000	0.000	0.000	0.000	0.000	0.000	0.000	0.000	0.000	1.727	-11.137	2.986	-4.435	3.121
TR.2 A0 H2 S	Syunik	0.000	0.000	0.000	0.000	0.000	0.000	0.000	0.000	0.000	0.000	0.000	0.000	1.000	0.000	-6.781	-12.606	-2.616	3.183	-0.634
TR.2 A0 H2 S	Gegham 1	0.000	0.000	0.000	1.000	0.000	0.000	0.000	0.000	0.000	0.000	0.000	0.000	0.000	0.000	4.061	-9.790	1.900	-4.072	3.646
TR.2 A0 H2 S	Tsaghkunyat	0.000	0.000	0.000	0.000	0.000	0.000	0.000	0.000	0.000	0.000	0.000	0.000	0.000	1.000	36.980	1.284	-4.805	3.152	-1.922
TR.2 A0 H2 S	Gegham 1	0.000	0.000	0.000	1.000	0.000	0.000	0.000	0.000	0.000	0.000	0.000	0.000	0.000	0.000	-1.399	-11.203	2.718	-4.266	2.869
TR.2 A0 H2 S	Gegham 1	0.000	0.000	0.000	1.000	0.000	0.000	0.000	0.000	0.000	0.000	0.000	0.000	0.000	0.000	-0.439	-11.070	3.923	-4.599	1.974
TR.2 A0 H2 S	Gegham 1	0.000	0.000	0.000	1.000	0.000	0.000	0.000	0.000	0.000	0.000	0.000	0.000	0.000	0.000	0.463	-11.174	1.478	-3.442	4.263
TR.2 A0 H2 S	Syunik	0.000	0.000	0.000	0.000	0.000	0.000	0.000	0.000	0.000	0.000	0.000	0.000	1.000	0.000	-6.427	-11.782	-1.262	3.870	-1.668
TR.2 A0 H2 S	Gegham 1	0.000	0.000	0.000	1.000	0.000	0.000	0.000	0.000	0.000	0.000	0.000	0.000	0.000	0.000	4.468	-10.563	1.733	-4.364	6.315
TR.2 A0 H2 S	Kelbadjar	0.000	0.000	0.000	0.000	0.000	0.000	0.000	0.000	1.000	0.000	0.000	0.000	0.000	0.000	-9.108	-8.904	1.664	3.847	-2.150
TR.2 A0 H2 S	Kelbadjar	0.000	0.000	0.000	0.000	0.000	0.000	0.000	0.000	0.553	0.000	0.000	0.000	0.447	0.000	-9.441	-10.346	-2.045	6.383	-2.654
TR.2 A0 H2 S	Kelbadjar	0.000	0.000	0.000	0.000	0.000	0.000	0.000	0.000	1.000	0.000	0.000	0.000	0.000	0.000	-7.220	-10.331	1.693	1.579	-2.201
TR.2 A0 H2 S	Kelbadjar	0.000	0.000	0.000	0.000	0.000	0.000	0.000	0.000	1.000	0.000	0.000	0.000	0.000	0.000	-9.634	-10.341	0.366	4.749	-1.724
TR.2 A0 H2 S	Arteni	0.000	1.000	0.000	0.000	0.000	0.000	0.000	0.000	0.000	0.000	0.000	0.000	0.000	0.000	-0.234	2.227	2.602	-3.894	-6.699
TR.2 A0 H2 S	Gegham 1	0.000	0.000	0.000	1.000	0.000	0.000	0.000	0.000	0.000	0.000	0.000	0.000	0.000	0.000	0.113	-9.503	4.882	-5.076	3.769
TR.2 A0 H2 S	Kelbadjar	0.000	0.000	0.000	0.000	0.000	0.000	0.000	0.000	0.992	0.000	0.000	0.000	0.008	0.000	-10.153	-10.734	-1.163	5.591	-1.102
TR.2 A0 H2 S	Gegham 1	0.000	0.000	0.000	1.000	0.000	0.000	0.000	0.000	0.000	0.000	0.000	0.000	0.000	0.000	-0.846	-10.010	3.542	-4.557	5.403
TR.2 A0 H2 S	Gegham 1	0.000	0.000	0.000	1.000	0.000	0.000	0.000	0.000	0.000	0.000	0.000	0.000	0.000	0.000	-0.157	-9.107	5.689	-5.245	9.035
TR.2 A0 H2 S	Syunik	0.000	0.000	0.000	0.000	0.000	0.000	0.000	0.000	0.000	0.000	0.000	0.000	1.000	0.000	-5.201	-10.719	-1.554	3.274	-1.311
TR.2 A0 H2 S	Gegham 1	0.000	0.000	0.000	1.000	0.000	0.000	0.000	0.000	0.000	0.000	0.000	0.000	0.000	0.000	-1.351	-10.885	4.069	-3.705	3.472
TR.2 A0 H2 S	Gegham 1	0.000	0.000	0.000	1.000	0.000	0.000	0.000	0.000	0.000	0.000	0.000	0.000	0.000	0.000	2.371	-9.294	2.889	-4.553	4.739
TR.2 A1 H1 S	Kelbadjar	0.000	0.000	0.000	0.000	0.000	0.000	0.000	0.000	1.000	0.000	0.000	0.000	0.000	0.000	-8.082	-10.886	1.760	4.962	-0.790
TR.2 A1 H1 S	Gegham 1	0.000	0.000	0.000	1.000	0.000	0.000	0.000	0.000	0.000	0.000	0.000	0.000	0.000	0.000	-0.543	-11.575	3.877	-4.785	3.647
TR.2 A1 H1 S	Gegham 1	0.000	0.000	0.000	1.000	0.000	0.000	0.000	0.000	0.000	0.000	0.000	0.000	0.000	0.000	-2.193	-12.006	2.973	-4.499	3.752
TR.2 A1 H1 S	Kelbadjar	0.000	0.000	0.000	0.000	0.000	0.000	0.000	0.000	1.000	0.000	0.000	0.000	0.000	0.000	-9.840	-9.843	0.361	4.298	-1.394
TR.2 A1 H1 S	Kelbadjar	0.000	0.000	0.000	0.000	0.000	0.000	0.000	0.000	1.000	0.000	0.000	0.000	0.000	0.000	-8.696	-9.026	1.224	3.229	-1.545
TR.2 A1 H1 S	Kelbadjar	0.000	0.000	0.000	0.000	0.000	0.000	0.000	0.000	1.000	0.000	0.000	0.000	0.000	0.000	-9.151	-10.088	-1.005	5.495	-1.221
TR.2 A1 H1 S	Gegham 1	0.000	0.000	0.000	1.000	0.000	0.000	0.000	0.000	0.000	0.000	0.000	0.000	0.000	0.000	-1.658	-12.352	1.496	-4.413	5.439
TR.2 A1 H1 S	Kelbadjar	0.000	0.000	0.000	0.000	0.000	0.000	0.000	0.000	1.000	0.000	0.000	0.000	0.000	0.000	-9.386	-9.098	1.126	3.972	-1.980
TR.2 A1 H1 S	Kelbadjar	0.000	0.000	0.000	0.000	0.000	0.000	0.000	0.000	1.000	0.000	0.000	0.000	0.000	0.000	-9.176	-7.273	0.181	3.532	-1.665
TR.2 A1 H1 S	Gegham 1	0.000	0.000	0.000	1.000	0.000	0.000	0.000	0.000	0.000	0.000	0.000	0.000	0.000	0.000	0.055	-9.852	3.552	-4.774	4.409
TR.2 A1 H1 S	Gegham 1	0.000	0.000	0.000	1.000	0.000	0.000	0.000	0.000	0.000	0.000	0.000	0.000	0.000	0.000	-0.625	-9.911	3.587	-4.241	3.576
TR.2 A1 H1 S	Gegham 1	0.000	0.000	0.000	1.000	0.000	0.000	0.000	0.000	0.000	0.000	0.000	0.000	0.000	0.000	-1.042	-10.933	3.413	-4.608	3.364
TR.2 A1 H1 S	Gegham 1	0.000	0.000	0.000	1.000	0.000	0.000	0.000	0.000	0.000	0.000	0.000	0.000	0.000	0.000	0.907	-11.021	3.371	-4.704	3.528
TR.2 A1 H1 S	Kelbadjar	0.000	0.000	0.000	0.000	0.000	0.000	0.000	0.000	1.000	0.000	0.000	0.000	0.000	0.000	-8.050	-8.339	2.056	1.719	-0.684
TR.2 A1 H1 S	Gegham 1	0.000	0.000	0.000	1.000	0.000	0.000	0.000	0.000	0.000	0.000	0.000	0.000	0.000	0.000	-1.744	-11.060	2.832	-4.222	1.639
TR.2 A1 H1 S	Kelbadjar	0.000	0.000	0.000	0.000	0.000	0.000	0.000	0.000	1.000	0.000	0.000	0.000	0.000	0.000	-9.260	-9.800	0.585	3.885	-0.776
TR.2 A1 H1 S	Gegham 1	0.000	0.000	0.000	1.000	0.000	0.000	0.000	0.000	0.000	0.000	0.000	0.000	0.000	0.000	-1.716	-11.809	3.282	-4.776	5.746
TR.2 A1 H1 S	Kelbadjar	0.000	0.000	0.000	0.000	0.000	0.000	0.000	0.000	1.000	0.000	0.000	0.000	0.000	0.000	-9.895	-9.667	0.624	4.306	-1.319
TR.2 A1 H1 S	Kelbadjar	0.000	0.000	0.000	0.000	0.000	0.000	0.000	0.000	1.000	0.000	0.000	0.000	0.000	0.000	-8.565	-9.624	1.662	3.333	-1.438
TR.2 A1 H1 S	Gegham 1	0.000	0.000	0.000	1.000	0.000	0.000	0.000	0.000	0.000	0.000	0.000	0.000	0.000	0.000	-2.113	-12.530	2.274	-4.560	5.568
TR.2 A1 H1 S	Gegham 1	0.000	0.000	0.000	1.000	0.000	0.000	0.000	0.000	0.000	0.000	0.000	0.000	0.000	0.000	-1.672	-10.979	3.823	-4.935	7.382
TR.2 A1 H1 S	Gegham 1	0.000	0.000	0.000	1.000	0.000	0.000	0.000	0.000	0.000	0.000	0.000	0.000	0.000	0.000	-1.078	-10.564	2.983	-4.375	5.342
TR.2 A1 H1 S	Gegham 1	0.000	0.000	0.000	1.000	0.000	0.000	0.000	0.000	0.000	0.000	0.000	0.000	0.000	0.000	-0.358	-12.040	2.565	-4.677	5.022
TR.2 A1 H1 S	Kelbadjar	0.000	0.000	0.000	0.000	0.000	0.000	0.000	0.000	1.000	0.000	0.000	0.000	0.000	0.000	-9.611	-9.273	0.551	4.362	-1.553
TR.2 A1 H1 S	Kelbadjar	0.000	0.000	0.000	0.000	0.000	0.000	0.000	0.000	1.000	0.000	0.000	0.000	0.000	0.000	-9.200	-9.729	0.100	3.222	-1.676
TR.2 A1 H1 S	Kelbadjar	0.000	0.000	0.000	0.000	0.000	0.000	0.000	0.000	1.000	0.000	0.000	0.000	0.000	0.000	-11.132	-9.000	0.098	5.340	-2.058
TR.2 A1 H1 S	Gegham 1	0.000	0.000	0.000	1.000	0.000	0.000	0.000	0.000	0.000	0.000	0.000	0.000	0.000	0.000	-0.131	-10.212	3.543	-4.700	3.770
TR.2 A1 H1 S	Syunik	0.000	0.000	0.000	0.000	0.000	0.000	0.000	0.000	0.000	0.000	0.000	0.000	1.000	0.000	-6.167	-11.588	-2.475	2.747	-0.319
TR.2 A1 H1 S	Gegham 1	0.000	0.000	0.000	1.000	0.000	0.000	0.000	0.000	0.000	0.000	0.000	0.000	0.000	0.000	-0.999	-9.433	4.476	-4.657	2.668
TR.2 A1 H1 S	Gegham 1	0.000	0.000	0.000	1.000	0.000	0.000	0.000	0.000	0.000	0.000	0.000	0.000	0.000	0.000	-0.990	-11.098	2.960	-4.816	5.058
TR.2 A1 H1 S	Gegham 1	0.000	0.000	0.000	1.000	0.000	0.000	0.000	0.000	0.000	0.000	0.000	0.000	0.000	0.000	0.344	-10.767	3.686	-4.790	3.530
TR.2 A1 H1 S	Gegham 1	0.000	0.000	0.000	1.000	0.000	0.000	0.000	0.000	0.000	0.000	0.000	0.000	0.000	0.000	-0.755	-11.624	3.249	-5.001	4.348
TR.2 A1 H1 S	Gegham 1	0.000	0.000	0.000	1.000	0.000	0.000	0.000	0.000	0.000	0.000	0.000	0.000	0.000	0.000	0.693	-10.913	3.194	-5.001	6.094
TR.2 A1 H1 S	Kelbadjar	0.000	0.000	0.000	0.000	0.000	0.000	0.000	0.000	1.000	0.000	0.000	0.000	0.000	0.000	-9.557	-9.318	0.637	4.572	-1.429
TR.2 A1 H1 S	Kelbadjar	0.000	0.000	0.000	0.000	0.000	0.000	0.000	0.000	1.000	0.000	0.000	0.000	0.000	0.000	-10.411	-9.706	0.140	5.434	-1.868
TR.2 A1 H1 S	Kelbadjar	0.000	0.000	0.000	0.000	0.000	0.000	0.000	0.000	1.000	0.000	0.000	0.000	0.000	0.000	-8.346	-8.999	-0.333	3.875	-1.264
TR.2 A1 H1 S	Kelbadjar	0.000	0.000	0.000	0.000	0.000	0.000	0.000	0.000	1.000	0.000	0.000	0.000	0.000	0.000	-10.344	-10.423	-0.946	6.006	-1.827
TR.2 A1 H1 S	Kelbadjar	0.000	0.000	0.000	0.000	0.000	0.000	0.000	0.000	1.000	0.000	0.000	0.000	0.000	0.000	-9.393	-10.002	0.552	6.363	-2.259
TR.2 A1 H1 S	Gegham 1	0.000	0.000	0.000	1.000	0.000	0.000	0.000	0.000	0.000	0.000	0.000	0.000	0.000	0.000	-1.816	-11.819	3.862	-4.822	6.533
TR.2 A1 H1 S	Gegham 1	0.000	0.000	0.000	1.000	0.000	0.000	0.000	0.000	0.000	0.000	0.000	0.000	0.000	0.000	-1.689	-11.075	3.884	-4.748	4.591
TR.2 A1 H1 S	Kelbadjar	0.000	0.000	0.000	0.000	0.000	0.000	0.000	0.000	1.000	0.000	0.000	0.000	0.000	0.000	-8.783	-9.670	0.176	4.009	-1.700
TR.2 A1 H1 S	Kelbadjar	0.000	0.000	0.000	0.000	0.000	0.000	0.000	0.000	1.000	0.000	0.000	0.000	0.000	0.000	-9.697	-9.899	-0.481	4.362	-1.776
TR.2 A1 H1 S	Gegham 1	0.000	0.000	0.000	1.000	0.000	0.000	0.000	0.000	0.000	0.000	0.000	0.000	0.000	0.000	-0.079	-11.491	3.768	-4.442	3.644

TR.2 A1 H1 S	Kelbadjar	0.000	0.000	0.000	0.000	0.000	0.000	0.000	0.000	1.000	0.000	0.000	0.000	0.000	0.000	-9.114	-9.628	0.606	4.599	-1.660
TR.2 A1 H1 S	Kelbadjar	0.000	0.000	0.000	0.000	0.000	0.000	0.000	0.000	1.000	0.000	0.000	0.000	0.000	0.000	-9.957	-9.580	0.352	6.043	-2.891
TR.2 A1 H1 S	Gegham 1	0.000	0.000	0.000	1.000	0.000	0.000	0.000	0.000	0.000	0.000	0.000	0.000	0.000	0.000	-1.151	-10.479	3.974	-4.862	6.577
TR.2 A1 H1 S	Kelbadjar	0.000	0.000	0.000	0.000	0.000	0.000	0.000	0.000	1.000	0.000	0.000	0.000	0.000	0.000	-9.464	-9.734	1.277	3.800	-1.449
TR.2 A1 H1 S	Kelbadjar	0.000	0.000	0.000	0.000	0.000	0.000	0.000	0.000	1.000	0.000	0.000	0.000	0.000	0.000	-9.440	-9.480	-0.280	4.749	-1.857
TR.2 A1 H1 S	Kelbadjar	0.000	0.000	0.000	0.000	0.000	0.000	0.000	0.000	1.000	0.000	0.000	0.000	0.000	0.000	-9.878	-8.970	0.888	3.740	-1.977
TR.2 A1 H1 S	Gegham 1	0.000	0.000	0.000	1.000	0.000	0.000	0.000	0.000	0.000	0.000	0.000	0.000	0.000	0.000	-0.807	-10.530	3.213	-4.589	4.517
TR.2 A1 H1 S	Kelbadjar	0.000	0.000	0.000	0.000	0.000	0.000	0.000	0.000	1.000	0.000	0.000	0.000	0.000	0.000	-11.426	-10.066	-1.033	5.363	-1.328
TR.2 A1 H1 S	Gegham 1	0.000	0.000	0.000	1.000	0.000	0.000	0.000	0.000	0.000	0.000	0.000	0.000	0.000	0.000	-0.464	-10.172	3.377	-4.447	5.133
TR.2 A1 H1 S	Kelbadjar	0.000	0.000	0.000	0.000	0.000	0.000	0.000	0.000	1.000	0.000	0.000	0.000	0.000	0.000	-9.864	-8.103	-0.241	4.063	-1.819
TR.2 A1 H1 S	Gegham 1	0.000	0.000	0.000	1.000	0.000	0.000	0.000	0.000	0.000	0.000	0.000	0.000	0.000	0.000	0.248	-10.107	3.021	-4.679	5.481
TR.2 A1 H1 S	Kelbadjar	0.000	0.000	0.000	0.000	0.000	0.000	0.000	0.000	1.000	0.000	0.000	0.000	0.000	0.000	-9.336	-9.118	2.093	3.625	-1.772
TR.2 A1 H1 S	Syunik	0.000	0.000	0.000	0.000	0.000	0.000	0.000	0.000	0.000	0.000	0.000	0.000	1.000	0.000	-6.856	-13.415	-2.506	4.569	-0.719
TR.2 A1 H1 S	Kelbadjar	0.000	0.000	0.000	0.000	0.000	0.000	0.000	0.000	1.000	0.000	0.000	0.000	0.000	0.000	-10.130	-8.995	-1.090	4.962	-1.611
TR.2 A1 H1 S	Syunik	0.000	0.000	0.000	0.000	0.000	0.000	0.000	0.000	0.448	0.000	0.000	0.000	0.552	0.000	-11.244	-10.063	-1.590	6.257	-2.163
TR.2 A1 H1 S	Syunik	0.000	0.000	0.000	0.000	0.000	0.000	0.000	0.000	0.004	0.000	0.000	0.000	0.996	0.000	-9.485	-10.859	-1.316	4.465	-1.713
TR.2 A1 H1 S	Kelbadjar	0.000	0.000	0.000	0.000	0.000	0.000	0.000	0.000	1.000	0.000	0.000	0.000	0.000	0.000	-10.347	-9.364	1.206	4.991	-1.424
TR.2 A1 H1 S	Kelbadjar	0.000	0.000	0.000	0.000	0.000	0.000	0.000	0.000	1.000	0.000	0.000	0.000	0.000	0.000	-7.895	-7.656	1.326	4.088	-1.993
Tr2 A2-A3 HO	Gegham 1	0.000	0.000	0.000	1.000	0.000	0.000	0.000	0.000	0.000	0.000	0.000	0.000	0.000	0.000	-0.872	-9.796	3.253	-4.415	2.098
Tr2 A2-A3 HO	Kelbadjar	0.000	0.000	0.000	0.000	0.000	0.000	0.000	0.000	1.000	0.000	0.000	0.000	0.000	0.000	-8.724	-9.458	1.126	2.984	-1.197
Tr2 A2-A3 HO	Gegham 1	0.000	0.000	0.000	1.000	0.000	0.000	0.000	0.000	0.000	0.000	0.000	0.000	0.000	0.000	-0.149	-10.343	4.293	-4.526	5.066
Tr2 A2-A3 HO	Kelbadjar	0.000	0.000	0.000	0.000	0.000	0.000	0.000	0.000	1.000	0.000	0.000	0.000	0.000	0.000	-9.870	-10.024	1.198	4.548	-1.905
Tr2 A2-A3 HO	Kelbadjar	0.000	0.000	0.000	0.000	0.000	0.000	0.000	0.000	1.000	0.000	0.000	0.000	0.000	0.000	-9.432	-9.635	-0.311	4.085	-0.957
Tr2 A2-A3 HO	Kelbadjar	0.000	0.000	0.000	0.000	0.000	0.000	0.000	0.000	1.000	0.000	0.000	0.000	0.000	0.000	-8.788	-8.990	1.252	3.043	-1.140
Tr2 A2-A3 HO	Gegham 1	0.000	0.000	0.000	1.000	0.000	0.000	0.000	0.000	0.000	0.000	0.000	0.000	0.000	0.000	-1.556	-11.215	3.192	-4.030	2.310
Tr2 A2-A3 HO	Kelbadjar	0.000	0.000	0.000	0.000	0.000	0.000	0.000	0.000	1.000	0.000	0.000	0.000	0.000	0.000	-9.508	-9.898	0.715	3.728	-1.825
Tr2 A2-A3 HO	Kelbadjar	0.000	0.000	0.000	0.000	0.000	0.000	0.000	0.000	1.000	0.000	0.000	0.000	0.000	0.000	-9.047	-9.229	1.048	3.940	-2.128
Tr2 A2-A3 HO	Kelbadjar	0.000	0.000	0.000	0.000	0.000	0.000	0.000	0.000	1.000	0.000	0.000	0.000	0.000	0.000	-8.944	-10.554	1.177	3.190	-0.726
Tr2 A2-A3 HO	Gegham 1	0.000	0.000	0.000	1.000	0.000	0.000	0.000	0.000	0.000	0.000	0.000	0.000	0.000	0.000	-0.692	-12.804	2.625	-4.704	3.497
Tr2 A2-A3 HO	Kelbadjar	0.000	0.000	0.000	0.000	0.000	0.000	0.000	0.000	1.000	0.000	0.000	0.000	0.000	0.000	-9.287	-10.502	1.279	3.642	-1.112
Tr2 A2-A3 HO	Gegham 1	0.000	0.000	0.000	1.000	0.000	0.000	0.000	0.000	0.000	0.000	0.000	0.000	0.000	0.000	-1.370	-12.253	3.018	-4.939	4.909
Tr2 A2-A3 HO	Kelbadjar	0.000	0.000	0.000	0.000	0.000	0.000	0.000	0.000	1.000	0.000	0.000	0.000	0.000	0.000	-6.896	-9.713	1.187	3.910	-0.952
Tr2 A2-A3 HO	Arteni	0.000	1.000	0.000	0.000	0.000	0.000	0.000	0.000	0.000	0.000	0.000	0.000	0.000	0.000	-4.180	0.872	6.021	-3.213	-6.926
Tr2 A2-A3 HO	Kelbadjar	0.000	0.000	0.000	0.000	0.000	0.000	0.000	0.000	1.000	0.000	0.000	0.000	0.000	0.000	-8.866	-9.554	1.642	3.810	-1.530
Tr2 A2-A3 HO	Gegham 1	0.000	0.000	0.000	1.000	0.000	0.000	0.000	0.000	0.000	0.000	0.000	0.000	0.000	0.000	-0.357	-10.143	3.413	-4.577	3.622
Tr2 A2-A3 HO	Kelbadjar	0.000	0.000	0.000	0.000	0.000	0.000	0.000	0.000	1.000	0.000	0.000	0.000	0.000	0.000	-8.612	-10.499	0.344	3.114	-0.765
Tr2 A2-A3 HO	Gegham 1	0.000	0.000	0.000	1.000	0.000	0.000	0.000	0.000	0.000	0.000	0.000	0.000	0.000	0.000	-0.924	-12.100	2.960	-4.031	4.533
Tr2 A2-A3 HO	Arteni	0.000	1.000	0.000	0.000	0.000	0.000	0.000	0.000	0.000	0.000	0.000	0.000	0.000	0.000	-0.831	2.204	3.607	-3.643	-6.850
Tr2 A2-A3 HO	Gegham 1	0.000	0.000	0.000	1.000	0.000	0.000	0.000	0.000	0.000	0.000	0.000	0.000	0.000	0.000	1.351	-9.922	4.375	-4.475	1.901
Tr2 A2-A3 HO	Gegham 1	0.000	0.000	0.000	1.000	0.000	0.000	0.000	0.000	0.000	0.000	0.000	0.000	0.000	0.000	-1.590	-10.110	3.336	-4.929	3.943
Tr2 A2-A3 HO	Gegham 1	0.000	0.000	0.000	1.000	0.000	0.000	0.000	0.000	0.000	0.000	0.000	0.000	0.000	0.000	-0.794	-11.869	3.020	-4.672	3.408
Tr2 A2-A3 HO	Gegham 1	0.000	0.000	0.000	1.000	0.000	0.000	0.000	0.000	0.000	0.000	0.000	0.000	0.000	0.000	-0.170	-10.811	2.836	-4.642	2.732
Tr2 A2-A3 HO	Kelbadjar	0.000	0.000	0.000	0.000	0.000	0.000	0.000	0.000	1.000	0.000	0.000	0.000	0.000	0.000	-9.157	-10.439	1.289	4.042	-2.223
Tr2 A2-A3 HO	Syunik	0.000	0.000	0.000	0.000	0.000	0.000	0.000	0.000	0.000	0.000	0.000	0.000	1.000	0.000	-7.241	-12.476	-3.128	4.138	-1.312
Tr2 A2-A3 HO	Gegham 1	0.000	0.000	0.000	1.000	0.000	0.000	0.000	0.000	0.000	0.000	0.000	0.000	0.000	0.000	-0.361	-11.397	2.721	-4.591	4.424
Tr2 A2-A3 HO	Kelbadjar	0.000	0.000	0.000	0.000	0.000	0.000	0.000	0.000	1.000	0.000	0.000	0.000	0.000	0.000	-9.851	-10.378	1.330	4.764	-1.701
Tr2 A2-A3 HO	Kelbadjar	0.000	0.000	0.000	0.000	0.000	0.000	0.000	0.000	1.000	0.000	0.000	0.000	0.000	0.000	-9.374	-9.897	-0.202	4.575	-1.303
Tr2 A2-A3 HO	Kelbadjar	0.000	0.000	0.000	0.000	0.000	0.000	0.000	0.000	1.000	0.000	0.000	0.000	0.000	0.000	-8.941	-8.566	0.715	3.070	-1.492
Tr2 A2-A3 HO	Kelbadjar	0.000	0.000	0.000	0.000	0.000	0.000	0.000	0.000	0.992	0.000	0.000	0.000	0.008	0.000	-9.861	-11.214	-1.074	4.675	-1.416
Tr2 A2-A3 HO	Kelbadjar	0.000	0.000	0.000	0.000	0.000	0.000	0.000	0.000	1.000	0.000	0.000	0.000	0.000	0.000	-10.674	-9.905	0.174	5.603	-2.354
Tr2 A2-A3 HO	Gegham 1	0.000	0.000	0.000	1.000	0.000	0.000	0.000	0.000	0.000	0.000	0.000	0.000	0.000	0.000	0.159	-10.835	3.835	-4.549	2.784
Tr2 A2-A3 HO	Gegham 1	0.000	0.000	0.000	1.000	0.000	0.000	0.000	0.000	0.000	0.000	0.000	0.000	0.000	0.000	-1.551	-12.467	2.880	-4.485	4.432
Tr2 A2-A3 HO	Syunik	0.000	0.000	0.000	0.000	0.000	0.000	0.000	0.000	0.000	0.000	0.000	0.000	1.000	0.000	-7.455	-12.490	-1.293	4.525	-1.979
Tr2 A2-A3 HO	Hatis	0.000	0.000	0.000	0.000	0.000	0.000	1.000	0.000	0.000	0.000	0.000	0.000	0.000	0.000	18.068	3.241	-1.601	-0.260	-1.516
Tr2 A2-A3 Ho	Gegham 1	0.000	0.000	0.000	1.000	0.000	0.000	0.000	0.000	0.000	0.000	0.000	0.000	0.000	0.000	-0.386	-12.041	2.621	-4.380	4.103
Tr2 A2-A3 Ho	Kelbadjar	0.000	0.000	0.000	0.000	0.000	0.000	0.000	0.000	1.000	0.000	0.000	0.000	0.000	0.000	-8.576	-8.551	0.942	2.960	-1.752
Tr2 A2-A3 Ho	Kelbadjar	0.000	0.000	0.000	0.000	0.000	0.000	0.000	0.000	1.000	0.000	0.000	0.000	0.000	0.000	-9.645	-8.817	2.009	3.887	-1.551
Tr2 A2-A3 Ho	Kelbadjar	0.000	0.000	0.000	0.000	0.000	0.000	0.000	0.000	1.000	0.000	0.000	0.000	0.000	0.000	-9.795	-10.252	1.522	4.401	-1.453
Tr2 A2-A3 Ho	Kelbadjar	0.000	0.000	0.000	0.000	0.000	0.000	0.000	0.000	1.000	0.000	0.000	0.000	0.000	0.000	-10.174	-9.390	-0.457	4.461	-1.563
Tr2 A2-A3 Ho	Kelbadjar	0.000	0.000	0.000	0.000	0.000	0.000	0.000	0.000	1.000	0.000	0.000	0.000	0.000	0.000	-8.929	-8.770	1.450	3.226	-1.658
Tr2 A2-A3 Ho	Gegham 1	0.000	0.000	0.000	1.000	0.000	0.000	0.000	0.000	0.000	0.000	0.000	0.000	0.000	0.000	-0.823	-10.664	2.633	-4.516	3.626
Tr2 A2-A3 Ho	Kelbadjar	0.000	0.000	0.000	0.000	0.000	0.000	0.000	0.000	1.000	0.000	0.000	0.000	0.000	0.000	-9.335	-9.736	1.331	4.660	-1.887
Tr2 A2-A3 Ho	Gegham 1	0.000	0.000	0.000	1.000	0.000	0.000	0.000	0.000	0.000	0.000	0.000	0.000	0.000	0.000	-1.078	-10.071	3.704	-4.565	6.561
Tr2 A2-A3 Ho	Kelbadjar	0.000	0.000	0.000	0.000	0.000	0.000	0.000	0.000	1.000	0.000	0.000	0.000	0.000	0.000	-10.295	-9.196	-1.362	5.477	-2.366
Tr2 A2-A3 Ho	Gegham 1	0.000	0.000	0.000	1.000	0.000	0.000	0.000	0.000	0.000	0.000	0.000	0.000	0.000	0.000	-0.396	-11.527	2.604	-3.923	3.977
Tr2 A2-A3 Ho	Kelbadjar	0.000	0.000	0.000	0.000	0.000	0.000	0.000	0.000	1.000	0.000	0.000	0.000	0.000	0.000	-10.419	-10.164	0.928	5.044	-1.797
Tr2 A2-A3 Ho	Kelbadjar	0.000	0.000	0.000	0.000	0.000	0.000	0.000	0.000	1.000	0.000	0.000	0.000	0.000	0.000	-10.133	-10.125	-1.189	4.588	-1.688

Tr2 A2-A3 Ho	Gegham 1	0.000	0.000	0.000	1.000	0.000	0.000	0.000	0.000	0.000	0.000	0.000	0.000	0.000	0.000	-0.645	-11.226	2.917	-4.164	3.555
Tr2 A2-A3 Ho	Kelbadjar	0.000	0.000	0.000	0.000	0.000	0.000	0.000	0.000	1.000	0.000	0.000	0.000	0.000	0.000	-9.795	-8.841	-0.367	3.577	-1.705
Tr2 A2-A3 Ho	Kelbadjar	0.000	0.000	0.000	0.000	0.000	0.000	0.000	0.000	1.000	0.000	0.000	0.000	0.000	0.000	-9.926	-10.679	0.445	3.890	-1.584
Tr2 A2-A3 Ho	Kelbadjar	0.000	0.000	0.000	0.000	0.000	0.000	0.000	0.000	1.000	0.000	0.000	0.000	0.000	0.000	-8.610	-8.478	-0.071	3.401	-1.451
Tr2 A2-A3 Ho	Kelbadjar	0.000	0.000	0.000	0.000	0.000	0.000	0.000	0.000	1.000	0.000	0.000	0.000	0.000	0.000	-10.220	-9.813	-0.149	5.811	-1.889
Tr2 A2-A3 Ho	Gegham 1	0.000	0.000	0.000	1.000	0.000	0.000	0.000	0.000	0.000	0.000	0.000	0.000	0.000	0.000	-0.353	-10.731	2.977	-4.758	4.355
Tr2 A2-A3 Ho	Gegham 1	0.000	0.000	0.000	1.000	0.000	0.000	0.000	0.000	0.000	0.000	0.000	0.000	0.000	0.000	-0.870	-11.243	2.887	-4.622	3.191
Tr2 A2-A3 Ho	Gegham 1	0.000	0.000	0.000	1.000	0.000	0.000	0.000	0.000	0.000	0.000	0.000	0.000	0.000	0.000	0.583	-10.095	3.353	-4.653	4.660
Tr2 A2-A3 Ho	Kelbadjar	0.000	0.000	0.000	0.000	0.000	0.000	0.000	0.000	1.000	0.000	0.000	0.000	0.000	0.000	-8.571	-9.891	0.500	4.269	-1.298
Tr2 A2-A3 Ho	Kelbadjar	0.000	0.000	0.000	0.000	0.000	0.000	0.000	0.000	1.000	0.000	0.000	0.000	0.000	0.000	-8.427	-9.556	-0.648	3.724	-1.542
Tr2 A2-A3 Ho	Kelbadjar	0.000	0.000	0.000	0.000	0.000	0.000	0.000	0.000	1.000	0.000	0.000	0.000	0.000	0.000	-9.715	-10.395	-1.056	4.944	-1.586
Tr2 A2-A3 Ho	Syunik	0.000	0.000	0.000	0.000	0.000	0.000	0.000	0.000	0.000	0.000	0.000	0.000	1.000	0.000	-6.095	-11.755	-1.611	4.251	-1.823
Tr2 A2-A3 Ho	Gegham 1	0.000	0.000	0.000	1.000	0.000	0.000	0.000	0.000	0.000	0.000	0.000	0.000	0.000	0.000	-1.148	-11.619	3.187	-4.751	5.243
Tr2 A2-A3 Ho	Syunik	0.000	0.000	0.000	0.000	0.000	0.000	0.000	0.000	0.000	0.000	0.000	0.000	1.000	0.000	-5.908	-10.846	-1.802	3.807	-1.270
Tr2 A2-A3 Ho	Gegham 1	0.000	0.000	0.000	1.000	0.000	0.000	0.000	0.000	0.000	0.000	0.000	0.000	0.000	0.000	-0.378	-9.761	3.936	-5.225	5.069
Tr2 A2-A3 Ho	Gegham 1	0.000	0.000	0.000	1.000	0.000	0.000	0.000	0.000	0.000	0.000	0.000	0.000	0.000	0.000	0.055	-10.659	2.995	-4.362	4.169
Tr2 A2-A3 Ho	Gegham 1	0.000	0.000	0.000	1.000	0.000	0.000	0.000	0.000	0.000	0.000	0.000	0.000	0.000	0.000	-1.394	-13.458	2.476	-4.675	6.186
Tr2 A2-A3 Ho	Gegham 1	0.000	0.000	0.000	1.000	0.000	0.000	0.000	0.000	0.000	0.000	0.000	0.000	0.000	0.000	-0.483	-10.661	3.335	-4.548	5.405
Tr2 A2-A3 Ho	Gegham 1	0.000	0.000	0.000	1.000	0.000	0.000	0.000	0.000	0.000	0.000	0.000	0.000	0.000	0.000	-1.247	-10.128	3.007	-4.539	3.486
Tr2 A2-A3 Ho	Kelbadjar	0.000	0.000	0.000	0.000	0.000	0.000	0.000	0.000	1.000	0.000	0.000	0.000	0.000	0.000	-8.962	-9.250	0.498	4.253	-1.345
Tr2 A2-A3 Ho	Gegham 1	0.000	0.000	0.000	1.000	0.000	0.000	0.000	0.000	0.000	0.000	0.000	0.000	0.000	0.000	-1.691	-10.259	3.272	-4.300	3.596
Tr2 A2-A3 Ho	Gegham 1	0.000	0.000	0.000	1.000	0.000	0.000	0.000	0.000	0.000	0.000	0.000	0.000	0.000	0.000	0.001	-10.303	3.469	-4.857	4.890
Tr2 A2-A3 Ho	Gegham 1	0.000	0.000	0.000	1.000	0.000	0.000	0.000	0.000	0.000	0.000	0.000	0.000	0.000	0.000	-0.212	-10.897	2.407	-4.590	4.472
Tr2 A2-A3 Ho	Kelbadjar	0.000	0.000	0.000	0.000	0.000	0.000	0.000	0.000	1.000	0.000	0.000	0.000	0.000	0.000	-9.715	-7.436	2.108	4.390	-2.293
Tr2 A2-A3 Ho	Gegham 1	0.000	0.000	0.000	1.000	0.000	0.000	0.000	0.000	0.000	0.000	0.000	0.000	0.000	0.000	-0.797	-12.371	2.553	-4.373	6.068
Tr2 A2-A3 Ho	Gegham 1	0.000	0.000	0.000	1.000	0.000	0.000	0.000	0.000	0.000	0.000	0.000	0.000	0.000	0.000	0.781	-9.428	3.108	-4.893	5.563
Tr2 A2-A3 Ho	Syunik	0.000	0.000	0.000	0.000	0.000	0.000	0.000	0.000	0.000	0.000	0.000	0.000	1.000	0.000	-6.713	-12.081	-2.796	4.740	-0.984
Tr2 A2-A3 Ho	Kelbadjar	0.000	0.000	0.000	0.000	0.000	0.000	0.000	0.000	1.000	0.000	0.000	0.000	0.000	0.000	-9.508	-9.603	0.603	4.196	-1.870
Tr2 A2-A3 Ho	Gegham 1	0.000	0.000	0.000	1.000	0.000	0.000	0.000	0.000	0.000	0.000	0.000	0.000	0.000	0.000	-1.176	-12.424	2.433	-4.726	4.754
Tr2 A2-A3 Ho	Gegham 1	0.000	0.000	0.000	1.000	0.000	0.000	0.000	0.000	0.000	0.000	0.000	0.000	0.000	0.000	0.481	-10.148	3.058	-4.491	7.079
Tr2 A2-A3 Ho	Gegham 1	0.000	0.000	0.000	1.000	0.000	0.000	0.000	0.000	0.000	0.000	0.000	0.000	0.000	0.000	-1.547	-11.149	2.744	-4.637	4.709
Tr2 A2-A3 Ho	Gegham 1	0.000	0.000	0.000	1.000	0.000	0.000	0.000	0.000	0.000	0.000	0.000	0.000	0.000	0.000	0.600	-10.090	4.801	-5.250	5.210
Tr2 A2-A3 Ho	Kelbadjar	0.000	0.000	0.000	0.000	0.000	0.000	0.000	0.000	1.000	0.000	0.000	0.000	0.000	0.000	-9.399	-8.970	0.291	2.800	-1.356
Tr2 A2-A3 Ho	Gegham 1	0.000	0.000	0.000	1.000	0.000	0.000	0.000	0.000	0.000	0.000	0.000	0.000	0.000	0.000	-1.071	-11.352	5.479	-5.339	9.300
Tr2 A2-A3 Ho	Kelbadjar	0.000	0.000	0.000	0.000	0.000	0.000	0.000	0.000	1.000	0.000	0.000	0.000	0.000	0.000	-8.711	-8.066	0.321	3.638	-1.834
Tr2 A2-A3 Ho	Gegham 1	0.000	0.000	0.000	1.000	0.000	0.000	0.000	0.000	0.000	0.000	0.000	0.000	0.000	0.000	-1.639	-12.135	3.185	-4.557	7.596
TR.2 UN.B2	Gegham 1	0.000	0.000	0.000	1.000	0.000	0.000	0.000	0.000	0.000	0.000	0.000	0.000	0.000	0.000	-0.624	-10.438	3.110	-3.884	1.252
TR.2 UN.B2	Gegham 1	0.000	0.000	0.000	1.000	0.000	0.000	0.000	0.000	0.000	0.000	0.000	0.000	0.000	0.000	-1.222	-9.856	3.360	-4.660	2.875
TR.2 UN.B2	Gegham 1	0.000	0.000	0.000	1.000	0.000	0.000	0.000	0.000	0.000	0.000	0.000	0.000	0.000	0.000	0.058	-11.972	3.254	-4.130	2.192
TR.2 UN.B2	Gegham 1	0.000	0.000	0.000	1.000	0.000	0.000	0.000	0.000	0.000	0.000	0.000	0.000	0.000	0.000	-1.666	-11.946	2.941	-4.455	4.913
TR.2 UN.B2	Gegham 2	0.000	0.020	0.000	0.000	0.980	0.000	0.000	0.000	0.000	0.000	0.000	0.000	0.000	0.000	0.857	4.907	0.695	-2.715	-3.251
TR.2 UN.B2	Gegham 1	0.000	0.000	0.000	1.000	0.000	0.000	0.000	0.000	0.000	0.000	0.000	0.000	0.000	0.000	-1.172	-10.144	3.292	-4.457	4.149
TR.2 UN.B2	Gegham 1	0.000	0.000	0.000	1.000	0.000	0.000	0.000	0.000	0.000	0.000	0.000	0.000	0.000	0.000	-1.015	-10.573	2.926	-4.363	4.029
TR.2 UN.B2	Gegham 1	0.000	0.000	0.000	1.000	0.000	0.000	0.000	0.000	0.000	0.000	0.000	0.000	0.000	0.000	-0.157	-8.531	4.454	-4.971	7.450
TR.2 UN.B2	Gegham 1	0.000	0.000	0.000	1.000	0.000	0.000	0.000	0.000	0.000	0.000	0.000	0.000	0.000	0.000	-0.039	-9.685	3.221	-4.420	4.960
TR.2 UN.B2	Kelbadjar	0.000	0.000	0.000	0.000	0.000	0.000	0.000	0.000	1.000	0.000	0.000	0.000	0.000	0.000	-9.814	-10.252	0.774	4.529	-1.648
TR.2 UN-C0	Gegham 1	0.000	0.000	0.000	1.000	0.000	0.000	0.000	0.000	0.000	0.000	0.000	0.000	0.000	0.000	-0.183	-11.888	2.847	-4.845	4.146
TR.2 UN-C0	Gegham 1	0.000	0.000	0.000	1.000	0.000	0.000	0.000	0.000	0.000	0.000	0.000	0.000	0.000	0.000	0.606	-8.981	4.700	-5.074	4.784
TR.2 UN-C0	Gegham 1	0.000	0.000	0.000	1.000	0.000	0.000	0.000	0.000	0.000	0.000	0.000	0.000	0.000	0.000	-0.371	-10.471	1.995	-4.346	4.976
TR.2 UN B1	Gegham 1	0.000	0.000	0.000	1.000	0.000	0.000	0.000	0.000	0.000	0.000	0.000	0.000	0.000	0.000	-0.683	-11.297	2.412	-4.585	3.584
TR.2 UN B1	Gegham 1	0.000	0.000	0.000	1.000	0.000	0.000	0.000	0.000	0.000	0.000	0.000	0.000	0.000	0.000	-1.678	-11.871	2.523	-4.208	2.576
TR.2 H2.S2 U	Gegham 2	0.000	0.000	0.000	0.000	1.000	0.000	0.000	0.000	0.000	0.000	0.000	0.000	0.000	0.000	-2.575	5.324	6.898	-3.718	-5.906
TR.2 UN-C2	Gegham 1	0.000	0.000	0.000	1.000	0.000	0.000	0.000	0.000	0.000	0.000	0.000	0.000	0.000	0.000	-1.092	-11.763	3.025	-4.117	2.293
TR.2 UN-C2	Gegham 1	0.000	0.000	0.000	1.000	0.000	0.000	0.000	0.000	0.000	0.000	0.000	0.000	0.000	0.000	-1.620	-11.241	2.686	-4.126	3.338
TR.2 UN-C2	Arteni	0.000	1.000	0.000	0.000	0.000	0.000	0.000	0.000	0.000	0.000	0.000	0.000	0.000	0.000	-1.095	3.172	7.928	-3.769	-6.269
TR.2 UN-C2	Gegham 1	0.000	0.000	0.000	1.000	0.000	0.000	0.000	0.000	0.000	0.000	0.000	0.000	0.000	0.000	-0.454	-10.094	2.786	-4.134	4.691
TR.2 UN-B0	Kelbadjar	0.000	0.000	0.000	0.000	0.000	0.000	0.000	0.000	1.000	0.000	0.000	0.000	0.000	0.000	-8.662	-9.349	0.076	3.562	-0.978
TR.2 UN-B0	Gegham 1	0.000	0.000	0.000	1.000	0.000	0.000	0.000	0.000	0.000	0.000	0.000	0.000	0.000	0.000	-1.798	-11.795	2.384	-3.701	2.154
TR.2 UN-B0	Kelbadjar	0.000	0.000	0.000	0.000	0.000	0.000	0.000	0.000	0.539	0.000	0.000	0.000	0.461	0.000	-7.000	-11.131	-1.391	1.369	-0.714
TR.2 UN-B0	Kelbadjar	0.000	0.000	0.000	0.000	0.000	0.000	0.000	0.000	1.000	0.000	0.000	0.000	0.000	0.000	-8.694	-9.455	1.272	2.367	-1.672
TR.2 UN-B0	Kelbadjar	0.000	0.000	0.000	0.000	0.000	0.000	0.000	0.000	1.000	0.000	0.000	0.000	0.000	0.000	-8.506	-9.818	-1.047	3.989	-1.285
TR.2 UN-B0	Gegham 1	0.000	0.000	0.000	1.000	0.000	0.000	0.000	0.000	0.000	0.000	0.000	0.000	0.000	0.000	-0.873	-10.563	3.106	-4.749	5.217
TR.2 UN-B0	Gegham 1	0.000	0.000	0.000	1.000	0.000	0.000	0.000	0.000	0.000	0.000	0.000	0.000	0.000	0.000	-0.376	-9.905	3.127	-4.537	3.275
TR.2 UN-B0	Gegham 1	0.000	0.000	0.000	1.000	0.000	0.000	0.000	0.000	0.000	0.000	0.000	0.000	0.000	0.000	-1.381	-11.293	3.383	-5.251	7.654
TR.2 UN-B0	Syunik	0.000	0.000	0.000	0.000	0.000	0.000	0.000	0.000	0.000	0.000	0.000	0.000	1.000	0.000	-6.288	-13.062	-3.907	5.047	-0.635
TR.2 UN-B0	Gegham 1	0.000	0.000	0.000	1.000	0.000	0.000	0.000	0.000	0.000	0.000	0.000	0.000	0.000	0.000	-0.832	-9.528	5.378	-5.440	7.265
TR.2 H2 S2 A	Gegham 1	0.000	0.000	0.000	1.000	0.000	0.000	0.000	0.000	0.000	0.000	0.000	0.000	0.000	0.000	-0.426	-11.804	3.060	-4.623	2.998

TR.2 H2 S2 A	Gegham 1	0.000	0.000	0.000	1.000	0.000	0.000	0.000	0.000	0.000	0.000	0.000	0.000	0.000	0.000	-2.507	-12.729	2.674	-4.110	3.441
TR.2 H2 S2 A	Arteni	0.000	1.000	0.000	0.000	0.000	0.000	0.000	0.000	0.000	0.000	0.000	0.000	0.000	0.000	-4.900	2.097	5.325	-3.886	-8.294
TR.2 H2 S2 A	Gegham 1	0.000	0.000	0.000	1.000	0.000	0.000	0.000	0.000	0.000	0.000	0.000	0.000	0.000	0.000	0.198	-11.158	2.971	-4.916	5.662
TR.2 H2 S2 A	Gegham 1	0.000	0.000	0.000	1.000	0.000	0.000	0.000	0.000	0.000	0.000	0.000	0.000	0.000	0.000	-2.448	-11.874	3.684	-4.642	7.344
TR.2 H2 S2 A	Arteni	0.000	1.000	0.000	0.000	0.000	0.000	0.000	0.000	0.000	0.000	0.000	0.000	0.000	0.000	-4.129	3.791	5.023	-3.093	-3.627
TR.2 H2 S2 A	Gegham 1	0.000	0.000	0.000	1.000	0.000	0.000	0.000	0.000	0.000	0.000	0.000	0.000	0.000	0.000	-0.811	-9.373	5.179	-5.316	7.487
TR.2 H2 S2 A	Gegham 1	0.000	0.000	0.000	1.000	0.000	0.000	0.000	0.000	0.000	0.000	0.000	0.000	0.000	0.000	-1.161	-10.926	3.901	-4.552	7.541
TR-2 Unit-A1	Kelbadjar	0.000	0.000	0.000	0.000	0.000	0.000	0.000	0.000	1.000	0.000	0.000	0.000	0.000	0.000	-8.629	-9.566	0.223	3.536	-0.834
TR-2 Unit-A1	Gegham 1	0.000	0.000	0.000	1.000	0.000	0.000	0.000	0.000	0.000	0.000	0.000	0.000	0.000	0.000	-0.878	-11.090	3.159	-4.575	6.332
Tr2 C0 H3 Sp	Gegham 1	0.000	0.000	0.000	1.000	0.000	0.000	0.000	0.000	0.000	0.000	0.000	0.000	0.000	0.000	-1.204	-9.342	3.552	-4.713	3.314
Tr2 Unit-A0	Gegham 1	0.000	0.000	0.000	1.000	0.000	0.000	0.000	0.000	0.000	0.000	0.000	0.000	0.000	0.000	-1.966	-11.852	2.426	-4.438	3.195
Tr2 Unit-A0	Gegham 1	0.000	0.000	0.000	1.000	0.000	0.000	0.000	0.000	0.000	0.000	0.000	0.000	0.000	0.000	-1.024	-10.502	3.668	-4.669	4.533
Tr-2 C1 H3 S	Gegham 1	0.000	0.000	0.000	1.000	0.000	0.000	0.000	0.000	0.000	0.000	0.000	0.000	0.000	0.000	-0.248	-10.693	3.709	-4.833	3.927
Tr-2 C1 H3 S	Arteni	0.000	1.000	0.000	0.000	0.000	0.000	0.000	0.000	0.000	0.000	0.000	0.000	0.000	0.000	-4.226	3.073	5.290	-3.880	-5.721
Tr-2 C1 H4 S	Gegham 1	0.000	0.000	0.000	1.000	0.000	0.000	0.000	0.000	0.000	0.000	0.000	0.000	0.000	0.000	0.368	-10.854	2.954	-4.777	4.378
Tr-2 C1 H4 S	Syunik	0.000	0.000	0.000	0.000	0.000	0.000	0.000	0.000	0.000	0.000	0.000	0.000	1.000	0.000	-4.372	-13.161	-2.663	3.468	-0.929
Tr-2 C1 H4 S	Gegham 1	0.000	0.000	0.000	1.000	0.000	0.000	0.000	0.000	0.000	0.000	0.000	0.000	0.000	0.000	-0.628	-11.313	3.823	-4.770	8.603
Tr-2 C1 H4 S	Gegham 1	0.000	0.000	0.000	1.000	0.000	0.000	0.000	0.000	0.000	0.000	0.000	0.000	0.000	0.000	1.326	-9.305	4.448	-5.301	5.822
Tr2 Unit-B0	Gegham 1	0.000	0.000	0.000	1.000	0.000	0.000	0.000	0.000	0.000	0.000	0.000	0.000	0.000	0.000	-1.080	-13.118	2.829	-4.503	3.544
Tr2 Unit-B0	Gegham 1	0.000	0.000	0.000	1.000	0.000	0.000	0.000	0.000	0.000	0.000	0.000	0.000	0.000	0.000	-1.232	-11.882	3.428	-4.400	2.986
Tr2 Unit-B0	Gegham 1	0.000	0.000	0.000	1.000	0.000	0.000	0.000	0.000	0.000	0.000	0.000	0.000	0.000	0.000	-1.025	-12.537	2.402	-4.276	2.920
Tr2 Unit-B0	Gegham 1	0.000	0.000	0.000	1.000	0.000	0.000	0.000	0.000	0.000	0.000	0.000	0.000	0.000	0.000	-0.775	-10.814	3.067	-4.718	4.545
Tr2 Unit-A1	Gegham 1	0.000	0.000	0.000	1.000	0.000	0.000	0.000	0.000	0.000	0.000	0.000	0.000	0.000	0.000	0.227	-9.964	3.819	-4.708	3.887
Tr2 Unit-A1	Syunik	0.000	0.000	0.000	0.000	0.000	0.000	0.000	0.000	0.000	0.000	0.000	0.000	1.000	0.000	-6.535	-12.461	-1.828	4.205	-0.993
Tr2 Unit-A1	Syunik	0.000	0.000	0.000	0.000	0.000	0.000	0.000	0.000	0.000	0.000	0.000	0.000	1.000	0.000	-5.232	-11.827	-1.562	3.461	-1.370
Tr2 Unit-A1	Arteni	0.000	1.000	0.000	0.000	0.000	0.000	0.000	0.000	0.000	0.000	0.000	0.000	0.000	0.000	-1.551	2.295	3.841	-3.226	-6.000
Tr2 A0 H4 Sp	Gegham 1	0.000	0.000	0.000	1.000	0.000	0.000	0.000	0.000	0.000	0.000	0.000	0.000	0.000	0.000	-0.952	-11.608	3.817	-4.940	4.868
Tr2 A0 H4 Sp	Gegham 1	0.000	0.000	0.000	1.000	0.000	0.000	0.000	0.000	0.000	0.000	0.000	0.000	0.000	0.000	-1.374	-11.271	2.375	-4.178	2.889
Tr2 A0 H4 Sp	Syunik	0.000	0.000	0.000	0.000	0.000	0.000	0.000	0.000	0.000	0.000	0.000	0.000	1.000	0.000	-8.425	-15.814	-3.385	2.794	-0.185
TR.2 UN-A0	Gegham 1	0.000	0.000	0.000	1.000	0.000	0.000	0.000	0.000	0.000	0.000	0.000	0.000	0.000	0.000	-1.385	-11.020	3.380	-4.373	2.714
TR.2 UN-A0	Gegham 1	0.000	0.000	0.000	1.000	0.000	0.000	0.000	0.000	0.000	0.000	0.000	0.000	0.000	0.000	-0.807	-9.846	3.780	-4.493	3.087
TR.2 UN-A0	Gegham 1	0.000	0.000	0.000	1.000	0.000	0.000	0.000	0.000	0.000	0.000	0.000	0.000	0.000	0.000	-1.244	-11.927	3.003	-4.587	3.818
TR.2 UN-A0	Gegham 1	0.000	0.000	0.000	1.000	0.000	0.000	0.000	0.000	0.000	0.000	0.000	0.000	0.000	0.000	-0.366	-11.496	2.813	-3.806	2.138
TR.2 UN-A0	Syunik	0.000	0.000	0.000	0.000	0.000	0.000	0.000	0.000	0.000	0.000	0.000	0.000	1.000	0.000	-8.005	-14.864	-1.670	1.731	-1.090
TR.2 UN-A0	Arteni	0.000	1.000	0.000	0.000	0.000	0.000	0.000	0.000	0.000	0.000	0.000	0.000	0.000	0.000	-0.304	2.479	4.758	-2.879	-6.867
TR.2 UN-A0	Gegham 1	0.000	0.000	0.000	1.000	0.000	0.000	0.000	0.000	0.000	0.000	0.000	0.000	0.000	0.000	-0.547	-12.419	2.487	-4.336	4.318
TR.2 UN-A0	Gegham 1	0.000	0.000	0.000	1.000	0.000	0.000	0.000	0.000	0.000	0.000	0.000	0.000	0.000	0.000	-0.727	-9.504	3.489	-4.619	5.295
TR.2 UN-A0	Gegham 1	0.000	0.000	0.000	1.000	0.000	0.000	0.000	0.000	0.000	0.000	0.000	0.000	0.000	0.000	0.685	-9.916	5.097	-4.736	3.704
TR.2 UN-A0	Gegham 1	0.000	0.000	0.000	1.000	0.000	0.000	0.000	0.000	0.000	0.000	0.000	0.000	0.000	0.000	-0.333	-9.979	3.317	-4.535	4.398
TR.2 UN-A0	Gegham 1	0.000	0.000	0.000	1.000	0.000	0.000	0.000	0.000	0.000	0.000	0.000	0.000	0.000	0.000	-1.021	-9.014	3.437	-4.757	7.395
TR.2 UN-A0	Syunik	0.000	0.000	0.000	0.000	0.000	0.000	0.000	0.000	0.000	0.000	0.000	0.000	1.000	0.000	-6.379	-13.598	-3.106	4.478	-1.056
TR.2 UN-A0	Gegham 1	0.000	0.000	0.000	1.000	0.000	0.000	0.000	0.000	0.000	0.000	0.000	0.000	0.000	0.000	-0.468	-10.069	3.213	-4.463	5.647
TR.2 UN-A0	Gegham 1	0.000	0.000	0.000	1.000	0.000	0.000	0.000	0.000	0.000	0.000	0.000	0.000	0.000	0.000	-1.609	-11.832	3.917	-4.914	8.567
TR.2 UN-A0	Syunik	0.000	0.000	0.000	0.000	0.000	0.000	0.000	0.000	0.000	0.000	0.000	0.000	1.000	0.000	-6.862	-12.872	-2.932	5.595	-1.171
TR.2 UN-A0	Gegham 1	0.000	0.000	0.000	1.000	0.000	0.000	0.000	0.000	0.000	0.000	0.000	0.000	0.000	0.000	0.378	-9.784	3.581	-4.841	4.571
TR.2 UN B0	Gegham 1	0.000	0.000	0.000	1.000	0.000	0.000	0.000	0.000	0.000	0.000	0.000	0.000	0.000	0.000	-1.756	-12.021	3.652	-3.807	0.886
TR.2 UN B0	Gegham 1	0.000	0.000	0.000	1.000	0.000	0.000	0.000	0.000	0.000	0.000	0.000	0.000	0.000	0.000	-0.250	-11.866	2.038	-4.394	3.851
TR.2 UN B0	Kelbadjar	0.000	0.000	0.000	0.000	0.000	0.000	0.000	0.000	1.000	0.000	0.000	0.000	0.000	0.000	-9.000	-10.122	2.098	2.898	-1.322
TR.2 UN B0	Gegham 1	0.000	0.000	0.000	1.000	0.000	0.000	0.000	0.000	0.000	0.000	0.000	0.000	0.000	0.000	-0.587	-10.690	3.569	-4.789	6.551
TR.2 UN-A1	Kelbadjar	0.000	0.000	0.000	0.000	0.000	0.000	0.000	0.000	1.000	0.000	0.000	0.000	0.000	0.000	-7.790	-10.036	0.710	3.749	-1.638
TR.2 UN-A1	Syunik	0.000	0.000	0.000	0.000	0.000	0.000	0.000	0.000	0.000	0.000	0.000	0.000	1.000	0.000	-7.404	-11.949	-2.161	3.820	-1.419
TR.2 UN-A1	Arteni	0.000	1.000	0.000	0.000	0.000	0.000	0.000	0.000	0.000	0.000	0.000	0.000	0.000	0.000	-4.443	1.541	5.501	-3.612	-7.501
TR.2 UN-A1	Gegham 1	0.000	0.000	0.000	1.000	0.000	0.000	0.000	0.000	0.000	0.000	0.000	0.000	0.000	0.000	-1.536	-11.241	3.021	-4.121	1.768
TR.2 UN-A1	Gegham 1	0.000	0.000	0.000	1.000	0.000	0.000	0.000	0.000	0.000	0.000	0.000	0.000	0.000	0.000	-1.173	-11.265	2.744	-4.273	2.740
TR.2 UN-A1	Syunik	0.000	0.000	0.000	0.000	0.000	0.000	0.000	0.000	0.000	0.000	0.000	0.000	1.000	0.000	-6.894	-11.818	-1.366	3.514	-1.324
TR.2 UN-A1	Kelbadjar	0.000	0.000	0.000	0.000	0.000	0.000	0.000	0.000	1.000	0.000	0.000	0.000	0.000	0.000	-8.358	-9.888	1.517	3.500	-2.014
TR.2 UN-A1	Gegham 1	0.000	0.000	0.000	1.000	0.000	0.000	0.000	0.000	0.000	0.000	0.000	0.000	0.000	0.000	-1.782	-11.372	3.219	-4.684	5.853
TR.2 UN-A1	Kelbadjar	0.000	0.000	0.000	0.000	0.000	0.000	0.000	0.000	1.000	0.000	0.000	0.000	0.000	0.000	-10.483	-8.662	-0.060	4.582	-1.923
TR.2 UN-A1	Kelbadjar	0.000	0.000	0.000	0.000	0.000	0.000	0.000	0.000	1.000	0.000	0.000	0.000	0.000	0.000	-9.144	-8.297	0.798	3.255	-1.802
Tr-2 B1 H4 S	Gegham 1	0.000	0.000	0.000	1.000	0.000	0.000	0.000	0.000	0.000	0.000	0.000	0.000	0.000	0.000	-1.171	-11.059	2.899	-4.442	4.331
Tr-2 B1 H4 S	Hatis	0.000	0.000	0.000	0.000	0.000	0.000	1.000	0.000	0.000	0.000	0.000	0.000	0.000	0.000	19.292	4.756	-4.723	-3.630	-2.134
Tr-2 B1 H4 S	Gegham 1	0.000	0.000	0.000	1.000	0.000	0.000	0.000	0.000	0.000	0.000	0.000	0.000	0.000	0.000	-1.068	-10.330	3.185	-4.647	3.683
Tr-2 B1 H4 S	Gegham 1	0.000	0.000	0.000	1.000	0.000	0.000	0.000	0.000	0.000	0.000	0.000	0.000	0.000	0.000	-0.226	-12.262	2.090	-4.632	4.869
Tr-2 B1 H4 S	Gegham 1	0.000	0.000	0.000	1.000	0.000	0.000	0.000	0.000	0.000	0.000	0.000	0.000	0.000	0.000	-1.085	-11.597	2.758	-4.525	4.061
Tr-2 B1 H4 S	Syunik	0.000	0.000	0.000	0.000	0.000	0.000	0.000	0.000	0.000	0.000	0.000	0.000	1.000	0.000	-6.242	-12.122	-1.235	4.029	-1.976
Tr-2 B1 H4 S	Gegham 1	0.000	0.000	0.000	1.000	0.000	0.000	0.000	0.000	0.000	0.000	0.000	0.000	0.000	0.000	-0.615	-10.892	3.403	-3.745	2.606
Tr-2 B1 H4 S	Arteni	0.000	1.000	0.000	0.000	0.000	0.000	0.000	0.000	0.000	0.000	0.000	0.000	0.000	0.000	-2.300	0.292	4.100	-2.894	-5.063

Tr-2 B1 H4 S	Gegham 1	0.000	0.000	0.000	1.000	0.000	0.000	0.000	0.000	0.000	0.000	0.000	0.000	0.000	0.000	-2.201	-13.056	2.723	-4.397	5.658
Tr-2 B1 H4 S	Gegham 1	0.000	0.000	0.000	1.000	0.000	0.000	0.000	0.000	0.000	0.000	0.000	0.000	0.000	0.000	-0.343	-11.850	3.302	-5.087	6.678
TR2 UN.C0 H	Gegham 1	0.000	0.000	0.000	1.000	0.000	0.000	0.000	0.000	0.000	0.000	0.000	0.000	0.000	0.000	-0.795	-11.683	3.273	-4.302	2.280
TR2 UN.C0 H	Gegham 1	0.000	0.000	0.000	1.000	0.000	0.000	0.000	0.000	0.000	0.000	0.000	0.000	0.000	0.000	-0.593	-12.427	2.236	-4.342	4.361
TR2 UN.C0 H	Gegham 1	0.000	0.000	0.000	1.000	0.000	0.000	0.000	0.000	0.000	0.000	0.000	0.000	0.000	0.000	-1.200	-12.420	2.128	-4.234	2.760
TR2 UN.C0 H	Gegham 1	0.000	0.000	0.000	1.000	0.000	0.000	0.000	0.000	0.000	0.000	0.000	0.000	0.000	0.000	-0.919	-12.414	2.305	-4.394	3.469
TR2 UN.C0 H	Syunik	0.000	0.000	0.000	0.000	0.000	0.000	0.000	0.000	0.000	0.000	0.000	0.000	1.000	0.000	-7.145	-13.089	-2.695	3.591	-0.887
TR2 UN.C0 H	Gegham 1	0.000	0.000	0.000	1.000	0.000	0.000	0.000	0.000	0.000	0.000	0.000	0.000	0.000	0.000	-0.963	-10.772	3.882	-4.535	5.359
TR2 UN.C0 H	Kelbadjar	0.000	0.000	0.000	0.000	0.000	0.000	0.000	0.000	1.000	0.000	0.000	0.000	0.000	0.000	-9.963	-9.322	-1.035	5.335	-1.954
TR2 UN.C0 H	Syunik	0.000	0.000	0.000	0.000	0.000	0.000	0.000	0.000	0.000	0.000	0.000	0.000	1.000	0.000	-5.550	-11.059	-1.565	4.161	-1.203
TR2 UN.C0 H	Gegham 1	0.000	0.000	0.000	1.000	0.000	0.000	0.000	0.000	0.000	0.000	0.000	0.000	0.000	0.000	-0.705	-10.745	2.966	-4.358	3.155
TR2 UN.C0 H	Kelbadjar	0.000	0.000	0.000	0.000	0.000	0.000	0.000	0.000	1.000	0.000	0.000	0.000	0.000	0.000	-8.916	-8.010	2.104	3.274	-2.031
TR2 UN.C0 H	Gegham 1	0.000	0.000	0.000	1.000	0.000	0.000	0.000	0.000	0.000	0.000	0.000	0.000	0.000	0.000	0.686	-9.424	3.712	-5.016	6.398
TR2 UN.C0 H	Kelbadjar	0.000	0.000	0.000	0.000	0.000	0.000	0.000	0.000	1.000	0.000	0.000	0.000	0.000	0.000	-8.726	-9.483	1.551	3.317	-1.611
TR2 UN.C0 H	Gegham 1	0.000	0.000	0.000	1.000	0.000	0.000	0.000	0.000	0.000	0.000	0.000	0.000	0.000	0.000	-0.634	-11.599	3.317	-4.928	6.231
TR2 UN.C0 H	Gegham 1	0.000	0.000	0.000	1.000	0.000	0.000	0.000	0.000	0.000	0.000	0.000	0.000	0.000	0.000	0.082	-9.186	4.546	-5.100	4.179
TR2 UN.C0 H	Gegham 1	0.000	0.000	0.000	1.000	0.000	0.000	0.000	0.000	0.000	0.000	0.000	0.000	0.000	0.000	-0.655	-13.035	3.278	-4.714	4.226
TR2 UN.C0 H	Gegham 1	0.000	0.000	0.000	1.000	0.000	0.000	0.000	0.000	0.000	0.000	0.000	0.000	0.000	0.000	1.356	-8.636	5.059	-5.422	5.546
TR2 UN.C0 H	Gegham 1	0.000	0.000	0.000	1.000	0.000	0.000	0.000	0.000	0.000	0.000	0.000	0.000	0.000	0.000	-0.304	-11.707	3.460	-4.964	5.451
TR2 UN.C0 H	Syunik	0.000	0.000	0.000	0.000	0.000	0.000	0.000	0.000	0.000	0.000	0.000	0.000	1.000	0.000	-12.561	-10.336	-4.229	10.425	-4.266
TR.2 UN-A1	Gegham 1	0.000	0.000	0.000	1.000	0.000	0.000	0.000	0.000	0.000	0.000	0.000	0.000	0.000	0.000	-0.709	-11.669	2.769	-5.001	5.787
TR.2 UN-A1	Gegham 1	0.000	0.000	0.000	1.000	0.000	0.000	0.000	0.000	0.000	0.000	0.000	0.000	0.000	0.000	-0.718	-11.067	2.953	-4.124	1.857
TR.2 UN-A1	Gegham 1	0.000	0.000	0.000	1.000	0.000	0.000	0.000	0.000	0.000	0.000	0.000	0.000	0.000	0.000	-0.764	-11.191	4.207	-4.188	2.575
TR.2 UN-A1	Gegham 1	0.000	0.000	0.000	1.000	0.000	0.000	0.000	0.000	0.000	0.000	0.000	0.000	0.000	0.000	-0.654	-11.183	4.198	-4.832	2.982
TR.2 UN-A1	Syunik	0.000	0.000	0.000	0.000	0.000	0.000	0.000	0.000	0.000	0.000	0.000	0.000	1.000	0.000	-7.076	-12.696	-3.290	3.420	-0.267
TR.2 UN-A1	Gegham 1	0.000	0.000	0.000	1.000	0.000	0.000	0.000	0.000	0.000	0.000	0.000	0.000	0.000	0.000	-0.626	-10.342	4.390	-5.408	5.714
TR.2 UN-A1	Gegham 1	0.000	0.000	0.000	1.000	0.000	0.000	0.000	0.000	0.000	0.000	0.000	0.000	0.000	0.000	-0.590	-10.615	3.061	-4.659	4.925
TR.2 UN-A1	Gegham 1	0.000	0.000	0.000	1.000	0.000	0.000	0.000	0.000	0.000	0.000	0.000	0.000	0.000	0.000	-0.545	-11.245	3.217	-4.143	2.690
TR.2 UN-A1	Kelbadjar	0.000	0.000	0.000	0.000	0.000	0.000	0.000	0.000	1.000	0.000	0.000	0.000	0.000	0.000	-8.827	-9.144	1.868	3.775	-1.576
TR.2 UN-A1	Gegham 1	0.000	0.000	0.000	1.000	0.000	0.000	0.000	0.000	0.000	0.000	0.000	0.000	0.000	0.000	-2.102	-8.052	6.727	-5.574	9.723
TR.2 UN-B1	Kelbadjar	0.000	0.000	0.000	0.000	0.000	0.000	0.000	0.000	1.000	0.000	0.000	0.000	0.000	0.000	-6.603	-9.798	0.640	2.545	-0.957
TR.2 UN-B1	Gegham 1	0.000	0.000	0.000	1.000	0.000	0.000	0.000	0.000	0.000	0.000	0.000	0.000	0.000	0.000	0.005	-11.813	3.190	-4.547	5.709
TR.2 UN-B1	Kelbadjar	0.000	0.000	0.000	0.000	0.000	0.000	0.000	0.000	1.000	0.000	0.000	0.000	0.000	0.000	-7.177	-8.966	0.557	3.262	-1.169
TR.2 UN-B1	Kelbadjar	0.000	0.000	0.000	0.000	0.000	0.000	0.000	0.000	1.000	0.000	0.000	0.000	0.000	0.000	-7.895	-9.422	0.390	5.383	-2.136
TR.2 UN-B1	Gegham 1	0.000	0.000	0.000	1.000	0.000	0.000	0.000	0.000	0.000	0.000	0.000	0.000	0.000	0.000	-1.101	-10.700	2.047	-4.334	3.955
TR.2 UN-B1	Kelbadjar	0.000	0.000	0.000	0.000	0.000	0.000	0.000	0.000	1.000	0.000	0.000	0.000	0.000	0.000	-9.938	-10.008	2.138	4.119	-1.610
TR.2 UN-B1	Kelbadjar	0.000	0.000	0.000	0.000	0.000	0.000	0.000	0.000	1.000	0.000	0.000	0.000	0.000	0.000	-9.594	-9.050	0.367	3.840	-1.412
TR.2 UN-B1	Kelbadjar	0.000	0.000	0.000	0.000	0.000	0.000	0.000	0.000	1.000	0.000	0.000	0.000	0.000	0.000	-9.430	-10.146	0.267	5.393	-1.923
TR.2 UN-B1	Gegham 1	0.000	0.000	0.000	1.000	0.000	0.000	0.000	0.000	0.000	0.000	0.000	0.000	0.000	0.000	0.271	-10.707	3.451	-4.813	4.818
TR.2 UN-B1	Syunik	0.000	0.000	0.000	0.000	0.000	0.000	0.000	0.000	0.024	0.000	0.000	0.000	0.976	0.000	-8.402	-11.232	-0.060	6.247	-1.485
TR.2 UN-B1	Gegham 1	0.000	0.000	0.000	1.000	0.000	0.000	0.000	0.000	0.000	0.000	0.000	0.000	0.000	0.000	-1.556	-12.193	2.481	-4.560	6.999
TR.2 UN-B1	Gegham 1	0.000	0.000	0.000	1.000	0.000	0.000	0.000	0.000	0.000	0.000	0.000	0.000	0.000	0.000	0.495	-10.153	3.012	-4.855	3.938
TR.2 UN-B1	Syunik	0.000	0.000	0.000	0.000	0.000	0.000	0.000	0.000	0.000	0.000	0.000	0.000	1.000	0.000	-6.568	-12.119	-2.934	3.421	-1.108
TR.2 UN-B1	Gegham 1	0.000	0.000	0.000	1.000	0.000	0.000	0.000	0.000	0.000	0.000	0.000	0.000	0.000	0.000	-1.169	-10.994	2.470	-4.045	4.552
TR.2 UN-B1	Gegham 1	0.000	0.000	0.000	1.000	0.000	0.000	0.000	0.000	0.000	0.000	0.000	0.000	0.000	0.000	-0.590	-9.924	4.203	-4.607	5.544
TR.2 UN-B1	Gegham 1	0.000	0.000	0.000	1.000	0.000	0.000	0.000	0.000	0.000	0.000	0.000	0.000	0.000	0.000	-0.797	-9.270	3.719	-4.422	3.449
TR.2 UN-B1	Gegham 1	0.000	0.000	0.000	1.000	0.000	0.000	0.000	0.000	0.000	0.000	0.000	0.000	0.000	0.000	-1.014	-12.244	2.734	-4.868	5.391
TR.2 UN-B1	Gegham 1	0.000	0.000	0.000	1.000	0.000	0.000	0.000	0.000	0.000	0.000	0.000	0.000	0.000	0.000	-0.604	-9.967	3.502	-4.165	2.580
TR.2 UN-B1	Gegham 1	0.000	0.000	0.000	1.000	0.000	0.000	0.000	0.000	0.000	0.000	0.000	0.000	0.000	0.000	-1.880	-10.236	3.298	-4.087	5.690
TR.2 UN-B1	Gegham 1	0.000	0.000	0.000	1.000	0.000	0.000	0.000	0.000	0.000	0.000	0.000	0.000	0.000	0.000	-0.175	-10.381	4.153	-5.012	4.577
TR.2 UN-B1	Gegham 1	0.000	0.000	0.000	1.000	0.000	0.000	0.000	0.000	0.000	0.000	0.000	0.000	0.000	0.000	1.675	-9.861	4.257	-4.651	6.913
TR.2 UN-B1	Gegham 1	0.000	0.000	0.000	1.000	0.000	0.000	0.000	0.000	0.000	0.000	0.000	0.000	0.000	0.000	0.680	-8.386	4.984	-5.283	5.719
TR.2 UN-B1	Gegham 1	0.000	0.000	0.000	1.000	0.000	0.000	0.000	0.000	0.000	0.000	0.000	0.000	0.000	0.000	-0.111	-10.967	3.637	-4.772	3.158
TR.2 UN-B1	Gegham 1	0.000	0.000	0.000	1.000	0.000	0.000	0.000	0.000	0.000	0.000	0.000	0.000	0.000	0.000	-0.221	-9.407	4.848	-5.520	7.031
TR.2 UN-B1	Gegham 1	0.000	0.000	0.000	1.000	0.000	0.000	0.000	0.000	0.000	0.000	0.000	0.000	0.000	0.000	-1.064	-10.959	3.038	-4.846	4.821
TR.2 UN-B1	Kelbadjar	0.000	0.000	0.000	0.000	0.000	0.000	0.000	0.000	1.000	0.000	0.000	0.000	0.000	0.000	-8.377	-7.201	0.611	2.790	-1.422
TR.2 UN-B1	Gegham 1	0.000	0.000	0.000	1.000	0.000	0.000	0.000	0.000	0.000	0.000	0.000	0.000	0.000	0.000	-0.942	-9.940	3.506	-4.947	5.242
TR.2 UN-B1	Gegham 2	0.000	0.004	0.000	0.000	0.996	0.000	0.000	0.000	0.000	0.000	0.000	0.000	0.000	0.000	-0.780	4.885	5.429	-1.869	-4.953
TR.2 UN-B1	Gegham 1	0.000	0.000	0.000	1.000	0.000	0.000	0.000	0.000	0.000	0.000	0.000	0.000	0.000	0.000	3.474	-10.710	4.064	-4.513	5.846
Tr2 TS-H1Sp	Kelbadjar	0.000	0.000	0.000	0.000	0.000	0.000	0.000	0.000	1.000	0.000	0.000	0.000	0.000	0.000	-10.008	-9.983	-0.671	4.650	-1.868
Tr2 TS-H1Sp	Gegham 1	0.000	0.000	0.000	1.000	0.000	0.000	0.000	0.000	0.000	0.000	0.000	0.000	0.000	0.000	-1.327	-12.168	3.105	-4.312	3.117
Tr2 TS-H1Sp	Gegham 1	0.000	0.000	0.000	1.000	0.000	0.000	0.000	0.000	0.000	0.000	0.000	0.000	0.000	0.000	-0.287	-10.651	3.571	-4.559	3.472
Tr2 TS-H1Sp	Gegham 1	0.000	0.000	0.000	1.000	0.000	0.000	0.000	0.000	0.000	0.000	0.000	0.000	0.000	0.000	-0.671	-10.982	2.897	-3.780	4.145
Tr2 TS-H1Sp	Gegham 1	0.000	0.000	0.000	1.000	0.000	0.000	0.000	0.000	0.000	0.000	0.000	0.000	0.000	0.000	0.009	-10.125	3.167	-4.976	5.803
Tr2 TS-H1Sp	Gegham 1	0.000	0.000	0.000	1.000	0.000	0.000	0.000	0.000	0.000	0.000	0.000	0.000	0.000	0.000	-0.954	-12.833	2.891	-4.648	6.988
Tr2 TS-H1Sp	Gegham 1	0.000	0.000	0.000	1.000	0.000	0.000	0.000	0.000	0.000	0.000	0.000	0.000	0.000	0.000	-1.492	-11.208	3.144	-3.610	2.337
Tr2 TS-H1Sp	Kelbadjar	0.000	0.000	0.000	0.000	0.000	0.000	0.000	0.000	1.000	0.000	0.000	0.000	0.000	0.000	-8.908	-8.806	2.287	3.035	-1.555

Tr2 TS-H1Sp	Kelbadjar	0.000	0.000	0.000	0.000	0.000	0.000	0.000	0.000	1.000	0.000	0.000	0.000	0.000	0.000	-10.337	-9.554	2.136	4.549	-2.091
Tr2 TS-H1Sp	Gegham 1	0.000	0.000	0.000	1.000	0.000	0.000	0.000	0.000	0.000	0.000	0.000	0.000	0.000	0.000	-0.540	-10.531	3.517	-4.730	2.544
Tr2 TS-H1Sp	Gegham 1	0.000	0.000	0.000	1.000	0.000	0.000	0.000	0.000	0.000	0.000	0.000	0.000	0.000	0.000	-1.849	-10.620	3.711	-4.422	5.029
Tr2 TS-H1Sp	Syunik	0.000	0.000	0.000	0.000	0.000	0.000	0.000	0.000	0.000	0.000	0.000	0.000	1.000	0.000	-7.195	-12.983	-1.816	3.673	-1.373
Tr2 TS-H1Sp	Gegham 1	0.000	0.000	0.000	1.000	0.000	0.000	0.000	0.000	0.000	0.000	0.000	0.000	0.000	0.000	0.566	-8.829	4.135	-5.097	4.916
Tr2 TS-H1Sp	Gegham 1	0.000	0.000	0.000	1.000	0.000	0.000	0.000	0.000	0.000	0.000	0.000	0.000	0.000	0.000	6.044	-9.917	1.962	-4.751	7.382
Tr2 TS-H1Sp	Gegham 1	0.000	0.000	0.000	1.000	0.000	0.000	0.000	0.000	0.000	0.000	0.000	0.000	0.000	0.000	-0.494	-10.942	3.426	-4.965	3.850
Tr2 TS-H1Sp	Kelbadjar	0.000	0.000	0.000	0.000	0.000	0.000	0.000	0.000	1.000	0.000	0.000	0.000	0.000	0.000	-7.357	-8.766	0.061	3.439	-1.320
Tr2 TS-H1Sp	Gegham 1	0.000	0.000	0.000	1.000	0.000	0.000	0.000	0.000	0.000	0.000	0.000	0.000	0.000	0.000	-1.345	-11.291	3.135	-4.822	5.180
Tr2 TS-H1Sp	Kelbadjar	0.000	0.000	0.000	0.000	0.000	0.000	0.000	0.000	1.000	0.000	0.000	0.000	0.000	0.000	-10.733	-11.209	0.528	5.080	-1.992
Tr2 TS-H1Sp	Gegham 1	0.000	0.000	0.000	1.000	0.000	0.000	0.000	0.000	0.000	0.000	0.000	0.000	0.000	0.000	-0.455	-12.222	2.331	-5.081	7.117
Tr2 TS-H1Sp	Kelbadjar	0.000	0.000	0.000	0.000	0.000	0.000	0.000	0.000	1.000	0.000	0.000	0.000	0.000	0.000	-8.736	-8.314	-0.012	2.929	-1.690
Tr2 TS-H1Sp	Gegham 1	0.000	0.000	0.000	1.000	0.000	0.000	0.000	0.000	0.000	0.000	0.000	0.000	0.000	0.000	-1.446	-11.772	1.986	-3.770	3.883
Tr2 TS-H1Sp	Gegham 1	0.000	0.000	0.000	1.000	0.000	0.000	0.000	0.000	0.000	0.000	0.000	0.000	0.000	0.000	-1.210	-11.401	2.777	-4.193	3.436
Tr2 TS-H1Sp	Kelbadjar	0.000	0.000	0.000	0.000	0.000	0.000	0.000	0.000	1.000	0.000	0.000	0.000	0.000	0.000	-8.964	-8.415	1.291	3.656	-1.727
Tr2 TS-H1Sp	Kelbadjar	0.000	0.000	0.000	0.000	0.000	0.000	0.000	0.000	1.000	0.000	0.000	0.000	0.000	0.000	-9.405	-9.403	-0.392	3.959	-1.372
Tr2 TS-H1Sp	Gegham 1	0.000	0.000	0.000	1.000	0.000	0.000	0.000	0.000	0.000	0.000	0.000	0.000	0.000	0.000	0.334	-10.387	3.249	-4.883	5.081
Tr2 TS-H1Sp	Gegham 1	0.000	0.000	0.000	1.000	0.000	0.000	0.000	0.000	0.000	0.000	0.000	0.000	0.000	0.000	-1.330	-11.287	3.473	-3.705	1.134
Tr2 TS-H1Sp	Gegham 1	0.000	0.000	0.000	1.000	0.000	0.000	0.000	0.000	0.000	0.000	0.000	0.000	0.000	0.000	-0.189	-10.140	3.275	-4.525	3.502
Tr2 TS-H1Sp	Gegham 1	0.000	0.000	0.000	1.000	0.000	0.000	0.000	0.000	0.000	0.000	0.000	0.000	0.000	0.000	-1.043	-11.333	4.155	-4.886	3.653
Tr2 TS-H1Sp	Gegham 1	0.000	0.000	0.000	1.000	0.000	0.000	0.000	0.000	0.000	0.000	0.000	0.000	0.000	0.000	-0.969	-10.861	3.763	-4.423	3.011
Tr2 TS-H1Sp	Kelbadjar	0.000	0.000	0.000	0.000	0.000	0.000	0.000	0.000	1.000	0.000	0.000	0.000	0.000	0.000	-9.394	-9.366	0.219	4.713	-1.840
Tr2 TS-H1Sp	Gegham 1	0.000	0.000	0.000	1.000	0.000	0.000	0.000	0.000	0.000	0.000	0.000	0.000	0.000	0.000	-1.891	-11.009	4.291	-5.320	8.078
Tr2 TS-H1Sp	Syunik	0.000	0.000	0.000	0.000	0.000	0.000	0.000	0.000	0.000	0.000	0.000	0.000	1.000	0.000	-7.808	-12.672	-2.807	4.330	-1.181
Tr2 TS-H1Sp	Gegham 1	0.000	0.000	0.000	1.000	0.000	0.000	0.000	0.000	0.000	0.000	0.000	0.000	0.000	0.000	-0.217	-10.786	2.947	-4.293	4.161
Tr2 TS-H1Sp	Gegham 1	0.000	0.000	0.000	1.000	0.000	0.000	0.000	0.000	0.000	0.000	0.000	0.000	0.000	0.000	-1.489	-11.927	3.236	-4.563	3.626
Tr2 TS-H1Sp	Gegham 1	0.000	0.000	0.000	1.000	0.000	0.000	0.000	0.000	0.000	0.000	0.000	0.000	0.000	0.000	-0.589	-11.127	3.688	-4.909	3.279
Tr2 TS-H1Sp	Syunik	0.000	0.000	0.000	0.000	0.000	0.000	0.000	0.000	0.000	0.000	0.000	0.000	1.000	0.000	-7.245	-13.037	-0.911	4.226	-1.521
Tr2 TS-H1Sp	Gegham 1	0.000	0.000	0.000	1.000	0.000	0.000	0.000	0.000	0.000	0.000	0.000	0.000	0.000	0.000	-0.256	-9.338	3.272	-4.863	4.909
Tr2 TS-H1Sp	Gegham 1	0.000	0.000	0.000	1.000	0.000	0.000	0.000	0.000	0.000	0.000	0.000	0.000	0.000	0.000	-1.061	-8.277	4.372	-4.231	2.649
Tr2 TS-H1Sp	Syunik	0.000	0.000	0.000	0.000	0.000	0.000	0.000	0.000	0.000	0.000	0.000	0.000	1.000	0.000	-2.382	-12.674	-4.235	4.166	-0.161
Tr2 TS-H1Sp	Gegham 1	0.000	0.000	0.000	1.000	0.000	0.000	0.000	0.000	0.000	0.000	0.000	0.000	0.000	0.000	0.080	-11.590	2.862	-4.764	4.563
Tr2 TS-H1Sp	Gegham 1	0.000	0.000	0.000	1.000	0.000	0.000	0.000	0.000	0.000	0.000	0.000	0.000	0.000	0.000	-0.300	-9.115	3.601	-4.629	2.919
Tr2 TS-H1Sp	Gegham 1	0.000	0.000	0.000	1.000	0.000	0.000	0.000	0.000	0.000	0.000	0.000	0.000	0.000	0.000	0.339	-10.953	3.501	-5.566	5.453
Tr2 TS-H1Sp	Gegham 1	0.000	0.000	0.000	1.000	0.000	0.000	0.000	0.000	0.000	0.000	0.000	0.000	0.000	0.000	-0.509	-10.339	3.781	-4.373	5.319
Tr2 TS-H1Sp	Gegham 1	0.000	0.000	0.000	1.000	0.000	0.000	0.000	0.000	0.000	0.000	0.000	0.000	0.000	0.000	-0.730	-10.603	3.404	-4.388	3.777
Tr2 TS-H1Sp	Gegham 1	0.000	0.000	0.000	1.000	0.000	0.000	0.000	0.000	0.000	0.000	0.000	0.000	0.000	0.000	-1.544	-10.371	3.313	-4.639	5.210
Tr2 TS-H1Sp	Kelbadjar	0.000	0.000	0.000	0.000	0.000	0.000	0.000	0.000	1.000	0.000	0.000	0.000	0.000	0.000	-9.805	-9.399	-0.931	4.224	-1.405
Tr2 TS-H1Sp	Gegham 1	0.000	0.000	0.000	1.000	0.000	0.000	0.000	0.000	0.000	0.000	0.000	0.000	0.000	0.000	-0.934	-10.916	3.263	-4.747	5.014
Tr2 TS-H1Sp	Gegham 1	0.000	0.000	0.000	1.000	0.000	0.000	0.000	0.000	0.000	0.000	0.000	0.000	0.000	0.000	0.231	-10.668	3.540	-4.675	3.361
Tr-2 B1 H4 S	Gegham 1	0.000	0.000	0.000	1.000	0.000	0.000	0.000	0.000	0.000	0.000	0.000	0.000	0.000	0.000	-1.187	-10.933	3.180	-4.553	3.261
Tr-2 B1 H4 S	Gegham 1	0.000	0.000	0.000	1.000	0.000	0.000	0.000	0.000	0.000	0.000	0.000	0.000	0.000	0.000	-2.243	-12.411	2.861	-4.243	4.361
Tr-2 B1 H4 S	Gegham 1	0.000	0.000	0.000	1.000	0.000	0.000	0.000	0.000	0.000	0.000	0.000	0.000	0.000	0.000	0.398	-11.354	0.959	-4.416	7.788
Tr-2 B1 H4 S	Gegham 1	0.000	0.000	0.000	1.000	0.000	0.000	0.000	0.000	0.000	0.000	0.000	0.000	0.000	0.000	-0.953	-11.341	2.289	-4.180	3.383
Tr-2 B1 H4 S	Gegham 1	0.000	0.000	0.000	1.000	0.000	0.000	0.000	0.000	0.000	0.000	0.000	0.000	0.000	0.000	-0.785	-9.529	3.698	-4.344	2.493
Tr-2 B1 H4 S	Gegham 1	0.000	0.000	0.000	1.000	0.000	0.000	0.000	0.000	0.000	0.000	0.000	0.000	0.000	0.000	-0.497	-10.844	3.103	-4.798	2.474
Tr-2 B1 H4 S	Gegham 1	0.000	0.000	0.000	1.000	0.000	0.000	0.000	0.000	0.000	0.000	0.000	0.000	0.000	0.000	0.868	-9.853	1.424	-3.670	4.571
Tr-2 B1 H4 S	Gegham 1	0.000	0.000	0.000	1.000	0.000	0.000	0.000	0.000	0.000	0.000	0.000	0.000	0.000	0.000	-2.098	-9.801	5.046	-4.744	6.544
Tr-2 B1 H4 S	Gegham 1	0.000	0.000	0.000	1.000	0.000	0.000	0.000	0.000	0.000	0.000	0.000	0.000	0.000	0.000	-1.268	-12.042	2.728	-4.593	3.494
Tr-2 B1 H4 S	Gegham 1	0.000	0.000	0.000	1.000	0.000	0.000	0.000	0.000	0.000	0.000	0.000	0.000	0.000	0.000	-1.353	-11.681	2.813	-4.145	1.646
Tr-2 B1 H4 S	Gegham 1	0.000	0.000	0.000	1.000	0.000	0.000	0.000	0.000	0.000	0.000	0.000	0.000	0.000	0.000	-2.350	-9.756	3.365	-4.028	2.925
Tr-2 B1 H4 S	Syunik	0.000	0.000	0.000	0.000	0.000	0.000	0.000	0.000	0.000	0.000	0.000	0.000	1.000	0.000	-8.701	-16.039	-2.885	2.889	-0.650
Tr-2 B1 H4 S	Gegham 1	0.000	0.000	0.000	1.000	0.000	0.000	0.000	0.000	0.000	0.000	0.000	0.000	0.000	0.000	-0.577	-10.886	3.110	-4.253	4.056
Tr-2 B1 H4 S	Gegham 1	0.000	0.000	0.000	1.000	0.000	0.000	0.000	0.000	0.000	0.000	0.000	0.000	0.000	0.000	0.390	-9.020	4.053	-4.758	7.435
Tr-2 B1 H4 S	Gegham 1	0.000	0.000	0.000	1.000	0.000	0.000	0.000	0.000	0.000	0.000	0.000	0.000	0.000	0.000	-1.474	-10.806	2.889	-4.509	4.212
Tr-2 B1 H4 S	Gegham 1	0.000	0.000	0.000	1.000	0.000	0.000	0.000	0.000	0.000	0.000	0.000	0.000	0.000	0.000	-0.001	-8.606	4.240	-5.109	7.254
T2 A1-C1 Ho	Gegham 1	0.000	0.000	0.000	1.000	0.000	0.000	0.000	0.000	0.000	0.000	0.000	0.000	0.000	0.000	0.357	-8.893	4.144	-4.543	2.962
T2 A1-C1 Ho	Kelbadjar	0.000	0.000	0.000	0.000	0.000	0.000	0.000	0.000	1.000	0.000	0.000	0.000	0.000	0.000	-9.429	-8.267	-0.356	3.882	-1.317
T2 A1-C1 Ho	Gegham 1	0.000	0.000	0.000	1.000	0.000	0.000	0.000	0.000	0.000	0.000	0.000	0.000	0.000	0.000	-0.375	-9.130	4.412	-5.270	6.077
T2 A1-C1 Ho	Gegham 1	0.000	0.000	0.000	1.000	0.000	0.000	0.000	0.000	0.000	0.000	0.000	0.000	0.000	0.000	-0.879	-10.036	3.868	-4.895	7.296
T2 A1-C1 Ho	Gegham 1	0.000	0.000	0.000	1.000	0.000	0.000	0.000	0.000	0.000	0.000	0.000	0.000	0.000	0.000	-1.836	-12.071	2.451	-4.272	4.004
Tr-2 A0-C0 H	Gegham 1	0.000	0.000	0.000	1.000	0.000	0.000	0.000	0.000	0.000	0.000	0.000	0.000	0.000	0.000	2.276	-9.877	3.145	-4.446	3.831
Tr-2 A1-C1 H	Kelbadjar	0.000	0.000	0.000	0.000	0.000	0.000	0.000	0.000	1.000	0.000	0.000	0.000	0.000	0.000	-9.643	-8.718	-0.047	4.429	-1.794
Tr-2 A1-C1 H	Kelbadjar	0.000	0.000	0.000	0.000	0.000	0.000	0.000	0.000	1.000	0.000	0.000	0.000	0.000	0.000	-10.005	-9.343	-1.286	5.359	-2.012
Tr-2 B0 H4 S	Gegham 1	0.000	0.000	0.000	1.000	0.000	0.000	0.000	0.000	0.000	0.000	0.000	0.000	0.000	0.000	-1.214	-11.462	3.939	-4.229	2.385
Tr-2 B0 H4 S	Gegham 1	0.000	0.000	0.000	1.000	0.000	0.000	0.000	0.000	0.000	0.000	0.000	0.000	0.000	0.000	-1.393	-12.264	2.612	-4.261	3.391
Tr-2 B0 H4 S	Gegham 1	0.000	0.000	0.000	1.000	0.000	0.000	0.000	0.000	0.000	0.000	0.000	0.000	0.000	0.000	-0.587	-10.719	2.445	-4.281	3.640

Tr-2 A1 H4 S	Gegham 1	0.000	0.000	0.000	1.000	0.000	0.000	0.000	0.000	0.000	0.000	0.000	0.000	0.000	0.000	-0.892	-10.861	3.322	-4.631	2.936
Tr-2 A1 H4 S	Gegham 1	0.000	0.000	0.000	1.000	0.000	0.000	0.000	0.000	0.000	0.000	0.000	0.000	0.000	0.000	-1.268	-11.173	2.642	-4.487	3.204
Tr-2 A1 H4 S	Gegham 1	0.000	0.000	0.000	1.000	0.000	0.000	0.000	0.000	0.000	0.000	0.000	0.000	0.000	0.000	-0.593	-10.836	3.332	-4.479	3.306
Tr-2 A1 H4 S	Gegham 1	0.000	0.000	0.000	1.000	0.000	0.000	0.000	0.000	0.000	0.000	0.000	0.000	0.000	0.000	-0.076	-11.551	2.995	-4.658	4.097
Tr-2 A1 H4 S	Gegham 1	0.000	0.000	0.000	1.000	0.000	0.000	0.000	0.000	0.000	0.000	0.000	0.000	0.000	0.000	-1.299	-11.828	2.940	-4.694	4.910
Tr-2 A1 H4 S	Gegham 1	0.000	0.000	0.000	1.000	0.000	0.000	0.000	0.000	0.000	0.000	0.000	0.000	0.000	0.000	-1.333	-11.476	3.460	-4.700	4.349
Tr-2 A1 H4 S	Gegham 1	0.000	0.000	0.000	1.000	0.000	0.000	0.000	0.000	0.000	0.000	0.000	0.000	0.000	0.000	1.443	-10.574	4.828	-5.629	5.532
Tr H5 Sp1 A5	Gegham 1	0.000	0.000	0.000	1.000	0.000	0.000	0.000	0.000	0.000	0.000	0.000	0.000	0.000	0.000	0.387	-10.143	3.648	-4.873	3.698
Tr-2 H3 Sp1 /	Gegham 1	0.000	0.000	0.000	1.000	0.000	0.000	0.000	0.000	0.000	0.000	0.000	0.000	0.000	0.000	-0.612	-13.211	2.078	-4.243	3.836
Tr-2 H3 Sp1 /	Gegham 1	0.000	0.000	0.000	1.000	0.000	0.000	0.000	0.000	0.000	0.000	0.000	0.000	0.000	0.000	-0.496	-11.983	2.931	-4.230	3.447
TR.2 UN A1-	Kelbadjar	0.000	0.000	0.000	0.000	0.000	0.000	0.000	0.000	1.000	0.000	0.000	0.000	0.000	0.000	-8.469	-9.449	0.802	2.527	-0.958
TR.2 UN A1-	Gegham 1	0.000	0.000	0.000	1.000	0.000	0.000	0.000	0.000	0.000	0.000	0.000	0.000	0.000	0.000	-1.252	-12.462	2.640	-4.531	3.580
TR.2 UN A1-	Gegham 1	0.000	0.000	0.000	1.000	0.000	0.000	0.000	0.000	0.000	0.000	0.000	0.000	0.000	0.000	-0.403	-10.341	3.295	-4.736	3.928
TR.2 UN A1-	Kelbadjar	0.000	0.000	0.000	0.000	0.000	0.000	0.000	0.000	1.000	0.000	0.000	0.000	0.000	0.000	-8.355	-8.269	0.905	2.584	-1.781
TR.2 UN A1-	Gegham 1	0.000	0.000	0.000	1.000	0.000	0.000	0.000	0.000	0.000	0.000	0.000	0.000	0.000	0.000	-0.966	-10.758	3.472	-5.157	7.012
TR.2 UN A1-	Gegham 1	0.000	0.000	0.000	1.000	0.000	0.000	0.000	0.000	0.000	0.000	0.000	0.000	0.000	0.000	0.271	-10.452	3.577	-4.643	5.078
TR.2 UN A1-	Kelbadjar	0.000	0.000	0.000	0.000	0.000	0.000	0.000	0.000	1.000	0.000	0.000	0.000	0.000	0.000	-9.527	-8.973	0.691	5.069	-1.994
TR-2 H3 S2 A	Gegham 1	0.000	0.000	0.000	1.000	0.000	0.000	0.000	0.000	0.000	0.000	0.000	0.000	0.000	0.000	-0.414	-10.994	2.872	-4.246	2.843
TR-2 H2 Sp1	Gegham 1	0.000	0.000	0.000	1.000	0.000	0.000	0.000	0.000	0.000	0.000	0.000	0.000	0.000	0.000	-0.567	-11.357	3.356	-4.756	2.507
TR.2 UN.C1 T	Syunik	0.000	0.000	0.000	0.000	0.000	0.000	0.000	0.000	0.000	0.000	0.000	0.000	1.000	0.000	-7.293	-14.391	-1.723	3.595	-1.193
Tr-2 H4 Sp2 /	Gegham 1	0.000	0.000	0.000	1.000	0.000	0.000	0.000	0.000	0.000	0.000	0.000	0.000	0.000	0.000	-0.430	-10.555	3.449	-4.664	3.187
Tr-2 H4 Sp2 /	Gegham 1	0.000	0.000	0.000	1.000	0.000	0.000	0.000	0.000	0.000	0.000	0.000	0.000	0.000	0.000	0.502	-9.932	4.979	-4.630	3.581
Tr-2 H4 Sp2 /	Gegham 1	0.000	0.000	0.000	1.000	0.000	0.000	0.000	0.000	0.000	0.000	0.000	0.000	0.000	0.000	-0.374	-10.249	3.235	-4.055	3.091
Tr-2 H4 Sp2 /	Gegham 1	0.000	0.000	0.000	1.000	0.000	0.000	0.000	0.000	0.000	0.000	0.000	0.000	0.000	0.000	-0.036	-10.323	4.019	-4.703	4.106
Tr-2 H4 Sp2 /	Gegham 1	0.000	0.000	0.000	1.000	0.000	0.000	0.000	0.000	0.000	0.000	0.000	0.000	0.000	0.000	-0.093	-10.292	3.149	-4.782	4.791
Tr-2 H4 Sp2 /	Gegham 1	0.000	0.000	0.000	1.000	0.000	0.000	0.000	0.000	0.000	0.000	0.000	0.000	0.000	0.000	-1.306	-11.272	2.599	-3.532	4.346
Tr-2 H4 Sp2 /	Gegham 1	0.000	0.000	0.000	1.000	0.000	0.000	0.000	0.000	0.000	0.000	0.000	0.000	0.000	0.000	-0.262	-9.034	4.051	-4.650	5.559
Tr-2 H4 Sp2 /	Gegham 1	0.000	0.000	0.000	1.000	0.000	0.000	0.000	0.000	0.000	0.000	0.000	0.000	0.000	0.000	-0.560	-9.939	3.209	-4.840	6.868
Tr-2 H4 Sp2 /	Gegham 1	0.000	0.000	0.000	1.000	0.000	0.000	0.000	0.000	0.000	0.000	0.000	0.000	0.000	0.000	0.406	-10.811	3.895	-4.758	5.862
TR.2 UN B0 I	Syunik	0.000	0.000	0.000	0.000	0.000	0.000	0.000	0.000	0.000	0.000	0.000	0.000	1.000	0.000	-6.891	-11.648	-1.810	2.714	-1.047
TR.2 B0 H2 S	Gegham 1	0.000	0.000	0.000	1.000	0.000	0.000	0.000	0.000	0.000	0.000	0.000	0.000	0.000	0.000	0.358	-10.289	2.941	-4.751	5.617
TR.2 UN A2-	Kelbadjar	0.000	0.000	0.000	0.000	0.000	0.000	0.000	0.000	1.000	0.000	0.000	0.000	0.000	0.000	-8.384	-8.982	1.604	4.273	-1.682
TR.2 UN A2-	Gegham 1	0.000	0.000	0.000	1.000	0.000	0.000	0.000	0.000	0.000	0.000	0.000	0.000	0.000	0.000	-0.035	-11.288	2.395	-4.628	4.786
TR.2 UN A2-	Arteni	0.000	1.000	0.000	0.000	0.000	0.000	0.000	0.000	0.000	0.000	0.000	0.000	0.000	0.000	-1.103	2.233	3.178	-2.856	-4.621
TR.2 UN A2-	Gegham 1	0.000	0.000	0.000	1.000	0.000	0.000	0.000	0.000	0.000	0.000	0.000	0.000	0.000	0.000	-1.257	-11.339	2.476	-4.367	2.726
TR.2 UN A2-	Gegham 1	0.000	0.000	0.000	1.000	0.000	0.000	0.000	0.000	0.000	0.000	0.000	0.000	0.000	0.000	-0.737	-11.675	2.279	-4.525	6.465
Tr-2 H4 Sp1 (	Gegham 1	0.000	0.000	0.000	1.000	0.000	0.000	0.000	0.000	0.000	0.000	0.000	0.000	0.000	0.000	-0.500	-11.757	2.988	-4.348	2.645
Tr-2 H4 Sp1 (	Gegham 1	0.000	0.000	0.000	1.000	0.000	0.000	0.000	0.000	0.000	0.000	0.000	0.000	0.000	0.000	0.042	-12.952	3.119	-4.996	6.694
Tr-2 H4 Sp1 (	Gegham 1	0.000	0.000	0.000	1.000	0.000	0.000	0.000	0.000	0.000	0.000	0.000	0.000	0.000	0.000	-0.115	-10.784	3.059	-4.304	2.846
Tr-2 H4 Sp1 (	Gegham 1	0.000	0.000	0.000	1.000	0.000	0.000	0.000	0.000	0.000	0.000	0.000	0.000	0.000	0.000	-1.528	-10.712	3.284	-4.650	3.618
Tr-2 H4 Sp1 (	Gegham 1	0.000	0.000	0.000	1.000	0.000	0.000	0.000	0.000	0.000	0.000	0.000	0.000	0.000	0.000	0.412	-10.585	3.231	-4.711	4.770
Tr-2 H4 Sp1 (	Gegham 1	0.000	0.000	0.000	1.000	0.000	0.000	0.000	0.000	0.000	0.000	0.000	0.000	0.000	0.000	-1.249	-11.268	3.805	-4.538	8.780
Tr-2 H4 Sp1 (	Gegham 1	0.000	0.000	0.000	1.000	0.000	0.000	0.000	0.000	0.000	0.000	0.000	0.000	0.000	0.000	-0.715	-10.014	3.968	-4.309	6.457
Tr-2 H4 Sp1 (	Gegham 1	0.000	0.000	0.000	1.000	0.000	0.000	0.000	0.000	0.000	0.000	0.000	0.000	0.000	0.000	-1.457	-12.582	2.993	-4.386	4.014
Tr-2 H4 Sp1 (	Gegham 1	0.000	0.000	0.000	1.000	0.000	0.000	0.000	0.000	0.000	0.000	0.000	0.000	0.000	0.000	-1.290	-11.112	3.517	-4.292	4.915
Tr-2 H4 Sp1 (	Gegham 1	0.000	0.000	0.000	1.000	0.000	0.000	0.000	0.000	0.000	0.000	0.000	0.000	0.000	0.000	-0.360	-11.010	3.625	-4.114	2.566
Tr-2 H4 Sp1 (	Gegham 1	0.000	0.000	0.000	1.000	0.000	0.000	0.000	0.000	0.000	0.000	0.000	0.000	0.000	0.000	-1.375	-11.199	3.328	-5.435	5.819
Tr-2 H4 Sp1 (	Arteni	0.000	1.000	0.000	0.000	0.000	0.000	0.000	0.000	0.000	0.000	0.000	0.000	0.000	0.000	-2.567	1.100	4.787	-3.232	-6.547
Tr-2 H4 Sp1 (	Gegham 1	0.000	0.000	0.000	1.000	0.000	0.000	0.000	0.000	0.000	0.000	0.000	0.000	0.000	0.000	-1.208	-9.977	4.455	-4.854	4.330
Tr-2 H4 Sp1 (	Gegham 1	0.000	0.000	0.000	1.000	0.000	0.000	0.000	0.000	0.000	0.000	0.000	0.000	0.000	0.000	-1.232	-10.661	4.652	-5.434	7.840
Tr-2 H4 Sp1 (	Gegham 1	0.000	0.000	0.000	1.000	0.000	0.000	0.000	0.000	0.000	0.000	0.000	0.000	0.000	0.000	-1.978	-7.457	5.554	-5.103	8.554
Tr-2 H4 Sp2 (	Syunik	0.000	0.000	0.000	0.000	0.000	0.000	0.000	0.000	0.000	0.000	0.000	0.000	1.000	0.000	-8.089	-17.963	-3.568	3.321	-0.912
Tr-2 H4 Sp2 (	Gegham 1	0.000	0.000	0.000	1.000	0.000	0.000	0.000	0.000	0.000	0.000	0.000	0.000	0.000	0.000	-0.895	-12.288	2.823	-4.605	4.347
Tr-2 H4 Sp2 (	Gegham 1	0.000	0.000	0.000	1.000	0.000	0.000	0.000	0.000	0.000	0.000	0.000	0.000	0.000	0.000	-0.999	-9.984	3.226	-4.020	2.184
Tr-2 H4 Sp2 (	Gegham 1	0.000	0.000	0.000	1.000	0.000	0.000	0.000	0.000	0.000	0.000	0.000	0.000	0.000	0.000	-0.447	-11.345	2.368	-4.355	3.974
Tr-2 H4 Sp2 (	Gegham 1	0.000	0.000	0.000	1.000	0.000	0.000	0.000	0.000	0.000	0.000	0.000	0.000	0.000	0.000	-0.361	-10.946	3.147	-4.975	3.105
Tr-2 H4 Sp2 (	Gegham 1	0.000	0.000	0.000	1.000	0.000	0.000	0.000	0.000	0.000	0.000	0.000	0.000	0.000	0.000	0.522	-10.304	2.176	-4.065	3.814
Tr-2 H4 Sp2 (	Gegham 1	0.000	0.000	0.000	1.000	0.000	0.000	0.000	0.000	0.000	0.000	0.000	0.000	0.000	0.000	-0.887	-11.783	3.126	-5.281	7.171
Tr-2 H4 Sp2 (	Khorapor	0.000	0.000	0.000	0.000	0.000	0.000	0.000	0.000	0.000	1.000	0.000	0.000	0.000	0.000	-7.754	-17.472	-3.489	-1.009	3.497
Tr-2 H4 Sp2 (	Gegham 1	0.000	0.000	0.000	1.000	0.000	0.000	0.000	0.000	0.000	0.000	0.000	0.000	0.000	0.000	-0.486	-9.866	3.508	-4.635	6.537
Tr-2 H4 Sp2 (	Gegham 1	0.000	0.000	0.000	1.000	0.000	0.000	0.000	0.000	0.000	0.000	0.000	0.000	0.000	0.000	-1.786	-11.655	3.074	-3.923	3.368
Tr-2 H4 Sp2 (	Gegham 1	0.000	0.000	0.000	1.000	0.000	0.000	0.000	0.000	0.000	0.000	0.000	0.000	0.000	0.000	-3.209	-11.994	3.042	-4.580	8.852
Tr-2 H4 Sp2 (	Gegham 1	0.000	0.000	0.000	1.000	0.000	0.000	0.000	0.000	0.000	0.000	0.000	0.000	0.000	0.000	-0.938	-11.166	3.542	-4.782	7.411
Tr-2 H4 Sp2 (	Gegham 1	0.000	0.000	0.000	1.000	0.000	0.000	0.000	0.000	0.000	0.000	0.000	0.000	0.000	0.000	-0.441	-11.155	3.559	-5.022	5.324
Tr-2 H4 Sp2 (	Gegham 1	0.000	0.000	0.000	1.000	0.000	0.000	0.000	0.000	0.000	0.000	0.000	0.000	0.000	0.000	-0.679	-12.124	2.760	-4.426	3.735
TR.2 A5 H1.S	Gegham 1	0.000	0.000	0.000	1.000	0.000	0.000	0.000	0.000	0.000	0.000	0.000	0.000	0.000	0.000	-0.923	-11.239	2.027	-4.081	2.265
TR.2 A5 H1.S	Gegham 1	0.000	0.000	0.000	1.000	0.000	0.000	0.000	0.000	0.000	0.000	0.000	0.000	0.000	0.000	-0.203	-11.700	3.088	-4.323	3.032

TR.2 A5 H1.S	Gegham 1	0.000	0.000	0.000	1.000	0.000	0.000	0.000	0.000	0.000	0.000	0.000	0.000	0.000	0.000	-1.084	-11.203	3.579	-4.155	2.412
TR.2 A5 H1.S	Kelbadjar	0.000	0.000	0.000	0.000	0.000	0.000	0.000	0.000	1.000	0.000	0.000	0.000	0.000	0.000	-9.309	-9.394	-0.411	4.503	-1.683
TR.2 A5 H1.S	Gegham 1	0.000	0.000	0.000	1.000	0.000	0.000	0.000	0.000	0.000	0.000	0.000	0.000	0.000	0.000	-0.830	-12.603	2.168	-4.350	5.076
TR.2 A5 H1.S	Gegham 1	0.000	0.000	0.000	1.000	0.000	0.000	0.000	0.000	0.000	0.000	0.000	0.000	0.000	0.000	-0.267	-10.868	2.809	-4.850	5.925
TR.2 A5 H1.S	Gegham 1	0.000	0.000	0.000	1.000	0.000	0.000	0.000	0.000	0.000	0.000	0.000	0.000	0.000	0.000	-1.514	-11.683	3.398	-4.431	3.189
T2 A1 to C1 H	Kelbadjar	0.000	0.000	0.000	0.000	0.000	0.000	0.000	0.000	1.000	0.000	0.000	0.000	0.000	0.000	-10.607	-8.944	0.663	4.378	-1.833
T2 A1-C1 H2	Hatis	0.000	0.000	0.000	0.000	0.000	0.000	1.000	0.000	0.000	0.000	0.000	0.000	0.000	0.000	11.827	6.491	4.202	1.157	-3.840
T2 A1-C1 H2	Kelbadjar	0.000	0.000	0.000	0.000	0.000	0.000	0.000	0.000	1.000	0.000	0.000	0.000	0.000	0.000	-10.221	-9.104	-0.664	5.088	-1.328
T2 A1-C1 H2	Gegham 1	0.000	0.000	0.000	1.000	0.000	0.000	0.000	0.000	0.000	0.000	0.000	0.000	0.000	0.000	-0.096	-8.930	3.334	-4.886	6.093
TR-2 A1-C1 H	Gegham 1	0.000	0.000	0.000	1.000	0.000	0.000	0.000	0.000	0.000	0.000	0.000	0.000	0.000	0.000	0.201	-10.549	3.879	-4.418	2.559
TR-2 A1-C1 H	Gegham 1	0.000	0.000	0.000	1.000	0.000	0.000	0.000	0.000	0.000	0.000	0.000	0.000	0.000	0.000	-1.931	-9.578	2.912	-4.172	5.753
TR-2 A1-C1 H	Gegham 1	0.000	0.000	0.000	1.000	0.000	0.000	0.000	0.000	0.000	0.000	0.000	0.000	0.000	0.000	0.997	-11.517	2.420	-4.895	5.395
TR-2 A1-C1 H	Syunik	0.000	0.000	0.000	0.000	0.000	0.000	0.000	0.000	0.000	0.000	0.000	0.000	1.000	0.000	-7.146	-13.454	-3.666	4.763	-0.923
TR-2 A0-C-0	Gegham 1	0.000	0.000	0.000	1.000	0.000	0.000	0.000	0.000	0.000	0.000	0.000	0.000	0.000	0.000	0.329	-9.577	2.531	-4.532	4.180
TR-2 A0-C-0	Gegham 1	0.000	0.000	0.000	1.000	0.000	0.000	0.000	0.000	0.000	0.000	0.000	0.000	0.000	0.000	0.072	-8.940	4.481	-4.625	4.672
TR2 Unit A1	Gegham 1	0.000	0.000	0.000	1.000	0.000	0.000	0.000	0.000	0.000	0.000	0.000	0.000	0.000	0.000	-1.164	-11.046	3.704	-4.683	2.334
TR2 Unit A1	Gegham 1	0.000	0.000	0.000	1.000	0.000	0.000	0.000	0.000	0.000	0.000	0.000	0.000	0.000	0.000	-0.516	-10.540	2.837	-4.674	3.955
TR2 Unit A1	Gegham 1	0.000	0.000	0.000	1.000	0.000	0.000	0.000	0.000	0.000	0.000	0.000	0.000	0.000	0.000	-1.168	-11.196	2.956	-4.208	2.466
TR2 Unit A1	Gegham 1	0.000	0.000	0.000	1.000	0.000	0.000	0.000	0.000	0.000	0.000	0.000	0.000	0.000	0.000	0.023	-12.349	2.663	-5.124	4.347
TR2 Unit A1	Gegham 1	0.000	0.000	0.000	1.000	0.000	0.000	0.000	0.000	0.000	0.000	0.000	0.000	0.000	0.000	-1.165	-13.400	2.845	-4.603	3.172
TR2 Unit A1	Gegham 1	0.000	0.000	0.000	1.000	0.000	0.000	0.000	0.000	0.000	0.000	0.000	0.000	0.000	0.000	-0.206	-11.775	3.308	-5.064	6.254
TR2 Unit A1	Gegham 1	0.000	0.000	0.000	1.000	0.000	0.000	0.000	0.000	0.000	0.000	0.000	0.000	0.000	0.000	-2.366	-10.021	2.418	-2.556	1.592
TR2 Unit A1	Gegham 1	0.000	0.000	0.000	1.000	0.000	0.000	0.000	0.000	0.000	0.000	0.000	0.000	0.000	0.000	-0.457	-11.997	2.964	-4.539	3.520
TR2 Unit A1	Gegham 1	0.000	0.000	0.000	1.000	0.000	0.000	0.000	0.000	0.000	0.000	0.000	0.000	0.000	0.000	-0.659	-11.391	3.190	-4.674	2.820
TR2 Unit A1	Gegham 1	0.000	0.000	0.000	1.000	0.000	0.000	0.000	0.000	0.000	0.000	0.000	0.000	0.000	0.000	-1.751	-11.864	2.688	-4.337	5.316
TR2 Unit A1	Gegham 1	0.000	0.000	0.000	1.000	0.000	0.000	0.000	0.000	0.000	0.000	0.000	0.000	0.000	0.000	-0.387	-10.010	3.411	-4.001	1.318
TR2 Unit A1	Gegham 1	0.000	0.000	0.000	1.000	0.000	0.000	0.000	0.000	0.000	0.000	0.000	0.000	0.000	0.000	-0.058	-10.169	4.421	-5.216	4.414
TR2 Unit A1	Gegham 1	0.000	0.000	0.000	1.000	0.000	0.000	0.000	0.000	0.000	0.000	0.000	0.000	0.000	0.000	-0.192	-12.261	1.313	-4.593	4.901
TR2 Unit A1	Gegham 1	0.000	0.000	0.000	1.000	0.000	0.000	0.000	0.000	0.000	0.000	0.000	0.000	0.000	0.000	-1.481	-11.545	2.857	-4.357	4.736
TR2 Unit A1	Gegham 1	0.000	0.000	0.000	1.000	0.000	0.000	0.000	0.000	0.000	0.000	0.000	0.000	0.000	0.000	-1.118	-12.716	2.647	-4.898	3.972
TR2 Unit A1	Gegham 1	0.000	0.000	0.000	1.000	0.000	0.000	0.000	0.000	0.000	0.000	0.000	0.000	0.000	0.000	-2.421	-11.045	3.244	-4.602	6.868
TR2 Unit A1	Gegham 1	0.000	0.000	0.000	1.000	0.000	0.000	0.000	0.000	0.000	0.000	0.000	0.000	0.000	0.000	-0.428	-10.321	3.856	-4.815	7.012
TR2 Unit A1	Gegham 1	0.000	0.000	0.000	1.000	0.000	0.000	0.000	0.000	0.000	0.000	0.000	0.000	0.000	0.000	-0.079	-11.232	3.231	-4.394	4.416
TR2 Unit A1	Gegham 1	0.000	0.000	0.000	1.000	0.000	0.000	0.000	0.000	0.000	0.000	0.000	0.000	0.000	0.000	-0.480	-11.200	3.148	-4.664	6.257
TR2 Unit A1	Gegham 1	0.000	0.000	0.000	1.000	0.000	0.000	0.000	0.000	0.000	0.000	0.000	0.000	0.000	0.000	-1.419	-11.705	2.986	-4.710	3.991
TR2 Unit A1	Gegham 1	0.000	0.000	0.000	1.000	0.000	0.000	0.000	0.000	0.000	0.000	0.000	0.000	0.000	0.000	-0.402	-10.092	3.077	-4.714	5.957
TR2 Unit A1	Gegham 1	0.000	0.000	0.000	1.000	0.000	0.000	0.000	0.000	0.000	0.000	0.000	0.000	0.000	0.000	-0.904	-10.849	4.442	-4.155	2.319
TR2 Unit A1	Gegham 1	0.000	0.000	0.000	1.000	0.000	0.000	0.000	0.000	0.000	0.000	0.000	0.000	0.000	0.000	0.590	-9.696	3.263	-5.062	5.195
TR2 Unit A1	Gegham 1	0.000	0.000	0.000	1.000	0.000	0.000	0.000	0.000	0.000	0.000	0.000	0.000	0.000	0.000	-1.007	-10.280	3.516	-4.350	3.802
TR2 Unit A1	Gegham 1	0.000	0.000	0.000	1.000	0.000	0.000	0.000	0.000	0.000	0.000	0.000	0.000	0.000	0.000	-0.139	-11.212	3.506	-4.805	3.474
TR2 Unit A1	Gegham 1	0.000	0.000	0.000	1.000	0.000	0.000	0.000	0.000	0.000	0.000	0.000	0.000	0.000	0.000	0.286	-9.229	3.647	-5.328	6.340
SR2 (TR2?) H	Gegham 1	0.000	0.000	0.000	1.000	0.000	0.000	0.000	0.000	0.000	0.000	0.000	0.000	0.000	0.000	-1.376	-11.105	2.201	-3.813	2.635
TR2 Unit B1	Gegham 1	0.000	0.000	0.000	1.000	0.000	0.000	0.000	0.000	0.000	0.000	0.000	0.000	0.000	0.000	-1.582	-11.128	2.712	-4.238	3.079
TR2 Unit B1	Gegham 1	0.000	0.000	0.000	1.000	0.000	0.000	0.000	0.000	0.000	0.000	0.000	0.000	0.000	0.000	-1.220	-10.610	2.535	-4.460	5.391
